# BIMP‐Catalyzed 1,3‐Prototropic Shift for the Highly Enantioselective Synthesis of Conjugated Cyclohexenones†

**DOI:** 10.1002/anie.202006202

**Published:** 2020-08-07

**Authors:** Jonathan C. Golec, Eve M. Carter, John W. Ward, William G. Whittingham, Luis Simón, Robert S. Paton, Darren J. Dixon

**Affiliations:** ^1^ Department of Chemistry Chemistry Research Laboratory University of Oxford Mansfield Road Oxford OX1 3TA UK; ^2^ Leverhulme Research Centre for Functional Materials Design The Materials Innovation Factory Department of Chemistry University of Liverpool Liverpool L7 3NY UK; ^3^ Jealott's Hill International Research Centre Bracknell Berkshire RG42 6EY UK; ^4^ Facultad de Ciencias Químicas Universidad de Salamanca Plaza de los Caídos 1–5 37008 Salamanca Spain; ^5^ Department of Chemistry Colorado State University 1301 Center Ave Ft. Collins CO 80523-1872 USA

**Keywords:** bifunctional iminophosphoranes, chiral cyclohexenone, asymmetric catalysis, organocatalysis, prototropic shift

## Abstract

A bifunctional iminophosphorane (BIMP)‐catalysed enantioselective synthesis of α,β‐unsaturated cyclohexenones through a facially selective 1,3‐prototropic shift of β,γ‐unsaturated prochiral isomers, under mild reaction conditions and in short reaction times, on a range of structurally diverse substrates, is reported. α,β‐Unsaturated cyclohexenone products primed for downstream derivatisation were obtained in high yields (up to 99 %) and consistently high enantioselectivity (up to 99 % *ee*). Computational studies into the reaction mechanism and origins of enantioselectivity, including multivariate linear regression of TS energy, were carried out and the obtained data were found to be in good agreement with experimental findings.

## Graphical abstract

**Get a shift on**: An enantioselective bifunctional iminophosphorane (BIMP)‐catalysed 1,3‐prototropic shift of structurally diverse β,γ‐unsaturated cyclohexenones is reported. The reaction is high yielding (up to 99 %) in a short time span and proceeds with a high level of selectivity (up to 99 % *ee*) on a wide range of substrates. To complement the experimental data, in‐depth computational studies were undertaken, including multivariate linear regression of TS energy.

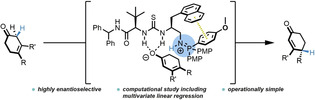

Chiral conjugated cyclohexenones are valuable building blocks for synthesis, offering great versatility across a broad spectrum of reactions and applications.^1^ A number of organocatalytic approaches have been explored to construct such scaffolds in an enantioselective manner, for example through the desymmetrisation of cyclohexadienones or Robinson annulation.^2^ However, a powerful yet underdeveloped approach for their enantioselective synthesis is through the double‐bond migration of their β,γ‐unsaturated prochiral isomers. Such transformations have been found to be catalysed by a number of small molecules and enzymes, and their reaction kinetics have been well‐documented.^3^ Until recently, chemocatalytic methods to accomplish this transformation enantioselectively have proven elusive.^4^ Currently, the approach reported by Lee and Deng for the enantioselective prototropic shift via cooperative Brønsted base/iminium ion catalysis offers the best solution for such a transformation, providing typically excellent yields and good enantioselectivity (Scheme 1 A).^5^ Despite these attributes, the reaction is limited in scope to alkyl/allyl‐substituted substrates at both the α‐ and β‐positions and requires extended reaction times of on average 85 hours. Furthermore, and relevant to the current study, the Deng group reported that cinchona‐derived bifunctional Brønsted base/H‐bond donor catalysts used previously to perform related enantioselective isomerization of butenolides were unable to effect the transformation, owing to the low acidity of the ketone α‐proton.^6^

**Scheme 1:**
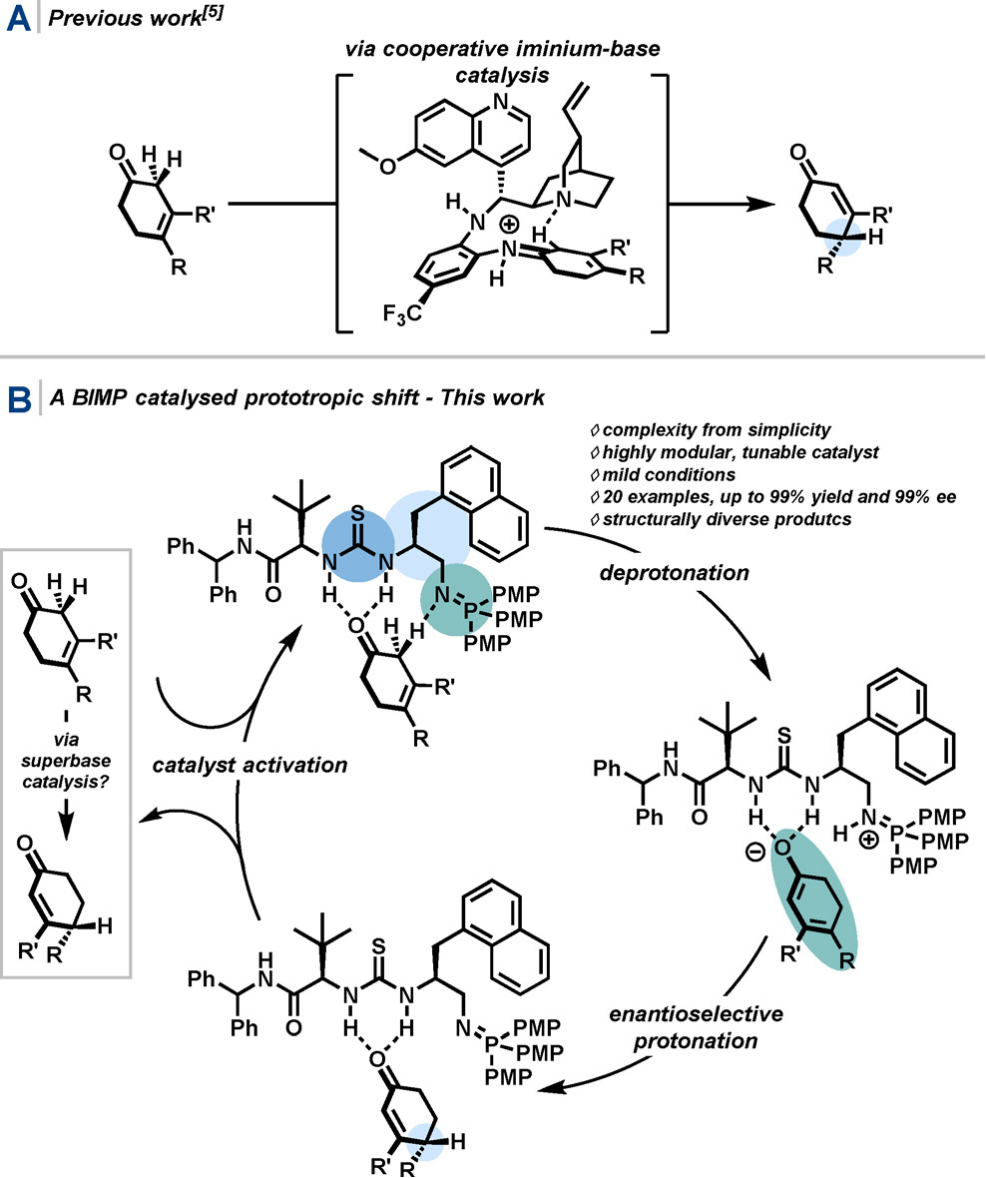
A) Cooperative iminium base catalysed enantioselective 1,3‐prototropic shift of β,γ‐unsaturated cyclohexenones.^5^ B) Conceptual mechanism for a BIMP‐catalysed prototropic shift. PMP=*para*‐methoxy phenyl.

Attracted by the numerous synthetic applications of such an enantioselective transformation, we sought to identify an operationally simple Brønsted base catalysed variant using our highly modular and tuneable bifunctional iminophosphorane (BIMP) superbase catalyst family. BIMP catalysts, like many other bifunctional organocatalysts, combine a Brønsted basic moiety with a hydrogen‐bond donor linked through a chiral scaffold (Scheme 1 B).^7^ They have previously been demonstrated to impart high levels of reactivity and enantiocontrol across a diverse range of reactions including, ketimine nitro‐Mannich reactions, sulfur‐Michael additions, conjugate additions to enone diesters, and (relevant to this work) a cascade heptenone isomerization/enantioselective intramolecular Diels–Alder reaction that was the key step of our group's total synthesis of (−)‐himalensine A.^8^

It was envisaged that in conjunction with the hydrogen‐bond donor group of the catalyst, the superbasic iminophosphorane moiety would provide sufficient activation to deprotonate the weakly acidic α‐position.^6^, ^9^ Kinetic and enantiodetermining reprotonation of the extended enolate would then occur preferentially at the γ‐position in an enantioselective manner to afford the desired cyclohexenone product.^10^ Our aim was to identify a catalyst system that would efficiently deliver excellent levels of enantioselectivity across a wide range of substrates in a short reaction time, and herein we wish to report our findings.

We began our investigation using Hagemann's ester‐derived β,γ‐cyclohexenone **1 a** (see the Supporting Information).^11^ Guided by our previous work, we initially investigated a range of 1^st^ generation BIMP catalysts (**3 a**–**d**), including catalyst **3 a**, which was used in the total synthesis of (−)‐himalensine A.^8^ Each catalyst provided the product in low to moderate yield (17–53 %) with low levels of enantioselectivity (1–14 % *ee*). Notably, more basic P(PMP)_3_‐derived iminophosphoranes performed with improved catalytic activity in comparison with those derived from PPh_3_ (Scheme 2). Accordingly, we turned our attention to P(PMP)_3_‐derived 2^nd^ generation BIMP catalysts, and with catalyst **3 e**, substrate **1 a** underwent the 1,3‐prototropic shift in decent yield (67 %), however enantiocontrol (18 % *ee*) remained poor. Replacing the *tert*‐butyl substituent at stereocentre **a** with methylnaphthyl group to provide catalyst **3 f** unfortunately led to almost complete loss of reactivity and offered no improvement in enantioselectivity. Consequently, the performance of catalyst **3 g**, a stereoisomer of **3 e**, was investigated, which interestingly led to a significant increase in both enantioselectivity (85 % *ee*) and yield (97 %). Two configurationally related catalysts, **3 h** and **3 i**, which possess phenyl and methylnaphthyl groups at stereocentre **b**, respectively, were synthesized and their performance investigated. Impressively, methylnaphthyl‐containing catalyst **3 i** resulted in the formation of **2 a** in near quantitative yield after 24 hours and 99 % *ee*.

**Scheme 2:**
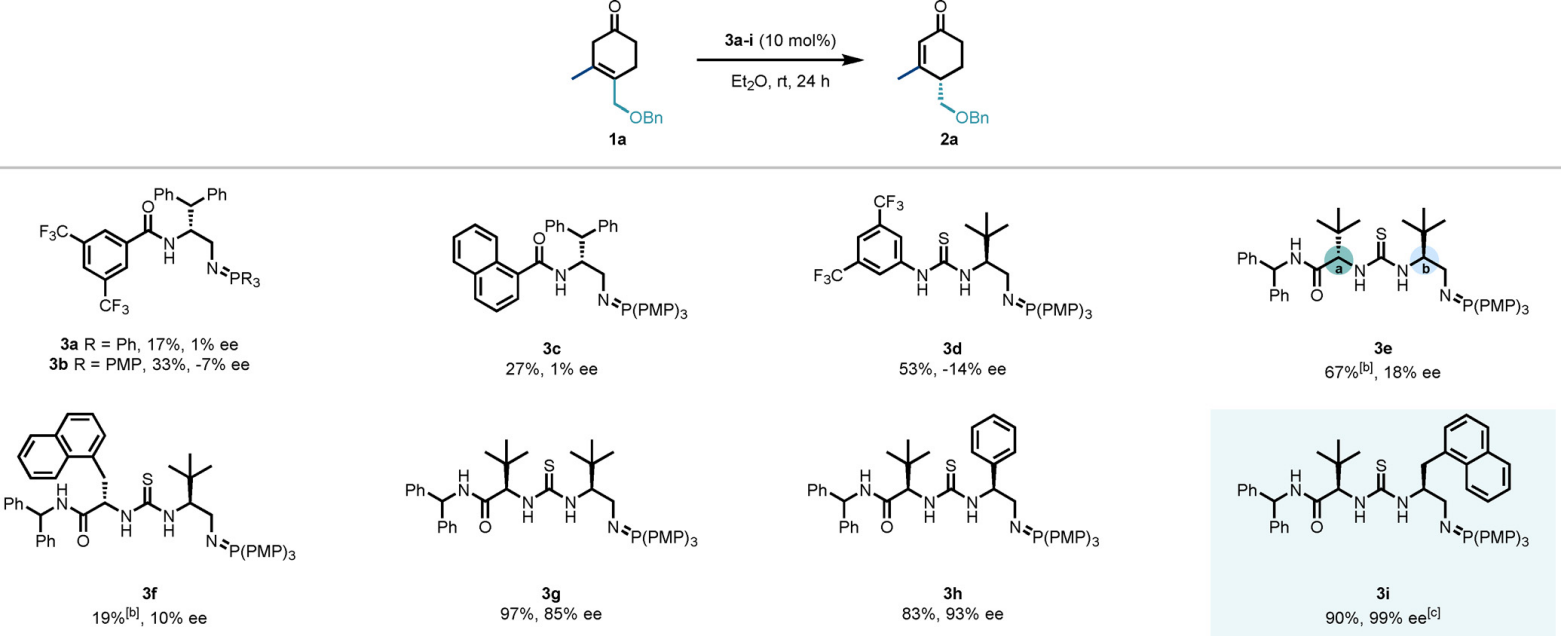
Catalyst optimization.^[a]^ [a] Reactions were carried out with 0.065 mmol of **1 a**. Enantiomeric excess (*ee*) was determined by HPLC analysis on chiral stationary phase. [b] NMR yield. [c] Reaction was carried out with 0.26 mmol of substrate.

With optimal catalyst and conditions identified, the scope of the enantioselective prototropic shift was investigated (Scheme 3 A). Wide variation at the ether substituent was well‐tolerated, with high yields and enantioselectivity (>95 % *ee*) being obtained for products **2 b**–**e**. Almost complete enantiocontrol and conversion to O‐TBS‐protected product **2 f** was observed even upon scale‐up to 1.5 g. Furthermore, unprotected alcohol **1 g** was a viable substrate, providing **2 g** in good yield and 85 % *ee*. We sought to apply our method to the synthesis of a key building block in the construction of both (−)‐reserpine and (−)‐penitrem D, achieved by Stork and co‐workers and Smith et al. respectively (Scheme 3 B).^12^ Isomerization substrate **1 h** was synthesized in a single step using methodology developed by Hilt,^13^ and smoothly underwent the 1,3‐prototropic shift to afford **2 h** in 62 % yield and 94 % *ee*, thereby shortening the previously reported synthesis of **2 h**.^12^

**Scheme 3:**
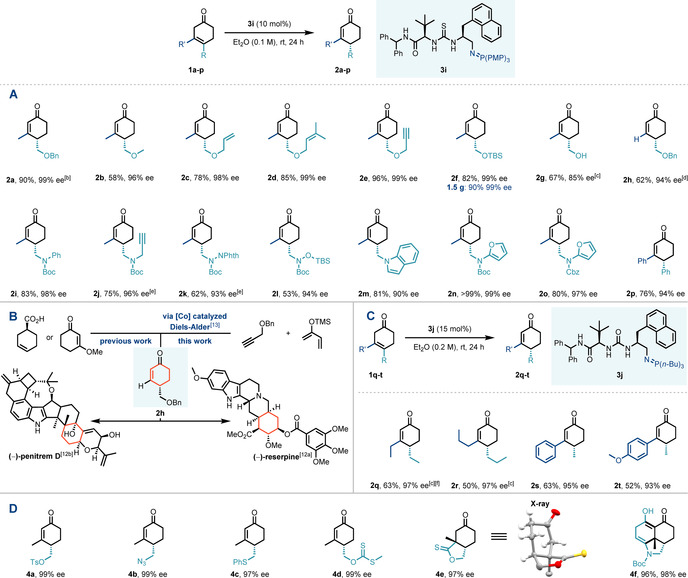
Reaction scope and derivatisation of enantioenriched cyclohexenones.^[a]^ [a] Reaction was carried out with 0.13 mmol of substrate. Enantiomeric excess (*ee*) was determined by HPLC analysis on chiral stationary phase. [b] Reaction carried out with 0.26 mmol of substrate. [c] Reaction carried out with 0.065 mmol of substrate. [d] 30 °C. [e] 48 h. [f] 0.15 M. TBS=*tert*‐butyldimethylsilyl, NPhth=phthalimidate, Cbz=benzyloxycarbonyl, Ts=*para*‐toluenesulfonyl.

In further exploration of the reaction scope, we looked at the effect of pendant‐heteroatom variance on reactivity and selectivity (Scheme 3 A). *N*‐Boc‐protected amines **2 i** and **2 j** were found to perform particularly well in the 1,3‐prototropic shift, with both high yields and high enantioselectivity being obtained in both cases. We turned our attention to more complex amine‐based functionalities to introduce further structural diversity. Accordingly, hydrazine‐ and hydroxylamine‐functionalized substrates **1 k** and **1 l** were synthesized. Both compounds underwent the 1,3‐prototropic shift in high yield and excellent enantioselectivity. Heterocyclic appendages incorporated into the starting material, for example, an indole substituent attached at the δ‐position (**2 m**), performed consistently. Introduction of an amidofuran moiety was easily achieved and subjection to the standard reaction conditions afforded **2 n** and **2 o** in high yield and 99 % and 97 % *ee*, respectively. Pleasingly β,γ‐diphenyl substrate **1 p** performed equally well, with the product **2 p** being obtained in 76 % yield and impressive 94 % *ee*.

A significant drop in reactivity was encountered with β,γ‐diethyl substrate **1 q**. Based on a previous study by Whalen and co‐workers, it was more than likely that the rate‐limiting step of the prototropic shift would show Brønsted base strength dependence.^3^ Thus, to further augment Brønsted base strength, we surveyed a range of iminophosphoranes whilst maintaining the chiral H‐bond‐donor scaffold (see the Supporting Information).^8^ An increase in reactivity with a tributylphosphine‐derived iminophosphorane was observed although the conversion was poor over the standard reaction time and the selectivity decreased significantly (20 % yield, 87 % *ee*).

Pleasingly, switching the hydrogen‐bond donor to a urea group provided the increase in reactivity we desired. After re‐optimization of the reaction conditions we were able to perform the 1,3‐prototropic shift on substrate **1 q** to afford **2 q** in 63 % yield and impressive 97 % *ee*. We also trialed more challenging substrates (Scheme 3 C). β,γ‐Dipropyl substrate **1 r** underwent the prototropic shift in 50 % yield and 97 % *ee*. Replacement of the β‐substituent with a phenyl group provided **2 s** in 63 % yield and 95 % *ee*, and analogous substrate **1 t** with an electron‐rich phenyl ring performed equally well.

Derivatisation of the enantioenriched products was realized through the removal of the TBS group of **2 f** group and activation of the free alcohol through tosylation in high yield, to provide **4 a** in 99 % enantiopurity (Scheme 3 D, see the Supporting Information).^14^ The tosylate could then be used to introduce further functionality, including azide **4 b** and thioether **4 c**, which were obtained in 99 % *ee* and 97 % *ee*, respectively. The free alcohol could also be transformed into xanthate ester **4 d** and subsequently enantiopure cyclic thionolactone **4 e**.^15^ Prolonged heating of **2 n** effected an intramolecular Diels–Alder reaction to afford the stereochemically congested tricyclic scaffold **4 f** with high *ee*.

Having succeeded in the development of an enantioselective Brønsted base catalysed 1,3‐prototropic shift, we then turned our investigation to the mechanistic pathway and origins of enantioselectivity using in‐depth computational analysis. Transition structures (TSs) were located for substrate **1 a** undergoing successive α‐deprotonation and γ‐reprotonation by BIMP catalyst **3 i**, resulting in the Gibbs energy profile shown in Figure 1. The reprotonation TSs are higher in energy, making this the rate‐ and enantio‐determining step.^16^ Along this reaction coordinate, the bifunctional catalyst engages the substrate oxygen with a dual H‐bonding interaction from both thiourea N‐H protons. Consistent with experimental observations, the *S* enantiomer is favoured in this step by 2.2 kcal mol^−1^, which is equivalent to a computed *ee* value of 95 %. Computations also predict that α‐deprotonation will occur reversibly, consistent with deuterium exchange between labelled and unlabelled substrates at the α‐position that we observe experimentally (see the Supporting Information). The catalyst/dienolate ion pair can reversibly dissociate prior to the irreversible protonation taking place.^17^ We performed a systematic conformational analysis of competing TSs, including varied substrate ring conformations and rotations about single bonds. In the preferred TSs, the thiourea binds the substrate oxygen while the iminophosphorane participates as proton acceptor and then donor. Alternative modes of N‐H proton transfer from the catalyst to substrate from the (thio)urea were much higher in energy and are not expected to contribute to the observed reactivity (see the Supporting Information). We located 112 different TS conformers and used statistical modelling to identify the most important structural features that influence their stability. Multivariate linear regression was performed to predict the conformational energy (*R* ^2^ 0.85 (train), 0.80 (test), 0.80 (5‐fold CV)), from which the statistically significant geometric features, automatically selected during model construction, are shown in the Supporting Information.^18^

**Figure 1:**
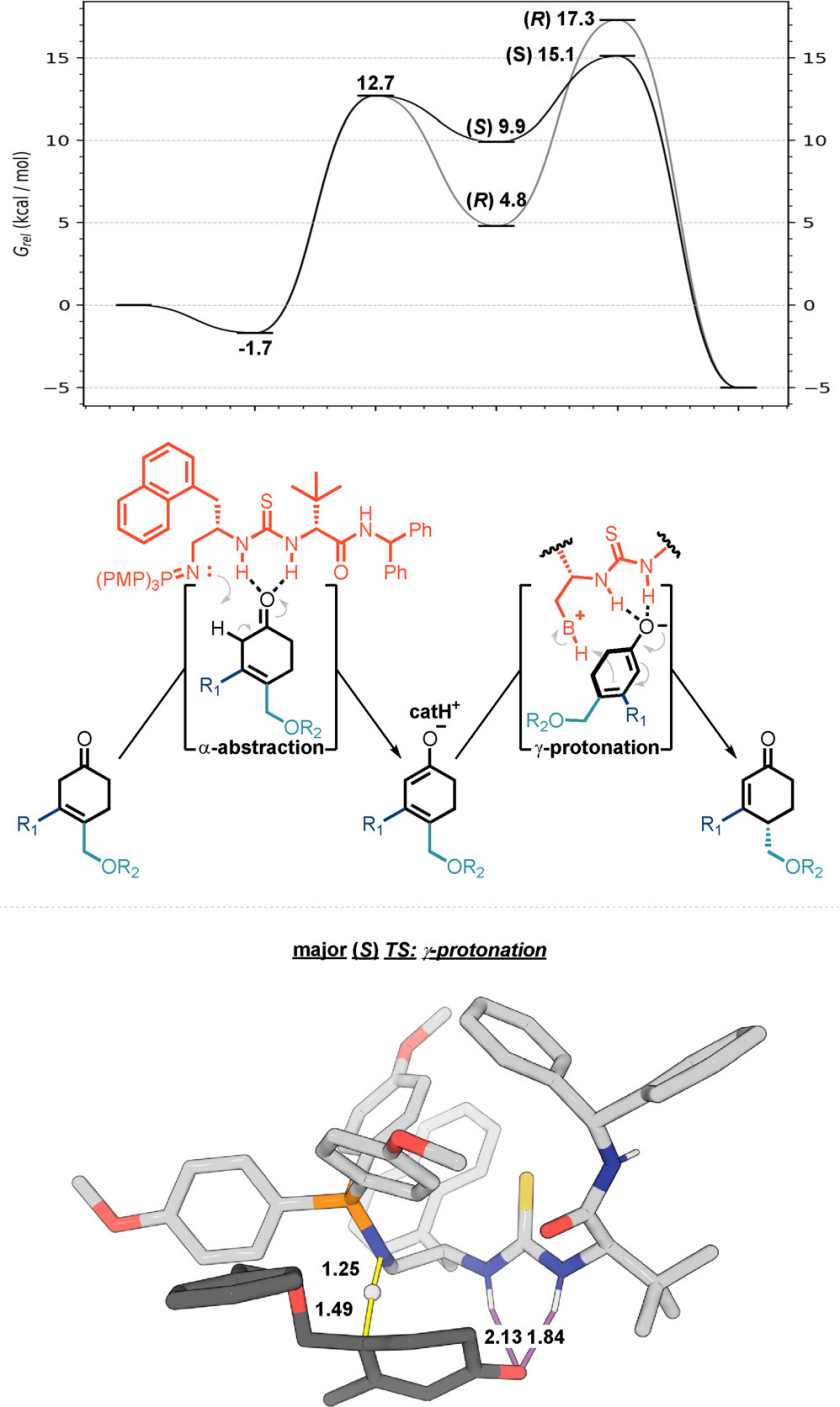
Gibbs energy profile (kcal mol^−1^) showing deprotonation and reprotonation steps (M06‐2X+D3/def2‐TZVP). The most stable major transition structure in the enantiodetermining step is shown (bond lengths in Å).

The substrate conformation is decisive in terms of enantioselectivity. The more favourable (*S*)‐TS has less torsional strain and less 1,3‐allylic strain. As shown in Figure 2 the (*R*)‐TS has greater eclipsing interactions in the ring and, due to the orientation of the alkoxy group, greater A^1,3^‐strain. Indeed, the computed substrate distortion energy^19^ is 1.6 kcal mol^−1^ greater in this TS, which is disfavored (ΔΔ*G* ^≠^) by 2.2 kcal mol^−1^ overall.

**Figure 2:**
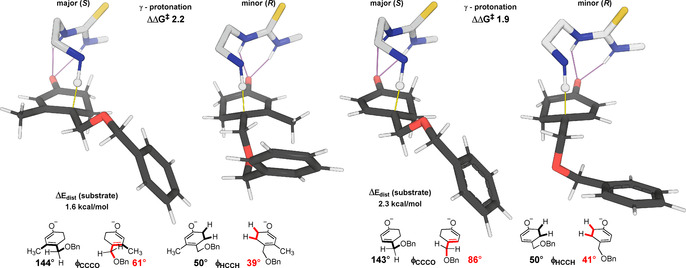
Distortion of β‐methyl substrate **1 a** and unsubstituted **1 h** controls enantioselectivity.

The γ‐substituent plays an important role in influencing enantioselectivity. It must adopt different conformations in response to the catalyst (principally to avoid clashes with the P‐substituents) protonating either enantioface. As a consequence, substrate conformational strain dictates the sense of enantioselectivity, rather than significant differences in the noncovalent interactions between substrate and catalyst. From these findings, we can predict that a flexible γ‐substituent is helpful for high levels of enantioselectivity since this creates the potential for differential allylic strain between the two pathways. Furthermore, it also follows that although a β‐substituent is not essential for enantioselectivity, its absence will reduce allylic strain in the TS. Accordingly, we compute a reduced ΔΔ*G* ^≠^ of 1.9 kcal mol^−1^ (92 % *ee*) for substrate **2 h**.

In summary, we have developed a new Brønsted base catalysed 1,3‐prototropic shift for the synthesis of enantioenriched functionalized cyclohexenones using our BIMP family of catalysts and have investigated the mechanistic pathway and origins of enantioselectivity in detail using DFT. The isomerization was found to proceed in high yield within a short time frame and demonstrates impressive levels of enantioselectivity across a range of functionally interesting substrates, which could be further derivatized to introduce more diversity and functionality. The catalyst itself is shown to be versatile enough to overcome reactivity issues through the modification of its Brønsted base strength, whilst maintaining good enantiocontrol; a design feature we hope to exploit in other challenging synthetic transformations.

## Conflict of interest

The authors declare no conflict of interest.

## Supporting information

As a service to our authors and readers, this journal provides supporting information supplied by the authors. Such materials are peer reviewed and may be re‐organized for online delivery, but are not copy‐edited or typeset. Technical support issues arising from supporting information (other than missing files) should be addressed to the authors.

Supplementary
