# Supplementary material for: BIMP‐Catalyzed 1,3‐Prototropic Shift for the Highly Enantioselective Synthesis of Conjugated Cyclohexenones
Source: Angew Chem Int Ed Engl. 2020 Aug 7;59(40):17417–22. doi: 10.1002/anie.202006202 (PMC7540019; doi:10.1002/anie.202006202)
Supplement: Supplementary file 1 — Supplementary [file ANIE-59-17417-s001.pdf]

Angewandte Chemie
Eine Zeitschrift der Gesellschaft Deutscher Chemiker

Supporting Information

BIMP-Catalyzed 1,3-Prototropic Shift for the Highly Enantioselective Synthesis of Conjugated Cyclohexenones**

Jonathan C. Golec, Eve M. Carter, John W. Ward, William G. Whittingham, Luis Simón, Robert S. Paton, and Darren J. Dixon**

anie_202006202_sm_miscellaneous_information.pdf

Supplementary information

Table of contents

1. Supplementary methods……………………………………………………………………1

1.1 General information…………………………………………………………………...2

1.2 Synthesis of substrates for use in the BIMP catalysed prototropic shift………………3

1.2.1 Substrates prepared from Hagemann's ester……………..………………………7

1.2.2 Substrates prepared via cobalt catalysed Diels-Alder reaction……….……………36

1.3 Optimisation of reaction conditions……………………………………………..42

1.4 The BIMP catalysed enantioselective prototropic shift of β,γ-unsaturated cyclohexenones ………………………………………………………………………45

1.5 Derivitisation of enantioenriched products……………..…………………………..66

1.6 Synthesis of catalysts………………..……………………………………..73

1.7 Deuterium labelling study…………………………..…………………………..81

2. Supplementary Data

2.1 Single Crystal X-Ray Diffraction Data………………………………………………90

2.2 Copies of NMR spectra……………………………………………………………….93

2.3 Copies of HPLC traces………………………………..………………………159

2.4 Computational data…………………………………………………………………194

3. References……...…………………...………..…………………………………..………..208

1.1 General information

Atmosphere

All reactions were carried out under an atmosphere of Ar unless otherwise stated.

Solvents and Reagents

Moisture sensitive reactions were carried out using solvents obtained from the MBRAUN-SPS solvent purification system (CH_2Cl_2, THF, pentane, toluene, DMF, Et_2O) and often dried over 3Å molecular sieves. Reactions that were not deemed moisture sensitive were carried out using solvents taken from Winchester bottles. Reagents were purchased at reagent-grade from Acros Organics, Sigma-Aldrich, Alfa Aeser, and Fluorochem and used without further purification.

Chromatography

TLC analysis was carried out using Merck aluminium backed DC60 F254 plates (particle size 0.2 mm). UV light was used to visualise spots which were often stained with $KMnO_4$, vanillin or ninhydrin depending on the compound. Flash column chromatography was carried out using Sigma Aldrich silicagel 60 Å (particle size 43-60 μm) with the indicated solvent system.

Data

Proton (^1H) and carbon (^{13}C) spectra were recorded on Bruker DPX200 (200 MHz) Bruker AVG400 (400/101 MHz), Bruker AVH400 (400/101 MHz), Bruker AVF400 (400/101 MHz), Bruker AVC500 (500/126 MHz), Bruker AVB500 (500/126 MHz), Bruker AVX500 (500/126 MHz) and Bruker AV600 (600/151) NMR spectrometers. Spectra were referenced with respect to the residual solvent peak ($CHCl_3$: δ_H 7.26, δ_C 77.16 ppm; C_6D_5H: δ_H 7.16, δ_C 128.06 ppm; CD_3COCD_2H: δ_H 2.05, δ_C 29.84 ppm; CD_2HCN: δ_H 1.94, δ_C 1.32 ppm; $C_6D_4HCD_3$: δ_H 7.09, δ_C 125.13 ppm; CHD_2OD: δ_H 3.31, δ_C 49). Peak assignments were made based on chemical shifts, integrations, coupling constants, 2-D COSY and NOSY, HMBC, HSQC, nOe and NOESY. Peak multiplicities are described as singlet (s), doublet (d), triplet (t), pentet (p), or a combination e.g. doublet of doublets, or as a multiplet over a peak range. Some peaks are described as broad (b). Coupling constants are reported to the nearest 0.5 Hz and ^{13}C chemical shifts are given to the nearest 0.1 ppm.

High-resolution mass spectra (ESI) were recorded using a Bruker µTOF mass spectrometer.

Infrared spectra (IR) were recorded using a Bruker Tensor 27 FT-IR spectrometer as a thin film or powder sample. Selected maximum absorbances were reported in ν_{max} (cm^{-1}).

Melting points (MP) were obtained using a Leica Galen III Hot-stage melting point apparatus and microscope and on a Kofler hot block and are reported uncorrected.

1.2 Synthesis of substrates for use in the enantioselective prototropic shift

Procedure for the synthesis of (4,4-dimethoxy-2-methylcyclohex-1-en-1-yl)methanol (S2)

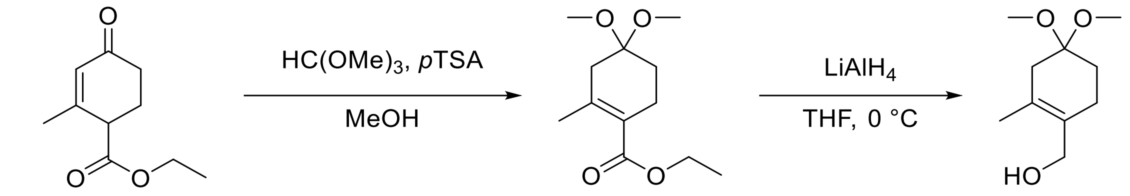

(**Hagemann's ester**) (**S1**) (**S2**)

ethyl 4,4-dimethoxy-2-methylcyclohex-1-ene-1-carboxylate (S1)

i. To a solution of ethyl-2-methyl-4-oxo-2-cyclohexenecarboxylate (Hagemann's ester) (10.0 g, 54.9 mmol, 1.00 eq) in MeOH (305 ml) was added trimethyl orthoformate (18.0 ml, 165 mmol, 2.99 eq) followed by *p*TsOH (2.09 g, 11.0 mmol, 0.200 eq). The reaction was stirred overnight at room temperature at which point completion was observed by TLC analysis (Pentane/EtOAc = 95/5). The reaction was quenched with sat. aq. K_2CO_3 (50 ml). The aqueous layer was extracted with EtOAc (3 x 50 ml). The combined organic phases were washed with brine, dried over Na_2SO_4, filtered, and the solvent was removed *in vacuo*. The crude mixture was purified by silica gel column chromatography (Pentane/EtOAc = 99/1 to 98/2) to afford ethyl 4,4-dimethoxy-2-methylcyclohex-1-ene-1-carboxylate as a yellow oil in 61% yield (7.70 g) (The product may also be taken on crude to the next step).

^{1}H NMR (500 MHz, $CDCl_3$) δ(ppm): 4.18 (q, J = 7.0 Hz, 2H, $C\underline{H_2}CH_3$), 3.23 (s, 6H, $C(OC\underline{H_3})_2$), 2.43 – 2.29 (m, 4H, $C\underline{H_2}CH_2C(OCH_3)_2$ and $C\underline{H_2}CCH_3$), 2.02 (bs, 3H, $C\underline{H_3}C$=C), 1.86 – 1.77 (m, 2H, $CH_2C\underline{H_2}C(OCH_3)_2$), 1.29 (t, J = 7.0 Hz, 3H, $CH_2C\underline{H_3}$).

^{13}C NMR (126 MHz, $CDCl_3$) δ(ppm): 168.5 ($\underline{C}$=O), 142.7 (C=$\underline{C}CH_3$), 123.9 ($\underline{C}$=CCH_3), 99.1 ($\underline{C}(OCH_3)_2$), 60.2 ($O\underline{C}H_2$), 48.1 ($O\underline{C}H_3$), 42.5 ($\underline{C}H_2CCH_3$), 28.4 ($CH_2\underline{C}H_2C(OCH_3)_2$), 24.8 ($\underline{C}H_2CH_2C(OCH_3)_2$), 21.8 ($C\underline{C}H_3$), 14.5 ($CH_2\underline{C}H_3$).

IR (film) ν_{max}/cm^{-1}: 2941 (C-H), 1711 (C=O), 1645 (C=C), 1245, 1127 (C-O).

HRMS (ES+) exact mass calculated for $[M+Na]^+$ $(C_{12}H_{20}NaO_4)^+$ requires *m/z* 251.1254, found *m/z* 251.1254.

(4,4-dimethoxy-2-methylcyclohex-1-en-1-yl)methanol (S2)

ii. To a stirred solution of $LiAlH_4$ (3.00 eq, 1.99 g, 52.6 mmol) in THF (29 ml) at 0 °C was added a solution of ethyl 4,4-dimethoxy-2-methylcyclohex-1-ene-1-carboxylate (**S1**) (1.00 eq, 4.00 g, 17.5 mmol) in THF (29 ml). The reaction was stirred at 0 °C and allowed to warm to room temperature over 2 h at which point completion was observed by TLC analysis (Pentane/EtOAc = 95/5). The reaction was diluted with Et_2O (40 ml) and cooled to 0 °C. H_2O (2 ml) was added slowly, followed by 15% aq. NaOH (2 ml) then H_2O (6 ml). The suspension was allowed to warm to room temperature with constant stirring over 15 min. $MgSO_4$ was added and stirred for a further 15 min at which point the suspension was filtered. The solvent was removed *in vacuo* and the crude mixture purified by silica gel column chromatography (Et_2O) to afford (4,4-dimethoxy-2- methylcyclohex-1-en-1-yl)methanol as a yellow oil in 79% yield (3.26 g).

^{1}H NMR (400 MHz, $CDCl_3$) δ(ppm): 4.11 (s, 2H, C$\underline{H_2}$OH), 3.21 (s, 6H, C(OC$\underline{H_3}$)$_2$), 2.25 – 2.15 (m, 4H, CH_2C$\underline{H_2}$C(OCH_3)$_2$ and CC$\underline{H_2}$C(OCH_3)$_2$), 1.86 – 1.76 (m, 2H, C$\underline{H_2}$$CH_2$C($OCH_3$)$_2$), 1.69 (s, 3H, CC$\underline{H_3}$), 1.57 (bs, 1H, O$\underline{H}$).

^{13}C NMR (101 MHz, $CDCl_3$) δ(ppm): 129.4 ($\underline{C}$$CH_2$OH), 127.4 ($\underline{C}$$CH_3$), 99.7 ($\underline{C}$($OCH_3$)$_2$), 62.4 ($\underline{C}$$H_2$OH), 48.0 (C(O$\underline{C}$$H_3$)$_2$), 40.8 (C$\underline{C}$$H_2$C($OCH_3$)$_2$), 28.8 ($\underline{C}$$H_2$$CH_2$C($OCH_3$)$_2$), 25.8 ($CH_2$$\underline{C}$$H_2$C($OCH_3$)$_2$), 18.8 (C$\underline{C}$$H_3$).

IR (film) ν_{max}/cm^{-1}: 3398 (O-H), 2939, 2915, 2829 (C-H), 1679 (C=C), 1127, 1049 (C-O).

HRMS (ES+) exact mass calculated for $[M+Na]^+$ ($C_{10}H_{18}NaO_3$) requires *m/z* 209.1148, found *m/z* 209.1150.

General procedure A:

For the addition of nucleophiles to (4,4-dimethoxy-2-methylcyclohex-1-en-1-yl)methanol (S2)

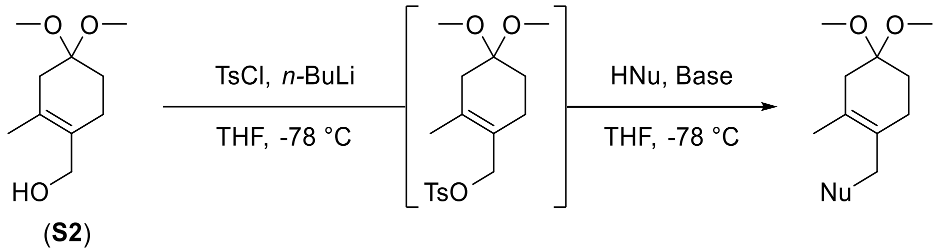

To a stirred solution of (4,4-dimethoxy-2-methylcyclohex-1-en-1-yl)methanol (**S2**) (1.00 eq) in THF (0.54 M) is added *n*-BuLi (1.07 eq, 2.5 M in hexanes) at -78 °C. The reaction was stirred for 15 min at which point a solution of tosyl chloride (1.05 eq) in THF (0.27 M wrt. alcohol) was added. The

solution was stirred for a further 30 min at -78 °C. A pre-prepared solution of the desired nucleophile (2.00 eq) and base (2.00 eq) in THF (0.27 M wrt. alcohol) was then added dropwise to the tosylate. The solution was then warmed to 0 °C and stirred until deemed complete by TLC analysis. The reaction mixture was quenched with sat. aq. K_2CO_3 solution and extracted with Et_2O three times. The combined organic phases were washed with H_2O and brine, dried over $MgSO_4$, and filtered. To the organic phase was added solid K_2CO_3 and the solvent was removed under a stream of N_2. The resultant residue was purified by silica gel column chromatography to yield the desired product.

*Compounds synthesised by this method were found to be extremely sensitive to acidic conditions (NMR spectra obtained in $CDCl_3$ often resulted in rapid decomposition) as well as heat (solvent was often removed under a stream of nitrogen rather than *in vacuo*).

General procedure B:

For the deprotection of acid sensitive substrates

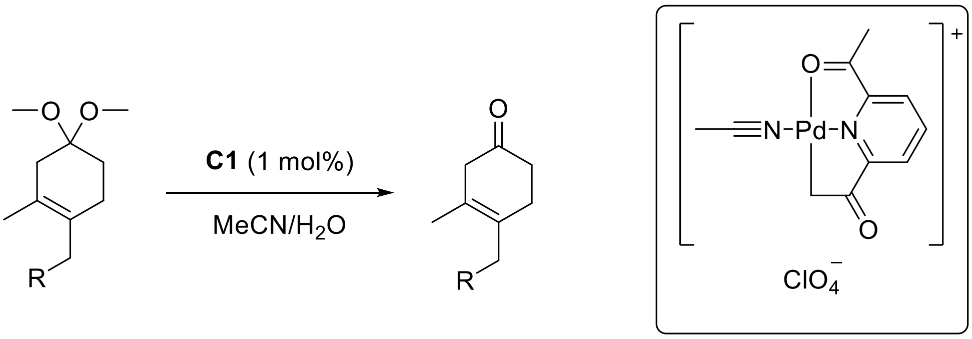

C1

According to a literature procedure,[1a-b] to a stirred solution of the desired protected substrate in wet acetonitrile (0.1 M) was added **C1** (0.01 eq.). The reaction was stirred until deemed complete by TLC analysis and additional catalyst was added if necessary. The reaction mixture was transferred directly onto silica gel and purified by silica gel column chromatography to yield the desired product.

General procedure C:

For the deprotection of substrates not sensitive to acid

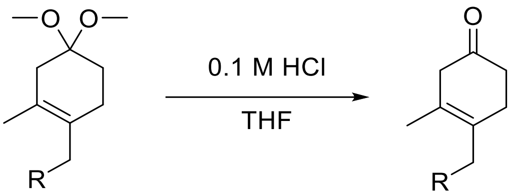

To a stirred solution of the desired protected substrate in THF (0.14 M) was added an equal volume of 0.1 M HCl. The solution was stirred until deemed complete by TLC analysis at which point the reaction was quenched with sat. aq. K_2CO_3 solution. The aqueous phase was extracted with Et_2O and the combined organic phases were washed with brine, dried over Na_2SO_4, filtered and concentrated *in vacuo*. The crude products were then purified by silica gel column chromatography to yield the desired products.

1.2.1 Substrates prepared from Hagemann's ester

(((4,4-dimethoxy-2-methylcyclohex-1-en-1-yl)methoxy)methyl)benzene (S3)

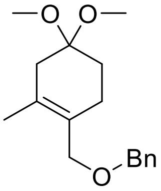

To a suspension of 60% NaH (0.215 g, 5.37 mmol, 2.00 eq) in THF (3.0 ml) at 0 °C was added a solution of (4,4-dimethoxy-2-methylcyclohex-1-en-1-yl)methanol (**S2**) (500 mg, 2.69 mmol, 1.00 eq) in THF (3.7 ml). The suspension was stirred for 10 min at which point benzyl bromide (0.48 ml, 4.03 mmol, 1.50 eq) was added followed by TBAI (99.0 mg, 0.270 mmol, 0.100 eq). The reaction was stirred until completion was observed by TLC analysis (Et_2O). The reaction was quenched with sat. aq. NH_4Cl (10 ml) and extracted with EtOAc (3 x 30 ml). The combined organic extracts were washed with brine, dried over Na_2SO_4, filtered, and the solvent was removed *in vacuo*. The crude mixture was purified by silica gel column chromatography (pentane/EtOAc = 9/1) to afford the title compound as a yellow oil in 77% yield (575 mg).

^{1}H NMR (400 MHz, $CDCl_3$) δ(ppm): 7.33 – 7.17 (m, 5H, Ar$\underline{H}$), 4.39 (s, 2H, C$\underline{H}_2$Ph), 3.94 (s, 2H, C$\underline{H}_2$OCH_2Ph), 3.16 (s, 6H, C(OC$\underline{H}_3$)$_2$), 2.18 (td, *J* = 2.0, 1.0 Hz, 2H, CC$\underline{H}_2$C(OCH_3)$_2$), 2.13 (ddt, *J* = 6.5, 4.5, 2.0 Hz, 2H, CH_2C$\underline{H}_2$C(OCH_3)$_2$), 1.77 (t, *J* = 6.5, 2H, (C$\underline{H}_2$CH_2C(OCH_3)$_2$), 1.66 – 1.56 (m, 3H, CC$\underline{H}_3$).

^{13}C NMR (101 MHz, $CDCl_3$) δ(ppm): 138.8 (Ar$\underline{C}$), 128.5 ($\underline{C}$CH_2OBn) , 128.5 (Ar$\underline{C}$H), 127.9 (Ar$\underline{C}$H), 127.6 (Ar$\underline{C}$H), 127.0 ($\underline{C}$CH_3), 99.7 ($\underline{C}$(OCH_3)$_2$), 71.9 ($\underline{C}H_2$Ph), 69.3 ($\underline{C}H_2$O), 48.0 (O$\underline{C}H_3$), 40.9 ($\underline{C}H_2$CCH_3), 28.7 ($\underline{C}H_2$CH_2C(OCH_3)$_2$), 26.0 (C$H_2\underline{C}H_2$C(OCH_3)$_2$), 19.1 (C$\underline{C}H_3$).

IR (film) v_{max}/cm^{-1}: 3030, 2936, 2856, 2827 (C-H), 1095, 1053 (C-O).

HRMS (ES+) exact mass calculated for $[M+Na]^+$ $(C_{17}H_{24}NaO_3)^+$ requires *m/z* 299.1617, found *m/z* 299.1618.

4,4-dimethoxy-1-(methoxymethyl)-2-methylcyclohex-1-ene (S4)

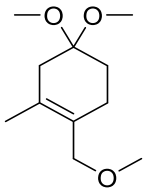

To a solution of (4,4-dimethoxy-2-methylcyclohex-1-en-1-yl)methanol (**S2**) (200 mg, 1.07 mmol, 1.00 eq) in DMF (1.10 ml) at 0 °C was added 60% NaH (64.4 mg, 1.61 mmol, 1.50 eq). The solution was stirred for 15 min at which point methyl iodide (0.130 ml, 2.15 mmol, 2.00 eq) was added. Additional DMF (0.50 ml) was added to aid solubility. The reaction was stirred overnight, quenched with sat. aq. NH_4Cl (10 ml) and extracted with Et_2O (3 x 10 ml). The combined organic layers were washed with H_2O (3 x 10 ml), brine, dried over Na_2SO_4, filtered and concentrated *in vacuo*. The crude product was purified by silica gel column chromatography (pentane/Et_2O = 95/5 to 90/10) to provide the title compound as a colourless oil in 68% yield (145 mg).

^{1}H NMR (500 MHz, Benzene-d_6) δ(ppm): 3.83 (s, 2H, C$\underline{H_2}$O), 3.11 (s, 3H, C$\underline{H_3}$OCH_2), 3.08 (s, 6H, C(OC$\underline{H_3}$)$_2$), 2.28 – 2.22 (m, 2H, CH_2C$\underline{H_2}$C=CCH_3), 2.27 – 2.22 (m, 2H, C$\underline{H_2}$CCH_3), 1.83 (t, *J* = 6.5, 2H, C$\underline{H_2}$$CH_2$C=C$CH_3$), 1.53 (s, 3H, CC$\underline{H_3}$).

^{13}C NMR (126 MHz, Benzene-d_6) δ(ppm): 127.7 (one of $\underline{C}$=C), 127.6 (one of C=$\underline{C}$), 99.8 ($\underline{C}$(OCH_3)$_2$), 71.8 ($\underline{C}H_2$OCH_3), 57.3 (CH_2O$\underline{C}H_3$), 47.5 (C(O$\underline{C}H_3$)$_2$), 40.9 ($\underline{C}H_2$CCH_3), 29.3 ($\underline{C}H_2$$CH_2$C=C$CH_3$), 26.4 ($CH_2$$\underline{C}H_2$C=C$CH_3$), 18.8 (C$\underline{C}H_3$).

IR (film) ν_{max}/cm^{-1}: 2921, 2826 (C-H), 1681 (C=C), 1130 (C-O).

HRMS (ES+) exact mass calculated for $[M+Na]^+$ $(C_{11}H_{20}NaO_3)^+$ requires *m/z* 223.1316, found *m/z* 223.1305.

1-((allyloxy)methyl)-4,4-dimethoxy-2-methylcyclohex-1-ene (S5)

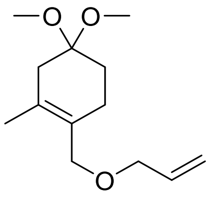

To a solution of (4,4-dimethoxy-2-methylcyclohex-1-en-1-yl)methanol (**S2**) (100 mg, 0.540 mmol, 1.00 eq) in THF (1.20 ml) at 0 °C was added 60% NaH (23.6 mg, 0.590 mmol, 1.10 eq). The resulting suspension was stirred for 30 min at which point TBAI (10.0 mg, 27 μmol, 0.05 eq) and allyl bromide (51.0 μl, 0.590 mmol, 1.10 eq) were added. The reaction was allowed to warm to room temperature and stirred overnight at which point H_2O (10 ml) was added. The aqueous layer was extracted with EtOAc (3 x 20 ml). The combined extracts were washed with H_2O (20 ml), brine, dried over Na_2SO_4, and the solvent was removed *in vacuo*. The crude reaction mixture was purified by silica gel column chromatography (pentane/Et_2O = 90/10) to afford the title compound as yellow oil in 53% yield (64.6 mg).

^{1}H NMR (500 MHz, Acetone-d_6) δ(ppm): 5.90 (ddt, *J* = 17.5, 10.5, 5.5 Hz, 1H, $C\underline{H}=CH_2$), 5.24 (dq, *J* = 17.5, 2.0 Hz, 1H, one of $C=C\underline{H_A}H_B$), 5.10 (dq, *J* = 10.5, 1.5 Hz, 1H, one of $C=CH_A\underline{H_B}$), 3.95 (s, 2H, $C\underline{H_2}OCH_2CH$), 3.88 (dt, *J* = 5.5, 1.5 Hz, 2H, $OC\underline{H_2}CH$), 3.15 (s, 6H, $C(OC\underline{H_3})_2$), 2.18 (s, 2H, $CH_3CC\underline{H_2}$), 2.10 (ddt, *J* = 7.0, 4.5, 2.0 Hz, 2H, $C\underline{H_2}CH_2C(OCH_3)_2$), 1.74 (t, *J* = 6.5 Hz, 2H, $CH_2C\underline{H_2}C(OCH_3)_2$), 1.65 (s, *J* = 2.0 Hz, 3H, $CC\underline{H_3}$).

^{13}C NMR (126 MHz, Acetone-d_6) δ(ppm): 136.7 ($\underline{C}H=CH_2$), 128.3 ($\underline{C}CH_3$), 127.8 ($\underline{C}=CCH_3$), 116.1 ($CH=\underline{C}H_2$), 100.1 ($\underline{C}(OCH_3)_2$), 70.9 ($\underline{C}H_2CH=CH_2$), 69.7 ($\underline{C}H_2OCH_2CH=CH_2$), 47.7 ($C(O\underline{C}H_3)_2$), 41.2 ($C\underline{C}H_2C(OCH_3)_2$), 29.5 ($CH_2\underline{C}H_2C(OCH_3)_2$), 26.6 ($\underline{C}H_2CH_2C(OCH_3)_2$), 18.9 ($C\underline{C}H_3$).

IR (film) v_{max}/cm^{-1}: 2937, 2828 (C-H), 1647 ($C=CH_2$), 1095 (C-O).

HRMS (ES+) exact mass calculated for $[M+Na]^+$ $(C_{13}H_{22}NaO_3)^+$ requires *m/z* 249.1461, found *m/z* 249.1461.

4,4-dimethoxy-2-methyl-1-(((3-methylbut-2-en-1-yl)oxy)methyl)cyclohex-1-ene (S6)

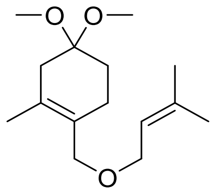

To a stirred solution of (4,4-dimethoxy-2-methylcyclohex-1-en-1-yl)methanol (**S2**) (500 mg, 2.68 mmol, 1.00 eq) in THF (2.7 ml) at 0 °C was added 60% NaH (118 mg, 2.95 mmol, 1.10 eq). The suspension was stirred for 30 min and allowed to warm to room temperature over a further 30 min. The suspension was cooled back down to 0 °C and 3,3-dimethylallyl bromide (0.310 ml, 2.68 mmol, 1.00 eq) was added dropwise. The reaction was stirred overnight and quenched with sat. aq. NH_4Cl (5 ml). The aqueous phase was extracted with Et_2O (3 x 30 ml) and the organic phases were combined, washed with brine, dried over Na_2SO_4, filtered and concentrated *in vacuo*. The crude product was purified by silica gel column chromatography (pentane/Et_2O = 9/1) to provide the title compound as a colourless oil in 70% yield (475 mg).

^{1}H NMR (600 MHz, $CDCl_3$) δ(ppm): 5.52 (tp, *J* = 6.5, 1.5 Hz, 1H, $C\underline{H}=C(CH_3)_2$), 3.96 (s, 2H, $C\underline{H_2}OCH_2CH=C(CH_3)_2$), 3.94 (d, *J* = 6.5 Hz, 2H, $CH_2OC\underline{H_2}CH=C(CH_3)_2$), 3.09 (s, 6H, $C(OC\underline{H_3})_2$), 2.33 – 2.27 (m, 2H, $C\underline{H_2}CH_2C(OCH_3)_2$), 2.22 (s, 2H, $CC\underline{H_2}C(OCH_3)_2$), 1.85 (t, *J* = 6.5 Hz, 2H, $CH_2C\underline{H_2}C(OCH_3)_2$), 1.60 (s, 3H, one of $CH=C(C\underline{H_3})_A(CH_3)_B$), 1.57 (s, 3H, $C\underline{H_3}CCH_2C(OCH_3)_3$), 1.52 (s, 3H, one of $CH=C(CH_3)_A(C\underline{H_3})_B$).

^{13}C NMR (151 MHz, $CDCl_3$) δ(ppm): 135.3 ($\underline{C}(CH_3)_2$), 128.4 ($C=\underline{C}(CH_2)(CH_2O)$), 127.4 ($\underline{C}=C(CH_2)(CH_2O)$), 123.0 ($\underline{C}H=C$), 99.9 ($\underline{C}(OCH_3)_2$), 69.4 ($C=C(CH_2)(\underline{C}H_2O)$), 66.4 ($O\underline{C}H_2CH=C$), 47.5 ($C(O\underline{C}H_3)_2$), 41.0 ($C\underline{C}H_2C(OCH_3)_2$), 29.4 ($CH_2\underline{C}H_2C(OCH_3)_2$), 26.5 ($\underline{C}H_2CH_2C(OCH_3)_2$), 25.7 (one of $CH=C(\underline{C}H_3)_A(CH_3)_B$), 18.9 ($\underline{C}H_3CCH_2C(OCH_3)_2$), 18.0 (one of $CH=C(CH_3)_A(\underline{C}H_3)_B$).

IR (film) ν_{max}/cm^{-1}: 2914 (C-H), 1677 (C=C), 1096, 1054 (C-O).

HRMS (ES+) exact mass calculated for $[M+Na]^+$ $(C_{15}H_{26}NaO_3)^+$ requires *m/z* 277.1774, found *m/z* 277.1774.

4,4-dimethoxy-2-methyl-1-((prop-2-yn-1-yloxy)methyl)cyclohex-1-ene (S7)

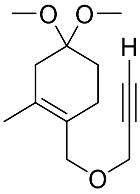

According to a modified literature procedure,[2] 60% NaH (171 mg, 4.30 mmol, 4.00 eq) was added to a stirred solution of (4,4-dimethoxy-2-methylcyclohex-1-en-1-yl)methanol (**S2**) (200 mg, 1.07 mmol, 1.00 eq) in DMF (6.90 ml) at 0 °C. The solution was stirred for 1 h at which point propargyl bromide (80% in toluene, 0.460 ml, 4.30 mmol, 4.00 eq). The reaction was allowed to warm to room temperature over 12 h at which point H_2O (10 ml) was added. The aqueous layer was extracted with EtOAc (3 x 20 ml). The combined extracts were washed with H_2O (3 x 20 ml), brine, dried over Na_2SO_4 and the solvent was removed *in vacuo*. The crude reaction mixture was purified by silica gel column chromatography (pentane/Et_2O = 85/15) to afford the title compound as a colourless oil in 78% yield (188 mg).

^{1}H NMR (500 MHz, Acetone-d_6) δ(ppm): 4.06 (d, *J* = 2.5 Hz, 2H, $C\underline{H}_2C{\equiv}C$), 4.03 (s, 2H, $C\underline{H}_2OCH_2C{\equiv}C$), 3.15 (s, 6H, $C(OC\underline{H}_3)_2$), 2.91 (t, *J* = 2.5 Hz, 1H, $C{\equiv}C\underline{H}$), 2.19 (s, 2H, $CC\underline{H}_2C(OCH_3)_2$), 2.08 (m, 2H, $C\underline{H}_2CH_2C(OCH_3)_2$), 1.74 (t, *J* = 6.5 Hz, 2H, $CH_2C\underline{H}_2C(OCH_3)_2$), 1.69 (s, 3H, $CC\underline{H}_3$).

^{13}C NMR (126 MHz, Acetone-d_6) δ(ppm): 128.7 ($\underline{C}CH_3$), 126.1 ($\underline{C}{=}CCH_3$), 99.2 ($\underline{C}(OCH_3)_2$), 80.4 ($\underline{C}{\equiv}CH$), 74.6 ($C{\equiv}\underline{C}H$), 68.1 ($\underline{C}H_2OCH_2C{\equiv}C$), 55.9 ($CH_2O\underline{C}H_2C{\equiv}C$), 46.8 ($C(O\underline{C}H_3)_2$), 40.3 ($C\underline{C}H_2C(OCH_3)_2$), 28.6 ($CH_2\underline{C}H_2C(OCH_3)_2$), 25.8 ($\underline{C}H_2CH_2C(OCH_3)_2$), 18.0 ($C\underline{C}H_3$).

IR (film) ν_{max}/cm^{-1}: 3252 (C≡C-H), 2940, 2829 (C-H), 2113 (C≡C), 1680 (C=C), 1258, 1131 (C-O).

HRMS (ES+) exact mass calculated for $[M+Na]^+$ $(C_{13}H_{20}NaO_3)^+$ requires *m/z* 247.1305, found *m/z* 247.1304.

tert-butyl((4,4-dimethoxy-2-methylcyclohex-1-en-1-yl)methoxy)dimethylsilane (S8)

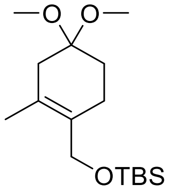

To a stirred solution of (4,4-dimethoxy-2-methylcyclohex-1-en-1-yl)methanol (**S2**) (2.00 g, 15.0 mmol, 1.00 eq) and NEt_3 (2.51 ml, 18.0 mmol, 1.20 eq) in CH_2Cl_2 (30 ml) at 0 °C was added a solution of TBSCl (2.49 g, 16.5 mmol, 1.10 eq) in CH_2Cl_2 (20 ml). The solution was stirred overnight and sat. aq. $NaHCO_3$ (30 ml) was added to the reaction and the phases were separated. The aqueous phase was washed with CH_2Cl_2 (2 x 30 ml). The organic extracts were combined, washed with H_2O (30 ml), brine, dried over Na_2SO_4, and concentrated *in vacuo*. The crude product was purified by silica gel column chromatography (pentane/Et_2O = 98/2 to 97/3) to afford the title compound as a white solid in 51% yield (2.31 g).

^{1}H NMR (500 MHz, Acetone-d_6) δ(ppm): 4.18 (s, 2H, $C\underline{H_2}O$), 3.14 (s, 6H, $C(OC\underline{H_3})_2$), 2.16 (s, 2H, $C\underline{H_2}C(OCH_3)_2$), 2.14 – 2.10 (m, 2H, $C\underline{H_2}CCH_2O$), 1.74 (t, J = 6.5 Hz, 2H, $CH_2C\underline{H_2}C(OCH_3)_2$), 1.64 (s, 3H, $C{=}CC\underline{H_3}$), 0.90 (s, 9H, $C(C\underline{H_3})_3$), 0.07 (s, 6H, $Si(C\underline{H_3})_2$).

^{13}C NMR (126 MHz, Acetone-d_6) δ(ppm): 130.1 ($CH_3C{=}\underline{C}$), 125.6 ($CH_3\underline{C}{=}C$), 100.1 ($\underline{C}(OCH_3)_2$), 63.1 ($\underline{C}H_2O$), 47.7 ($C(O\underline{C}H_3)_2$), 41.2 ($C\underline{C}H_2C(OCH_3)_2$), 29.5 ($CH_2\underline{C}H_2C(OCH_3)_2$), 26.3 ($C(\underline{C}H_3)_3$), 25.9 ($\underline{C}H_2CH_2C(OCH_3)_2$), 18.9 ($\underline{C}(CH_3)_3$), 18.8 ($C{=}C\underline{C}H_3$), -5.1 ($Si(\underline{C}H_3)_2$).

IR (film) ν_{max}/cm^{-1}: 2929, 2856 (C-H), 1130 (C-O), 1054, 834.

HRMS (ES+) exact mass calculated for $[M+Na]^+$ $(C_{16}H_{32}NaO_3Si)^+$ requires *m/z* 323.2013, found *m/z* 323.2012.

MP: 32-33 °C.

tert-butyl ((4,4-dimethoxy-2-methylcyclohex-1-en-1-yl)methyl)(phenyl)carbamate (S9)

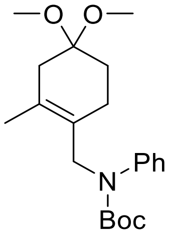

The title compound was prepared according to general procedure **A** from (4,4-dimethoxy-2-methylcyclohex-1-en-1-yl)methanol (**S2**) (400 mg, 2.15 mmol) with *tert*-butyl phenylcarbamate as a nucleophile and *n*-BuLi (2.5 M in hexanes) as the base. The compound was purified by silica gel column chromatography (pentane/Et_2O = 85/15) and obtained as a colourless oil in 50% yield (386 mg).

^{1}H NMR (400 MHz, Benzene-d_6) δ(ppm): 7.18 – 7.04 (m, 4H, ArH), 6.99 – 6.91 (m, 1H, ArH), 4.35 (s, 2H, CH_2N), 3.02 (s, 6H, $C(OCH_3)_2$), 2.26 (m, 2H, $CH_2CH_2C(OCH_3)_2$), 2.03 (s, 2H, CH_2CCH_3), 1.76 (t, J = 6.5 Hz, 2H, $CH_2CH_2C(OCH_3)_2$), 1.40 (s, 9H, $C(CH_3)_3$), 1.20 (s, 3H, CH_2CCH_3).

^{13}C NMR (101 MHz, Benzene-d_6) δ(ppm): 155.0 (C=O), 143.0 (ArC), 128.6 (ArCH), 127.4 (one of C=C), 126.9 (one of C=C), 126.1 (ArCH), 99.7 ($C(OCH_3)_2$), 79.5 ($C(CH_3)_3$), 50.4 (CH_2N), 47.5 ($C(OCH_3)_2$), 40.7 (CH_2CCH_3), 29.3 ($CH_2CH_2C(OCH_3)_2$), 28.4 ($C(CH_3)_3$), 26.5 ($CH_2CH_2C(OCH_3)_2$), 18.6 (CH_2CCH_3) (one ArCH is hidden beneath the solvent peak).

IR (film) ν_{max}/cm^{-1}: 2936, 2828 (C-H), 1694 (C=O), 1597, 1496 (C=C), 1127 (C-O).

HRMS (ES+) exact mass calculated for $[M+Na]^+$ $(C_{21}H_{31}NNaO_4)^+$ requires *m/z* 384.2145, found *m/z* 384.2146.

tert-butyl ((4,4-dimethoxy-2-methylcyclohex-1-en-1-yl)methyl)(prop-2-yn-1-yl)carbamate (S10)

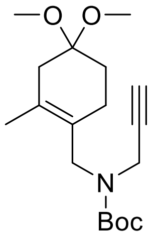

The title compound was prepared according to general procedure **A** from (4,4-dimethoxy-2-methylcyclohex-1-en-1-yl)methanol (**S2**) (400 mg, 2.15 mmol) with *tert*-butyl prop-2-yn-1-ylcarbamate as a nucleophile and KHMDS (0.5 M in toluene) as the preferred base. The compound was purified by silica gel column chromatography (pentane/Et_2O = 90/10 to 80/20) and obtained as a colourless oil in 44% yield (304 mg).

^{1}H NMR (500 MHz, Acetone-d_6) δ(ppm): 4.02 (s, 2H, C$\underline{H_2}$NCH_2C≡CH), 3.90 (bs, 2H, CH_2NC$\underline{H_2}$C≡CH), 3.15 (s, 6H, C(OC$\underline{H_3}$)$_2$), 2.69 (t, *J* = 2.5 Hz, 1H, C≡C$\underline{H}$), 2.21 (bs, 2H, CH_3CC$\underline{H_2}$C(OCH_3)$_2$), 1.99 – 1.92 (m, 2H, C$\underline{H_2}$$CH_2$C($OCH_3$)$_2$), 1.78 – 1.70 (m, 5H, CH_2C$\underline{H_2}$C(OCH_3)$_2$ and C$\underline{H_3}$C=C), 1.46 (s, 9H, C(C$\underline{H_3}$)$_3$).

^{13}C NMR (126 MHz, Acetone-d_6) δ(ppm): 155.6 ($\underline{C}$=O), 128.9 (C=$\underline{C}$$CH_3$) 126.1 ($\underline{C}$=C$CH_3$), 100.1 ($\underline{C}$($OCH_3$)$_2$), 80.9 ($\underline{C}$≡CH), 80.2 ($\underline{C}$($CH_3$)$_3$), 72.5 (C≡$\underline{C}$H), 47.8 (C(O$\underline{C}$$H_3$)$_2$), 46.6 ($\underline{C}$$H_2NCH_2$C≡CH), 41.3 ($\underline{C}$$H_2CCH_3$), 34.7 ($\underline{C}$$H_2$C≡CH), 29.4 ($CH_2$$\underline{C}$$H_2$C($OCH_3$)$_2$), 28.5 (C($\underline{C}$$H_3$)$_3$), 26.1 ($\underline{C}$$H_2$$CH_2$C($OCH_3$)$_2$), 18.9 (C=C$\underline{C}$$H_3$).

IR (film) ν_{max}/cm^{-1}: 3251 (C≡C-H), 2975 (C-H), 1694 (C=O), 1126 (C-O).

HRMS (ES+) exact mass calculated for $[M+Na]^+$ $(C_{18}H_{29}NNaO_4)^+$ requires m/z 346.1989, found m/z 346.1987.

tert-butyl ((4,4-dimethoxy-2-methylcyclohex-1-en-1-yl)methyl)(1,3-dioxoisoindolin-2-yl)carbamate (S11)

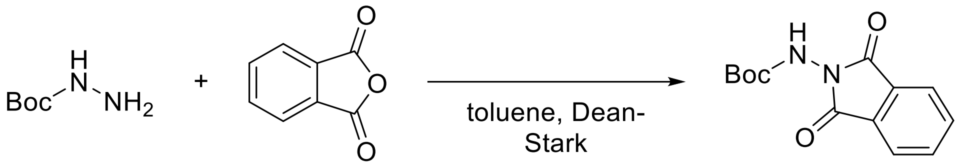

i. According to a modified literature procedure,[4] phthalic anhydride (5.60 g, 37.8 mmol, 1.00 eq) was added to a stirred solution of *tert*-butyl carbazate (5.00 g, 37.8 g, 1.00 eq) in toluene (76 ml). The solution was refluxed under Dean-Stark conditions until no more H_2O was liberated at which point the solution was cooled and concentrated *in vacuo*. The crude product was purified by silica gel column chromatography (pentane/EtOAc = 85/15 to 80/20 to 70/30 to 40/60 to MeOH). The product crashed out on the column after 3.31 g was isolated as a white solid (33%). The remaining product was removed from the column by with MeOH and left to slowly crystalize. The crystals were filtered and washed with MeOH to provide the remainder of the product as colourless crystals in 35% yield (3.49 g) (total yield = 69%, 6.80 g).

^{1}H NMR (400 MHz, $CDCl_3$) δ(ppm): 7.89 (dt, *J* = 7.5, 3.5 Hz, 2H, Ar<u>H</u>), 7.77 (dd, *J* = 5.5, 3.0 Hz, 2H, Ar<u>H</u>), 6.72 (s, 1H, N<u>H</u>), 1.49 (s, 9H, C(C<u>H</u>$_3$)$_3$).

^{13}C NMR (101 MHz, $CDCl_3$) δ(ppm): 165.6 (N(<u>C</u>(=O)$_2$), 153.6 (O<u>C</u>(=O)), 134.8 (Ar<u>C</u>H), 130.1 (Ar<u>C</u>), 124.1 (Ar<u>C</u>H), 83.2 (O<u>C</u>(CH$_3$)$_3$), 28.2 (OC(<u>C</u>H$_3$)$_3$).

Data is consistent with that published in the literature.[4]

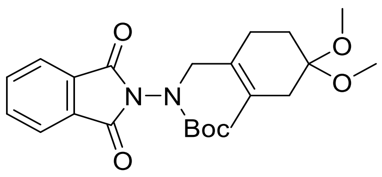

ii. The title compound was prepared according to general procedure **A** from (4,4-dimethoxy-2-methylcyclohex-1-en-1-yl)methanol (**S2**) (400 mg, 2.15 mmol) with tert-butyl (1,3-dioxoisoindolin-2-yl)carbamate as a nucleophile and KHMDS (0.5 M in toluene) as the base. The compound was purified by silica gel column chromatography (pentane/EtOAc = 70/30) and obtained as an unstable white solid (foam) in 61% yield (565 mg) which was quickly taken on to the next step.

tert-butyl ((tert-butyldimethylsilyl)oxy)((4,4-dimethoxy-2-methylcyclohex-1-en-1-yl)methyl)carbamate (S12)

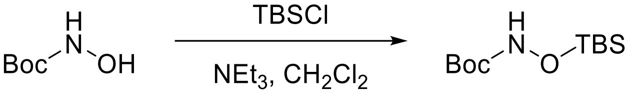

ii. According to a literature procedure,[5] TBSCl (3.40 g, 22.5 mmol, 1.00 eq) was added to a stirred solution of tert-butyl hydroxycarbamate (3.00 g, 22.5 mmol, 1.00 eq) and NEt_3 (3.45 ml, 24.8 mmol, 1.10 eq) in CH_2Cl_2 (30 ml) at 0 °C. The reaction was stirred overnight and allowed to warm to room temperature. H_2O (30 ml) was added and the phases were partitioned. The aqueous layer was extracted with CH_2Cl_2 (2 x 30 ml). The combined organic extracts were washed with brine, dried over $MgSO_4$, filtered and concentrated *in vacuo* to provide tert-butyl ((tert-butyldimethylsilyl)oxy)carbamate as a white solid in 83% yield (4.65 g).

^{1}H NMR (400 MHz, $CDCl_3$) δ(ppm): 6.75 (s, 1H, N<u>H</u>), 1.47 (s, 9H, OC(C<u>H</u>$_3$)$_3$), 0.94 (s, 9H, SiC(C<u>H</u>$_3$)$_3$), 0.15 (s, 6H, Si(CH$_3$)$_2$).

^{13}C NMR (101 MHz, $CDCl_3$) δ(ppm): 157.9 (<u>C</u>=O), 81.5 (O<u>C</u>(CH$_3$)$_3$), 28.2 (one of C(C<u>H</u>$_3$)$_3$), 25.9 (one of C(C<u>H</u>$_3$)$_3$), 18.0 (Si<u>C</u>(CH$_3$)$_3$), -5.8 (Si(<u>C</u>H$_3$)$_2$).

IR (powder) ν_{max}/cm^{-1}: 3279 (N-H), 2931, 2896, 2859 (C-H), 1692 (C=O), 1098 (C-O).

Data is consistent with that published in the literature.[5]

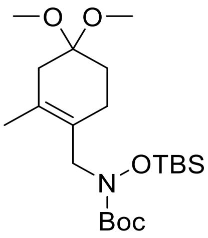

iii. The title compound was prepared according to general procedure **A** from (4,4-dimethoxy-2-methylcyclohex-1-en-1-yl)methanol (**S2**) (400 mg, 2.15 mmol) with tert-butyl ((tert-butyldimethylsilyl)oxy)carbamate as a nucleophile and KHMDS (0.5 M in toluene) as the preferred base. The compound was purified by silica gel column chromatography (pentane/Et_2O = 90/10) and obtained as a colourless oil in 41% yield (365 mg).

^{1}H NMR (400 MHz, Acetone-d_6) δ(ppm): 4.11 (s, 2H, C<u>H</u>$_2$N), 3.14 (s, 6H, C(OC<u>H</u>$_3$)$_2$), 2.17 (s, 2H, C<u>H</u>$_2$CCH$_3$), 2.11 – 2.06 (m, 2H, C<u>H</u>$_2$CH$_2$C(OCH$_3$)$_3$), 1.74 (t, J = 6.5 Hz, 2H, CH$_2$C<u>H</u>$_2$C(OCH$_3$)$_3$), 1.66 (s, 3H, C=CC<u>H</u>$_3$), 1.47 (s, 9H, OC(C<u>H</u>$_3$)$_3$), 0.94 (s, 9H, SiC(C<u>H</u>$_3$)$_3$), 0.15 (s, 6H, Si(C<u>H</u>$_3$)$_2$).

^{13}**C NMR** (126 MHz, Acetone-d_6) δ(ppm): 158.9 ($\underline{C}$=O), 127.7 ($C=\underline{C}CH_3$), 126.7 ($\underline{C}=CCH_3$), 100.0 ($\underline{C}(OCH_3)_2$), 81.2 ($O\underline{C}(CH_3)_3$), 54.1 ($\underline{C}H_2N$), 47.7 ($C(O\underline{C}H_3)_2$), 41.2 ($C\underline{C}H_2C(OCH_3)_2$), 29.5 ($CH_2\underline{C}H_2C(OCH_3)_3$), 28.41 ($OC(\underline{C}H_3)_3$), 26.3 ($\underline{C}H_2CH_2C(OCH_3)_3$ or $SiC(\underline{C}H_3)_3$), 26.3 ($\underline{C}H_2CH_2C(OCH_3)_3$ or $SiC(\underline{C}H_3)_3$), 19.1 ($C=C\underline{C}H_3$), 18.3 ($Si\underline{C}(CH_3)_3$), -4.7 ($Si(\underline{C}H_3)_2$).

IR (film) ν_{max}/cm^{-1}: 2953, 2931, 2896, 2858, 2828 (C-H), 1702 (C=O), 1391 (N-O), 1056 (C-O).

HRMS (ES+) exact mass calculated for $[M+Na]^+$ $(C_{21}H_{41}NNaO_5Si)^+$ requires *m/z* 438.2646, found *m/z* 438.2643.

1-((4,4-dimethoxy-2-methylcyclohex-1-en-1-yl)methyl)-1H-indole (S13)

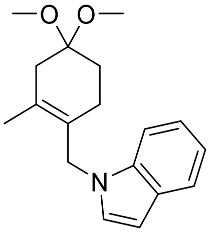

The title compound was prepared according to general procedure **A** from (4,4-dimethoxy-2-methylcyclohex-1-en-1-yl)methanol (**S2**) (400 mg, 2.15 mmol) with indole as a nucleophile and KHMDS (0.5 M in toluene) as the preferred base. The compound was purified by silica gel column chromatography (pentane/Et_2O = 95/5 to 90/10 to 80/20) and obtained as a colourless oil in 21% yield (130 mg).

^{1}H NMR (500 MHz, Acetone-d_6) δ(ppm): 7.55 (dt, *J* = 8.0, 1.0 Hz, 1H, Ar$\underline{H}$), 7.39 (dq, *J* = 8.0, 1.0 Hz, 1H, Ar$\underline{H}$), 7.17 (d, *J* = 3.0 Hz, 1H, Ar$\underline{H}$), 7.13 (ddd, *J* = 8.5, 7.0, 1.0 Hz, 1H, Ar$\underline{H}$), 7.02 (ddd, *J* = 8.0, 7.0, 1.0 Hz, 1H, Ar$\underline{H}$), 6.43 (dd, *J* = 3.0, 1.0 Hz, 1H, Ar$\underline{H}$), 4.80 (s, 2H, $C\underline{H_2}N$), 3.12 (s, 6H, $C(OC\underline{H_3})_2$), 2.28 (s, 2H, $C\underline{H_2}CCH_3$), 1.88 (s, 3H, $CC\underline{H_3}$), 1.83 – 1.77 (m, 2H, $C\underline{H_2}CH_2C(OCH_3)_2$), 1.68 – 1.63 (m, 2H, $CH_2C\underline{H_2}C(OCH_3)_2$).

^{13}C NMR (126 MHz, Acetone-d_6) δ(ppm): 137.5 (Ar$\underline{C}$), 129.8 (Ar$\underline{C}$), 128.7 ($\underline{C}CH_3$), 128.6 (Ar$\underline{C}$H), 126.6 ($\underline{C}=CCH_3$), 121.9 (Ar$\underline{C}$H), 121.3 (Ar$\underline{C}$H), 119.8 (Ar$\underline{C}$H), 110.5 (Ar$\underline{C}$H), 101.5 (Ar$\underline{C}$H), 100.0 ($\underline{C}(OCH_3)_3$), 47.8 ($C(O\underline{C}H_3)_3$), 47.7 ($\underline{C}H_2N$), 41.2 ($\underline{C}H_2CCH_3$), 29.4 ($CH_2\underline{C}H_2C(OCH_3)_3$), 26.2 ($\underline{C}H_2CH_2C(OCH_3)_3$), 19.1 ($C\underline{C}H_3$).

IR (film) ν_{max}/cm^{-1}: 2938, 2828 (C-H), 1612, 1511, 1461 (C=C), 1127 (C-O).

HRMS (ES+) exact mass calculated for $[M+H]^+$ $(C_{18}H_{24}NO_2)^+$ requires m/z 286.1802, found m/z 286.1804.

tert-butyl ((4,4-dimethoxy-2-methylcyclohex-1-en-1-yl)methyl)(furan-2-yl)carbamate (S14)

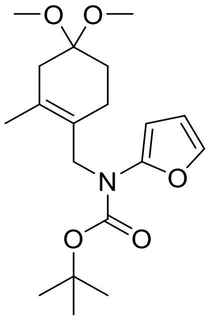

The title compound was prepared according to general procedure **A** from (4,4-dimethoxy-2-methylcyclohex-1-en-1-yl)methanol (**S2**) (300 mg, 1.61 mmol) with tert-butyl furan-2-ylcarbamate as a nucleophile and KHMDS (0.5 M in toluene) as the preferred base. The compound was purified by silica gel column chromatography (pentane/Et_2O = 90/10 to 80/20) and obtained as a colourless oil in 62% yield (348 mg).

^{1}H NMR (400 MHz, Benzene-d_6) δ(ppm): 6.90 (dd, *J* = 2.0, 1.0 Hz, 1H, $NCCHCHC\underline{H}O$), 6.06 (dd, *J* = 3.0, 2.0 Hz, 1H, $NCCHC\underline{H}CHO$), 5.96 (s, 1H, $NCC\underline{H}CHCHO$), 4.30 (s, 2H, $C\underline{H_2}N$), 3.03 (s, 6H, $C(OC\underline{H_3})_2$), 2.26 – 2.14 (m, 2H, $C\underline{H_2}CH_2C(OCH_3)_3$), 2.08 (s, 2H, $CC\underline{H_2}C(OCH_3)_2$), 1.77 (t, *J* = 6.5, 2H, $CH_2C\underline{H_2}C(OCH_3)_3$), 1.39 (s, 9H, $C(C\underline{H_3})_3$), 1.35 (s, 3H, $C\underline{H_3}C{=}C$).

^{13}C NMR (101 MHz, Benzene-d_6) δ(ppm): 154.5 ($N\underline{C}(=O)$), 148.9 ($N\underline{C}CHCHCHO$), 138.5 ($NCCHCH\underline{C}HO$), 126.2 ($CH_3C{=}\underline{C}$ (the other is beneath the solvent peak)), 111.1 ($NCCH\underline{C}HCHO$), 102.5 ($NC\underline{C}HCHCHO$), 100.0 ($\underline{C}(OCH_3)_2$), 80.4 ($O\underline{C}(CH_3)_3$), 49.3 ($\underline{C}H_2N$), 47.5 ($OC(\underline{C}H_3)_3$), 40.9 ($C\underline{C}H_2C(OCH_3)_2$), 29.3 ($CH_2\underline{C}H_2C(OCH_3)_2$), 28.2 ($C(\underline{C}H_3)_3$), 26.2 ($\underline{C}H_2CH_2C(OCH_3)_2$), 18.6 ($\underline{C}H_3C{=}C$).

IR (film) ν_{max}/cm^{-1} 2980 (C-H), 1711 (C=O), 1366, 1150 (C-O ester), 1055 (C-O acetal).

HRMS (ES+) exact mass calculated for $[M+Na]^+$ $(C_{19}H_{29}NNaO_5)^+$ requires *m/z* 374.1938, found *m/z* 374.1934.

benzyl ((4,4-dimethoxy-2-methylcyclohex-1-en-1-yl)methyl)(furan-2-yl)carbamate (S15)

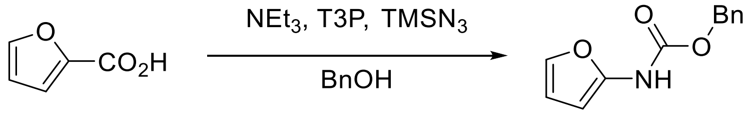

i. To a solution of furan-2-carboxylic acid (2.00 g, 17.8 mmol, 1.00 eq) in toluene (45 mL) was added NEt_3 (3.73 mL, 26.8 mmol, 1.50 eq) followed by T3P (50% in EtOAc, 12.8 mL, 21.4 mmol, 1.20 eq), azidotrimethylsilane (2.84 mL, 21.4 mmol, 1.20 eq) and benzyl alcohol (3.69 mL, 35.7 mmol, 2.00 eq) sequentially. The reaction was refluxed at 120 ˚C for 16 h at which point it was quenched with dilute aq. K_2CO_3 (200 mL) and extracted with EtOAc (3 x 200 mL). The combined organic layers were dried over Na_2SO_4, filtered and concentrated *in vacuo*. The crude product was purified by silica gel column chromatography (pentane/Et_2O = 19/1) to provide benzyl furan-2-ylcarbamate in 64% yield (2.48 g) as a yellow solid.

^{1}H NMR (400 MHz, $CDCl_3$) δ(ppm): 7.33-7.44 (m, 5H, PhCH), 7.09 (s, 1H, OCHCHCHCN), 6.92 (s, 1H, NH), 6.37 (dd, J = 3.0, 2.0 Hz, 1H, OCHCHCHCN), 6.11 (d, J = 15.0 Hz, 1H, OCHCHCHCN), 5.22 (s, 2H, CH_2).

^{13}C NMR (101 MHz, $CDCl_3$) δ(ppm): 144.9 (C=O), 136.7 (OCHCHCHCN), 135.9 (OCHCHCHCN), 128.7 (PhC), 111.6 (OCHCHCHCN), 108.1 (OCHCHCHCN), 67.80 (CH_2).

LRMS (ES+) exact mass calculated for $[M+Na]^+$ $(C_{12}H_{11}NNaO_3)^+$ requires *m/z* 240, found *m/z* 240.

MP: 42-45 ˚C.

Data is consistent with that published in the literature.[3]

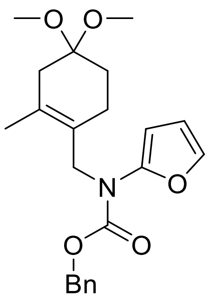

ii. The title compound was prepared according to general procedure **A** from (4,4-dimethoxy-2-methylcyclohex-1-en-1-yl)methanol (**S2**) (219 mg, 1.18 mmol) with benzyl furan-2-ylcarbamate as a nucleophile and KHMDS (0.5 M in toluene) as the base. The compound was purified by silica gel

column chromatography (pentane/Et_2O = 90/10) and obtained as a colourless oil in 37% yield (170 mg).

^{1}H NMR (500 MHz, Acetone-d_6) δ(ppm): 7.39 – 7.26 (m, 6H, ArC$\underline{H}$ and NCCHCHC$\underline{H}$O), 6.38 (dd, J = 3.5, 2.0 Hz, 1H, NCCHC$\underline{H}$CHO), 6.08 (dd, J = 3.5, 1.0 Hz, 1H, NCC$\underline{H}$CHCHO), 5.16 (s, 2H, OC$\underline{H_2}$), 4.25 (s, 2H, C$\underline{H_2}$N), 3.12 (s, 6H, C(OC$\underline{H_3}$)$_2$), 2.09 (s, 2H, CC$\underline{H_2}$C(OCH_3)$_2$), 2.04 – 1.98 (m, 2H, C$\underline{H_2}$$CH_2$C($OCH_3$)$_2$), 1.72 – 1.66 (m, 2H ($CH_2C\underline{H_2}$C($OCH_3$)$_2$)), 1.43 (s, 3H, C$\underline{H_3}$C=C).

^{13}C NMR (126 MHz, Acetone-d_6) δ(ppm): 155.9 (N$\underline{C}$(=O)), 148.1 (N$\underline{C}$CHCHCHO), 140.2 (NCCHCHC$\underline{H}$O), 137.8 (Ar$\underline{C}$), 129.6 ($CH_3$$\underline{C}$=C), 129.2 (Ar$\underline{C}$H), 128.7 (Ar$\underline{C}$H), 128.3 (Ar$\underline{C}$H), 125.8 ($CH_3$C=$\underline{C}$), 111.8 (NCCH$\underline{C}$HCHO), 104.3 (NC$\underline{C}$HCHCHO), 100.0 ($\underline{C}$($OCH_3$)$_2$), 67.9 (O$\underline{C}$$H_2$), 50.2 ($\underline{C}$$H_2$N), 47.7 (C(O$\underline{C}$$H_3$)$_2$), 41.2 (C$\underline{C}$$H_2$C($OCH_3$)$_2$), 29.5 ($CH_2$$\underline{C}$$H_2$C($OCH_3$)$_2$), 26.5 ($\underline{C}$$H_2$$CH_2$C($OCH_3$)$_2$), 18.7 (C$\underline{C}$$H_3$).

IR (film) v_{max}/cm^{-1} 2981 (C-H), 1718 (C=O), 1151 (C-O ester), 1054 (C-O acetal).

HRMS (ES+) exact mass calculated for $[M+Na]^+$ $(C_{22}H_{27}NNaO_5)^+$ requires 408.1781, found 408.1777.

4-((benzyloxy)methyl)-3-methylcyclohex-3-en-1-one (1a)

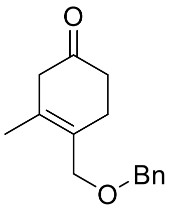

The title compound was prepared according to general procedure **B** from (((4,4-dimethoxy-2-methylcyclohex-1-en-1-yl)methoxy)methyl)benzene (**S3**) (575 mg, 2.08 mmol, 1.00 eq). Upon completion of the reaction as judged by TLC analysis (pentane/Et_2O = 9/1) the reaction mixture was transferred directly to silica gel and purified by silica gel column chromatography (pentane/Et_2O = 95/5) to afford the title compound as a yellow oil in 95% yield (457 mg).

^{1}H NMR (500 MHz, $CDCl_3$) δ(ppm): 7.44 – 7.27 (m, 5H, Ar$\underline{H}$), 4.50 (s, 2H, $C\underline{H_2}Ph$), 4.08 (s, 2H, $C\underline{H_2}OCH_2Ph$), 2.84 (s, 2H, $CC\underline{H_2}C(=O)$), 2.60 – 2.52 (m, 2H, $CH_2C\underline{H_2}C(=O)$), 2.51 – 2.44 (m, 2H, $C\underline{H_2}CH_2C(=O)$), 1.72 (s, 3H, $C\underline{H_3}$).

^{13}C NMR (126 MHz, $CDCl_3$) δ(ppm): 210.6 ($\underline{C}$=O), 138.4 (Ar$\underline{C}$), 129.6 ($CH_3\underline{C}$), 128.6 (Ar$\underline{C}$H), 128.4 ($CH_3C=\underline{C}$), 127.9 (Ar$\underline{C}$H), 127.9 (Ar$\underline{C}$H), 72.4 ($\underline{C}H_2Ph$), 69.1 ($\underline{C}H_2OCH_2Ph$), 45.7 ($C\underline{C}H_2C(=O)$), 39.0 ($\underline{C}H_2CH_2C(=O)$), 27.8 ($CH_2\underline{C}H_2C(=O)$), 18.7 ($\underline{C}H_3$).

IR (film) ν_{max}/cm^{-1}: 1717 (C=O), 1262 (C-H), 1068.

HRMS (ES+) exact mass calculated for $[M+H]^+$ $(C_{15}H_{18}NaO_2)^+$ requires *m/z* 253.1199, found *m/z* 253.1201.

4-(methoxymethyl)-3-methylcyclohex-3-en-1-one (1b)

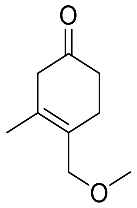

The title compound was prepared according to general procedure **B** from 4,4-dimethoxy-1-(methoxymethyl)-2-methylcyclohex-1-ene (**S4**) (135mg, 0.670 mmol, 1.00 eq). Upon reaction completion as judged by TLC analysis the reaction mixture was diluted with H_2O (20 ml) and extracted with Et_2O (3 x 10 ml). The combined organic layers were washed with H_2O (10 ml), brine, dried over Na_2SO_4 and concentrated *in vacuo*. The crude reaction mixture was purified by silica gel column chromatography (pentane/Et_2O = 1/1) to obtain the title compound as a colourless oil in 71% yield (73.0 mg).

^{1}H NMR (500 MHz, Benzene-d_6) δ(ppm): 3.65 (s, 2H, C$\underline{H_2}$O), 3.01 (s, 3H, C$\underline{H_3}$O), 2.46 (s, 2H, CC$\underline{H_2}$C(=O)), 2.23 – 2.17 (m, 2H, C$\underline{H_2}$CH$_2$C(=O)), 2.17 – 2.11 (m, 2H, CH$_2$C$\underline{H_2}$C(=O)), 1.29 (s, 3H, C$\underline{H_3}$C).

^{13}C NMR (126 MHz, Benzene-d_6) δ(ppm): 207.7 ($\underline{C}$=O), 128.9 (one of $\underline{C}$=C), 128.6 (one of C=$\underline{C}$), 71.2 ($\underline{C}$H$_2$O), 57.4 ($\underline{C}$H$_3$O), 45.5 (C$\underline{C}$H$_2$C(=O)), 38.8 (CH$_2\underline{C}$H$_2$C(=O)), 27.6 ($\underline{C}$H$_2$CH$_2$C(=O)), 18.2 (C$\underline{C}$H$_3$).

IR (film) ν_{max}/cm^{-1}: 2980, 2891, 2818 (C-H), 1717 (C=O), 1083 (C-O).

HRMS (ES+) exact mass calculated for $[M+H]^+$ $(C_9H_{15}O_2)^+$ requires *m/z* 155.1066, found *m/z* 155.1067.

4-((allyloxy)methyl)-3-methylcyclohex-3-en-1-one (1c)

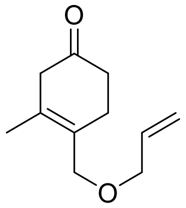

The title compound was prepared according to general procedure **B** from 1-((allyloxy)methyl)-4,4-dimethoxy-2-methylcyclohex-1-ene (**S5**) (37 mg, 0.163 mmol, 1.00 eq). Upon completion of the reaction as judged by TLC analysis the reaction mixture was transferred directly to silica gel and purified by silica gel column chromatography (pentane/Et_2O = 9/1) to afford the title compound as a colourless oil in 99% yield (25.0 mg).

^{1}H NMR (400 MHz, Acetone-d_6) δ(ppm): 5.92 (ddt, *J* = 17.5, 10.5, 5.5 Hz, 1H, C<u>H</u>=CH_2), 5.26 (dq, *J* = 17.5, 2.0 Hz, 1H, one of CH=C$\underline{H}_A H_B$), 5.12 (ddt, *J* = 10.5, 2.0, 1.5 Hz, 1H, one of CH=C$H_A \underline{H}_B$), 4.05 (s, 2H, CH_3C=C$\underline{H_2}$O), 3.94 (dt, *J* = 5.5, 1.5 Hz, 2H, C$\underline{H_2}$CH=CH_2), 2.80 (s, 2H, CC$\underline{H_2}$C(=O)), 2.52 – 2.45 (m, 2H, CH_2C$\underline{H_2}$C(=O)), 2.42 – 2.36 (m, 2H, C$\underline{H_2}$$CH_2$C(=O)), 1.73 (s, 3H, C$\underline{H_3}$).

^{13}C NMR (101 MHz, Acetone-d_6) δ(ppm): 209.5 (<u>C</u>=O), 136.5 (<u>C</u>H=CH_2), 129.8 (CH_3<u>C</u>=C), 129.2 (CH_3C=<u>C</u>), 116.4 (CH=<u>C</u>H_2), 71.1 (<u>C</u>H_2CH=CH_2), 69.3 (CH_3C=C<u>C</u>H_2O), 46.0 (C<u>C</u>H_2C(=O)), 39.2 (CH_2<u>C</u>H_2C(=O)), 28.2 (<u>C</u>$H_2$$CH_2$C(=O)),), 18.5 (<u>C</u>$H_3$).

IR (film) ν_{max}/cm^{-1}: 1717 (C=O), 1671 (C=C), 1072 (C-O).

HRMS (ES+) exact mass calculated for $[M+H]^+$ $(C_{11}H_{17}O_2)^+$ requires *m/z* 181.1223, found *m/z* 181.1224.

3-methyl-4-(((3-methylbut-2-en-1-yl)oxy)methyl)cyclohex-3-en-1-one (1d)

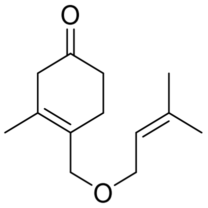

The title compound was prepared according to general procedure **B** from 4,4 dimethoxy-2-methyl-1-(((3-methylbut-2-en-1-yl)oxy)methyl)cyclohex-1-ene (**S6**) (443 mg, 1.74 mmol). The crude product was purified by silica gel column chromatography (pentane/ Et_2O = 85/15) to afford the title compound as a colourless oil in 91% yield (331 mg).

^{1}H NMR (500 MHz, Acetone-d_6) δ(ppm): 5.32 (tdt, J = 6.5, 3.0, 1.5 Hz, 1H, $C\underline{H}CH_2O$), 4.01 (s, 2H, $C\underline{H_2}OCH_2CH$), 3.93 (d, J = 6.5 Hz, 2H, $OC\underline{H_2}CH$), 2.79 (s, 2H, $CC\underline{H_2}C(=O)$) 2.52 – 2.44 (m, 2H, $C\underline{H_2}CH_2C(=O)$), 2.41 – 2.36 (m, 2H, $CH_2C\underline{H_2}C(=O)$), 1.73 (s, 3H, $C\underline{H_3}CCH_2C(=O)$), 1.72 (d, J = 1.0 Hz, 3H, one of $C=C(C\underline{H_3})_A(CH_3)_B$), 1.65 (s, 3H, one of $C=C(CH_3)_A(C\underline{H_3})_B$).

^{13}C NMR (126 MHz, Acetone-d_6) δ(ppm): 209.3 ($\underline{C}=O$), 136.3 ($\underline{C}(CH_3)_2$), 129.5 (one of $(CH_3)(CH_2)\underline{C}=C(CH_2)(CH_2)$), 129.5 ($(CH_3)(CH_2)C=\underline{C}(CH_2)(CH_2)$), 122.9 ($CH_2\underline{C}H=C$), 69.0 ($\underline{C}H_2OCH_2CH$), 66.8 ($CH_2O\underline{C}H_2CH$), 46.0 ($C\underline{C}H_2C(=O)$), 39.2 ($CH_2C\underline{H_2}C(=O)$), 28.3 ($\underline{C}H_2CH_2C(=O)$), 25.8 (one of $C(\underline{C}H_3)_A(CH_3)_B$), 18.5 ($\underline{C}H_3CCH_2C(=O)$), 18.0 (one of $C(CH_3)_A(\underline{C}H_3)_B$).

IR (film) ν_{max}/cm^{-1}: 2970, 2914, 2857 (C-H), 1718 (C=O), 1676 (C=C), 1061 (C-O).

HRMS (ES+) exact mass calculated for $[M+Na]^+$ $(C_{13}H_{20}NaO_2)^+$ requires m/z 231.1356, found m/z 231.1358.

3-methyl-4-((prop-2-yn-1-yloxy)methyl)cyclohex-3-en-1-one (1e)

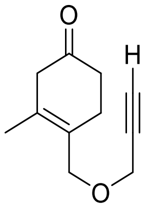

The title compound was prepared according to general procedure **B** from 4,4-dimethoxy-2-methyl-1-((prop-2-yn-1-yloxy)methyl)cyclohex-1-ene (**S7**) (168 mg, 0.75 mmol, 1.00 eq). Upon completion of the reaction as judged by TLC analysis the reaction mixture was transferred directly to silica gel and purified by silica gel column chromatography (pentane/Et_2O = 7/3) to afford the title compound as a colourless oil in 77% yield (103 mg).

^{1}H NMR (500 MHz, Benzene-d_6) δ(ppm): 3.82 (s, 2H, $C\underline{H_2}CCH_2$), 3.77 (d, J = 2.5 Hz, 2H, $C\underline{H_2}C{\equiv}CH$), 2.43 (s, 2H, $C\underline{H_2}CCH_3$), 2.18 – 2.06 (m, 4H, $C\underline{H_2}C\underline{H_2}C(=O)$), 2.02 (t, J = 2.5 Hz, 1H, $C{\equiv}C\underline{H}$), 1.32 (s, 3H, $C\underline{H_3}$).

^{13}C NMR (126 MHz, Benzene-d_6) δ(ppm): 207.4 ($\underline{C}=O$), 130.1 ($\underline{C}CH_3$), 127.7 ($\underline{C}=CCH_3$), 80.5 ($\underline{C}{\equiv}CH$), 74.4 ($C{\equiv}\underline{C}H$), 68.2 ($\underline{C}H_2C=CCH_3$), 56.8 ($\underline{C}H_2C{\equiv}CH$), 45.5 ($\underline{C}H_2CCH_3$), 38.7 ($CH_2\underline{C}H_2C(=O)$), 27.6 ($\underline{C}H_2CH_2C(=O)$), 18.3 ($\underline{C}H_3$).

IR (film) ν_{max}/cm^{-1}: 3263 (C≡C-H), 2911, 2852 (C-H), 2113 (C≡C), 1715 (C=O).

HRMS (APCI+) exact mass calculated for $[M+H]^+$ $(C_{11}H_{15}O_2)^+$ requires *m/z* 179.1067, found *m/z* 179.1070.

4-(((tert-butyldimethylsilyl)oxy)methyl)-3-methylcyclohex-3-en-1-one (1f)

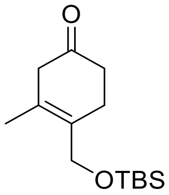

The title compound was prepared according to general procedure **B** from tert-butyl((4,4-dimethoxy-2-methylcyclohex-1-en-1-yl)methoxy)dimethylsilane (**S8**) (2.30 g, 7.65 mmol). The crude product was purified by silica gel column chromatography (pentane/Et_2O = 9/1) to afford the title compound as a colourless oil in 84% yield (1.63 g).

^{1}H NMR (500 MHz, Acetone-d_6) δ(ppm): 4.28 (s, 2H, $C\underline{H_2}O$), 2.78 (s, 2H, $CH_3CC\underline{H_2}C(=O)$), 2.55 – 2.48 (m, 2H, $C\underline{H_2}CH_2C(=O)$), 2.42 – 2.36 (m, 2H, $CH_2C\underline{H_2}C(=O)$), 1.72 (s, 3H, $C=CC\underline{H_3}$), 0.91 (s, 9H, $C(C\underline{H_3})_3$), 0.09 (s, 6H, $Si(C\underline{H_3})_2$).

^{13}C NMR (126 MHz, Acetone-d_6) δ(ppm): 209.4 ($\underline{C}=O$), 131.4 ($\underline{C}=CCH_3$), 127.1 ($C=\underline{C}CH_3$), 62.7 ($\underline{C}H_2O$), 46.0 ($CH_3C\underline{C}H_2C(=O)$), 39.2 ($CH_2\underline{C}H_2C(=O)$), 27.6 ($\underline{C}H_2CH_2C(=O)$), 26.3 ($C(\underline{C}H_3)_3$), 18.8 ($\underline{C}(CH_3)_3$), 18.4 ($C=C\underline{C}H_3$), -5.1 ($Si(\underline{C}H_3)_2$).

IR (film) ν_{max}/cm^{-1}: 2956, 2929, 2887, 2856 (C-H), 1721 (C=O), 1063 (C-O).

HRMS (APCI+) exact mass calculated for $[M+H]^+$ $(C_{14}H_{27}O_2Si)^+$ requires *m/z* 255.1775, found *m/z* 255.1778.

4-(hydroxymethyl)-3-methylcyclohex-3-en-1-one (1g)

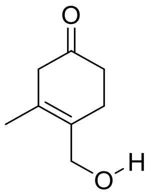

The title compound was prepared according to general procedure **B** from (4,4-dimethoxy-2-methylcyclohex-1-en-1-yl)methanol (**S2**) (100 mg, 0.536 mmol, 1.00 eq) using catalyst **C1** (11.0 mg, 0.0286 mmol, 0.05 eq). Upon completion of the reaction as judged by TLC analysis (Et_2O) the reaction mixture was transferred directly to silica gel and purified by silica gel column chromatography (Et_2O) to afford the title compound as a yellow oil in 83% yield (63 mg).

^{1}H NMR (400 MHz, $CDCl_3$) δ(ppm): 4.22 (s, 2H, $C\underline{H_2}OH$), 2.83 (s, 2H, $CC\underline{H_2}C(=O)$), 2.57 (m, 2H, $CH_2C\underline{H_2}C(=O)$), 2.51 – 2.46 (m, 2H, $C\underline{H_2}CH_2C(=O)$), 1.75 (s, 3H, $C\underline{H_3}$), 1.46 (s, 1H, $O\underline{H}$).

^{13}C NMR (101 MHz, $CDCl_3$) δ(ppm): 210.6 ($\underline{C}$=O), 130.5 ($CH_3\underline{C}$=C), 128.5 (CH_3C=$\underline{C}$), 62.0 ($\underline{C}H_2OH$), 45.7 ($C\underline{C}H_2C(=O)$), 39.0 ($\underline{C}H_2CH_2C(=O)$), 27.5 ($CH_2\underline{C}H_2C(=O)$), 18.5 ($\underline{C}H_3$).

IR (film) ν_{max}/cm^{-1}: 3391 (O-H), 2919, 2857 (C-H), 1709 (C=O), 995 (C-O).

HRMS (ES+) exact mass calculated for $[M+H]^+$ $(C_8H_{13}O_2)^+$ requires *m/z* 141.0910, found *m/z* 141.0911.

tert-butyl ((2-methyl-4-oxocyclohex-1-en-1-yl)methyl)(phenyl)carbamate (1i)

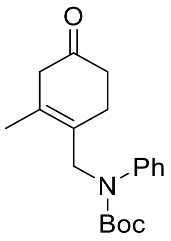

The title compound was prepared according to general procedure **C** from tert-butyl ((4,4-dimethoxy-2-methylcyclohex-1-en-1-yl)methyl)(phenyl)carbamate (**S9**) (330 mg, 0.913 mmol). The crude product was purified by silica gel column chromatography (pentane/Et_2O = 7/3) to afford the title compound as a white solid in 89% yield (256 mg).

^{1}H NMR (600 MHz, Acetone-d_6) δ(ppm): 7.37 – 7.28 (m, 2H, ArH), 7.28 – 7.22 (m, 2H, ArH), 7.22 – 7.10 (m, 1H, ArH), 4.45 (s, 2H, $C\underline{H_2}N$), 2.65 (s, 2H, $CC\underline{H_2}C(=O)$), 2.46 – 2.39 (m, 2H, $C\underline{H_2}CH_2C(=O)$), 2.29 (t, *J* = 7.0 Hz, 2H, $CH_2C\underline{H_2}C(=O)$), 1.48 (s, 3H, $C=CC\underline{H_3}$), 1.40 (s, 9H, $C(C\underline{H_3})_3$).

^{13}C NMR (151 MHz, Acetone-d_6) δ(ppm): 209.1 ($CH_2\underline{C}(=O)$), 155.4 ($N\underline{C}(=O)$), 143.2 (ArC), 129.6 ($C=\underline{C}CH_3$), 129.3 (ArCH), 128.5 ($\underline{C}=CCH_3$), 128.3 (ArCH), 126.8 (ArCH), 80.2 ($O\underline{C}(CH_3)_3$), 50.3 ($\underline{C}H_2N$), 46.0 ($C\underline{C}H_2C(=O)$), 39.1 ($CH_2\underline{C}H_2C(=O)$), 28.5 ($C(\underline{C}H_3)_3$), 28.0 ($\underline{C}H_2CH_2C(=O)$), 18.4 ($C=C\underline{C}H_3$).

IR (powder) ν_{max}/cm^{-1}: 2979, 3048 (C-H), 1717 (ketone C=O), 1687 (boc C=O), 754, 702 (C-H).

HRMS (ES+) exact mass calculated for $[M+H]^+$ $(C_{19}H_{26}NO_3)^+$ requires *m/z* 316.1907, found *m/z* 316.1908.

MP: 52-54 °C.

tert-butyl ((2-methyl-4-oxocyclohex-1-en-1-yl)methyl)(prop-2-yn-1-yl)carbamate (1j)

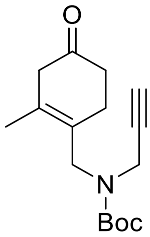

The title compound was prepared according to general procedure **C** from tert-butyl ((4,4-dimethoxy-2-methylcyclohex-1-en-1-yl)methyl)(prop-2-yn-1-yl)carbamate (**S10**) (255 mg, 0.788 mmol). The crude product was purified by silica gel column chromatography (pentane/Et_2O = 8/2) to afford the title compound as a white solid in 85% yield (185 mg).

^{1}H NMR (600 MHz, Acetone-*d*$_6$) δ(ppm): 4.12 (s, 2H, C=CC$\underline{H_2}$N), 3.97 (bs, 2H, C$\underline{H_2}$C≡CH), 2.81 (s, 2H, CC$\underline{H_2}$C(=O) (beneath water peak)), 2.71 (t, *J* = 2.5 Hz, 1H, C≡C$\underline{H}$), 2.42 – 2.35 (m, 4H, C$\underline{H_2}$CH$_2$C(=O) and CH$_2$C$\underline{H_2}$C(=O)), 1.81 (m, 3H, C=CC$\underline{H_3}$), 1.47 (s, 9H, C(C$\underline{H_3}$)$_3$).

^{13}C NMR (151 MHz, Acetone-*d*$_6$) δ(ppm): 209.1 (CH$_2$$\underline{C}$(=O)), 155.7 (N$\underline{C}$(=O)), 130.4 (C=$\underline{C}CH_3$), 127.7 ($\underline{C}$=CCH$_3$), 80.9 (O$\underline{C}$(CH$_3$)$_3$), 80.4 ($\underline{C}$≡CH), 72.6 (C≡$\underline{C}$H), 46.5 (C=C$\underline{C}H_2$N), 46.1 (C$\underline{C}H_2$C(=O)), 39.1 (CH$_2$$\underline{C}H_2$C(=O)), 35.3 ($\underline{C}H_2$C≡CH), 28.5 (C($\underline{C}H_3$)$_3$), 27.6 ($\underline{C}H_2CH_2$C(=O)), 18.6 (C=C$\underline{C}H_3$).

IR (film) ν_{max}/cm^{-1}: 3235 (C≡C-H), 2929, 2855 (C-H), 2362 (C≡C), 1689 (C=O).

HRMS (ES+) exact mass calculated for [M+Na]$^+$ ($C_{16}H_{23}NNaO_3$)$^+$ requires *m/z* 300.1570, found *m/z* 300.1571.

MP: 46-48 °C.

tert-butyl (1,3-dioxoisoindolin-2-yl)((2-methyl-4-oxocyclohex-1-en-1-yl)methyl)carbamate (1k)

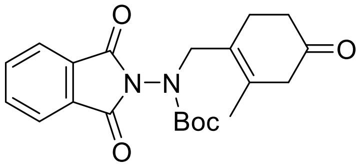

The title compound was prepared according to general procedure **C** from tert-butyl ((4,4-dimethoxy-2-methylcyclohex-1-en-1-yl)methyl)(1,3-dioxoisoindolin-2-yl)carbamate (**S11**) (255 mg, 0.788 mmol). The crude product was purified by silica gel column chromatography (pentane/EtOAc = 6/4) to afford the title compound as a white solid (foam) in 44% yield over two steps (363 mg).

^{1}H NMR (400 MHz, Methanol-*d*$_4$) δ(ppm): 8.06 – 7.84 (m, 4H, ArH), 4.40 (s, 2H, CH_2N), 2.71 (s, 2H, $CCH_2C(=O)$), 2.68 – 2.58 (m, 2H, $CH_2CH_2C(=O)$), 2.52 – 2.43 (m, 2H, $CH_2CH_2C(=O)$), 1.55 (s, 3H, $C=CCH_3$), 1.53 (s, 3H, $C(CH_3)_3$ (minor rotamer)), 1.33 (s, 6H, $C(CH_3)_3$ (major rotamer)).

^{13}C NMR (101 MHz, Methanol-*d*$_4$) δ(ppm): 212.8 ($CH_2C(=O)$), 166.7 (ArC(=O)), 155.3 (OC(=O)), 136.5 (ArCH, major rotamer), 136.4 (ArCH, minor rotamer), 132.7 (ArC), 130.9 ($C=CCH_3$), 126.9 ($C=CCH_3$), 124.9 (ArCH), 84.6 ($OC(CH_3)_3$, minor rotamer), 83.6 ($OC(CH_3)_3$, major rotamer), 51.0 (CH_2N, minor rotamer), 49.1 (CH_2N, major rotamer), 46.1 ($CCH_2C(=O)$), 39.5 ($CH_2CH_2C(=O)$), 28.7 ($CH_2CH_2C(=O)$), 28.4 ($C(CH_3)_3$ minor rotamer), 28.1 ($C(CH_3)_3$ major rotamer), 18.3 ($C=CCH_3$).

HRMS (ES+) exact mass calculated for $[M+Na]^+$ ($C_{21}H_{24}N_2NaO_5$) requires *m/z* 407.1577, found *m/z* 407.1573.

IR (powder) ν_{max}/cm^{-1}: 2981, 2889 (C-H), 1732, 1704 (C=O), 1425 (C-O), 1149 (C-N).

MP: 106-109 °C.

tert-butyl ((tert-butyldimethylsilyl)oxy)((2-methyl-4-oxocyclohex-1-en-1-yl)methyl)carbamate (1l)

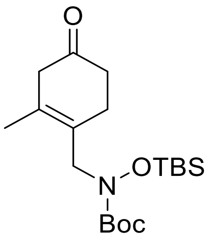

The title compound was prepared according to general procedure **B** from tert-butyl ((tert-butyldimethylsilyl)oxy)((4,4-dimethoxy-2-methylcyclohex-1-en-1-yl)methyl)carbamate (**S12**) (331 mg, 0.796 mmol). The crude product was purified by silica gel column chromatography (pentane/Et_2O = 9/1) to afford the title compound as a colourless oil in 60% yield (176 mg).

^{1}H NMR (400 MHz, Acetone-d_6) δ(ppm): 4.23 (s, 2H, C$\underline{H_2}$N), 2.79 (s, 2H, C$\underline{H_2}$C(=O)), 2.49 – 2.43 (m, 2H, C$\underline{H_2}$$CH_2$C(=O)), 2.43 – 2.35 (m, 2H, CH_2C$\underline{H_2}$C(=O)), 1.76 (s, 3H, C=CC$\underline{H_3}$), 1.48 (s, 9H, OC(C$\underline{H_3}$)$_3$), 0.93 (s, 9H, SiC(C$\underline{H_3}$)$_3$), 0.17 (s, 6H, Si(C$\underline{H_3}$)$_2$).

^{13}C NMR (126 MHz, Acetone-d_6) δ(ppm): 209.1 ($CH_2$$\underline{C}$(=O)), 158.9 (N$\underline{C}$(=O)), 129.6 (C=$\underline{C}$$CH_3$), 128.1 ($\underline{C}$=C$CH_3$), 81.5 (O$\underline{C}$($CH_3$)$_3$, 53.8 ($\underline{C}$$H_2$N), 46.1 (C$\underline{C}$$H_2$C(=O)), 39.0 ($CH_2$$\underline{C}$$H_2$C(=O)), 28.4 (OC($\underline{C}$$H_3$)$_3$), 28.3 ($\underline{C}$$H_2$$CH_2$C(=O)), 26.2 (SiC($\underline{C}$$H_3$)$_3$), 18.9 (C=C$\underline{C}$$H_3$), 18.3 (Si$\underline{C}$($CH_3$)$_3$), -4.7 (Si($\underline{C}$$H_3$)$_2$).

IR (film) ν_{max}/cm^{-1}: 2957. 2931, 2852 (C-H), 1721 (ketone C=O), 1700 (boc C=O).

HRMS (ES+) exact mass calculated for $[M+Na]^+$ $(C_{19}H_{35}NNaO_4Si)^+$ requires *m/z* 392.2228, found *m/z* 392.2226.

4-((1H-indol-1-yl)methyl)-3-methylcyclohex-3-en-1-one (1m)

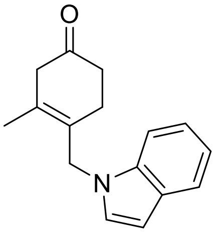

The title compound was prepared according to general procedure **C** from 1-((4,4-dimethoxy-2-methylcyclohex-1-en-1-yl)methyl)-1H-indole (**S13**) (113 mg, 0.397 mmol). The crude product was purified by silica gel column chromatography (pentane/Et_2O = 8/2) to afford the title compound as a white solid in 95% yield (85.0 mg).

^{1}H NMR (500 MHz, Acetone-d_6) δ(ppm): 7.56 (dt, *J* = 8.0, 1.0 Hz, 1H, Ar<u>H</u>), 7.40 (dq, *J* = 8.5, 1.0 Hz, 1H, Ar<u>H</u>), 7.26 (d, *J* = 3.0 Hz, 1H, Ar<u>H</u>), 7.13 (ddd, *J* = 8.0, 7.0, 1.0 Hz, 1H, Ar<u>H</u>), 7.02 (ddd, *J* = 8.0, 7.0, 1.0 Hz, 1H, Ar<u>H</u>), 6.46 (dd, *J* = 3.0, 1.0 Hz, 1H, Ar<u>H</u>), 4.94 (s, 2H, C<u>H</u>$_2$N), 2.89 (s, 2H, CC<u>H</u>$_2$C(=O)), 2.27 (t, *J* = 7.0 Hz, 2H, C<u>H</u>$_2$CH$_2$C(=O)), 2.19 – 2.11 (m, 2H, CH$_2$C<u>H</u>$_2$C(=O)), 2.01 – 1.97 (m, 3H, CH$_3$).

^{13}C NMR (126 MHz, Acetone-d_6) δ(ppm): 208.8 (<u>C</u>=O), 137.5 (Ar<u>C</u>), 129.9 (Ar<u>C</u>), 129.8 (CH$_3$<u>C</u>=C), 129.0 (Ar<u>C</u>H), 128.0 (CH$_3$C=<u>C</u>), 122.1 (Ar<u>C</u>H), 121.5 (Ar<u>C</u>H), 120.0 (Ar<u>C</u>H), 110.4 (Ar<u>C</u>H), 101.8 (Ar<u>C</u>H), 47.6 (<u>C</u>H$_2$N), 46.0 (C<u>C</u>H$_2$C(=O)), 38.9 (CH$_2$<u>C</u>H$_2$C(=O)), 27.5 (<u>C</u>H$_2$CH$_2$C(=O)), 18.8 (<u>C</u>H$_3$).

IR (powder) ν_{max}/cm^{-1}: 2980, 2913 (C-H), 1715 (C=O).

HRMS (ES+) exact mass calculated for $[M+H]^+$ $(C_{16}H_{18}NO)^+$ requires *m/z* 240.1383, found *m/z* 240.1384.

MP: 72-74 °C.

tert-butyl furan-2-yl((2-methyl-4-oxocyclohex-1-en-1-yl)methyl)carbamate (1n)

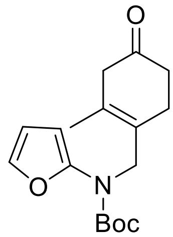

The title compound was prepared according to general procedure **C** from tert-butyl ((4,4-dimethoxy-2-methylcyclohex-1-en-1-yl)methyl)(furan-2-yl)carbamate (**S14**) (307 mg, 0.870 mmol). The crude product was purified by silica gel column chromatography (pentane/Et_2O = 8/2 to 7/3) to afford the title compound as a colourless oil in 86% yield (229 mg).

^{1}H NMR (500 MHz, Benzene-d_6) δ(ppm): 6.83 (dd, J = 2.0, 1.0 Hz, 1H, NCCHCHC<u>H</u>O), 6.00 (dd, J = 3.0, 2.0 Hz, 1H, NCCHC<u>H</u>CHO), 5.76 (bs, 1H, NCC<u>H</u>CHCHO), 4.20 (s, 2H, C<u>H$_2$</u>N), 2.36 (s, 2H, CC<u>H$_2$</u>C(=O)), 2.25 – 2.18 (m, 2H, C<u>H$_2$</u>CH$_2$C(=O)), 2.12 (t, J = 7.0 Hz, 2H, CH$_2$C<u>H$_2$</u>C(=O)), 1.36 (s, 9H, C(CH$_3$)$_3$), 1.19 (s, 3H, C=CC<u>H$_3$</u>).

^{13}C NMR (126 MHz, Benzene-d_6) δ(ppm): 207.5 (CH$_2$<u>C</u>(=O)), 154.4 (N<u>C</u>(=O)), 148.8 (N<u>C</u>CHCHCHO), 138.6 (NCCHCH<u>C</u>HO), 129.5 (CH$_3$<u>C</u>=C), 127.2 (CH$_3$C=<u>C</u>), 111.1 (NCCH<u>C</u>HCHO), 102.4 (NC<u>C</u>HCHCHO), 80.8 (<u>C</u>(CH$_3$)$_3$), 49.1 (<u>C</u>H$_2$N), 45.5 (C<u>C</u>H$_2$C(=O)), 38.7 (CH$_2$<u>C</u>H$_2$C(=O)), 28.2 (C(<u>C</u>H$_3$)$_3$), 27.4 (<u>C</u>H$_2$CH$_2$C(=O)), 18.1 (<u>C</u>H$_3$C=C).

HRMS (ES+) exact mass calculated for $[M+H]^+$ $(C_{17}H_{24}NO_4)^+$ requires *m/z* 306.1700, found *m/z* 306.1701.

benzyl furan-2-yl((2-methyl-4-oxocyclohex-1-en-1-yl)methyl)carbamate (1o)

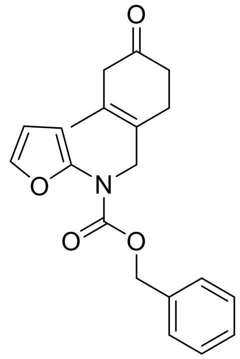

The title compound was prepared according to general procedure **C** from tert-butyl benzyl ((4,4-dimethoxy-2-methylcyclohex-1-en-1-yl)methyl)(furan-2-yl)carbamate (**S15**) (157 mg, 0.408 mmol). The crude product was purified by silica gel column chromatography (pentane/Et_2O = 8/2 to 7/3) to afford the title compound as a colourless oil in 82% yield (114 mg).

^{1}H NMR (500 MHz, Acetone-d_6) δ(ppm): 7.42 – 7.24 (m, 6H, ArCH and NCCHCHCHO), 6.40 (dd, *J* = 3.5, 2.0 Hz, 1H, NCCHCHCHO), 6.17 (dd, *J* = 3.5, 1.0 Hz, 1H, NCCHCHCHO), 5.17 (s, 2H, CH_2O), 4.38 (s, 2H, CH_2N), 2.70 (s, 2H, CCH_2C(=O)), 2.45 – 2.36 (m, 2H, CH_2CH_2C(=O)), 2.32 (m, 2H, CH_2CH_2C(=O)), 1.55 (s, 3H, CH_3).

^{13}C NMR (126 MHz, Acetone-d_6) δ(ppm): 209.0 (CH_2C(=O)), 155.9 (NC(=O)), 148.3 (NCCHCHCHO), 140.3 (NCCHCHCHO), 137.7 (ArC), 131.0 (CH_3C), 129.3 (ArCH), 128.8 (ArCH), 128.3 (ArCH), 127.3 (CH_3C=C), 111.9 (NCCHCHCHO), 104.2 (NCCHCHCHO), 68.1 (CH_2O), 50.2 (CH_2N), 46.0 (CCH_2C(=O)), 39.1 (CH_2CH_2C(=O)), 28.0 (CH_2CH_2C(=O)), 18.4 (CH_3).

IR (film) ν_{max}/cm^{-1}: 2981 (C-H), 1717 (C=O ester), 1612 (C=O ketone), 1395, 1277, 1154 (C-O).

HRMS (ES+) exact mass calculated for $[M+H]^+$ $(C_{20}H_{22}NO_4)^+$ requires *m/z* 340.1543, found *m/z* 340.1544.

1.2.2 Substrates prepared via cobalt catalysed Diels-Alder reaction

4-((benzyloxy)methyl)cyclohex-3-en-1-one (1h)

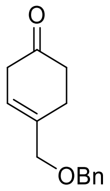

ii. According to a literature procedure,[6] ((prop-2-yn-1-yloxy)methyl)benzene (100 mg, 0.684 mmol, 1.00 eq) and (buta-1,3-dien-2-yloxy)trimethylsilane (181 μl, 1.03 mmol, 1.50 eq) were added sequentially to a solution of $CoBr_2$(DPPE) (42.0 mg, 0.0680 mmol, 0.100 eq), ZnI (44.0 mg, 0.137 mmol, 0.200 eq) and Zn (8.70 mg, 0.137 mmol, 0.200 eq) in CH_2Cl_2 (0.68 ml) in a schlenk tube under an atmosphere of Ar. The reaction was stirred vigorously overnight. The reaction mixture was taken up in THF/H_2O = 5/1 (6.8 ml) and added to a freshly prepared solution of TBAF (ca. 100 mg TBAB in sat. aq. KF solution (1.4 ml) brought to pH 7 by the addition of 1.0 M AcOH). The mixture was stirred vigorously for 1 h at which point the phases were separated. The reaction was diluted with H_2O (20 ml) and the aqueous phase was extracted with EtOAc (3 x 20 ml). The combined organic layers were washed with brine, dried over Na_2SO_4, filtered, and concentrated *in vacuo*. The crude product was taken up in Et_2O (10 ml), filtered through a plug of celite, and concentrated *in vacuo*. The crude product was purified by silica gel column chromatography (pentane/Et_2O = 9/1 to 8/2) to provide the title compound as a colourless oil in 64% yield (94 mg).

^{1}H NMR (400 MHz, Acetone-d_6) δ(ppm): 7.40 – 7.31 (m, 4H, $ArC\underline{H}$), 7.28 (m, 1H, $ArC\underline{H}$), 5.79 (tt, J = 3.5, 1.5 Hz, 1H, $C{=}C\underline{H}$), 4.50 (s, 2H, $C\underline{H_2}Ph$), 4.03 – 3.99 (m, 2H, $C\underline{H_2}OCH_2Ph$), 2.87 – 2.82 (m, 2H, $CHC\underline{H_2}C({=}O)$), 2.53 – 2.41 (m, 4H, $C\underline{H_2}CH_2C({=}O)$ and $CH_2C\underline{H_2}C({=}O)$).

^{13}C NMR (151 MHz, Acetone-d_6) δ(ppm): 209.0 ($\underline{C}{=}O$), 139.8 ($Ar\underline{C}$), 136.8 ($\underline{C}{=}CH$), 129.1 ($Ar\underline{C}H$), 128.4 ($Ar\underline{C}H$), 128.2 ($Ar\underline{C}H$), 122.0 ($C{=}\underline{C}H$), 73.9 ($\underline{C}H_2OCH_2Ph$), 72.4 ($CH_2O\underline{C}H_2Ph$), 39.8 ($CH\underline{C}H_2C({=}O)$), 38.7 ($CH_2\underline{C}H_2C({=}O)$), 26.7 ($\underline{C}H_2CH_2C({=}O)$).

IR (film) ν_{max}/cm^{-1}: 2980, 2889, 2852 (C-H), 1714 (C=O), 1073 (C-O), 737, 697 (C-H).

HRMS (ES+) exact mass calculated for $[M+Na]^+$ $(C_{14}H_{16}NaO_2)^+$ requires *m/z* 239.1043, found *m/z* 239.1045.

5',6'-dihydro-[1,1':2',1''-terphenyl]-4'(3'H)-one (1p)

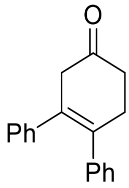

According to a literature procedure,[6] 1,2-diphenylethyne (96.2 mg, 0.54 mmol, 1.00 eq) and (buta-1,3-dien-2-yloxy)trimethylsilane (119 mg, 0.810 mmol, 1.50 eq) were added sequentially to a solution of $CoBr_2$(DPPE) (33.3 mg, 0.0540 mmol, 0.100 eq), ZnI_2 (32.5 mg, 0.108 mmol, 0.200 eq) and Zn (7.06 mg, 0.108 mmol, 0.200 eq) in CH_2Cl_2 (0.54 ml). The reaction was stirred vigorously under Ar overnight. The reaction mixture was taken up in THF/H_2O = 5/1 (5.4 ml) and added to a freshly prepared solution of TBAF (ca. 100 mg TBAB in sat. aq. KF solution (1.08 ml) brought to pH 7 by the addition of 1.0 M AcOH). The mixture was stirred vigorously for 1 h at which point the phases were separated. The organic layer was taken up in Et_2O (10 ml), washed with H_2O (3 x 20 ml), brine, dried over Na_2SO_4, filtered and concentrated *in vacuo.* The crude product was purified by trituration with Et_2O to afford the title compound as a white solid in 26% yield (35.3 mg).

^{1}H NMR (500 MHz, $CDCl_3$) δ(ppm): 7.18 – 7.08 (m, 6H, Ar<u>H</u>), 7.00 (m, 4H, Ar<u>H</u>), 3.35 (t, *J* = 1.5 Hz, 2H, CC<u>H2</u>C(=O)), 2.94 (tt, *J* = 7.0, 1.5 Hz, 2H, C<u>H2</u>CH2C(=O)), 2.72 (t, *J* = 7.0 Hz, 2H, CH2C<u>H2</u>C(=O));

^{13}C NMR (126 MHz, $CDCl_3$) δ(ppm): 210.2 (<u>C</u>=O), 141.8 (Ar<u>C</u>), 141.1 (Ar<u>C</u>), 135.6 (<u>C</u>=CCH2C(=O)), 132.0 (C=<u>C</u>CH2C(=O)), 129.0 (Ar<u>C</u>H), 129.0 (Ar<u>C</u>H), 128.2 (Ar<u>C</u>H), 128.1 (Ar<u>C</u>H), 126.8 (Ar<u>C</u>H), 126.7 (Ar<u>C</u>H), 45.9 (C<u>C</u>H2C(=O)), 38.8 (CH2<u>C</u>H2C(=O)), 32.0 (<u>C</u>H2CH2C(=O));

IR (powder) ν_{max}/cm^{-1}: 1714 (C=O), 1666 (C=C), 754, 698 (C-H).

Data is consistent with that published in the literature.[7]

3,4-diethylcyclohex-3-en-1-one (1q)

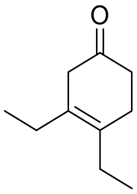

According to a literature procedure,[6] hex-3-yne(500 mg, 6.09 mmol, 1.00 eq) and (buta-1,3-dien-2-yloxy)trimethylsilane (1.61 ml, 9.13 mmol, 1.50 eq) were added sequentially to a solution of $CoBr_2$(py-imine) (270 mg, 0.609 mmol, 0.100 eq), ZnI_2 (389 mg, 1.22 mmol, 0.200 eq) and Zn (79.6 mg, 1.22 mmol, 0.200 eq) in CH_2Cl_2 (6 ml). The reaction was stirred vigorously under Ar overnight. The reaction mixture was taken up in THF/H_2O = 5/1 (60 ml) and added to a freshly prepared solution of TBAF (1 g of TBAB in sat. aq. KF solution (12 ml) brought to pH 7 by the addition of 1.0 M AcOH). The mixture was stirred vigorously for 1 h at which point Et_2O (30 ml) was added and the phases were separated. The aqueous layer was extracted with Et_2O (2 x 30 ml) and the combined organic layers were washed with brine, dried over Na_2SO_4, filtered and concentrated *in vacuo*. The crude product was purified by silica gel column chromatography (pentane/Et_2O = 9/1) to afford the desired compound as a colourless oil in 66% yield (616 mg).

^{1}H NMR (500 MHz, Benzene-d_6) δ(ppm): 2.59 (s, 2H, C$C\underline{H_2}$C(=O)), 2.12 (t, *J* = 7.0 Hz, 2H, $CH_2C\underline{H_2}$C(=O)), 1.92 (t, *J* = 7.0 Hz, 2H, $C\underline{H_2}CH_2$C(=O)), 1.81 (q, *J* = 7.5 Hz, 2H, $CH_3C\underline{H_2}C=CCH_2$C(=O)), 1.73 (q, *J* = 7.5 Hz, 2H, $CH_3C\underline{H_2}CCH_2$C(=O)), 0.79 (t, *J* = 7.5 Hz, 3H, $C\underline{H_3}CH_2C=CCH_2$C(=O)), 0.74 (t, *J* = 7.5 Hz, 3H, $C\underline{H_3}CH_2CCH_2$C(=O)).

^{13}C NMR (126 MHz, C_6D_6) δ(ppm): 208.6 ($\underline{C}$=O), 132.2 ($\underline{C}=CCH_2$C(=O)), 129.5 ($C=\underline{C}CH_2$C(=O)), 43.1 ($C\underline{C}H_2$C(=O)), 39.0 ($CH_2\underline{C}H_2$C(=O)), 29.1 ($\underline{C}H_2CH_2$C(=O)), 25.7 (2 x $\underline{C}H_2CH_3$), 13.1 ($\underline{C}H_3CH_2C=CCH_2$C(=O)), 13.0 ($\underline{C}H_3CH_2CCH_2$C(=O)).

HRMS (ES+) exact mass calculated for $[M+H]^+$ $(C_{10}H_{17}O)^+$ requires *m/z* 153.1274, found *m/z* 153.1275.

3,4-dipropylcyclohex-3-en-1-one (1r)

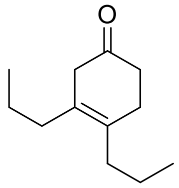

According to a literature procedure,[6] oct-4-yne (671 mg, 6.09 mmol, 1.00 eq) and (buta-1,3-dien-2-yloxy)trimethylsilane (1.61 ml, 9.13 mmol, 1.50 eq) were added sequentially to a solution of $CoBr_2$(py-imine) (270 mg, 0.609 mmol, 0.100 eq), ZnI_2 (389 mg, 1.22 mmol, 0.200 eq) and Zn (79.6 mg, 1.22 mmol, 0.200 eq) in CH_2Cl_2 (6 ml). The reaction was stirred vigorously under Ar overnight. The reaction mixture was taken up in THF/H_2O = 5/1 (60 ml) and added to a freshly prepared solution of (1 g of TBAB in sat. aq. KF solution (12 ml) brought to pH 7 by the addition of 1.0 M AcOH). The mixture was stirred vigorously for 1 h at which point Et_2O (30 ml) was added and the phases were separated. The aqueous layer was extracted with Et_2O (2 x 30 ml) and the combined organic layers were washed with brine, dried over Na_2SO_4, filtered and concentrated *in vacuo*. The crude product was purified by silica gel column chromatography (pentane/Et_2O = 95/5 followed by pentane/CH_2Cl_2 = 7/3 to 6/4 to remove an impurity) to afford the desired compound as a colourless oil in 21% yield (233 mg).

^{1}H NMR (500 MHz, Benzene-d_6) δ(ppm): 2.61 (s, 2H, $C\underline{H_2}$C(=O)), 2.13 (t, *J* = 7.0 Hz, 2H, $CH_2C\underline{H_2}$C(=O)), 1.98 – 1.91 (m, 2H, $C\underline{H_2}CH_2$C(=O)), 1.90 – 1.83 (m, 2H, $CH_3CH_2C\underline{H_2}C{=}CCH_2$C(=O)), 1.82 – 1.76 (m, 2H, $CH_3CH_2C\underline{H_2}CCH_2$C(=O)), 1.27 – 1.14 (m, 4H, $CH_3C\underline{H_2}CH_2C{=}CCH_2$C(=O) and $CH_3C\underline{H_2}CH_2CCH_2$C(=O)), 0.80 (t, *J* = 7.5 Hz, 3H, $C\underline{H_3}CH_2CH_2C{=}CCH_2$C(=O)), 0.76 (t, *J* = 7.4 Hz, 3H, $C\underline{H_3}CH_2CH_2CCH_2$C(=O)).

^{13}C NMR (126 MHz, Benzene-d_6) δ(ppm): 208.6 ($\underline{C}$=O), 131.6 ($\underline{C}{=}CCH_2$C(=O)), 128.7 ($C{=}\underline{C}CH_2$C(=O)), 43.7 ($C\underline{C}H_2$C(=O)), 38.8 ($CH_2\underline{C}H_2$C(=O)), 34.9 ($CH_3CH_2\underline{C}H_2C{=}CCH_2$C(=O)), 34.7 ($CH_3CH_2\underline{C}H_2CCH_2$C(=O)), 29.4 ($\underline{C}H_2CH_2$C(=O)), 21.7 ($CH_3\underline{C}H_2CH_2C{=}CCH_2$C(=O)), 21.5 ($CH_3\underline{C}H_2CH_2CCH_2$C(=O)), 14.2 ($\underline{C}H_3CH_2CH_2C{=}CCH_2$C(=O)), 14.1 ($\underline{C}H_3CH_2CH_2CCH_2$C(=O)).

IR (film) ν_{max}/cm^{-1}: 2959, 2871 (C-H), 1717 (C=O).

HRMS (ES+) exact mass calculated for $[M+H]^+$ $(C_{12}H_{21}O)^+$ requires *m/z* 181.1587, found *m/z* 181.1589.

6-methyl-4,5-dihydro-[1,1'-biphenyl]-3(2H)-one (1s)

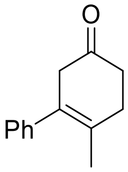

According to a literature procedure,[6] prop-1-yn-1-ylbenzene (0.348 mg, 3.00 mmol, 1.00 eq) and (buta-1,3-dien-2-yloxy)trimethylsilane (0.780 ml, 4.50 mmol, 1.50 eq) were added sequentially to a solution of $CoBr_2$(py-imine) (132 mg, 0.300 mmol, 0.100 eq), ZnI_2 (192 mg, 0.600 mmol, 0.200 eq) and Zn (39.3 mg, 0.600 mmol, 0.200 eq) in CH_2Cl_2 (3 ml). The reaction was stirred vigorously under Ar overnight. The reaction mixture was taken up in THF/H_2O = 5/1 (30 ml) and added to a freshly prepared solution of TBAF (600 mg of TBAB in sat. aq. KF solution (6 ml) brought to pH 7 by the addition of 1.0 M AcOH). The mixture was stirred vigorously for 1 h at which point Et_2O (20 ml) was added and the phases were separated. The aqueous layer was extracted with Et_2O (2 x 20 ml) and the combined organic layers were washed with brine, dried over Na_2SO_4, filtered and concentrated *in vacuo.* The crude product was purified by silica gel column chromatography (pentane/Et_2O = 95/5 to 9/1) to afford the desired compound as a colourless oil in 40% yield (223 mg).

^{1}H NMR (500 MHz, Acetone-d_6) δ(ppm): 7.41 – 7.31 (m, 2H, Ar$\underline{H}$), 7.30 – 7.17 (m, 3H, Ar$\underline{H}$), 3.09 – 3.04 (m, 2H, $C\underline{H_2}C$(=O)), 2.59 – 2.48 (m, 4H, $C\underline{H_2}C\underline{H_2}C$(=O)), 1.72 – 1.69 (m, 3H, $C\underline{H_3}$).

^{13}C NMR (126 MHz, Acetone-d_6) δ(ppm): 209.1 ($\underline{C}$=O), 142.8 (Ar$\underline{C}$), 130.8 ($CH_3\underline{C}$=C or CH_3C=$\underline{C}$), 130.8 ($CH_3\underline{C}$=C or CH_3C=$\underline{C}$), 129.1 (Ar$\underline{C}$H), 129.1 (Ar$\underline{C}$H), 127.5 (Ar$\underline{C}$H), 45.8 ($C\underline{C}H_2C$(=O)), 38.9 ($CH_2\underline{C}H_2C$(=O)), 32.1 ($\underline{C}H_2CH_2C$(=O)), 20.3 ($\underline{C}H_3$).

IR (film) ν_{max}/cm^{-1}: 2980, 2908, 2889 (C-H), 1713 (C=O).

HRMS (ES+) exact mass calculated for $[M+H]^+$ $(C_{13}H_{15}O)^+$ requires *m/z* 187.1117, found *m/z* 187.1120.

4'-methoxy-6-methyl-4,5-dihydro-[1,1'-biphenyl]-3(2H)-one (1t)

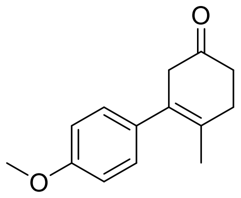

According to a literature procedure,[6] 1-methoxy-4-(prop-1-yn-1-yl)benzene (439 mg, 3.00 mmol, 1.00 eq) and (buta-1,3-dien-2-yloxy)trimethylsilane (0.780 ml, 4.50 mmol, 1.50 eq) were added sequentially to a solution of $CoBr_2$(py-imine) (132 mg, 0.300 mmol, 0.100 eq), ZnI_2 (192 mg, 0.600 mmol, 0.200 eq) and Zn (39.3 mg, 0.600 mmol, 0.200 eq) in CH_2Cl_2 (3 ml). The reaction was stirred vigorously under Ar overnight. The reaction mixture was taken up in THF/H_2O = 5/1 (30 ml) and added to a freshly prepared solution of TBAF (ca. 600 mg TBAB in sat. aq. KF solution (6 ml) brought to pH 7 by the addition of AcOH). The mixture was stirred vigorously for 1 h at which point the phases were separated. The organic layer was taken up in Et_2O (20 ml), washed with H_2O (3 x 30 ml), brine, dried over Na_2SO_4, filtered and concentrated *in vacuo*. The crude product was purified by silica gel column chromatography (pentane/Et_2O = 95/5 to 9/1) to afford the desired compound as a yellow oil in 36% yield (234 mg).

^{1}H NMR (500 MHz, Acetone-*d*$_6$) δ(ppm): 7.16 – 7.10 (m, 2H, ArH), 6.94 – 6.89 (m, 2H, ArH), 3.80 (s, 3H, OCH_3), 3.06 – 3.01 (m, 2H, CCH_2C(=O)), 2.56 – 2.46 (m, 4H, CH_2CH_2C(=O)), 1.72 – 1.69 (m, 3H, CCH_3).

^{13}C NMR (126 MHz, Acetone-*d*$_6$) δ(ppm): 209.2 (C=O), 159.4 (ArC), 135.0 (ArC), 130.3 (CH_3C=C or CH_3C=C), 130.3 (CH_3C=C or CH_3C=C), 130.2 (ArCH), 114.4 (ArCH), 55.5 (OCH_3), 45.9 (CCH_2C(=O)), 38.9 (CH_2CH_2C(=O)), 32.1 (CH_2CH_2C(=O)), 20.4 (CH_3C).

IR (film) ν_{max}/cm^{-1}: 2910, 2838 (C-H), 1715 (C=O), 1510 (C=C), 1244 (C-O).

HRMS (ES+) exact mass calculated for $[M+H]^+$ $(C_{14}H_{17}O_2)^+$ requires *m/z* 217.1223, found *m/z* 217.1226.

1.3 Optimisation of reaction conditions

Reactions were carried out on a 0.065 mmol scale following general procedures **D** and **E**.

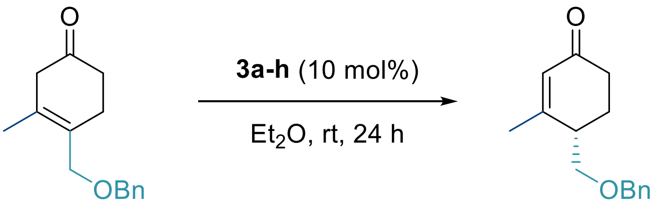

1a **2a**

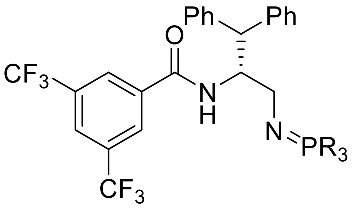

3a R = Ph, 17%, 1% ee
3b R = PMP, 33%, -7% ee

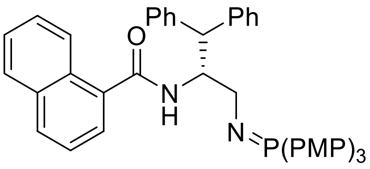

3c
27%, 1% ee

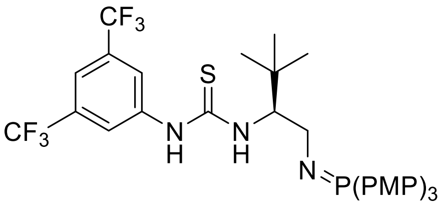

3d
53%, -14% ee

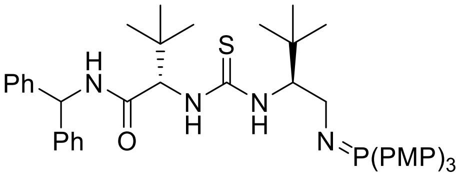

3e
67%[a], 18% ee

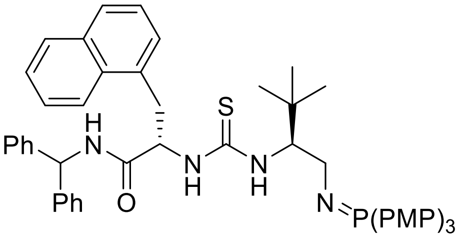

3f
19%[a], 10% ee

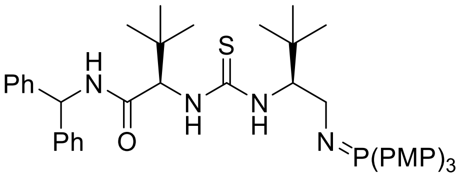

3g
97%, 85% ee

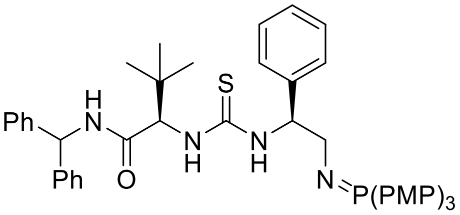

3h
83%, 93% ee

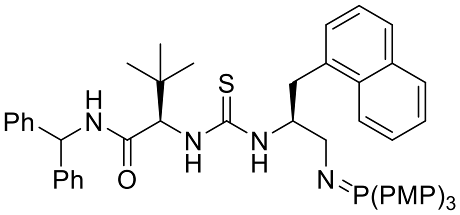

3i
90%, 99% ee[b]

[a]NMR yield.[b]Reaction was carried out with 0.26 mmol of substrate.

Reactions were carried out on a 0.065 mmol scale following general procedures **D** and **E**.

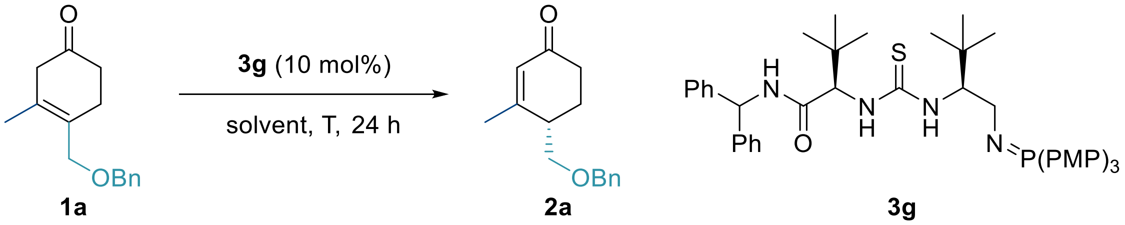

entry	*solvent*	*temp (°C)*	*conc (M)*	*yield (%)*	*ee (%)*
1	Et_2O	rt	0.10	97	85
2	THF	rt	0.10	49	46
3	TBME	rt	0.10	93	69

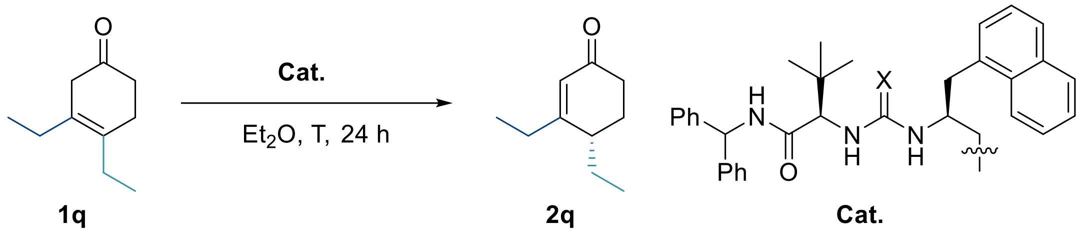

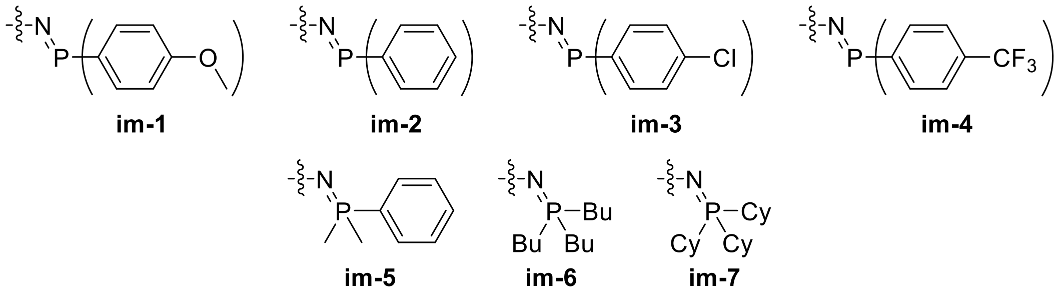

entry	*X*	*im*	*loading (mol%)*	*temp (°C)*	*conc (M)*	*yield (%)*	*ee (%)*
1	S	im-1	10	rt	0.10	0	N/A
2	S	im-2	10	rt	0.10	0	N/A
3	S	im-3	10	rt	0.10	0	N/A
4	S	im-4	10	rt	0.10	0	N/A
5	S	im-5	10	rt	0.10	trace	88
6	S	im-6	10	rt	0.10	20	87
7	S	im-6	10	rt	0.35	39	87
8	O	im-6	10	rt	0.10	45	98
9	O	im-6	10	rt	0.20	65	98
10	O	im-6	10	rt	0.40	32	97
11	O	im-6	10	40	0.20	45	98
12	O	im-6	15	rt	0.15	63	97
13	O	im-6	20	rt	0.20	63	97

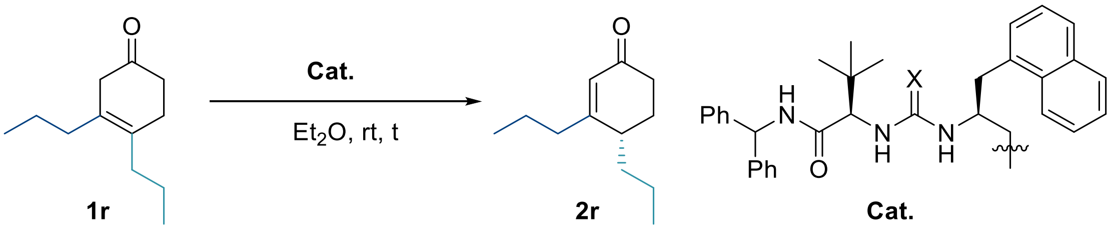

entry	*X*	*im*	*loading (mol%)*	*time (h)*	*conc (M)*	*yield (%)*	*ee (%)*
1	O	im-6	10	24	0.20	39	97
2	O	im-7	10	24	0.20	trace	N/D
3	O	im-6	10	48	0.35	42	91
4	O	im-6	15	24	0.20	50	97
5	O	im-6	20	24	0.20	36	97

1.4 The BIMP catalysed enantioselective prototropic shift of β,γ-unsaturated cyclohexenones

General procedure D: BIMP formation

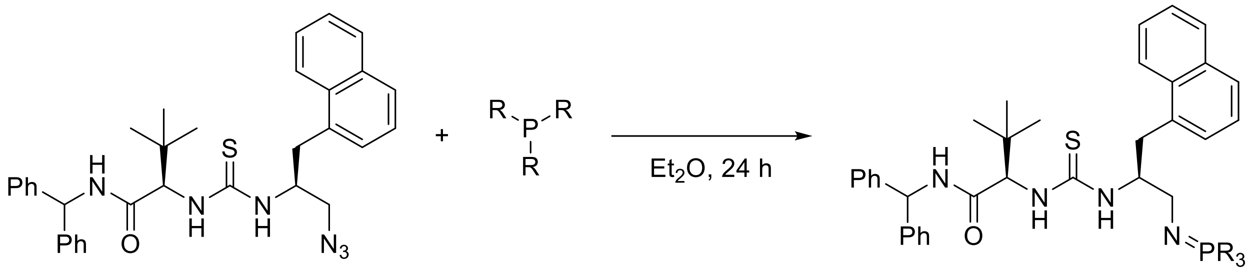

To a mass spectrometry vial was weighed the desired azide (1.00 eq) and the desired phosphine (1.00 eq). The vial was placed under an atmosphere of Ar and Et_2O (0.05M) was added. The cap was replaced and sealed with parafilm® and the mixture was stirred for 18 – 24 h.

General procedure E: The prototropic shift

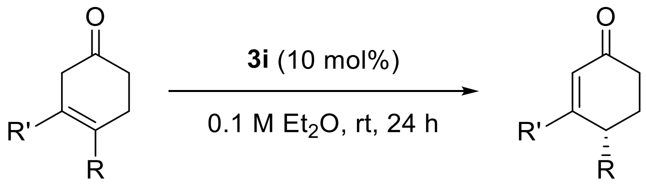

The preformed BIMP catalyst **3i** (0.100 eq) was concentrated to dryness under a stream of Ar without exposure to air. The desired substrate (1.00 eq) was taken up in Et_2O (0.10 M) under an atmosphere of Ar and transferred to the vial containing the BIMP catalyst. The reaction was stirred for 24 h under Ar at which point the reaction mixture is transferred directly onto silica gel and the products were purified by silica gel column chromatography.

General procedure F: The prototropic shift

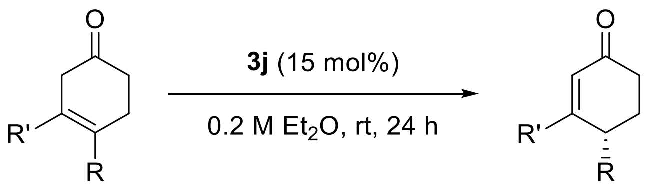

The preformed BIMP catalyst **3j** (0.15 eq) was concentrated to dryness under a stream of Ar without exposure to air. The desired substrate (1.00 eq) was taken up in Et_2O (0.20 M) under an atmosphere of Ar and transferred to the vial containing the BIMP catalyst. The reaction was stirred for 24 h under Ar at which point the reaction mixture was transferred directly onto silica gel and the products were purified by silica gel column chromatography.

(S)-4-((benzyloxy)methyl)-3-methylcyclohex-2-en-1-one (2a)

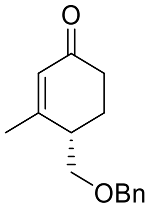

The title compound was prepared according to general procedure **E** from 4-((benzyloxy)methyl)-3-methylcyclohex-3-en-1-one (**1a**) (59.9 mg, 0.260 mmol). The crude reaction mixture was transferred directly onto silica and purified by silica gel column chromatography (pentane/EtOAc = 9/1 to 8/2) to provide the title compound as a colourless oil in 90% yield (54.2 mg) and 99% ee. [determined by HPLC chiralpak AS-H, hexane/isopropanol = 70/30, 1 ml/min, λ = 220 nm, t(major) = 15.19 min, t(minor) = 22.07 min].

^{1}H NMR (500 MHz, $CDCl_3$) δ(ppm): 7.41 – 7.26 (m, 5H, Ar<u>H</u>), 5.92 – 5.88 (m, 1H, C<u>H</u>C(=O)), 4.57 (d, *J* =12.0 Hz, 1H, C$\underline{H_A}H_B$Ph), 4.51 (d, *J* =12.0 Hz, 1H, C$H_A\underline{H_B}$Ph), 3.60 (d, *J* = 6.0 Hz, 2H, CHC$\underline{H_2}$O), 2.57 (p, *J* = 5.5 Hz, 1H, C<u>H</u>CH_2), 2.45 (ddd, *J* = 17.5, 10.0, 5.5 Hz, 1H, one of C$\underline{H_A}H_B$C(=O)), 2.35 – 2.26 (m, 1H, one of C$H_A\underline{H_B}$C(=O)), 2.11 (m, 2H, C$\underline{H_2}CH_2$C(=O)), 1.98 (m, 3H, C$\underline{H_3}$).

^{13}C NMR (126 MHz, $CDCl_3$) δ(ppm): 199.4 (<u>C</u>=O), 162.4 (<u>C</u>CH_3), 138.0 (Ar<u>C</u>), 128.6 (Ar<u>C</u>H), 128.3 (<u>C</u>=CCH_3), 127.9 (Ar<u>C</u>H), 127.7 (Ar<u>C</u>H), 73.4 (<u>C</u>H_2Ph), 70.5 (CH<u>C</u>H_2O), 40.4 (<u>C</u>HCH_2O), 34.6 (<u>C</u>H_2C(=O)), 25.8 (<u>C</u>H_2CH_2C(=O)), 23.1 (<u>C</u>H_3).

$[\alpha]_D^{25}$ = -91.22 (*c* 2.01, $CHCl_3$) (99% ee); lit: $[\alpha]_D^{25}$ = +65.8 (*c* 1.98, $CHCl_3$) 81% ee, (R)-4-((benzyloxy)methyl)-3-methylcyclohex-2-en-1-one).[8a] $[\alpha]_D^{22}$ = +62.6 (*c* 1.05, $CHCl_3$), (R)-4-((benzyloxy)methyl)-3-methylcyclohex-2-en-1-one).[8b]

Data is consistent with that published in the literature.[8a-b]

(S)-4-(methoxymethyl)-3-methylcyclohex-2-en-1-one (2b)

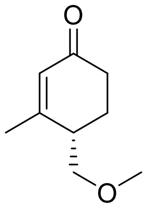

The title compound was prepared according to general procedure **E** from 4-(methoxymethyl)-3-methylcyclohex-3-en-1-one (**1b**) (20.0 mg, 0.130 mmol). The crude reaction mixture was transferred directly onto silica and purified by silica gel column chromatography (pentane/Et_2O = 7/3) to provide the title compound as a colourless oil in 58% yield (11.6 mg) and 96% ee. [determined by HPLC chiralpak AD-H, hexane/isopropanol = 95/5, 1 ml/min, λ = 230 nm, t(major) = 8.24 min, t(minor) = 8.90 min].

^{1}H NMR (500 MHz, Benzene-*d*$_6$) δ(ppm): 5.92 – 5.90 (m, 1H, C<u>H</u>C(=O)), 2.97 – 2.90 (m, 5H, OC<u>H$_3$</u> and OC<u>H$_2$</u>), 2.30 (ddd, *J* = 17.0, 10.0, 5.0 Hz, 1H, one of C<u>H$_A$</u>H$_B$C(=O)), 2.10 (ddd, *J* = 17.0, 7.5, 5.0 Hz, 1H, one of CH$_A$<u>H$_B$</u>C(=O)), 1.96 (p, *J* = 5.5 Hz, 1H, C<u>H</u>CH$_2$O), 1.79 – 1.71 (m, 1H, one of C<u>H$_A$</u>H$_B$CH$_2$C(=O)), 1.59 (ddt, *J* = 13.5, 10.0, 5.0 Hz, 1H, one of CH$_A$<u>H$_B$</u>CH$_2$C(=O)), 1.47 (s, 3H, C<u>H$_3$</u>C).

^{13}C NMR (126 MHz, Benzene-*d*$_6$) δ(ppm): 197.1 (<u>C</u>=O), 160.4 (<u>C</u>CH$_3$), 128.6 (C=<u>C</u>H), 73.2 (<u>C</u>H$_2$O), 58.6 (<u>C</u>H$_3$O), 40.3 (<u>C</u>HCH$_2$O), 34.9 (<u>C</u>H$_2$C(=O)), 25.9 (<u>C</u>H$_2$CH$_2$C(=O)), 22.6 (C<u>C</u>H$_3$).

IR (film) ν_{max}/cm^{-1}: 2926, 2873 (C-H), 1667 (C=O), 1625 (C=C), 1207 (C-O).

HRMS (ES+) exact mass calculated for $[M+H]^+$ $(C_9H_{15}O_2)^+$ requires *m/z* 155.1067, found *m/z* 155.1066.

IR (film) ν_{max}/cm^{-1}: 2926, 2873, 2830 (C-H), 1666 (C=O), 1112 (C-O).

$[\alpha]_D^{25}$ = -84 (*c* 0.265, $CHCl_3$).

(S)-4-((allyloxy)methyl)-3-methylcyclohex-2-en-1-one (2c)

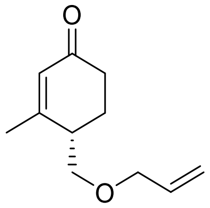

The title compound was prepared according to general procedure **E** from 4-((allyloxy)methyl)-3-methylcyclohex-3-en-1-one (**1c**) (23.4 mg, 0.130 mmol). The crude reaction mixture was transferred directly onto silica and purified by silica gel column chromatography (pentane/Et_2O = 8/2) to provide the title compound as a colourless oil in 78% yield (18.3 mg) and 98% ee. [determined by HPLC chiralpak AS-H, hexane/isopropanol = 70/30, 1 ml/min, λ = 240 nm, t(major) = 12.70 min, t(minor) = 17.07 min].

^{1}H NMR (400 MHz, $CDCl_3$) δ(ppm): 5.94 – 5.83 (m, 2H, CH=CH_2 and CHC(=O)), 5.26 (dq, *J* = 17.0, 1.5 Hz, 1H, one of CH=CH_AH_B), 5.18 (dq, *J* = 10.5, 1.5 Hz, 1H, one of CH=CH_AH_B), 4.04 – 3.92 (m, 2H, CH_2CH=CH_2), 3.57 – 3.54 (m, 2H, CH_2COCH_2CH=CH_2), 2.54 (p, *J* = 5.5 Hz, 1H, $CH_2CH_2CHCH_2O$), 2.51 – 2.41 (m, 1H, one of CH_AH_BC(=O)), 2.35 – 2.25 (m, 1H, CH_AH_BC(=O)), 2.13 – 2.03 (m, 2H, CH_2CHCH_2O), 1.99 (dd, *J* = 1.5, 1.0 Hz, 3H, CH_3).

^{13}C NMR (101 MHz, $CDCl_3$) δ(ppm): 199.5 (C=O), 162.5 (CCH_3), 134.5 (CH=CH_2), 128.3 (C=CCH_3), 117.4 (CH=CH_2), 72.3 (CH_2CH=CH_2), 70.5 (CH_2OCH_2CH=CH_2), 40.4 ($CHCH_2OCH_2$CH=CH_2), 34.6 (CH_2C(=O)), 25.8 (CH_2CH_2C(=O)), 23.2 (CH_3).

IR (film) ν_{max}/cm^{-1}: 2980, 2867 (C-H), 1667 (C=O), 1625 (C=C), 1135 (C-O), 925 (C=C-H).

HRMS (ES+) exact mass calculated for $[M+H]^+$ $(C_{11}H_{17}O_2)^+$ requires *m/z* 181.1223, found *m/z* 181.1223.

$[\alpha]_D^{25}$ = -116.7 (*c* 0.185, $CHCl_3$).

(S)-3-methyl-4-(((3-methylbut-2-en-1-yl)oxy)methyl)cyclohex-2-en-1-one (2d)

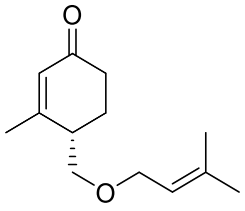

The title compound was prepared according to general procedure **E** from 3-methyl-4-(((3-methylbut-2-en-1-yl)oxy)methyl)cyclohex-3-en-1-one (**1d**) (27.1 mg, 0.130 mmol). The crude reaction mixture was transferred directly onto silica and purified by silica gel column chromatography (pentane/Et_2O = 75/25) to provide the title compound as a yellow oil in 85% yield (23.0 mg) and 99% ee. [determined by HPLC chiralpak AS-H, hexane/isopropanol = 95/5, 1 ml/min, λ = 240 nm, t(major) = 39.05 min, t(minor) = 56.23 min].

^{1}H NMR (500 MHz, $CDCl_3$) δ(ppm): 5.89 (t, *J* = 1.5 Hz, 1H, C$\underline{H}$C(=O)), 5.36 – 5.29 (m, 1H, C$\underline{H}$C(CH_3)$_2$), 4.04 – 3.92 (m, 2H, OC$\underline{H_2}$CH=C), 3.54 (d, *J* = 6.0 Hz, 2H, C$\underline{H_2}$OCH_2CH=C), 2.53 (p, J = 5.5, 1H, C$\underline{H}$$CH_2$$CH_2$), 2.47 (ddd, *J* = 17.0, 9.5, 5.5 Hz, 1H, one of C$\underline{H_A}$$H_B$C(=O)), 2.30 (ddd, *J* = 17.0, 6.5, 5.5 Hz, 1H, one of $CH_A$$\underline{H_B}$C(=O)), 2.14 – 2.02 (m, 2H, C$\underline{H_2}$$CH_2$C(=O)), 2.01 – 1.97 (m, 3H, C$\underline{H_3}$C=CHC(=O)), 1.75 (s, 3H, one of C(C$\underline{H_3}$)$_A$(CH_3)$_B$), 1.67 (s, 3H, one of C(CH_3)$_A$(C$\underline{H_3}$)$_B$).

^{13}C NMR (126 MHz, $CDCl_3$) δ(ppm): 199.6 ($\underline{C}$=O), 162.7 ($\underline{C}$=CHC(=O)), 137.5 ($\underline{C}$(CH_3)$_2$), 128.3 (C=$\underline{C}$HC(=O)), 120.9 ($\underline{C}$H=C(CH_3)$_2$), 70.2 ($\underline{C}$$H_2OCH_2$CH=C), 67.8 ($CH_2O\underline{C}$$H_2$CH=C), 40.5 ($CH_2$$\underline{C}HCCH_3$), 34.6 ($CH_2$$\underline{C}$$H_2$C(=O)), 25.9 (one of C($\underline{C}$$H_3$)$_A$($CH_3$)$_B$), 25.7 ($\underline{C}$$H_2$$CH_2$C(=O)), 23.2 ($\underline{C}$$H_3$C=CHC(=O)), 18.2 (one of C(CH_3)$_A$($\underline{C}$$H_3$)$_B$).

HRMS (ES+) exact mass calculated for $[M+H]^+$ $(C_{13}H_{21}O_2)^+$ requires *m/z* 209.1536, found *m/z* 209.1538.

IR (film) ν_{max}/cm^{-1}: 2970, 2914, 2865, (C-H), 1667 (C=O), 1625 (C=C), 1089 (C-O).

$[\alpha]_D^{25}$ = -74.0 (*c* 0.115, $CHCl_3$).

(S)-3-methyl-4-((prop-2-yn-1-yloxy)methyl)cyclohex-2-en-1-one (2e)

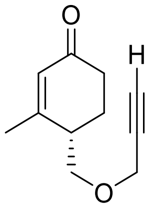

The title compound was prepared according to general procedure **E** from 3-methyl-4-((prop-2-yn-1-yloxy)methyl)cyclohex-3-en-1-one (**1e**) (23.2 mg, 0.130 mmol). The crude reaction mixture was transferred directly onto silica and purified by silica gel column chromatography (pentane/Et_2O = 8/2) to provide the title compound as a colourless oil in 96% yield (22.2 mg) and 99% ee. [determined by HPLC chiralpak AS-H, hexane/isopropanol = 70/30, 1 ml/min, λ = 230 nm, t(minor) = 25.83 min, t(major) = 27.16 min].

^{1}H NMR (400 MHz, Benzene-d_6) δ(ppm): 5.91 – 5.86 (m, 1H, CHC(=O)), 3.72 (dd, *J* = 16.0, 2.5 Hz, 1H, $CH_AH_BC{\equiv}CH$), 3.67 (dd, *J* = 16.0, 2.5 Hz, 1H, $CH_AH_BC{\equiv}CH$), 3.16 (dd, *J* = 9.0, 7.0 Hz, 1H, one of $COCH_AH_BCHCH_2CH_2$), 3.09 (dd, *J* = 9.0, 4.5 Hz, 1H, one of $COCH_AH_BCHCH_2CH_2$), 2.30 (ddd, *J* = 17.0, 10.0, 5.0 Hz, 1H, one of $CH_AH_BC(=O)$), 2.08 (ddd, *J* = 17.0, 7.5, 5.0 Hz, 1H, one of $CH_AH_BC(=O)$), 2.03 (t, *J* = 2.5 Hz, 1H, C≡CH), 1.96 (p, *J* = 6.0 Hz, 1H, $CHCH_2CH_2$), 1.81 – 1.70 (m, 1H, one of $CH_AH_BCH_2C(=O)$), 1.62 – 1.52 (m, 1H, one of $CH_AH_BCH_2C(=O)$), 1.47 (dd, *J* = 1.5, 1.0 Hz, 3H, CH_3).

^{13}C NMR (101 MHz, Benzene-d_6) δ(ppm): 197.0 (C=O), 160.0 (CCH_3), 128.7 (CHC(=O)), 79.9 (C≡CH), 74.8 (C≡CH), 70.1 (OCH_2CHCH_2), 58.2 ($CH_2C{\equiv}CH$), 40.1 ($CHCH_2CO$), 34.9 ($CH_2C(=O)$), 25.8 ($CH_2CH_2C(=O)$), 22.5 (CH_3).

IR (film) ν_{max}/cm^{-1}: 3248 (C≡C-H), 2916, 2869 (C-H), 2113 (C≡C), 1664 (C=O), 1625 (C=C), 1204 (C-O).

HRMS (ES+) exact mass calculated for $[M+H]^+$ $(C_{11}H_{15}O_2)^+$ requires *m/z* 179.1067, found *m/z* 179.1067.

$[\alpha]_D^{25}$ = -121.9 (*c* 0.130, $CHCl_3$).

(S)-4-(((tert-butyldimethylsilyl)oxy)methyl)-3-methylcyclohex-2-en-1-one (2f)

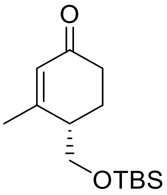

The title compound was prepared according to general procedure **E** from 4-(((tert-butyldimethylsilyl)oxy)methyl)-3-methylcyclohex-3-en-1-one (**1f**) (1.50 g, 5.90 mmol). The crude reaction mixture was flushed through a silica plug (Et_2O), concentrated *in vacuo* and then purified by silica gel column chromatography (pentane/Et_2O = 9/1) to provide the title compound as a colourless oil in 90% yield (1.35 g) and 99% ee. [determined by HPLC chiralpak AS-H, hexane/isopropanol = 90/10 to 70/30, 1 ml/min, λ = 230 nm, t(major) = 8.39 min, t(minor) = 11.86 min].

^{1}H NMR (500 MHz, Acetone-d_6) δ(ppm): 5.80 – 5.79 (m, 1H, C=C<u>H</u>), 3.89 (dd, *J* = 10.0, 6.0 Hz, 1H, C$\underline{H_A}H_B$O), 3.85 (dd, *J* = 10.0, 4.0 Hz, 1H, C$H_A\underline{H_B}$O), 2.49 (p, *J* = 5.5 Hz, 1H, C<u>H</u>CH_2O), 2.43 (ddd, *J* = 17.0, 9.5, 5.5 Hz, 1H, one of C$\underline{H_A}H_B$C(=O)), 2.20 (ddd, *J* = 17.0, 7.0, 5.5 Hz, 1H, one of C$H_A\underline{H_B}$C(=O)), 2.13 – 2.02 (m, 2H, C$\underline{H_2}$$CH_2$C(=O)), 2.02 – 1.99 (m, 3H, C=CC$\underline{H_3}$), 0.90 (s, 9H, C(C$\underline{H_3}$)$_3$), 0.09 (2 x s, 6H, Si(C$\underline{H_3}$)$_A$(C$\underline{H_3}$)$_B$).

^{13}C NMR (126 MHz, Acetone-d_6) δ(ppm): 198.3 (<u>C</u>=O), 162.7 (C=<u>C</u>CH_3), 128.6 (<u>C</u>=CCH_3), 64.4 (<u>C</u>H_2O), 43.0 (<u>C</u>HCH_2O), 35.3 (<u>C</u>H_2C(=O)), 26.4 (<u>C</u>$H_2$$CH_2$C(=O)), 26.2 (C(<u>C</u>$H_3$)$_3$), 22.9 (C=C<u>C</u>$H_3$), 18.8 (<u>C</u>($CH_3$)$_3$), -5.4 (one of Si(<u>C</u>H_3)$_A$(CH_3)$_B$), -5.4 (one of Si(CH_3)$_A$(<u>C</u>H_3)$_B$).

HRMS (ES+) exact mass calculated for $[M+H]^+$ $(C_{14}H_{27}O_2Si)^+$ requires *m/z* 255.1775, found *m/z* 255.1775.

IR (film) ν_{max}/cm^{-1}: 2953, 2929, 2857 (C-H), 1670 (C=O), 1626 (C=C), 1102 (C-O).[37]

$[\alpha]_D^{25}$ = -94.0 (*c* 0.205, $CHCl_3$).

(S)-4-(hydroxymethyl)-3-methylcyclohex-2-en-1-one (2g)

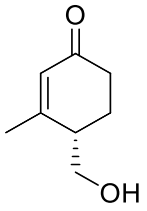

The title compound was prepared according to general procedure **E** from 4-(hydroxymethyl)-3-methylcyclohex-3-en-1-one (**1g**) (9.00 mg, 0.065 mmol). The crude reaction mixture was transferred directly onto silica and purified by silica gel column chromatography (Et_2O) to provide the title compound as a yellow oil in 67% yield (6.0 mg) and 85% ee. [determined by HPLC chiralpak AS-H, hexane/isopropanol = 70/30, 1 ml/min, λ = 230 nm, t(minor) = 12.56 min, t(major) = 15.93 min].

^{1}H NMR (400 MHz, $CDCl_3$) δ(ppm): 5.93 – 5.90 (m, 1H, $C\underline{H}C(=O)$), 3.87 – 3.75 (m, 2H, $C\underline{H_2}OH$), 2.57 – 2.42 (m, 2H, one of $C\underline{H_A}H_BC(=O)$ and $C\underline{H}CH_2OH$), 2.37 – 2.27 (m, 1H, one of $CH_A\underline{H_B}C(=O)$), 2.17 – 2.04 (m, 3H, $C\underline{H_2}CH_2C(=O)$ and $O\underline{H}$), 2.01 (s, 3H, $C\underline{H_3}$).

^{13}C NMR (101 MHz, $CDCl_3$) δ(ppm): 199.5 ($\underline{C}=O$), 162.2 ($\underline{C}CH_3$), 128.7 ($\underline{C}=CCH_3$), 63.3 ($\underline{C}H_2OH$), 42.4 ($C\underline{H}CH_2OH$), 34.5 ($CH_2\underline{C}H_2C(=O)$), 25.2 ($\underline{C}H_2CH_2C(=O)$), 23.2 ($\underline{C}H_3$).

IR (film) ν_{max}/cm^{-1}: 3406 (O-H), 2980, 2885 (C-H), 1649 (C=O), 1074 (C-O).

HRMS (ES+) exact mass calculated for $[M+H]^+$ $(C_8H_{13}O_2)^+$ requires *m/z* 141.0910, found *m/z* 141.0910.

Data is consistent with that published in the literature.[9]

$[\alpha]_D^{25}$ = -37.6 (*c* 1.005, $CHCl_3$). (96% ee)

(S)-4-((benzyloxy)methyl)cyclohex-2-en-1-one (2h)

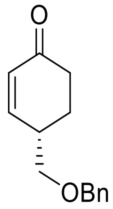

The title compound was prepared according to general procedure **E** from 4-((benzyloxy)methyl)cyclohex-3-en-1-one (**1h**) (28.1 mg, 0.130 mmol) at 30 °C. The crude reaction mixture was transferred directly onto silica and purified by silica gel column chromatography (pentane/Et_2O = 8/2). Mixed fractions were re-purified by silica gel column chromatography (pentane/Et_2O = 9/1) three times to provide the title compound as a colourless oil in 62% yield (17.5 mg) and 94% ee. [determined by HPLC chiralpak AS-H, hexane/isopropanol = 70/30, 1 ml/min, λ = 230 nm, t(minor) = 16.47 min, t(major) = 47.98 min].

^{1}H NMR (400 MHz, $CDCl_3$) δ(ppm): 7.41 – 7.28 (m, 5H, Ar$\underline{H}$), 6.95 (ddd, *J* = 10.0, 2.5, 1.5 Hz, 1H, C$\underline{H}$CHC(=O)), 6.04 (ddd, *J* = 10.0, 2.5, 1.0 Hz, 1H, CHC$\underline{H}$C(=O)), 4.55 (s, 2H, C$\underline{H_2}$Ph), 3.51 (dd, *J* = 9.0, 6.5 Hz, 1H, C$\underline{H_A}H_B$OCH_2Ph), 3.47 (dd, *J* = 9.0, 7.0 Hz, 1H, C$H_A\underline{H_B}$OCH_2Ph), 2.80 – 2.68 (m, 1H, C$\underline{H}$CH_2O), 2.57 – 2.47 (m, 1H, one of C$\underline{H_A}H_B$C(=O)), 2.44 – 2.33 (m, 1H, one of C$H_A\underline{H_B}$C(=O)), 2.18 – 2.06 (m, 1.5 Hz, 1H, one of C$\underline{H_A}H_B$CH_2C(=O)), 1.89 – 1.74 (m, 1H one of C$\underline{H_A}H_B$CH_2C(=O)).

^{13}C NMR (101 MHz, $CDCl_3$) δ(ppm): 199.7 ($\underline{C}$=O), 151.7 ($\underline{C}$HCHC(=O)), 138.1 (Ar$\underline{C}$), 130.2 (CH$\underline{C}$HC(=O)), 128.6 (Ar$\underline{C}$H), 128.0 (Ar$\underline{C}$H), 127.8 (Ar$\underline{C}$H), 73.5 ($\underline{C}H_2$Ph), 72.6 ($\underline{C}H_2$OCH_2Ph), 37.1 ($\underline{C}$HCH_2O), 36.9 ($\underline{C}H_2$C(=O)), 26.0 ($\underline{C}H_2$CH_2C(=O)).

$[\alpha]_D^{25}$= -86.5 (*c* 1.75, MeOH); lit: $[\alpha]_D^{26}$= -109.4 (*c* 3.58, MeOH) (S)-4-((benzyloxy)methyl)cyclohex-2-en-1-one.[10a] $[\alpha]_D^{25}$= -124.4 (*c* 1.72, MeOH) (R)-4-((benzyloxy)methyl)cyclohex-2-en-1-one.[10b]

Data is consistent with that published in the literature.[10a-c]

tert-butyl (S)-((2-methyl-4-oxocyclohex-2-en-1-yl)methyl)(phenyl)carbamate (2i)

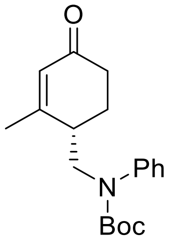

The title compound was prepared according to general procedure **E** from tert-butyl ((2-methyl-4-oxocyclohex-1-en-1-yl)methyl)(phenyl)carbamate (**1i**) (41 mg, 0.13 mmol). The crude reaction mixture was transferred directly onto silica and purified by silica gel column chromatography (pentane/Et_2O = 6/4) to provide the title compound as a colourless oil in 83% yield (34 mg) and 98% ee. [determined by HPLC chiralpak AD-H, hexane/isopropanol = 90/10, 1 ml/min, λ = 230 nm, t(major) = 6.16 min, t(minor) = 7.32 min].

^{1}H NMR (500 MHz, Acetone-d_6) δ(ppm): 7.43 – 7.37 (m, 2H, ArH), 7.37 – 7.33 (m, 2H, ArH), 7.24 (tt, J = 7.0, 1.5 Hz, 1H, ArH), 5.77 (s, 1H, CHC(=O)), 4.03 (dd, J = 14.5, 10.5 Hz, 1H, one of CH$_A$H$_B$N), 3.83 (dd, J = 14.5, 4.0 Hz, 1H, one of CH$_A$H$_B$N), 2.54 – 2.40 (m, 2H, CHCH$_2$N and one of CH$_A$H$_B$C(=O)), 2.11 (dt, J = 17.5, 4.5 Hz, 1H, one of CH$_A$H$_B$C(=O)), 2.02 – 1.96 (m, 2H, CH$_2$CH$_2$C(=O)), 1.90 (d, J = 1.5 Hz, 3H, C=CCH$_3$), 1.43 (s, 9H, C(CH$_3$)$_3$).

^{13}C NMR (126 MHz, Acetone-d_6) δ(ppm): 198.0 (C=O), 162.6 (C=CH), 155.3 (NC(=O)), 143.1 (ArC), 129.6 (ArCH), 128.3 (C=CH), 128.0 (ArCH), 126.9 (ArCH), 80.7 (C(CH$_3$)$_3$), 50.0 (CH$_2$N), 39.8 (CHCH$_2$N), 33.7 (CH$_2$C(=O)), 28.4 (C(CH$_3$)$_3$), 25.3 (CH$_2$CH$_2$C(=O)), 22.9 (C=CCH$_3$).

IR (film) ν_{max}/cm^{-1}: 2977 (C-H), 1692, 1667 (C=O), 1153 (C-N).

HRMS (ES+) exact mass calculated for $[M+Na]^+$ $(C_{19}H_{25}NNaO_3)^+$ requires m/z 338.1727, found m/z 338.1726.

$[\alpha]_D^{25}$ = -1.72 (c 0.145, $CHCl_3$).

tert-butyl (S)-((2-methyl-4-oxocyclohex-2-en-1-yl)methyl)(prop-2-yn-1-yl)carbamate (2j)

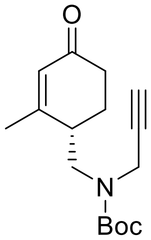

The title compound was prepared according to general procedure **E** from tert-butyl ((2-methyl-4-oxocyclohex-1-en-1-yl)methyl)(prop-2-yn-1-yl)carbamate (**1j**) (36.0 mg, 0.130 mmol) and left for 48 h. The crude reaction mixture was transferred directly onto silica and purified by silica gel column chromatography (pentane/Et_2O = 6/4) to provide the title compound as an off white solid in 75% yield (27.1 mg) and 96% ee. [determined by HPLC chiralpak AD, hexane/isopropanol = 90/10 to 70/30, 1 ml/min, λ = 240 nm, t(major) = 7.50 min, t(minor) = 8.62 min].

^{1}H NMR (500 MHz, Toluene-d_8, 363 K) δ(ppm): 5.78 (s, 1H, CHC(=O)), 3.94 – 3.73 (m, 2H, CH_2C≡CH), 3.38 – 3.28 (m, 1H, one of $CHCH_AH_BN$), 3.23 (dd, *J* = 14.5, 4.5 Hz, 1H, one of $CHCH_AH_BN$), 2.51 – 2.37 (m, 1H, one of $CH_AH_BC(=O)$), 2.37 – 2.28 (m, 1H, $CHCH_2N$), 2.17 – 2.12 (m, 1H, one of $CH_AH_BC(=O)$), 1.88 – 1.81 (m, 1H, C≡CH), 1.78 – 1.67 (m, 2H, $CH_2CH_2C(=O)$), 1.60 (s, 3H, $CH_3C=C$), 1.38 (s, 9H, $C(CH_3)_3$).

^{13}C NMR (126 MHz, Toluene-d_8, 363 K) δ(ppm): 195.8 (CHC(=O)), 159.3 (C=CCH_3), 155.0 (NC(=O)), 128.8 (CHC(=O)), 80.5 ($OC(CH_3)_3$), 79.9 (C≡CH), 72.0 (C≡CH), 47.6 ($CHCH_2N$), 39.2 ($CHCH_2N$), 37.1 (CH_2C≡CH), 33.7 ($CH_2CH_2C(=O)$), 28.4 ($C(CH_3)_3$), 25.5 ($CH_2CH_2C(=O)$), 22.3 (C=CCH_3).

IR (powder) ν_{max}/cm^{-1}: 3235 (C≡C-H), 2981, 2934, 2892, 2872 (C-H), 2110 (C≡C), 1667 (C=O), 1632 (C=C).

HRMS (ES+) exact mass calculated for $[M+H]^+$ $(C_{16}H_{24}NO_3)^+$ requires *m/z* 278.1751, found *m/z* 278.1752.

MP: 74-76 ˚C.

$[\alpha]_D^{25}$ = -103.9 (*c* 0.095, $CHCl_3$).

tert-butyl (S)-(1,3-dioxoisoindolin-2-yl)((2-methyl-4-oxocyclohex-2-en-1-yl)methyl)carbamate (2k)

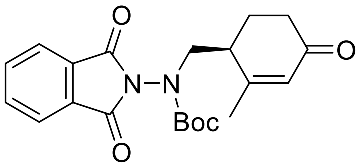

The title compound was prepared according to general procedure **E** from tert-butyl (1,3-dioxoisoindolin-2-yl)((2-methyl-4-oxocyclohex-1-en-1-yl)methyl)carbamate (**1k**) (50 mg, 0.13 mmol) and left for 48 h. The crude reaction mixture was transferred directly onto silica and purified by silica gel column chromatography (pentane/EtOAc = 4/6) to provide the title compound as a white solid (foam) in 62% yield (31.1 mg) and 93% ee. [determined by HPLC chiralpak AD, hexane/isopropanol = 90/10 to 70/30, 1 ml/min, λ = 230 nm, t(minor) = 11.10 min, t(major) = 12.97 min].

^{1}H NMR (400 MHz, Methanol-d_4) δ(ppm): 8.00 – 7.88 (m, 4H, ArH), 5.88 (s, 1H, CHC(=O)), 3.97 (m, 1H, one of CH_AH_BN, minor and major rotamer), 3.79 – 3.61 (m, 1H, one of CH_AH_BN, minor and major rotamer), 2.72 – 2.52 (m, 2H, $CHCH_2N$ and one of CH_AH_BC(=O)), 2.40 – 2.09 (m, 3H, one of CH_AH_BC(=O) and CH_2CH_2C(=O)), 1.99 (d, J = 1.5 Hz, 3H, $C{=}CCH_3$), 1.54 (s, 3.46:5.45, $C(CH_3)_3$ minor rotamer), 1.30 (s, 3.46:5.47, $C(CH_3)_3$ major rotamer).

^{13}C NMR (101 MHz, Methanol-d_4) δ(ppm): 202.0 (CHC(=O) major rotamer), 201.6 (CHC(=O) minor rotamer), 167.2 (ArC(=O) minor rotamer), 166.8 (ArC(=O) major rotamer), 165.6 ($C{=}CCH_3$ major rotamer), 165.3 ($C{=}CCH_3$ major rotamer), 155.0 (CH_2NC(=O)), 136.4 (ArCH major rotamer), 136.3 (ArCH minor rotamer), 131.2 (ArC major rotamer), 131.2 (ArC minor rotamer), 128.7 (CHC(=O) minor rotamer), 128.6 (CHC(=O) major rotamer), 124.9 (ArCH), 85.1 ($C(CH_3)_3$ minor rotamer), 83.8 ($C(CH_3)_3$ major rotamer), 51.9 (CH_2N minor rotamer), 50.5 (CH_2N major rotamer), 40.4 ($CHCH_2N$ minor rotamer), 40.2 ($CHCH_2N$ major rotamer), 34.2 (CH_2C(=O)), 28.4 ($C(CH_3)_3$ minor rotamer), 28.1 ($C(CH_3)_3$ major rotamer), 25.9 (CH_2CH_2C(=O) major rotamer), 25.8 (CH_2CH_2C(=O) minor rotamer), 23.1 ($C{=}CCH_3$ minor rotamer), 23.0 ($C{=}CCH_3$ minor rotamer).

IR (powder) ν_{max}/cm^{-1}: 2933, 2871 (C-H), 1733, 1665 (C=O).

HRMS (ES+) exact mass calculated for $[M+Na]^+$ $(C_{21}H_{24}N_2NaO_5)^+$ requires m/z 407.1577, found m/z 407.1577.

$[\alpha]_D^{25}$ = +29.5 (c 0.055, $CHCl_3$).

tert-butyl (S)-((tert-butyldimethylsilyl)oxy)((2-methyl-4-oxocyclohex-2-en-1-yl)methyl)carbamate (2l)

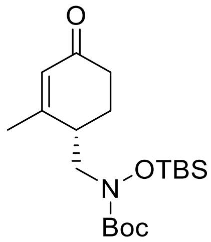

The title compound was prepared according to general procedure E from tert-butyl ((tert-butyldimethylsilyl)oxy)((2-methyl-4-oxocyclohex-1-en-1-yl)methyl)carbamate (**1l**) (48 mg, 0.13 mmol). The crude reaction mixture was transferred directly onto silica and purified by silica gel column chromatography (pentane/Et_2O = 9/1 to 8/2) to provide the title compound as a colourless oil in 53% yield (25.6 mg) and 94% ee. [determined by HPLC chiralpak AS-H, hexane/isopropanol = 90/10 to 70/30, 1 ml/min, λ = 240 nm, t(major) = 6.82 min, t(minor) = 8.09 min].

^{1}H NMR (500 MHz, Acetone-d_6) δ(ppm): 5.80 (s, 1H, $C\underline{H}C(=O)$), 3.81 (dd, *J* = 14.5, 10.0 Hz, 1H, one of $C\underline{H_A}H_BN$), 3.56 (dd, *J* = 14.5, 4.0 Hz, 1H, one of $CH_A\underline{H_B}N$), 2.94 – 2.87 (m, 1H, $C\underline{H}CH_2N$), 2.49 (ddd, *J* = 17.5, 12.5, 5.5 Hz, 1H, one of $C\underline{H_A}H_BC(=O)$), 2.23 – 2.13 (m, 1H, one of $CH_A\underline{H_B}C(=O)$), 2.02 (m, 1H, one of $C\underline{H_A}H_BCH_2C(=O)$), 2.00 (d, *J* = 1.5 Hz, 3H, $C\underline{H_3}C=C$), 1.99 – 1.93 (m, 1H, one of $CH_A\underline{H_B}CH_2C(=O)$), 1.49 (s, 9H, $OC(C\underline{H_3})_3$), 0.98 (s, 9H, $SiC(C\underline{H_3})_3$), 0.22 (2x s, 3H, $Si(C\underline{H_3})_A(CH_3)_B$), 0.22 (2x s, 3H, $Si(CH_3)_A(C\underline{H_3})_B$).

^{13}C NMR (126 MHz, Acetone-d_6) δ(ppm): 198.0 ($CH\underline{C}=O$), 162.7 ($C=\underline{C}CH_3$), 157.9 ($N\underline{C}=O$), 128.4 ($C=\underline{C}H$), 81.8 ($O\underline{C}(CH_3)_3$), 52.2 ($\underline{C}H_2N$), 38.2 ($\underline{C}HCH_2N$), 33.7 ($\underline{C}H_2C(=O)$), 28.4 ($OC(\underline{C}H_3)_3$), 26.2 ($SiC(\underline{C}H_3)_3$), 25.5 ($\underline{C}H_2CHCH_2N$), 23.1 ($C=C\underline{C}H_3$), 18.4 ($Si\underline{C}(CH_3)_3$), -4.8 (one of $Si(\underline{C}H_3)_A(CH_3)_B$), -4.8 (one of $Si(CH_3)_A(\underline{C}H_3)_B$).

HRMS (ES+) exact mass calculated for $[M+Na]^+$ $(C_{19}H_{35}NNaO_4Si)^+$ requires *m/z* 392.2228, found *m/z* 392.2227.

IR (film) ν_{max}/cm^{-1}: 2955, 2931, 2859 (C-H), 1696 (ketone C=O), 1673 (boc C=O), 1630 (C=C), 1156 (C-N).

$[\alpha]_D^{25}$ = -42.6 (*c* 0.08, $CHCl_3$).

(S)-4-((1H-indol-1-yl)methyl)-3-methylcyclohex-2-en-1-one (2m)

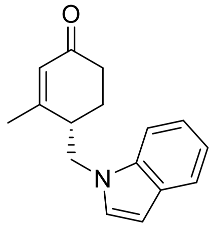

The title compound was prepared according to general procedure E from 4-((1H-indol-1-yl)methyl)-3-methylcyclohex-3-en-1-one (**1m**) (31.4 mg, 0.130 mmol). The crude reaction mixture was transferred directly onto silica and purified by silica gel column chromatography (pentane/Et_2O = 7/3) to provide the title compound as a yellow amorphous solid in 81% yield (25.3 mg) and 90% ee. [determined by HPLC chiralpak AD-H, hexane/isopropanol = 95/5, 1 ml/min, λ = 230 nm, t(major) = 8.92 min, t(minor) = 10.08 min].

^{1}H NMR (500 MHz, $CDCl_3$) δ(ppm): 7.69 (d, *J* = 8.0 Hz, 1H, ArH), 7.37 (d, *J* = 8.0 Hz, 1H, ArH), 7.27 (m, 1H, ArH), 7.19 – 7.14 (m, 1H, ArH), 7.10 (d, *J* = 3.0 Hz, 1H, ArH), 6.57 (d, *J* = 3.0 Hz, 1H, ArH), 6.00 (s, 1H, CH(C=O)), 4.49 (dd, *J* = 14.5, 5.5 Hz, 1H, one of CH_AH_BN), 4.15 (dd, *J* = 14.5, 10.0 Hz, 1H, one of CH_AH_BN), 2.96 – 2.89 (m, 1H, $CHCH_2N$), 2.50 (ddd, *J* = 17.5, 12.0, 5.0 Hz, 1H, one of $CH_AH_BC(=O)$), 2.33 (dt, *J* = 17.0, 5.0 Hz, 1H, one of $CH_AH_BC(=O)$), 2.01 (s, 3H, CH_3), 1.99 – 1.89 (m, 1H, one of $CH_AH_BCH_2C(=O)$), 1.81 – 1.72 (m, 1H, one of $CH_AH_BCH_2C(=O)$);

^{13}C NMR (126 MHz, $CDCl_3$) δ(ppm): 198.4 (C=O), 161.5 (CCH_3), 135.9 (ArC), 128.8 (ArC), 128.5 (CHC(=O)), 127.9 (ArCH), 122.0 (ArCH), 121.4 (ArCH), 119.8 (ArCH), 109.1 (ArCH), 102.1 (ArCH), 47.3 (CH_2N), 40.3 ($CHCH_2N$), 33.1 ($CH_2C(=O)$), 25.3 ($CH_2CH_2C(=O)$), 23.2 (CH_3);

HRMS (ES+) exact mass calculated for $[M+H]^+$ $(C_{16}H_{18}NO)^+$ requires *m/z* 240.1383, found *m/z* 240.1386.

IR (film) ν_{max}/cm^{-1}: 3051, 2944 (C-H), 1664 (C=O), 1626 (C=C).

$[\alpha]_D^{25}$ = -34.9 (*c* 0.16, $CHCl_3$).

tert-butyl (S)-furan-2-yl((2-methyl-4-oxocyclohex-2-en-1-yl)methyl)carbamate (2n)

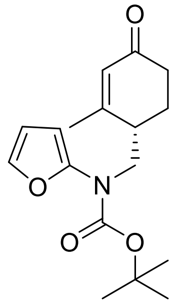

The title compound was prepared according to general procedure **E** from tert-butyl furan-2-yl((2-methyl-4-oxocyclohex-1-en-1-yl)methyl)carbamate (**1n**) (39.7 mg, 0.130 mmol). The crude reaction mixture was transferred directly onto silica and purified by silica gel column chromatography (pentane/Et_2O = 8/2) to provide the title compound as an off white solid in quantitative yield (39.7 mg) and 99% ee. [determined by HPLC chiralpak AD-H, hexane/isopropanol = 90/10, 1 ml/min, λ = 230 nm, t (major) = 5.62 min, t (minor) = 6.33 min].

^{1}H NMR (400 MHz, $CDCl_3$) δ(ppm): 7.18 (dd, *J* = 2.0, 1.0 Hz, 1H, NCCHCHC<u>H</u>O), 6.35 (dd, *J* = 3.5, 2.0 Hz, 1H, NCCHC<u>H</u>CHO), 6.03 (bs, 1H, NCC<u>H</u>CHCHO), 5.86 (s, 1H, C=C<u>H</u>C(=O)), 3.81 – 3.69 (m, 2H, C<u>H</u>$_2$N), 2.61 – 2.54 (m, 1H, C<u>H</u>CH$_2$N), 2.48 (ddd, *J* = 17.5, 11.5, 6.0 Hz, 1H, one of C<u>H</u>$_A$H$_B$C(=O)), 2.54 – 2.42 (m, 1H, one of CH$_A$<u>H</u>$_B$C(=O)), 2.08 – 1.96 (m, 2H, C<u>H</u>$_2$CH$_2$C(=O)), 1.94 (d, *J* = 1.5 Hz, 3H, C<u>H</u>$_3$C=C), 1.45 (s, 9H, C(C<u>H</u>$_3$)$_3$).

^{13}C NMR (101 MHz, $CDCl_3$) δ(ppm): 199.1 (CH$_2$<u>C</u>(=O)), 162.1 (CH$_3$<u>C</u>=C), 153.9 (N<u>C</u>(=O)), 148.3 (N<u>C</u>CHCHCHO), 138.3 (NCCHCH<u>C</u>HO), 128.2 (CH$_3$C=<u>C</u>H), 111.2 (NCCH<u>C</u>HCHO), 101.3 (NC<u>C</u>HCHCHO), 81.9 (O<u>C</u>(CH$_3$)$_3$), 48.6 (<u>C</u>H$_2$N), 39.4 (<u>C</u>HCH$_2$N), 33.3 (<u>C</u>H$_2$C(=O)), 28.3 (OC(<u>C</u>H$_3$)$_3$), 24.7 (<u>C</u>H$_2$CH$_2$C(=O)), 23.0 (<u>C</u>H$_3$C=C).

HRMS (ESI+) exact mass calculated for $[M+Na]^+$ $(C_{17}H_{23}NNaO_4)^+$ requires *m/z* 328.1519, found *m/z* 328.1519.

IR (film) ν_{max}/cm^{-1}: 2921 (C-H), 1712 (C=O ester), 1670 (C=O ketone), 1157 (C-O).

MP: 42-45 ˚C.

$[\alpha]_D^{25}$ = -39.2 (*c* 0.50, $CHCl_3$).

benzyl (S)-furan-2-yl((2-methyl-4-oxocyclohex-2-en-1-yl)methyl)carbamate (2o)

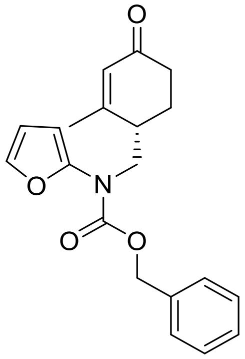

The title compound was prepared according to general procedure **E** from benzyl furan-2-yl((2-methyl-4-oxocyclohex-1-en-1-yl)methyl)carbamate (**1o**) (44.1 mg, 0.130 mmol). The crude reaction mixture was transferred directly onto silica and purified by silica gel column chromatography (pentane/Et_2O = 7/3) to provide the title compound as colourless oil in 80% yield (35.3 mg) and 97% ee. [determined by HPLC chiralpak IA, hexane/isopropanol = 90/10, 1 ml/min, λ = 230 nm, t (major) = 16.47 min, t (minor) = 20.68 min].

^{1}H NMR (400 MHz, $CDCl_3$) δ(ppm): 7.46 – 7.15 (m, 6H, ArC<u>H</u> and NCCHCHC<u>H</u>O), 6.39 (dd, *J* = 3.5, 2.0 Hz, 1H, NCCHC<u>H</u>CHO), 6.09 (bs, 1H, NCC<u>H</u>CHCHO), 5.86 (s, 1H, CH_3C=C<u>H</u>), 5.19 (s, 2H, C<u>H</u>$_2$O), 3.93 – 3.64 (m, 2H, C<u>H</u>$_2$O), 2.64 – 2.53 (m, 1H, C<u>H</u>CH_2N), 2.46 (bs, 1H, one of C<u>H</u>$_A$$H_B$C(=O)), 2.32 – 2.14 (bm, 1H, one of CH_A<u>H</u>$_B$C(=O)), 2.13 – 1.95 (bm, 2H, C<u>H</u>$_2$$CH_2$C(=O)), 1.91 (bs, 3H, C<u>H</u>$_3$).

^{13}C NMR (101 MHz, $CDCl_3$) δ(ppm): 199.0 (CH_2<u>C</u>=O), 161.7 (CH_3<u>C</u>=C), 155.1 (N<u>C</u>(=O)), 147.5 (N<u>C</u>CHCHCHO), 139.1 (NCCHCH<u>C</u>HO), 135.9 (Ar<u>C</u>), 128.6 (CH_3C=<u>C</u>H), 128.4 (Ar<u>C</u>H (may be two under this peak)), 127.6 (Ar<u>C</u>H), 111.4 (NCCH<u>C</u>HCHO), 102.7 (NC<u>C</u>HCHCHO), 68.1 (O<u>C</u>H_2), 49.4 (<u>C</u>H_2N), 39.2 (<u>C</u>HCH_2N), 33.2 (<u>C</u>H_2C(=O)), 24.6 (<u>C</u>$H_2$$CH_2$C(=O)), 22.9 (<u>C</u>$H_3$).

IR (film) ν_{max}/cm^{-1}: 2922 (C-H), 1718 (C=O ester/amide), 1669 (C=O ketone), 1281, 1167 (C-O).

HRMS (ES+) exact mass calculated for $[M+Na]^+$ $(C_{20}H_{21}NNaO_4)^+$ requires *m/z* 362.1363, found *m/z* 362.1363.

$[\alpha]_D^{25}$ = -20.8 (*c* 0.49, $CHCl_3$).

(R)-5',6'-dihydro-[1,1':2',1''-terphenyl]-4'(1'H)-one (2p)

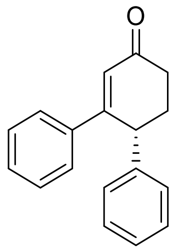

The title compound was prepared according to general procedure **E** from 5',6'-dihydro-[1,1':2',1''-terphenyl]-4'(3'H)-one (**1p**) (16.1 mg, 0.0650 mmol). The crude reaction mixture was transferred directly onto silica and purified by silica gel column chromatography (pentane/Et_2O = 9/1) to provide the title compound as a yellow solid in 76% yield (12.3 mg) and 94% ee. [determined by HPLC chiralpak OD-H, hexane/isopropanol = 90/10, 1 ml/min, λ = 230 nm, t(minor) = 9.40 min, t(major) = 13.19 min].

^{1}H NMR (500 MHz, $CDCl_3$) δ(ppm): 7.48 – 7.43 (m, 2H, Ar<u>H</u>), 7.35 – 7.21 (m, 8H, Ar<u>H</u>), 6.70 (s, 1H, C<u>H</u>C(=O)), 4.31 (dd, *J* = 5.0, 3.0 Hz, 1H, C<u>H</u>Ph), 2.57 (dddd, *J* = 13.0, 12.0, 6.0, 5.0 Hz, 1H, one of C<u>H_A</u>$H_B$$CH_2$C(=O)), 2.44 – 2.31 (m, 2H, CH_2C<u>H_2</u>C(=O)), 2.24 – 2.18 (m, 1H, one of CH_A<u>H_B</u>CH_2C(=O)).

^{13}C NMR (126 MHz, $CDCl_3$) δ(ppm): 200.1 (<u>C</u>=O), 159.6 (Ar<u>C</u>), 140.3 (Ar<u>C</u>), 138.0 (<u>C</u>=CH), 130.0 (Ar<u>C</u>H), 129.0 (Ar<u>C</u>H), 128.9 (Ar<u>C</u>H), 128.2 (Ar<u>C</u>H), 127.2 (Ar<u>C</u>H), 127.1 (Ar<u>C</u>H), 127.0 (C=<u>C</u>H), 43.3 (<u>C</u>HPh), 32.9 (<u>C</u>H_2C(=O)), 32.1 (<u>C</u>$H_2$$CH_2$C(=O)).

$[\boldsymbol{\alpha}]_{\boldsymbol{D}}^{\mathbf{25}}$ = -237.9 (*c* 0.55, $CHCl_3$).

Data is consistent with that published in the literature.[7]

(S)-3,4-diethylcyclohex-2-en-1-one (2q)

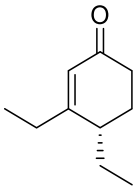

The title compound was prepared according to general procedure **F** from 3,4-diethylcyclohex-3-en-1-one (**1q**) (9.9 mg, 0.0650 mmol) using Et_2O (0.43 ml). The crude reaction mixture was concentrated under a stream of nitrogen and transferred directly onto silica then purified by silica gel column chromatography (pentane/Et_2O = 85/15) to provide the title compound as a yellow oil in 63% yield (6.2 mg) and 97% ee.* [determined by HPLC chiralpak AS-H, hexane/isopropanol = 70/30, 1 ml/min, λ = 240 nm, t(minor) = 19.23 min, t(major) = 28.23 min].

*Care should be taken when removing solvent from this compound as it appears to be slightly volatile.

^{1}H NMR (400 MHz, $CDCl_3$) δ(ppm): 5.83 (s, 1H, C=C<u>H</u>), 2.45 (ddd, *J* = 17.0, 11.5, 5.5 Hz, 1H, one of C$\underline{H_A}$$H_B$C(=O)), 2.38 – 2.14 (m, 4H, one of $CH_A$$\underline{H_B}$C(=O), C<u>H</u>$CH_2$ and C$\underline{H_2}$C=C), 2.09 – 1.90 (m, 2H, C$\underline{H_2}$$CH_2$C(=O)), 1.67 (dddd, *J* = 14.0, 11.5, 7.5, 3.5 Hz, 1H, one of CHC$\underline{H_A}$$CH_B$$CH_3$), 1.54 – 1.39 (m, 1H, one of CHCH_AC$\underline{H_B}$$CH_3$), 1.10 (t, *J* = 7.5 Hz, 3H, C$\underline{H_3}$$CH_2$C=C), 1.01 (t, *J* = 7.5 Hz, 3H, C$\underline{H_3}$$CH_2$CH).

^{13}C NMR (126 MHz, $CDCl_3$) δ(ppm): 200.0 (<u>C</u>=O), 171.7 (<u>C</u>=CH), 124.3 (C=<u>C</u>H), 40.1 (<u>C</u>HC=C), 33.8 (<u>C</u>H_2C(=O)), 28.8 (<u>C</u>H_2C=C), 25.7 (<u>C</u>$H_2$$CH_2$C(=O)), 24.1 ($CH_3$<u>C</u>$H_2$CH), 12.7 (<u>C</u>$H_3$$CH_2$CH), 11.7 (<u>C</u>$H_3$$CH_2$C=C).

IR (film) ν_{max}/cm^{-1}: 2965, 2931, 2876 (C-H), 1671 (C=O).

HRMS (ES+) exact mass calculated for $[M+H]^+$ $(C_{10}H_{17}O)^+$ requires *m/z* 153.1274, found *m/z* 153.1273.

$[\alpha]_D^{25}$ = -78.4 (*c* 0.54, $CHCl_3$).

(S)-3,4-dipropylcyclohex-2-en-1-one (2r)

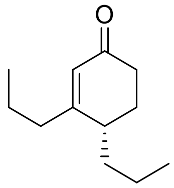

The title compound was prepared according to general procedure **F** from 3,4-dipropylcyclohex-3-en-1-one (**1r**) (11.7 mg, 0.0650 mmol). The crude reaction mixture was transferred directly onto silica and purified by silica gel column chromatography (pentane/Et_2O = 95/5) to provide the title compound as a yellow oil in 50% yield (5.8 mg) and 97% ee. [determined by HPLC chiralpak AS-H, hexane/isopropanol = 90/10 to 70/30, 1 ml/min, λ = 240 nm, t(minor) = 20.13 min, t(major) = 31.25 min].

^{1}H NMR (500 MHz, $CDCl_3$) δ(ppm): 5.81 (s, 1H, C=C<u>H</u>), 2.45 (ddd, *J* = 17.5, 12.0, 5.0 Hz, 1H, one of C$\underline{H_A}$$H_B$C(=O)), 2.32 – 2.12 (m, 4H, one of C$H_A$$\underline{H_B}$C(=O), C<u>H</u>$CH_2$ and C$\underline{H_2}$C=C), 2.06 – 1.97 (m, 1H, one of C$\underline{H_A}$$H_B$$CH_2$C(=O)), 1.96 – 1.89 (m, 1H, one of C$H_A$$\underline{H_B}$$CH_2$C(=O)), 1.65 – 1.21 (m, 8H, 2 x C$\underline{H_2}$$CH_3$ and 2 x $CH_3$$CH_2C\underline{H_2}$CH), 0.98 – 0.91 (m, 6H, 2 x C$\underline{H_3}$).

^{13}C NMR (126 MHz, $CDCl_3$) δ(ppm): 200.0 (C=O), 170.5 (<u>C</u>=CH), 125.3 (C=<u>C</u>H), 38.0 (<u>C</u>HCH_2 and <u>C</u>H_2C=C), 33.6 (<u>C</u>H_2C(=O)), 33.1 ($CH_3$$CH_2$<u>C</u>$H_2$CH), 26.1 (<u>C</u>$H_2$$CH_2$C(=O)), 21.3 ($CH_3$<u>C</u>$H_2$$CH_2$CH), 20.6 ($CH_3$<u>C</u>$H_2$$CH_2$C=C), 14.2 (one of <u>C</u>H_3), 14.0 (one of <u>C</u>H_3).

IR (film) ν_{max}/cm^{-1}: 2957, 2930, 2872 (C-H), 1673 (C=O), 1622 (C=C).

HRMS (ES+) exact mass calculated for $[M+H]^+$ $(C_{12}H_{21}O)^+$ requires *m/z* 181.1587, found *m/z* 181.1587.

$[\alpha]_D^{25}$ = -81.9 (*c* 0.47, $CHCl_3$).

(S)-6-methyl-5,6-dihydro-[1,1'-biphenyl]-3(4H)-one (2s)

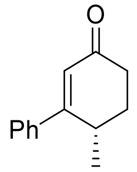

The title compound was prepared according to general procedure **F** from 6-methyl-4,5-dihydro-[1,1'-biphenyl]-3(2H)-one (**1s**) (24.2 mg, 0.130 mmol). The crude reaction mixture was transferred directly onto silica and purified by silica gel column chromatography (pentane/Et_2O = 9/1) to provide the title compound as an amorphous yellow solid in 63% yield (15.2 mg) and 95% ee. [determined by HPLC chiralpak OD-H, hexane/isopropanol = 85/15, 1 ml/min, λ = 280 nm, t(minor) = 8.50 min, t(major) = 9.71 min].

^{1}H NMR (500 MHz, $CDCl_3$) δ(ppm): 7.53 – 7.46 (m, 2H, Ar$\underline{H}$), 7.41 (m, 3H, Ar$\underline{H}$), 6.25 (s, 1H, C$\underline{H}$C(=O)), 3.16 (ddq, J = 11.0, 7.5, 4.0, 3.5 Hz, 1H, C$\underline{H}$$CH_3$), 2.60 (ddd, *J* = 17.5, 13.0, 5.0 Hz, 1H, one of C$\underline{H_A}$$H_B$C(=O)), 2.44 (dt, *J* = 17.0, 4.5 Hz, 1H, one of $CH_A$$\underline{H_B}$C(=O)), 2.37 – 2.24 (m, 1H, one of C$\underline{H_A}$$H_B$$CH_2$C(=O)), 1.98 (dtd, *J* = 13.5, 5.0, 3.5 Hz, 1H, one of $CH_A$$\underline{H_B}$$CH_2$C(=O)), 1.18 (d, *J* = 7.0 Hz, 3H, C$\underline{H_3}$).

^{13}C NMR (126 MHz, $CDCl_3$) δ(ppm): 199.9 ($\underline{C}$=O), 165.4 ($\underline{C}$=CC(=O)), 138.4 (Ar$\underline{C}$), 129.9 (Ar$\underline{C}$H), 128.9 (Ar$\underline{C}$H), 126.8 (Ar$\underline{C}$H), 125.2 (C=$\underline{C}$C(=O)), 33.3 ($\underline{C}H_2$C(=O)), 31.3 ($\underline{C}$HCH_3), 29.7 ($\underline{C}H_2CH_2$C(=O)), 18.5 ($\underline{C}H_3$).

IR (film) ν_{max}/cm^{-1}: 2963, 2929, 2869 (C-H), 1665 (C=O), 1601 (C=C).

HRMS (ES+) exact mass calculated for $[M+H]^+$ $(C_{13}H_{15}O)^+$ requires *m/z* 187.1117, found *m/z* 187.1118.

Data is consistent with that published in the literature.[11]

$[\alpha]_D^{25}$ = -287.4 (*c* 0.85, $CHCl_3$).

(S)-4'-methoxy-6-methyl-5,6-dihydro-[1,1'-biphenyl]-3(4H)-one (2t)

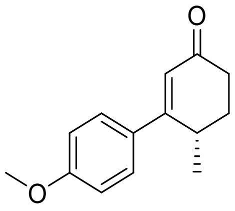

The title compound was prepared according to general procedure **F** from 4'-methoxy-6-methyl-4,5-dihydro-[1,1'-biphenyl]-3(2H)-one (**1t**) (28.1 mg, 0.130 mmol). The crude reaction mixture was transferred directly onto silica and purified by silica gel column chromatography (pentane/Et_2O = 7/3) to provide the title compound as an amorphous yellow solid in 52% yield (14.6 mg) and 93% ee. [determined by HPLC chiralpak AD-H, hexane/isopropanol = 90/10 to 70/30, 1 ml/min, λ = 220 nm, t(major) = 10.00 min, t(minor) = 11.67 min].

^{1}H NMR (500 MHz, $CDCl_3$) δ(ppm): 7.56 – 7.41 (m, 2H, ArC$\underline{H}$), 6.99 – 6.88 (m, 2H, ArC$\underline{H}$), 6.25 (s, 1H, C$\underline{H}$C(=O)), 3.84 (s, 3H, OC$\underline{H_3}$), 3.19 – 3.10 (m, 1H, C$\underline{H}$CH$_3$), 2.58 (ddd, *J* = 17.5, 13.5, 5.0 Hz, 1H, one of C$\underline{H_A}$H$_B$C(=O)), 2.46 – 2.38 (m, 1H, one of CH$_A$$\underline{H_B}$C(=O)), 2.34 – 2.22 (m, 1H, one of C$\underline{H_A}$CH$_B$CH$_2$C(=O)), 2.01 – 1.93 (m, 1H, one of CH$_A$C$\underline{H_B}$CH$_2$C(=O)), 1.20 (d, *J* = 7.0 Hz, 3H, CHC$\underline{H_3}$).

^{13}C NMR (126 MHz, $CDCl_3$) δ(ppm): 199.8 ($\underline{C}$=O), 164.6 (Ar$\underline{C}$), 161.3 (Ar$\underline{C}$), 130.2 ($\underline{C}$=CC(=O)), 128.3 (Ar$\underline{C}$H), 123.4 ($\underline{C}$HC(=O)), 114.3 (Ar$\underline{C}$H), 55.5 (O$\underline{C}$H$_3$), 32.9 ($\underline{C}$H$_2$C(=O)), 30.8 ($\underline{C}$HCH$_3$), 29.5 ($\underline{C}$H$_2$CH$_2$C(=O)), 18.4 (CH$\underline{C}$H$_3$).

IR (film) ν_{max}/cm^{-1}: 2932, 2839 (C-H), 1659 (C=O), 1593 (C=C), 1251 (C-O).

HRMS (ES+) exact mass calculated for $[M+H]^+$ $(C_{14}H_{17}O_2)^+$ requires *m/z* 217.1223, found *m/z* 217.1223.

Data is consistent with that published in the literature.[12]

$[\boldsymbol{\alpha}]_D^{25}$ = -66.0 (*c* 0.685, $CHCl_3$).

1.5 Derivitisation of enantioenriched products

(S)-(2-methyl-4-oxocyclohex-2-en-1-yl)methyl 4-methylbenzenesulfonate (4a)

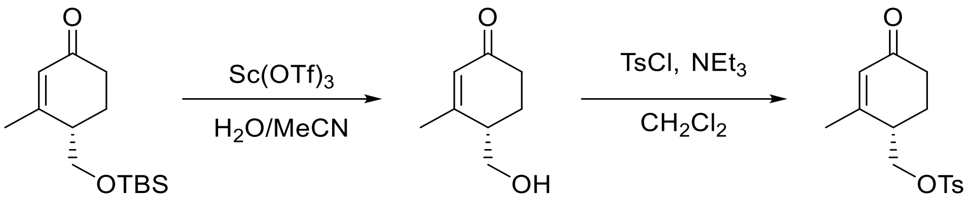

(**2f**) (**2g**) (**4a**)

i. According to a literature procedure,[13] to a stirred solution of (S)-4-(hydroxymethyl)-3-methylcyclohex-2-en-1-one (**2f**) (479 mg, 1.88 mmol, 1.00 eq) in MeCN (9.5 ml) was added H_2O (170 µl, 5.00 eq, 9.4 mmol) followed by $Sc(OTf)_3$ (4.60 mg, 0.009 mmol, 0.005 eq). The reaction was stirred overnight at which point pH 7 phosphate buffer was added (15 ml). The aqueous phase was extracted with CH_2Cl_2 (3 x 20 ml). The combined organic phases were washed with brine, dried over Na_2SO_4, filtered and concentrated *in vacuo*. The crude product was purified by silica gel column chromatography (EtOAc) to afford **2g** as a yellow oil in 58% yield (154 mg) and 99% ee.

ii. To a stirred solution of **2g** (98.0 mg, 0.700 mmol, 1.00 eq) in CH_2Cl_2 (2.3 ml) at 0 °C was added NEt_3 (117 µl, 0.840 mmol, 1.20 eq) followed by tosyl chloride (147 mg, 0.770 mmol, 1.10 eq). The reaction was stirred overnight at which point sat. aq. NH_4Cl (10 ml) was added. The aqueous layer was extracted with EtOAc (3 x 20 ml). The combined organic phases were washed with brine, dried over Na_2SO_4, filtered, and concentrated *in vacuo*. The crude reaction mixture was purified by silica gel column chromatography (pentane/Et_2O = 50/50 to 0/100) to afford the title compound as a colourless oil in 76% yield (157 mg) and 99% ee. [determined by HPLC chiralpak AD-H, hexane/isopropanol = 90/10 to 70/30, 1 ml/min, λ = 240 nm, t(major) = 18.90 min, t(minor) = 19.89 min].*

*The title compound should be stored in a freezer to avoid decomposition which occurs over a period of hours at ambient temperature.

^{1}H NMR (500 MHz, Acetone-d_6) δ(ppm): 7.89 – 7.80 (m, 2H, Ar$\underline{H}$), 7.55 – 7.45 (m, 2H, Ar$\underline{H}$), 5.81 – 5.78 (m, 1H, C$\underline{H}$C(=O)), 4.31 (dd, *J* = 10.0, 6.5 Hz, 1H, one of C$\underline{H_A}H_B$O), 4.23 (dd, *J* = 10.0, 4.0 Hz, 1H, one of C$H_A\underline{H_B}$O), 2.78 – 2.71 (m, 1H, C$\underline{H}CH_2$O), 2.45 (s, 3H, ArC$\underline{H_3}$), 2.29 (ddd, *J* = 17.0, 9.0, 5.0 Hz, 1H, one of $CH_2C\underline{H_A}H_B$C(=O)), 2.20 (ddd, *J* = 17.0, 8.0, 5.0 Hz, 1H, one of $CH_2CH_A\underline{H_B}$C(=O)), 2.00 – 1.93 (m, 2H, C$\underline{H_2}CH_2$C(=O) and solvent peak), 1.91 (dd, *J* = 1.5, 1.0 Hz, 3H, C$\underline{H_3}$).

^{13}C NMR (126 MHz, Acetone-d_6) δ(ppm): 197.8 (C=O), 159.8 (CH_3C=CHC(=O)), 146.2 (ArC), 133.9 (ArC), 131.0 (ArCH), 129.5 (CH_3C=CHC(=O)), 128.8 (ArCH), 70.9 (CH_2O), 39.9 (CHCH_2O), 34.9 (CH_2CH_2C(=O)), 25.8 (CH_2CH_2C(=O)), 22.4 (CH_3C=CHC(=O)), 21.5 (ArCH_3).

IR (film) ν_{max}/cm^{-1}: 2955 (C-H), 1667 (C=O), 1121 (C-O).

HRMS (ES+) exact mass calculated for $[M+H]^+$ $(C_{15}H_{19}O_4S)^+$ requires *m/z* 295.0999, found *m/z* 295.1000.

$[\alpha]_D^{25}$ = -91.4 (*c* 0.05, $CHCl_3$).

(S)-4-(azidomethyl)-3-methylcyclohex-2-en-1-one (4b)

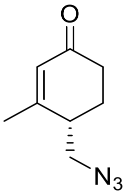

Sodium azide (43.5 mg, 0.680 mmol, 5.00 eq) was added to a stirred solution of (S)-(2-methyl-4-oxocyclohex-2-en-1-yl)methyl 4-methylbenzenesulfonate (**4a**) (40.0 mg, 0.136 mmol, 1.00 eq) in DMF (0.68 ml). The reaction was warmed to 45 °C and stirred for 1 hour at which point the reaction was deemed complete by TLC analysis (pentane/Et_2O = 1/1). H_2O (10 ml) was added and the aqueous layer extracted with EtOAc (3 x 10 ml). The combined organic phases were washed with brine, dried over Na_2SO_4, filtered, and concentrated *in vacuo*. The crude product was purified by silica gel column chromatography (pentane/Et_2O = 1/1) to afford the title compound as a colourless oil in 51% yield (11.4 mg) and 99% ee. [determined by HPLC chiralpak AS-H, hexane/isopropanol = 70/30, 1 ml/min, λ = 230 nm, t(minor) = 19.37 min, t(major) = 25.50 min].

^{1}H NMR (500 MHz, $CDCl_3$) δ(ppm): 5.94 (s, 1H, C$\underline{H}$C(=O)), 3.57 (dd, *J* = 12.5, 4.5 Hz, 1H, one of C$\underline{H_A}$$H_B$$N_3$), 3.49 (dd, *J* = 12.5, 8.0 Hz, 1H, one of $CH_A$$\underline{H_B}$$N_3$), 2.55 – 2.43 (m, 2H, C$\underline{H}$$CH_2N_3$ and one of C$\underline{H_A}$$H_B$C(=O)), 2.35 (ddd, *J* = 17.5, 7.5, 5.0 Hz, 1H, one of $CH_A$$\underline{H_B}$C(=O)), 2.17 – 2.02 (m, 2H, C$\underline{H_2}$$CH_2$C(=O)), 2.00 (s, 3H, C$\underline{H_3}$).

^{13}C NMR (126 MHz, $CDCl_3$) δ(ppm): 198.6 ($\underline{C}$=O), 160.6 ($CH_3$$\underline{C}$=C), 129.1 ($CH_3$C=$\underline{C}$), 52.7 ($\underline{C}$$H_2N_3$), 39.8 ($\underline{C}HCH_2N_3$), 34.2 ($\underline{C}$$H_2$C(=O)), 25.9 ($\underline{C}$$H_2$$CH_2$C(=O)), 22.9 ($\underline{C}$$H_3$).

HRMS (ES+) exact mass calculated for $[M+Na]^+$ $(C_8H_{11}N_3NaO)^+$ requires *m/z* 188.0794, found *m/z* 188.0796.

IR (film) ν_{max}/cm^{-1}: 2980, 2884 (C-H), 2095 (N=N=N), 1665 (C=O), 1626 (C=C).

$[\alpha]_D^{25}$ = -88.5 (*c* 0.225, $CHCl_3$).

(S)-3-methyl-4-((phenylthio)methyl)cyclohex-2-en-1-one (4c)

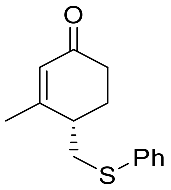

To a solution of 60% NaH (6.00 mg, 0.150 mmol, 1.10 eq) in DMF (0.7 ml) at 0 °C was added thiophenol (16.4 µl, 0.16 mmol, 1.20 eq). The suspension was stirred for 30 min at which point (S)-(2-methyl-4-oxocyclohex-2-en-1-yl)methyl 4-methylbenzenesulfonate (**4a**) (40.0 mg, 0.140 mmol, 1.00 eq) in DMF (0.7 ml) was added at 0 °C. The reaction was deemed complete by TLC analysis at 1 h (pentane/Et_2O = 1/1) at which point sat. aq. NH_4Cl (10 ml) was added. The aqueous phase was extracted with Et_2O (3 x 10 ml). The combined organic phases were washed with brine, dried over Na_2SO_4, filtered, and concentrated *in vacuo*. The crude product was purified by silica gel column chromatography (pentane/Et_2O = 8/2 to 7/3) to afford the title compound as a yellow oil in 75% yield (24.4 mg) and 97% ee. [determined by HPLC chiralpak AS-H, hexane/isopropanol = 70/30, 1 ml/min, λ = 230 nm, t(minor) = 28.27 min, t(major) = 38.01 min].

^{1}H NMR (400 MHz, $CDCl_3$) δ(ppm): 7.41 – 7.34 (m, 2H, ArH), 7.34 – 7.27 (m, 2H, ArH), 7.25 – 7.18 (m, 1H, ArH), 5.87 (t, *J* = 1.5 Hz, 1H, C=CH), 3.24 (dd, *J* = 13.0, 3.5 Hz, 1H, one of CH_AH_BS), 2.94 (dd, *J* = 13.0, 10.0 Hz, 1H, one of CH_AH_BS), 2.53 – 2.45 (m, 1H, $CHCH_2S$), 2.45 – 2.37 (m, 1H, one of CH_AH_BC(=O)), 2.35 – 2.17 (m, 2H, one of CH_AH_BC(=O) and one of $CH_AH_BCH_2C$(=O)), 2.17 – 2.05 (m, 1H, one of $CH_AH_BCH_2C$(=O)), 1.96 (dd, *J* = 1.5, 0.5 Hz, 3H, CH_3).

^{13}C NMR (101 MHz, $CDCl_3$) δ(ppm): 198.8 (C=O), 162.7 (CH_3C), 135.9 (ArC), 130.1 (ArCH), 129.2 (ArCH), 127.9 (CHC(=O)), 126.8 (ArCH), 39.3 ($CHCH_2S$), 35.8 (CH_2S), 33.6 (CH_2C(=O)), 26.1 (CH_2CH_2C(=O)), 22.9 (CH_3).

IR (film) ν_{max}/cm^{-1}: 2980, 2930, 2890 (C-H), 1665 (C=O), 1626 (C=C).

HRMS (ES+) exact mass calculated for $[M+H]^+$ $(C_{14}H_{17}OS)^+$ requires *m/z* 233.0995, found *m/z* 233.0996.

$[\alpha]_D^{25}$ = -8.76 (*c* 0.25, $CHCl_3$).

(S)-S-methyl O-((2-methyl-4-oxocyclohex-2-en-1-yl)methyl) carbonodithioate (4d)

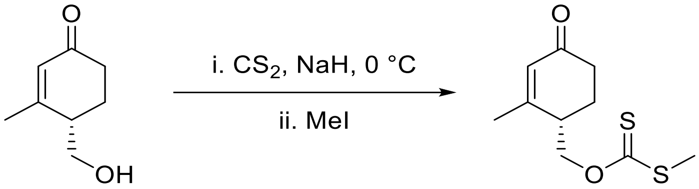

According to a modified literature procedure,[14] to a stirred solution of **2g** (50.0 mg, 0.357 mmol, 1.00 eq) in THF (0.7 ml) at 0 °C was added CS_2 (43.0 µl, 0.714 mmol, 2.00 eq). The solution was stirred for 30 min at which point 60% NaH (14.3 mg, 1.43 mmol, 4.00 eq) was added. The reaction was stirred for a further hour at which point methyl iodide (88.9 µl, 1.43 mmol, 4.00 eq) was added. The reaction was stirred until completion was observed by TLC analysis (Et_2O) at which point H_2O (10 ml) was added. The aqueous phase was extracted with Et_2O (3 x 10 ml). The combined organic phases were washed with brine, dried over Na_2SO_4, filtered, and concentrated *in vacuo*. The crude product was purified by silica gel column chromatography (pentane/Et_2O = 7/3 to 6/4) to provide the title compound as a yellow oil in 71% (58.4 mg) and 99% ee. [determined by HPLC chiralpak AS-H, hexane/isopropanol = 70/30, 1 ml/min, λ = 280 nm, t(major) = 25.27 min, t(minor) = 27.73 min].

^{1}H NMR (500 MHz, Benzene-d_6) δ(ppm): 5.86 – 5.71 (m, 1H, C=C<u>H</u>), 4.31 (dd, *J* =11.0, 4.5 Hz, 1H C$\underline{H_A}H_B$O), 4.27 (dd, *J* =11.0, 7.0 Hz, 1H C$H_A\underline{H_B}$O), 2.16 (ddd, *J* = 17.0, 9.5, 5.0 Hz, 1H, one of C$\underline{H_A}H_B$C(=O)), 2.09 (s, 3H, S$C\underline{H_3}$), 1.97 – 1.94 (m, 2H, one of CH_AH_BC(=O) and C<u>H</u>CH_2O), 1.53 – 1.40 (m, 2H, C$\underline{H_2}CH_2$C(=O)), 1.32 (t, *J* = 1.0 Hz, 3H, C$C\underline{H_3}$).

^{13}C NMR (126 MHz, Benzene-d_6) δ(ppm): 216.2 (<u>C</u>=S), 196.3 (<u>C</u>=O), 157.7 (C=<u>C</u>CH_3), 129.5 (<u>C</u>=CCH_3), 73.3 (<u>C</u>H_2O), 38.9 (<u>C</u>HCH_2O), 34.6 (<u>C</u>H_2C(=O)), 25.6 (<u>C</u>H_2CH_2C(=O)), 22.1 (C<u>C</u>H_3), 18.9 (S<u>C</u>H_3).

IR (film) ν_{max}/cm^{-1}: 2950 (C-H), 1670 (C=O) 1627 (C=C).

HRMS (ES+) exact mass calculated for $[M+Na]^+$ $(C_{10}H_{14}NaO_2S_2)^+$ requires *m/z* 253.0327, found *m/z* 253.0328.

$[\alpha]_D^{25}$ = -119.1 (*c* 0.23, $CHCl_3$).

(3aR,7aS)-3a-methyl-3-thioxohexahydroisobenzofuran-5(3H)-one (4e)

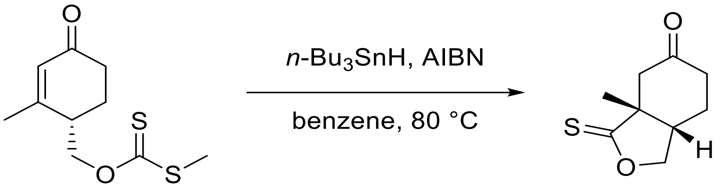

According to a modified literature procedure,[14] a solution of (S)-S-methyl O-((2-methyl-4-oxocyclohex-2-en-1-yl)methyl) carbonodithioate (**4d**) (20.0 mg, 87.0 µmol, 1.00 eq), *n*-Bu_3SnH (35.0 µl, 0.13 mmol, 1.15 eq) and AIBN (1.40 mg, 8.70 µmol, 0.100 eq) were heated to 80 °C in degassed benzene (2.9 ml) under an atmosphere of argon in a schlenk tube. The reaction mixture was stirred overnight and then allowed to cool to room temperature at which point the reaction mixture was transferred directly onto a column of silica. The compound was purified by silica gel column chromatography (pentane/EtOAc = 9/1 to 8/2 to 4/6) and obtained as a white solid in 39% yield (6.2 mg) and 97% ee. [determined by HPLC chiralpak AS-H, hexane/isopropanol = 70/30, 1 ml/min, λ = 254 nm, t(minor) = 18.18 min, t(major) = 22.75 min].*

The resulting solid could be crystallised by slow evaporation of Et_2O. The crystal obtained was >99% ee.

*The presence of oxygen in this reaction will result in the formation of the lactone which is inseparable from the thionolactone by silica gel column chromatography.

^{1}H NMR (500 MHz, Benzene-*d*$_6$) δ(ppm): 3.71 (dd, *J* = 9.5, 7.0 Hz, 1H, one of C$\underline{H_A}H_B$O), 3.40 (dd, *J* = 9.5, 4.5 Hz, 1H, one of C$H_A\underline{H_B}$O), 2.58 (d, *J* = 15.5 Hz, 1H, one of C$\underline{H_A}H_B$CCH_3), 2.05 (dd, *J* = 15.5, 1.0 Hz, 1H, one of C$H_A\underline{H_B}$CCH_3), 1.75 – 1.53 (m, 2H, $CH_2C\underline{H_2}$C(=O)), 1.30 – 1.21 (m, 1H, C$\underline{H}$$CH_2$CO), 1.01 – 0.93 (m, 1H, one of C$\underline{H_A}H_B$$CH_2$C(=O)), 0.90 – 0.86 (m, 1H, one of C$H_A\underline{H_B}$$CH_2$C(=O)), 0.84 (s, 3H, C$\underline{H_3}$).

^{13}C NMR (126 MHz, Benzene-*d*$_6$) δ(ppm): 227.4 ($\underline{C}$=S), 205.4 ($\underline{C}$=O), 76.8 ($\underline{C}$-O), 56.4 ($\underline{C}CH_3$), 48.4 ($\underline{C}H_2CCH_3$), 41.2 ($\underline{C}HCH_2$CO), 36.6 ($CH_2\underline{C}H_2$C(=O)), 26.9 ($\underline{C}H_3$), 24.7 ($\underline{C}H_2CH_2$C(=O)).

IR (film) ν_{max}/cm^{-1}: 2948, 2911, 2857 (C-H), 1714 (C=O) 1228 (C=S).

MP: 92-94 °C.

$[\alpha]_D^{25}$ = -51.3 (*c* 0.065, $CHCl_3$).

tert-Butyl (*2aS,2a1S*)-6-hydroxy-2a1-methyl-5-oxo-2a,2a1,3,4,5,7-hexahydrobenzo[cd]indole-1(2H)-carboxylate (4f)

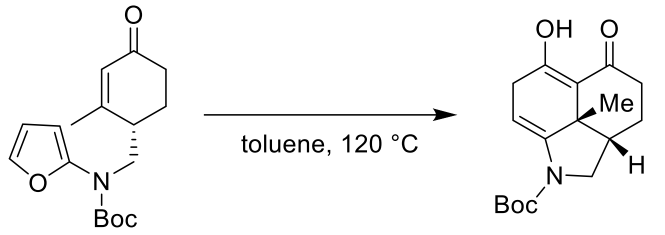

A solution of tert-butyl (S)-furan-2-yl((2-methyl-4-oxocyclohex-2-en-1-yl)methyl)carbamate (**2n**) (7.2 mg, 0.024 mmol) in distilled and degassed toluene (0.72 mL) was heated to 120 °C for 72 h. The reaction was allowed to cool to room temperature at which point the crude mixture was flushed through a plug of silica (pentane/Et_2O = 1/1) to provide the product as a red-orange oil in 96% yield (6.9 mg) and 98% ee. [determined by HPLC chiralpak AS, hexane/isopropanol = 99/1, 1 ml/min, λ = 240 nm, t(major) = 15.64 t(minor) = 22.00].*

*Trace impurities from the prototropic shift visible by staining with ninhydrin appear to inhibit this reaction.

^{1}H NMR (500 MHz, Methanol-d_4) δ(ppm): 5.76 (bs, 1H, C=C<u>H</u>), 4.07 (dd, *J* = 11.5, 8.0 Hz, 1H, one of C<u>H_A</u>H_BN), 3.55 – 3.45 (dd, *J* = 11.0 Hz, 1H, one of CH_AH_BN (minor tautomer peak underneath)), 3.24 (dd, *J* = 21.0, 2.0 Hz, 1H, one of $C\underline{H_A}H_BC$=CN), 2.90 (dd, *J* = 21.0, 6.5 Hz, 1H, one of $CH_A\underline{H_B}C$=CN), 2.38 (ddd, *J* = 17.0, 13.0, 4.0 Hz, 1H, one of $CH_2C\underline{H_A}H_BC$(=O)), 2.32 – 2.22 (m, 1H, one of $CH_2CH_A\underline{H_B}C$(=O)), 2.15 (dddd, *J* = 13.0, 8.0, 4.5, 2.0 Hz, 1H, $C\underline{H}CH_2N$), 1.90 – 1.82 (m, 1H, one of $C\underline{H_A}H_BCH_2C$(=O)), 1.51 (d, *J* = 3.5 Hz, 9H, $C(C\underline{H_3})_3$), 1.41 – 1.29 (m, 1H, one of $CH_A\underline{H_B}CH_2C$(=O) (minor tautomer peak underneath)), 1.25 (s, 3H, $CHCC\underline{H_3}$).

^{13}C NMR (126 MHz, Methanol-d_4) δ(ppm): 196.4 ($CH_2\underline{C}$(=O)), 184.5 ($\underline{C}$OH), 153.8 (N$\underline{C}$(=O)), 148.4 (N$\underline{C}$=CH), 113.1 ($\underline{C}$C(=O)), 96.6 (NC=$\underline{C}$H), 82.0 ($\underline{C}(CH_3)_3$), 54.7 ($\underline{C}H_2N$), 46.0 ($CH\underline{C}CH_3$), 43.0 ($CH_3CC\underline{H}$), 36.0 (C=C$\underline{C}H_2$), 32.8 ($C(\underline{C}H_3)_3$), 28.6 ($\underline{C}H_2CH_2C$(=O)), 28.5 ($\underline{C}H_2CH_2C$(=O)), 27.8 ($CHC\underline{C}H_3$).

IR (film) ν_{max}/cm^{-1}: 2974 (C-H), 1709 (C=O), 1392, 1370, 1146 (C-O).

HRMS (ES+) exact mass calculated for $[M+H]^+$ $(C_{17}H_{23}NO_4)^+$ requires *m/z* 306.1700, found *m/z* 306.1700.

$[\alpha]_D^{25}$ = +84.8 (*c* 0.55, $CHCl_3$).

1.6 Synthesis of catalysts

(S)-2-amino-3-(naphthalen-1-yl)propan-1-ol (S16)

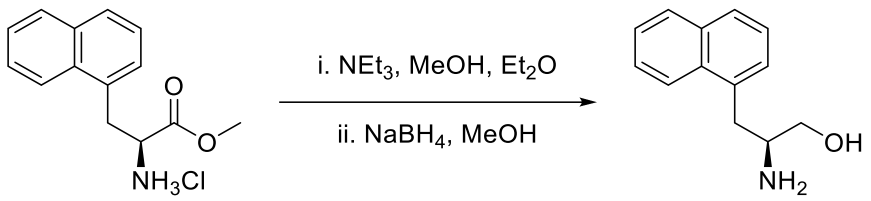

i. According to a literature procedure,[15] methyl (S)-2-amino-3-(naphthalen-1-yl)propanoate hydrochloride (2.00 g, 6.34 mmol, 1.00 eq) was stirred in MeOH as a slurry. NEt_3 (1 ml) was added followed by Et_2O (35 ml). The mixture was stirred at -10 °C for 1 h at which point the mixture was filtered and washed with Et_2O (30 ml). The filtrate was concentrated *in vacuo* and then placed under an Ar atmosphere. MeOH (18 ml) was added and the solution was cooled to 0 °C. To the stirred solution was added $NaBH_4$ (0.600 g, 15.9 mmol, 2.50 eq) and the reaction mixture was allowed to warm to room temperature overnight. H_2O (30 ml) was added and the aqueous layer was extracted with EtOAc (3 x 30 ml). The combined organic phases were washed with brine, dried over $MgSO_4$, filtered, and concentrated *in vacuo*. The title compound (**S16**) was taken on without further purification.

$[\alpha]_D^{25}$ = -47 (*c* 0.21, $CHCl_3$).

tert-butyl (S)-(1-hydroxy-3-(naphthalen-1-yl)propan-2-yl)carbamate (S17)

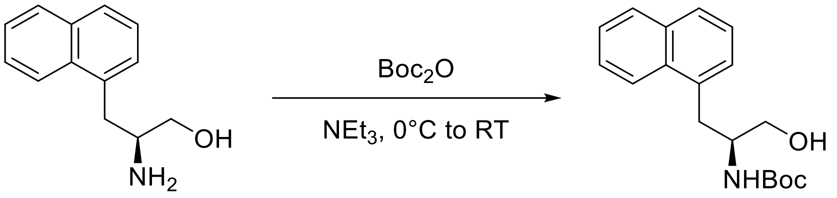

ii. To a stirred solution of (S)-2-amino-3-(naphthalen-1-yl)propan-1-ol (1.13 g, 5.61 mmol, 1.00 eq) in a 4/1 mixture of THF/H_2O (14 ml) at 0 °C was added Na_2CO_3 (1.33 g, 12.6 mmol, 2.24 eq) and Boc_2O (1.35 g, 6.18 mmol, 1.10 eq). The reaction was allowed to warm to room temperature overnight at which point H_2O was added (30 ml) and the solution brought to pH 2 by the addition of 0.1 M HCl. The aqueous layer was extracted with CH_2Cl_2 (3 x 30 ml). The combined organic phases were washed with brine, dried over $MgSO_4$, filtered, and concentrated *in vacuo*. The title compound (**S17**), a white solid, was taken on without further purification.

^{1}H NMR (400 MHz, $CDCl_3$) δ(ppm): 8.19 (d, *J* = 8.5 Hz, 1H, ArH), 7.94 – 7.82 (m, 1H, ArH), 7.75 (dt, *J* = 8.0, 1.0 Hz, 1H¸ ArH), 7.59 – 7.51 (m, 1H, ArH), 7.51 – 7.44 (m, 1H, ArH), 7.44 – 7.32 (m, 2H¸ ArH), 4.94 (bs, 1H, NH), 4.09 – 3.98 (m, 1H, CHNH), 3.76 – 3.49 (bm, 2H, CH_2OH), 3.46 – 3.17 (bm, 2H, CH_2Ar), 2.50 (bs, 1H, OH), 1.42 (bs, 9H).

^{13}C NMR (101 MHz, $CDCl_3$) δ(ppm): 156.3 (C=O), 134.32 (ArC), 134.1 (ArC), 132.3 (ArC), 128.9 (ArCH), 127.7 (ArCH), 127.5 (ArCH), 126.4 (ArCH), 125.8 (ArCH), 125.6 (ArCH), 124.0 (ArCH), 79.8 ($OC(CH_3)_3$), 64.3 (CH_2OH), 53.2 (CHNH), 34.8 (CH_2Ar), 28.5 ($C(CH_3)_3$).

(S)-2-((tert-butoxycarbonyl)amino)-3-(naphthalen-1-yl)propyl 4-methylbenzenesulfonate (S18)

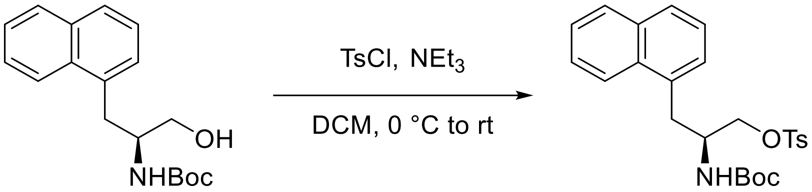

iii. To a solution of tert-butyl (S)-(1-hydroxy-3-(naphthalen-1-yl)propan-2-yl)carbamate (1.28 g, 6.35 mmol, 1.00 eq) in CH_2Cl_2 (21 ml) at 0 °C was added NEt_3 (1.06 ml, 7.62 mmol, 1.20 eq) followed by tosyl chloride (1.33 g, 6.99 mmol, 1.10 eq). The reaction was allowed to warm to room temperature overnight until completion was observed by TLC analysis at which point H_2O (30 ml) was added. The aqueous phase was extracted with EtOAc (3 x 30 ml), washed with brine, dried over $MgSO_4$, filtered, and concentrated *in vacuo*. The crude product was purified by silica gel column chromatography (pentane/EtOAc = 85/15 to 8/2) to provide the title compound (**S18**) as an unstable white solid (foam) which was quickly taken on to the next step.

tert-butyl (S)-(1-azido-3-(naphthalen-1-yl)propan-2-yl)carbamate (S19)

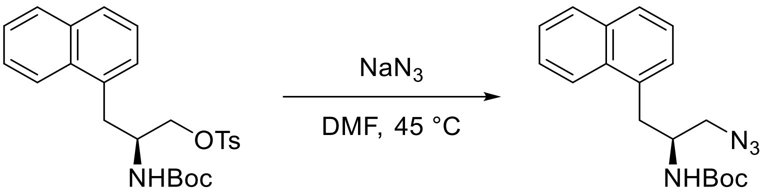

iv. To a stirred solution of (S)-2-((tert-butoxycarbonyl)amino)-3-(naphthalen-1-yl)propyl 4-methylbenzenesulfonate (2.10 g, 4.61 mmol, 1.00 eq) in DMF (23 ml) was added sodium azide (1.50 g, 23.1 mmol, 5.00 eq) behind a blast shield. The reaction was warmed to 45 °C and stirred until deemed complete by TLC analysis. H_2O (30 ml) was added and the aqueous layer was extracted with EtOAc (3 x 30 ml). The combined organic phases were washed with brine, dried over $MgSO_4$, filtered, and concentrated *in vacuo* behind a blast shield. The crude product was purified by silica gel column chromatography (pentane/EtOAc = 8/2) to provide the title compound (**S19**) as a white solid in 65% yield (0.975 g).

^{1}H NMR (400 MHz, $CDCl_3$) δ(ppm): 8.17 (d, *J* = 8.5 Hz, 1H, Ar<u>H</u>), 7.87 (dd, *J* = 8.0, 1.5 Hz, 1H, Ar<u>H</u>), 7.77 (d, *J* = 8.0 Hz, 1H, Ar<u>H</u>), 7.57 (t, *J* = 7.5 Hz, 1H, Ar<u>H</u>), 7.50 (ddd, *J* = 8.0, 7.0, 1.5 Hz, 1H, Ar<u>H</u>), 7.42 (dd, *J* = 8.0, 7.0 Hz, 1H, Ar<u>H</u>), 7.35 (d, *J* = 7.0 Hz, 1H, Ar<u>H</u>), 4.80 (d, *J* = 8.5 Hz, 1H, N<u>H</u>), 4.21 – 4.03 (m, 1H, C<u>H</u>NH), 3.43 (m, 2H, one of $C\underline{H_A}H_BAr$ and one of $C\underline{H_A}H_BN_3$), 3.33 (dd, *J* = 12.5, 4.0 Hz, 1H, one of $CH_A\underline{H_B}N_3$), 3.20 (dd, *J* = 14.0, 9.0 Hz, 1H, one of $CH_A\underline{H_B}Ar$), 1.44 (s, 9H, $C(C\underline{H_3})_3$).

^{13}C NMR (101 MHz, $CDCl_3$) δ(ppm): 155.3 (<u>C</u>=O), 134.1 (Ar<u>C</u>), 133.6 (Ar<u>C</u>), 132.2 (Ar<u>C</u>), 129.0 (Ar<u>C</u>H), 127.9 (Ar<u>C</u>H), 127.8 (Ar<u>C</u>H), 126.6 (Ar<u>C</u>H), 125.9 (Ar<u>C</u>H), 125.6 (Ar<u>C</u>H), 123.9 (Ar<u>C</u>H), 79.9 ($O\underline{C}(CH_3)_3$), 53.3 ($\underline{C}H_2N_3$), 50.8 (<u>C</u>HNH), 35.6 ($\underline{C}H_2Ar$), 28.5 ($OC(\underline{C}H_3)_3$).

IR (powder) ν_{max}/cm^{-1}: 3361 (N-H), 2093 (N=N=N), 1687 (C=O).

HRMS (ES+) exact mass calculated for $[M+Na]^+$ $(C_{18}H_{22}N_4NaO_2)^+$ requires *m/z* 349.1635, found *m/z* 349.1634.

$[\alpha]_D^{25}$ = +5.2 (*c* 0.47, $CHCl_3$).

(R)-2-(3-((S)-1-azido-3-(naphthalen-1-yl)propan-2-yl)thioureido)-N-benzhydryl-3,3-dimethylbutanamide (S20)

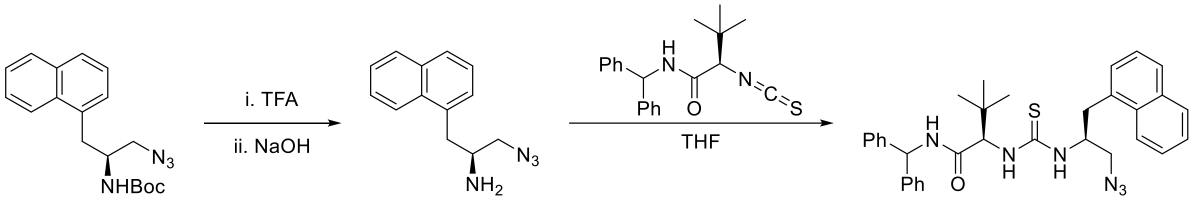

v. According to a literature procedure,[16] TFA (3 ml (1ml/mmol)) was added to (S)-1-azido-3-(naphthalen-1-yl)propan-2-amine (975 mg, 2.99 mmol, 1.00 eq). The reaction was stirred at 0 °C and allowed to warm to ambient temperature over 2h. The reaction was then concentrated under a stream of N_2 and diluted with Et_2O (15 ml) and H_2O (9 ml). NaOH pellets were added with stirring and the biphasic mixture was brought to pH 14. The phases were partitioned and the aqueous phase was extracted with Et_2O (2 x 30 ml), washed with brine, dried over $MgSO_4$, filtered, and concentrated *in vacuo* behind a blast shield. The amine was then diluted with THF (10.3 ml) and (R)-*N*-benzhydryl-2-isothiocyanato-3,3-dimethylbutanamide[16] (1.11 g, 3.29 mmol, 1.10 eq) was added. Once reaction completion was observed by TLC analysis the reaction mixture was concentrated *in vacuo* and purified by silica gel column chromatography (pentane/Et_2O = 7/3 to 6/4) to provide the title compound (**S20**) as a white solid in 85% yield (1.44 g).

^{1}H NMR (400 MHz, Methanol-*d*$_4$) δ(ppm): 8.39 (dd, *J* = 8.5, 1.0 Hz, 1H, ArC$\underline{H}$), 7.90 – 7.82 (m, 1H, ArC$\underline{H}$), 7.76 (dd, *J* = 6.5, 2.5 Hz, 1H, ArC$\underline{H}$), 7.54 (ddd, *J* = 8.5, 7.0, 1.5 Hz, 1H, ArC$\underline{H}$), 7.48 (ddd, *J* = 8.0, 7.0, 1.0 Hz, 1H, ArC$\underline{H}$), 7.44 – 7.36 (m, 2H, ArC$\underline{H}$), 7.34 – 7.16 (m, 10H, ArC$\underline{H}$), 6.19 (t, *J* = 4.0 Hz, 1H, C$\underline{H}$(Ph)$_2$), 5.10 – 5.00 (m, 1H, C$\underline{H}$(CH$_2$)$_2$), 4.96 (s, 1H, C$\underline{H}$C(CH$_3$)$_3$), 3.55 – 3.39 (m, 2H, one of C$\underline{H_A}$H$_B$Ar and one of C$\underline{H_A}$H$_B$N$_3$), 3.39 – 3.17 (m, 2H, one of CH$_A\underline{H_B}$Ar and one of CH$_A\underline{H_B}$N$_3$ and solvent residue peak), 0.97 (s, 9H, C(C$\underline{H_3}$)$_3$).

^{13}C NMR (101 MHz, Methanol-*d*$_4$) δ(ppm): 183.2 ($\underline{C}$=S), 171.2 ($\underline{C}$=O), 141.7 (Ar$\underline{C}$), 141.3 (Ar$\underline{C}$), 134.1 (Ar$\underline{C}$), 133.7 (Ar$\underline{C}$), 133.3 (Ar$\underline{C}$), 128.4 (Ar$\underline{C}$H), 128.1 (Ar$\underline{C}$H), 128.0 (Ar$\underline{C}$H), 127.9 (Ar$\underline{C}$H), 127.4 (Ar$\underline{C}$H), 127.3 (Ar$\underline{C}$H), 127.1 (Ar$\underline{C}$H), 127.1 (Ar$\underline{C}$H), 126.7 (Ar$\underline{C}$H), 125.8 (Ar$\underline{C}$H), 125.3 (Ar$\underline{C}$H), 125.0 (Ar$\underline{C}$H), 123.9 (Ar$\underline{C}$H), 65.1 ($\underline{C}$HC(=O)), 56.8 ($\underline{C}$H(Ph)$_2$), 53.7 ($\underline{C}$H(CH$_2$)$_2$), 53.1 ($\underline{C}$H$_2$N$_3$), 34.7 ($\underline{C}$H$_2$Ar), 34.4 ($\underline{C}$(CH$_3$)$_3$), 26.0 (C($\underline{C}$H$_3$)$_3$).

IR (powder) ν_{max}/cm^{-1}: 3287 (N-H), 2101 (N=N=N), 1644 (C=O).

HRMS (ES+) exact mass calculated for [M+Na]$^+$ ($C_{33}H_{36}N_6NaOS$)$^+$ requires *m/z* 587.2564, found *m/z* 587.2564.

MP: 92-93 °C.

$[\alpha]_D^{25}$ = +65 (c 0.15, $CHCl_3$).

(R)-N-benzhydryl-2-isocyanato-3,3-dimethylbutanamide (S21)

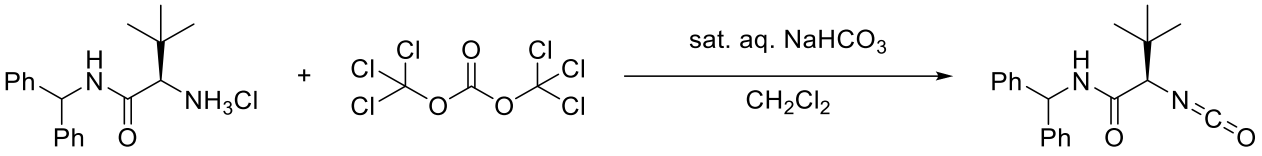

To a stirred solution of (R)-2-amino-N-benzhydryl-3,3-dimethylbutanamide hydrochloride (2.00 g, 6.01 mmol, 3.00 eq) in CH_2Cl_2 (33.4 ml) at 0 °C was added sat. aq. $NaHCO_3$. The reaction was stirred for 20 min at which point triphosgene (595 mg, 2.00 mmol, 1.00 eq) was added and the reaction was warmed to room temperature. Upon completion of the reaction the phases were separated and the aqueous layer was washed with CH_2Cl_2 (3 x 30 ml). The combined organic phases were washed with brine, dried over Na_2SO_4, filtered and concentrated *in vacuo* to provide the title compound (**S21**) as a white solid in 66% yield (1.27 g) which was taken on without further purification.

^{1}H NMR (400 MHz, $CDCl_3$) δ(ppm): 7.37 – 7.05 (m, 10H, Ar<u>H</u>), 6.53 (bd, J = 8.0 Hz, 1H, N<u>H</u>), 6.18 (d, J = 8.0 Hz, 1H, C<u>H</u>NH), 3.71 (s, 1H, C<u>H</u>N=C=O), 0.95 (s, 9H, C(C<u>H</u>$_3$)$_3$).

^{13}C NMR (101 MHz, $CDCl_3$) δ(ppm): 167.7 (NH<u>C</u>(=O)), 141.1 (Ar<u>C</u> (rotamer A)), 141.0 (Ar<u>C</u> (rotamer B)), 128.9 (2 x Ar<u>C</u>H (rotamer A and B)), 127.8 (Ar<u>C</u>H), 127.6 (N=<u>C</u>=O), 127.5 (Ar<u>C</u>H), 68.7 (<u>C</u>HN=C=O), 57.4 (<u>C</u>HNH), 36.3 (<u>C</u>(CH$_3$)$_3$), 26.8 (C(<u>C</u>H$_3$)$_3$).

IR (powder) ν_{max}/cm^{-1}: 3659 (N-H), 1651 (N=C=O), 1651 (NHC=O).

HRMS (ES+) exact mass calculated for $[M+Na]^+$ $(C_{20}H_{22}N_2NaO_2)^+$ requires *m/z* 345.1574, found *m/z* 345.1574.

$[\alpha]_D^{25}$ = +2.6 (c 1.06, $CHCl_3$).

(R)-2-(3-((S)-1-azido-3-(naphthalen-1-yl)propan-2-yl)ureido)-N-benzhydryl-3,3-dimethylbutanamide (S22)

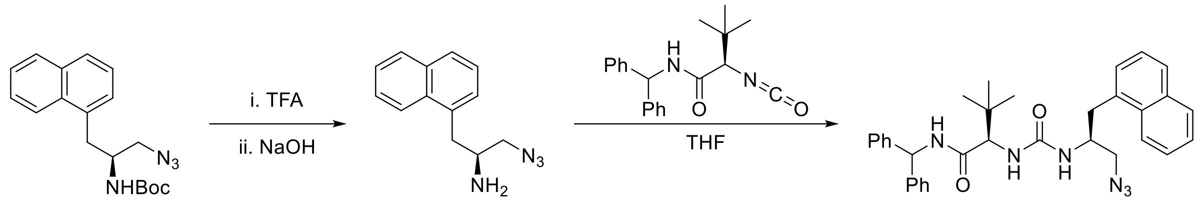

TFA (1.2 ml (1ml/mmol)) was added to (S)-1-azido-3-(naphthalen-1-yl)propan-2-amine (389 mg, 1.19 mmol, 1.00 eq). The reaction was stirred at 0 °C and allowed to warm to ambient temperature over 2h. The reaction was then concentrated under a stream of N_2 and diluted with Et_2O (6 ml) and H_2O (3.6 ml). NaOH pellets were added with stirring and the biphasic mixture was brought to pH 14. The phases were partitioned and the aqueous phase was extracted with Et_2O (2 x 30 ml), washed with brine, dried over $MgSO_4$, filtered, and concentrated *in vacuo* behind a blast shield. The amine was then diluted with THF (3.8 ml) and (R)-N-benzhydryl-2-isocyanato-3,3-dimethylbutanamide (387 mg, 1.20 mmol, 1.10 eq) was added. Once reaction completion was observed by TLC analysis the reaction mixture was concentrated *in vacuo* and purified by silica gel column chromatography (pentane/Et_2O = 7/3 to 6/4) to provide the title compound (**S22**) as a white solid.

^{1}H NMR (500 MHz, Methanol-d_4) δ(ppm): 8.19 (d, *J* = 8.5 Hz, 1H, ArH), 7.85 (dd, *J* = 8.0, 1.5 Hz, 1H, ArH), 7.74 (dd, *J* = 7.0, 2.5 Hz, 1H, ArH), 7.53 (ddd, *J* = 8.5, 7.0, 1.4 Hz, 1H, ArH), 7.47 (ddd, *J* = 8.0, 7.0, 1.0 Hz, 1H, ArH), 7.40 – 7.34 (m, 2H, ArH), 7.34 – 7.16 (m, 11H, ArH and NH), 6.16 (s, 1H, CH(Ph)$_2$), 4.59 (s, 1H, NH), 4.25 (tt, *J* = 7.0, 5.0 Hz, 1H, CHCH$_2$N$_3$), 4.07 (s, 1H, CHC(=O)), 3.42 – 3.36 (m, 2H, CH$_2$N$_3$), 3.36 – 3.27 (m, 1H, one of CH$_A$H$_B$CHCH$_2$N$_3$ and solvent residue peak), 3.21 (dd, *J* = 14.0, 7.5 Hz, 1H, one of CH$_A$H$_B$CHCH$_2$N$_3$), 0.86 (s, 9H, C(CH$_3$)$_3$).

^{13}C NMR (126 MHz, Methanol-d_4) δ(ppm): 172.9 (CHC(=O)), 159.7 (NHC(=O)NH), 143.0 (ArC), 142.8 (ArC), 135.5 (ArC), 135.3 (ArC), 133.5 (ArC), 129.8 (ArCH), 129.5 (ArCH), 129.4 (ArCH), 129.1 (ArCH), 128.7 (ArCH), 128.5 (ArCH), 128.4 (ArCH), 128.2 (ArCH), 127.2 (ArCH), 126.6 (ArCH), 126.4 (ArCH), 124.9 (ArCH), 62.3 (CHC(=O)), 58.1 (CH(Ph)$_2$), 55.6 (CH$_2$N$_3$), 51.7 (CHCH$_2$N$_3$), 36.8 (CH$_2$CHCH$_2$N$_3$), 35.6 (C(CH$_3$)$_3$), 27.1 (C(CH$_3$)$_3$).

IR (powder) ν_{max}/cm^{-1}: 3659 (N-H), 2100 (N=N=N), 1629 (C=O).

HRMS (ES+) exact mass calculated for $[M+Na]^+$ $(C_{33}H_{36}N_6NaO_2)^+$ requires *m/z* 571.2792, found *m/z* 571.2784.

MP: 107-108 ˚C.

$[\alpha]_D^{25}$ = +29.2 (*c* 0.59, $CHCl_3$).

C1

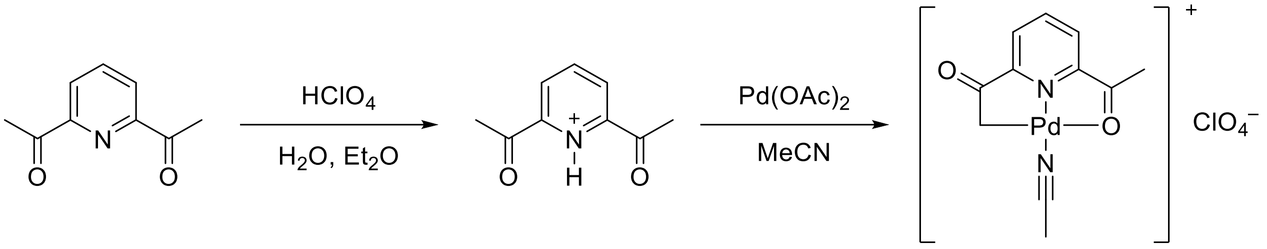

i. According to a literature procedure,[1b] 48-50% aq. $HClO_4$ (3.6 g, 17.9 mmol, 1.5 eq) was slowly added to a solution of 1,1'-(pyridine-2,6-diyl)bis(ethan-1-one) (1.95 g, 12.0 mmol, 1.00 eq) in Et_2O (40 ml). The reaction mixture was stirred for 2 h. The precipitate was filtered, washed with Et_2O and recrystallized from acetone/Et_2O to provide the desired salt in 52% yield (1.64 g).

ii. The salt (894 mg, 3.39 mmol, 1.00 eq) was then stirred in acetonitrile (40 ml) with $Pd(OAc)_2$ (762 mg, 3.39 mmol, 1.00 eq) for 2 h. The reaction mixture was concentrated to dryness. Acetone was added and the mixture concentrated again, this was repeated two more times. The resulting mixture was dissolved in acetonitrile (2 ml) and Et_2O (10 ml) was added. The suspension was filtered and washed with Et_2O to provide a brown solid in quantitative yield (1.38 g).

^{1}H NMR (400 MHz, Acetonitrile-d_3) δ(ppm): 8.39 (t, *J* = 8.0 Hz, 1H, Ar<u>H</u>), 8.12 (dd, *J* = 8.0, 1.5 Hz, 1H, Ar<u>H</u>), 8.03 (dd, *J* = 8.0, 1.5 Hz, 1H, Ar<u>H</u>), 3.60 (s, 2H, $C\underline{H_2}$), 2.84 (s, 3H, $C\underline{H_3}$).

^{13}C NMR (101 MHz, Acetonitrile-d_3) δ(ppm): 200.0 ($\underline{C}$=O-Pd), 197.7 ($CH_2\underline{C}$=O), 156.9 (Ar$\underline{C}$), 156.1 (Ar$\underline{C}$), 143.9 (Ar$\underline{C}$H), 129.9 (Ar$\underline{C}$H), 39.1 ($\underline{C}H_2$), 28.5 ($\underline{C}H_3$).

IR (powder) ν_{max}/cm^{-1}: 2328 (C≡N), 1705, 1637 (C=O), 1595 (C=N), 1074 (Cl-O).

Data is consistent with that given in the literature.[1b]

1.7 Deuterium labelling study

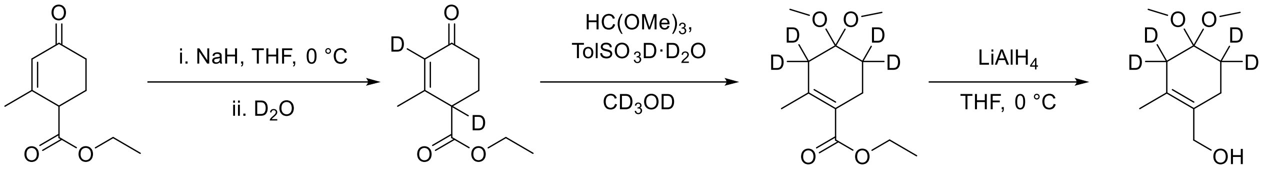

(**S23**) (**S24**) (**S25**)

S23

i. To a stirred solution of 60% NaH (242 mg, 6.04 mmol, 1.10 eq) in THF (27.5 ml) was added Hagemann's ester (1.00 g, 5.49 mmol, 1.00 eq). The reaction was stirred at 0 °C for 10 min at which point D_2O (10 ml) was added. The aqueous phase was extracted with EtOAc (3 x 20 ml). The combined organic phases were washed with brine, dried over Na_2SO_4, filtered and concentrated *in vacuo*. The entire process was repeated a second time to afford **S23** in 72% yield (728 mg) which was taken on without further purification.

S24

ii. *p*TsOH·H_2O (150 mg, 0.790 mmol, 0.200 eq) was dissolved in D_2O (5 ml) and concentrated *in vacuo*. This process was repeated twice to provide *p*TsOD·D_2O. *p*TsOD·D_2O was placed under an atmosphere of Ar and CD_3OD (22 ml) was added. To this solution was added deuterated **S23** (728 mg, 3.95 ml, 1.00 eq) followed by trimethyl orthoformate (1.30 ml, 11.9 mmol, 3.00 eq). The reaction was stirred for 3 hours at which point a saturated solution of K_2CO_3 in D_2O was added (20 ml). The aqueous layer was extracted with EtOAc (3 x 20 ml). The combined organic phases were washed with brine, dried over Na_2SO_4, filtered and concentrated *in vacuo* to afford the crude product (**S24**) as a yellow oil in 65% yield (614 mg) which was taken on without further purification.

S25

iii. To a solution of **S24** (614 mg, 2.67 mmol, 1.00 eq) in THF (4.5 ml) at 0° C was added $LiAlH_4$ (506 mg, 13.3 mmol, 5.00 eq). The reaction was stirred for 2 h at which point the reaction was diluted with Et_2O (20 ml) and cooled to 0 °C. H_2O (0.5 ml) was added, followed by 15% aqueous NaOH solution (0.5 ml), followed by H_2O (1.5 ml). The biphasic mixture was stirred for 15 min at rt at which point $MgSO_4$ was added and stirring was maintained for a further 15 min. The mixture was filtered and concentrated *in vacuo*. The crude product was purified by silica gel column chromatography (Et_2O) to provide **S25** in 46% yield (243 mg).

^{1}H NMR (500 MHz, $CDCl_3$) δ(ppm): 4.12 (d, *J* = 5.0 Hz, 2H, C<u>H</u>$_2$OH), 3.22 (s, C(OC<u>H</u>$_3$)$_2$ (partial deuterium incorporation)), 2.18 (s, 2H, CC<u>H</u>$_2$C(OCH_3)$_2$ (deuterated) and C<u>H</u>$_2$$CH_2$C($OCH_3$)$_2$), 1.81 (m, CH_2C<u>H</u>$_2$C(OCH_3)$_2$ (deuterated)), 1.73 – 1.63 (m, 2H, CC<u>H</u>$_3$ (partial deuterium incorporation)), 1.33 (t, *J* = 5.5 Hz, 1H, OH).

^{13}C NMR (126 MHz, $CDCl_3$) δ(ppm): 129.5 (<u>C</u>CH_2OH), 127.4 (<u>C</u>CH_3), 99.5 (<u>C</u>(OCH_3)$_2$), 62.5(<u>C</u>H_2OH), 48.0 (C(O<u>C</u>H_3)$_2$), 47.8 – 46.6 (m, C(O<u>C</u>D_3)$_2$), 40.0 (m, C<u>C</u>D_2C(OCH_3)$_2$), 28.9 – 27.7 (m, <u>C</u>$H_2$$CD_2$C($OCH_3$)$_2$), 25.9 – 25.4 (m, CH_2<u>C</u>D_2C(OCH_3)$_2$), 19.1 – 17.7 (m, C<u>C</u>H_3).

IR (film) ν_{max}/cm^{-1}: 3401 (O-H), 2215, 2129, 2070 (C-H), 1118, 1052 (C-O).

^{1}H NMR (500 MHz, $CDCl_3$) of **S25** overlaid with **S2**.

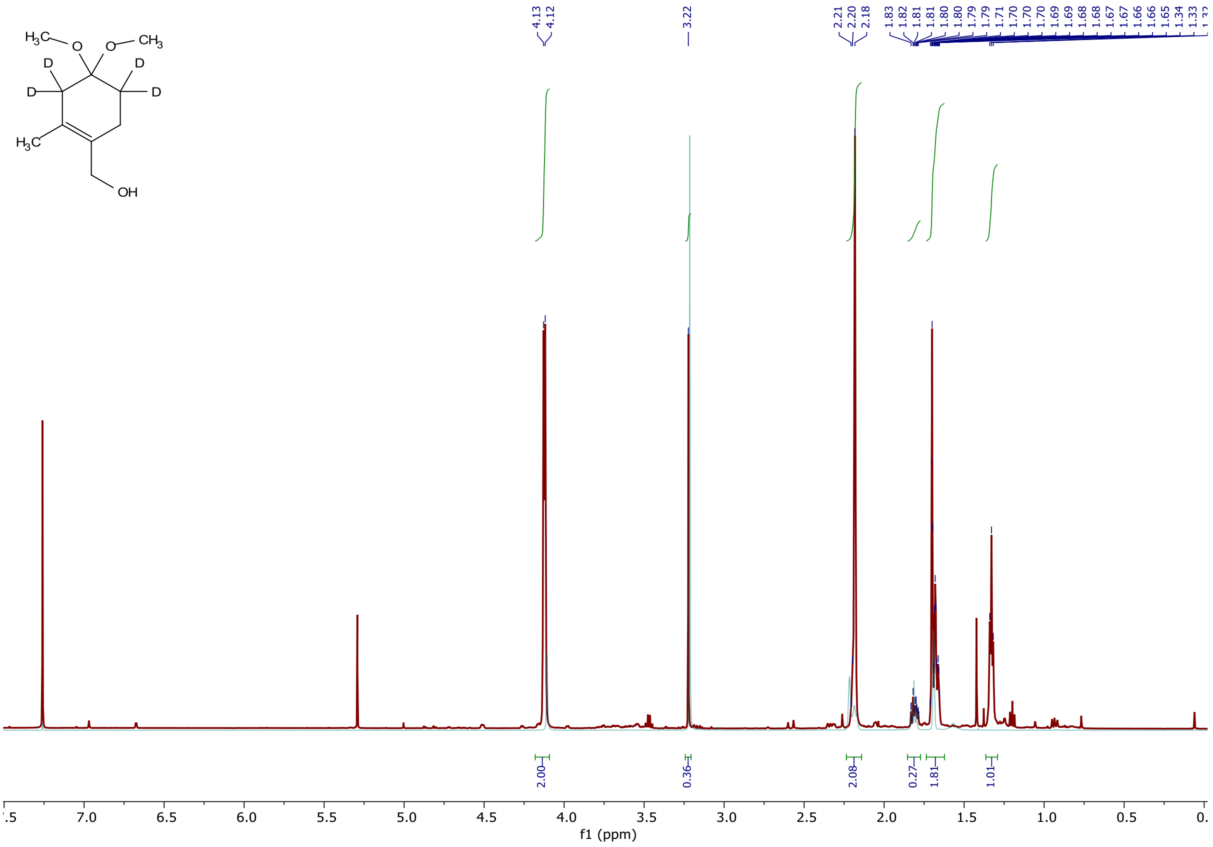

^{13}C NMR (126 MHz, $CDCl_3$) of **S25** overlaid with **S2.**

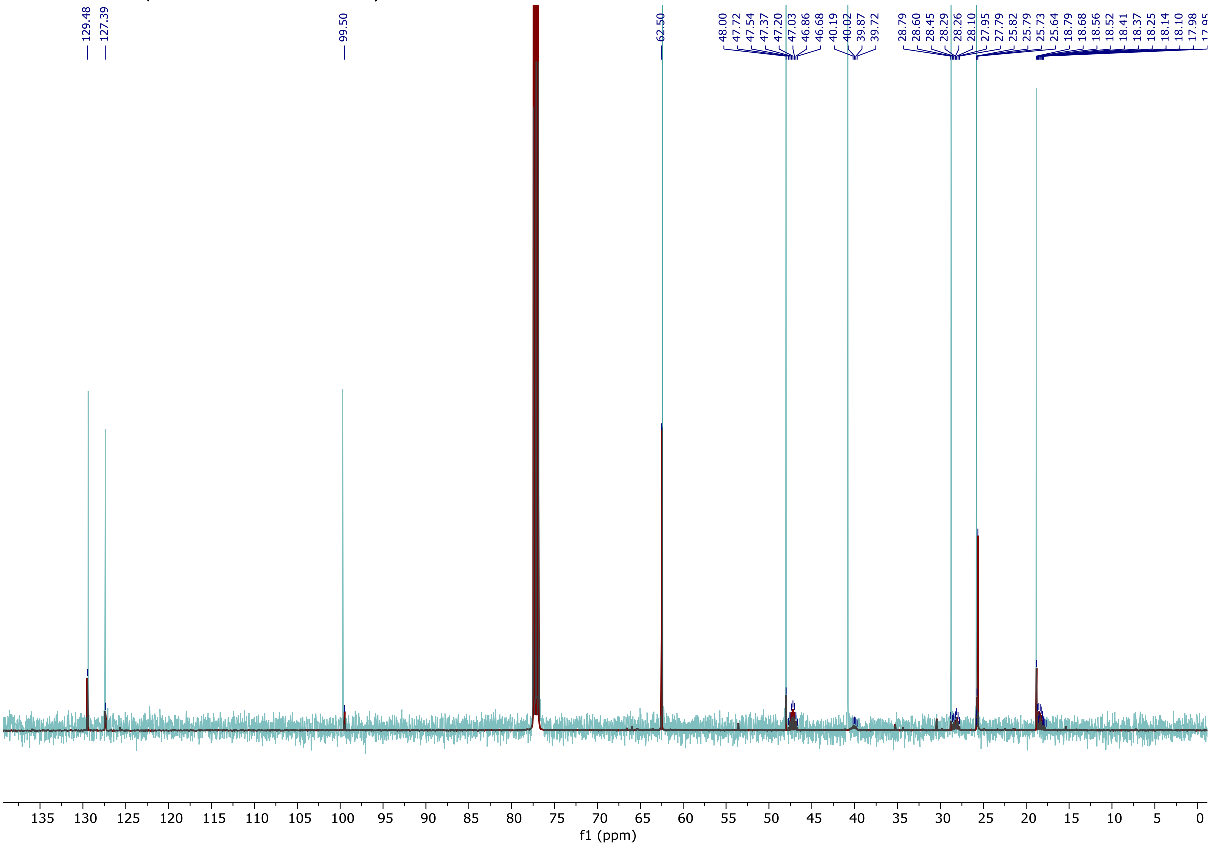

S26

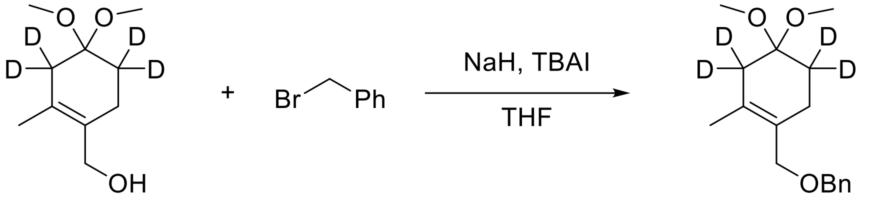

(**S25**) (**S26**)

To a solution of **S25** (282 mg, 1.22 mmol, 1.00 eq) in THF (3.1 ml) at 0 °C was added 60% NaH (98 mg, 2.45 mmol, 2.00 eq). The suspension was stirred for 30 min at which point BnBr (315 mg, 1.84 mmol, 1.50 eq) and TBAI (44.0 mg, 0.12 mmol, 0.100 eq) were added. The reaction was stirred overnight at which point sat. aq NH_4Cl (20 ml) was added. The aqueous layer was extracted with EtOAc (3 x 20 ml). The combined organic phases were washed with brine, dried over Na_2SO_4, filtered and concentrated *in vacuo*. The crude product was purified by silica gel column chromatography (pentane/Et_2O 9/1 to 8/2) to afford the **S26** as a yellow oil in 68% yield (238 mg).

^{1}H NMR (500 MHz, $CDCl_3$) δ(ppm): 7.34 (d, *J* = 4.5 Hz, 4H, Ar$\underline{H}$), 7.30 – 7.26 (m, 1H, Ar$\underline{H}$), 4.46 (s, 2H, C$\underline{H_2}$Ph), 4.01 (s, 2H, C$\underline{H_2}$OCH_2Ph), 3.23 (s, C(OC$\underline{H_3}$)$_2$ (partial deuterium incorporation)), 2.22 (s, C$\underline{CH_2}$C(OCH_3), (deuterated)), 2.18 (s, 2H, C$\underline{H_2}$$CH_2$C($OCH_3$)), 1.81 (dt, *J* = 9.5, 6.5 Hz, CH_2C$\underline{H_2}$C(OCH_3), (deuterated)), 1.70 – 1.60 (m, C$\underline{CH_3}$ (partial deuterium incorporation)).

^{13}C NMR (126 MHz, $CDCl_3$) δ(ppm): 138.8 (Ar$\underline{C}$), 128.5 ($\underline{C}$$CH_2$OBn), 127.9 (Ar$\underline{C}$H), 127.7 (Ar$\underline{C}$H), 127.1 ($\underline{C}$$CH_3$), 99.5 ($\underline{C}$($OCH_3$)$_2$), 72.0 ($\underline{C}$$H_2$Ph), 69.3 ($\underline{C}$$H_2$O), 48.0 (O$\underline{C}$$H_3$), 47.2 (m, O$\underline{C}$$D_3$), 41.5 – 39.3 (m, $\underline{C}$$D_2CCH_3$), 28.9 – 27.6 (m, $CH_2$$\underline{C}$$D_2$C($OCH_3$)$_2$), 25.9 (m, $\underline{C}$$H_2$$CH_2$C($OCH_3$)$_2$), 19.2 – 18.0 (m, C$\underline{C}$$H_3$).

IR (film) ν_{max}/cm^{-1}: 3030, 2981, 2916, 2851 (C-H), 1715 (C=O), 1066 (C-O).

^{1}H NMR (500 MHz, $CDCl_3$) of **S26** overlaid with **S3**.

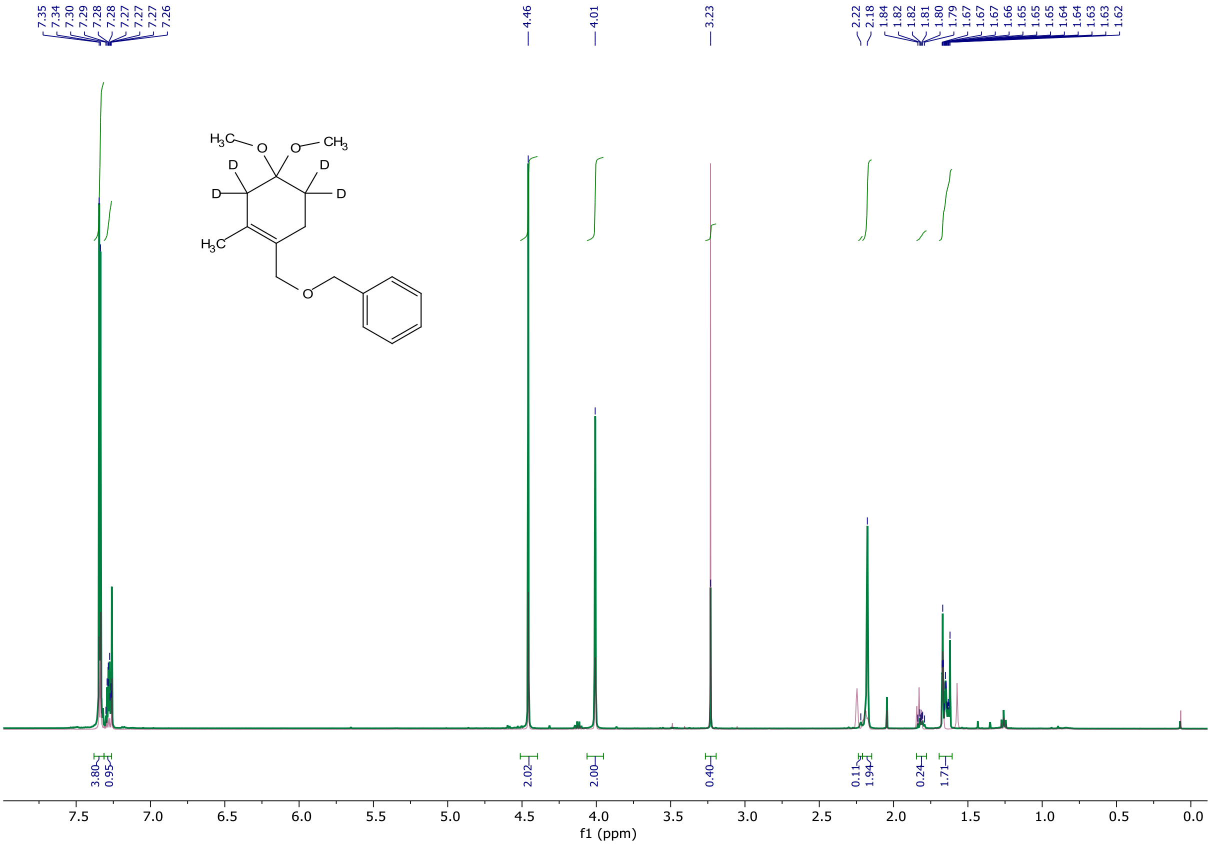

^{13}C NMR (126 MHz, $CDCl_3$) of **S26** overlaid with **S3**

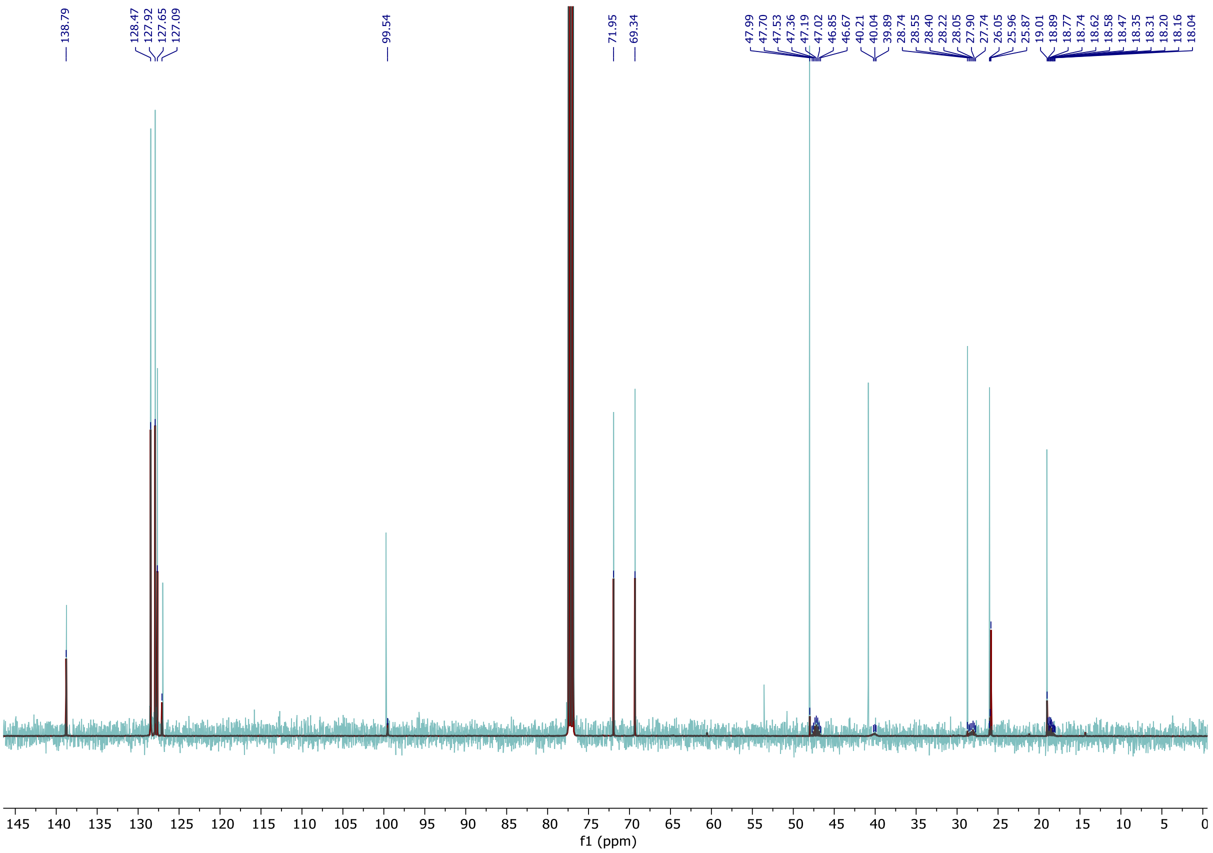

S27

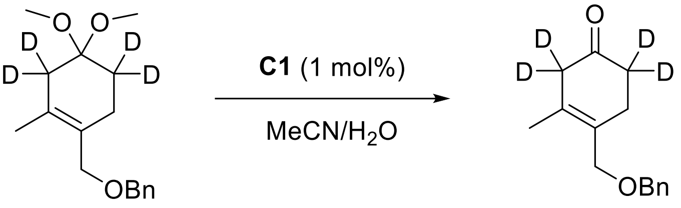

(**S26**) (**S27**)

Compound **S27** was prepared according to general procedure **B** from **S26** (218 mg, 0.78 mmol, 1.00 eq). Upon completion of the reaction as judged by TLC analysis (pentane/Et_2O = 9/1) the reaction mixture was transferred directly to silica gel and purified by silica gel column chromatography (pentane/Et_2O = 95/5) to afford the title compound as a yellow oil. Yield assumed quantitative.

^{1}H NMR (400 MHz, $CDCl_3$) δ(ppm): 7.42 – 7.26 (m, 5H, Ar$\underline{H}$), 4.50 (s, 2H, C$\underline{H_2}$Ph), 4.06 (s, 2H, C$\underline{H_2}$OCH_2Ph), 2.82 (s, 91.5 % D, CC$\underline{H_2}$C(=O)), 2.54 (s, 2H, C$\underline{H_2}$CH_2C(=O)), 2.47 (m, 88% D, CH_2C$\underline{H_2}$C(=O)), 1.76 – 1.66 (m, 48% D, C$\underline{H_3}$).

^{13}C NMR (126 MHz, $CDCl_3$) δ(ppm): 210.9 ($\underline{C}$=O), 138.4 (Ar$\underline{C}$), 129.5 ($CH_3\underline{C}$), 128.6 (Ar$\underline{C}$H), 128.4 (CH_3C=$\underline{C}$), 127.9 (Ar$\underline{C}$H), 127.9 (Ar$\underline{C}$H), 72.4 ($\underline{C}H_2$Ph), 69.1 ($\underline{C}H_2$OCH_2Ph), 45.13 (m, C$\underline{C}H_2$C(=O)), 39.05 – 37.84 (m, $\underline{C}H_2$CH_2C(=O)), 27.88 – 27.59 (m, C$H_2\underline{C}H_2$C(=O)), 18.84 – 17.64 (m, $\underline{C}H_3$).

IR (film) ν_{max}/cm^{-1}: 2030, 2905, 2845 (C-H), 1674 (C=C), 1069 (C-O).

Quantitative ^{1}H NMR (400 MHz, $CDCl_3$) of **S27** overlaid with **1a**.

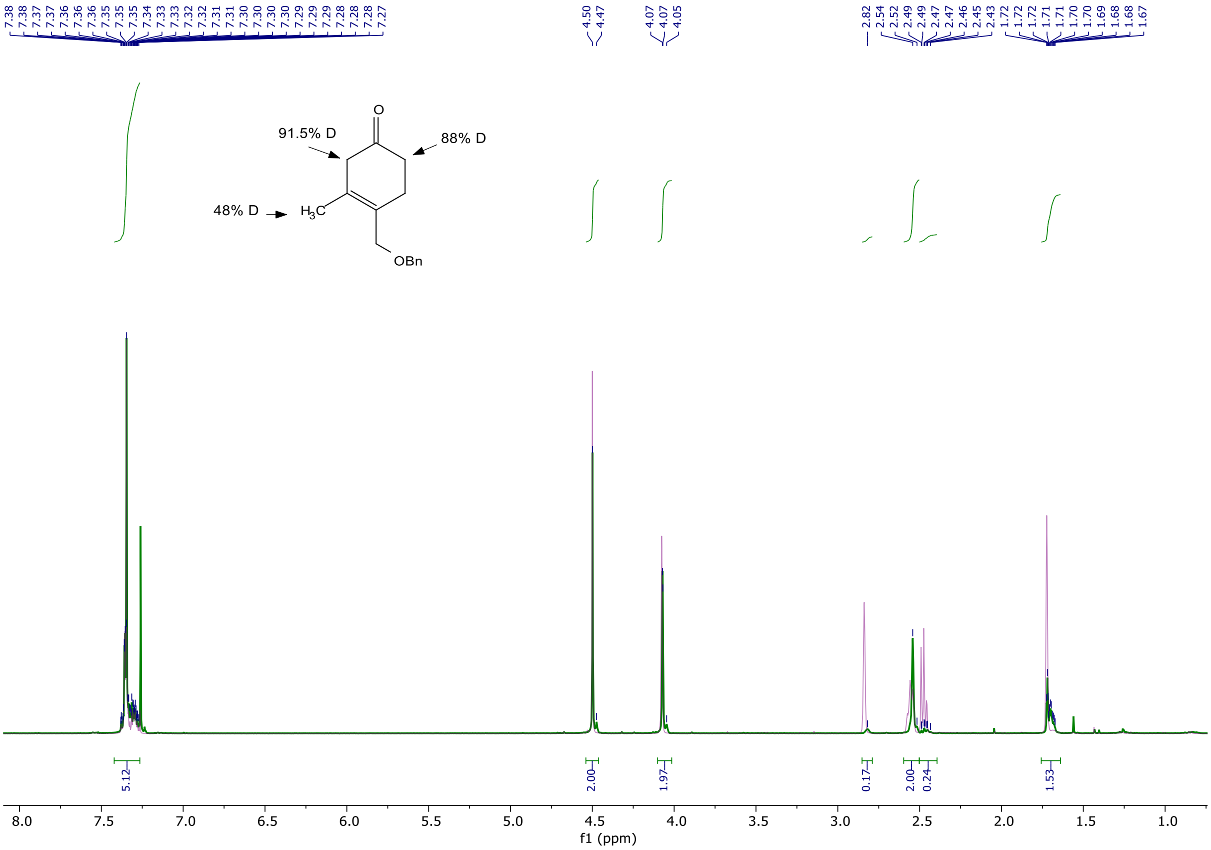

^{13}C NMR (126 MHz, $CDCl_3$) of **S27** overlaid with **1a**.

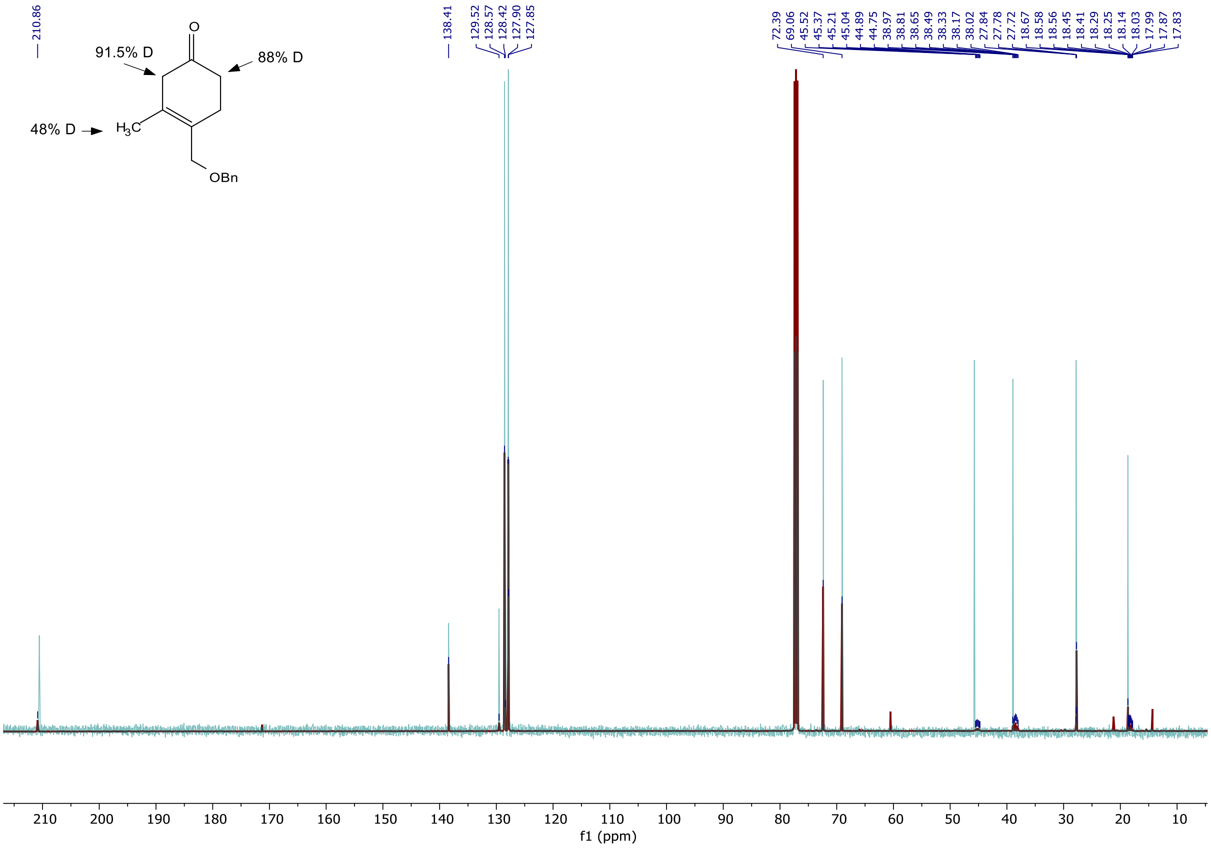

Crossover Experiment

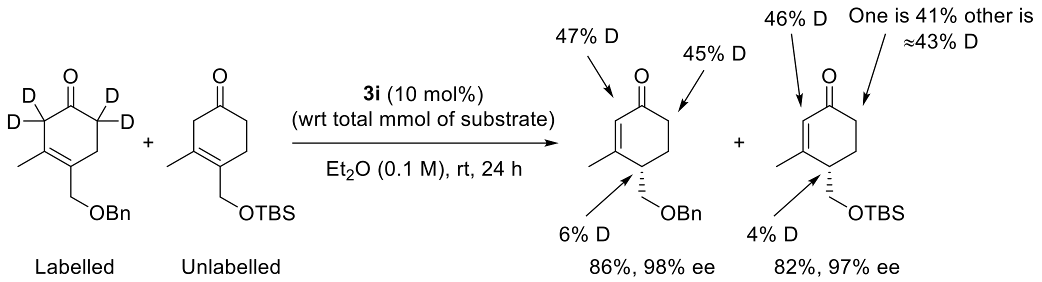

Compounds **S27** and **1f** were subjected to the prototropic shift reaction conditions. Exchange of the alpha protons was observed, resulting in approximately 50% deuterium incorporation in both alpha positions of the final products. This is consistent with the pre-equilibrium described in the literature as well as computational studies.[17] Only low levels of deuterium incorporation were observed at the γ-position consistent with reprotonation being the rate determining step.

Quantitative NMR spectra obtained after crossover experiment

^{1}H NMR (400 MHz, $CDCl_3$)

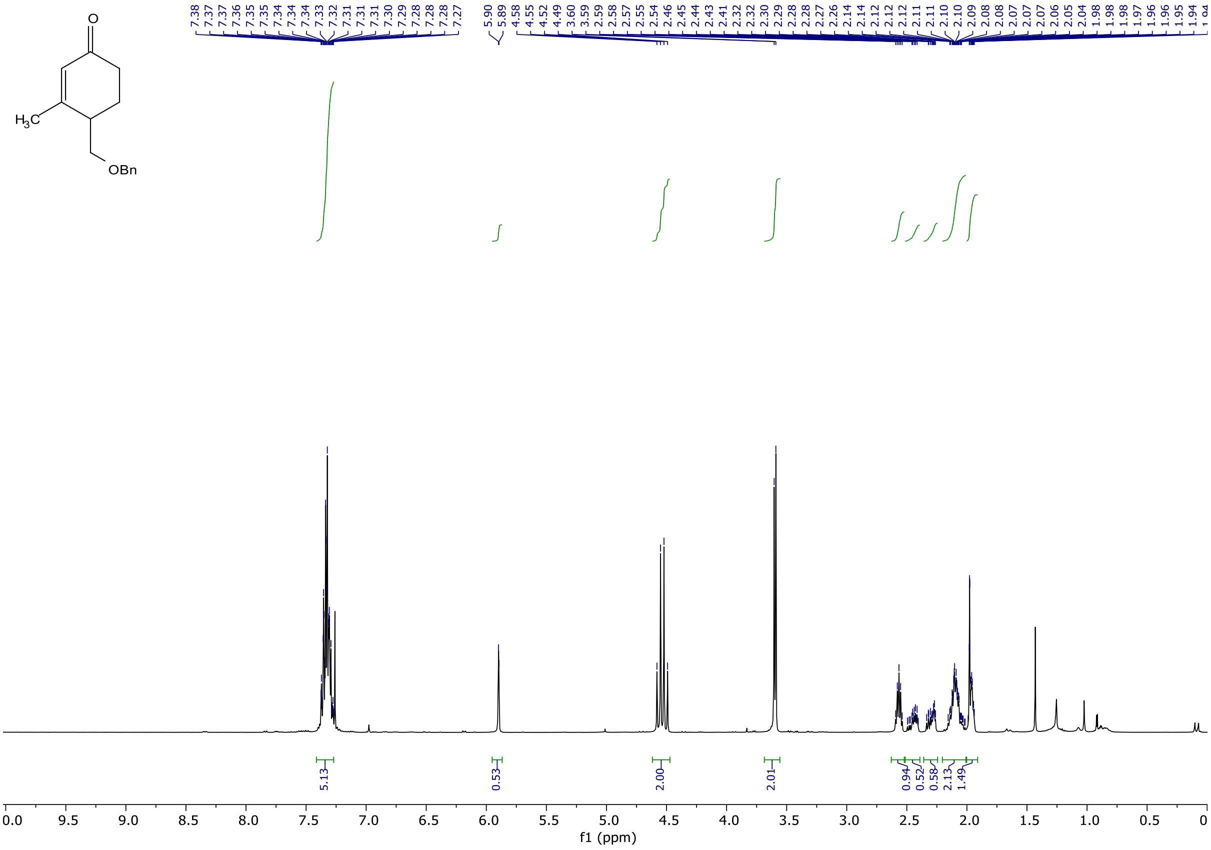

^{1}H NMR (400 MHz, $CDCl_3$)

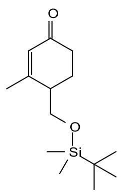

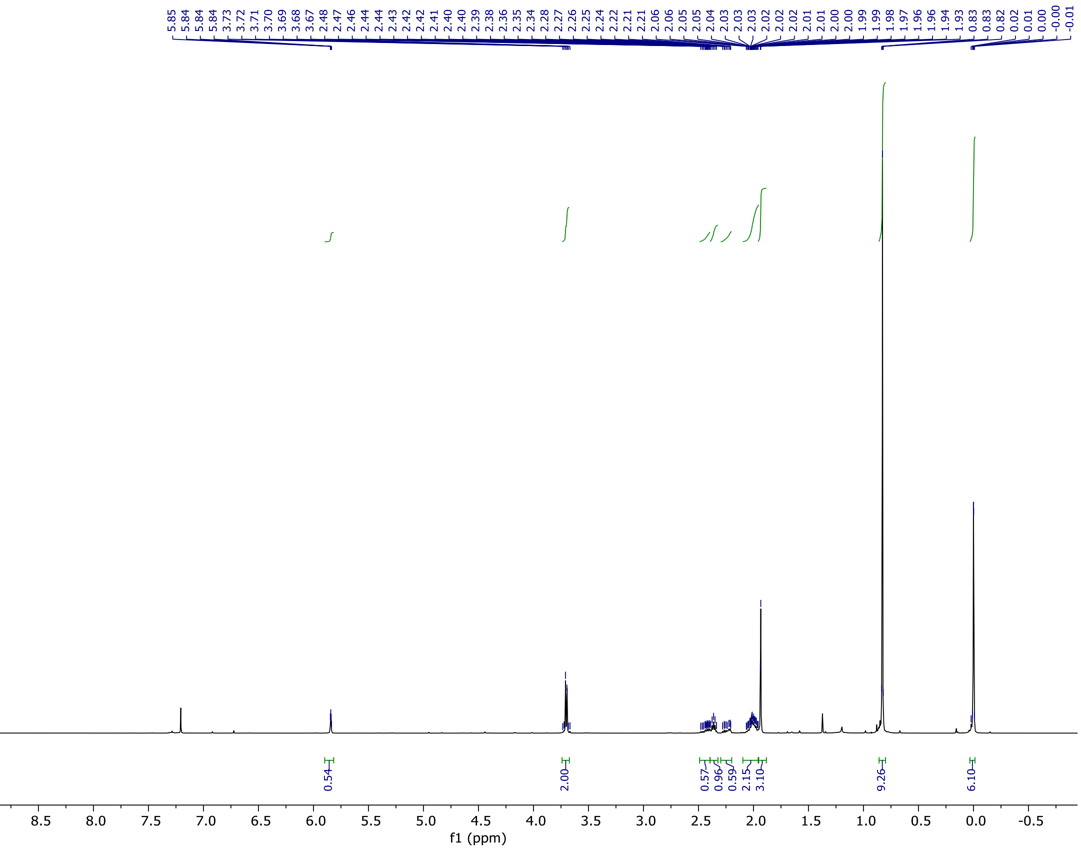

2.1 Single Crystal X-Ray Diffraction Data

CCDC 1982997

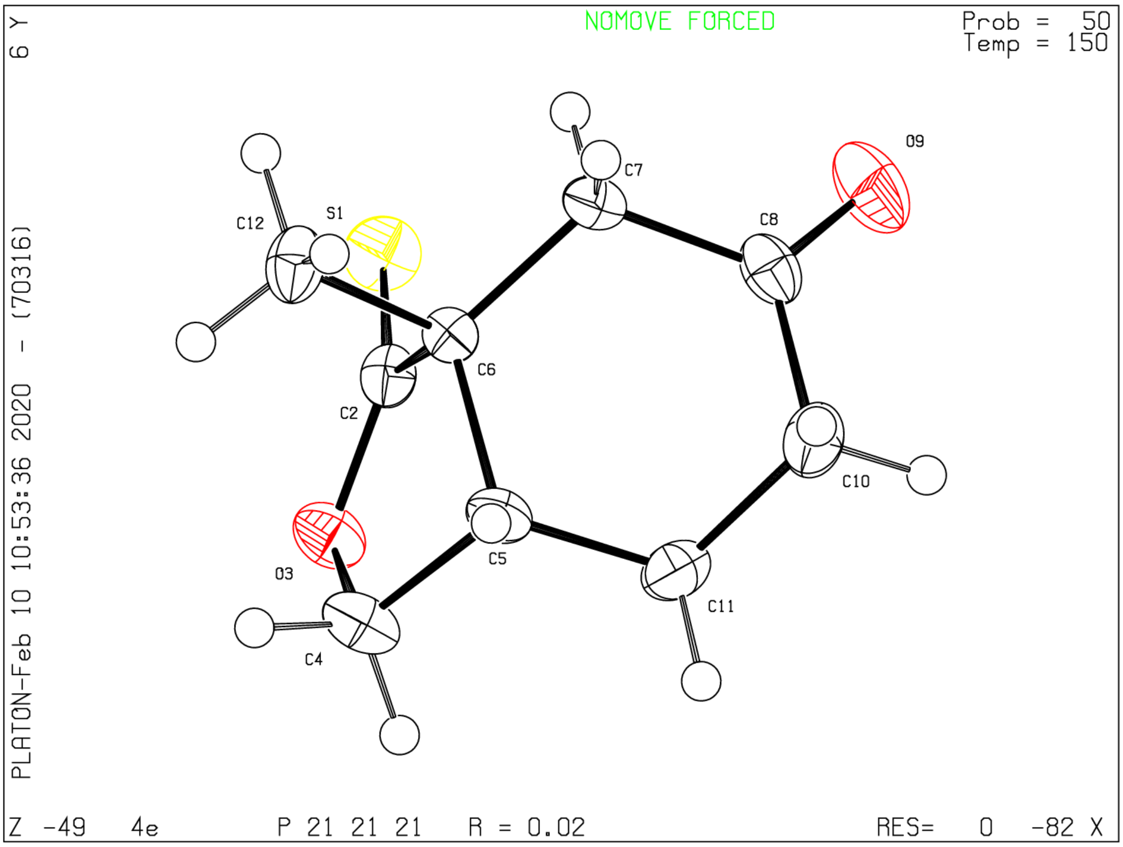

Crystal data	
Chemical formula	$C_9H_{12}O_2S$
M_r	184.26
Crystal system, space group	Orthorhombic, $P2_12_12_1$
Temperature (K)	150
a, *b*, *c* (Å)	8.0589 (1), 9.1929 (1), 12.3333 (2)
V ($Å^3$)	913.71 (2)
Z	4
Radiation type	Cu *K*α
μ (mm^{-1})	2.80
Crystal size (mm)	0.20 × 0.10 × 0.10
Data collection	
Diffractometer	Oxford Diffraction SuperNova
Absorption correction	Multi-scan<br>*CrysAlis PRO* (Rigaku Oxford Diffraction, 2017)

T_{min}, T_{max}	0.64, 0.76
No. of measured, independent and observed [$I > 2.0\sigma(I)$] reflections	14164, 1919, 1910
R_{int}	0.024
$(\sin \theta/\lambda)_{max}$ (Å^{-1})	0.632
Refinement	
$R[F^2 > 2\sigma(F^2)]$, $wR(F^2)$, S	0.021, 0.058, 1.03
No. of reflections	1919
No. of parameters	110
H-atom treatment	H-atom parameters constrained
$\Delta\rho_{max}$, $\Delta\rho_{min}$ (e Å^{-3})	0.20, −0.17
Absolute structure	Parsons, Flack & Wagner (2013), 785 Friedel Pairs
Absolute structure parameter	−0.009 (3)

Computer programs: SuperNova, (Oxford Diffraction, 2010), *CrysAlis PRO* (Rigaku Oxford Diffraction, 2017), *SUPERFLIP* (Palatinus & Chapuis, 2007), *CRYSTALS* (Betteridge *et al.*, 2003), *CAMERON* (Watkin *et al.*, 1996).

Table 2

Selected geometric parameters (Å, º)

S1—C2	1.6260 (11)	C6—C7	1.5333 (14)
C2—O3	1.3329 (14)	C6—C12	1.5426 (14)
C2—C6	1.5216 (14)	C7—C8	1.5042 (15)
O3—C4	1.4706 (14)	C8—O9	1.2122 (15)
C4—C5	1.5178 (15)	C8—C10	1.5028 (16)
C5—C6	1.5422 (14)	C10—C11	1.5249 (17)
C5—C11	1.5290 (16)		
S1—C2—O3	121.15 (9)	C2—C6—C7	114.24 (9)
S1—C2—C6	129.19 (8)	C5—C6—C12	111.30 (9)
O3—C2—C6	109.63 (9)	C2—C6—C12	106.86 (8)
C2—O3—C4	110.43 (8)	C7—C6—C12	108.60 (8)
O3—C4—C5	103.93 (9)	C6—C7—C8	115.71 (9)
C4—C5—C6	101.09 (9)	C7—C8—O9	121.70 (11)
C4—C5—C11	110.47 (10)	C7—C8—C10	115.44 (10)
C6—C5—C11	112.26 (9)	O9—C8—C10	122.83 (12)
C5—C6—C2	100.35 (8)	C8—C10—C11	110.18 (10)
C5—C6—C7	115.08 (9)	C5—C11—C10	112.23 (10)

Alert level C

PLAT230_ALERT_2_C Hirshfeld Test Diff for S1 --C2 . 6.4 s.u.

Alert level G

PLAT791_ALERT_4_G Model has Chirality at C5 (Chiral SPGR)
S Verify

PLAT791_ALERT_4_G Model has Chirality at C6 (Chiral SPGR)
R Verify

2.2 Copies of NMR Spectra

^{1}H NMR (500 MHz, $CDCl_3$) of **S1**

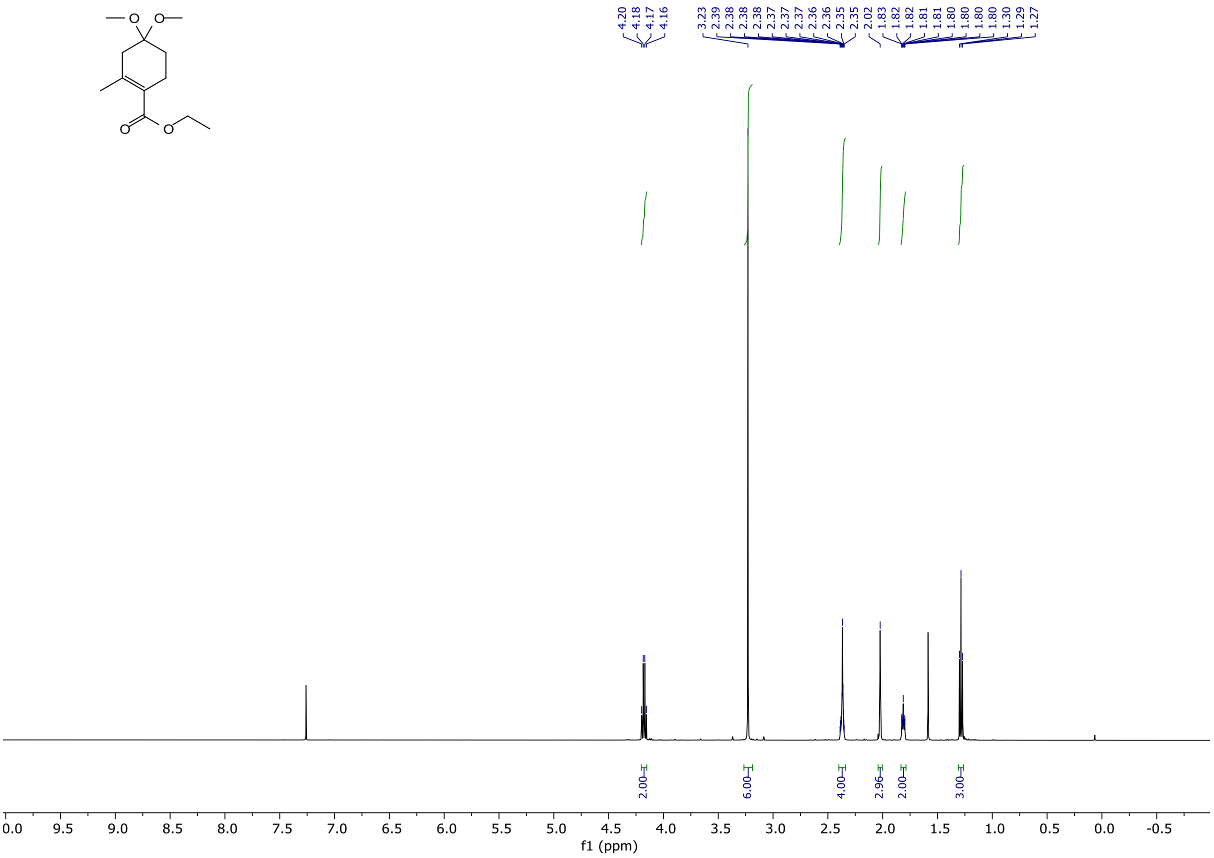

^{13}C NMR (126 MHz, $CDCl_3$) of **S1**

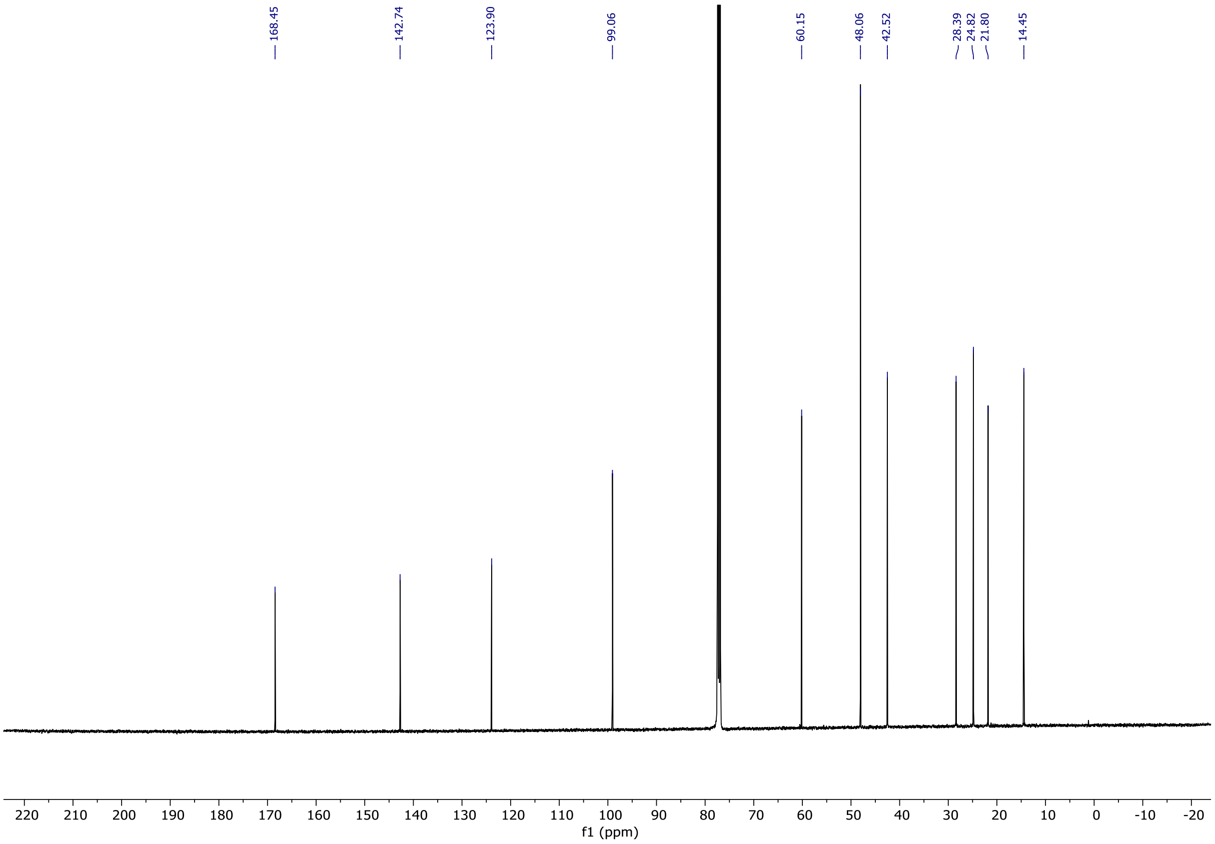

^{1}H NMR (400 MHz, $CDCl_3$) of **S2**

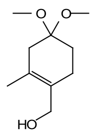

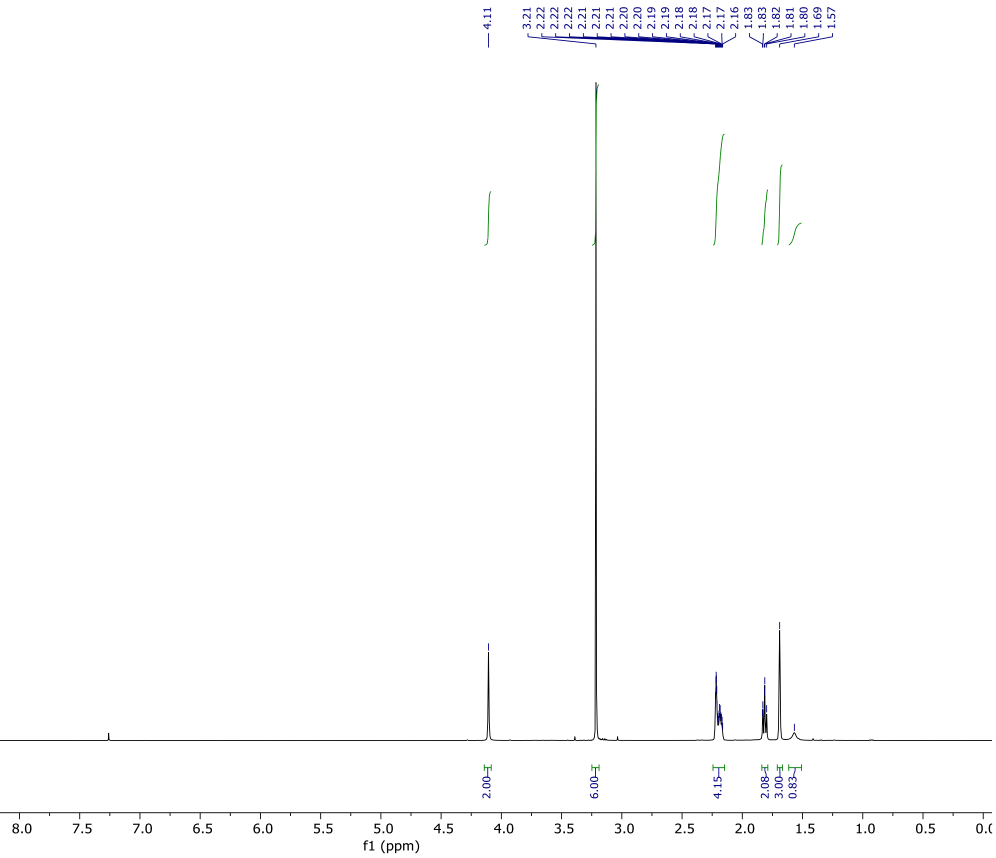

^{13}C NMR (101 MHz, $CDCl_3$) of **S2**

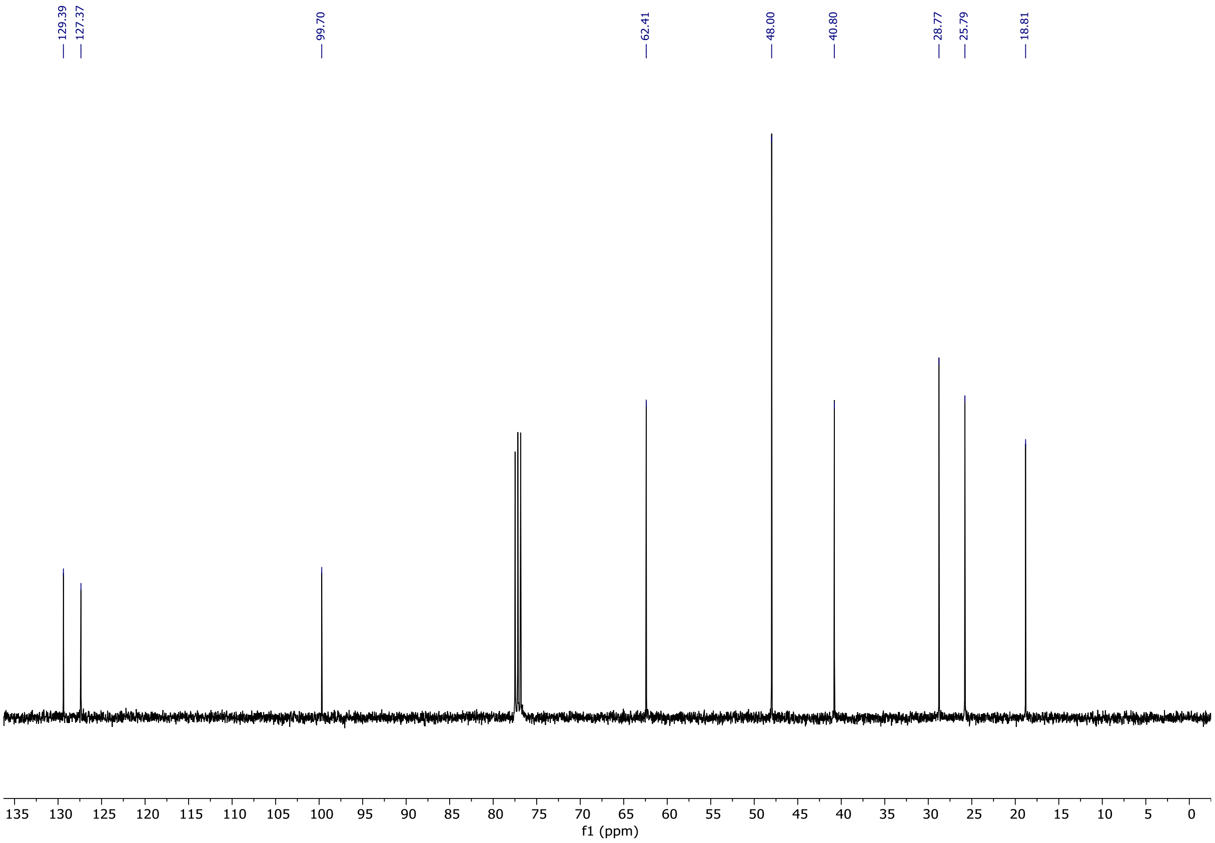

^{1}H NMR (400 MHz, $CDCl_3$) of **S3**

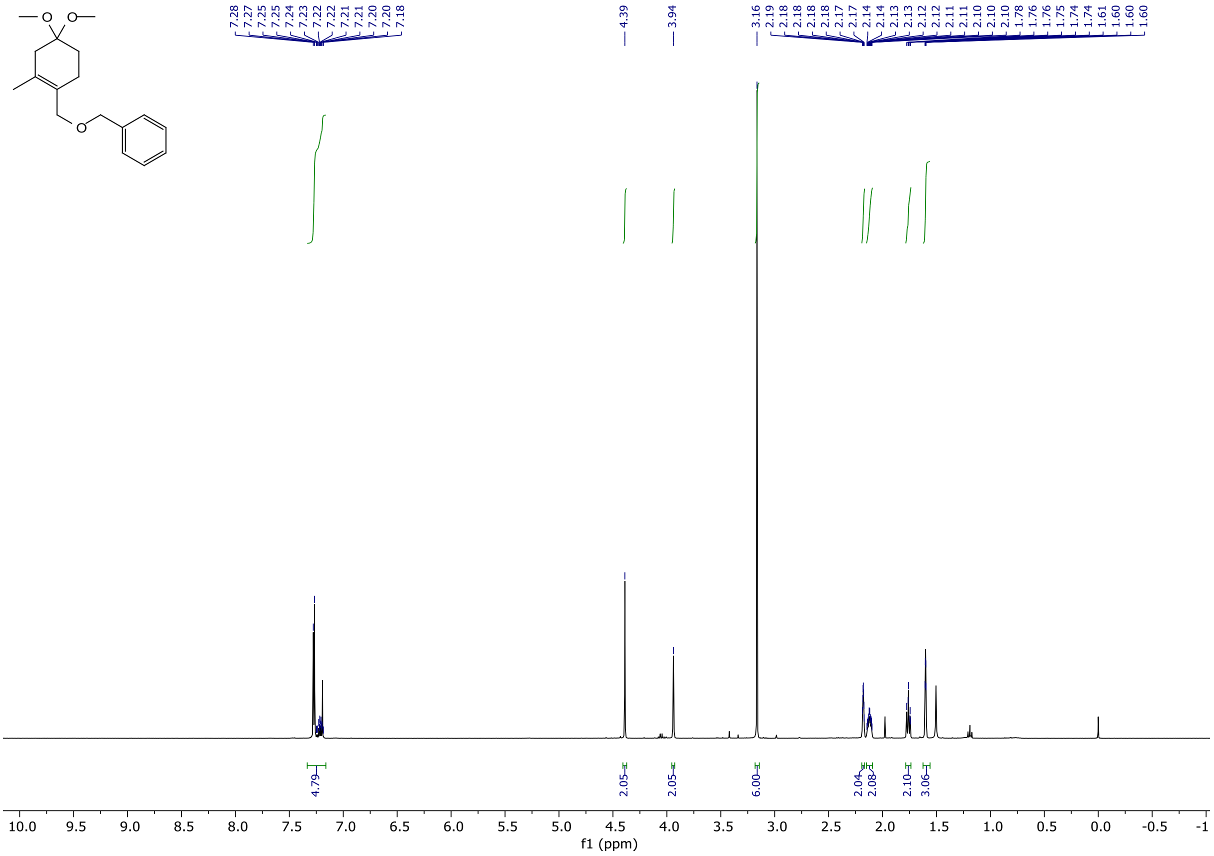

^{13}C NMR (101 MHz, $CDCl_3$) of **S3**

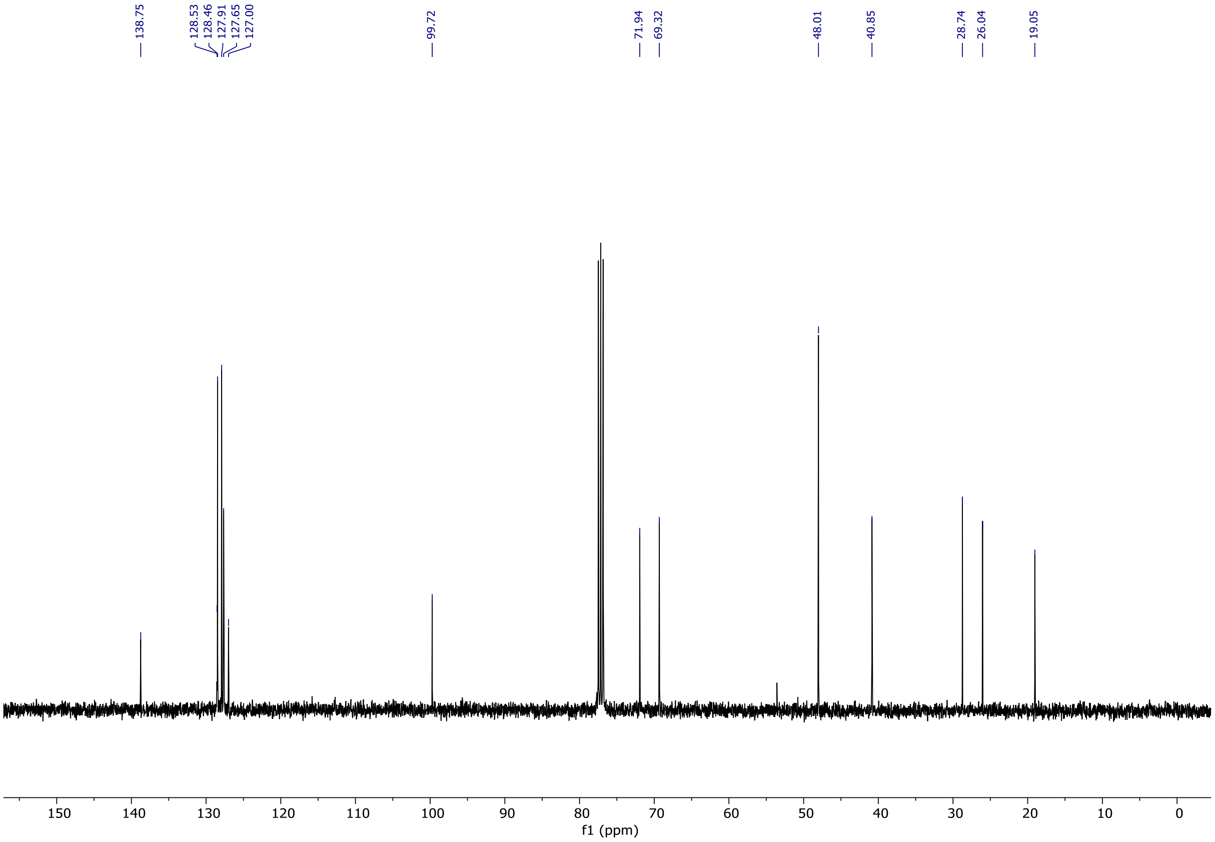

^{1}H NMR (500 MHz, Benzene-d_6) of **S4**

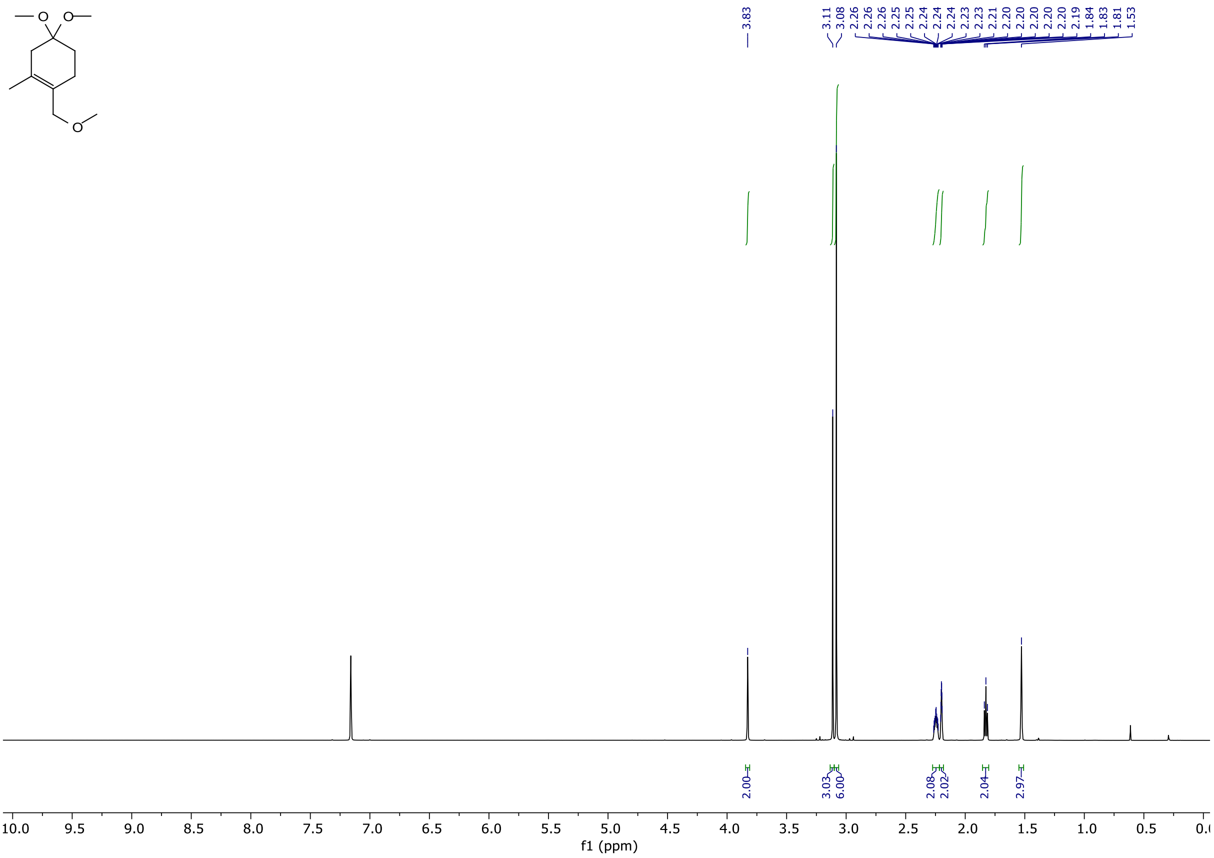

^{13}C NMR (126 MHz, Benzene-d_6) of **S4**

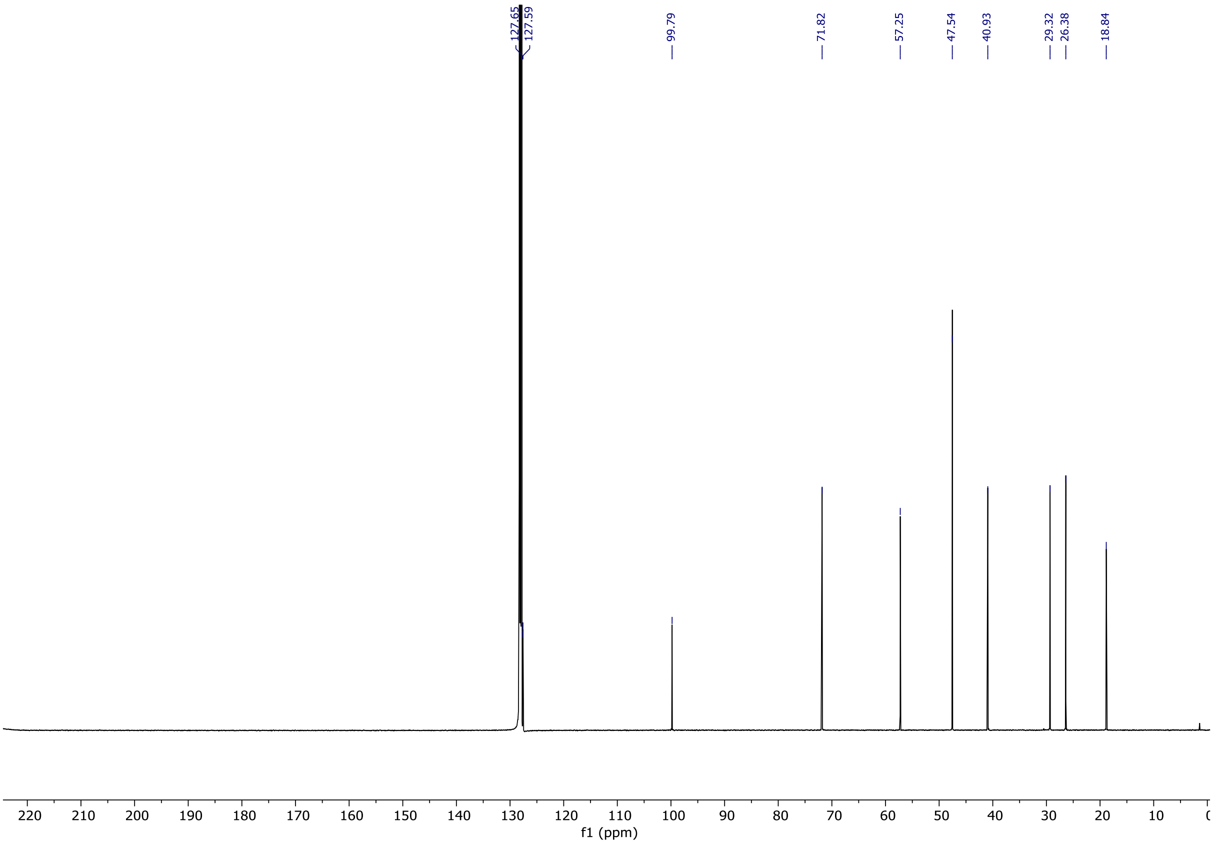

^{1}H NMR (500 MHz, Acetone-d_6) of S5

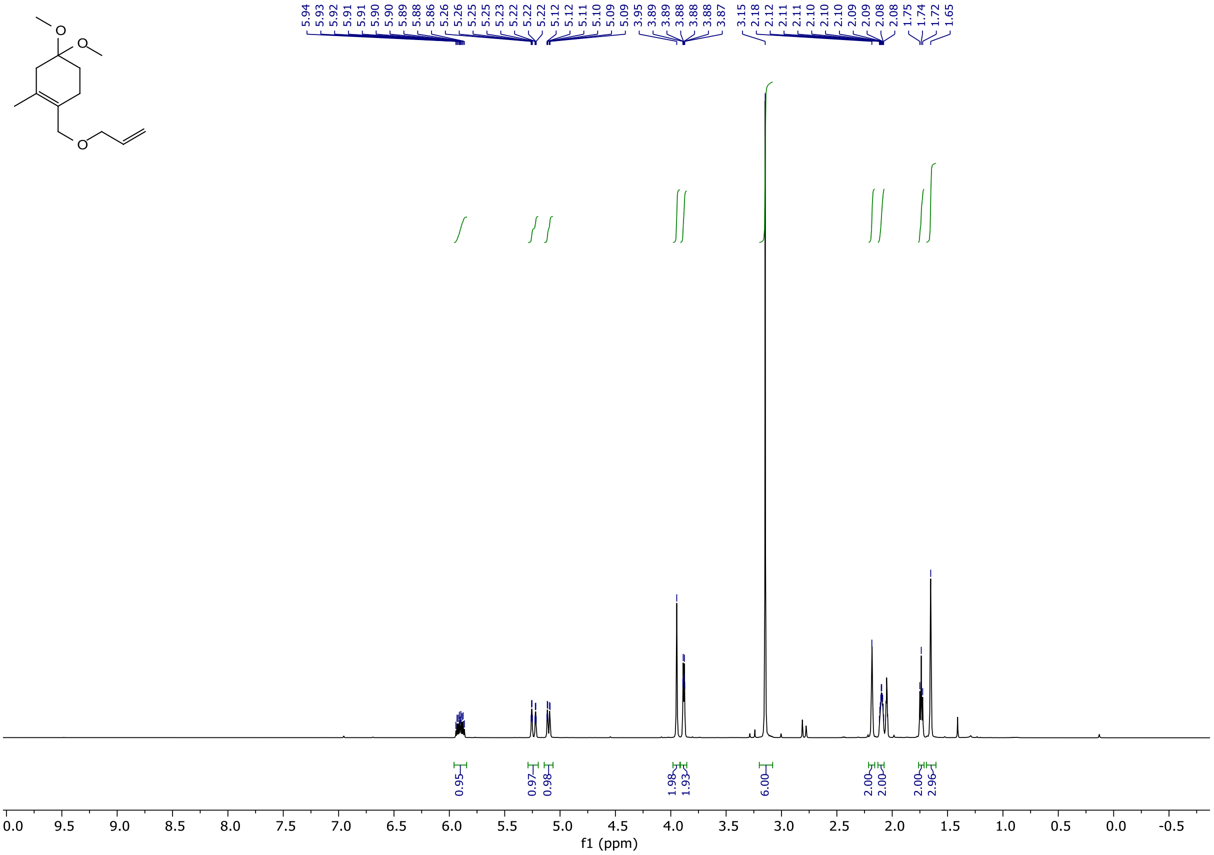

^{13}C NMR (126 MHz, Acetone-d_6) of S5

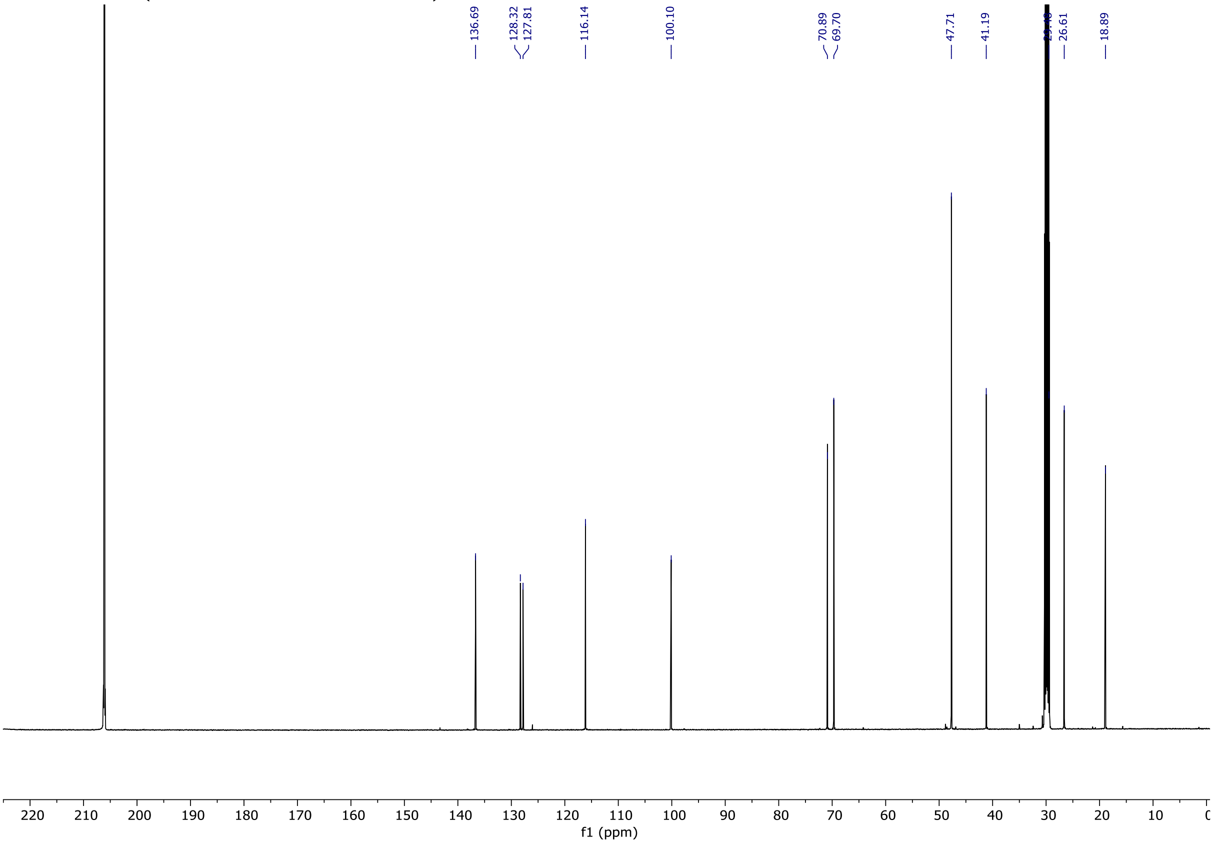

^{1}H NMR (600 MHz, Benzene-d_6) of **S6**

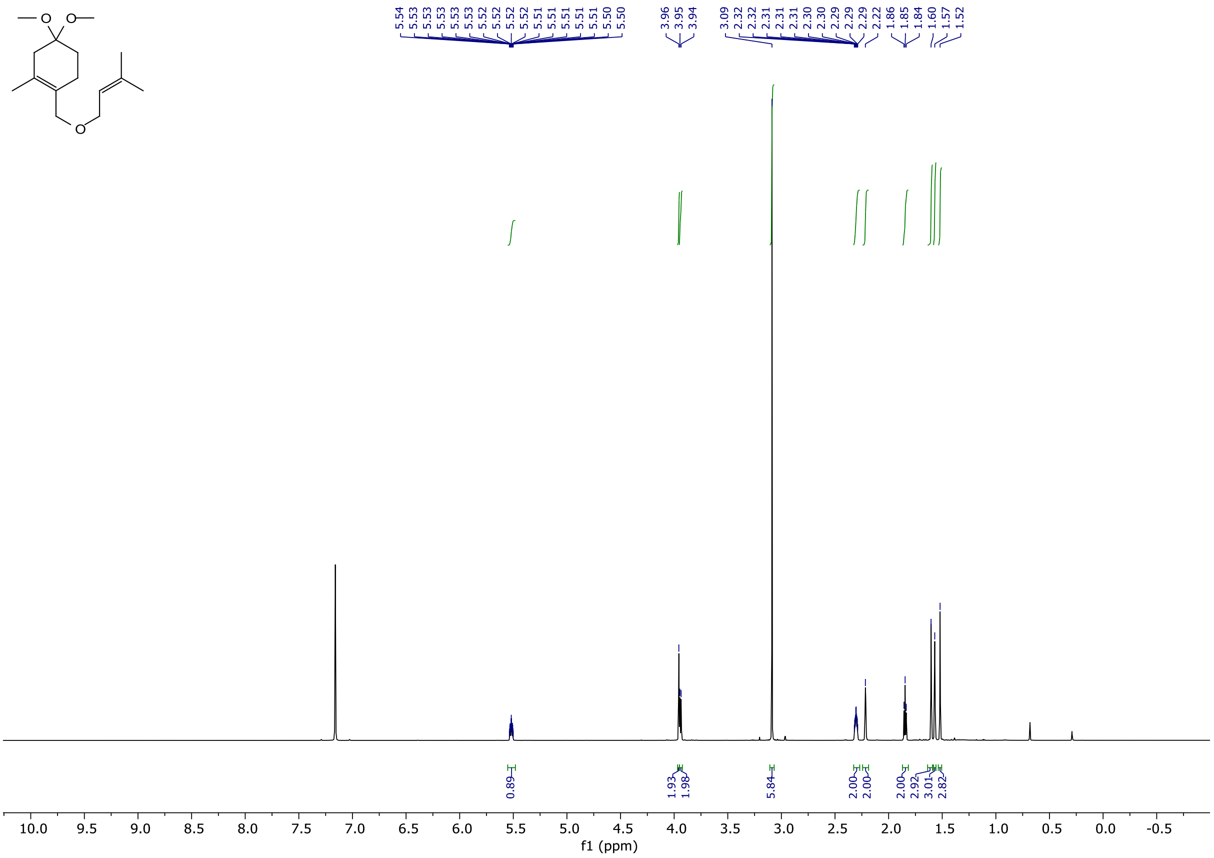

^{13}C NMR (151 MHz, Benzene-d_6) of **S6**

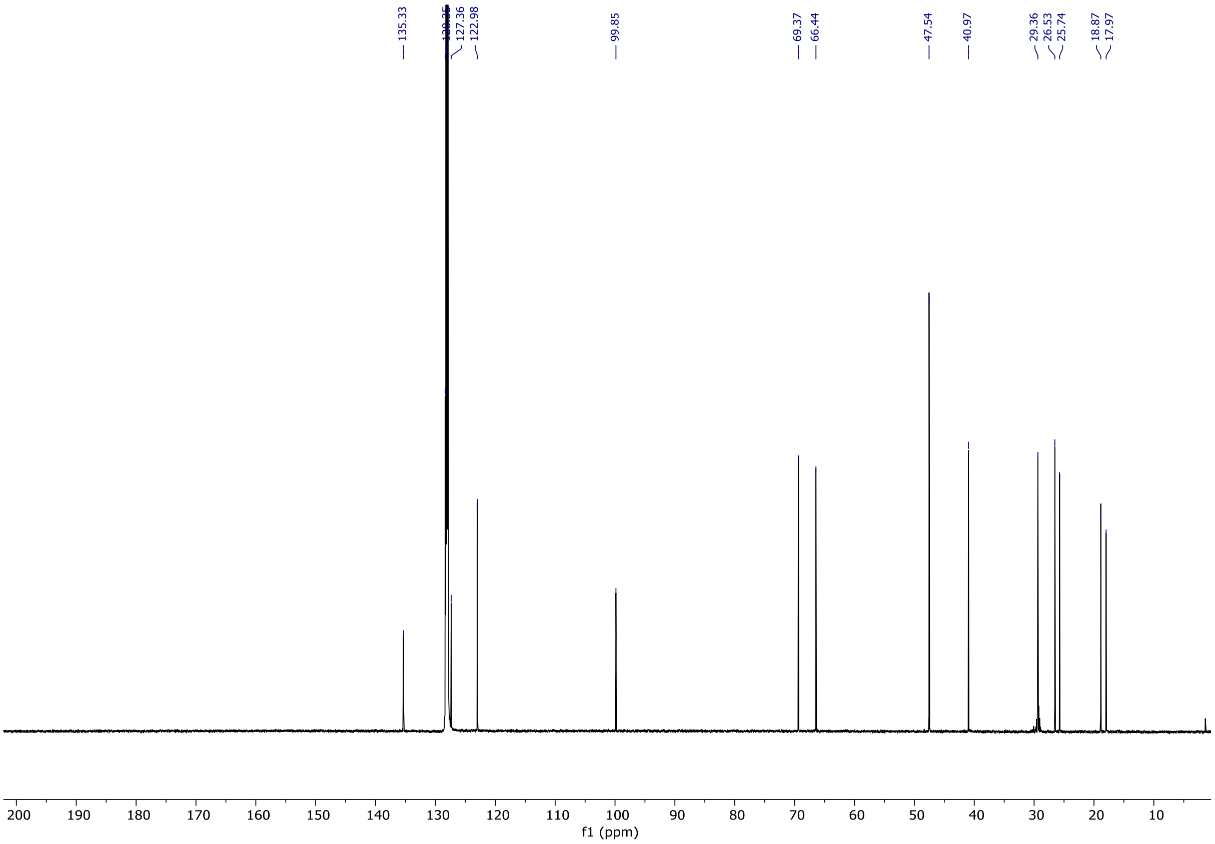

^{1}H NMR (500 MHz, Acetone-d_6) of **S7**

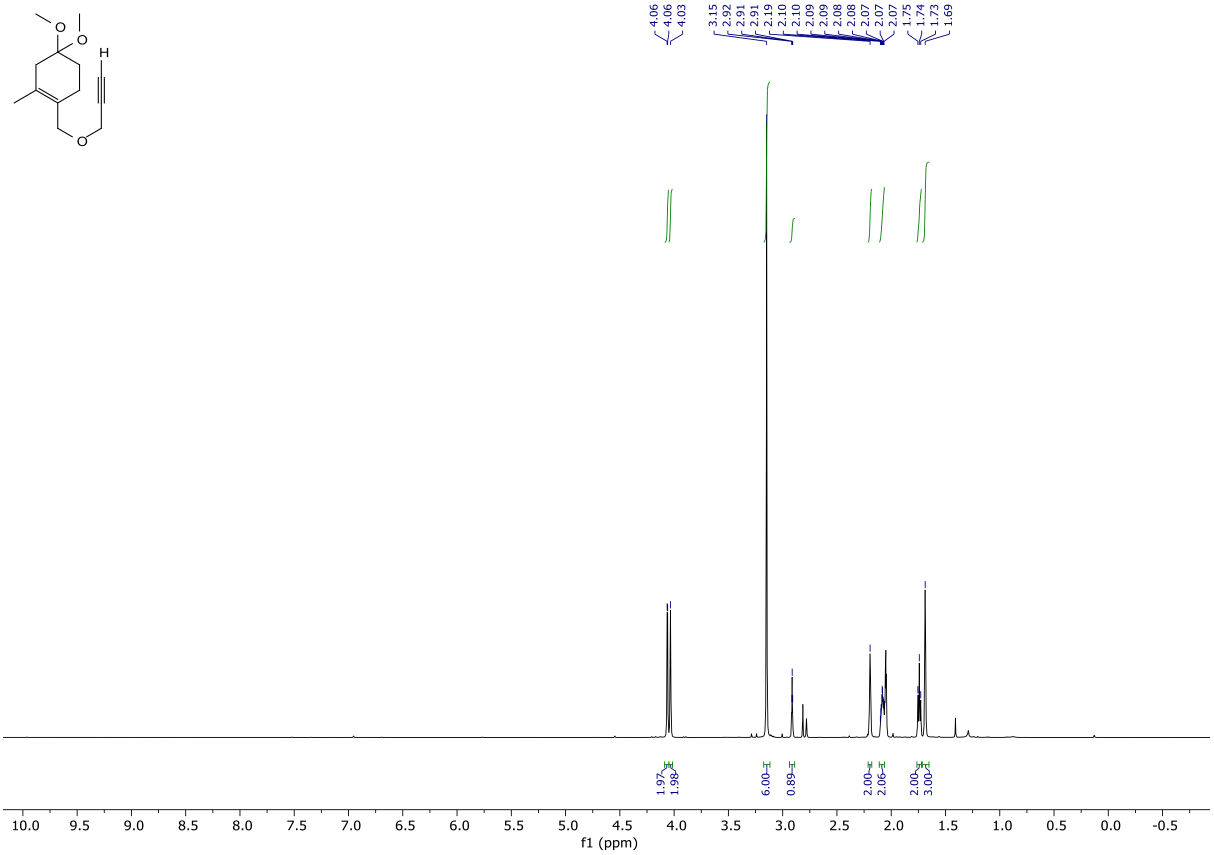

^{13}C NMR (126 MHz, Acetone-d_6) of **S7**

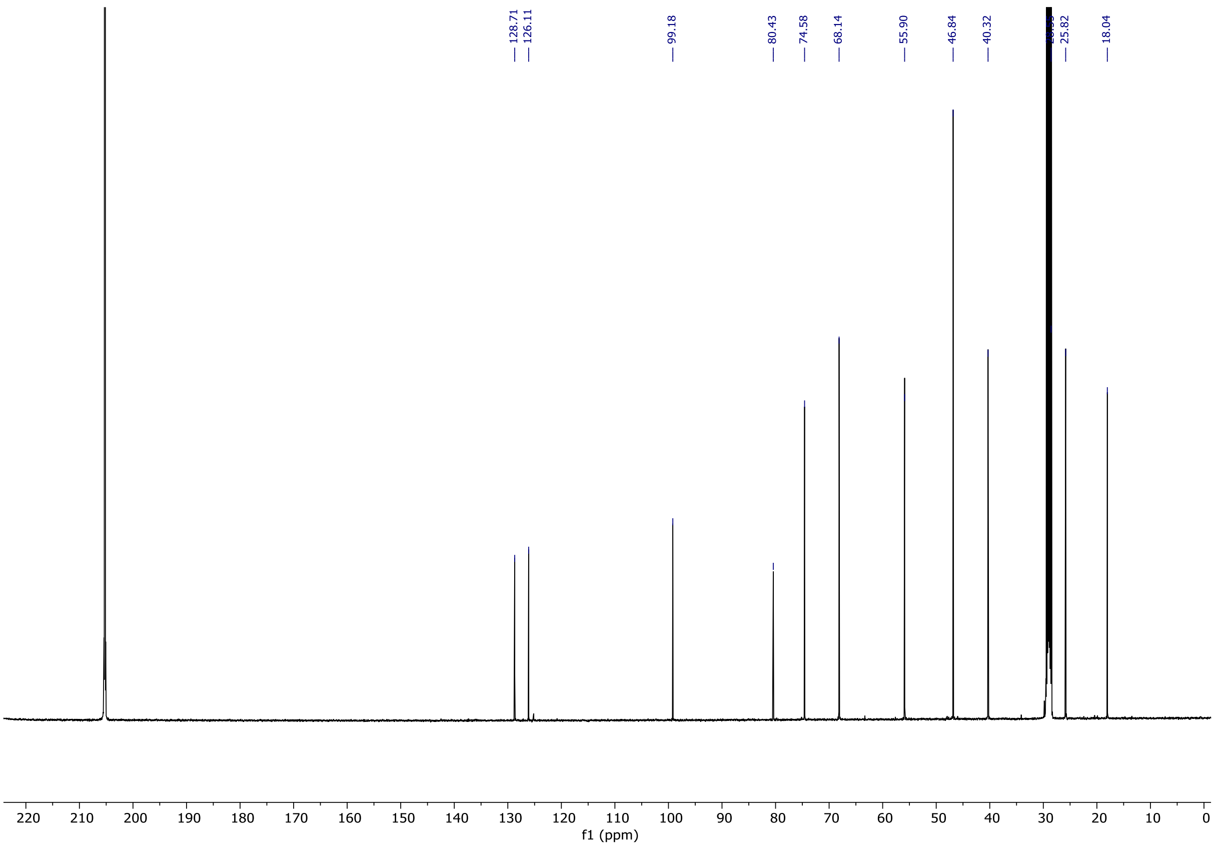

^{1}H NMR (500 MHz, Acetone-d_6) of **S8**

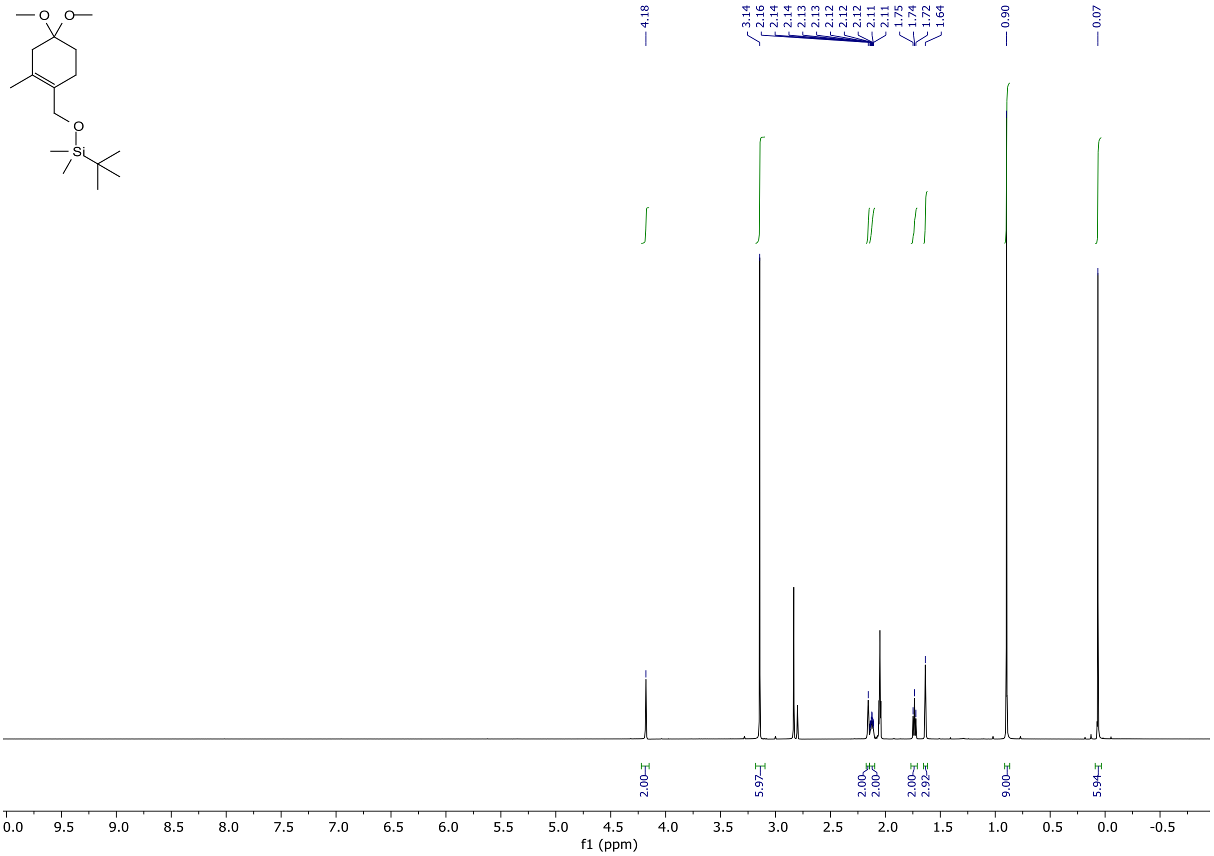

^{13}C NMR (126 MHz, Acetone-d_6) of **S8**

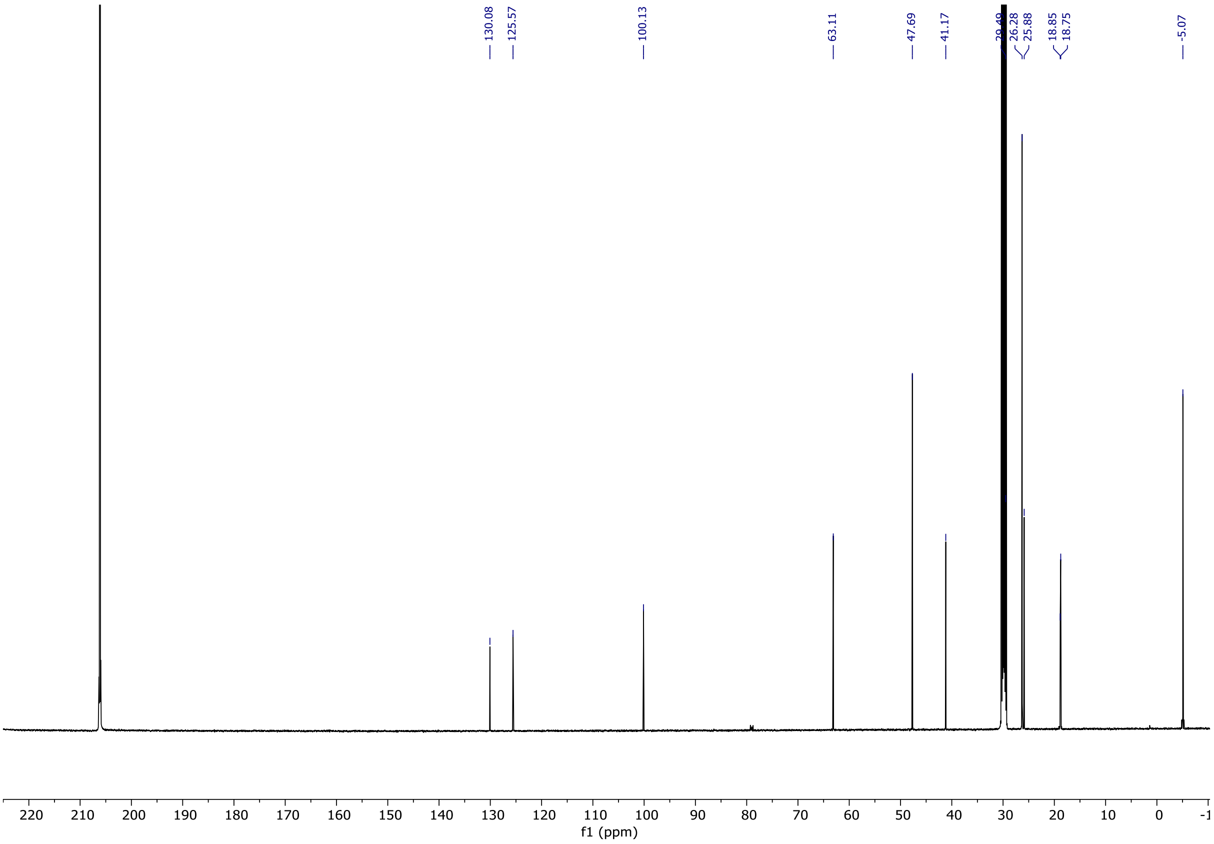

^{1}H NMR (400 MHz, Benzene-d_6) of **S9**

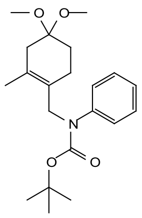

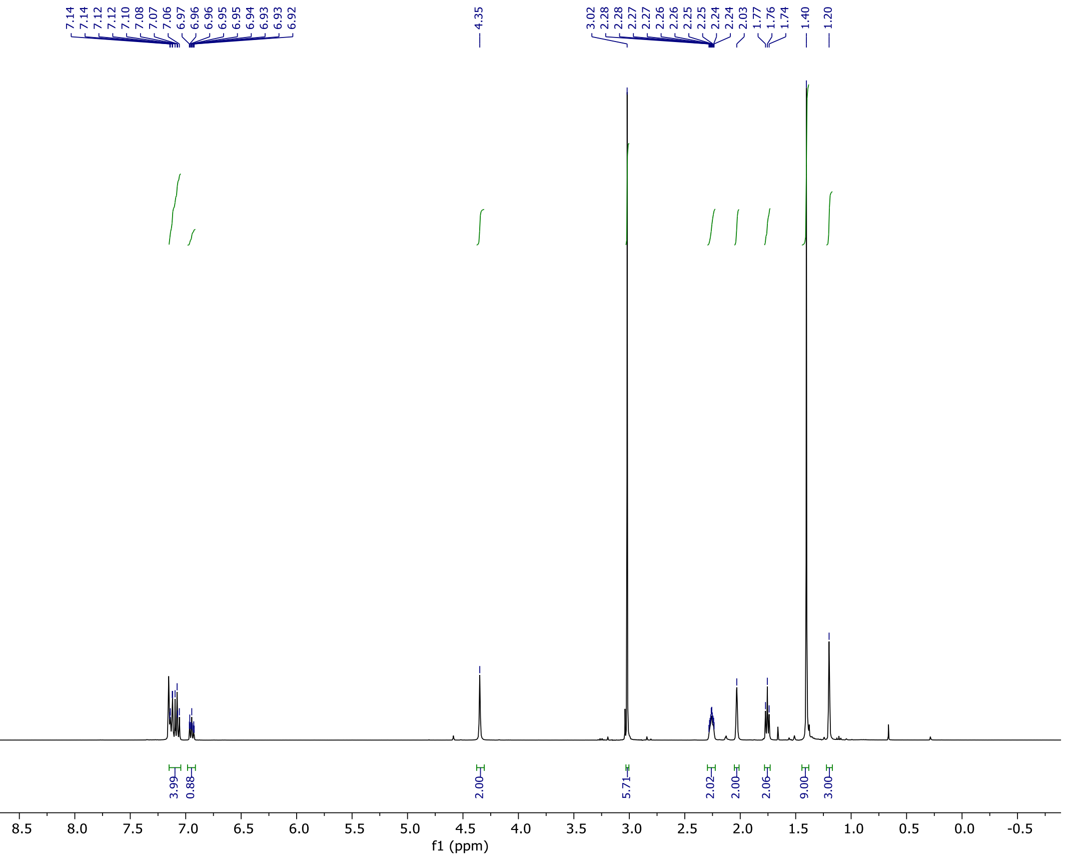

^{13}C NMR (101 MHz, Benzene-d_6) of **S9**

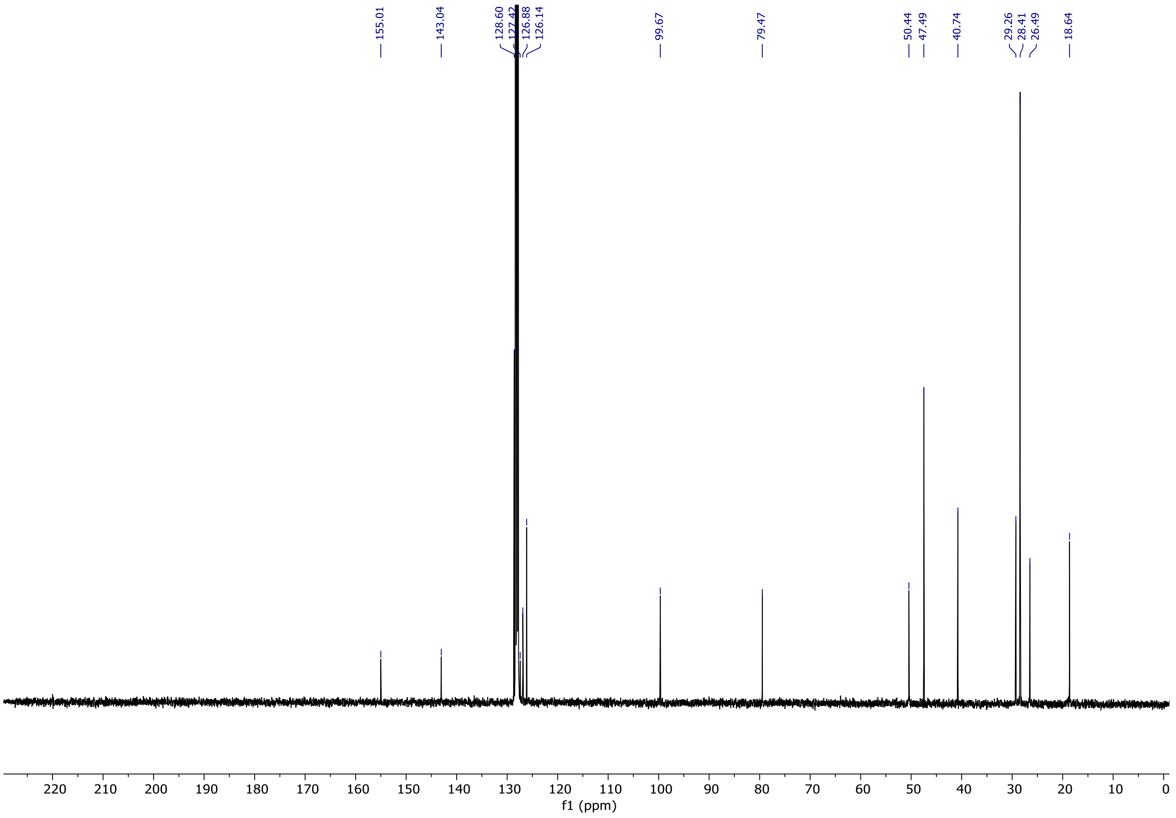

^{1}H NMR (500 MHz, Acetone-d_6) of **S10**

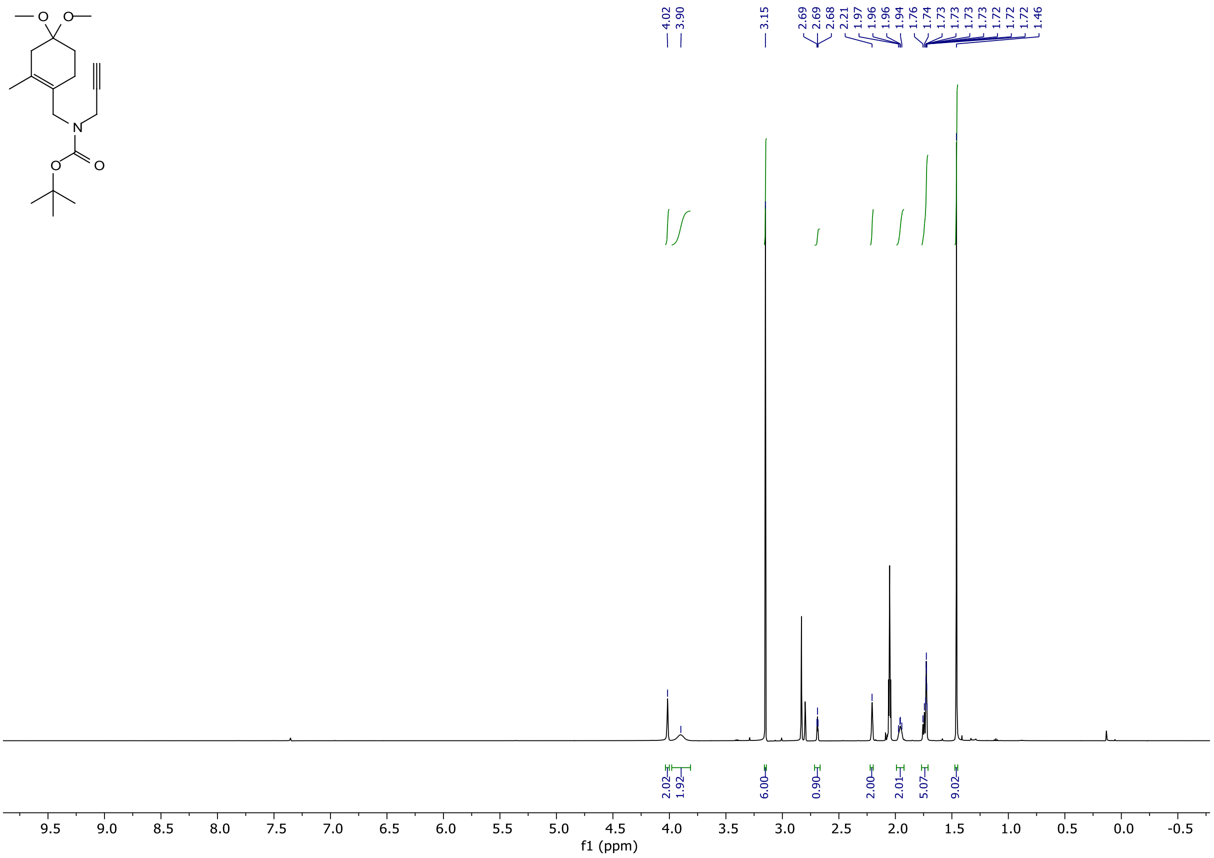

^{13}C NMR (126 MHz, Acetone-d_6) of **S10**

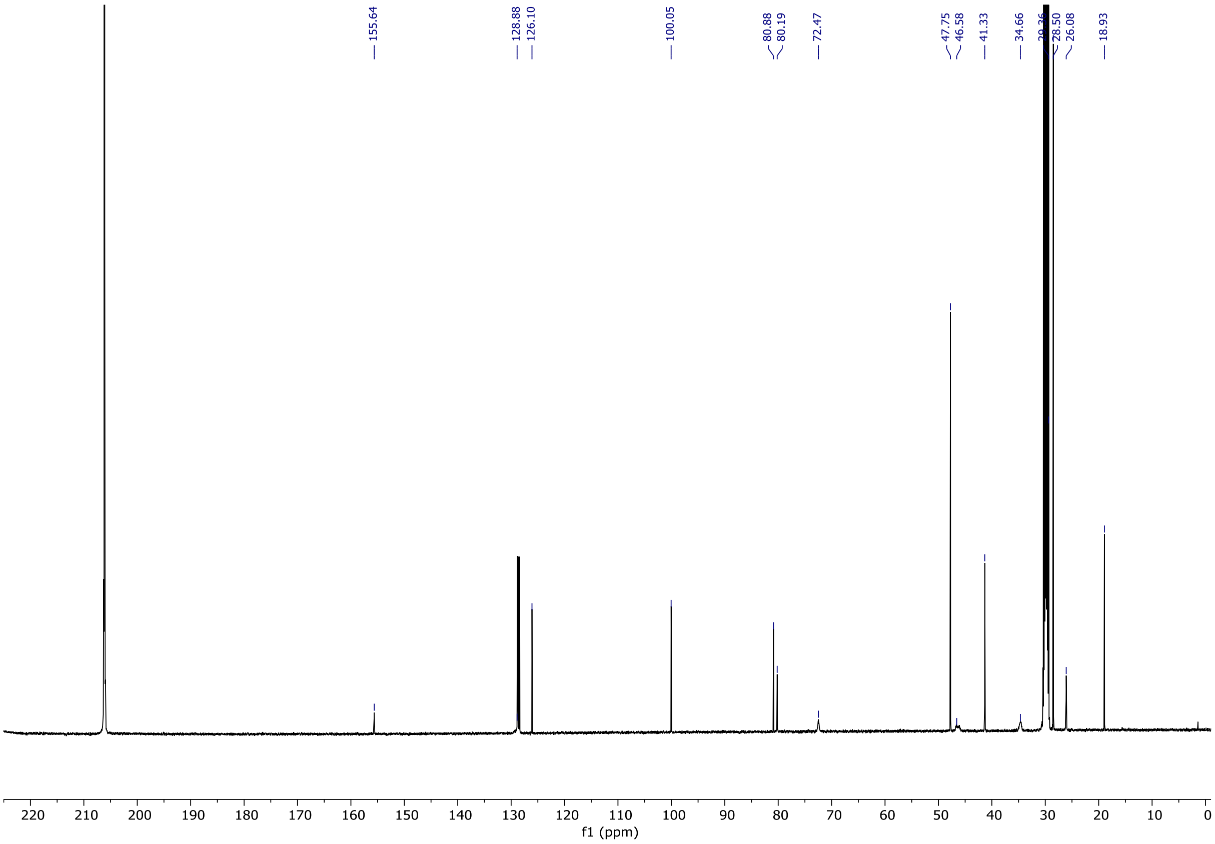

^{1}H NMR (400 MHz, Acetone-d_6) of **S12**

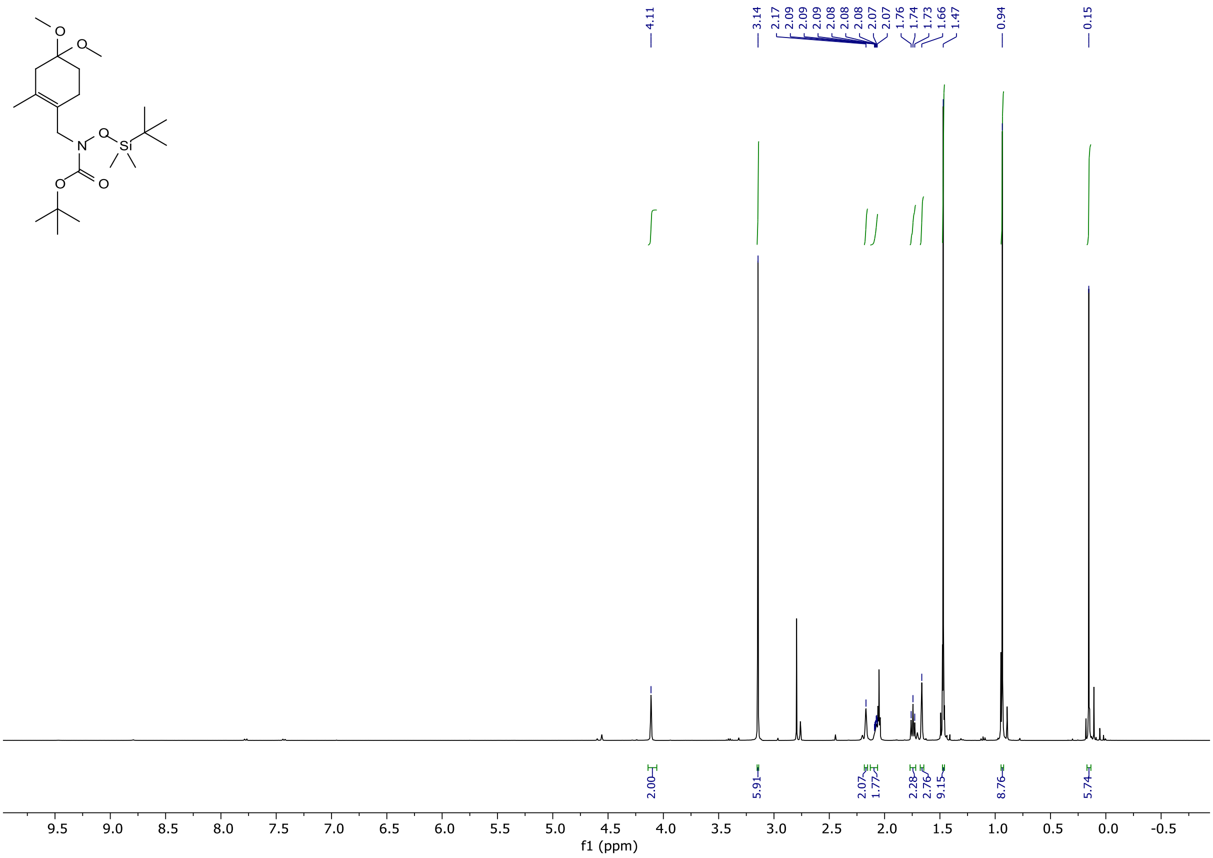

^{13}C NMR (126 MHz, Acetone-d_6) of **S12**

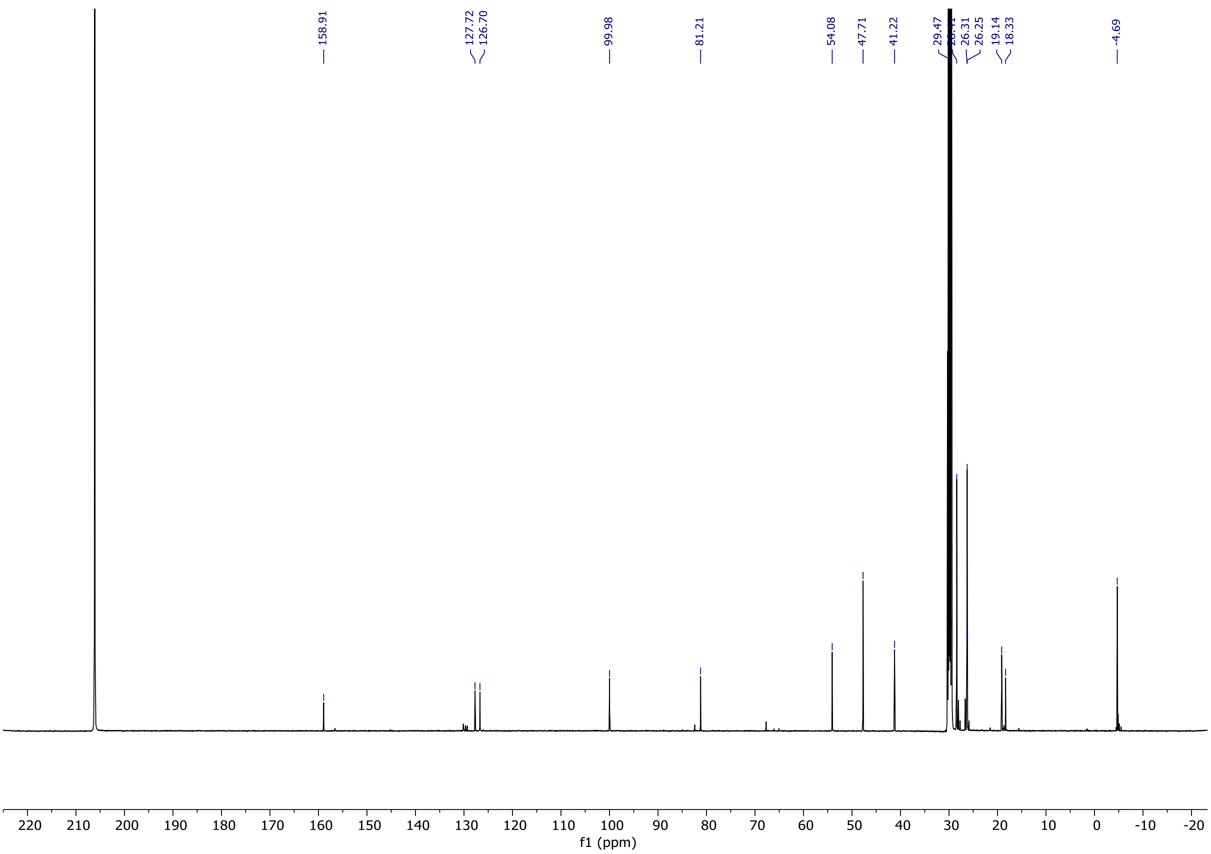

^{1}H NMR (500 MHz, Acetone-d_6) of **S13**

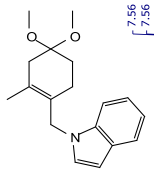

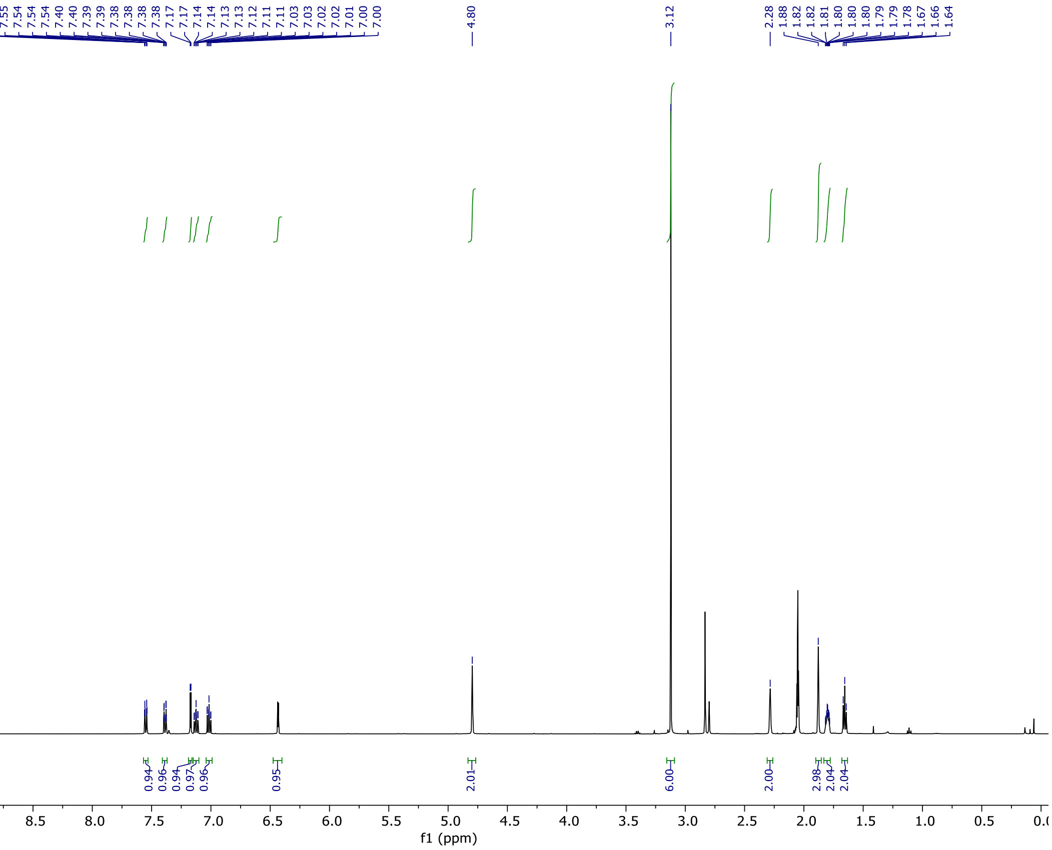

^{13}C NMR (126 MHz, Acetone-d_6) of **S13**

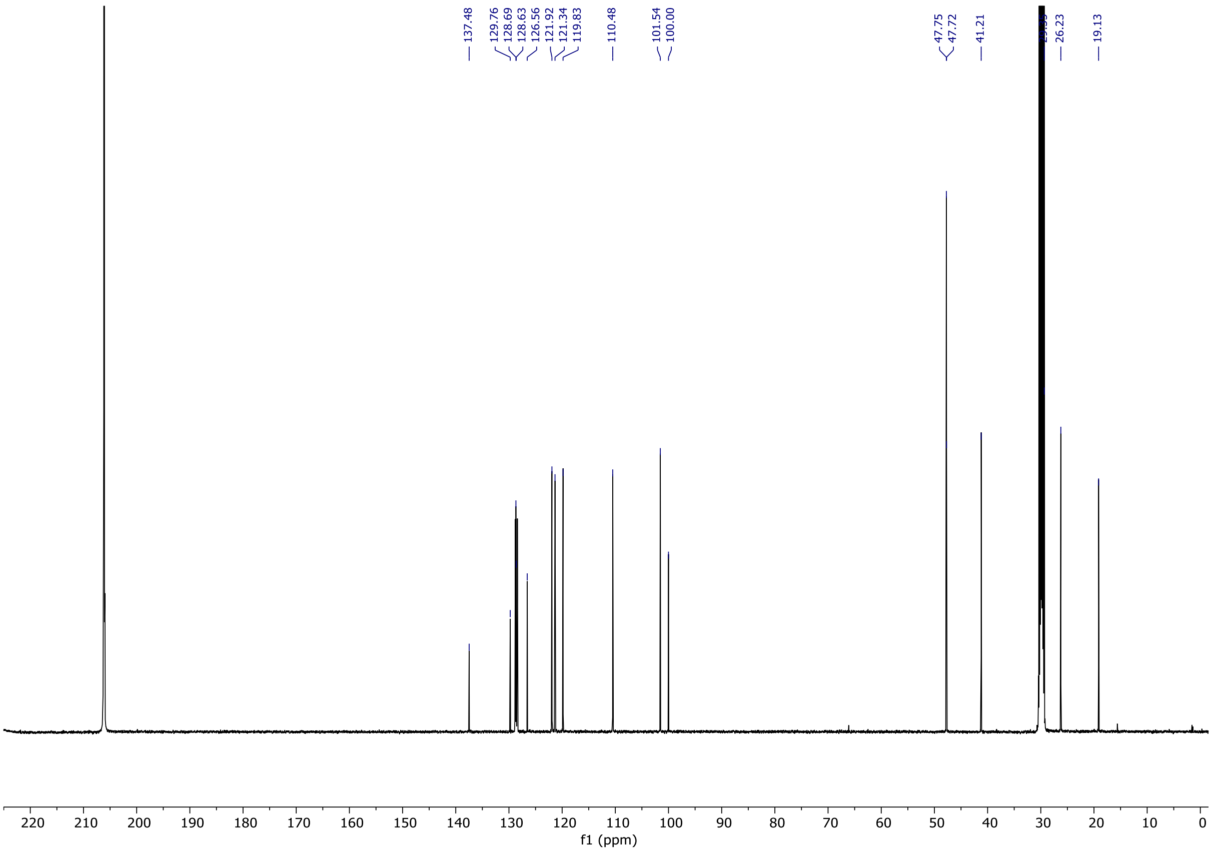

^{1}H NMR (400 MHz, Benzene-d_6) of **S14**

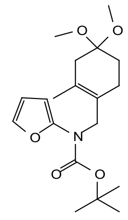

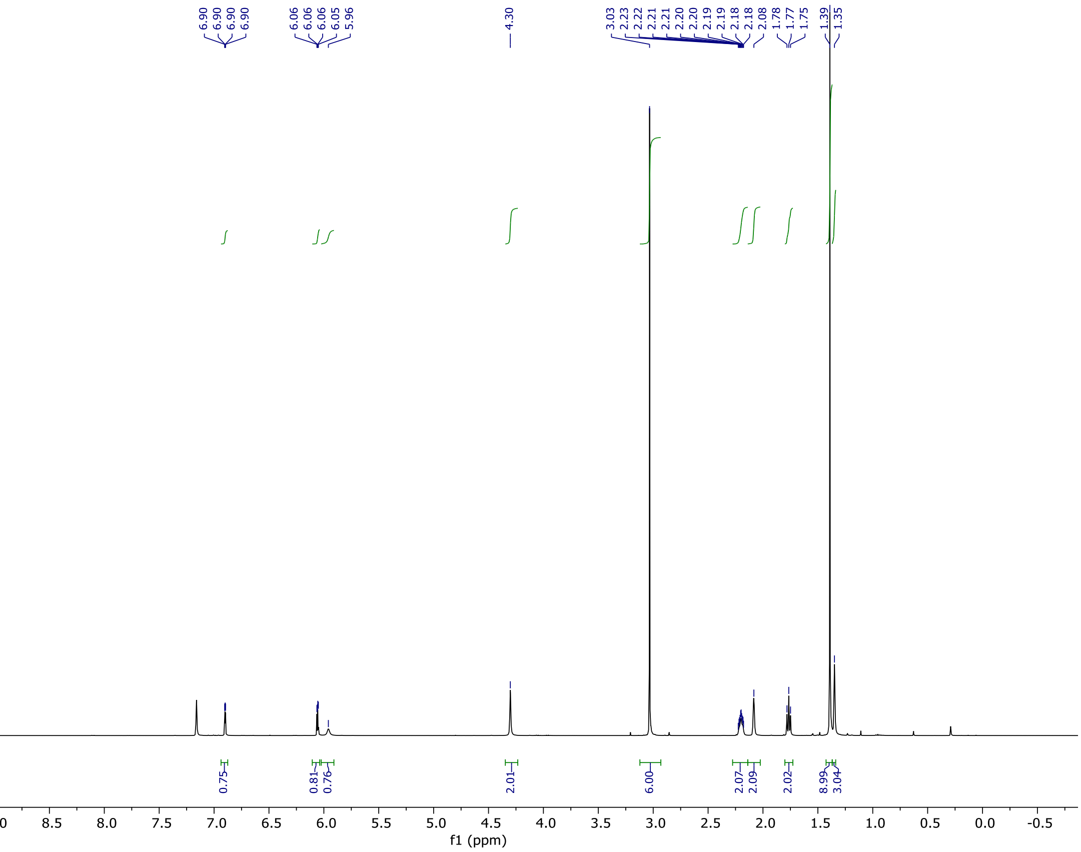

^{13}C NMR (101 MHz, Benzene-d_6) of **S14**

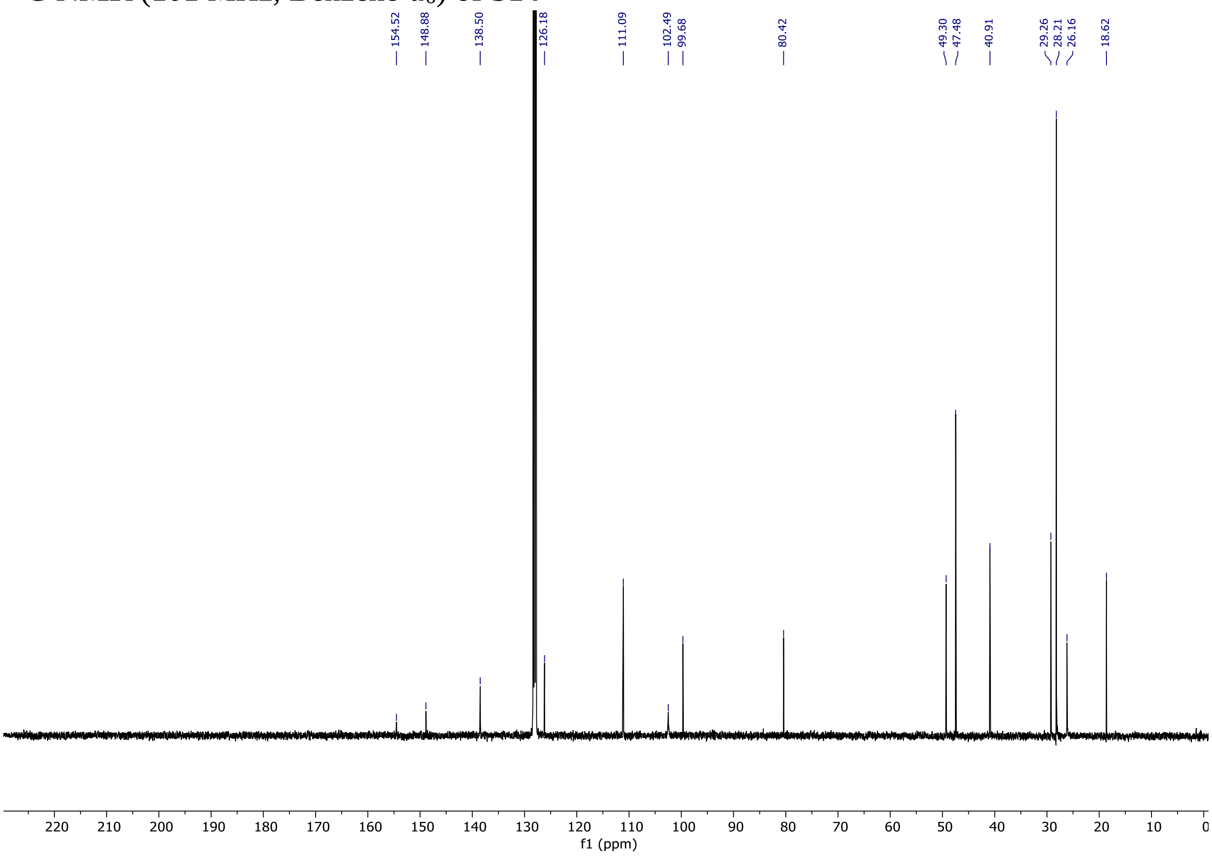

^{1}H NMR (500 MHz, Acetone-d_6) of **S15**

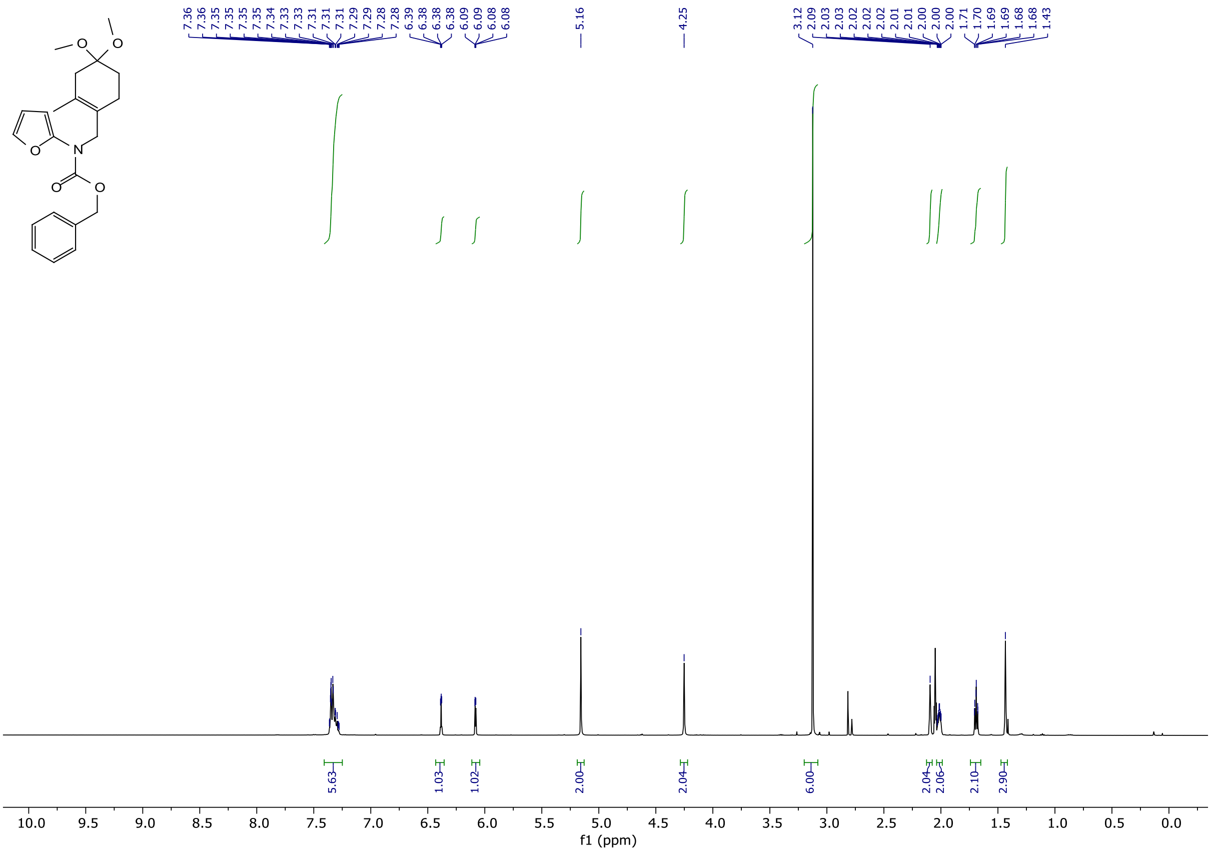

^{13}C NMR (126 MHz, Acetone-d_6) of **S15**

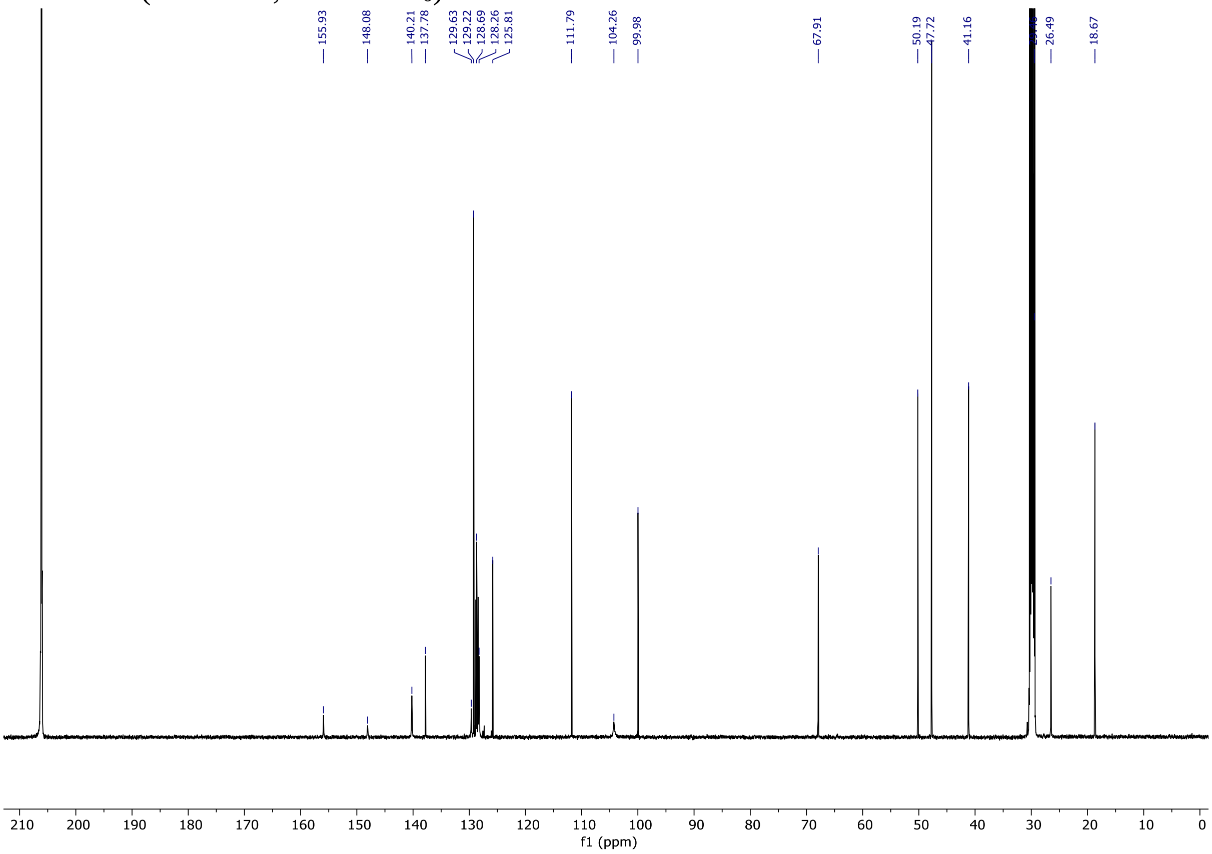

^{1}H NMR (400 MHz, $CDCl_3$) of **1a**

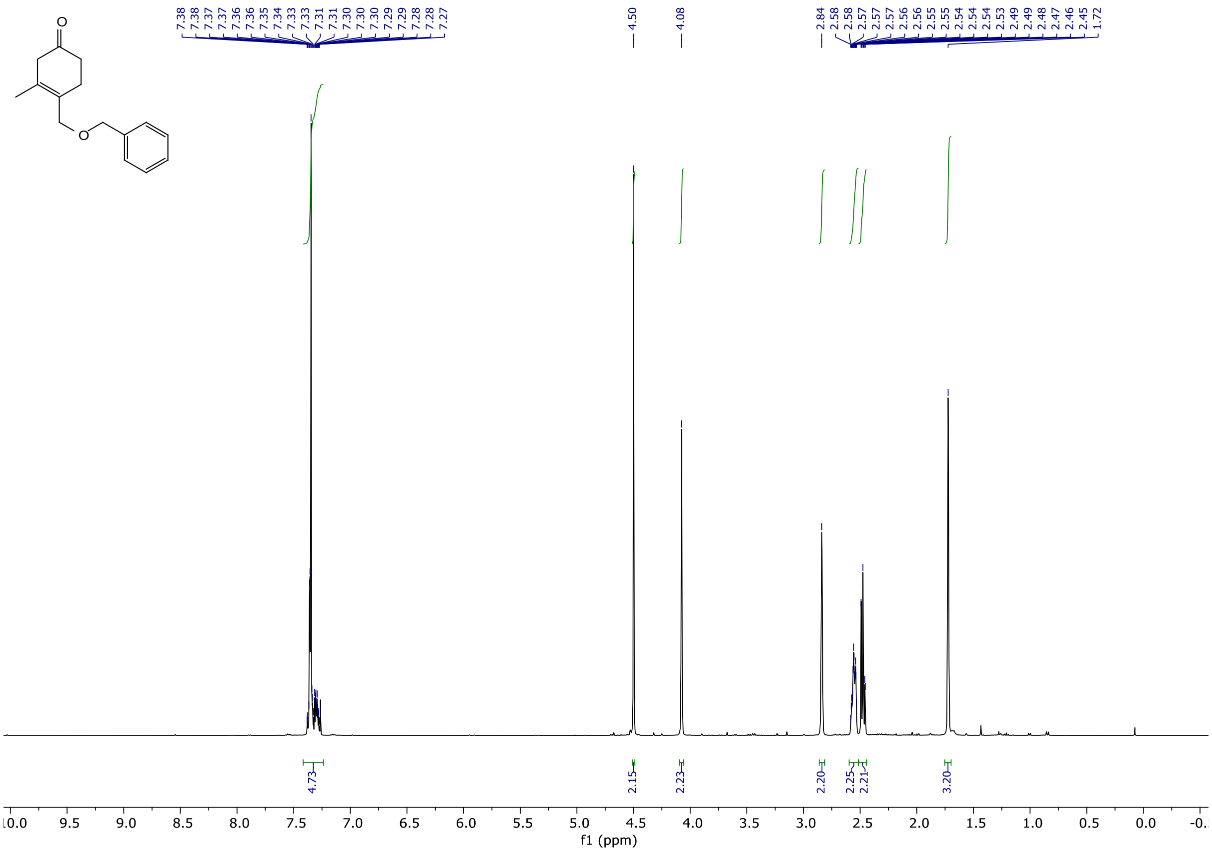

^{13}C NMR (101 MHz, $CDCl_3$) of **1a**

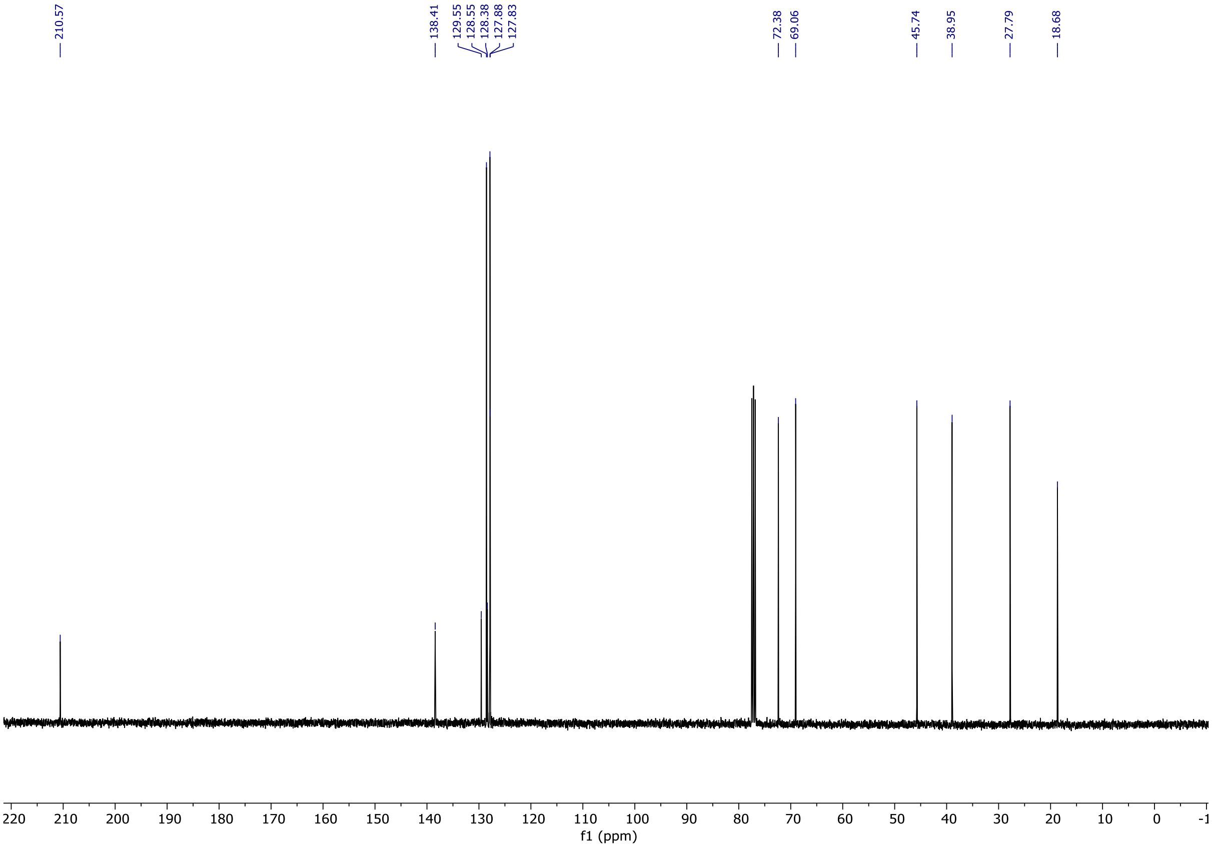

^{1}H NMR (500 MHz, Benzene-d_6) of **1b**

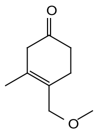

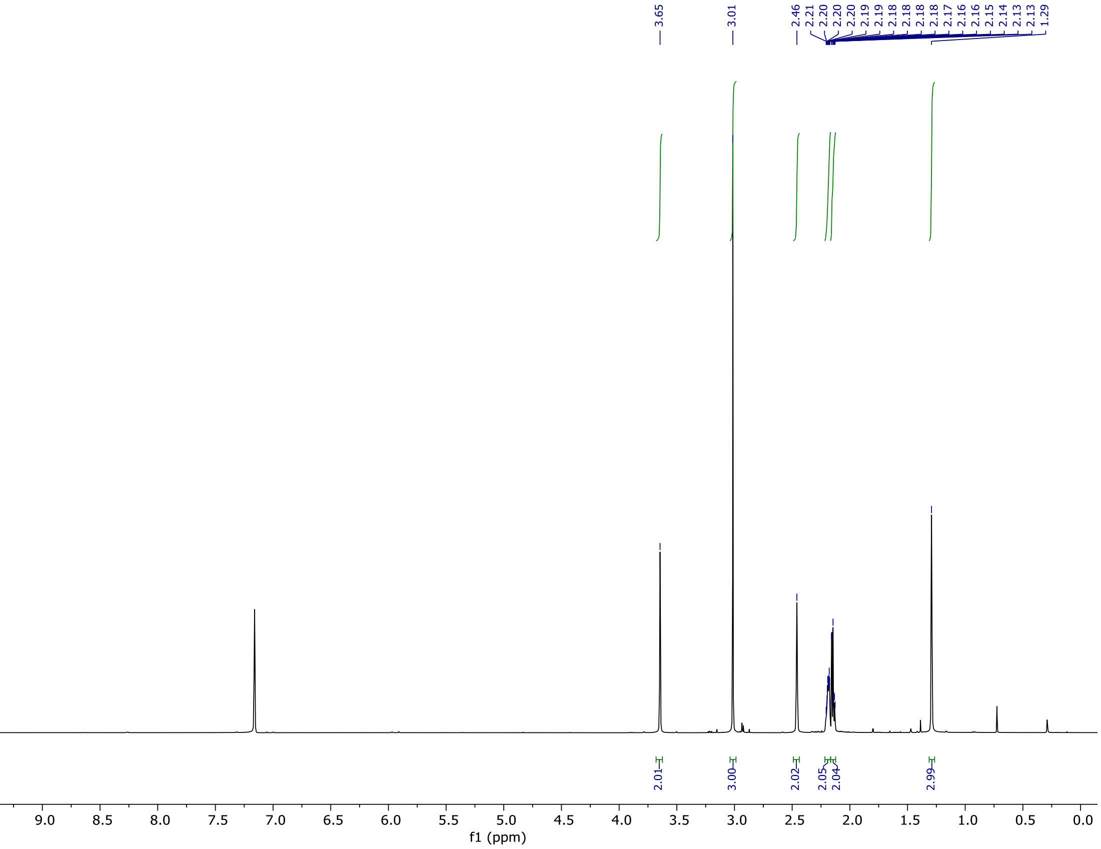

^{13}C NMR (126 MHz, Benzene-d_6) of **1b**

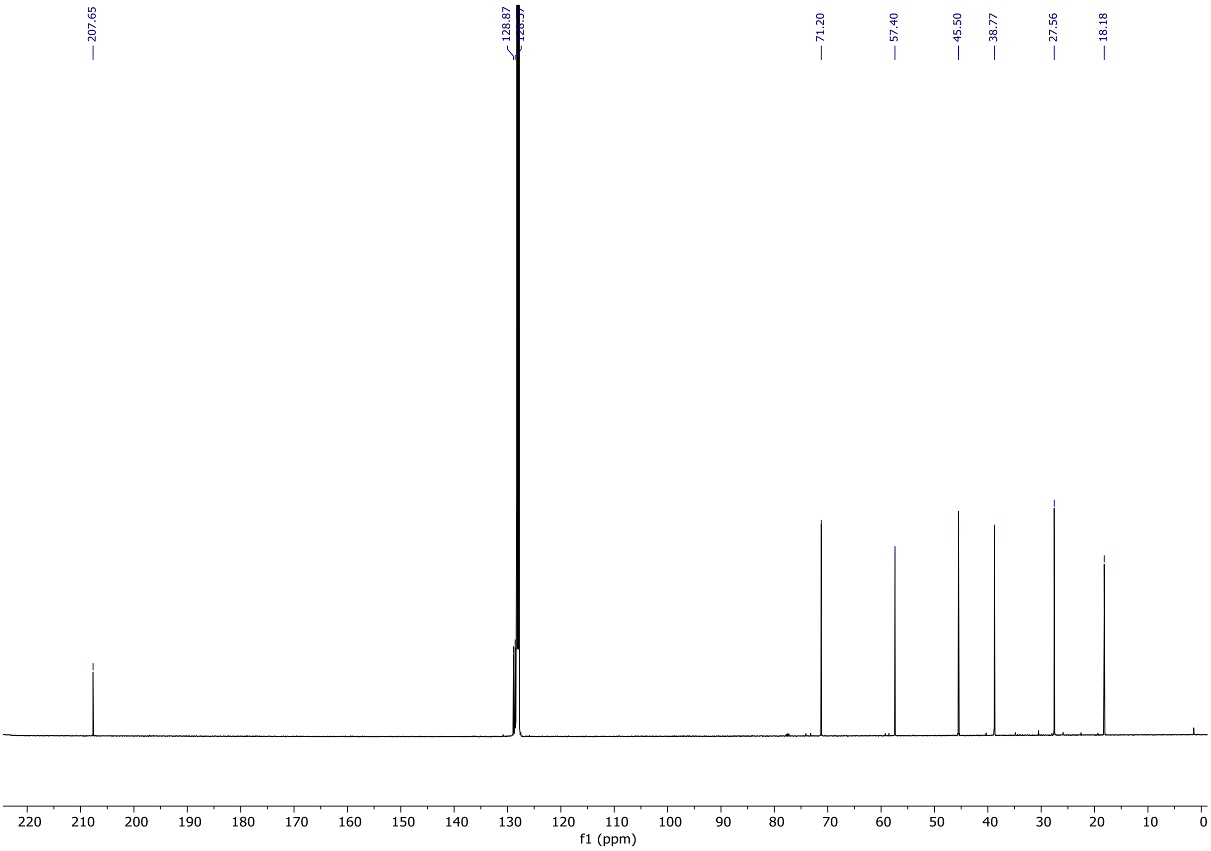

^{1}H NMR (400 MHz, Acetone-d_6) of **1c**

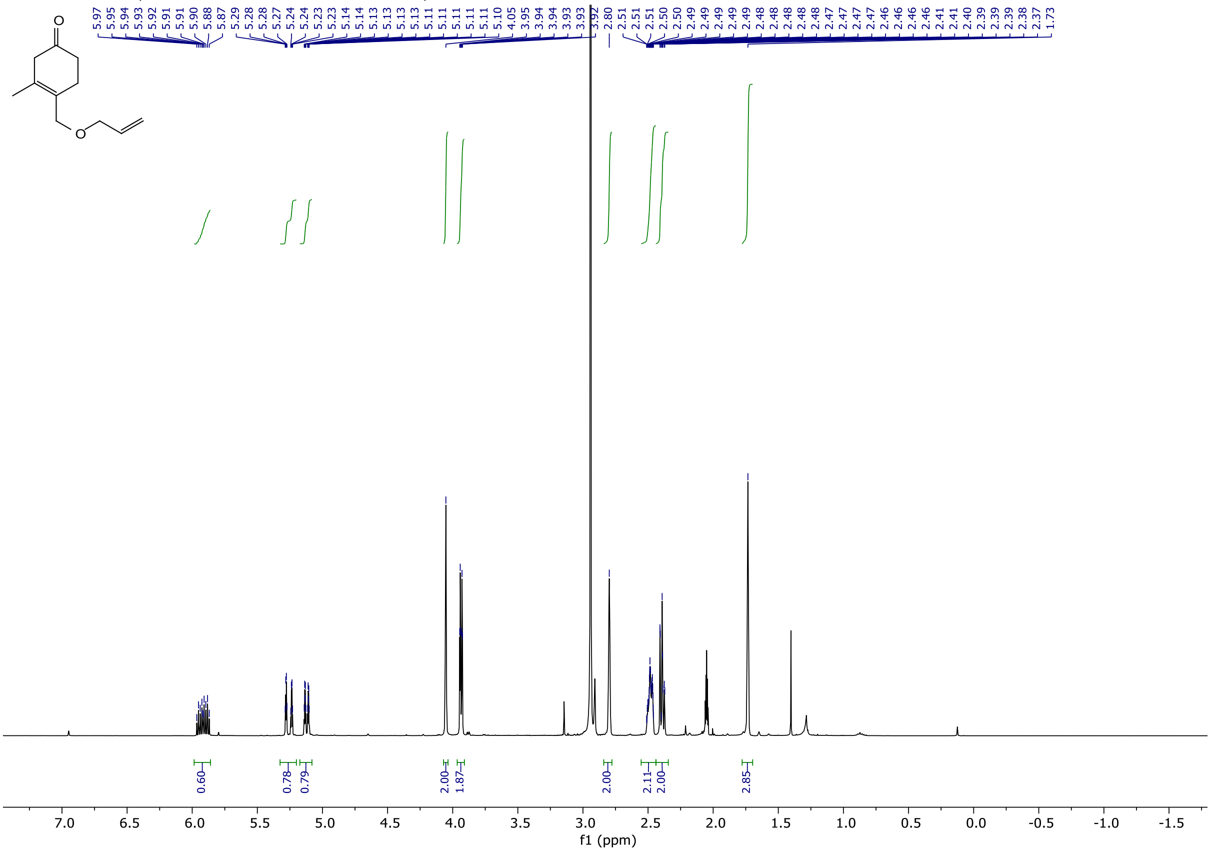

^{13}C NMR (101 MHz, Acetone-d_6) of **1c**

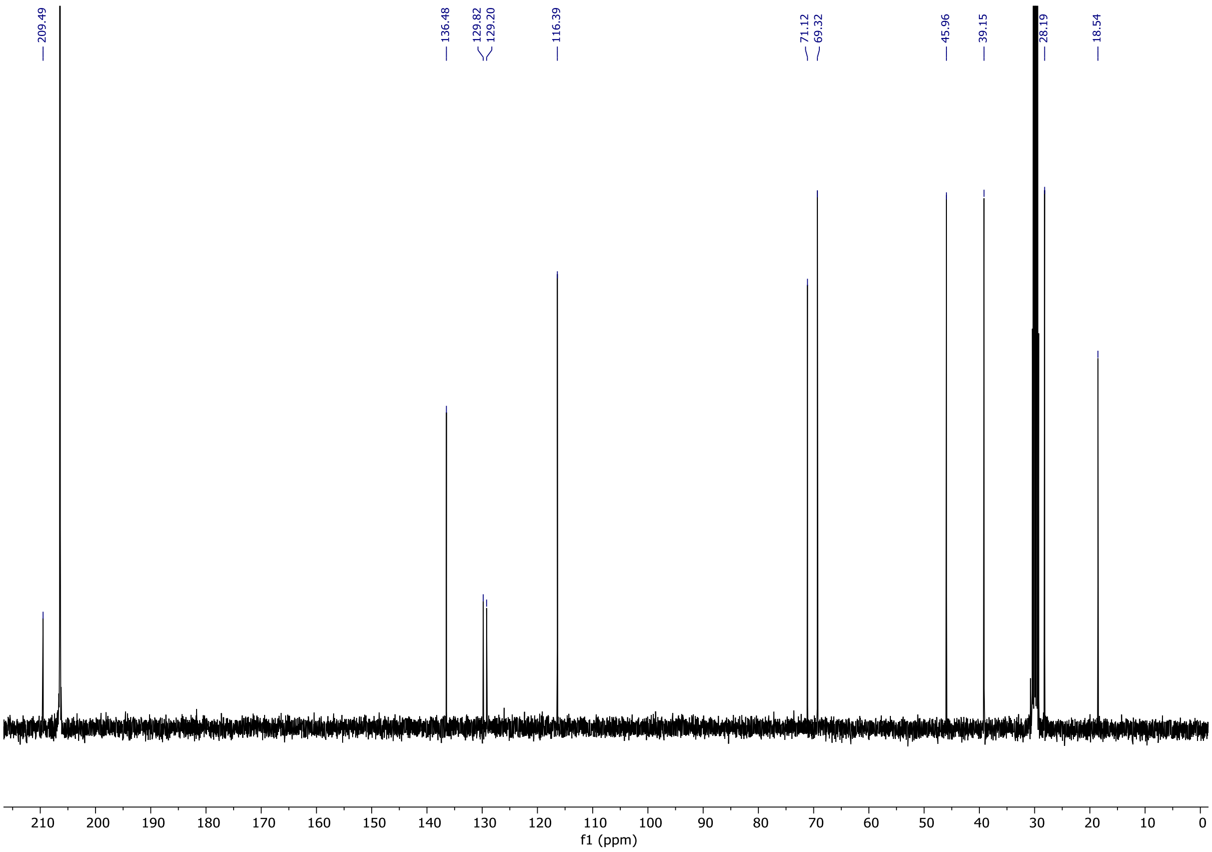

^{1}H NMR (500 MHz, Acetone-d_6) of **1d**

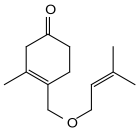

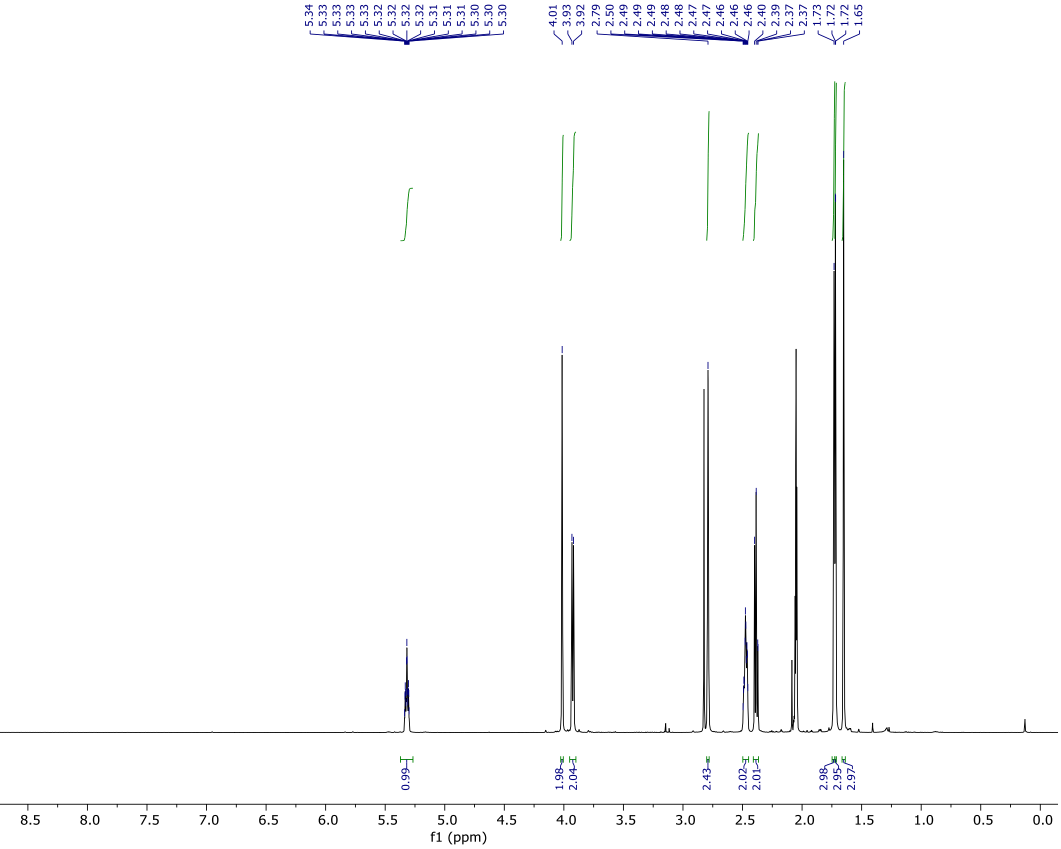

^{13}C NMR (126 MHz, Acetone-d_6) of **1d**

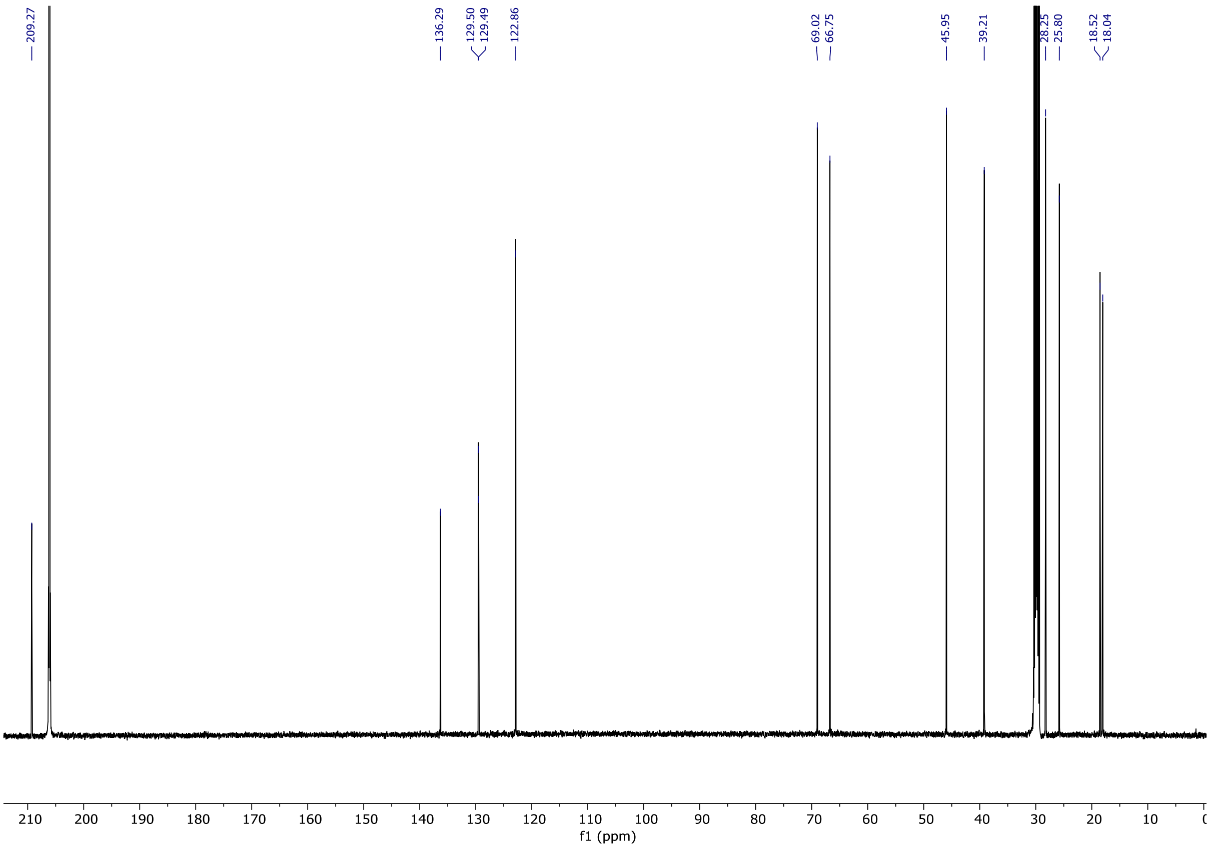

^{1}H NMR (500 MHz, Benzene-d_6) of **1e**

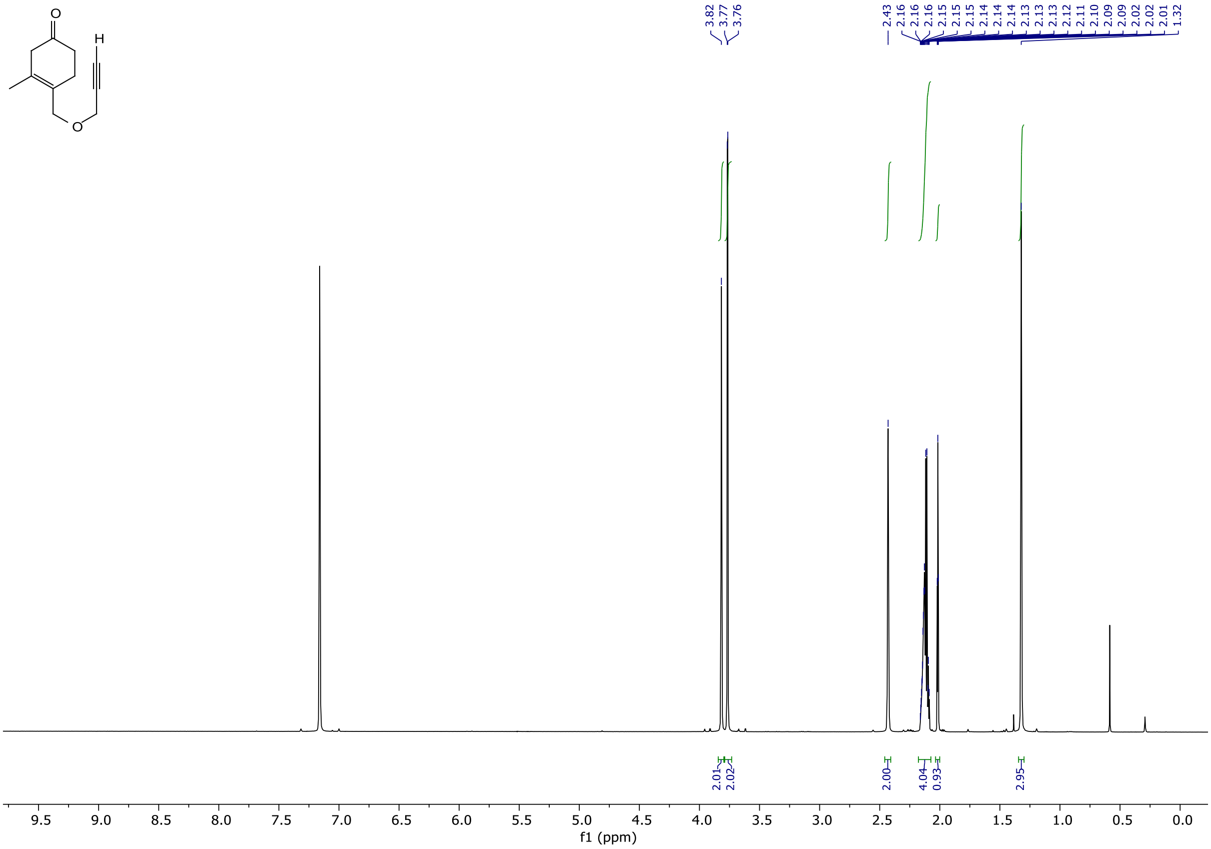

^{13}C NMR (126 MHz, Benzene-d_6) of **1e**

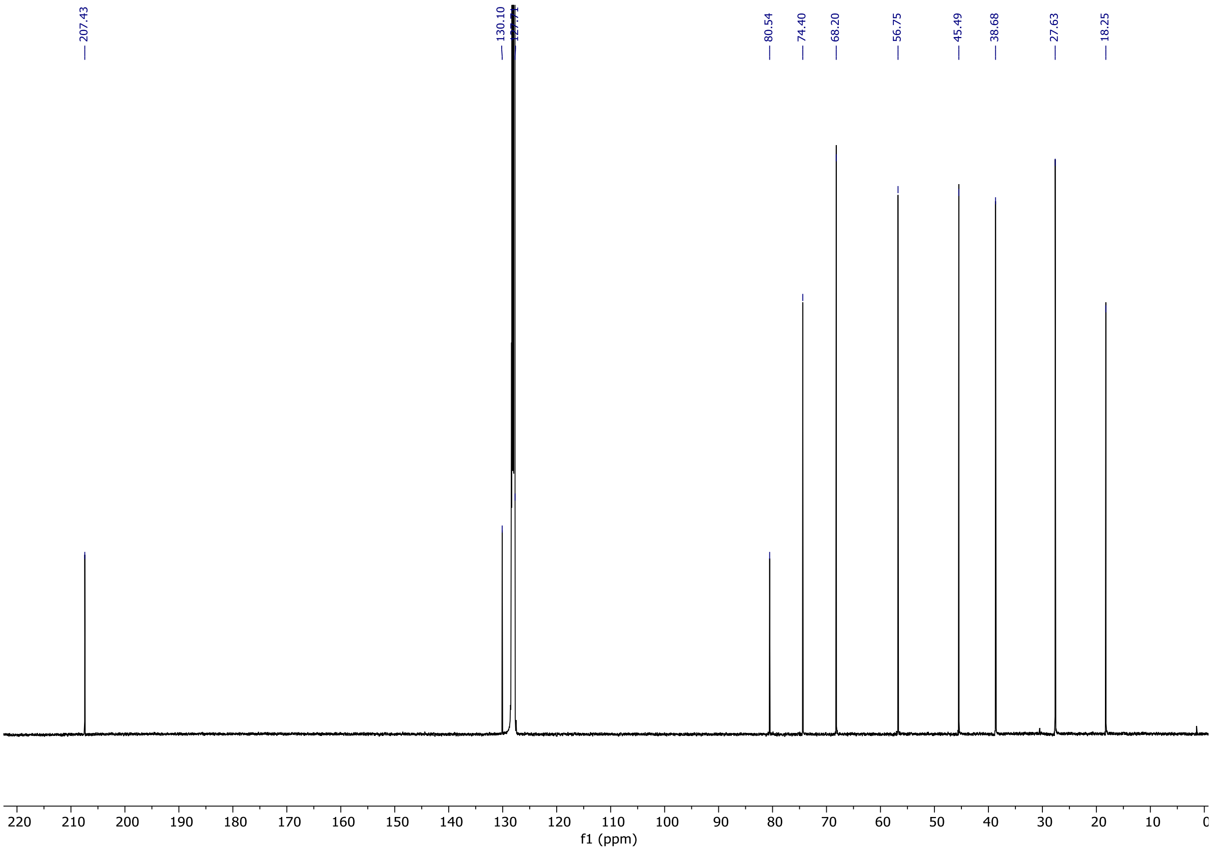

^{1}H NMR (500 MHz, Acetone-*d*$_6$) of **1f**

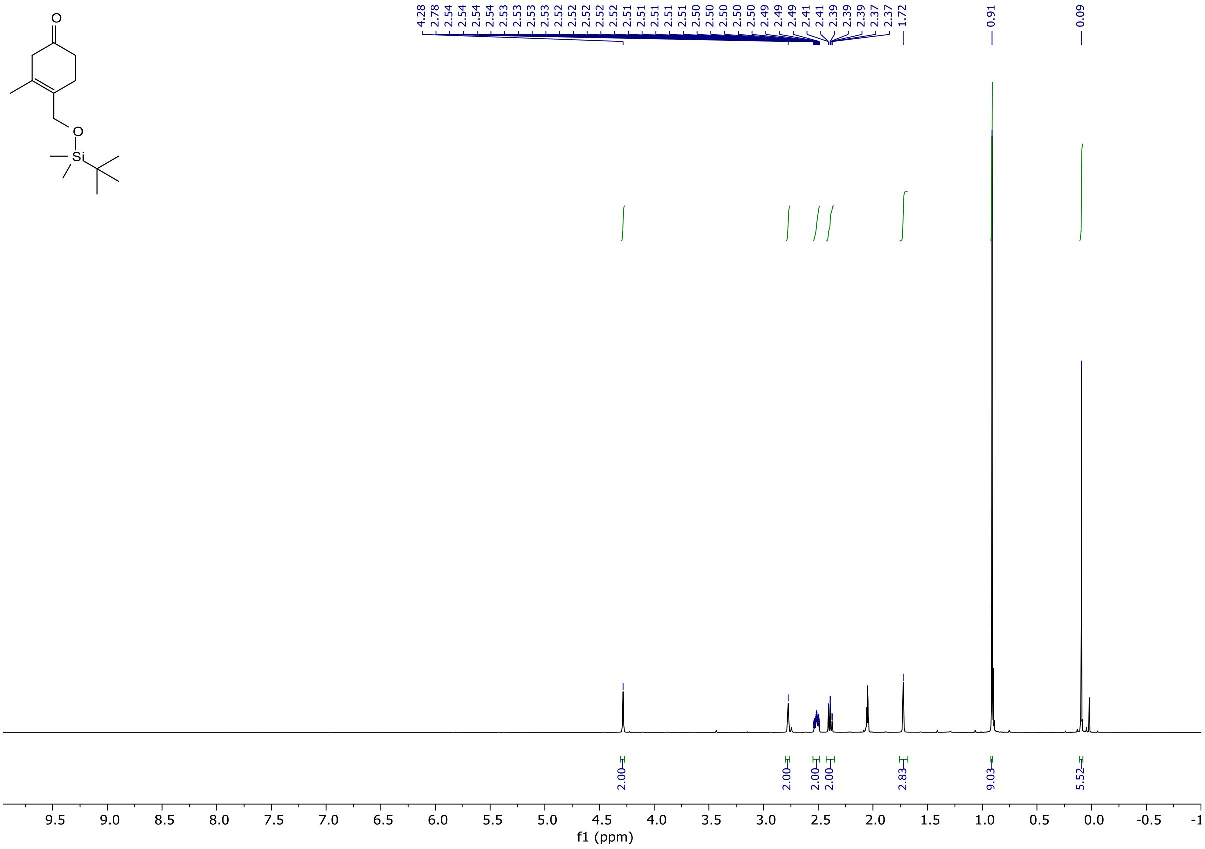

^{13}C NMR (126 MHz, Acetone-*d*$_6$) of **1f**

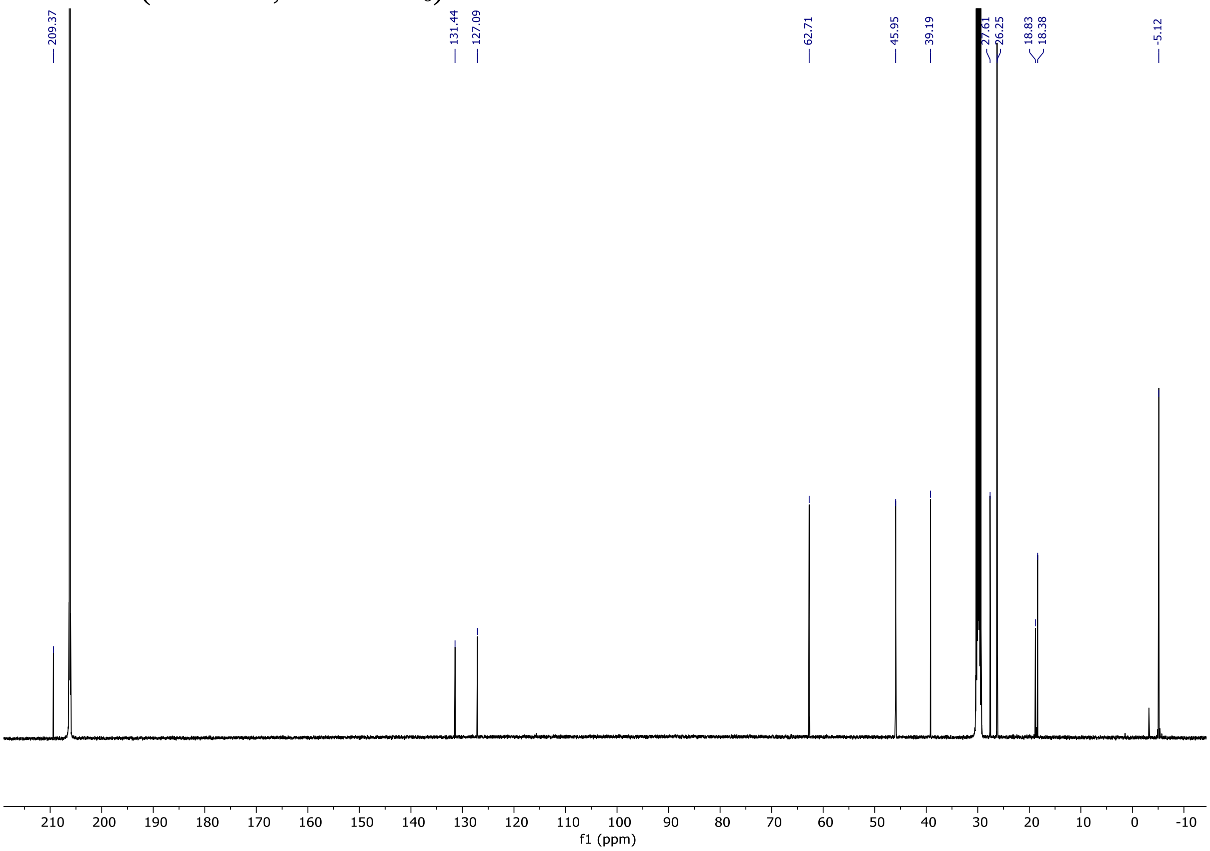

¹H NMR (400 MHz, CDCl₃) of **1g**

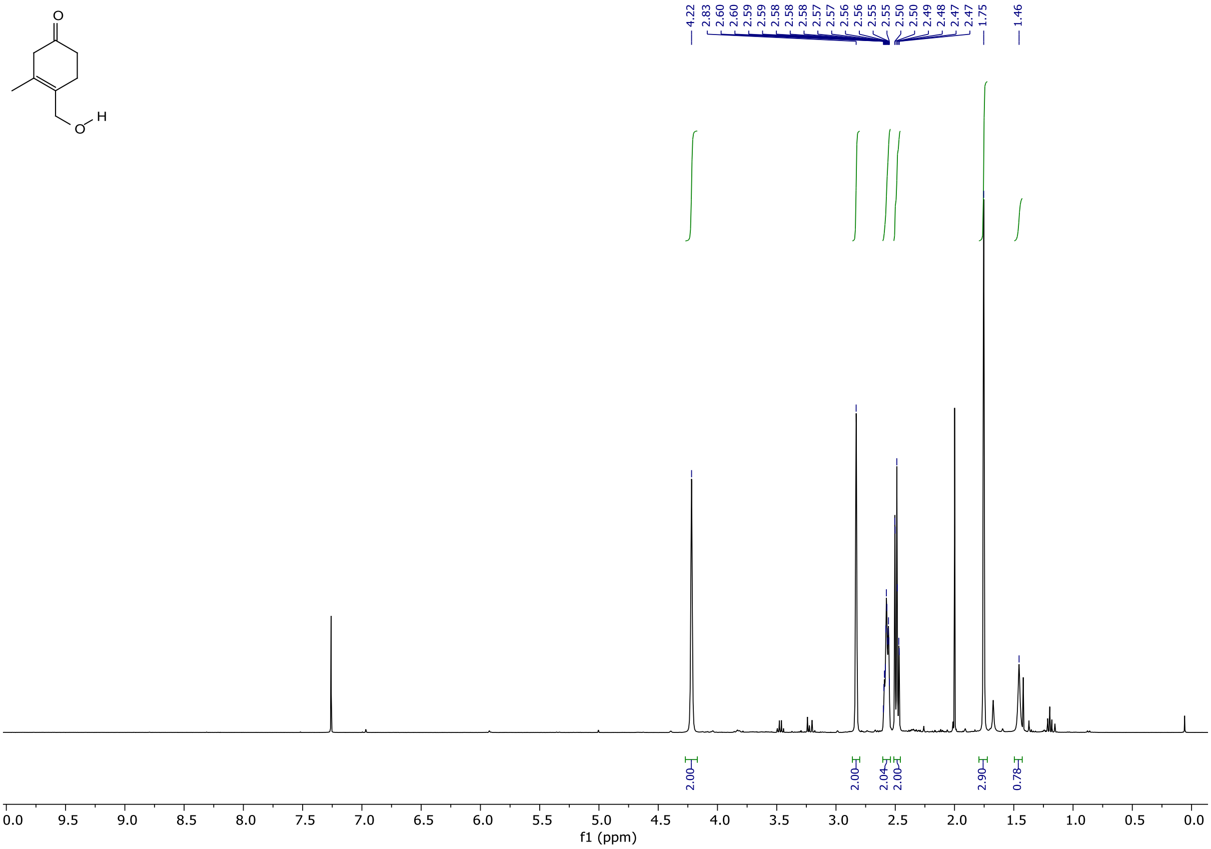

¹³C NMR (101 MHz, CDCl₃) of **1g**

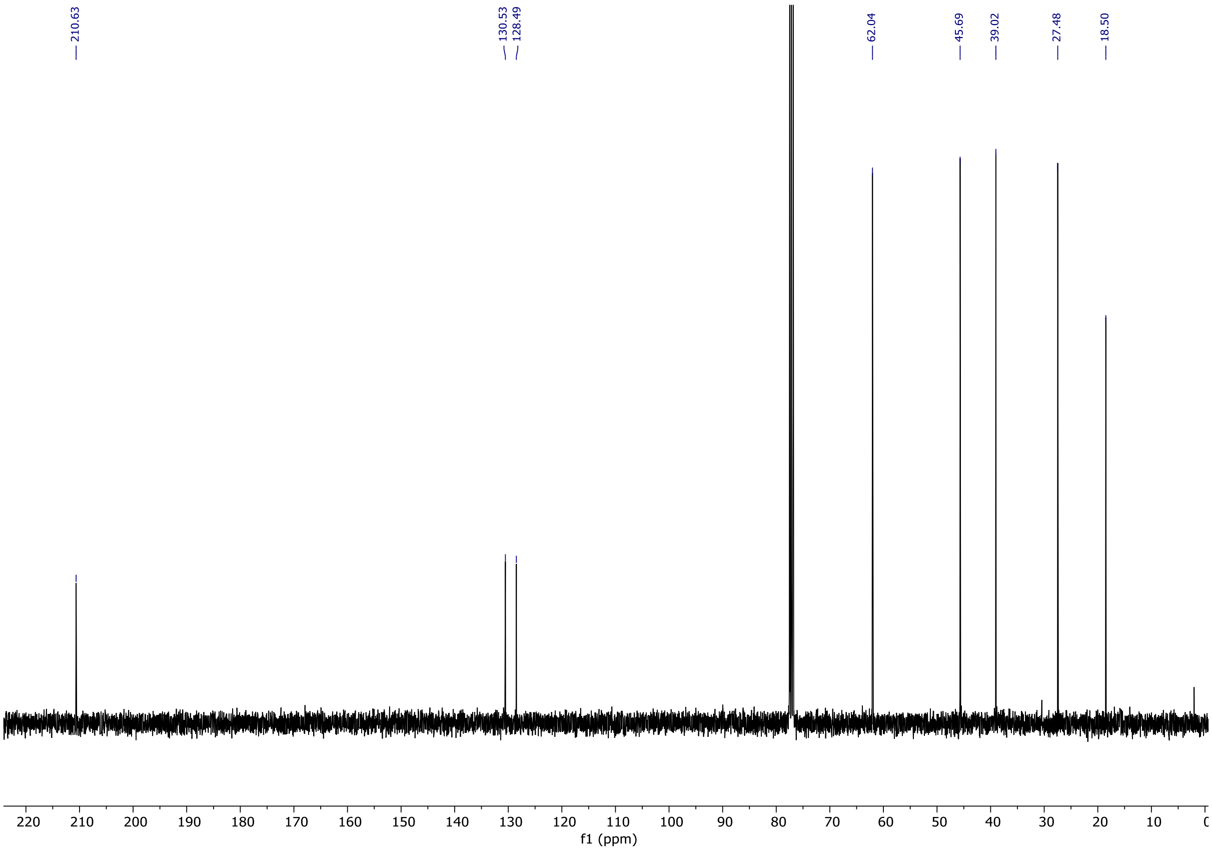

^{1}H NMR (400 MHz, Acetone-d_6) of **1h**

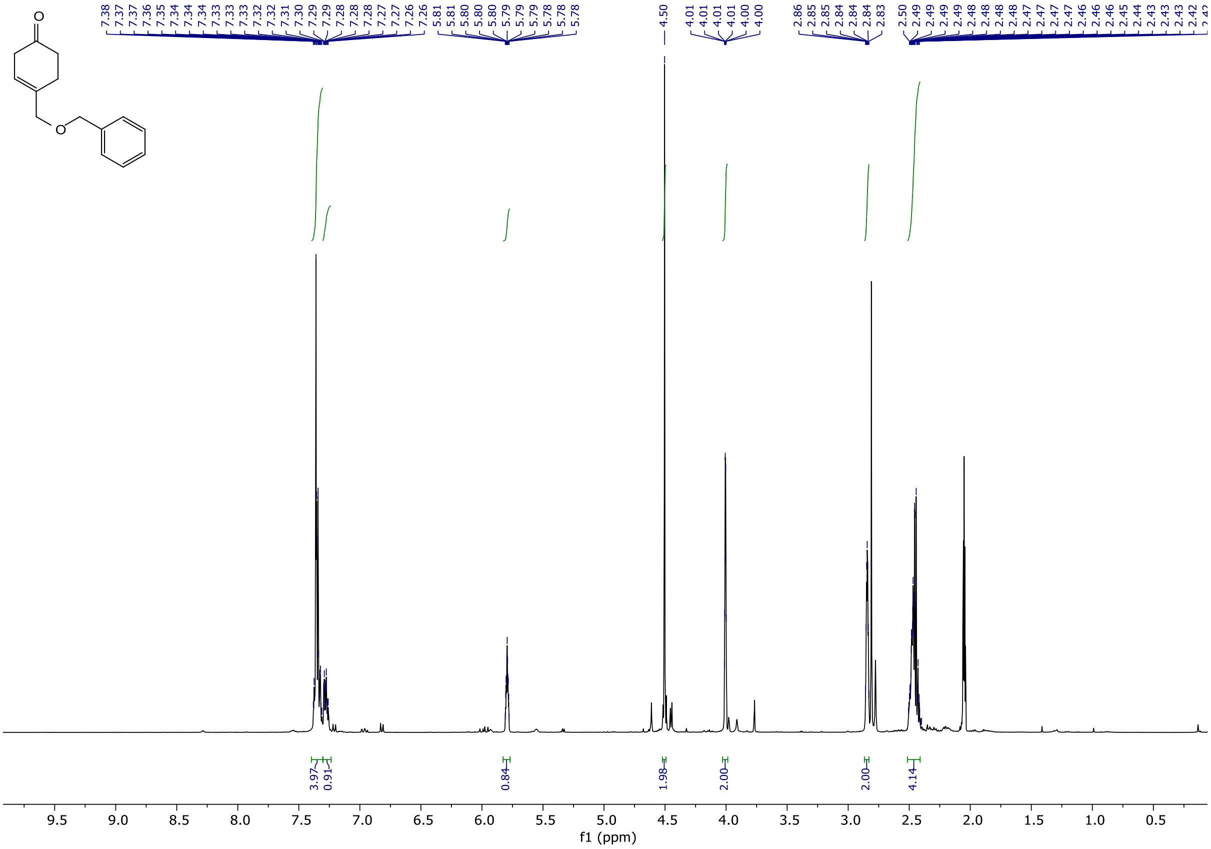

^{13}C NMR (151 MHz, Acetone-d_6) of **1h**

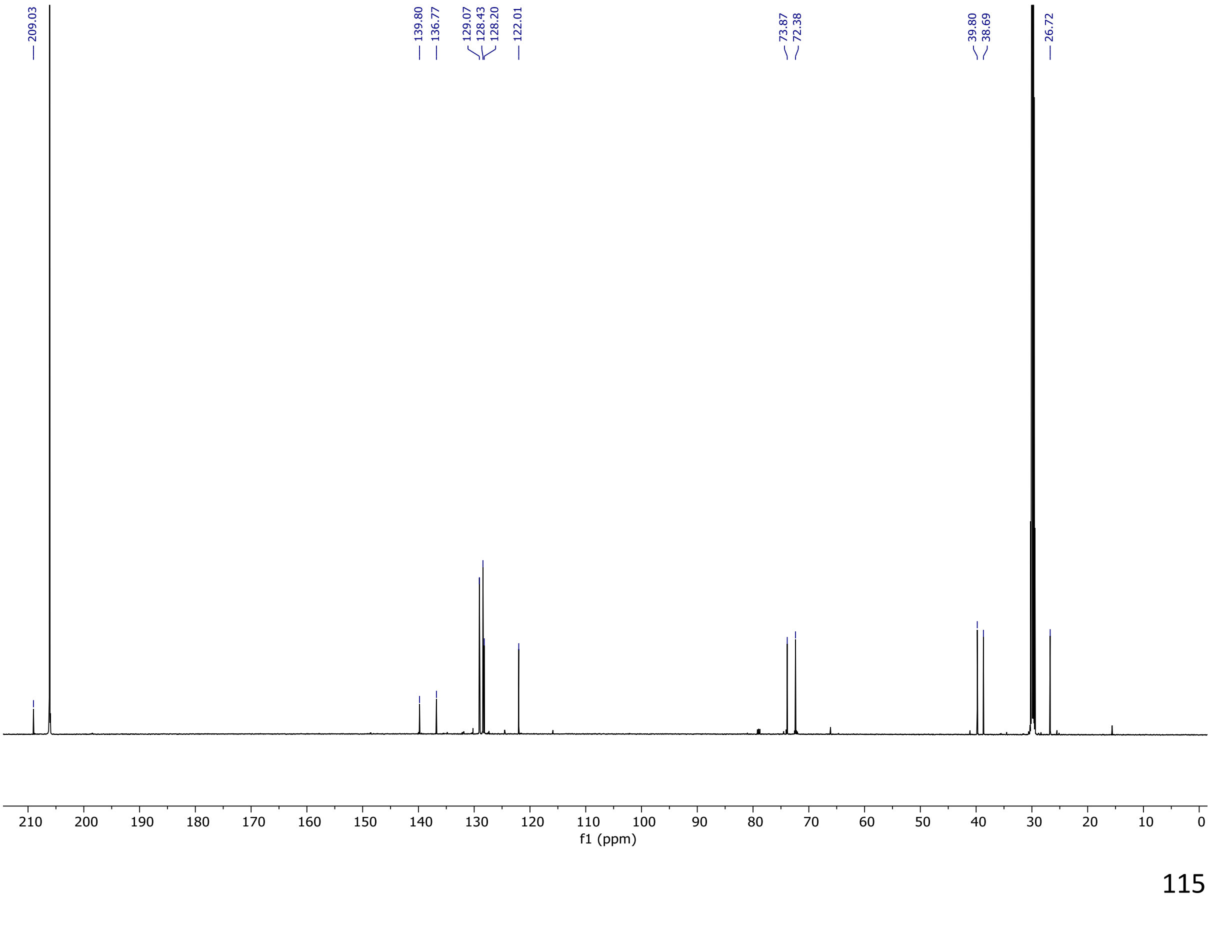

^{1}H NMR (600 MHz, Acetone-d_6) of **1i**

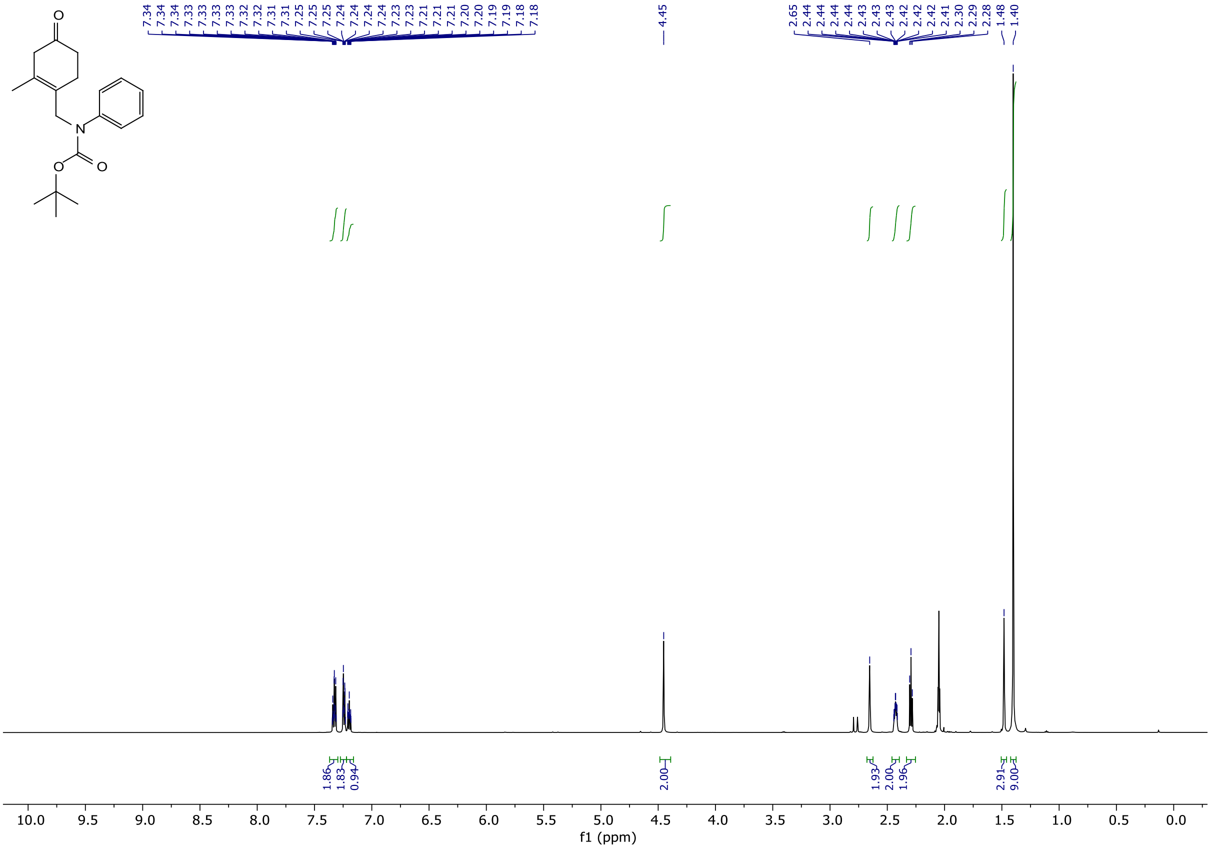

^{13}C NMR (151 MHz, Acetone-d_6) of **1i**

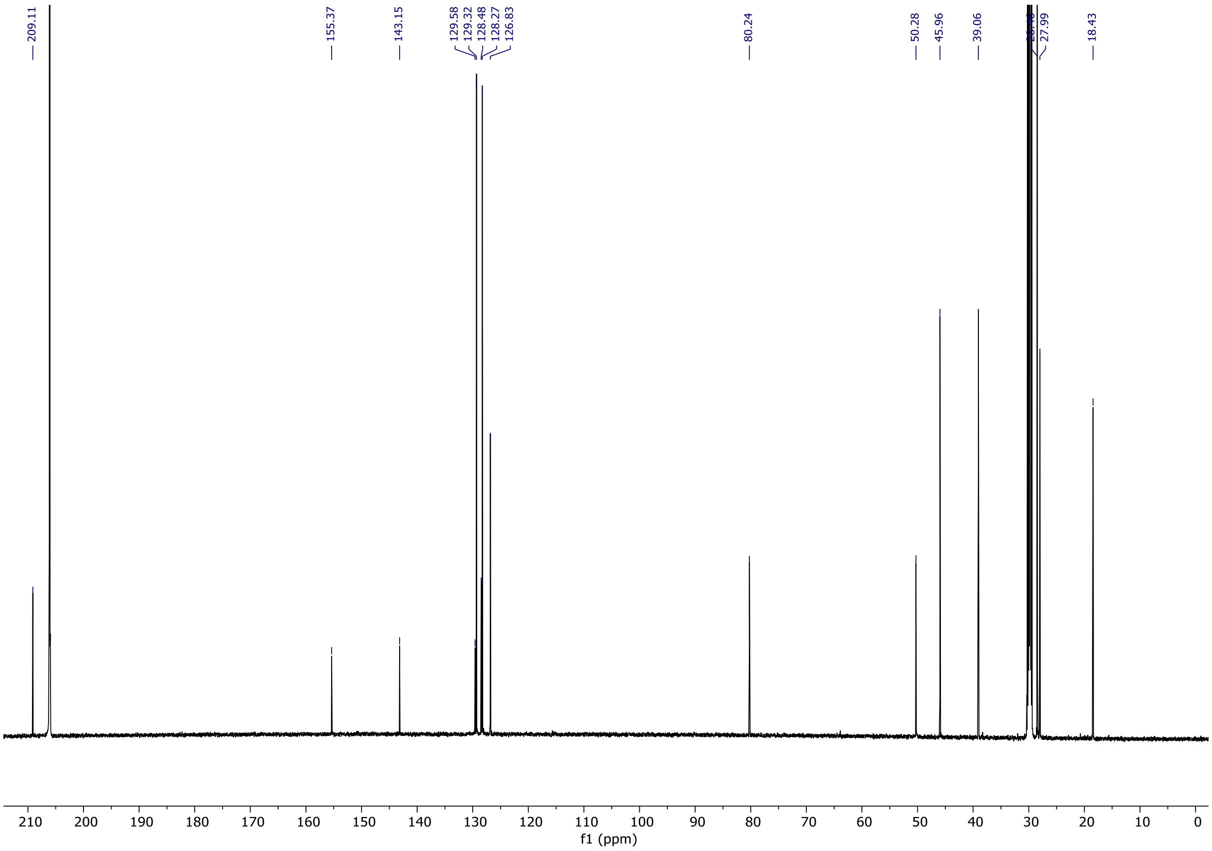

^{1}H NMR (600 MHz, Acetone-d_6) of **1j**

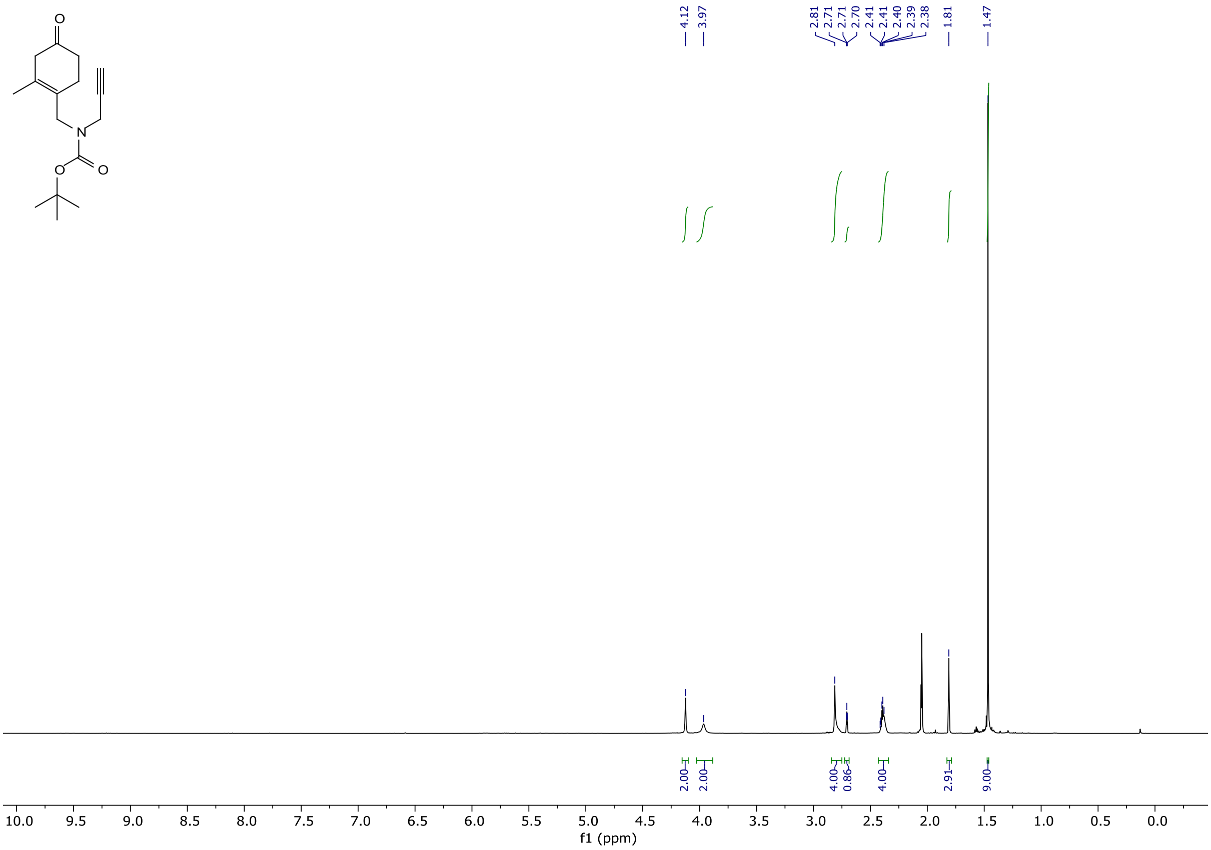

^{13}C NMR (151 MHz, Acetone-d_6) of **1j**

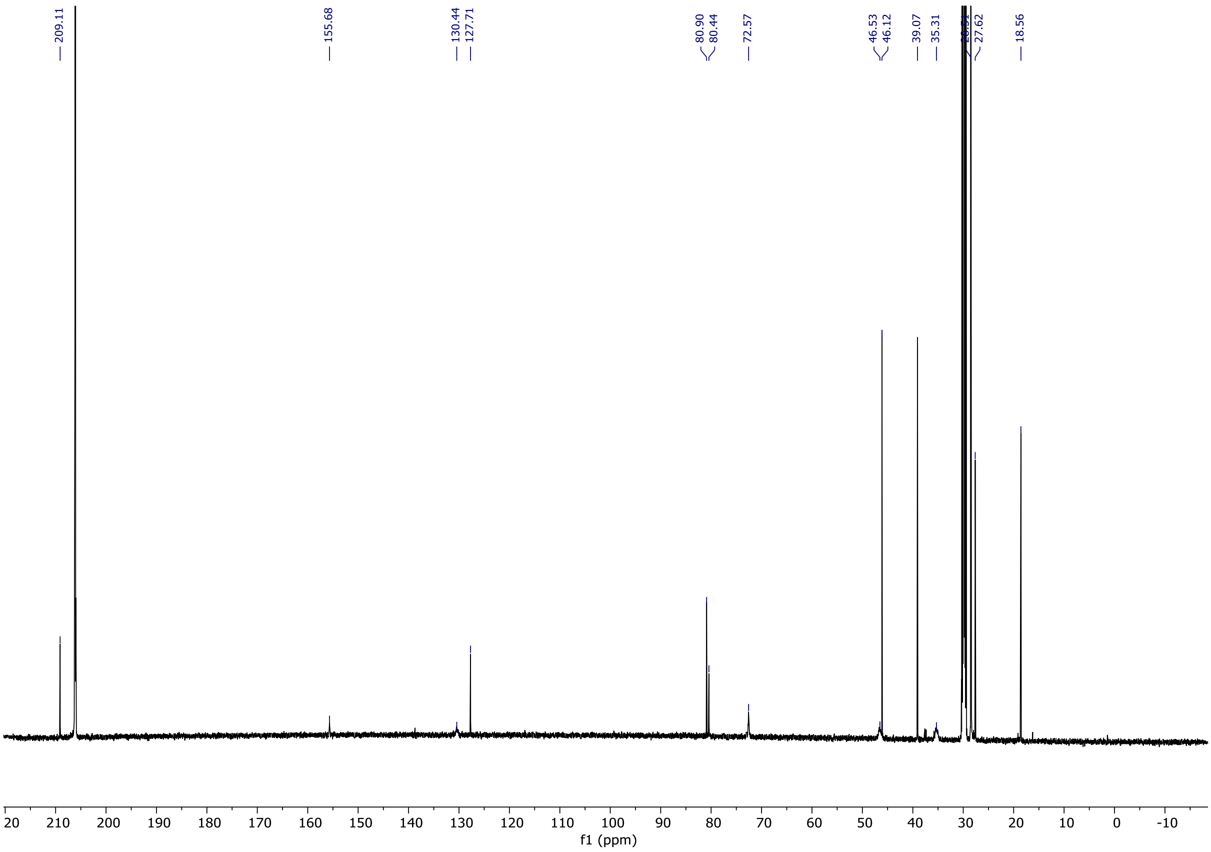

^{1}H NMR (400 MHz, Methanol-d_4) of **1k**

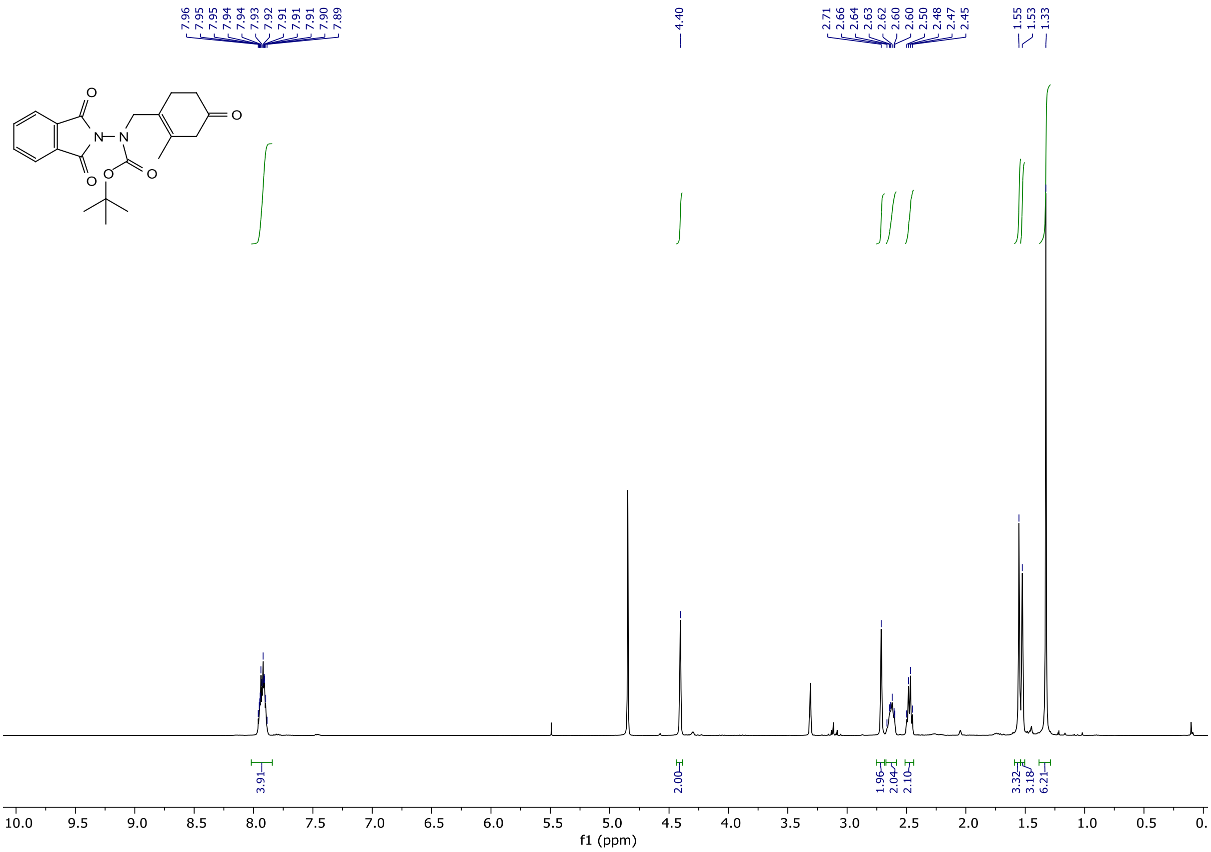

^{13}C NMR (101 MHz, Methanol-d_4) of **1k**

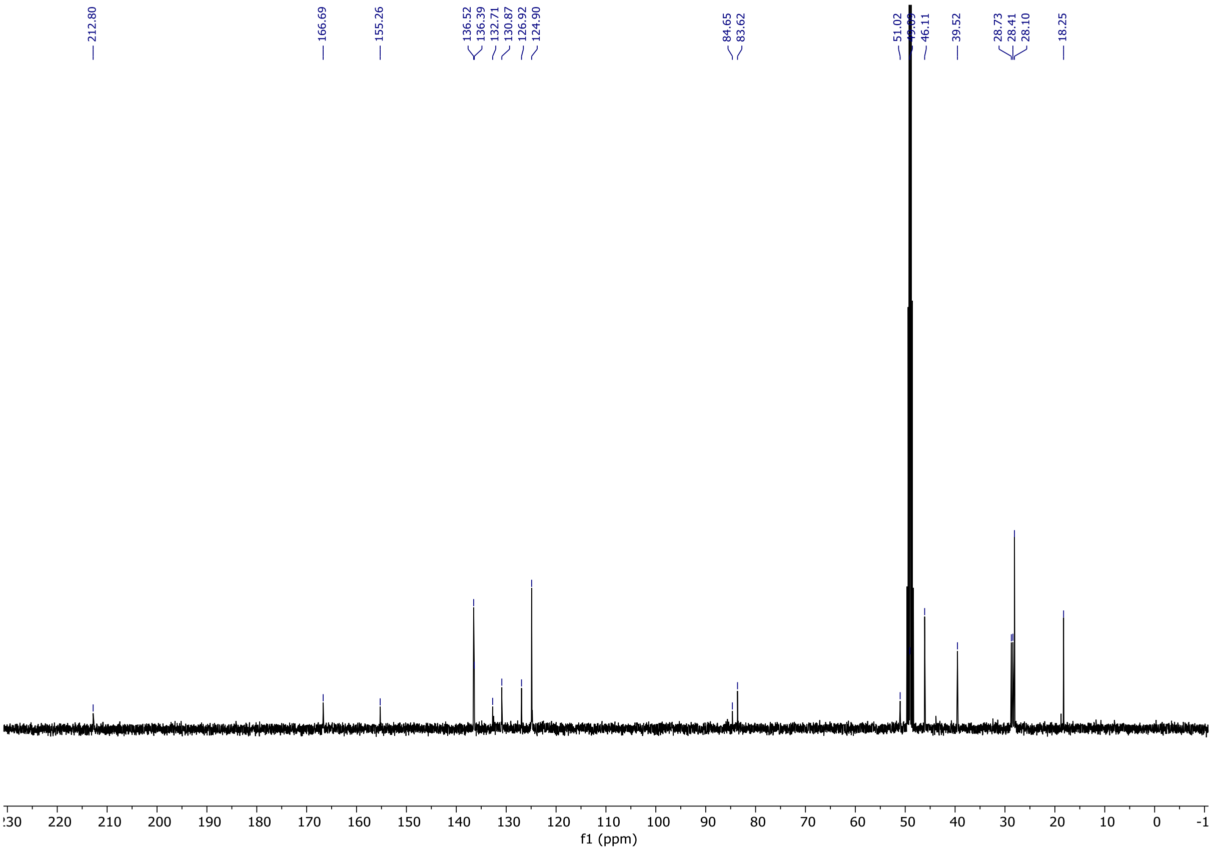

^{1}H NMR (400 MHz, Acetone-d_6) of **1l**

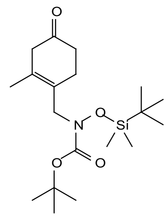

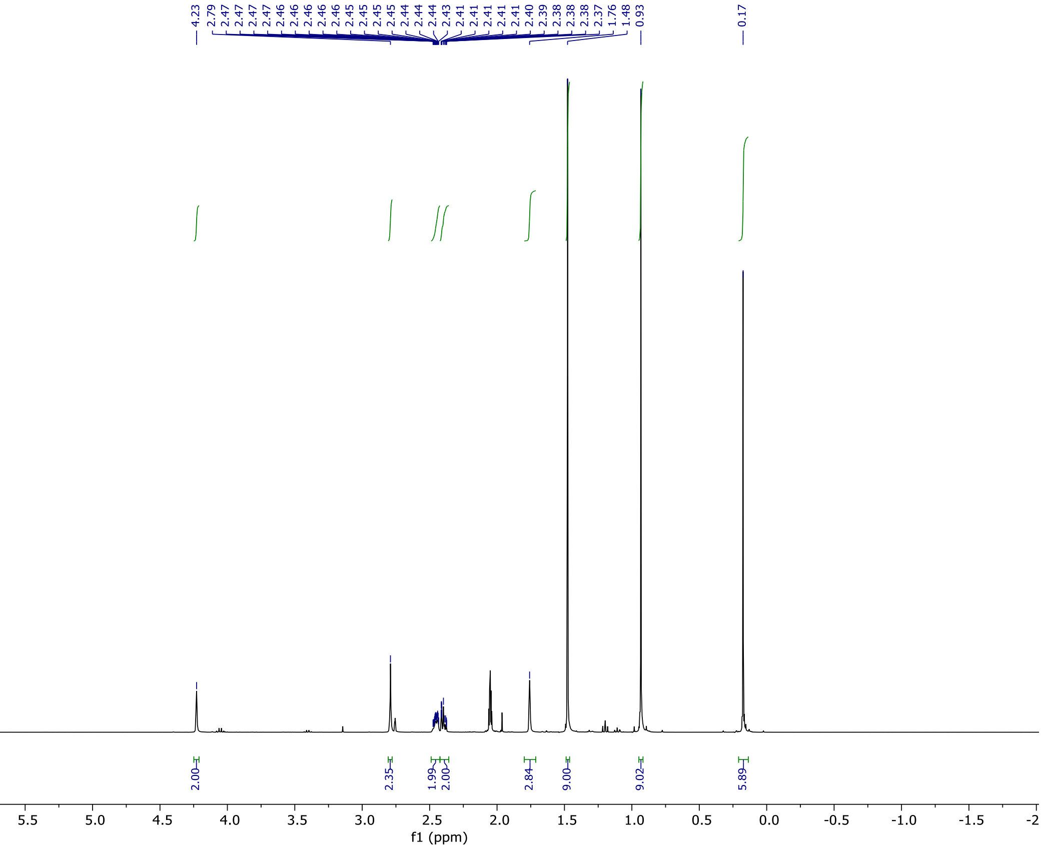

^{13}C NMR (126 MHz, Acetone-d_6) of **1l**

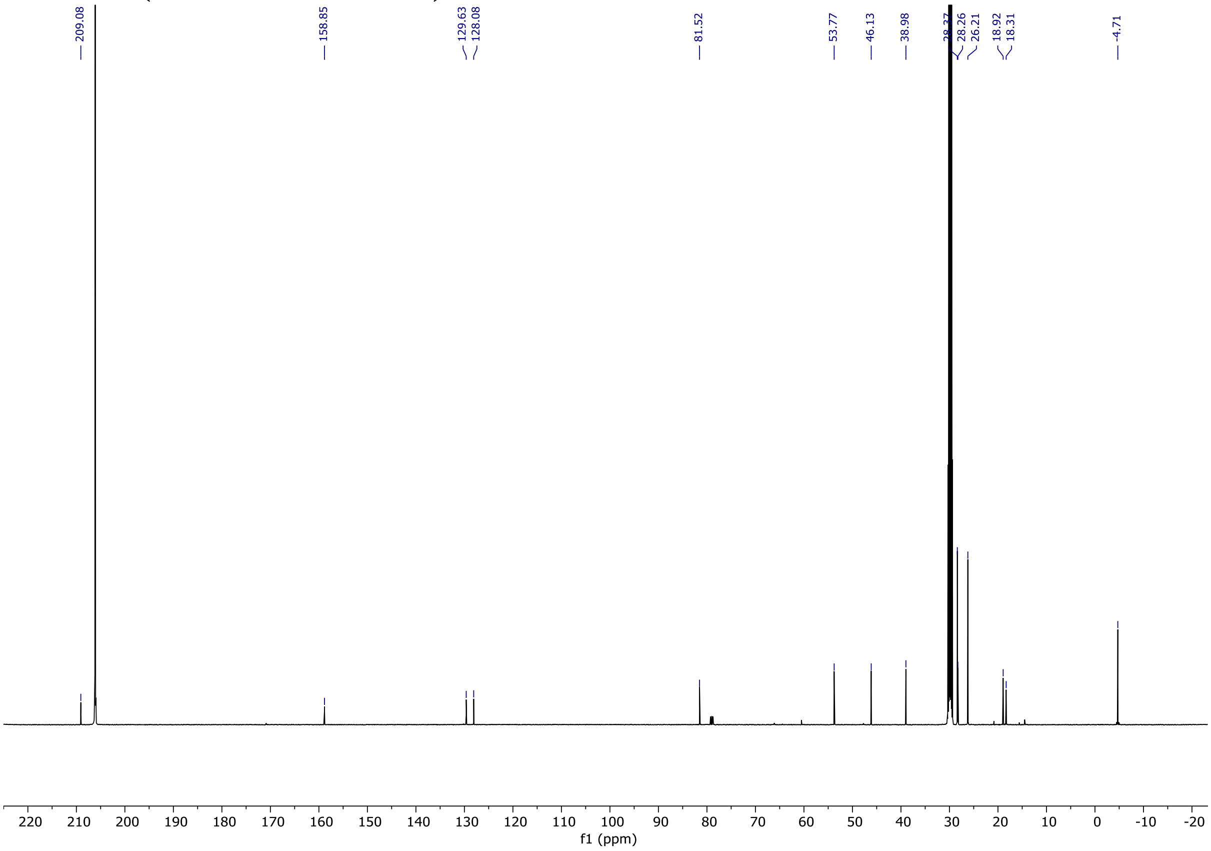

^{1}**H NMR** (500 MHz, Acetone-d_6) of **1m**

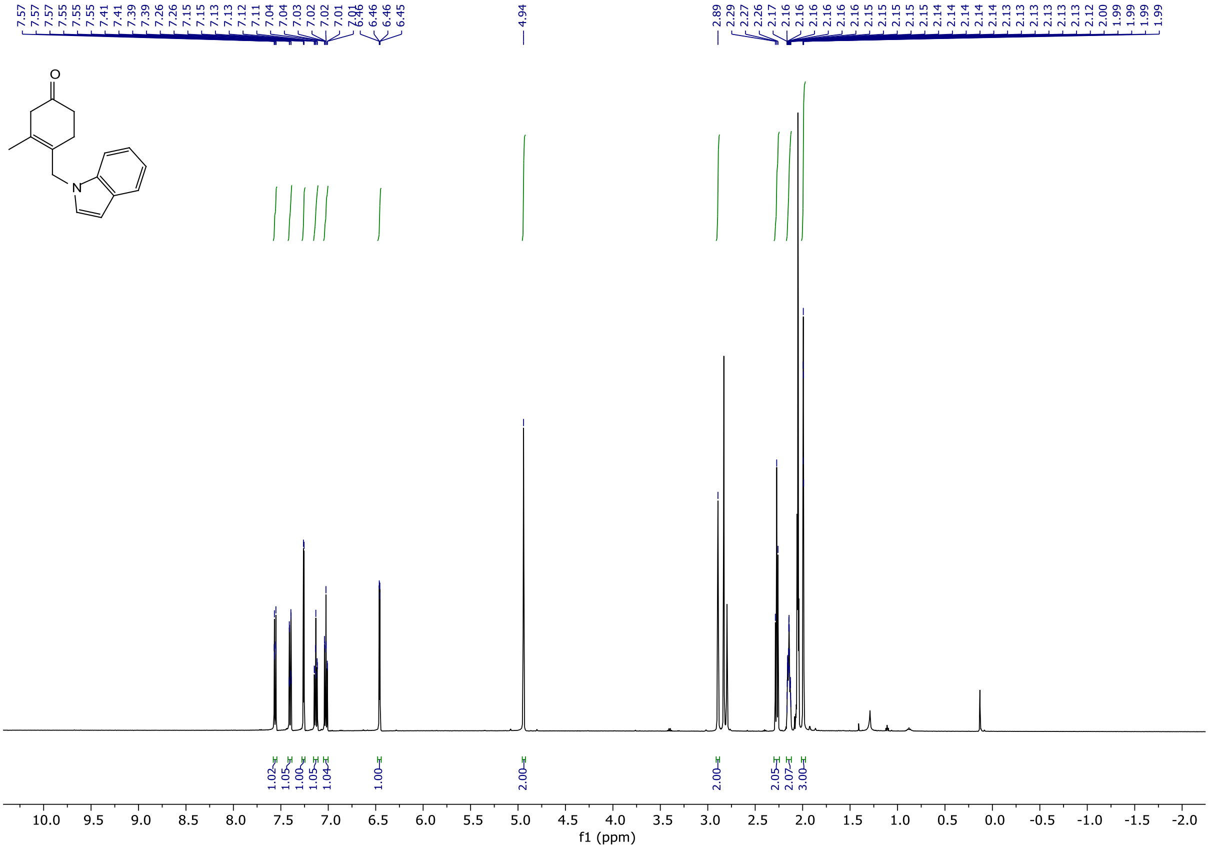

^{13}**C NMR** (126 MHz, Acetone-d_6) of **1m**

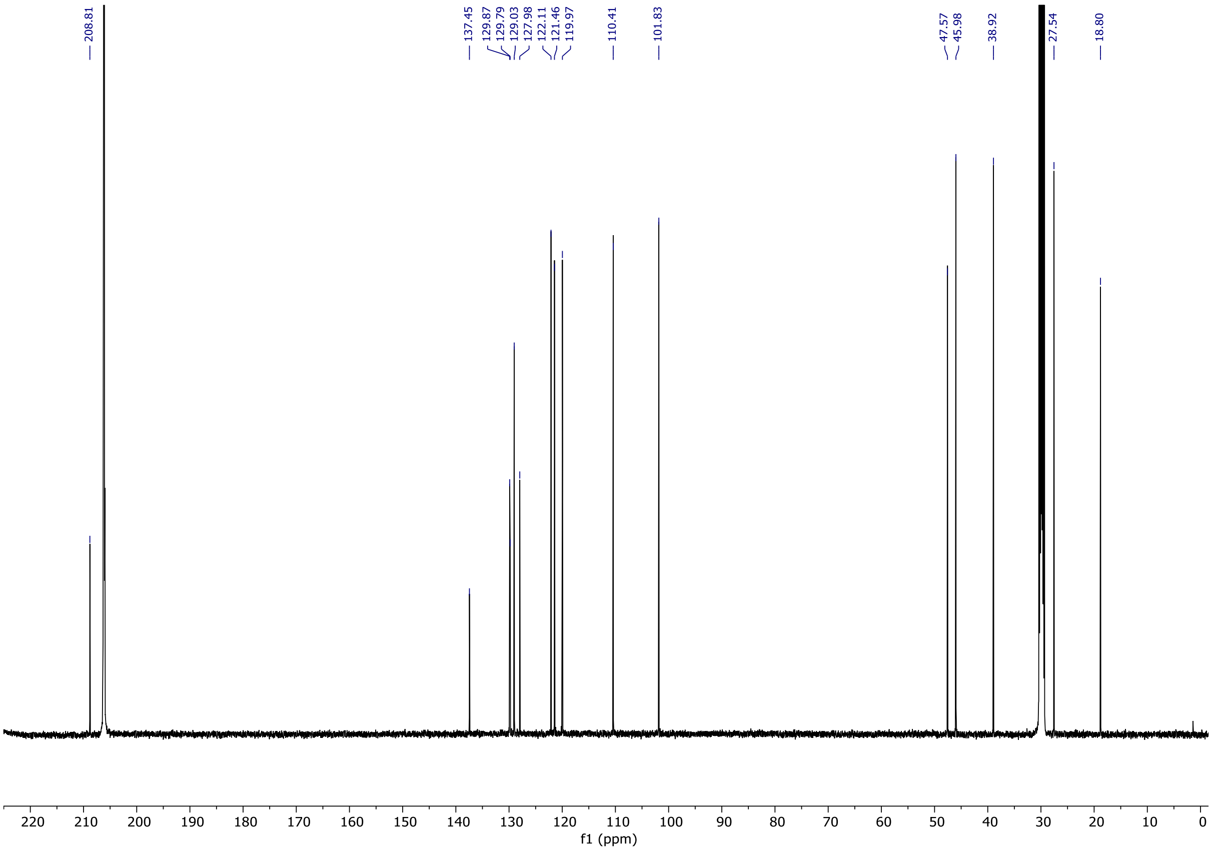

^{1}H NMR (500 MHz, Benzene-d_6) of **1n**

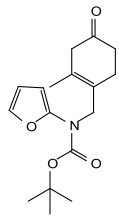

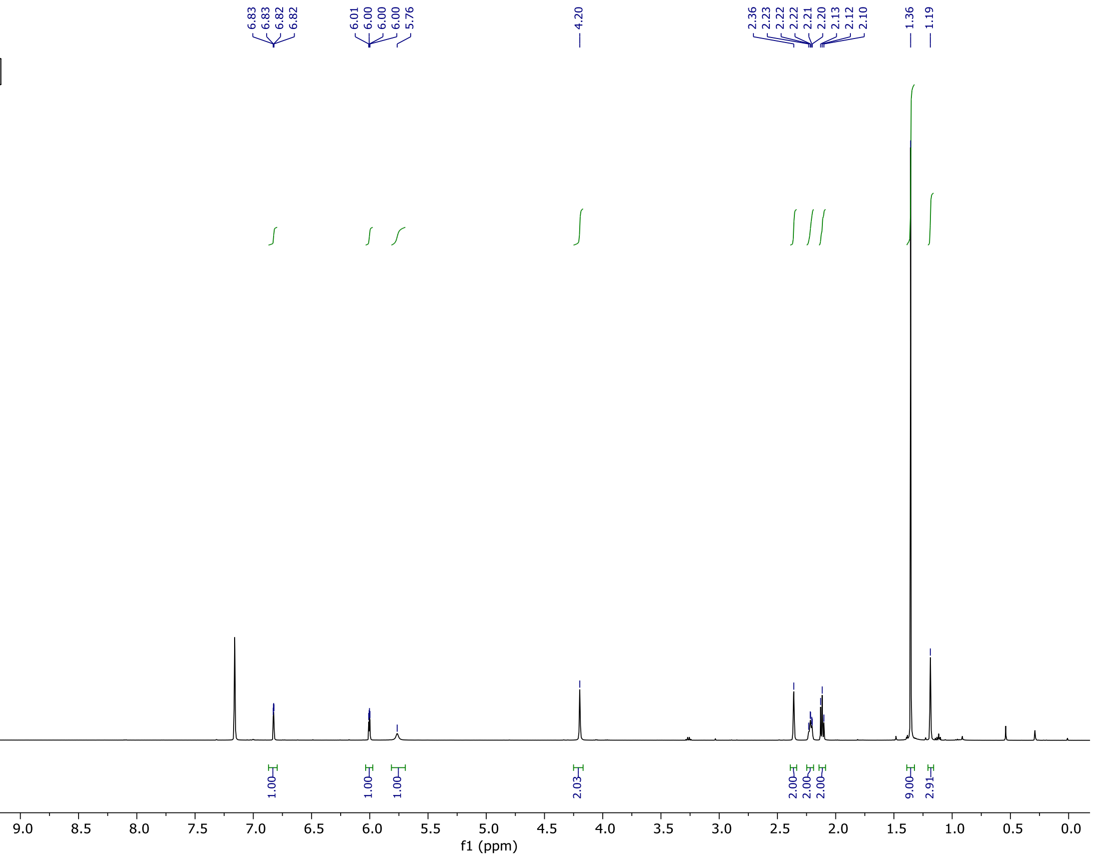

^{13}C NMR (126 MHz, Benzene-d_6) of **1n**

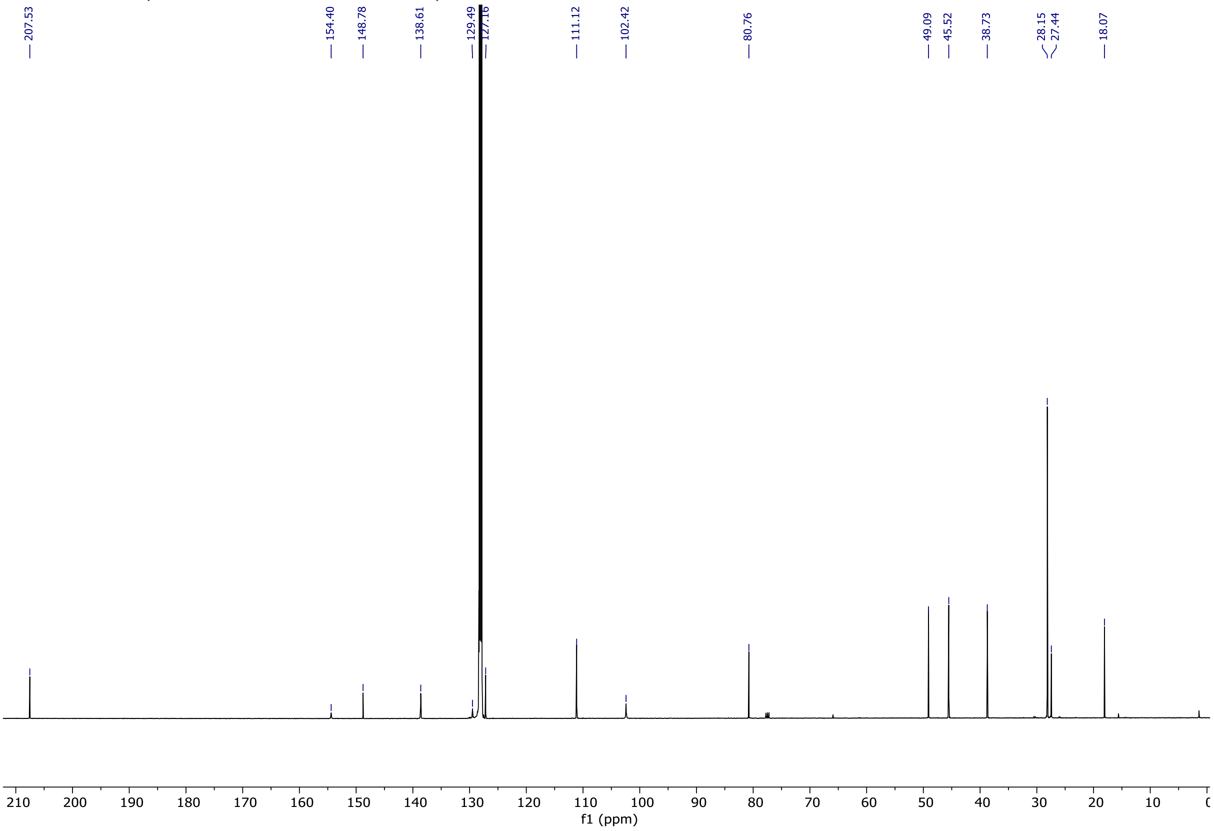

^{1}H NMR (500 MHz, Acetone-d_6) of **1o**

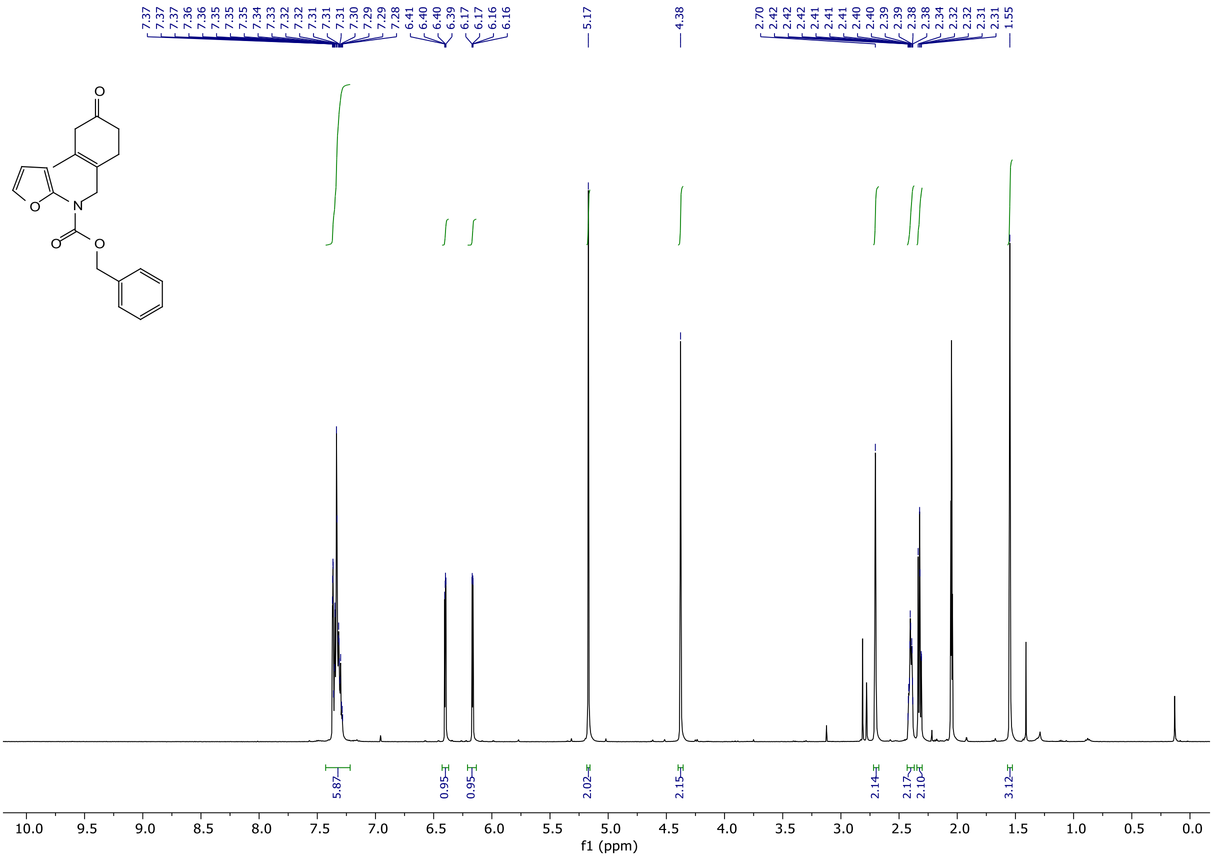

^{13}C NMR (126 MHz, Acetone-d_6) of **1o**

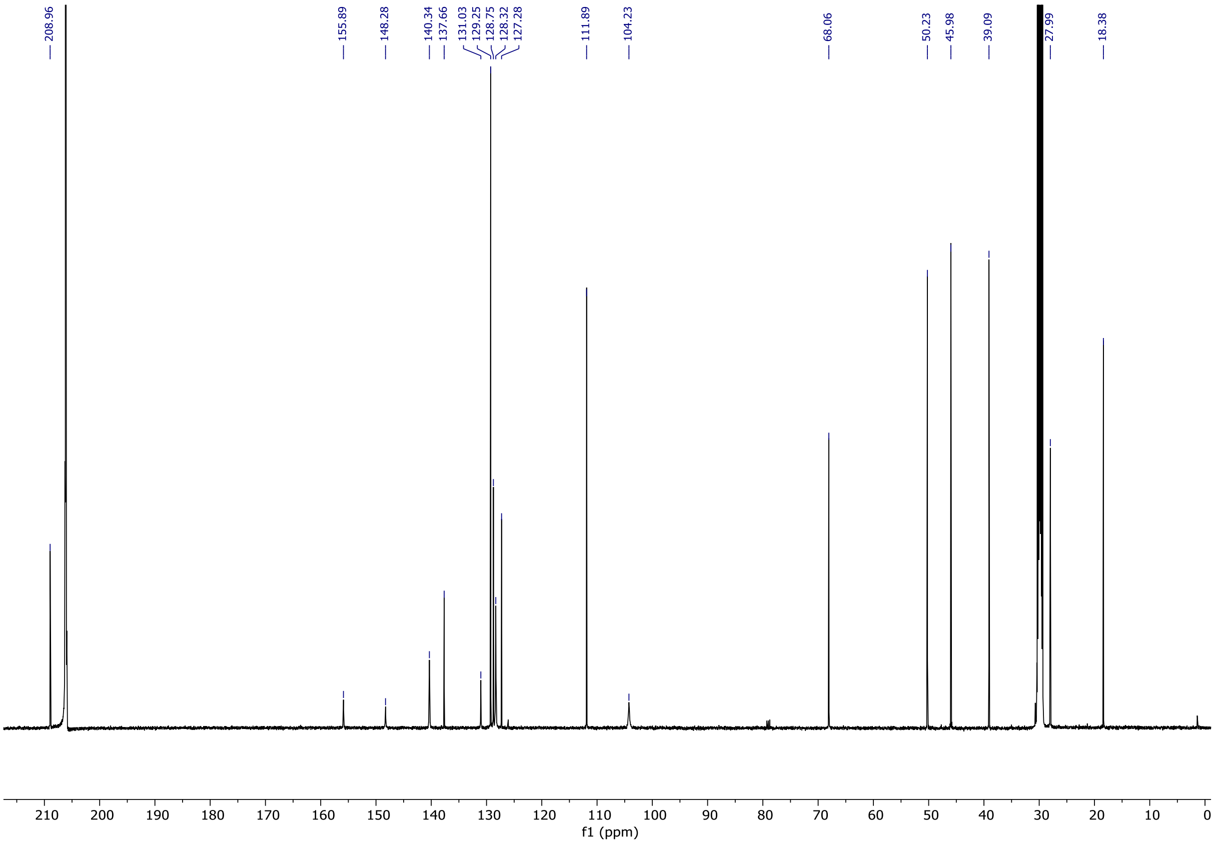

^{1}H NMR (500 MHz, CDCl$_3$) of **1p**

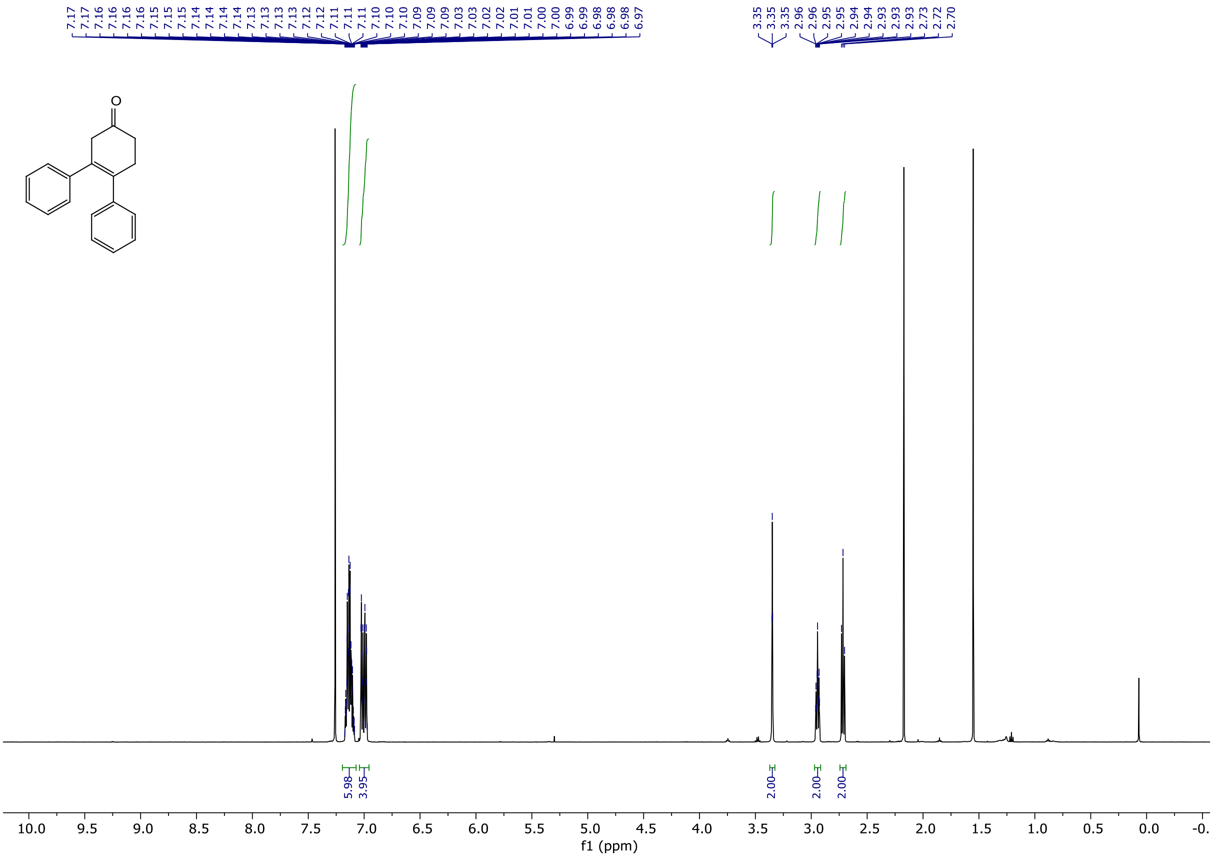

^{13}C NMR (126 MHz, CDCl$_3$) of **1p**

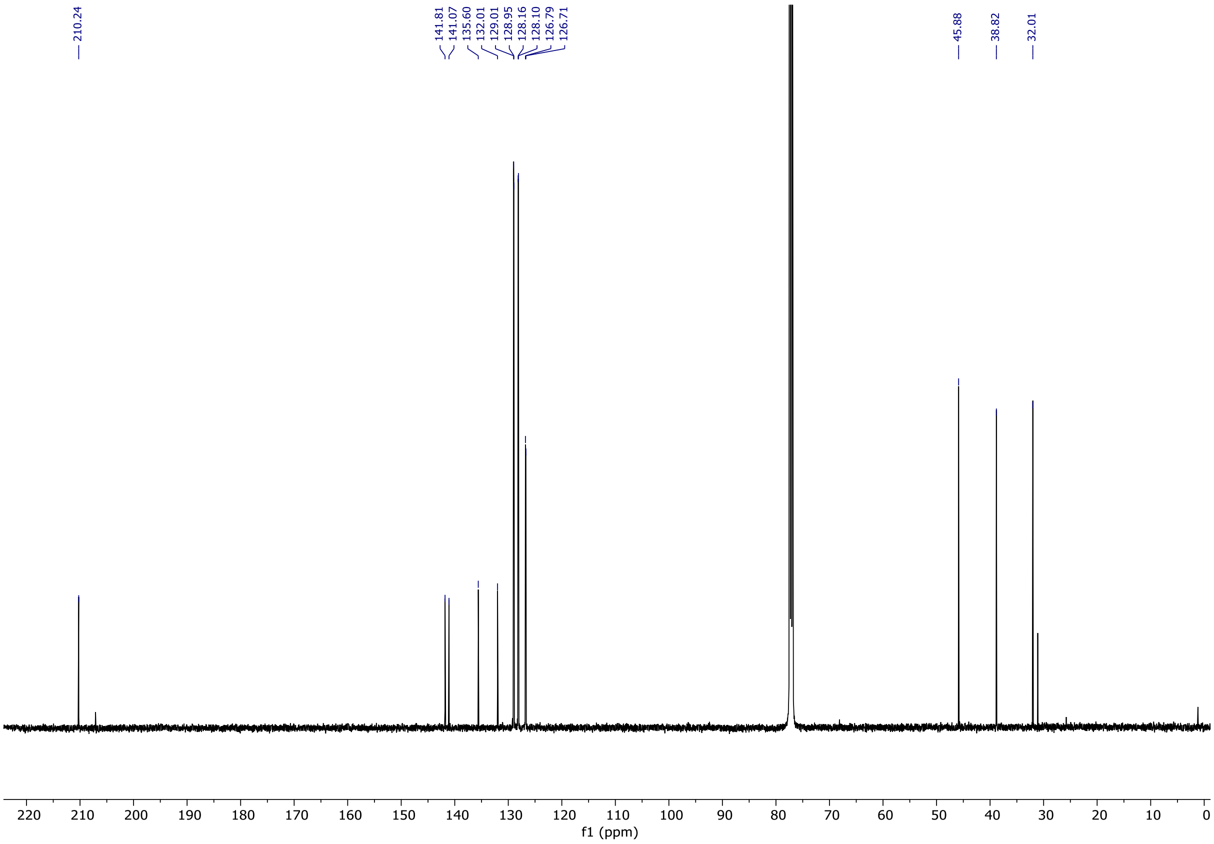

^{1}H NMR (500 MHz, Benzene-d_6) of **1q**

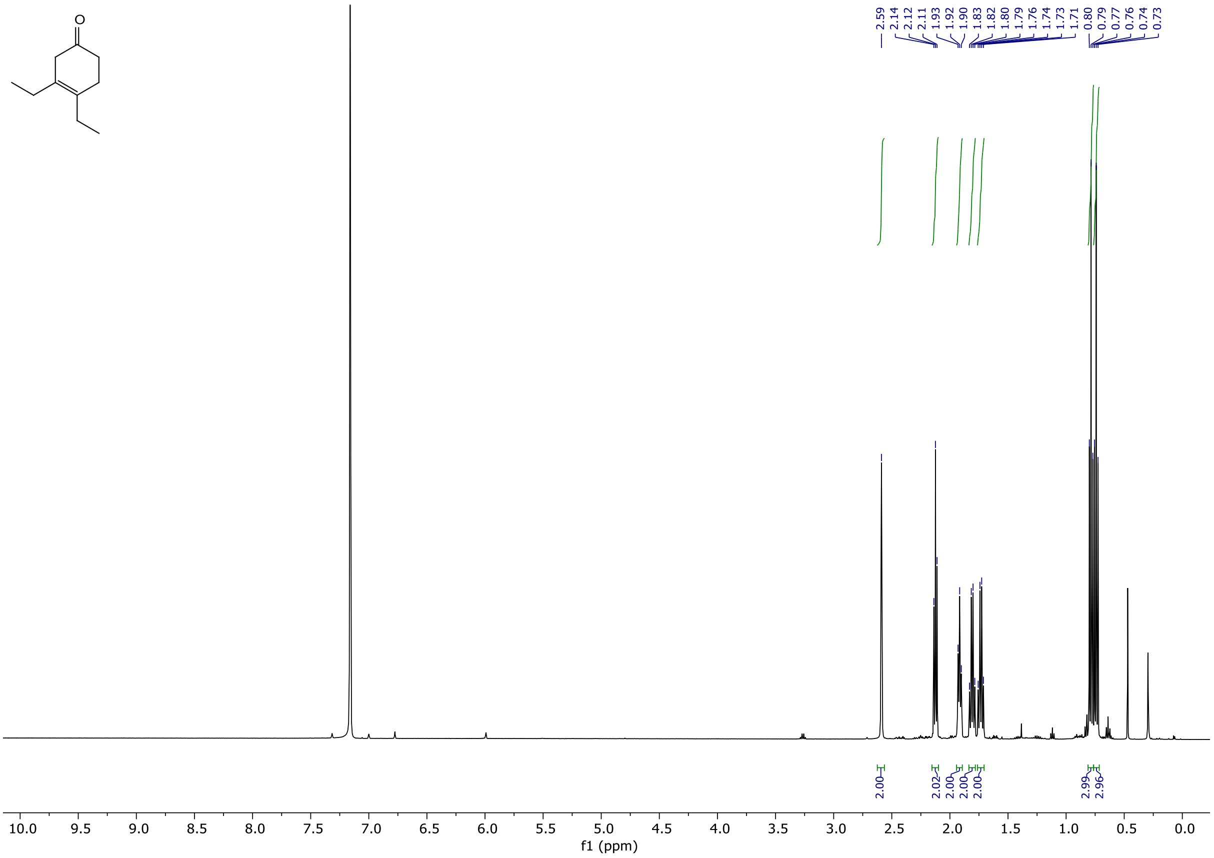

^{13}C NMR (126 MHz, Benzene-d_6) of **1q**

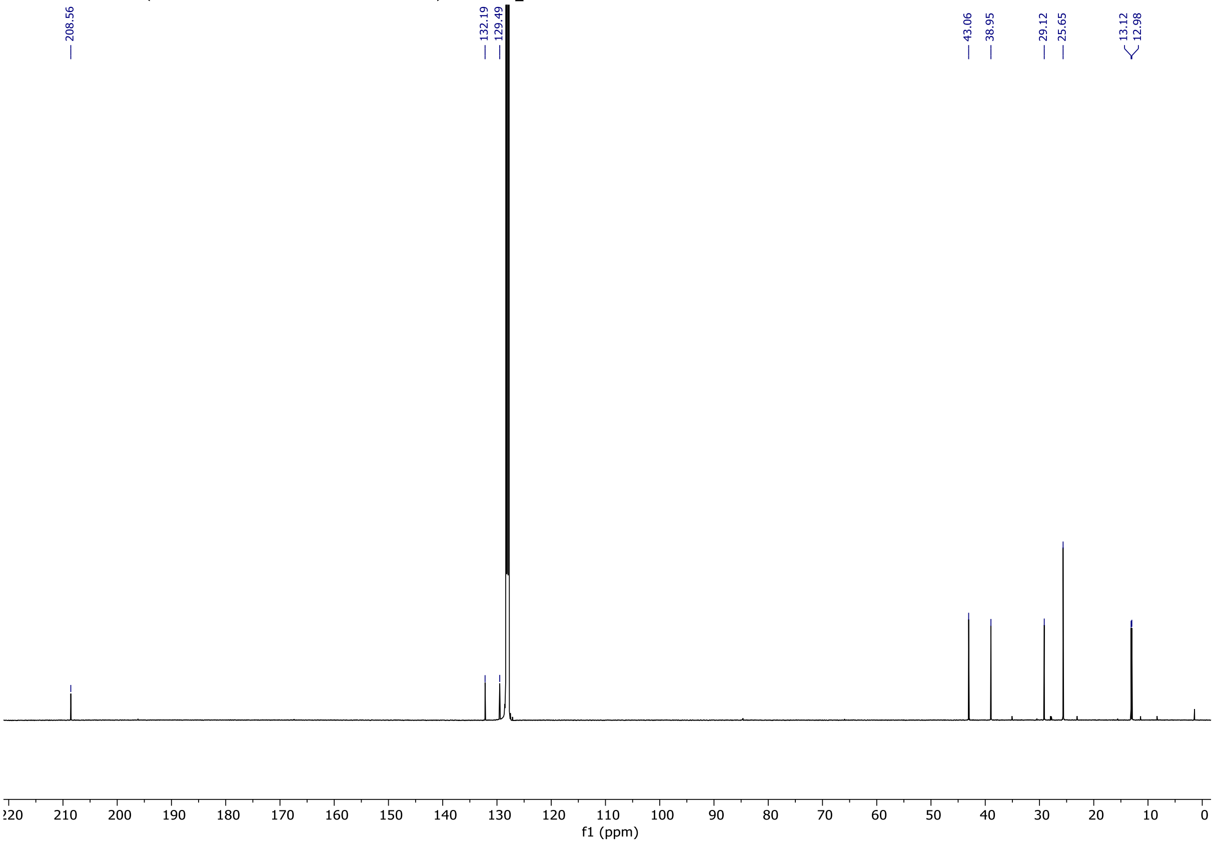

^{1}H NMR (400 MHz, Benzene-*d*$_6$) of **1r**

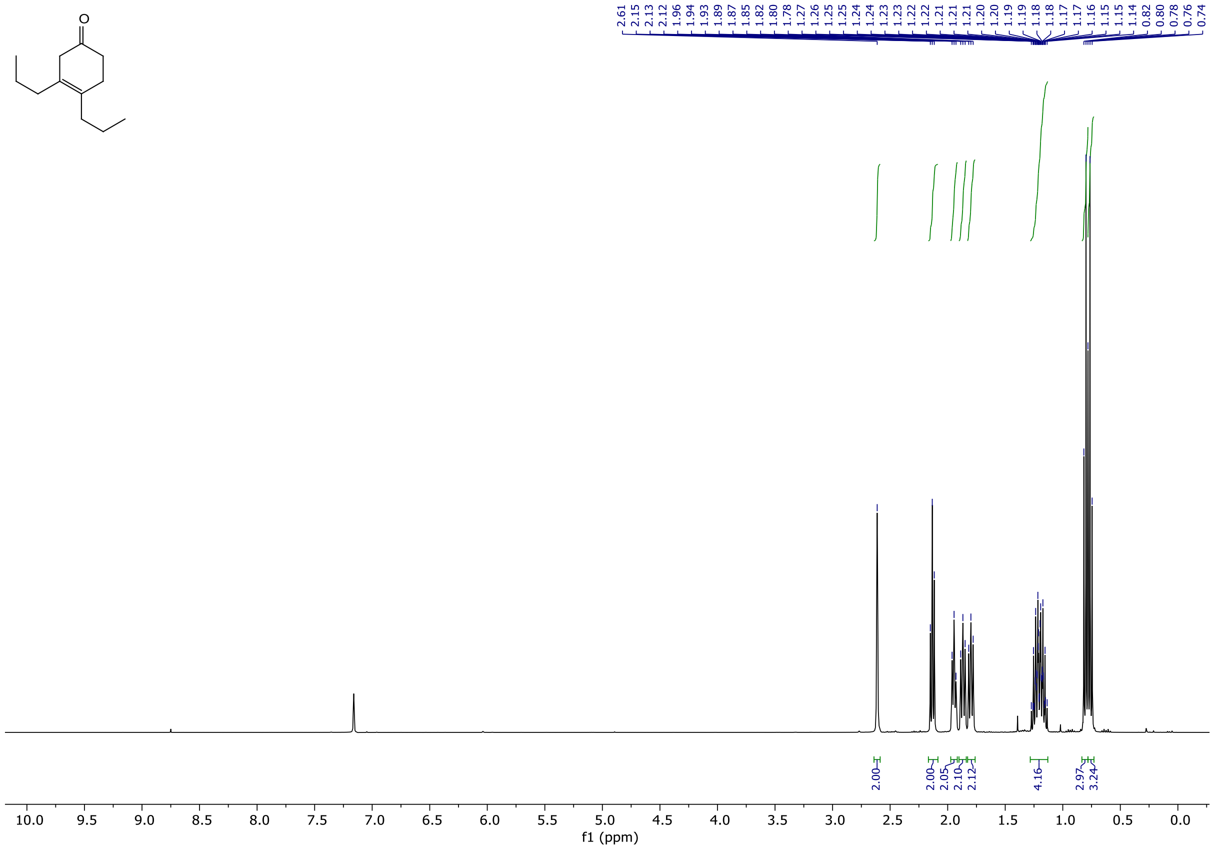

^{1}H NMR (101 MHz, Benzene-*d*$_6$) of **1r**

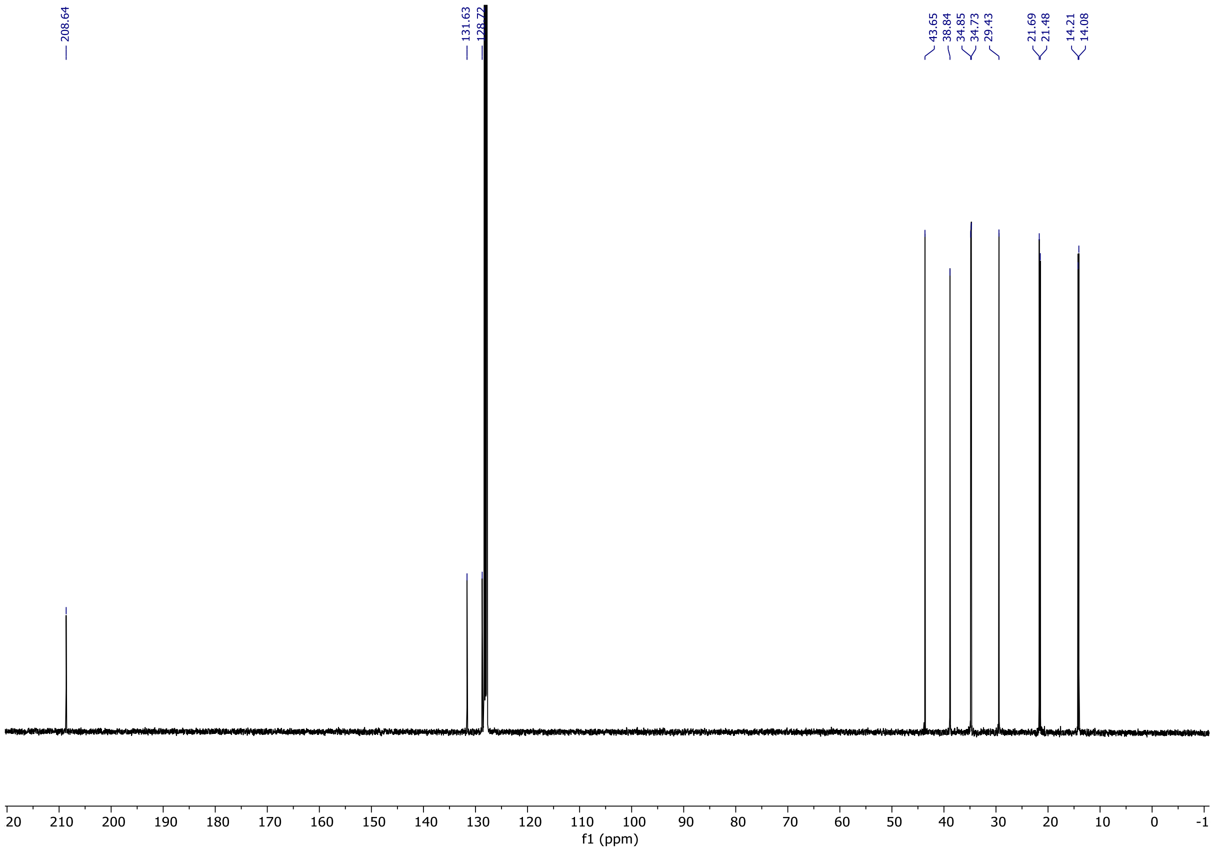

^{1}H NMR (400 MHz, Acetone-d_6) of 1s

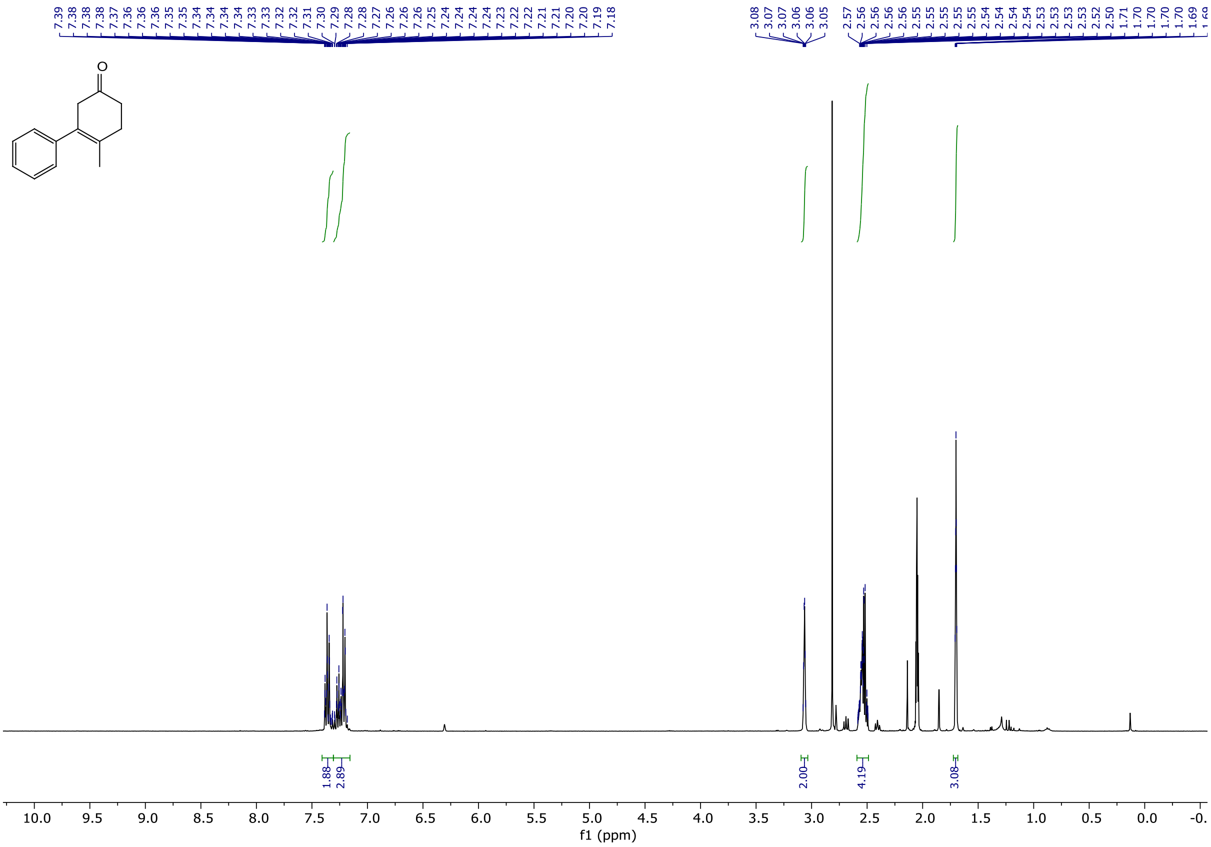

^{13}C NMR (126 MHz, Acetone-d_6) of 1s

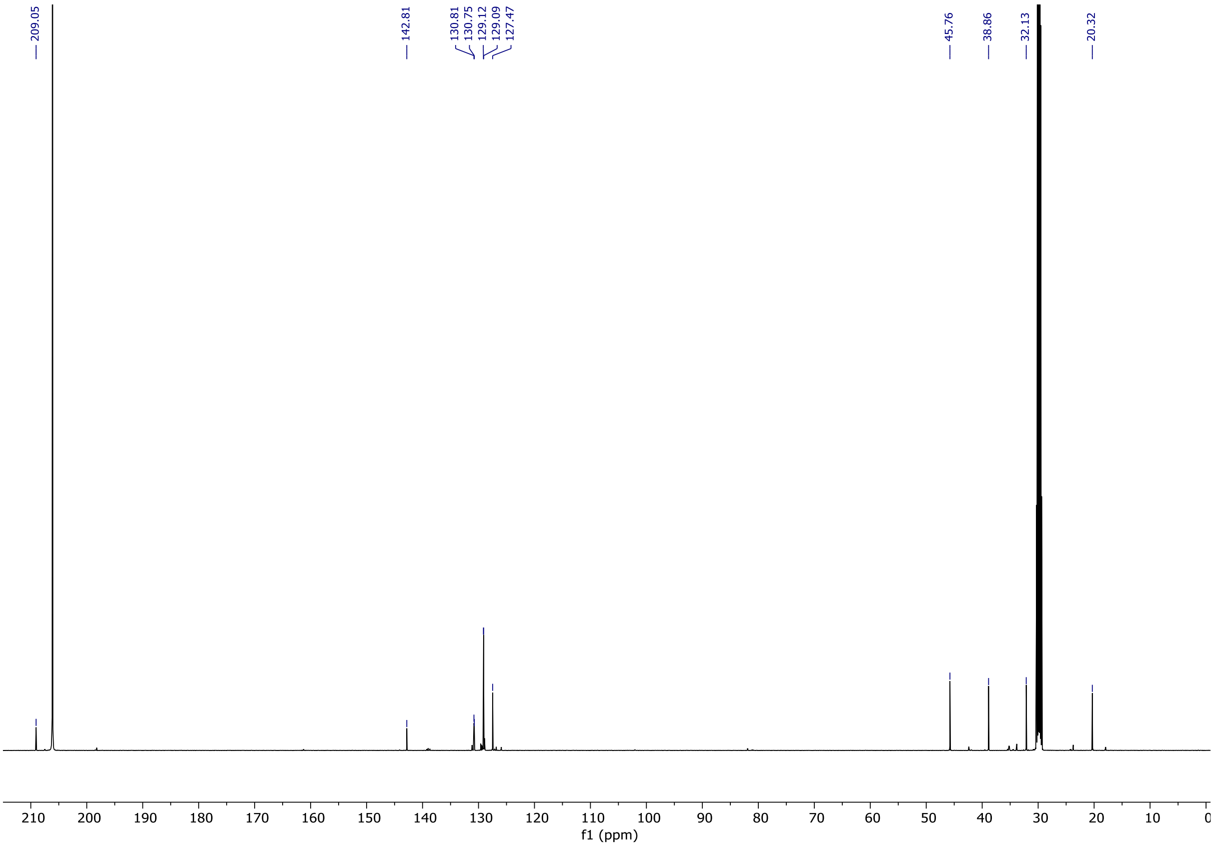

^{1}H NMR (400 MHz, Acetone-d_6) of **1t**

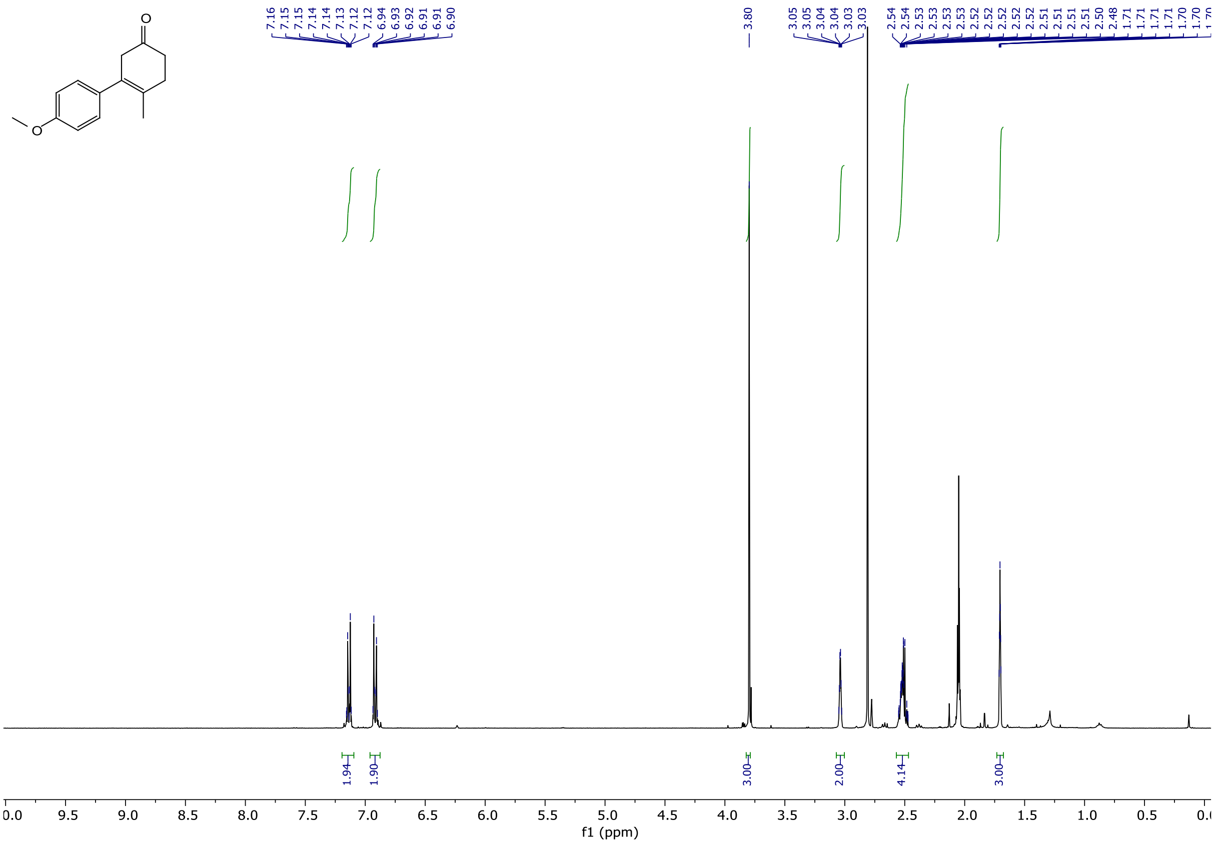

^{13}C NMR (126 MHz, Acetone-d_6) of **1t**

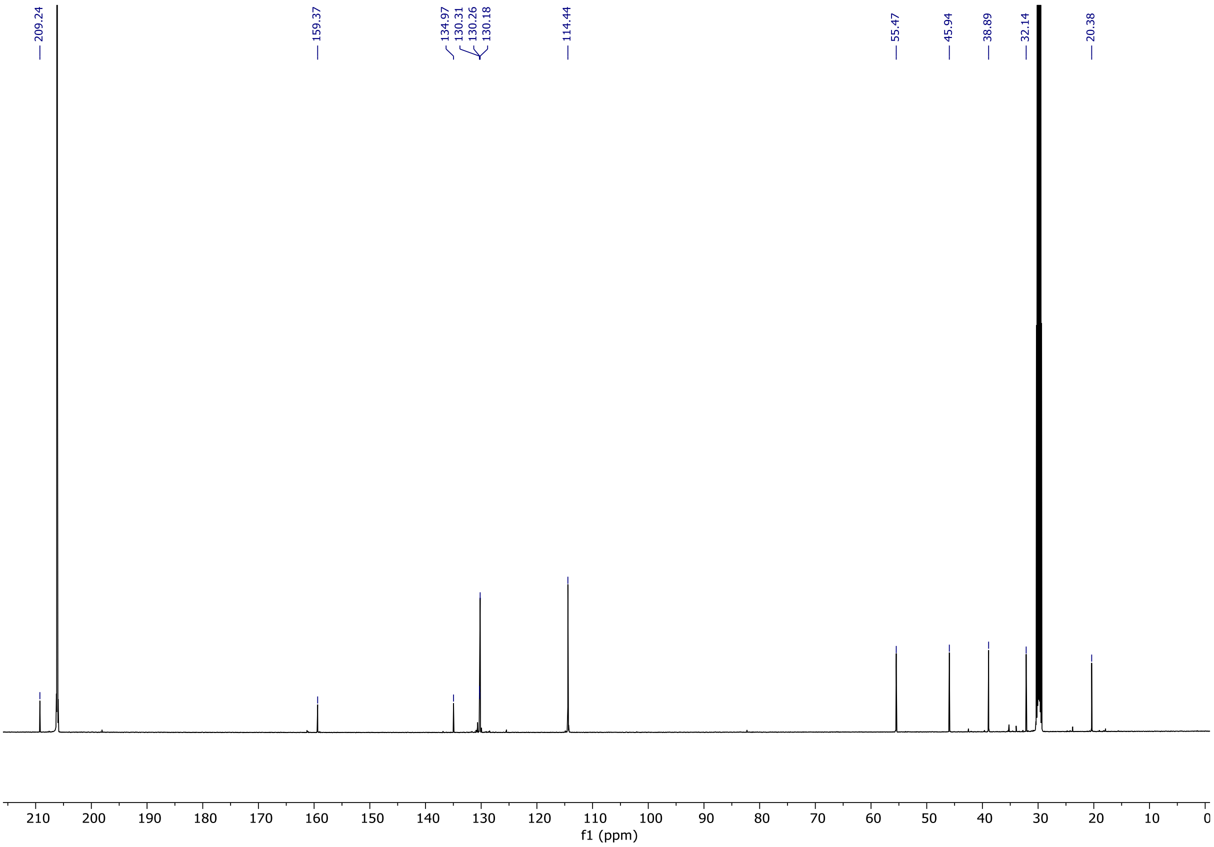

^{1}H NMR (400 MHz, $CDCl_3$) of **2a**

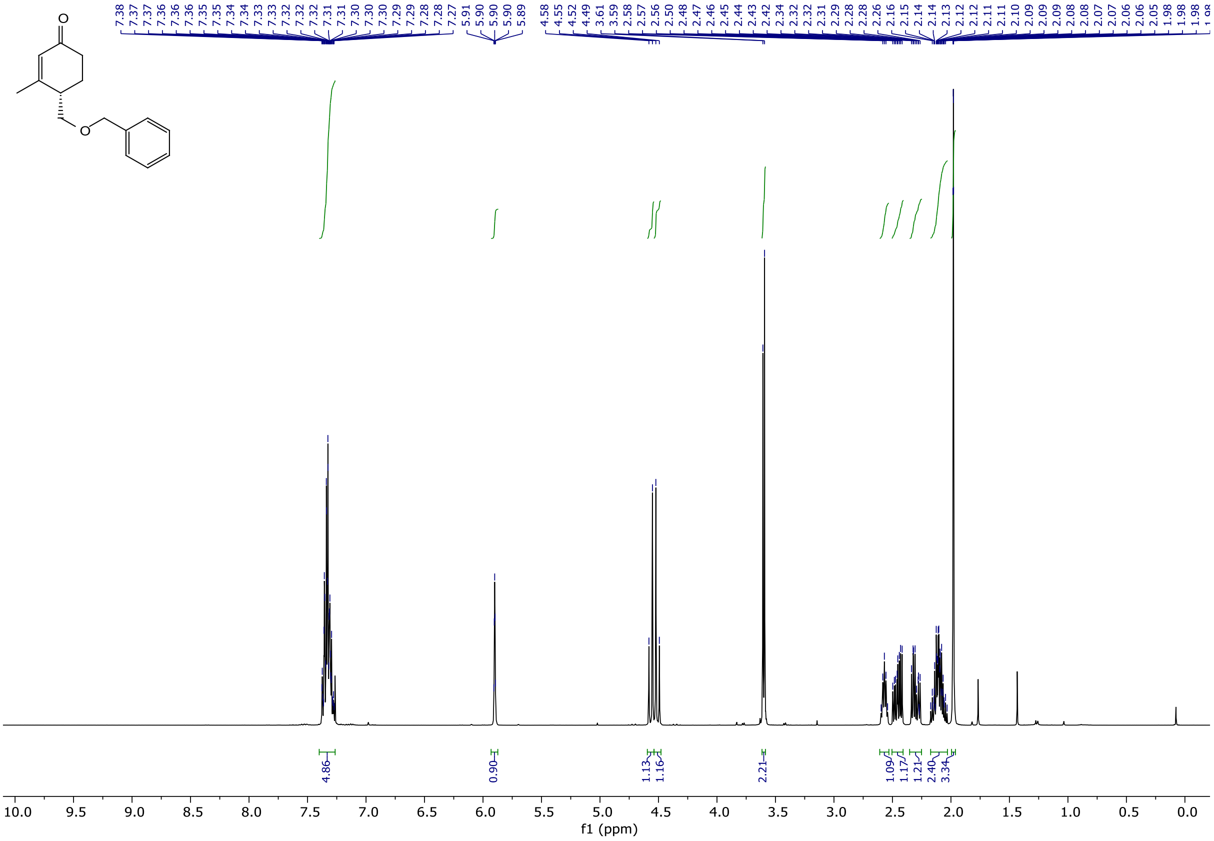

^{13}C NMR (101 MHz, $CDCl_3$) of **2a**

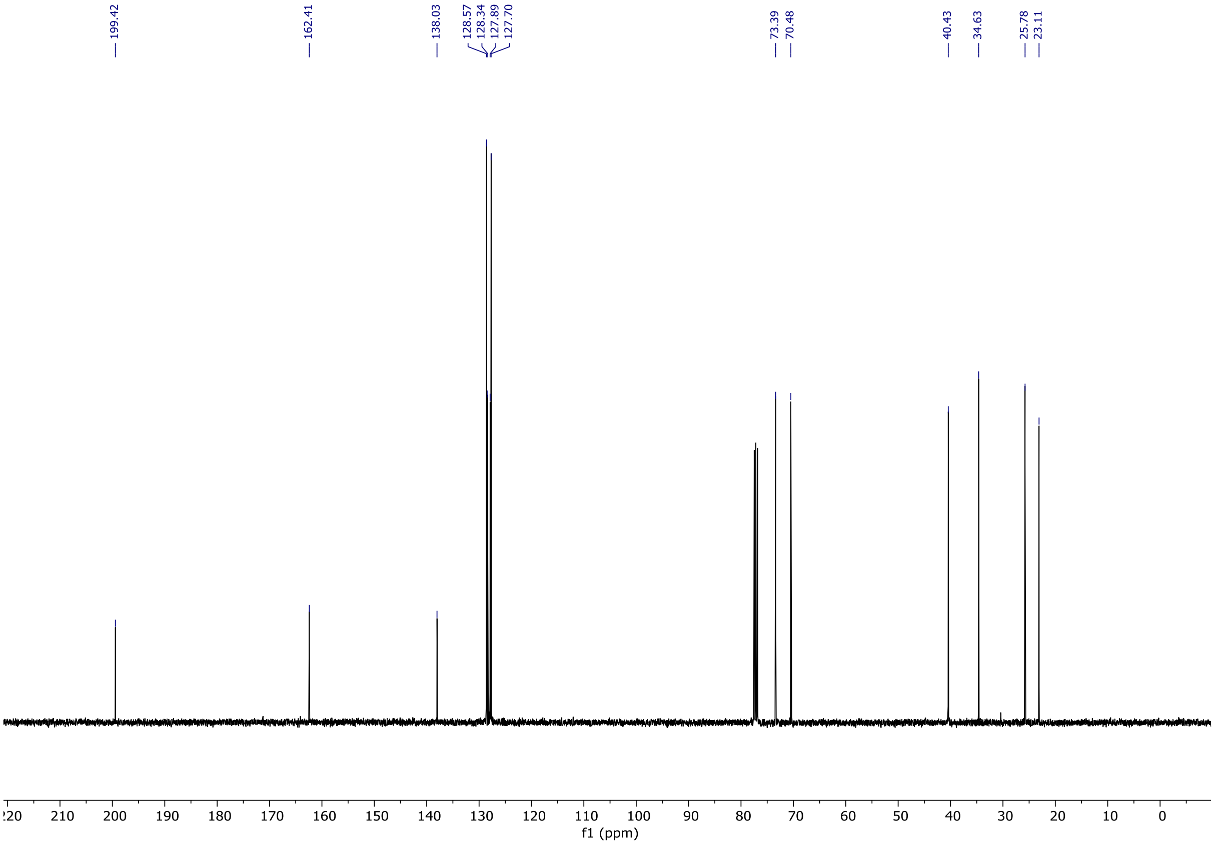

^{1}H NMR (500 MHz, Benzene-d_6) of **2b**

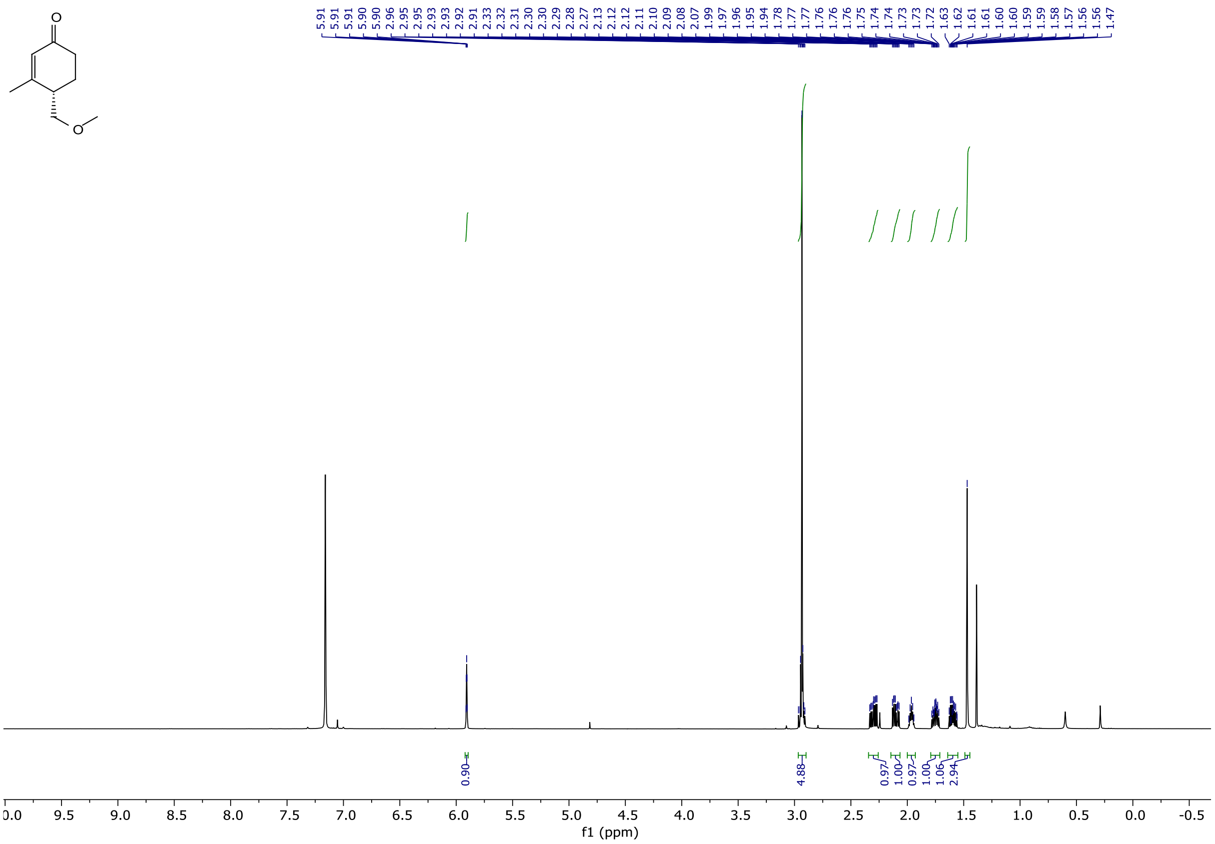

^{13}C NMR (126 MHz, Benzene-d_6) of **2b**

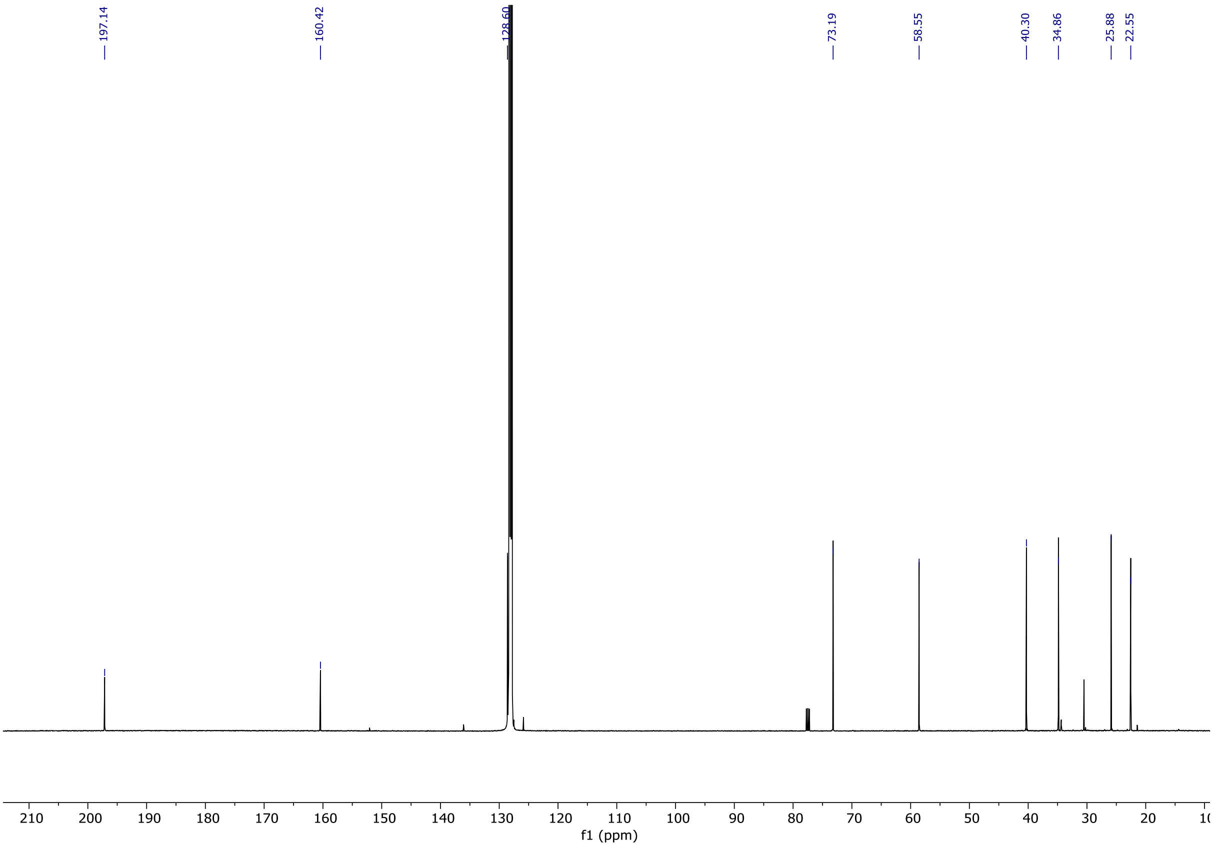

¹H NMR (400 MHz, CDCl$_3$) of **2c**

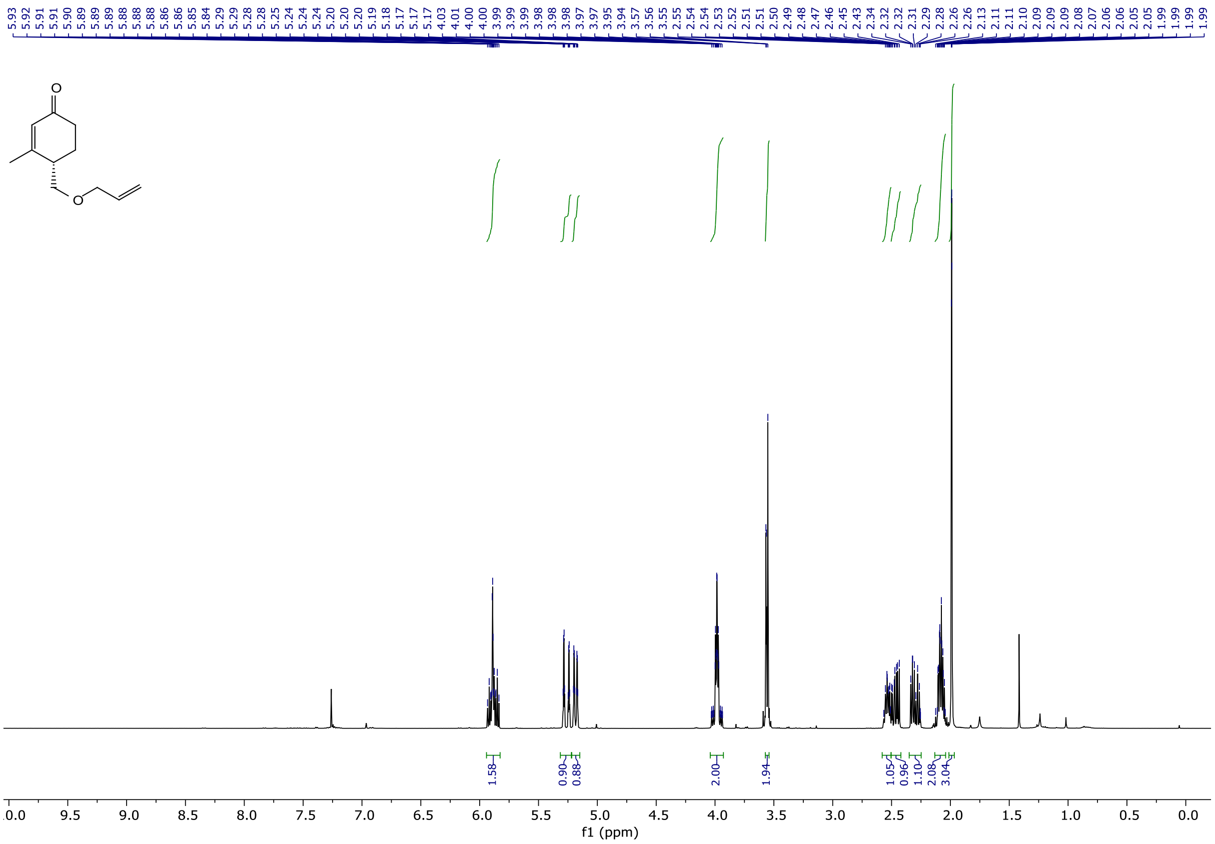

¹³C NMR (101 MHz, CDCl$_3$) of **2c**

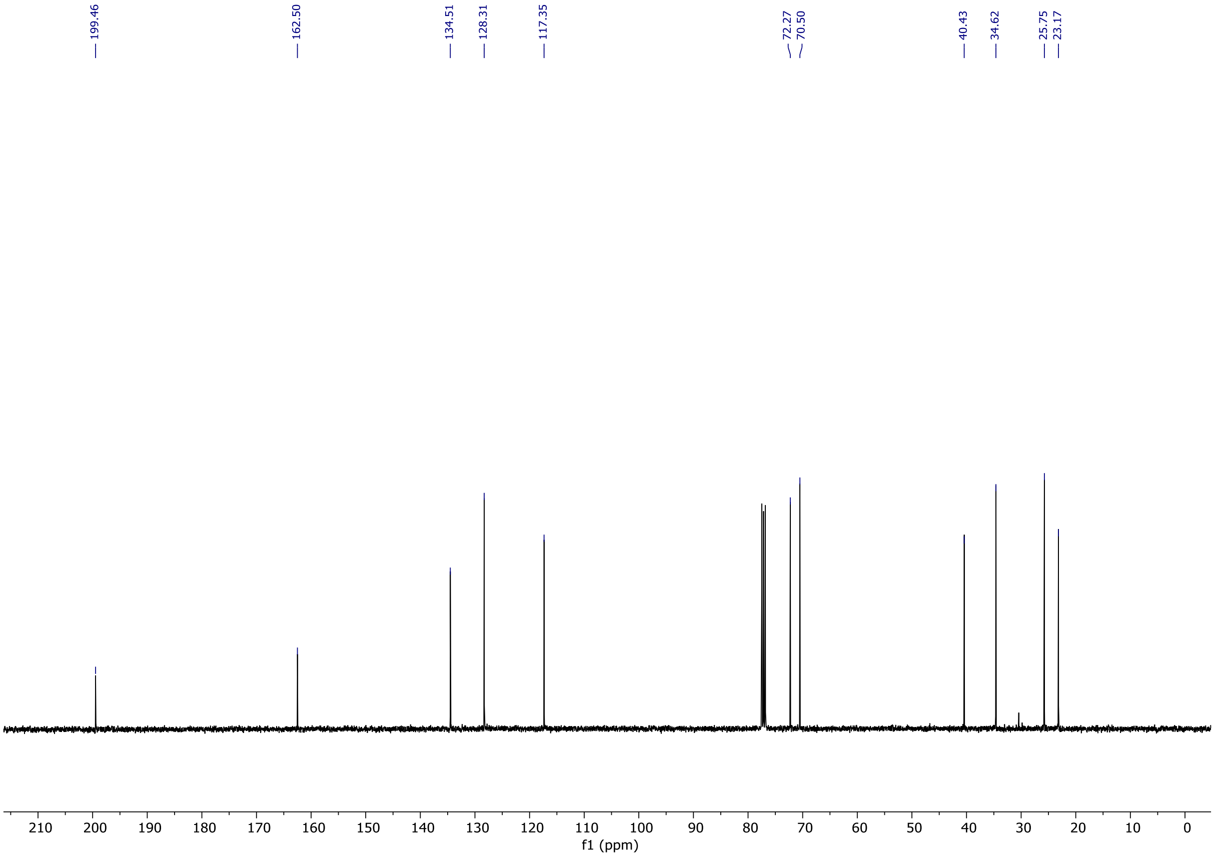

^{1}H NMR (500 MHz, $CDCl_3$) of **2d**

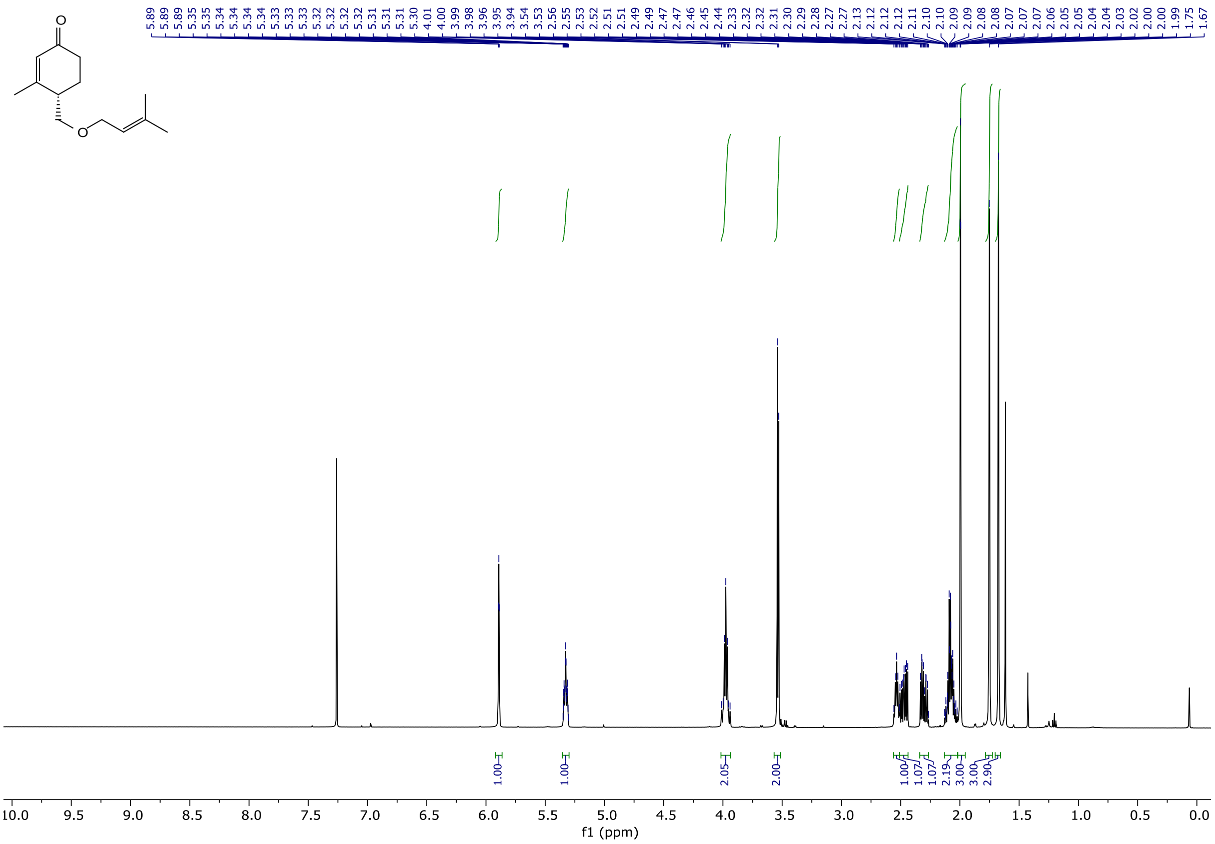

^{13}C NMR (126 MHz, $CDCl_3$) of **2d**

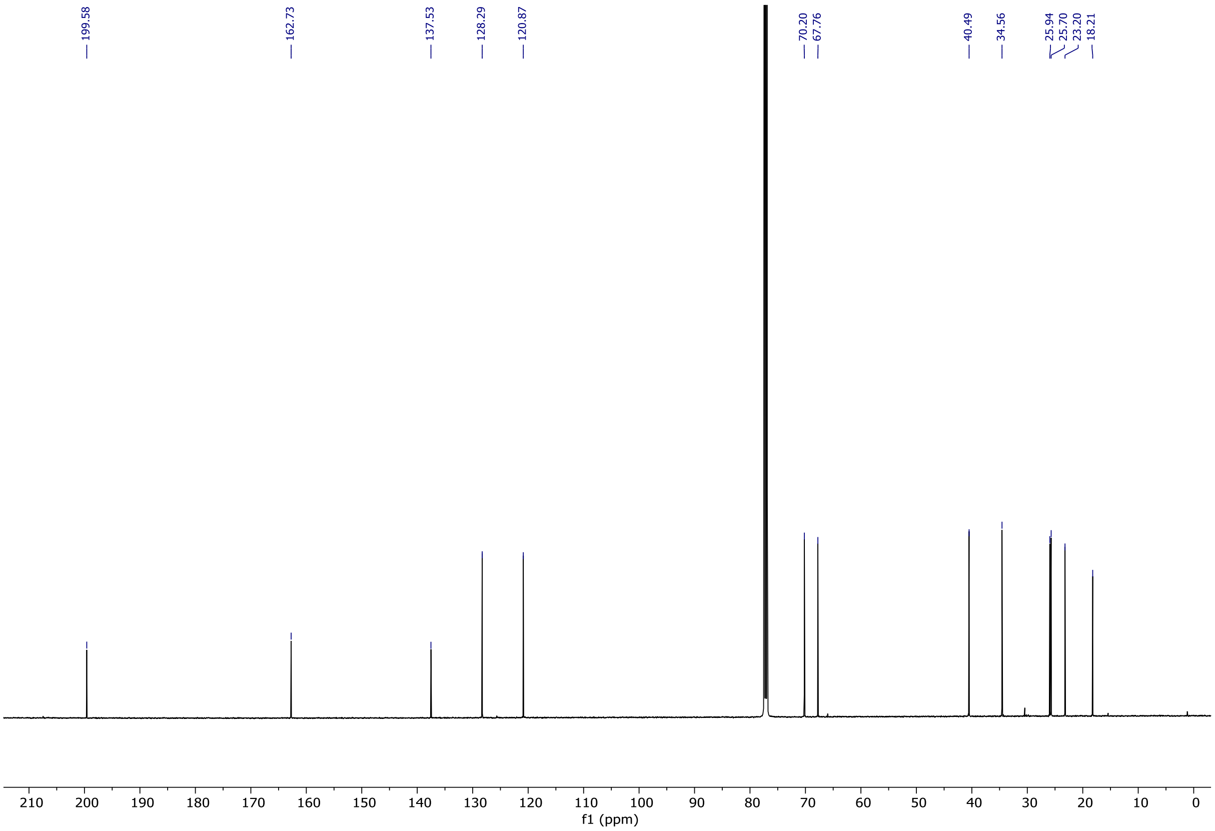

^{1}H NMR (400 MHz, Benzene-d_6) of **2e**

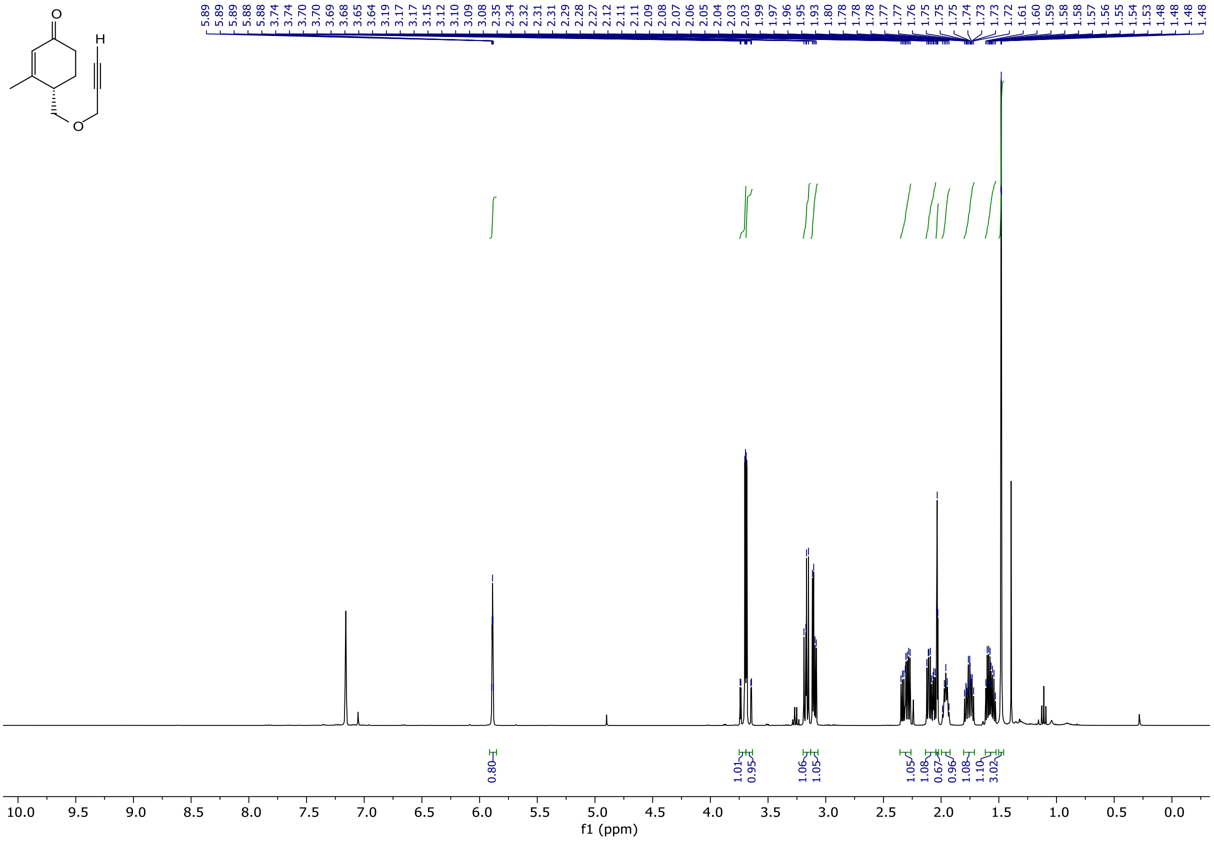

^{13}C NMR (101 MHz, Benzene-d_6) of **2e**

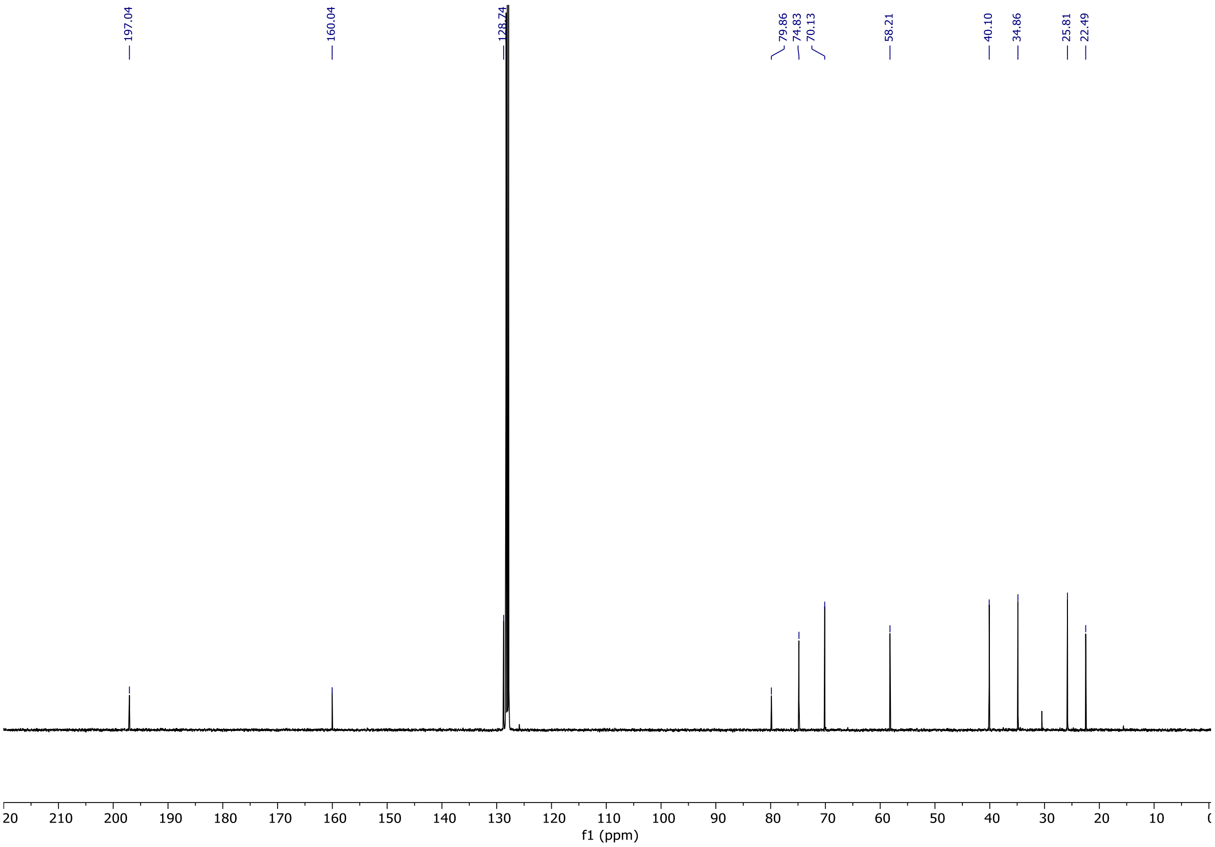

^{1}H NMR (500 MHz, Acetone-d_6) of **2f**

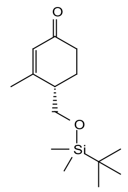

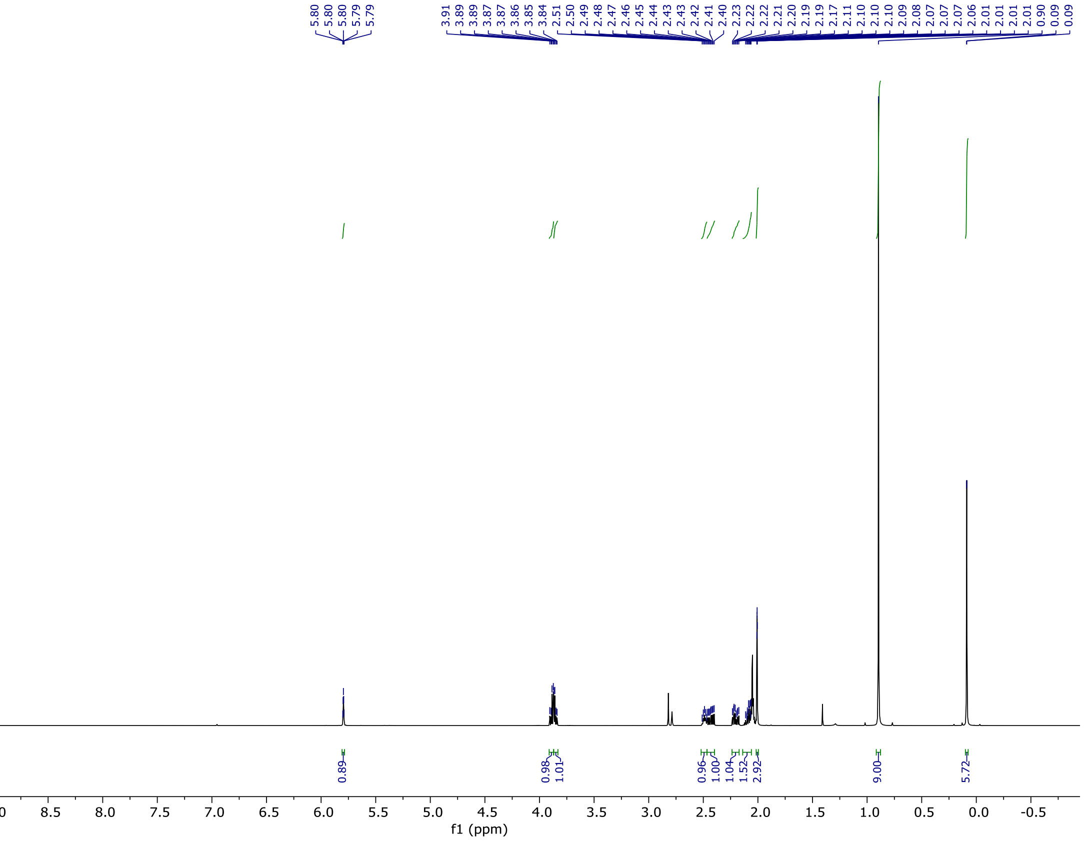

^{13}C NMR (126 MHz, Acetone-d_6) of **2f**

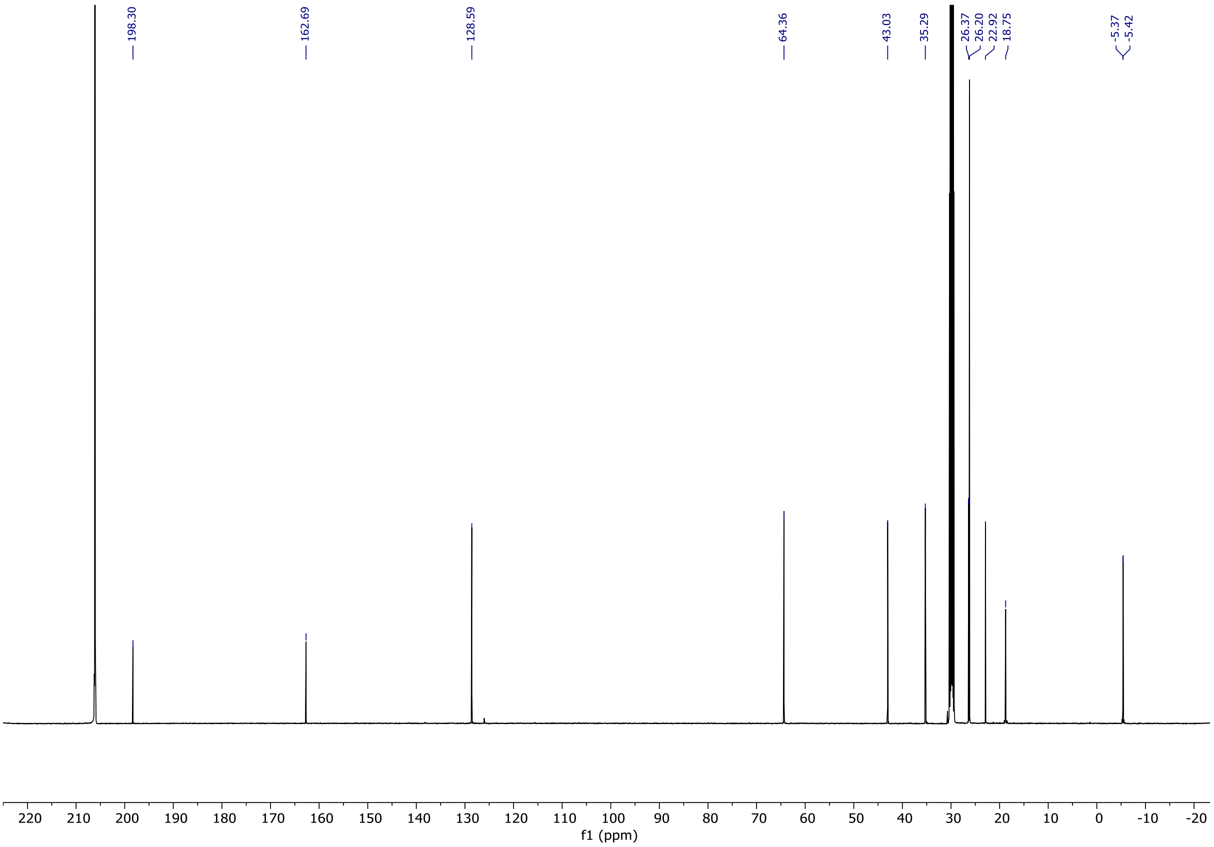

^{1}H NMR (400 MHz, $CDCl_3$) of **2g**

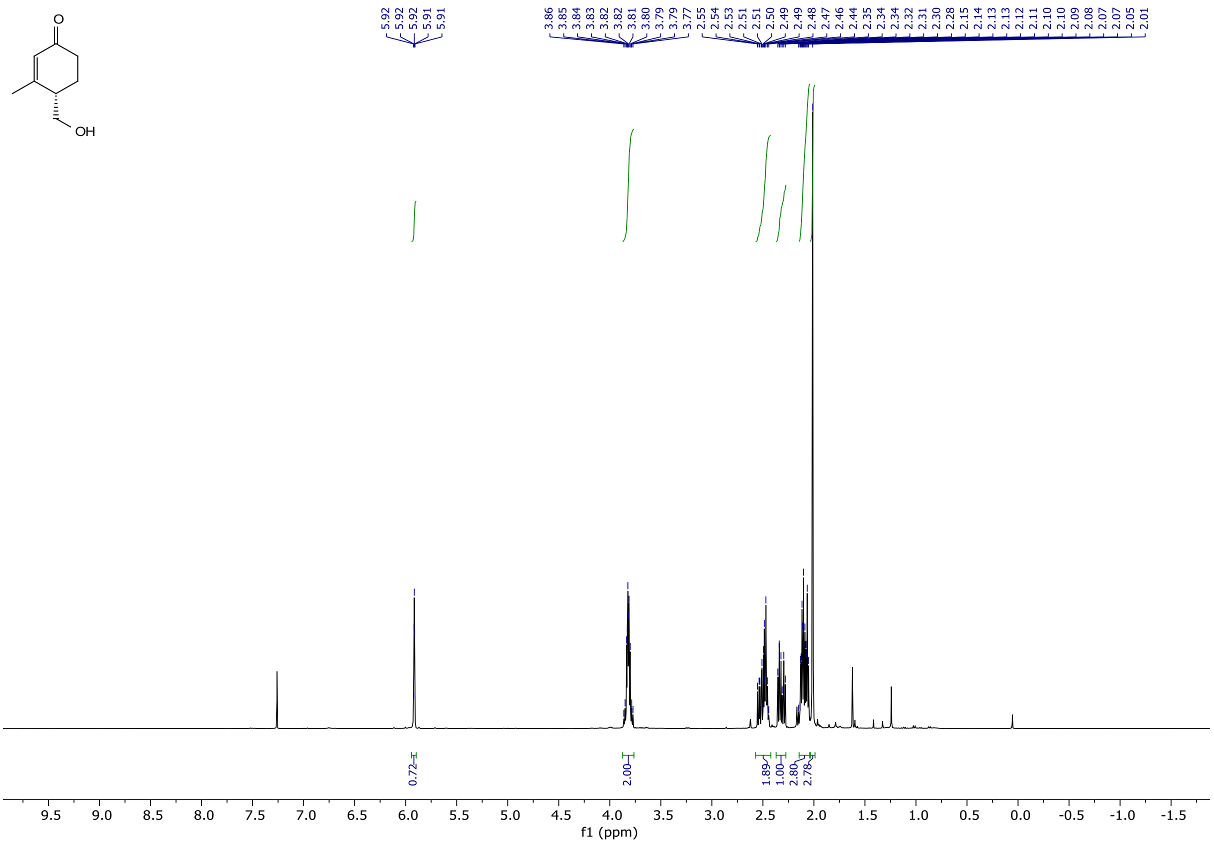

^{13}C NMR (101 MHz, $CDCl_3$) of **2g**

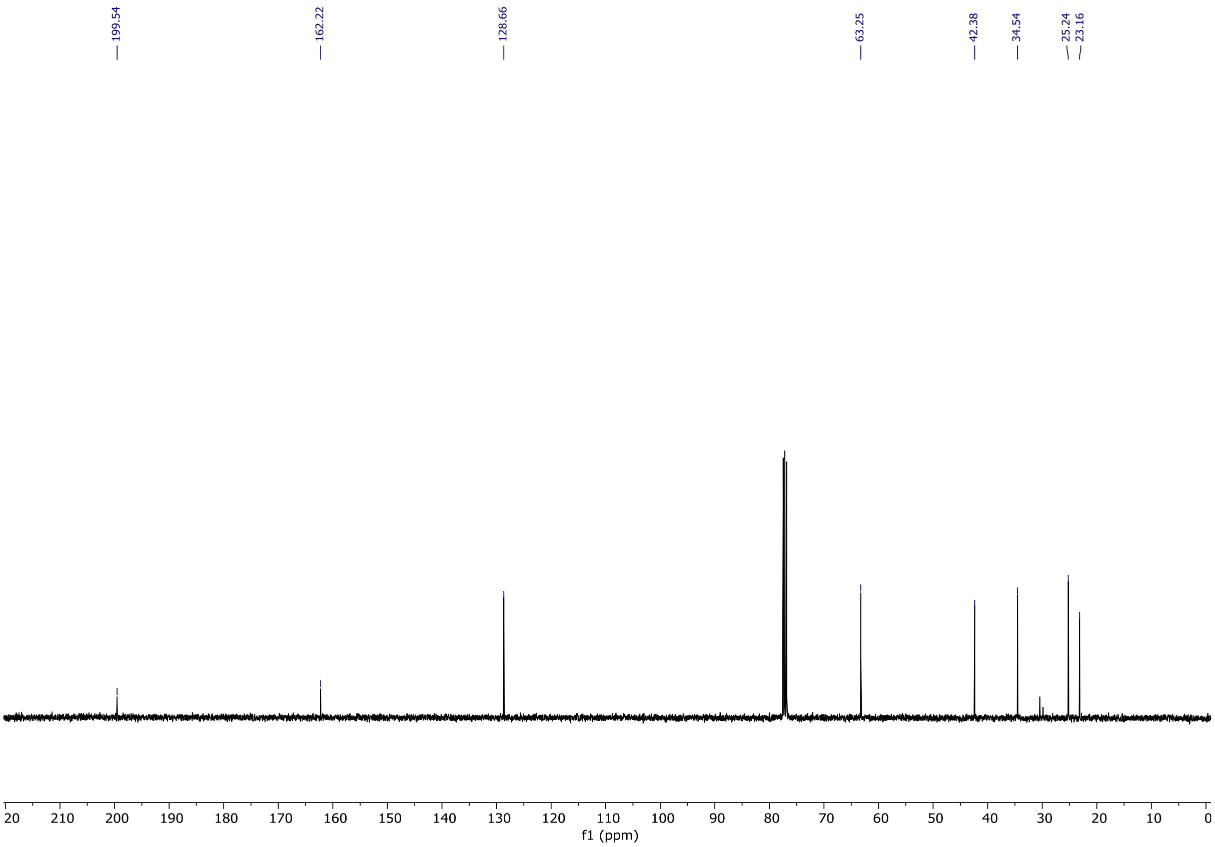

^{1}**H NMR** (400 MHz, $CDCl_3$) of **2h**

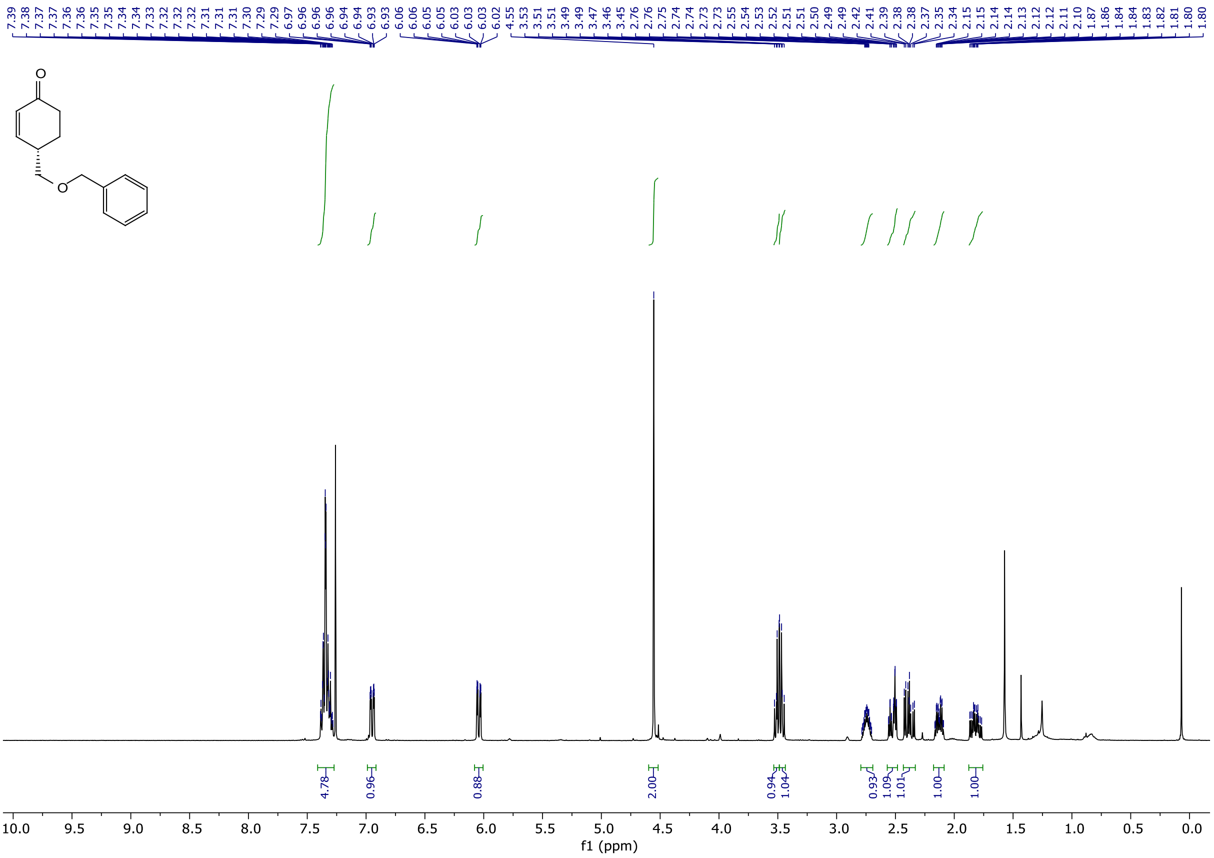

^{13}**C NMR** (101 MHz, $CDCl_3$) of **2h**

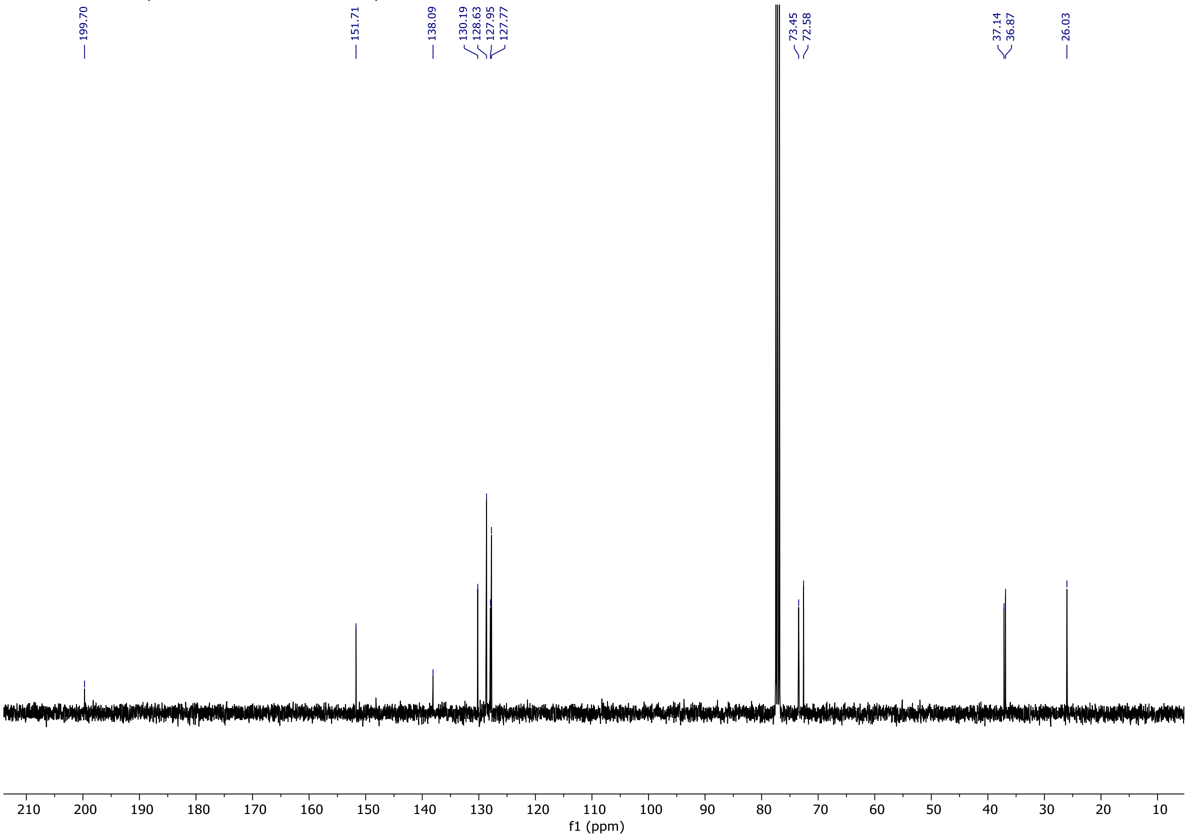

^{1}H NMR (500 MHz, Acetone-d_6) of **2i**

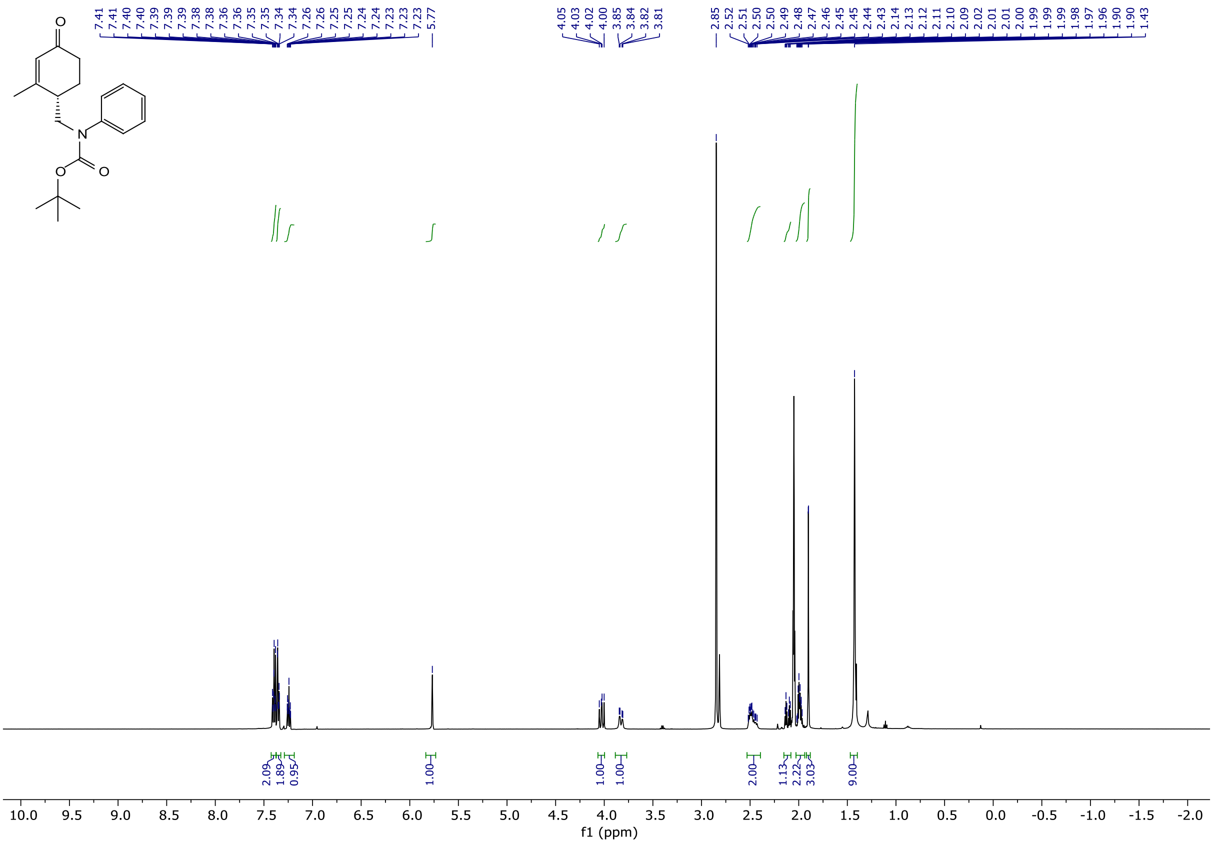

^{13}C NMR (126 MHz, Acetone-d_6) of **2i**

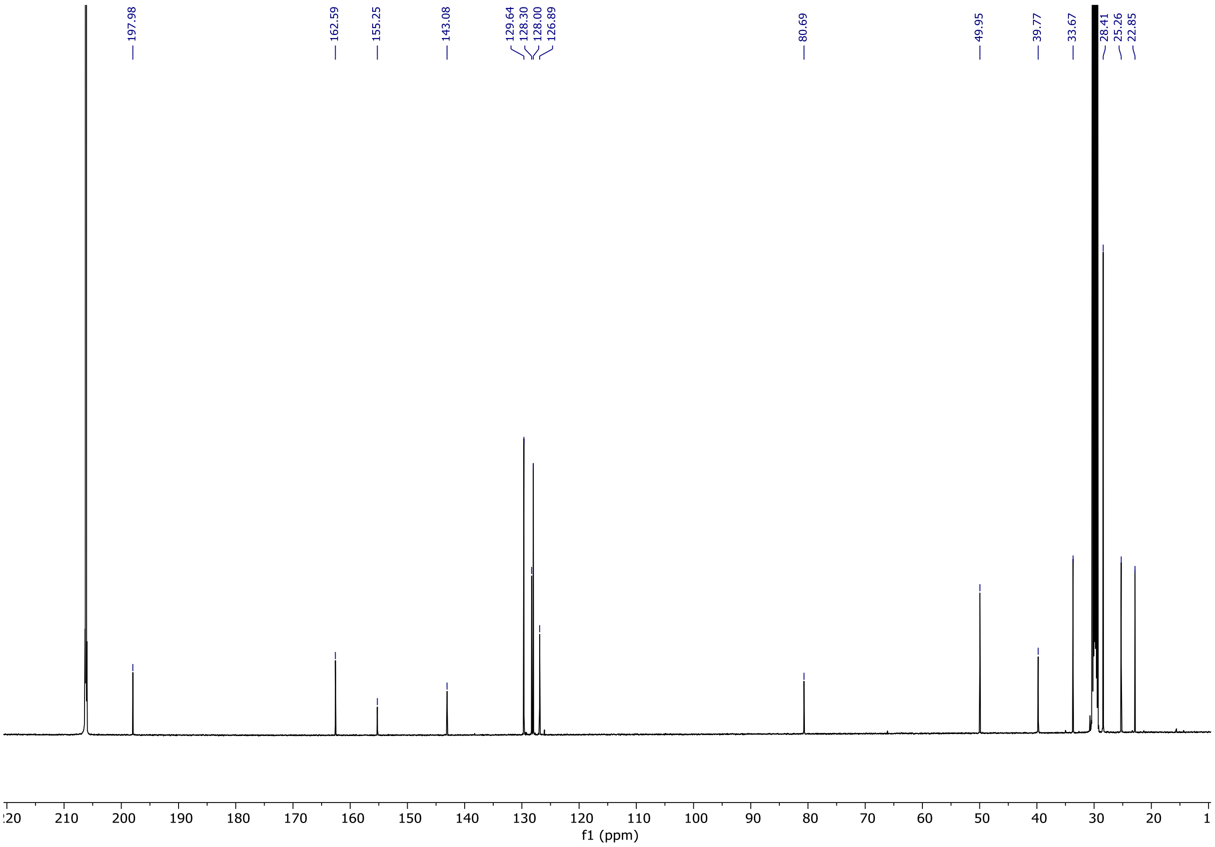

^{1}H NMR (500 MHz, Toluene-d_8, 363 K) of **2j**

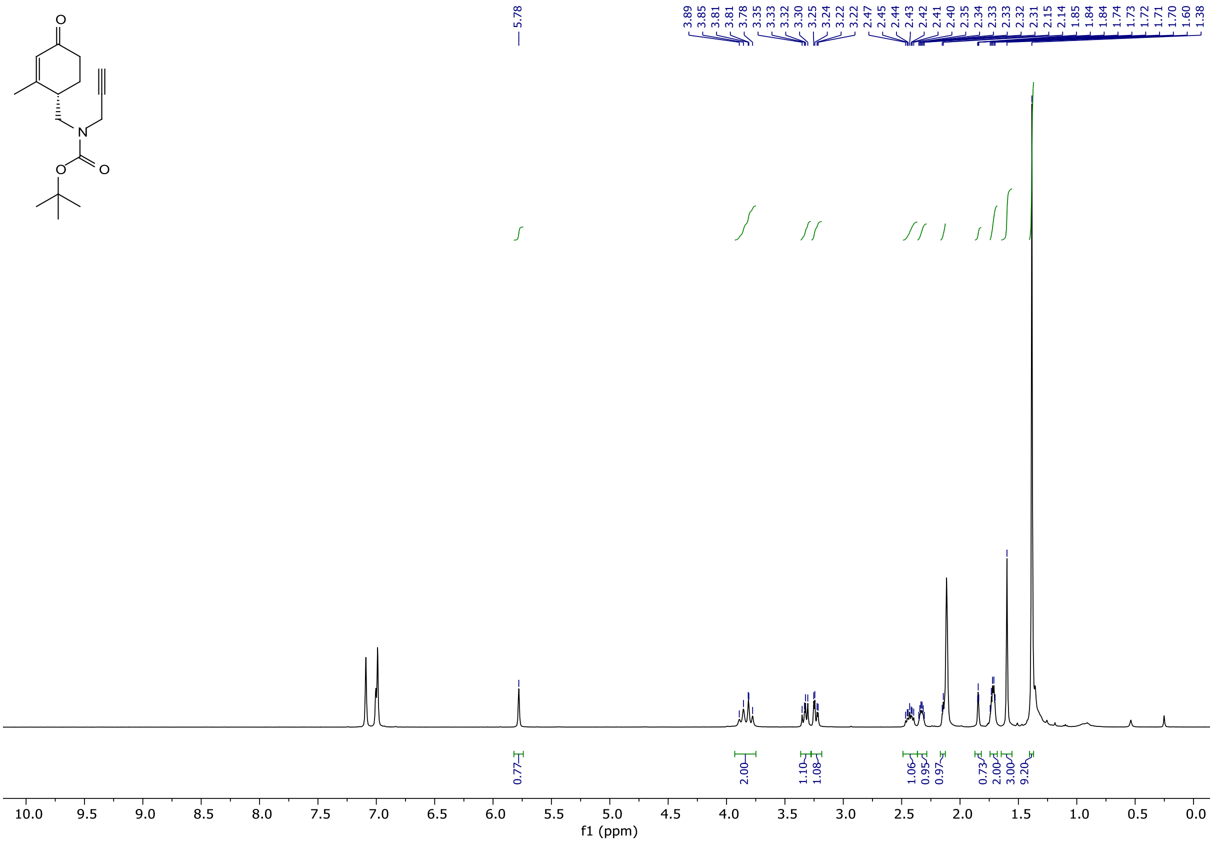

^{13}C NMR (126 MHz, Toluene-d_8, 363 K) of **2j**

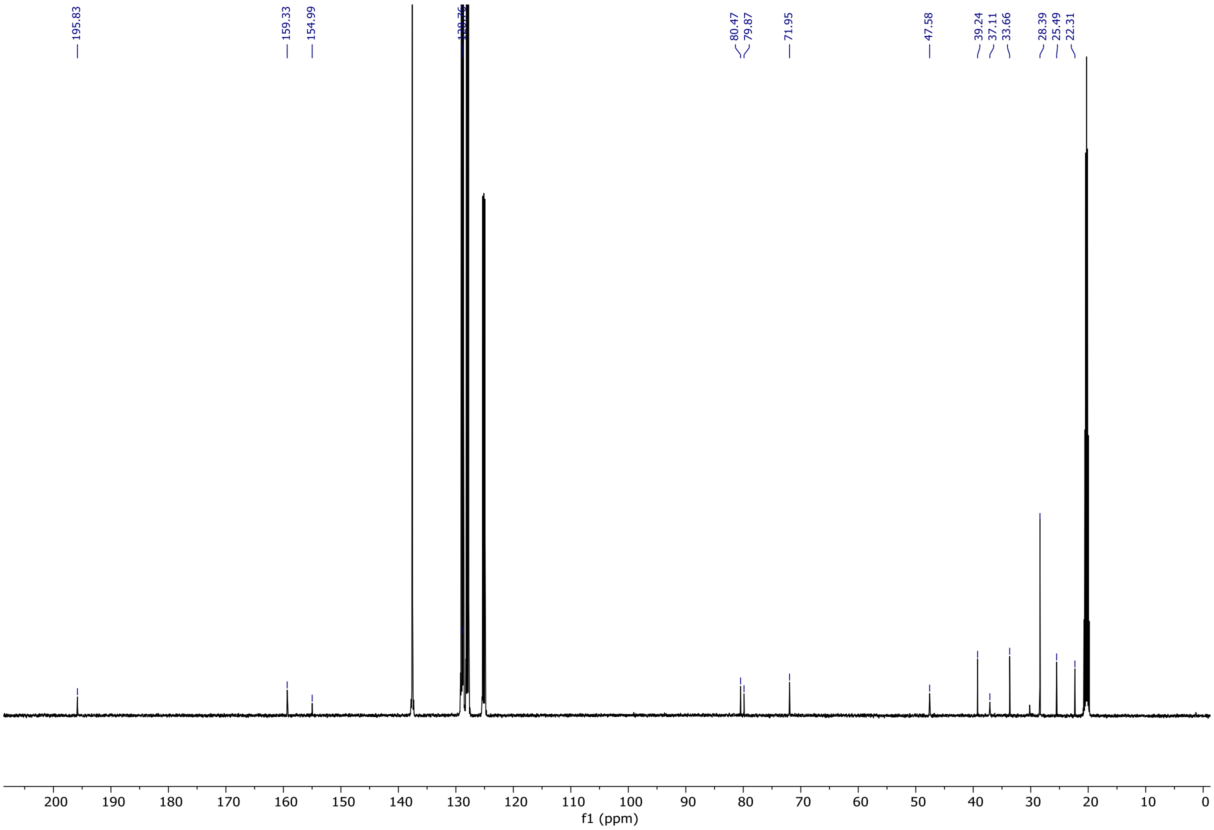

^{1}H NMR (400 MHz, Methanol-d_4) of **2k**

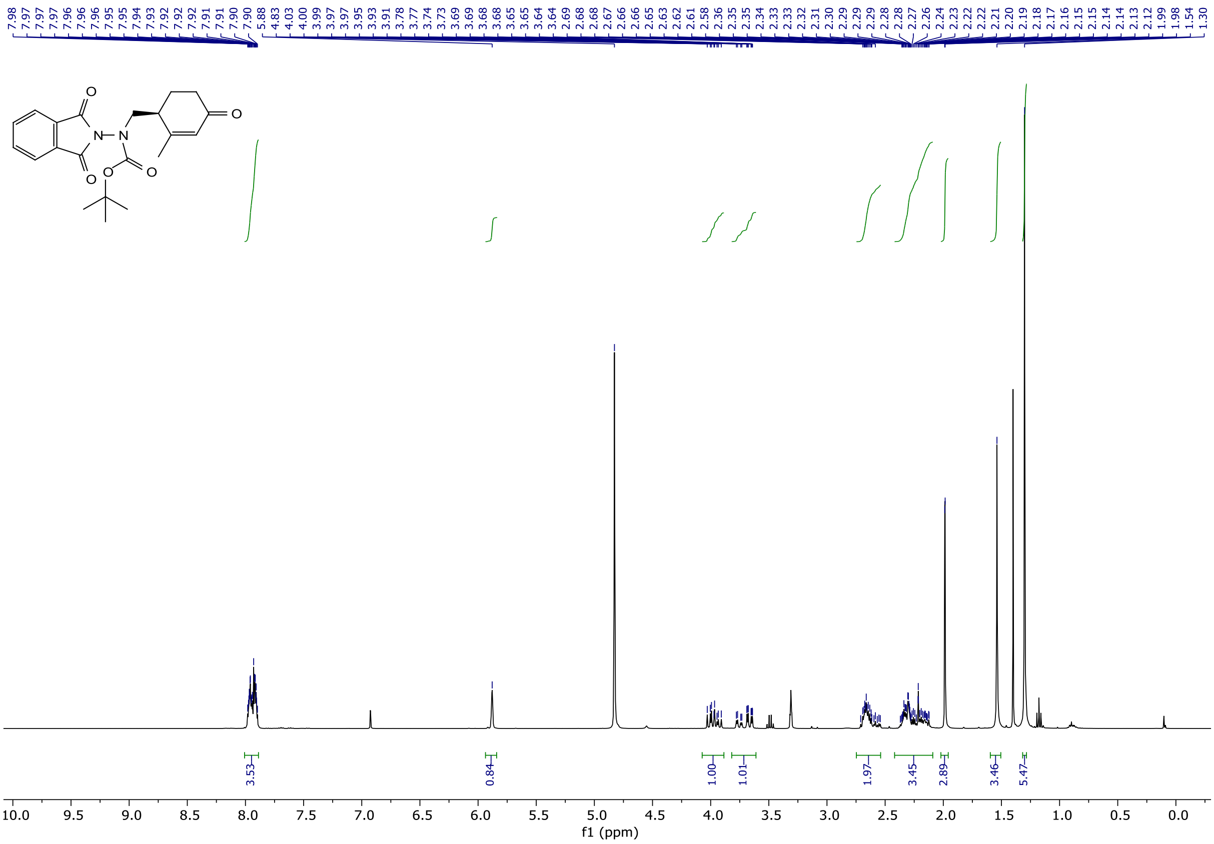

^{13}C NMR (101 MHz, Methanol-d_4) of **2k**

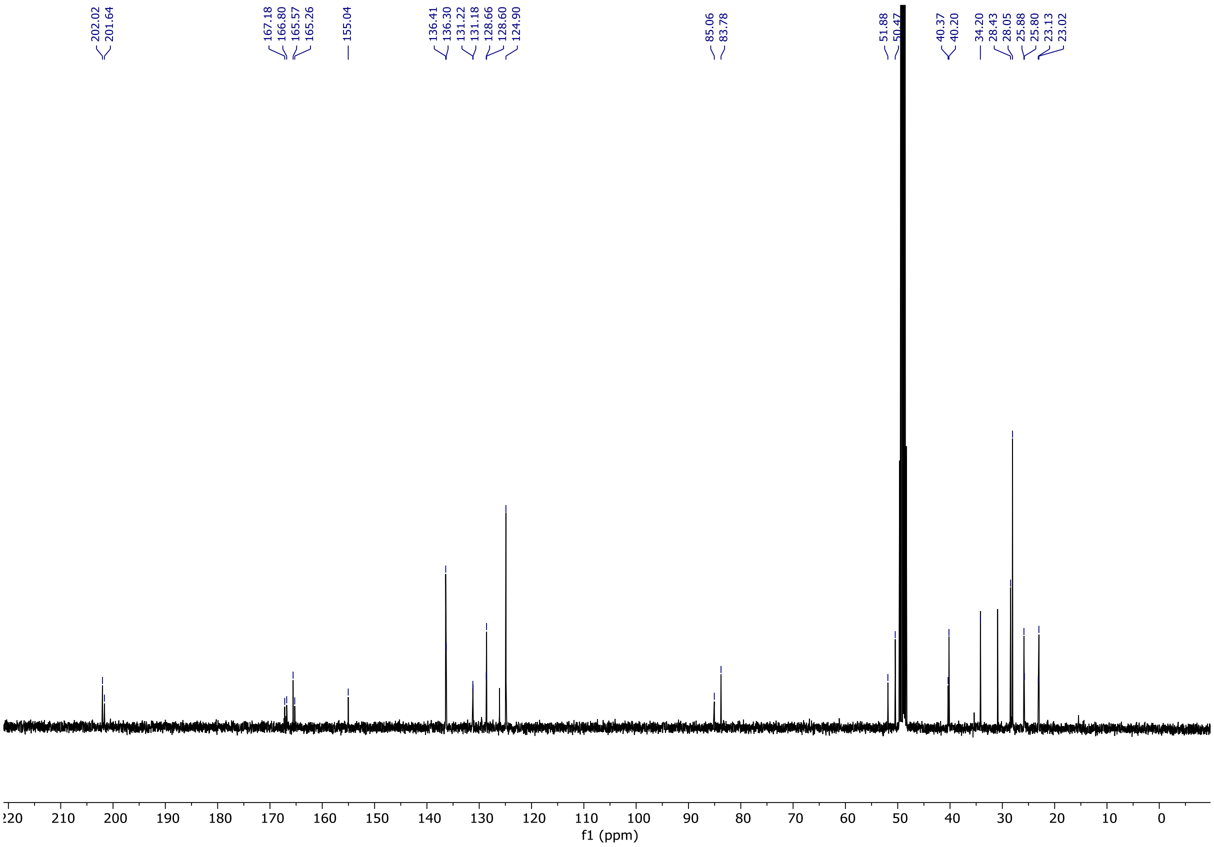

^{1}H NMR (500 MHz, Acetone-d_6) of **2l**

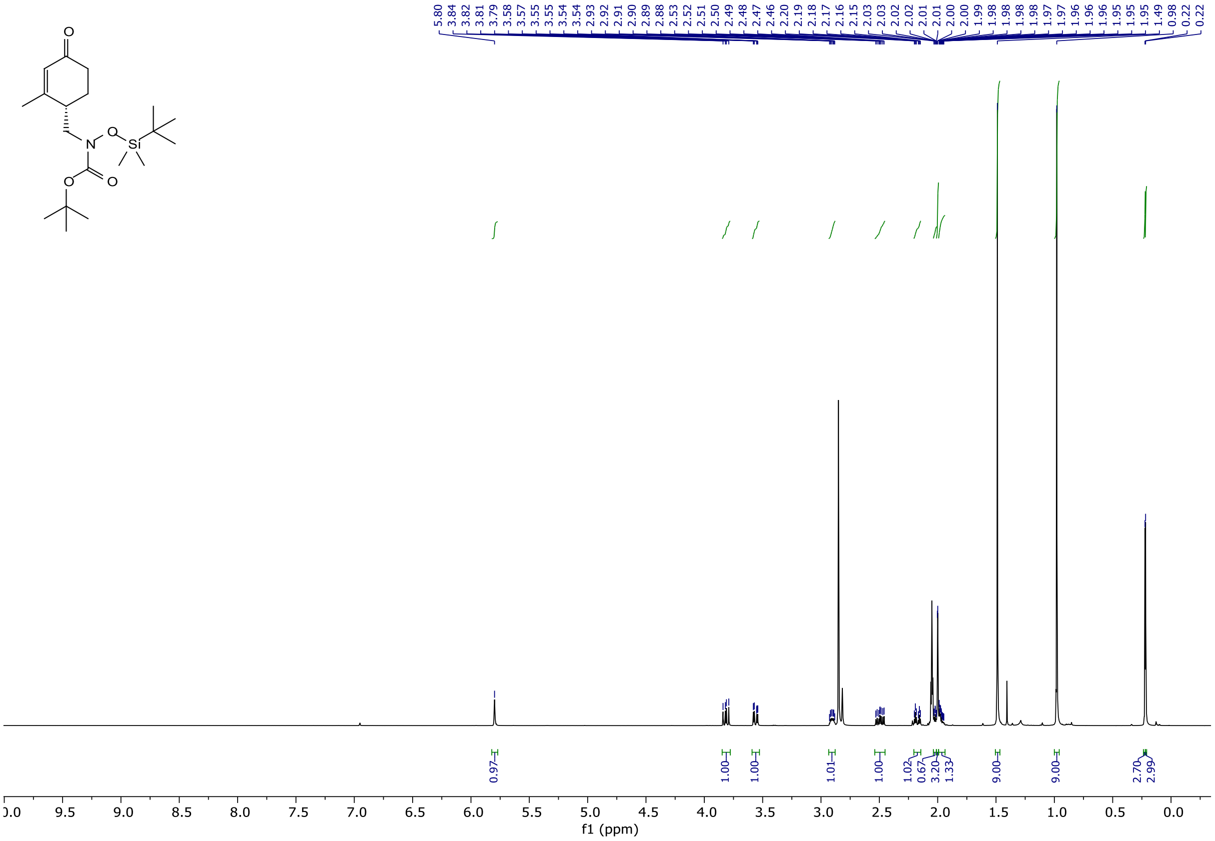

^{13}C NMR (126 MHz, Acetone-d_6) of **2l**

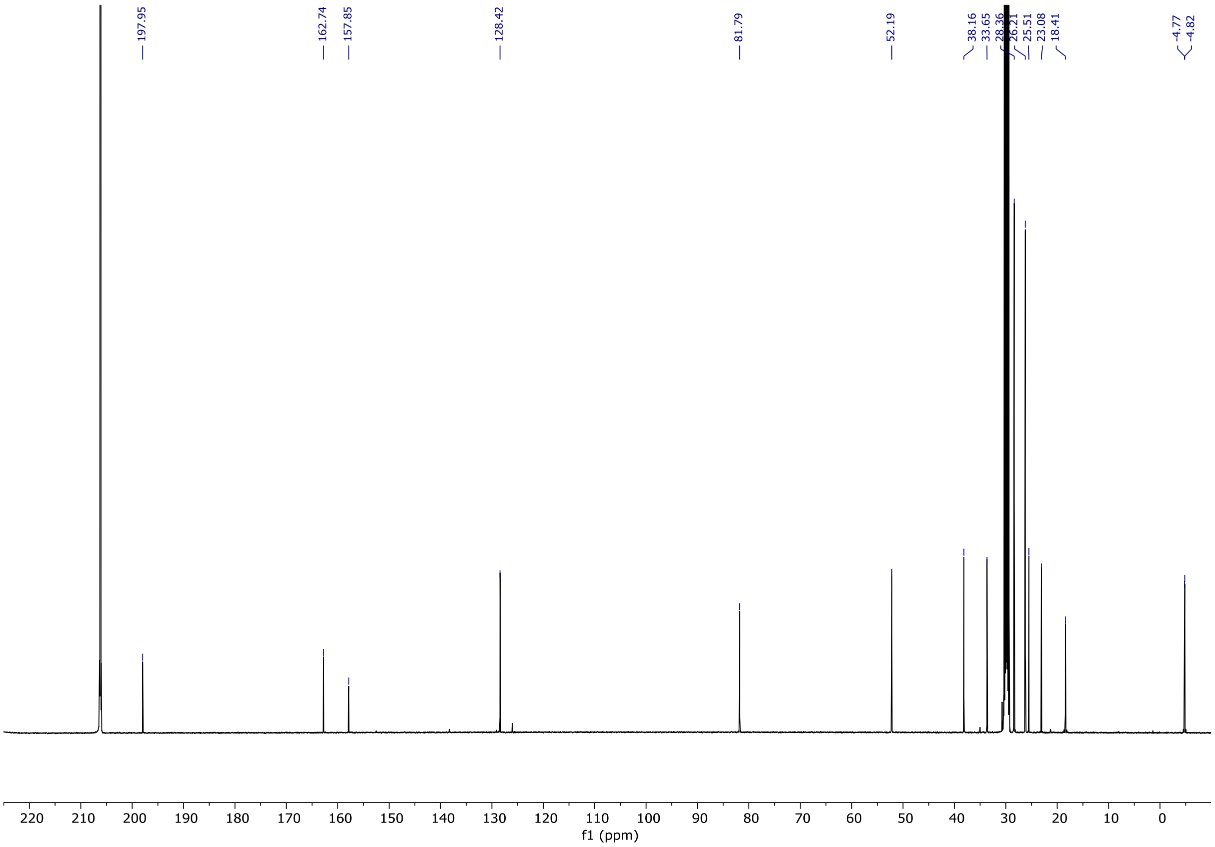

^{1}H NMR (500 MHz, $CDCl_3$) of **2m**

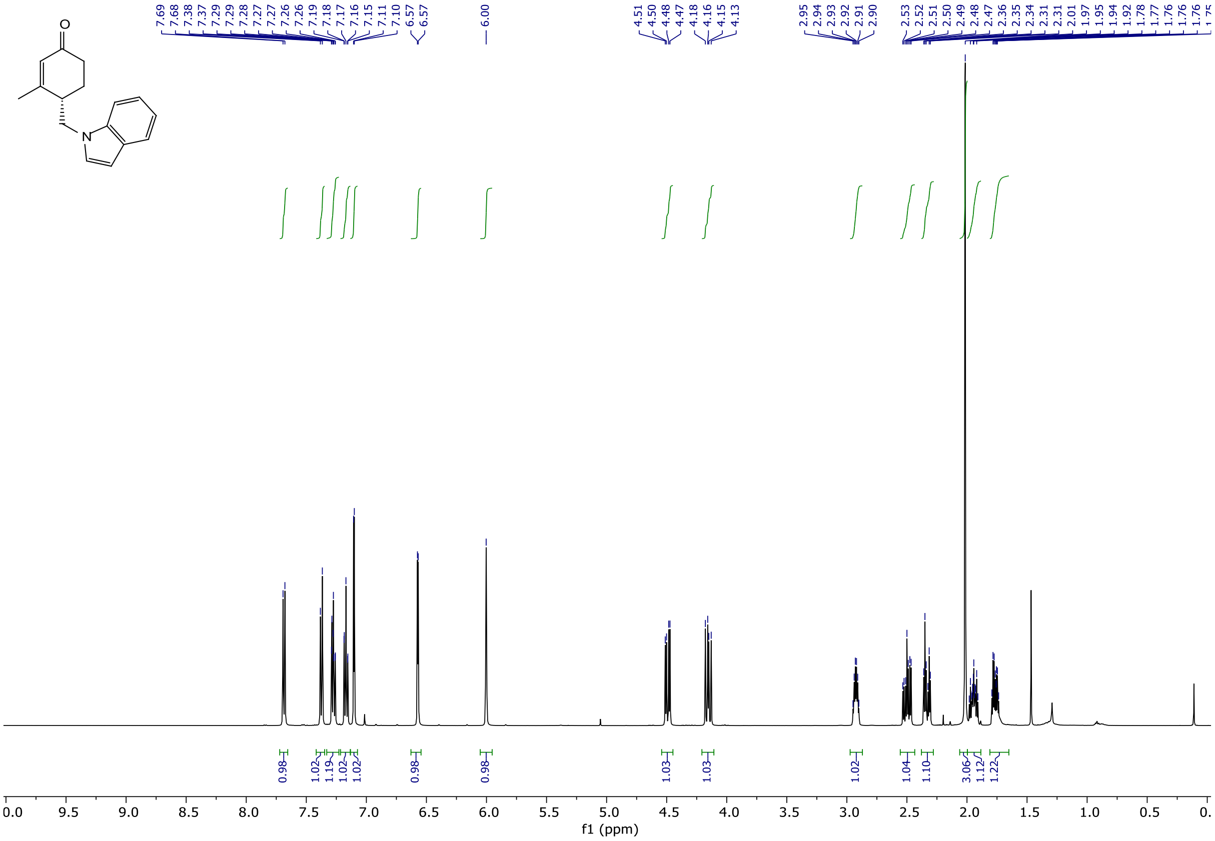

^{13}C NMR (126 MHz, $CDCl_3$) of **2m**

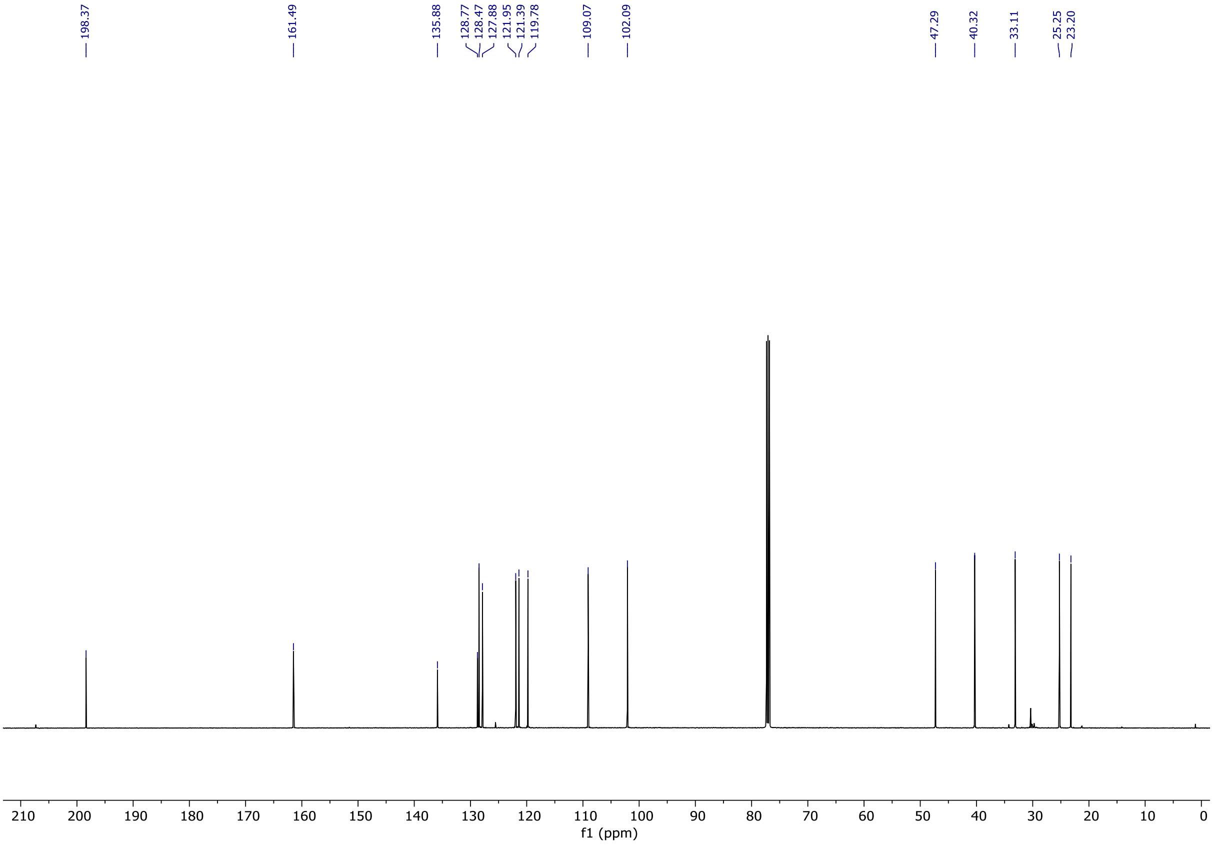

^{1}H NMR (400 MHz, CDCl$_3$) of **2n**

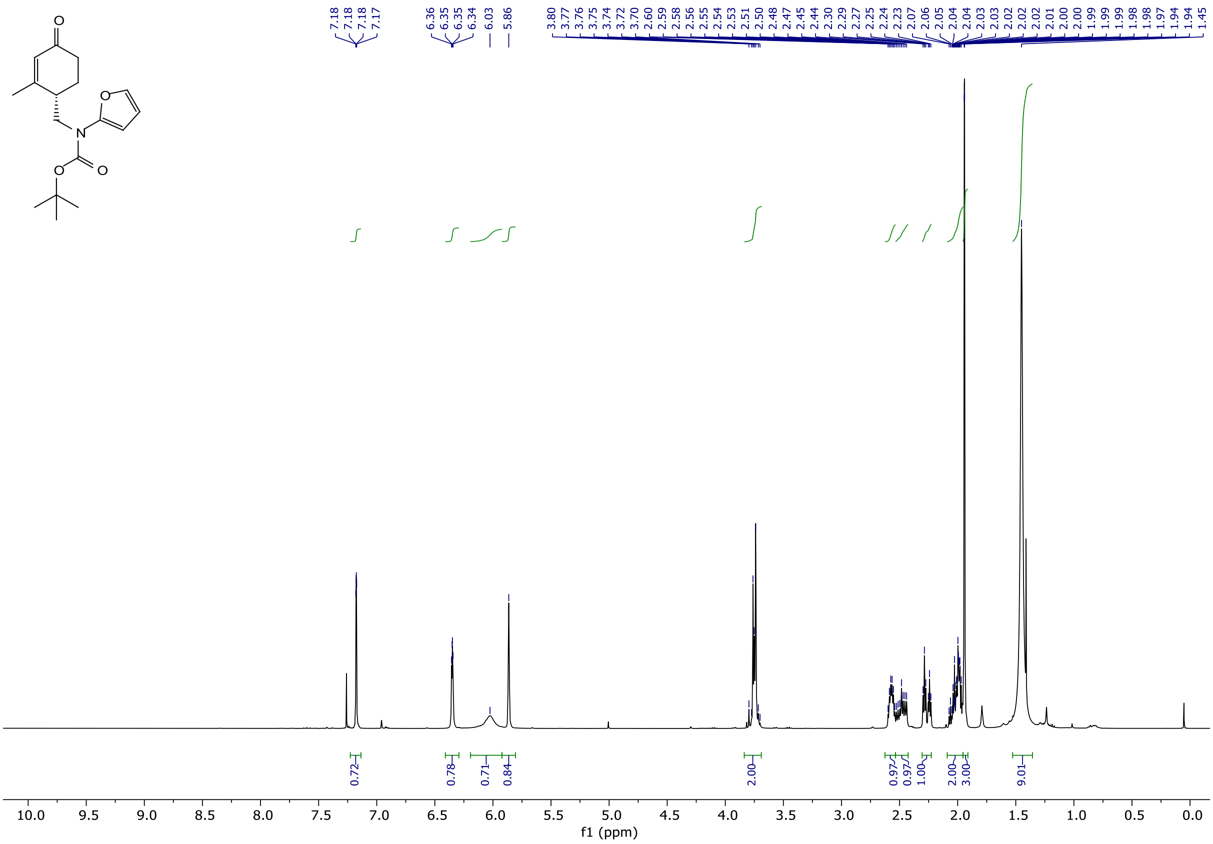

^{13}C NMR (101 MHz, CDCl$_3$) of **2n**

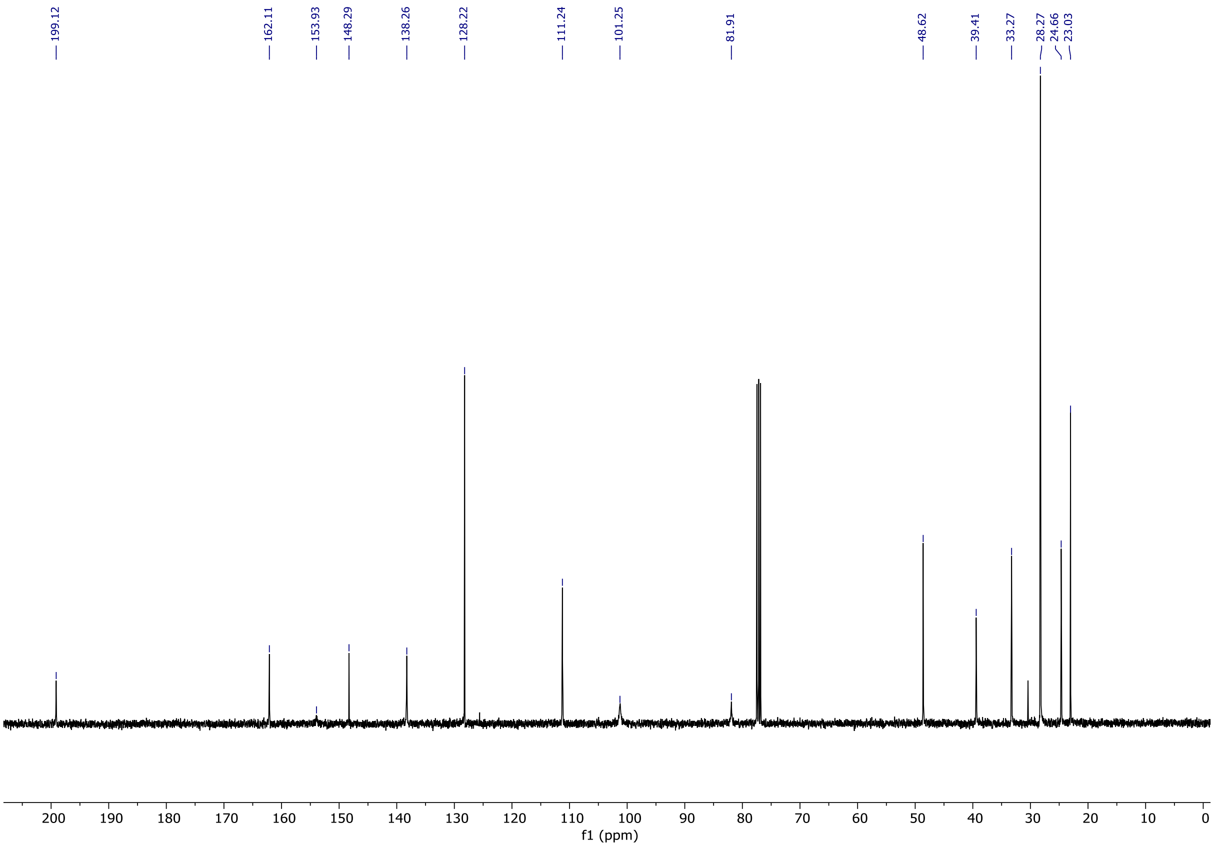

^{1}H NMR (400 MHz, $CDCl_3$) of **2o**

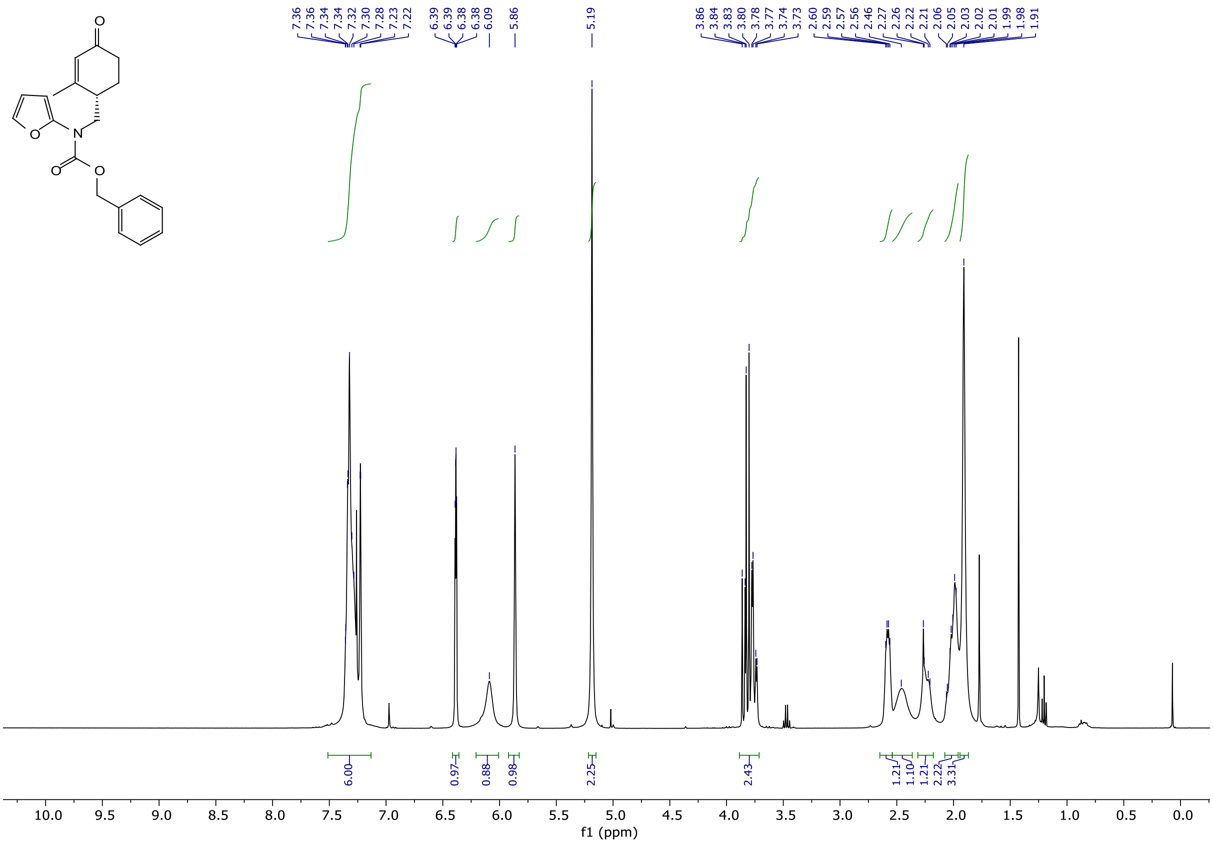

^{13}C NMR (101 MHz, $CDCl_3$) of **2o**

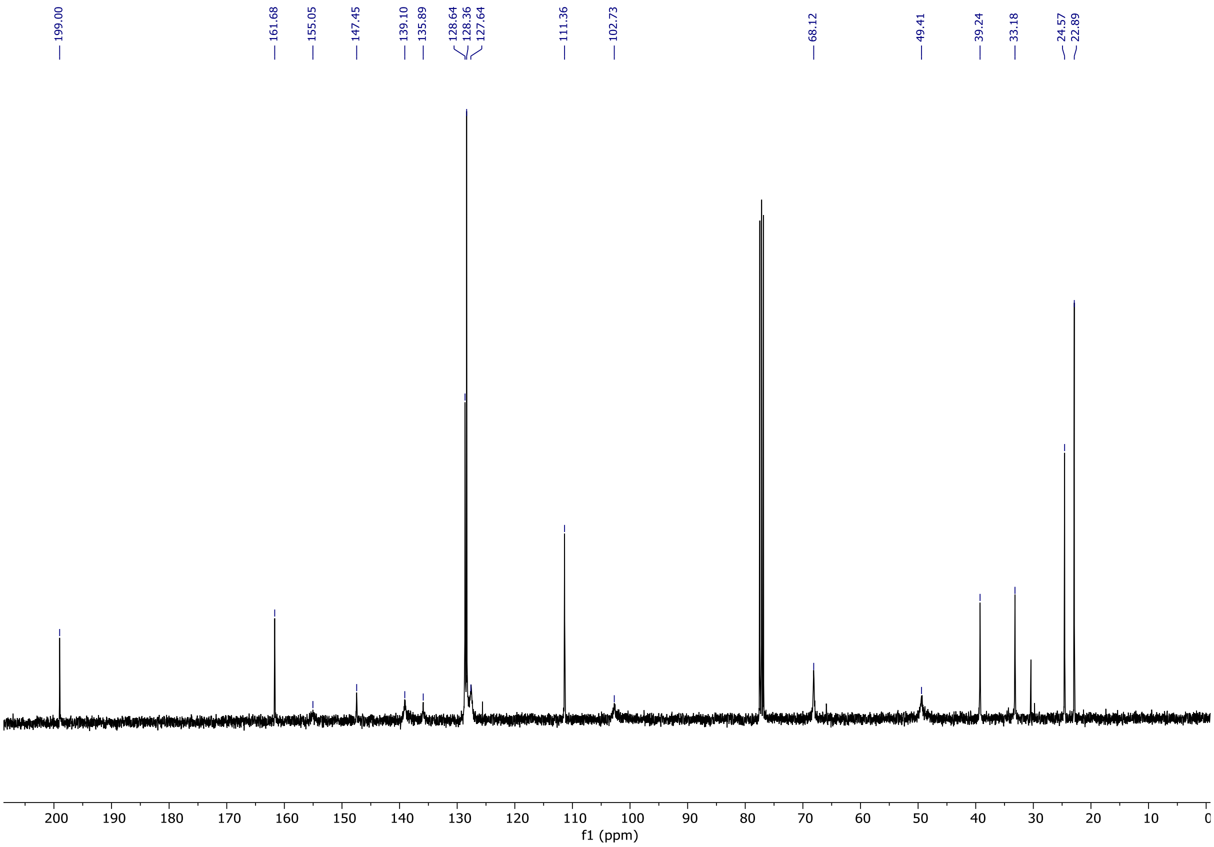

^{1}H NMR (500 MHz, $CDCl_3$) of **2p**

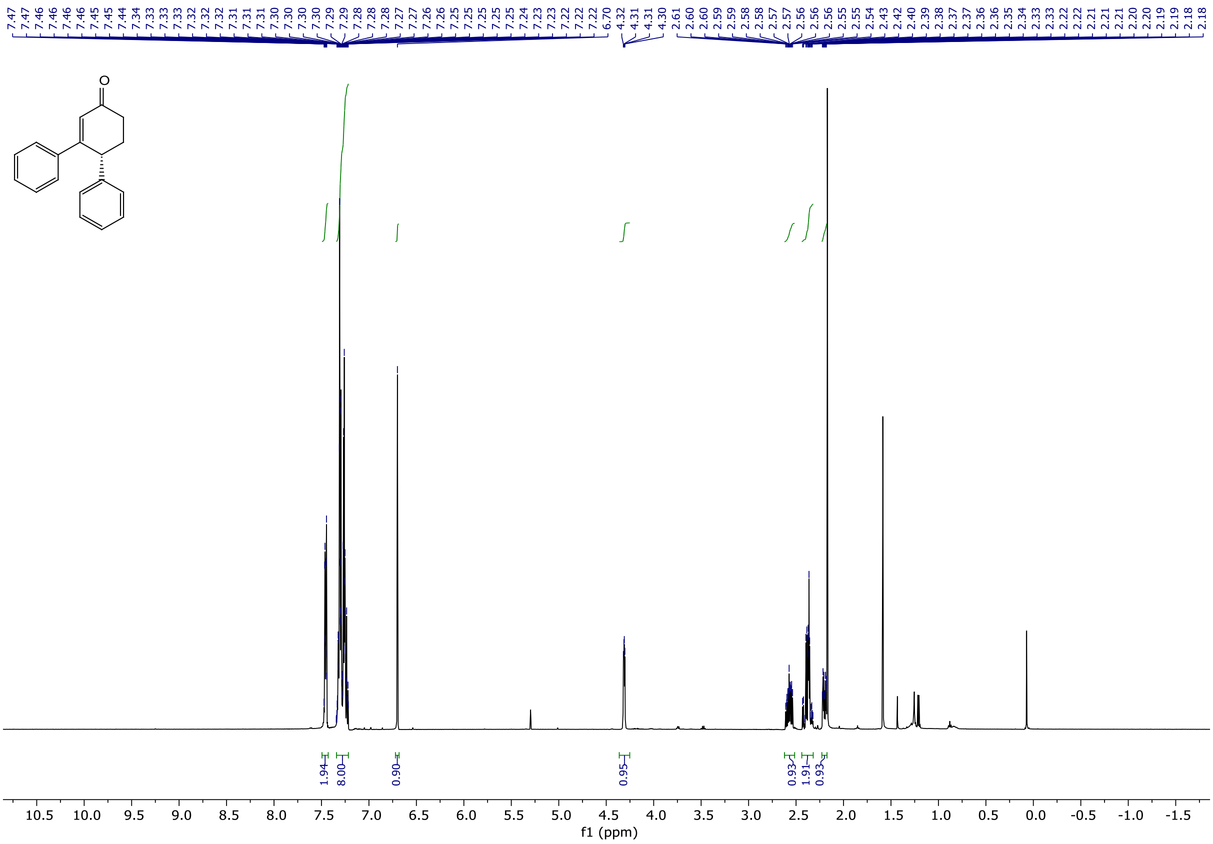

^{13}C NMR (126 MHz, $CDCl_3$) of **2p**

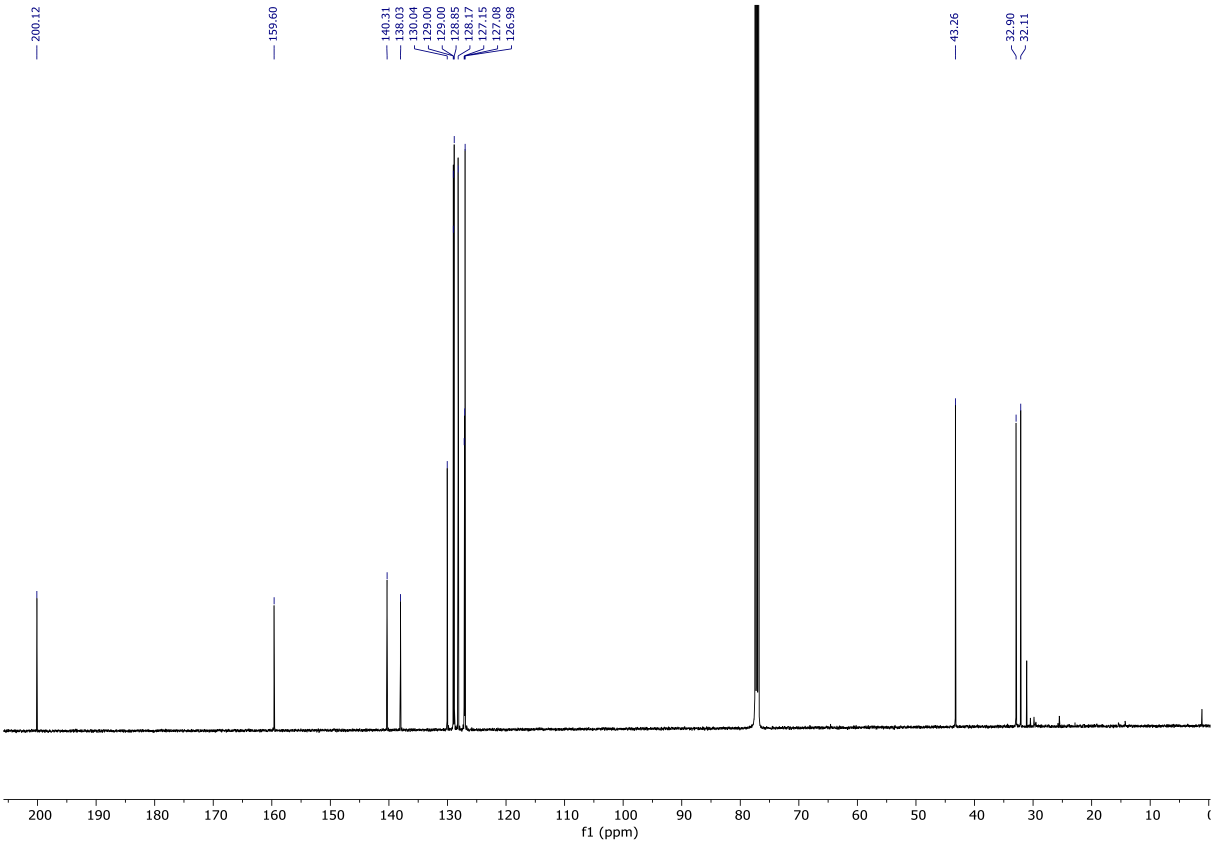

^{1}H NMR (400 MHz, $CDCl_3$) of **2q**

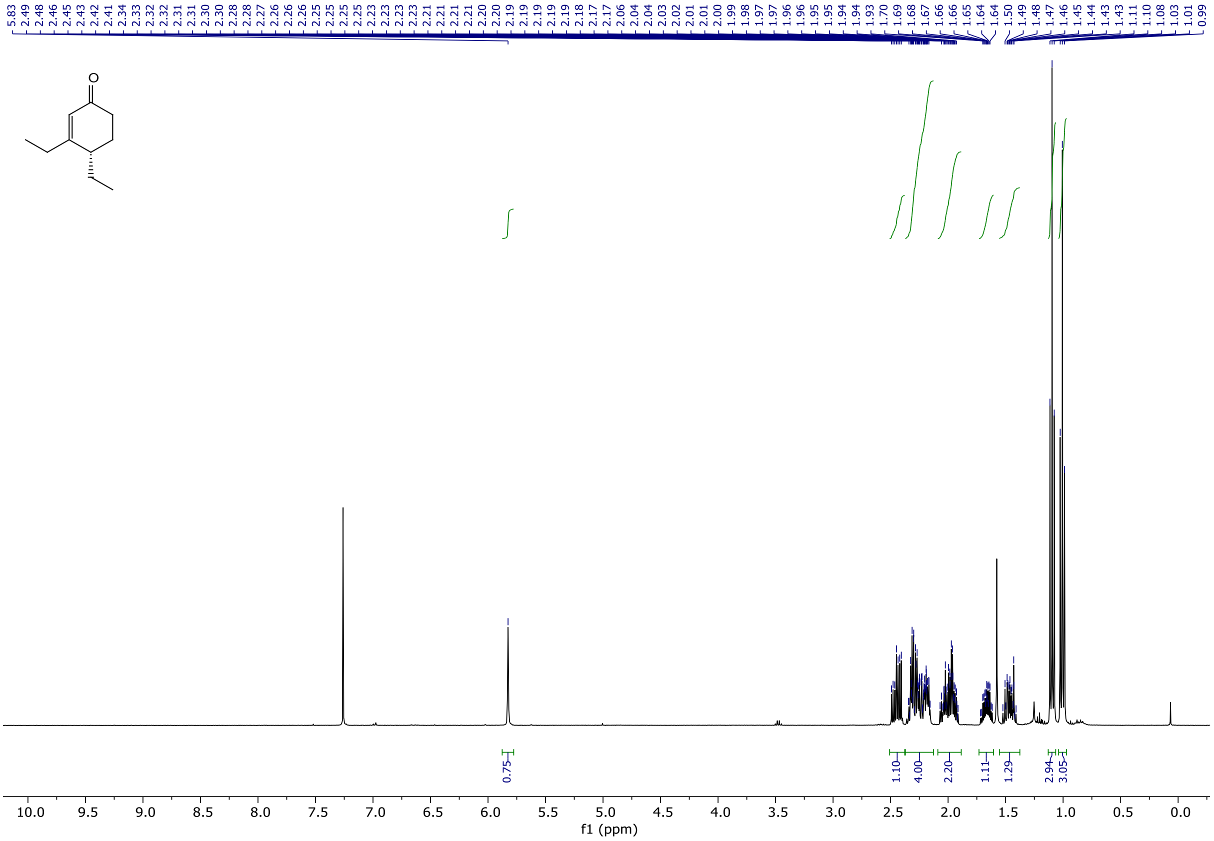

^{13}C NMR (126 MHz, $CDCl_3$) of **2q**

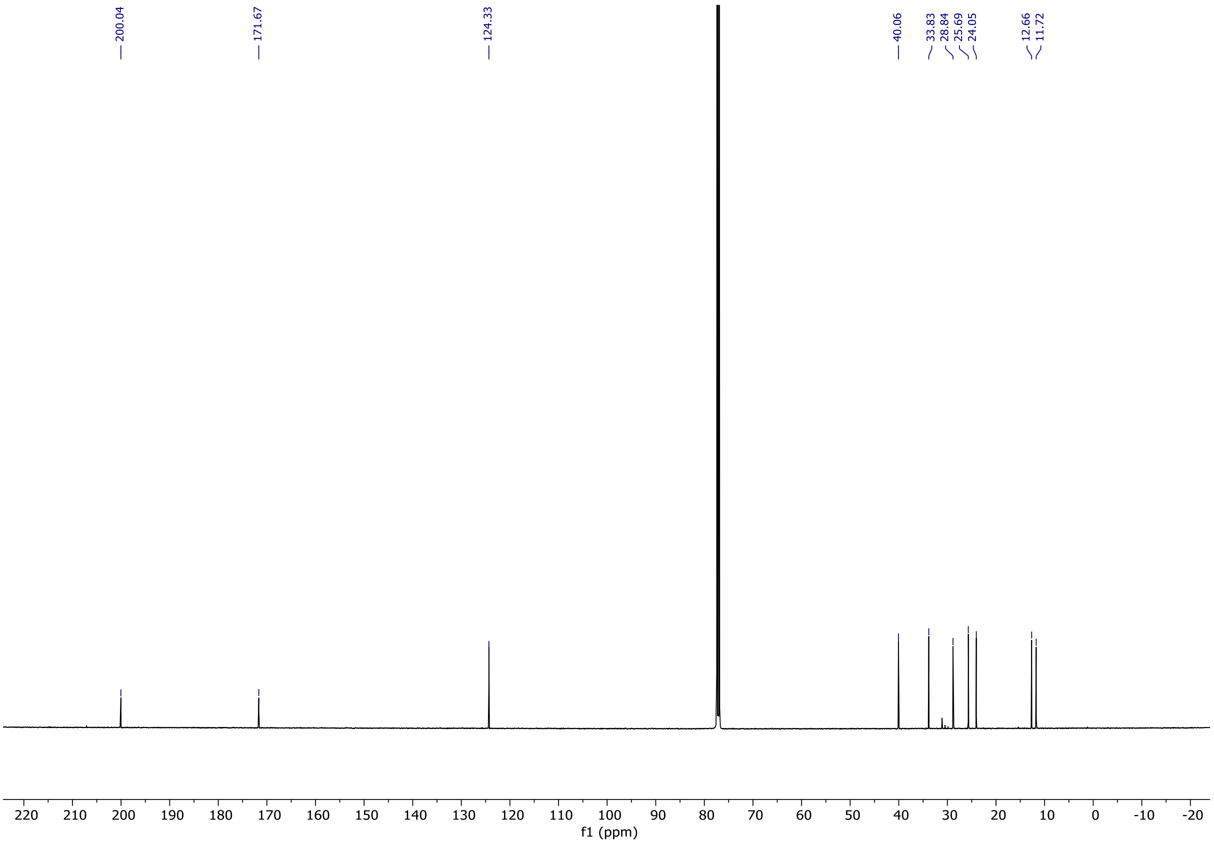

^{1}**H NMR** (500 MHz, $CDCl_3$) of **2r**

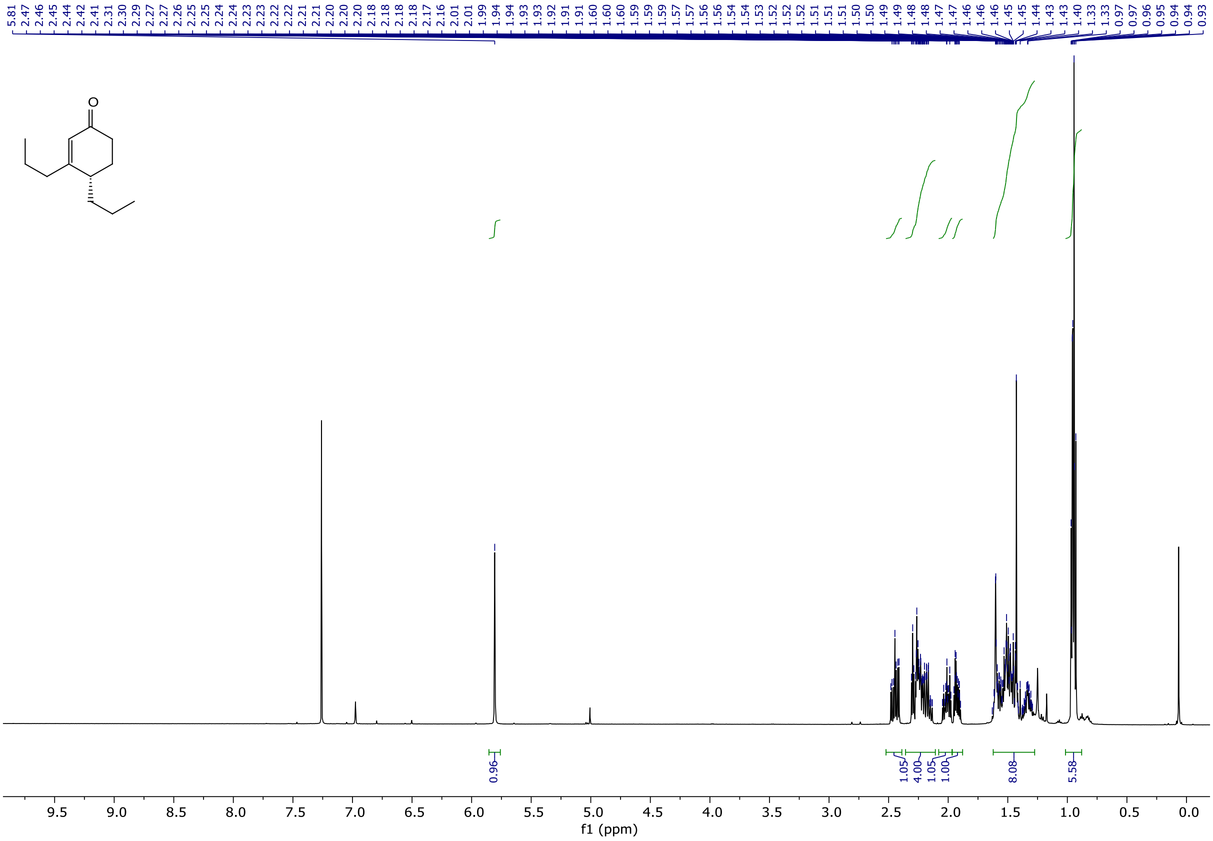

^{13}**C NMR** (126 MHz, $CDCl_3$) of **2r**

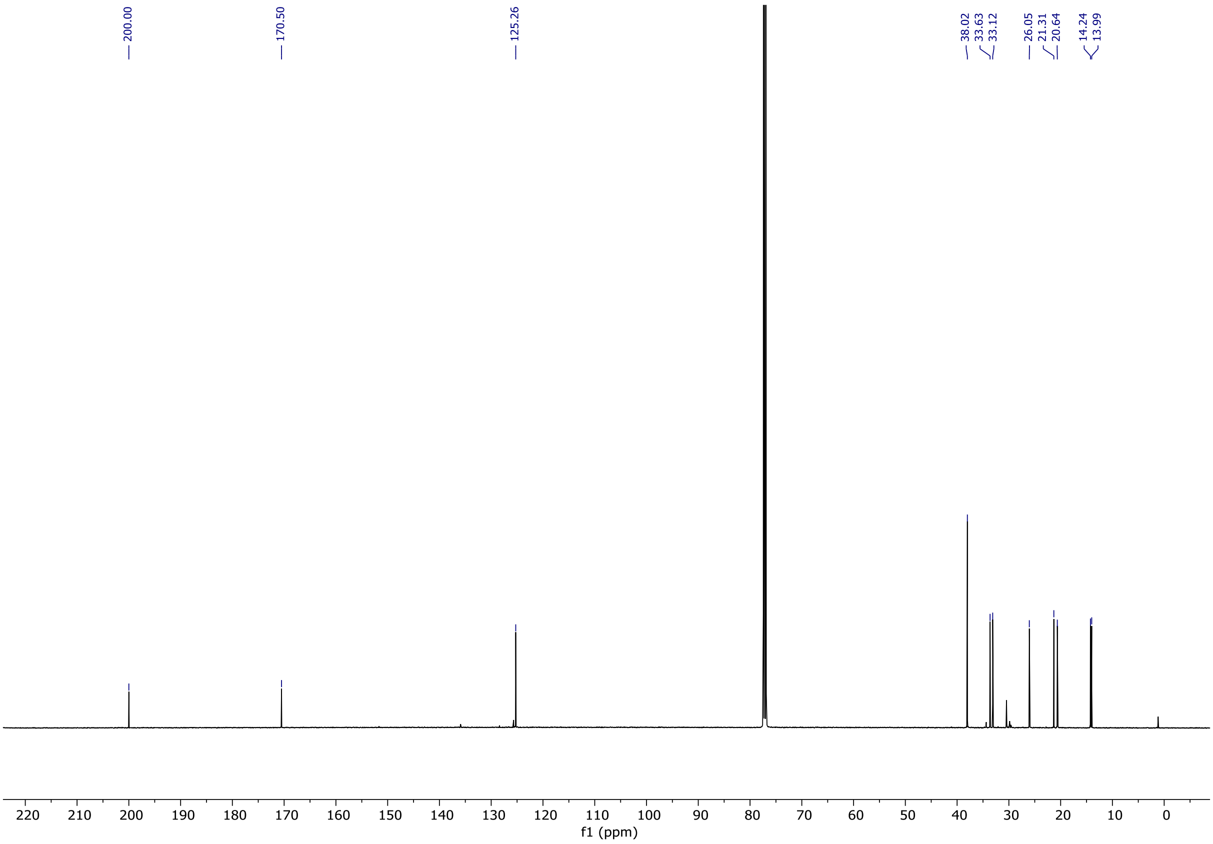

^{1}H NMR (500 MHz, CDCl$_3$) of **2s**

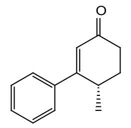

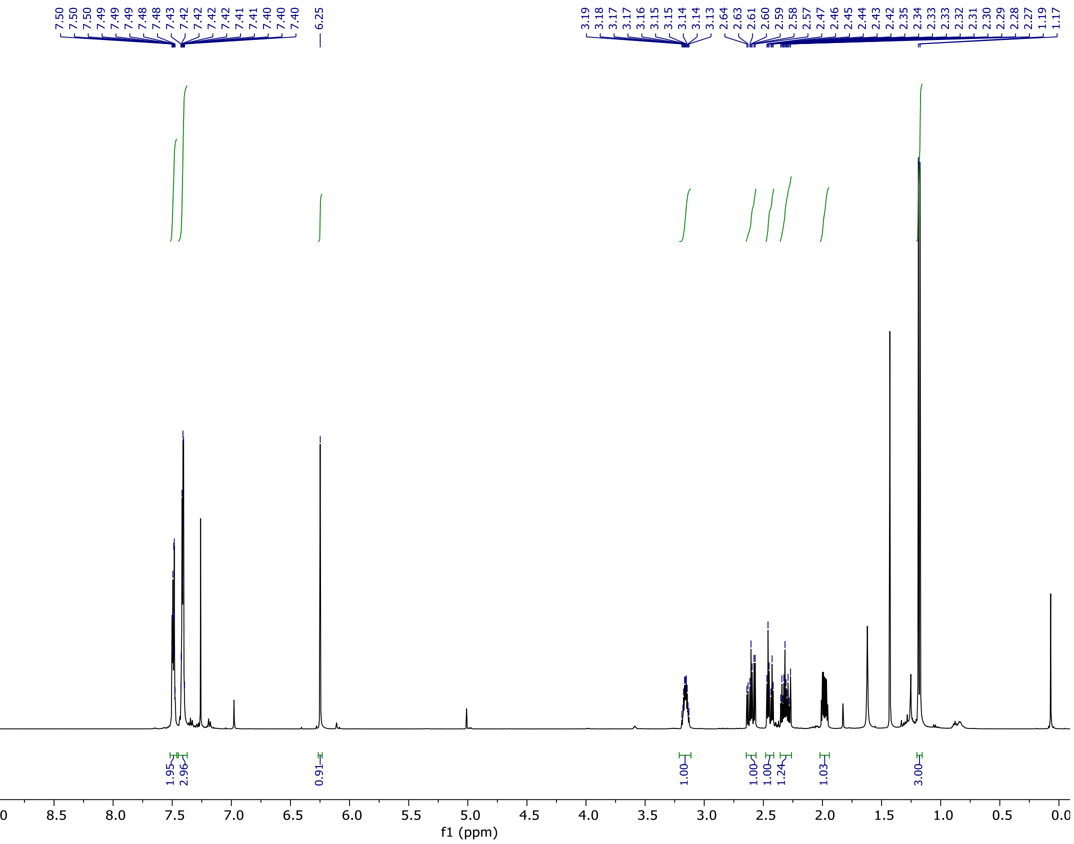

^{13}C NMR (126 MHz, CDCl$_3$) of **2s**

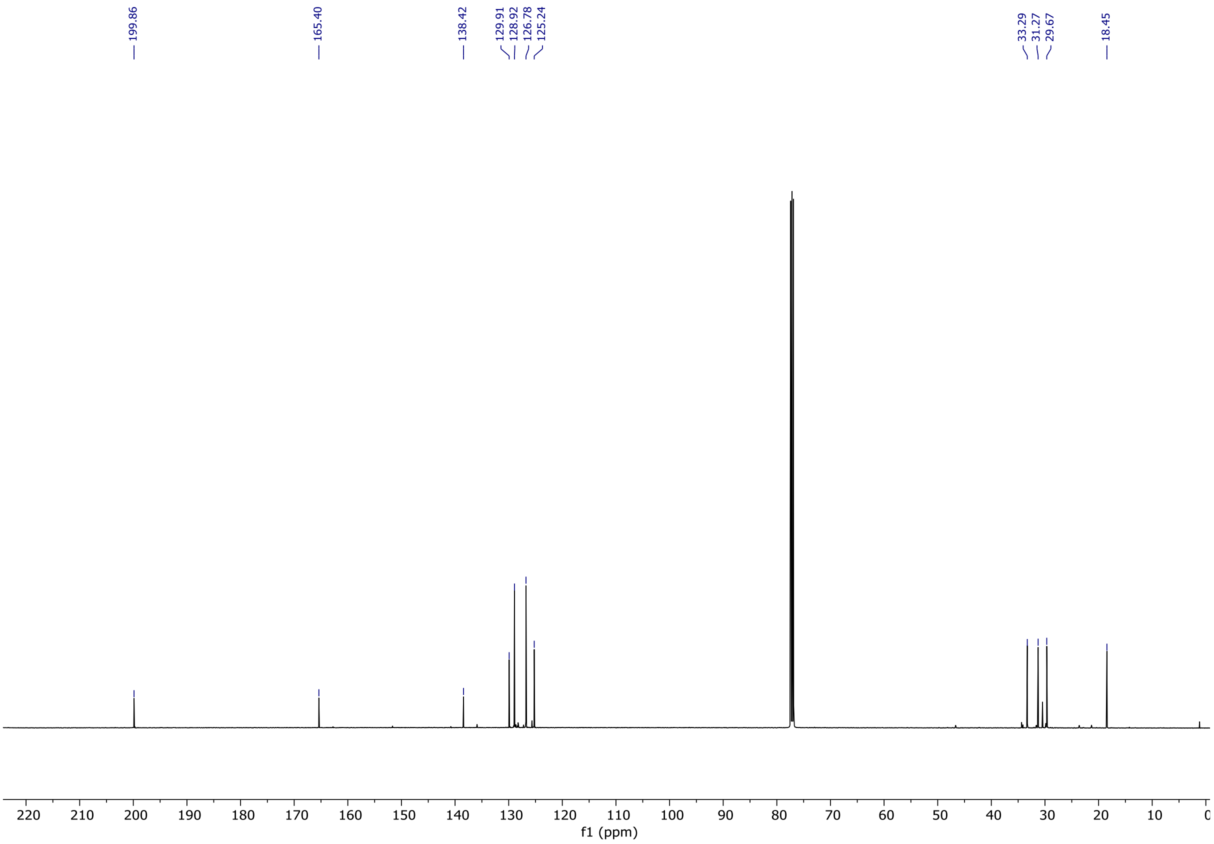

^{1}H NMR (500 MHz, $CDCl_3$) of **2t**

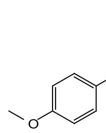

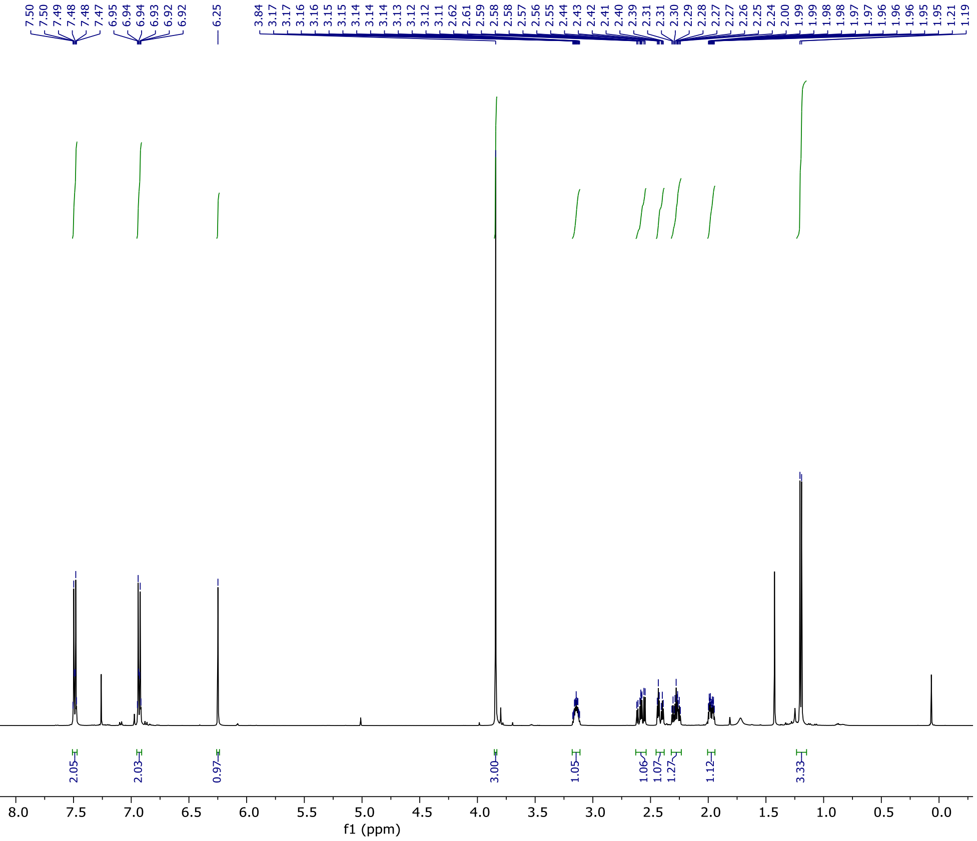

^{13}C NMR (126 MHz, $CDCl_3$) of **2t**

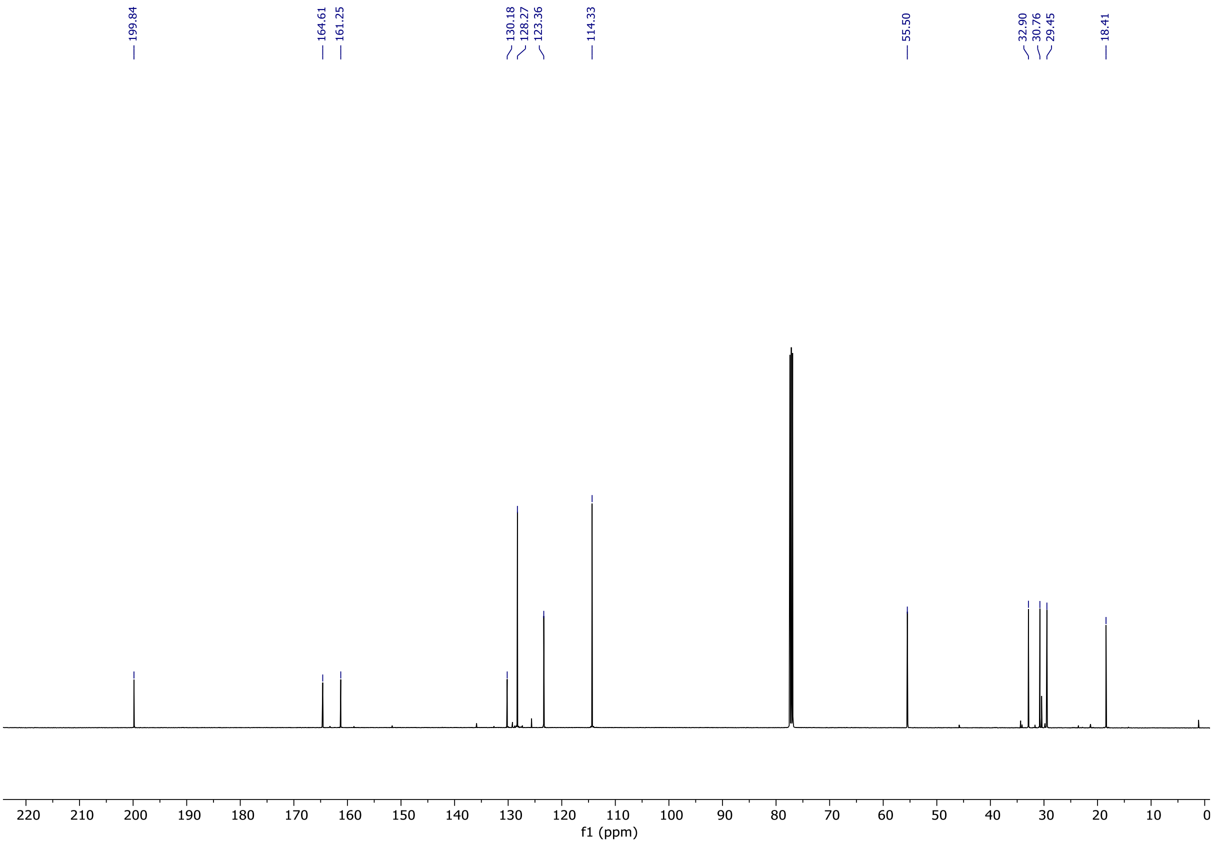

^{1}H NMR (400 MHz, Acetone-d_6) of **4a**

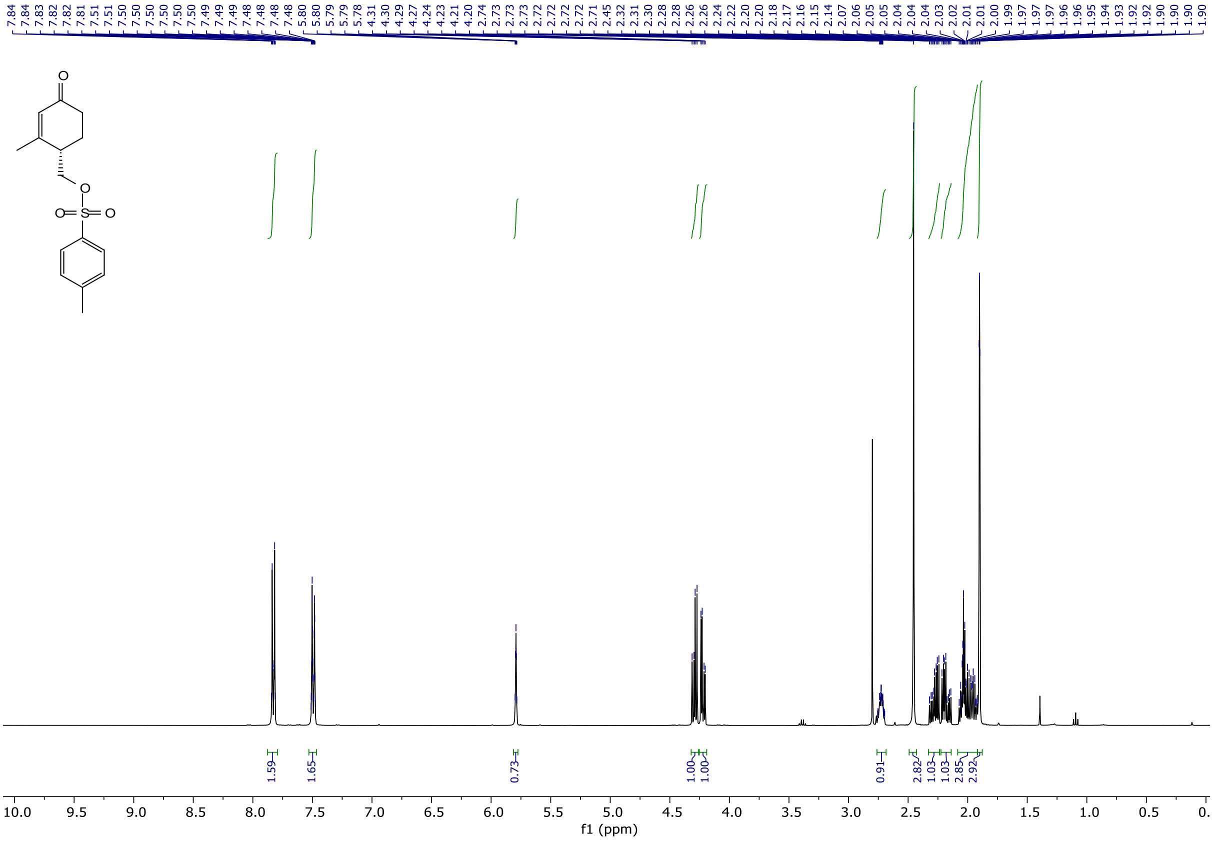

^{13}C NMR (126 MHz, Acetone-d_6) of **4a**

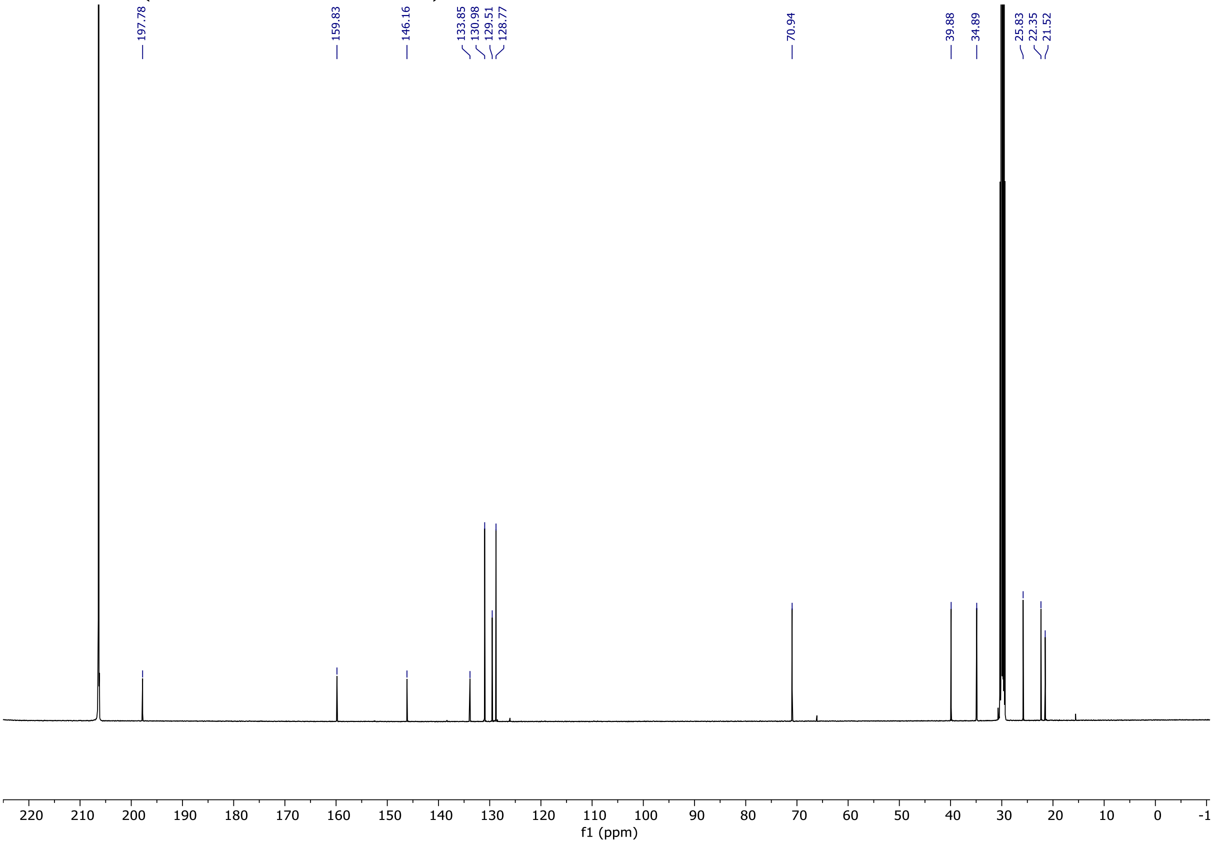

^{1}H NMR (500 MHz, $CDCl_3$) of **4b**

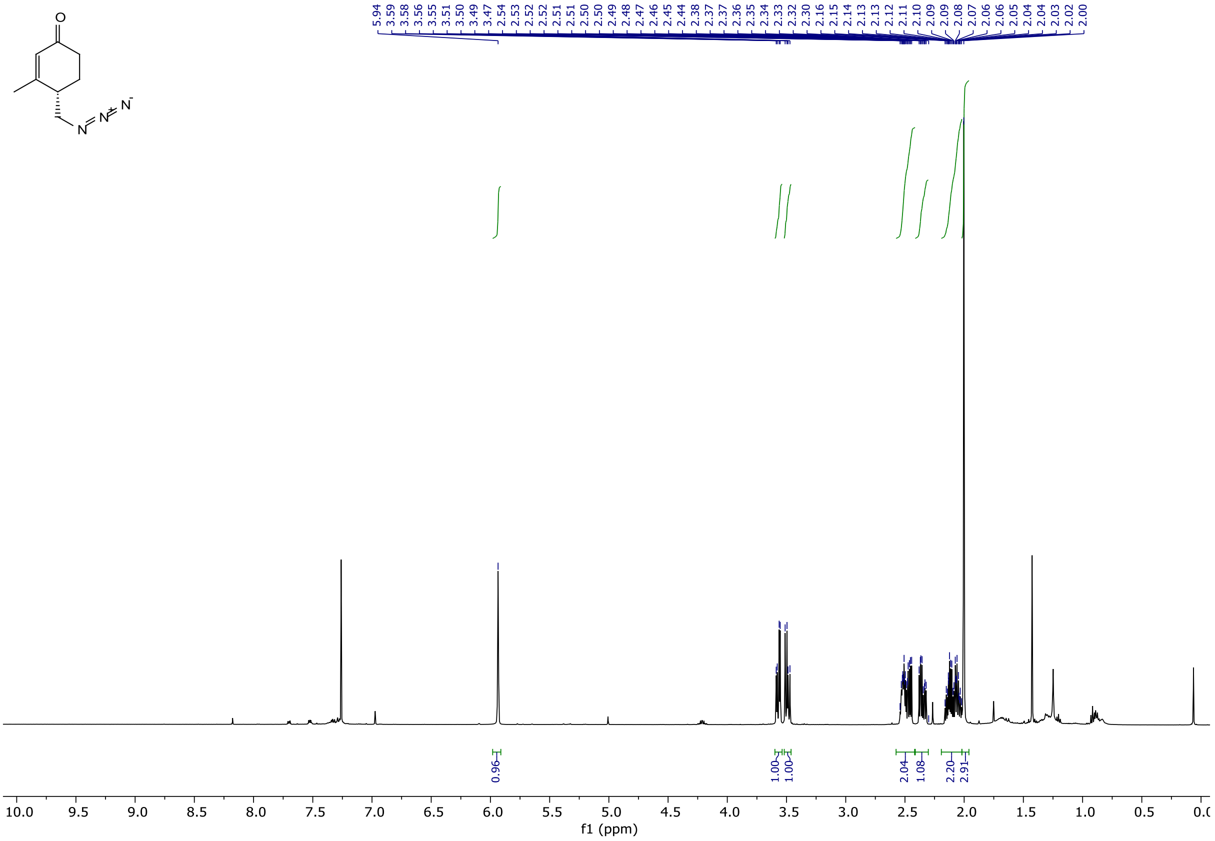

^{13}C NMR (126 MHz, $CDCl_3$) of **4b**

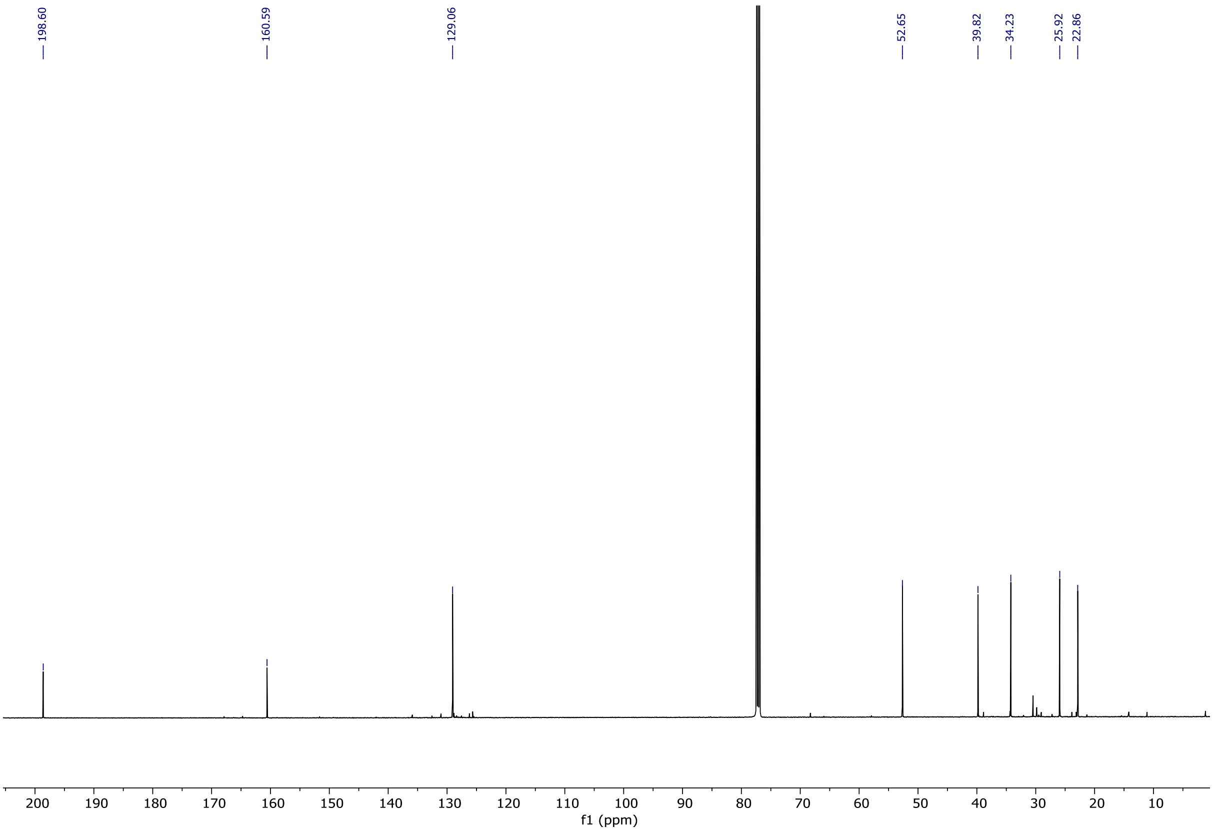

^{1}H NMR (400 MHz, CDCl$_3$) of **4c**

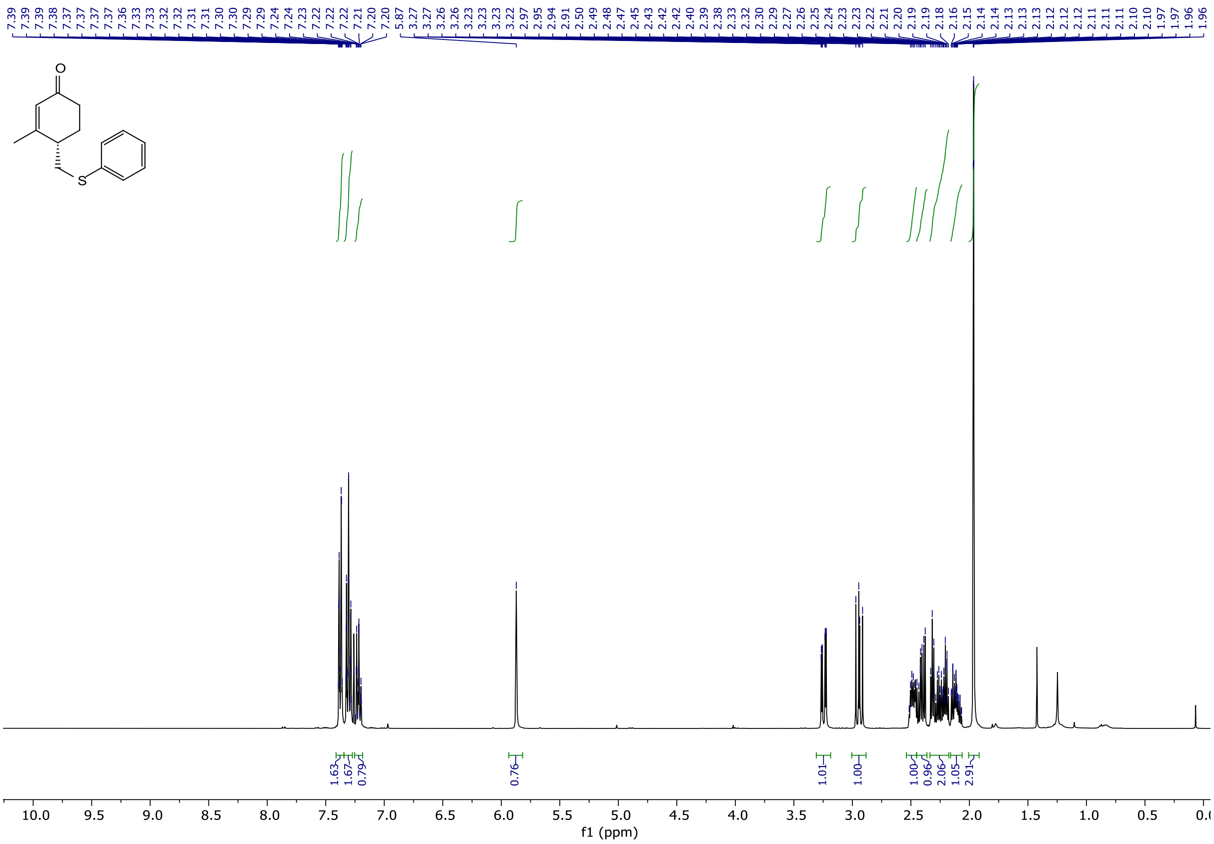

^{13}C NMR (101 MHz, CDCl$_3$) of **4c**

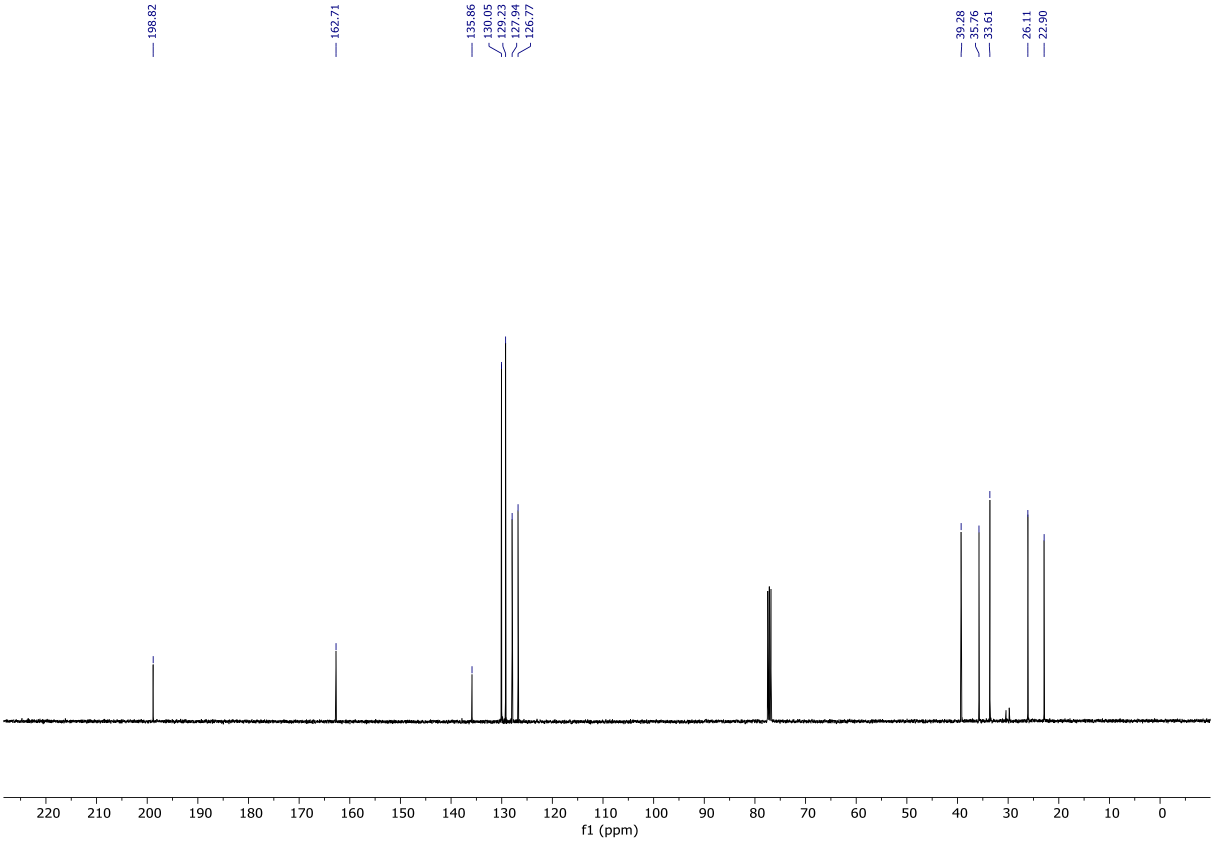

^{1}H NMR (500 MHz, Benzene-d_6) of **4d**

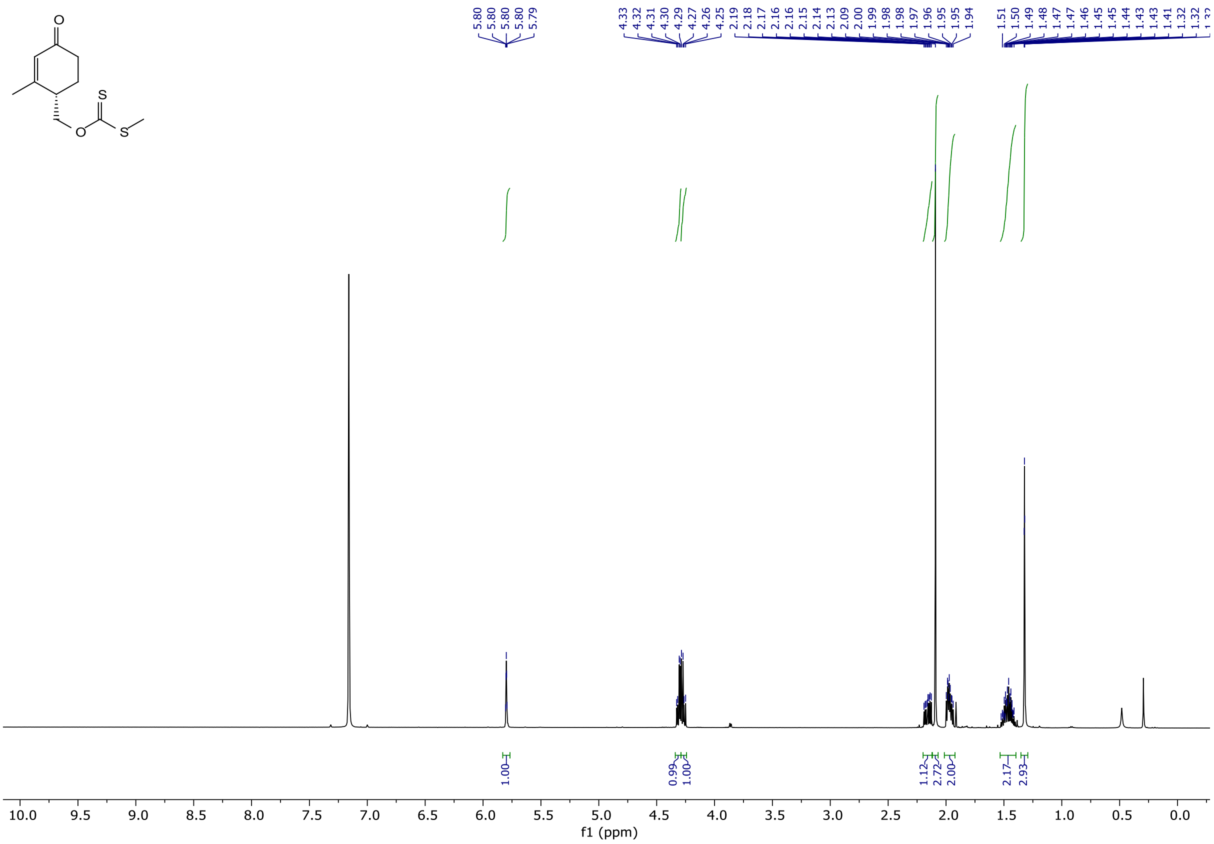

^{13}C NMR (126 MHz, Benzene-d_6) of **4d**

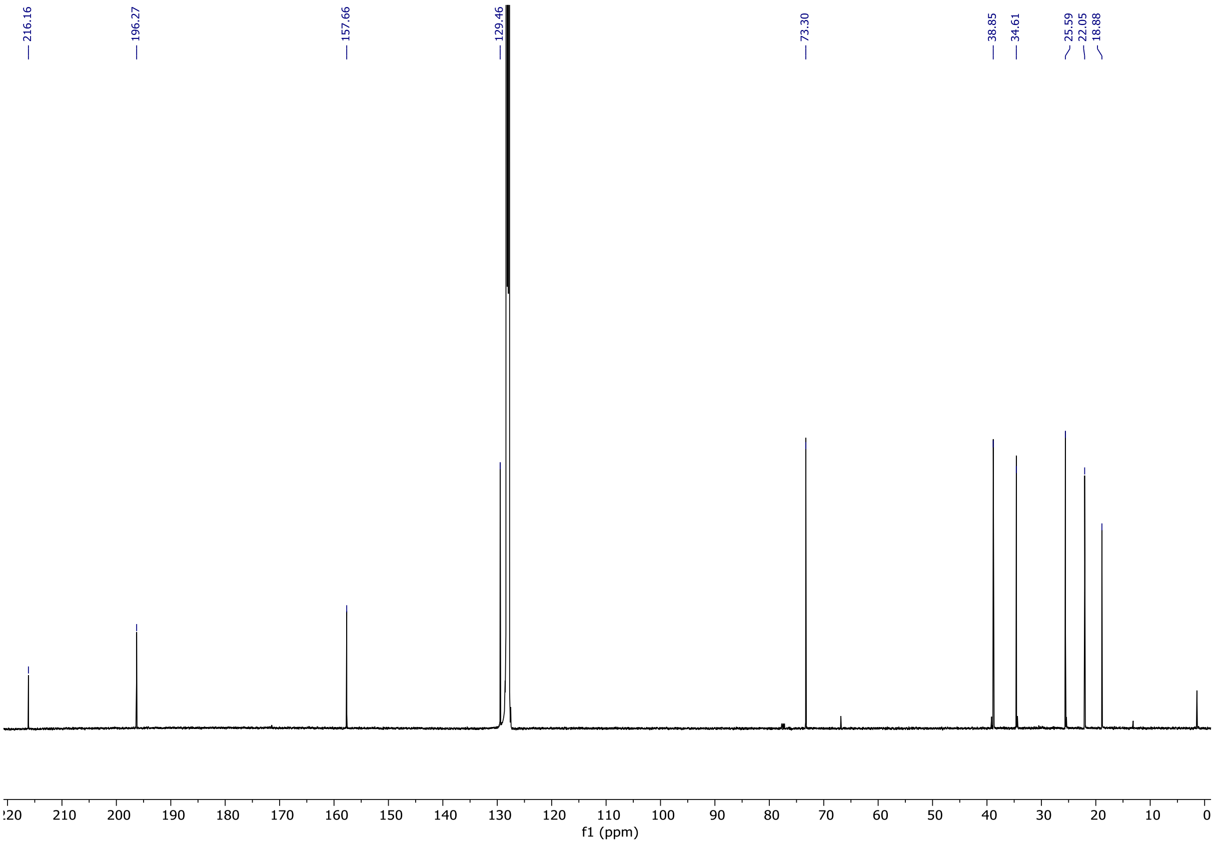

^{1}H NMR (500 MHz, Benzene-d_6) of **4e**

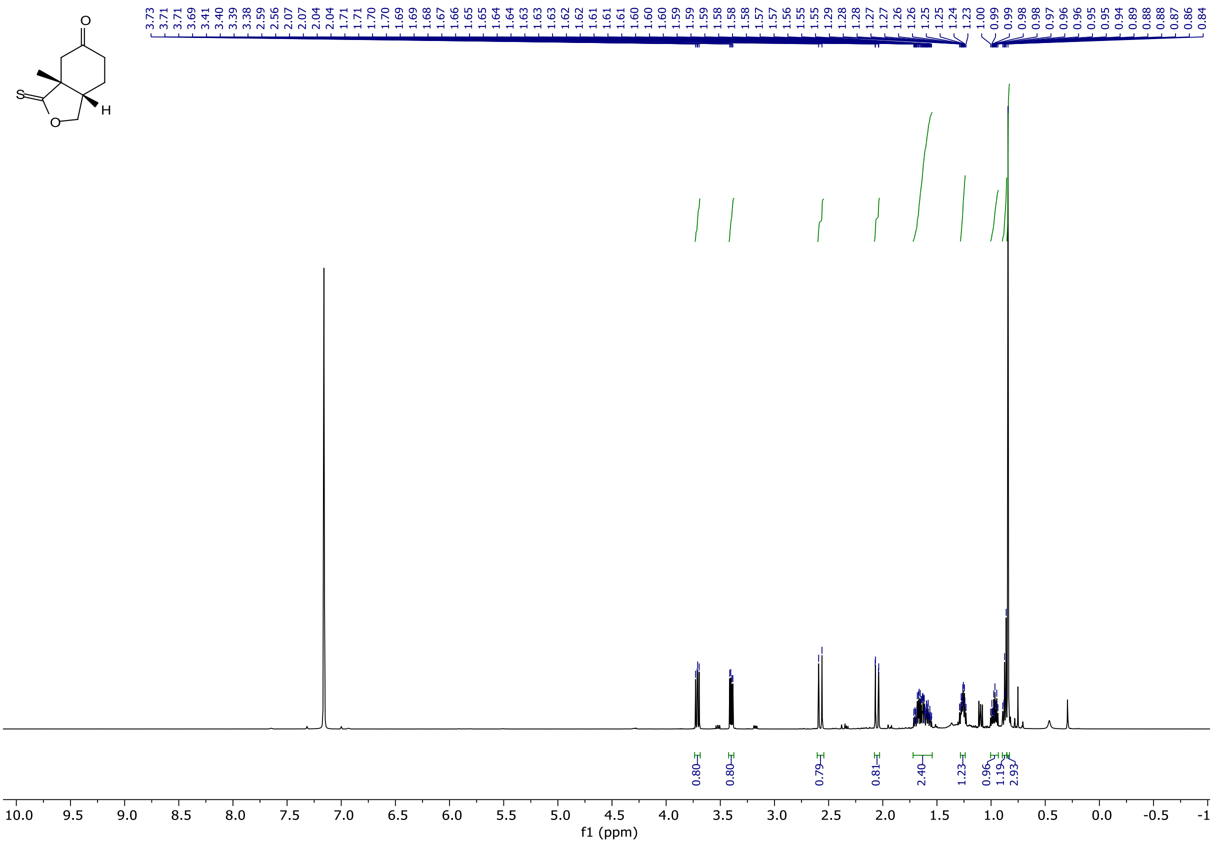

^{13}C NMR (126 MHz, Benzene-d_6) of **4e**

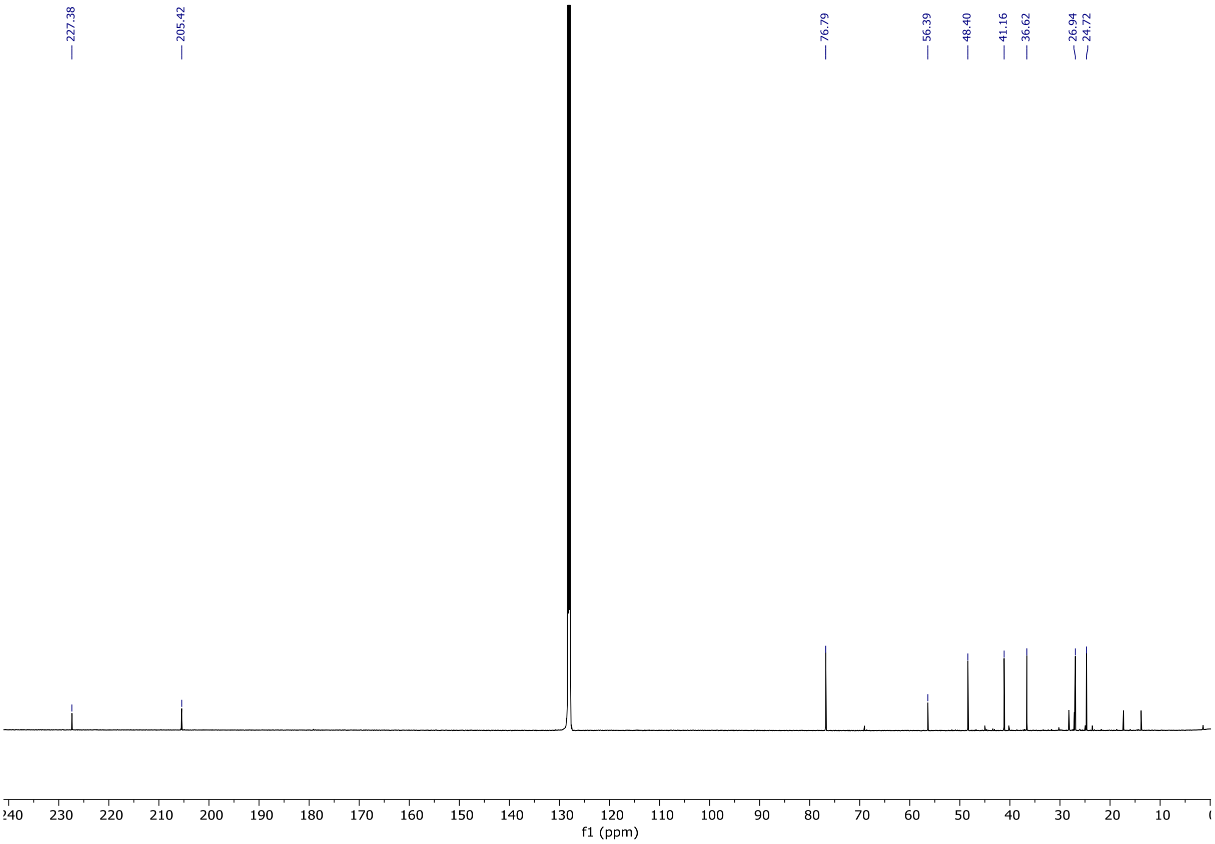

^{1}H NMR (500 MHz, Methanol-d_4) of **4f**

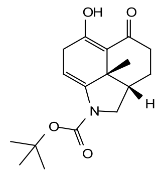

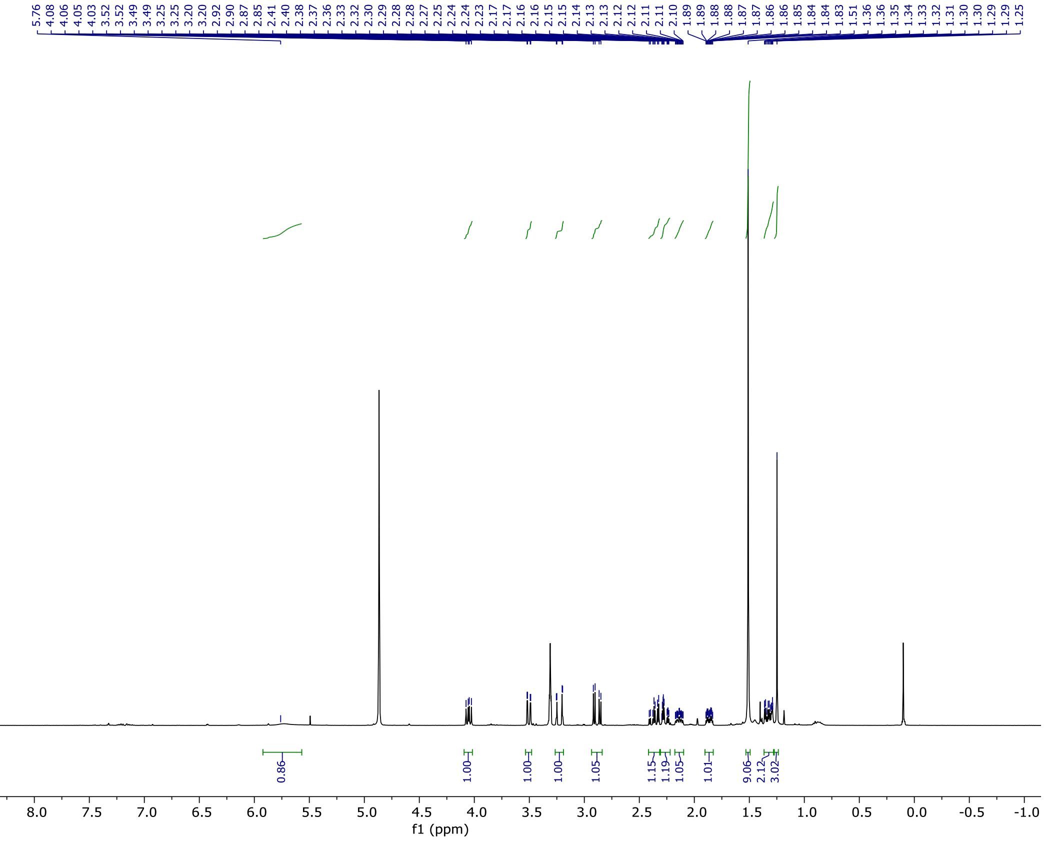

^{13}C NMR (126 MHz, Methanol-d_4) of **4f**

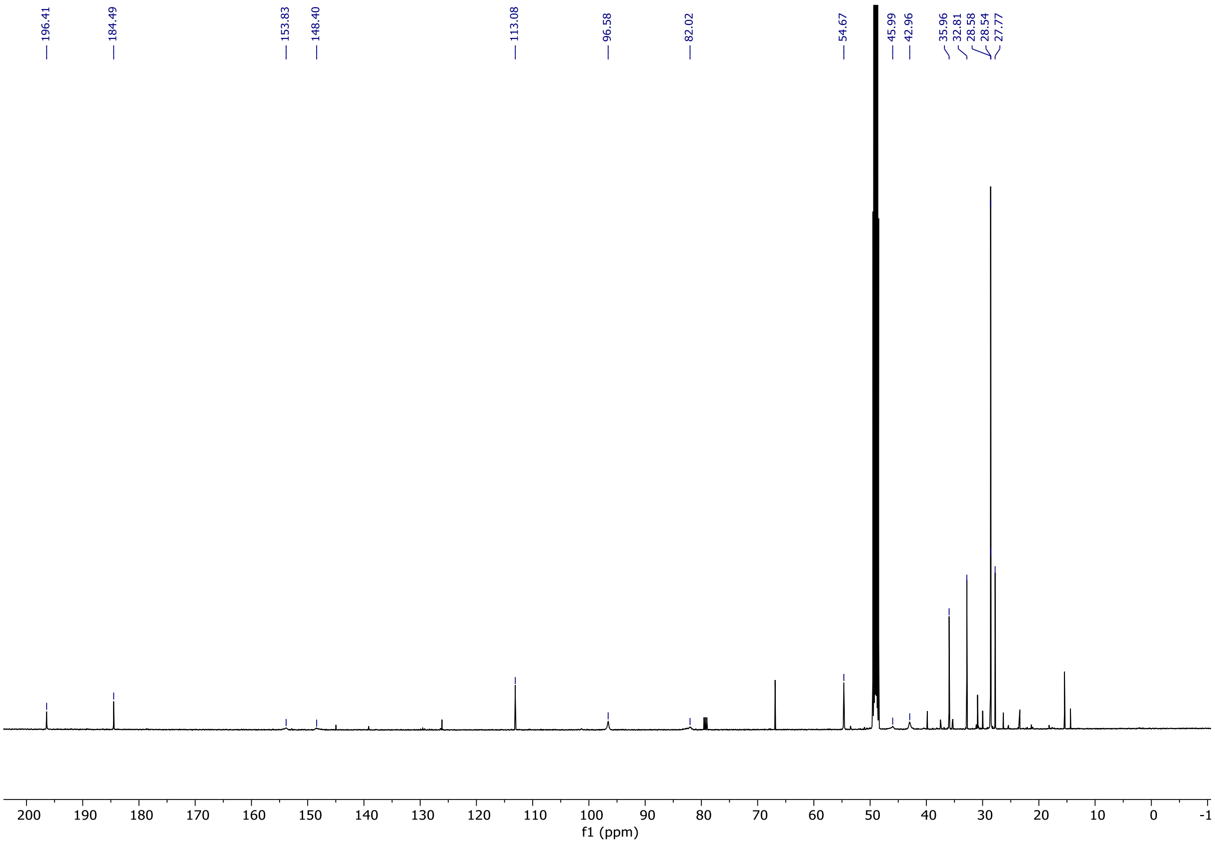

^{1}H NMR (400 MHz, $CDCl_3$) of **S17**

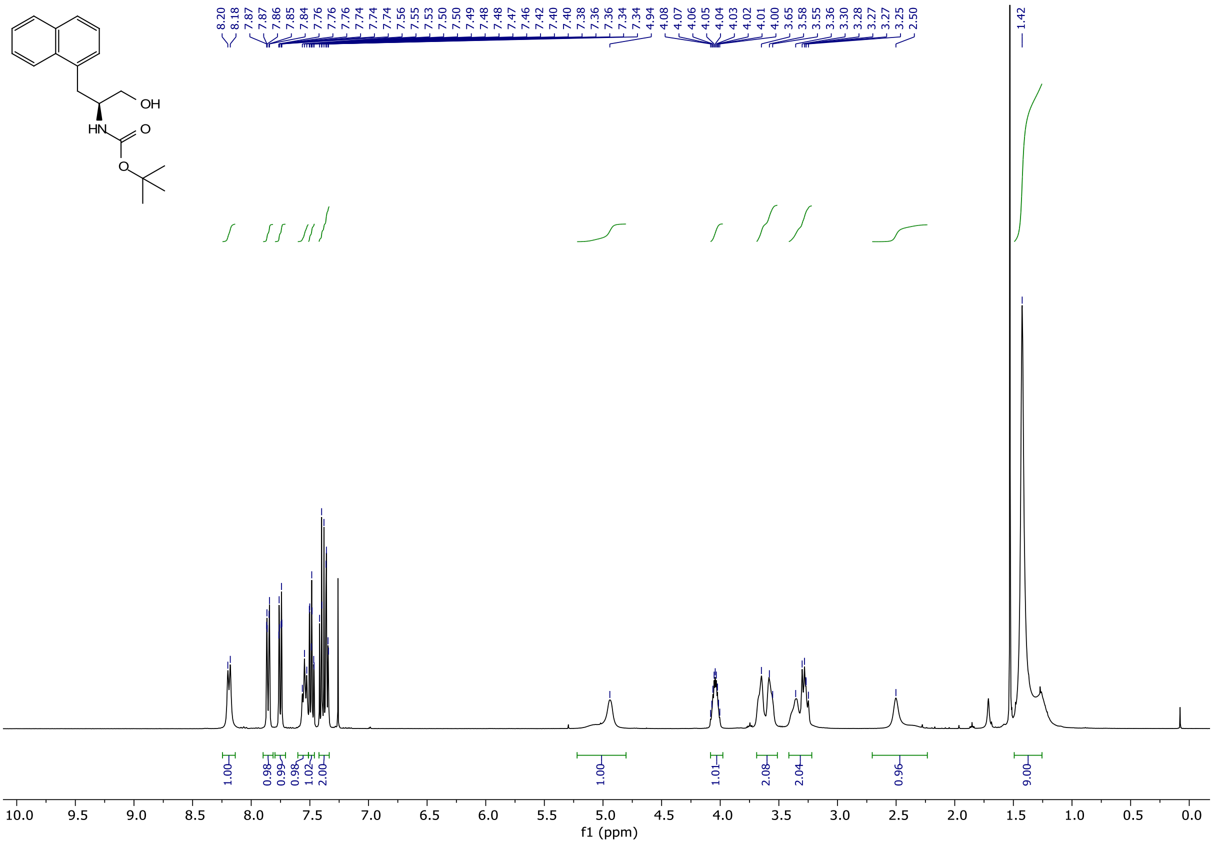

^{13}C NMR (101 MHz, $CDCl_3$) of **S17**

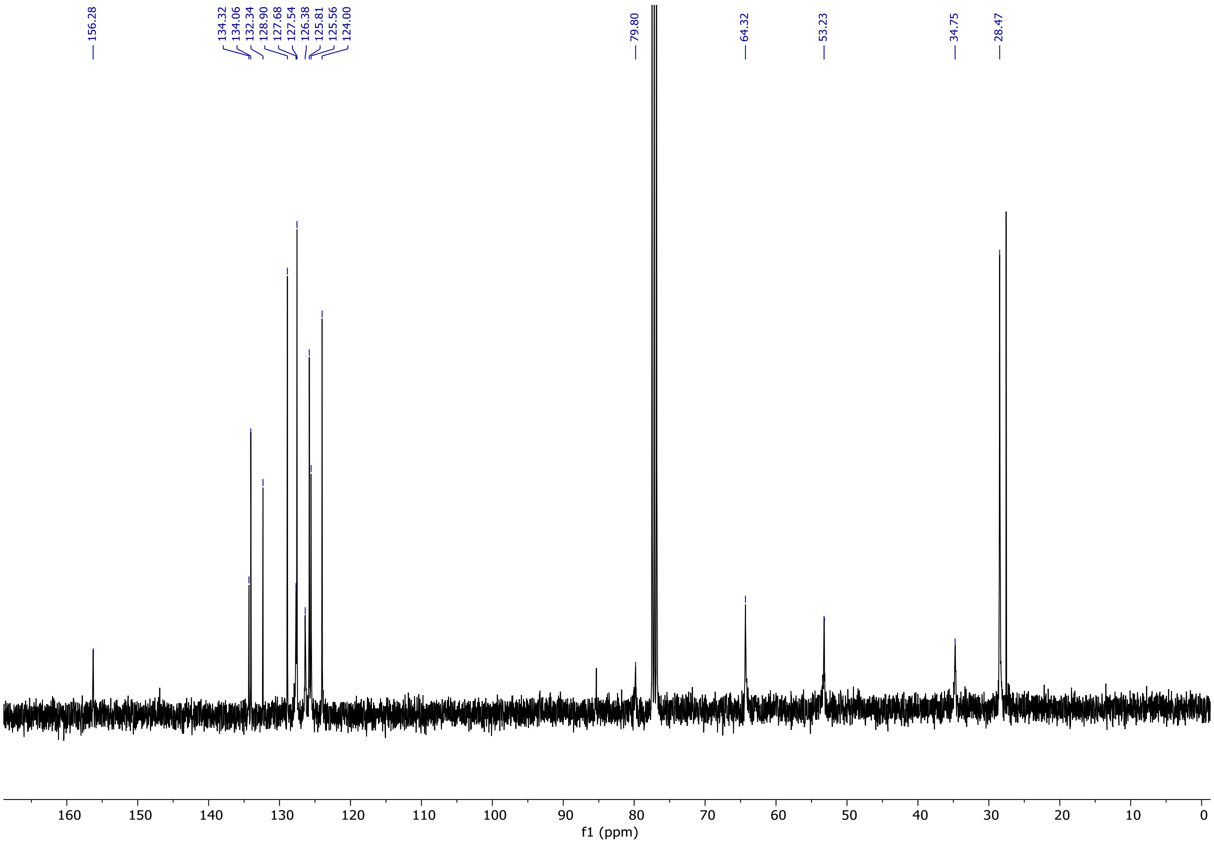

^{1}H NMR (400 MHz, CDCl$_3$) of **S19**

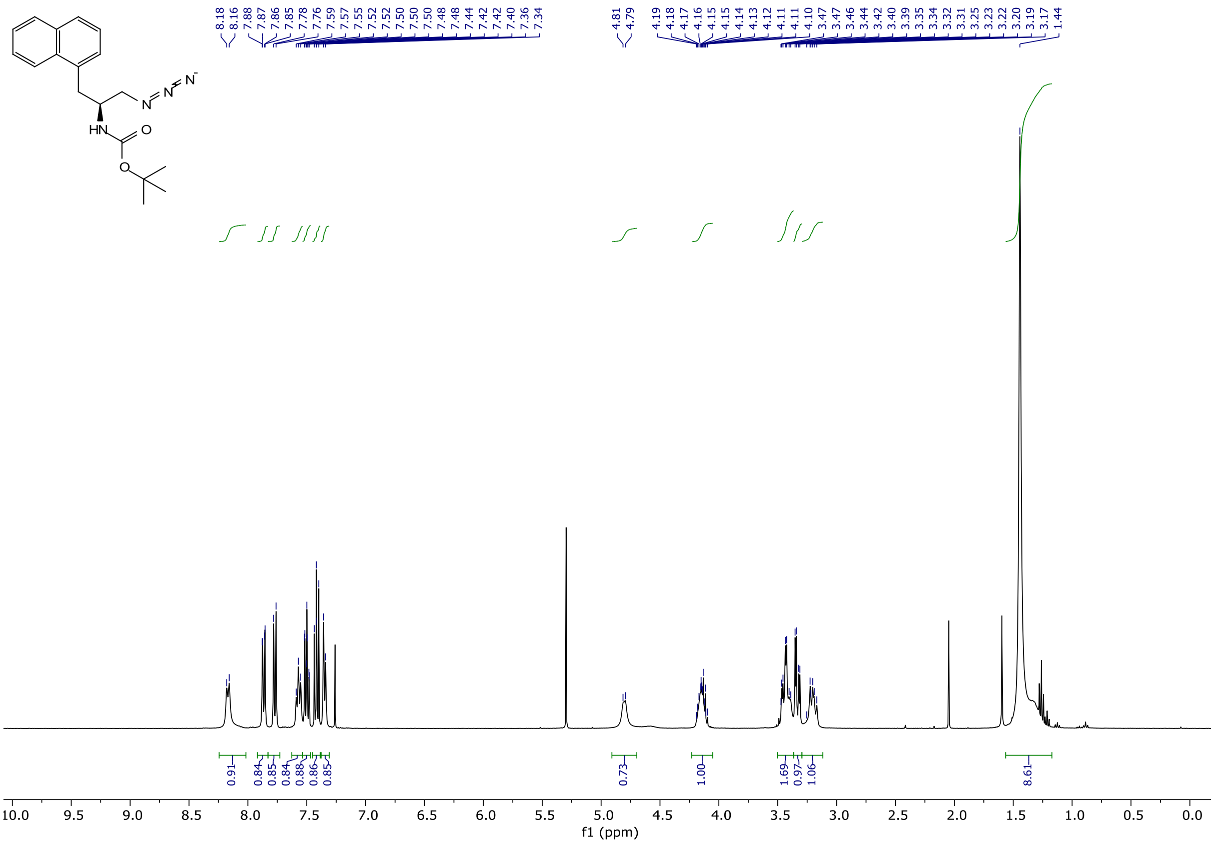

^{13}C NMR (101 MHz, CDCl$_3$) of **S19**

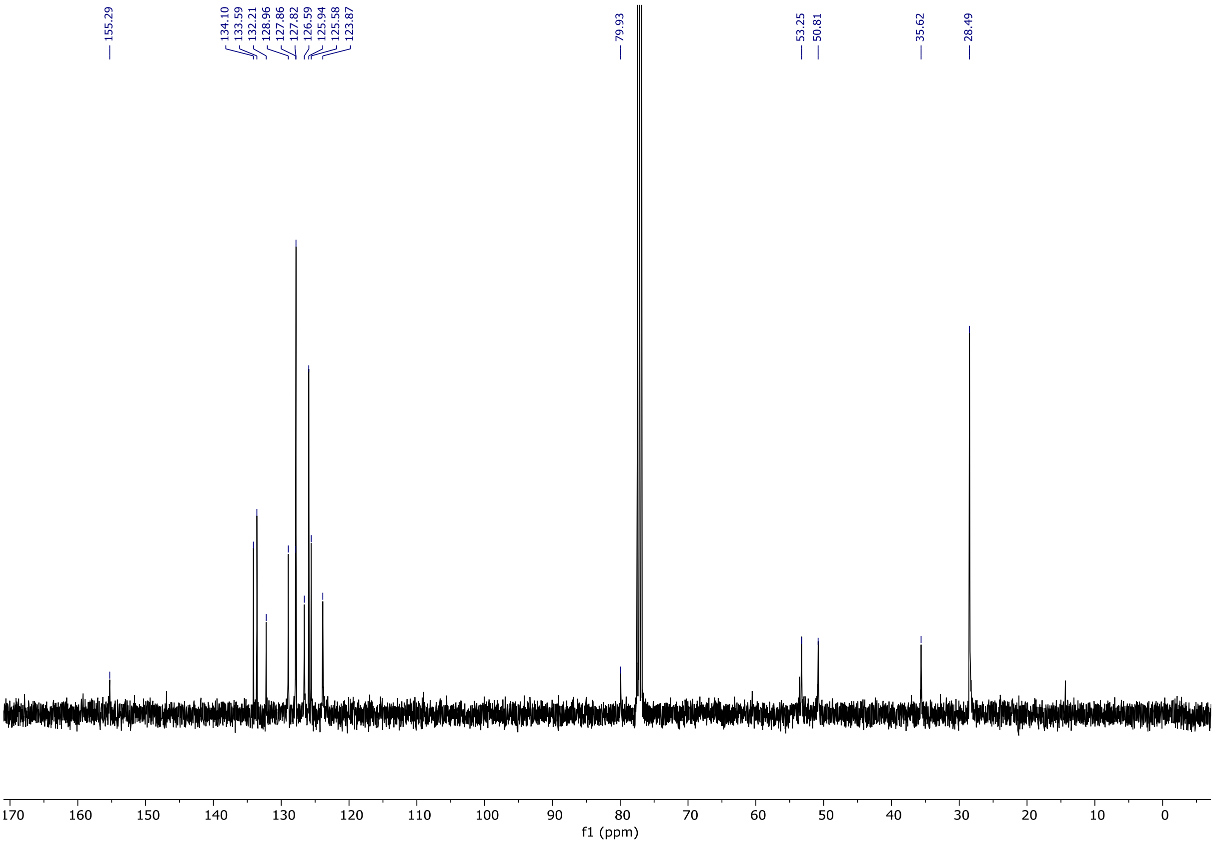

^{1}H NMR (400 MHz, Methanol-d^4) of **S20**

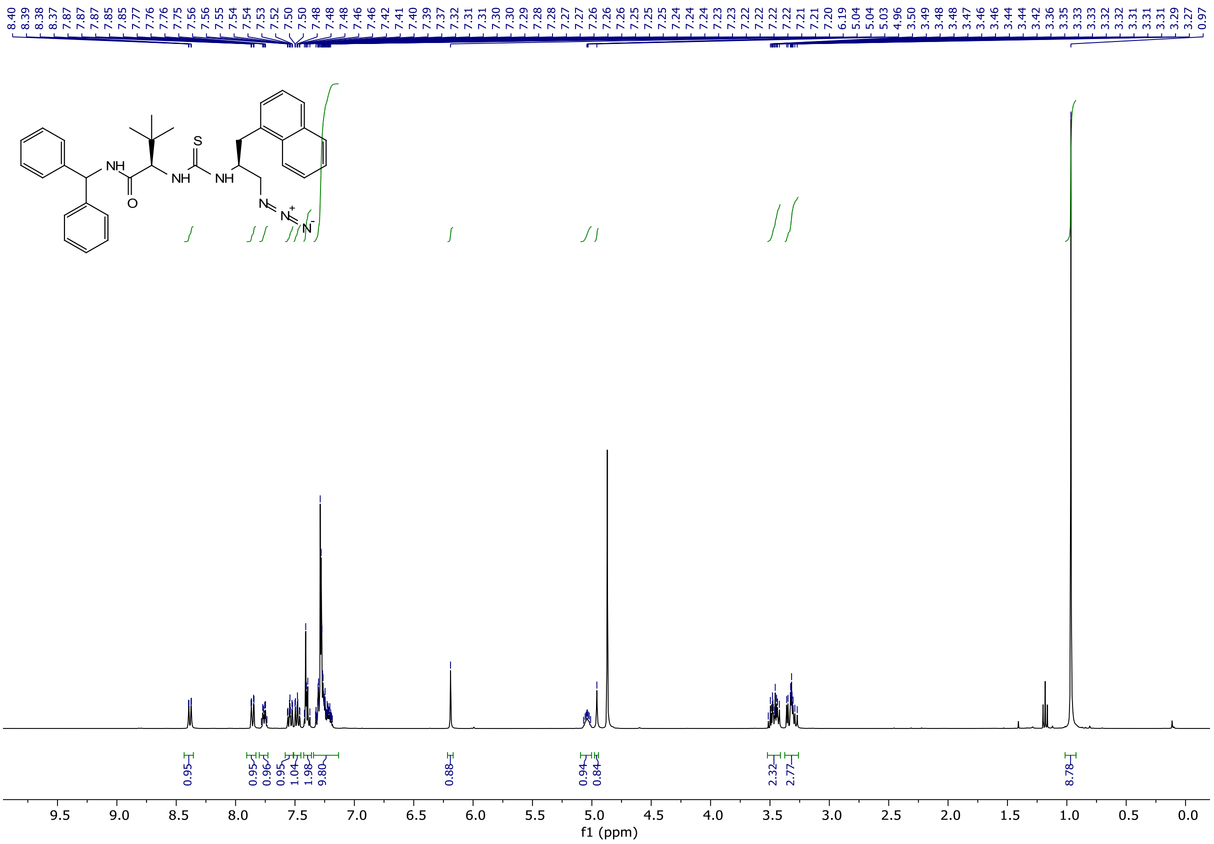

^{13}C NMR (101 MHz, Methanol-d^4) of **S20**

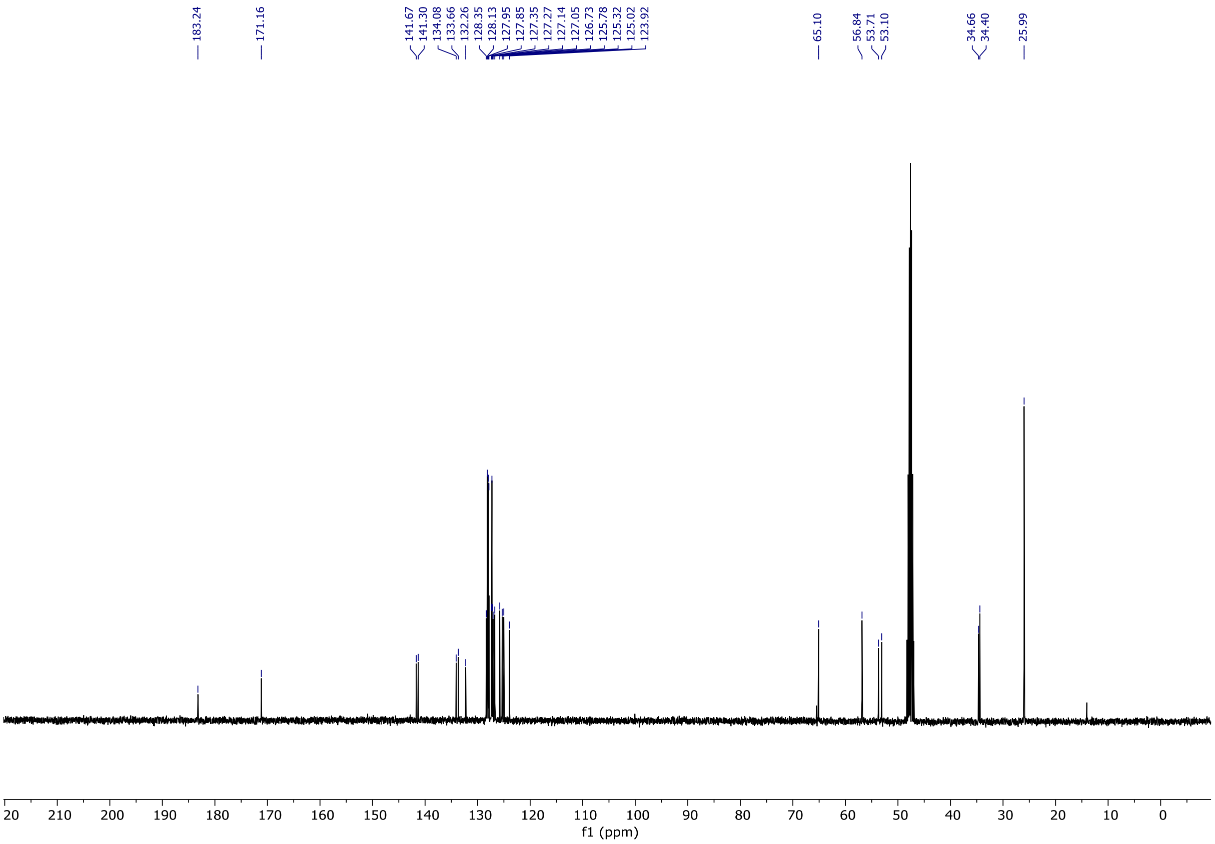

^{1}H NMR (400 MHz, $CDCl_3$) of **S21**

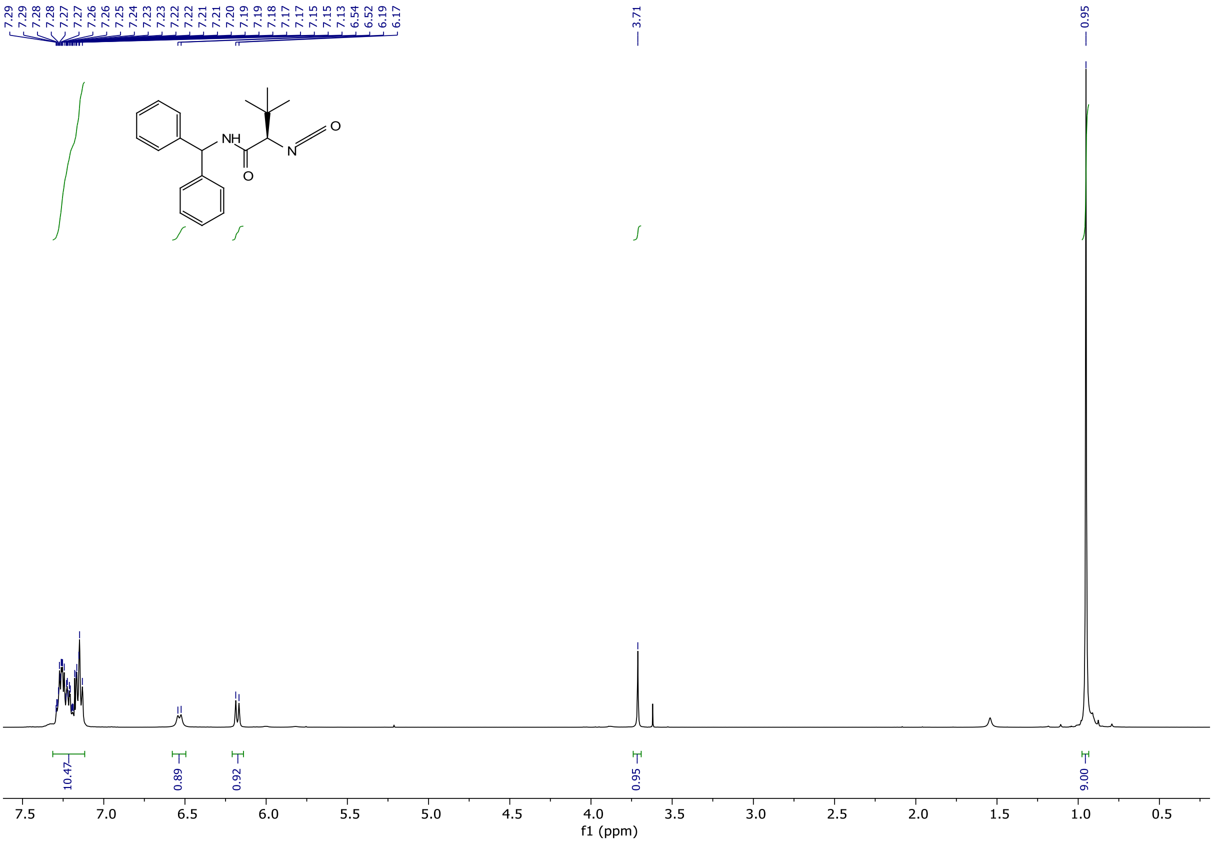

^{13}C NMR (101 MHz, $CDCl_3$) of **S21**

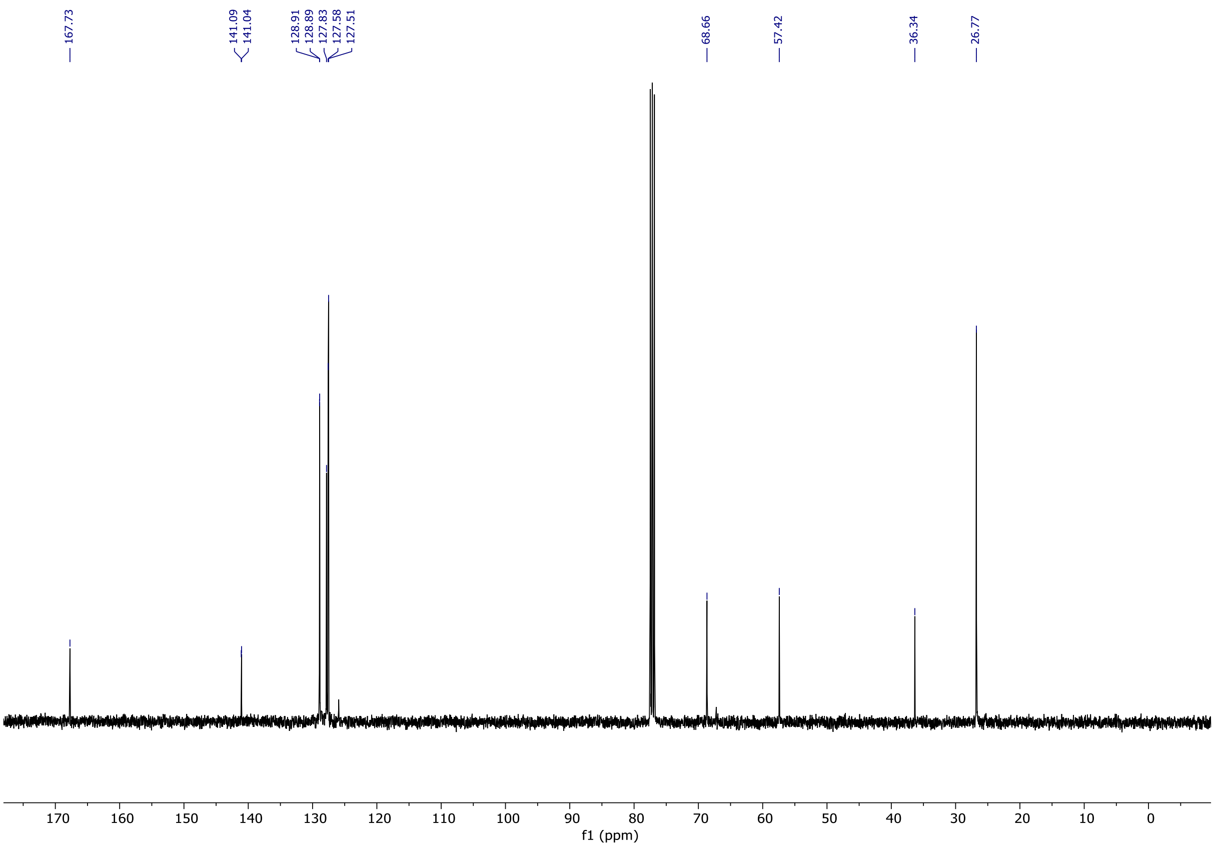

^{1}**H NMR** (500 MHz, Methanol-d_4) of **S22**

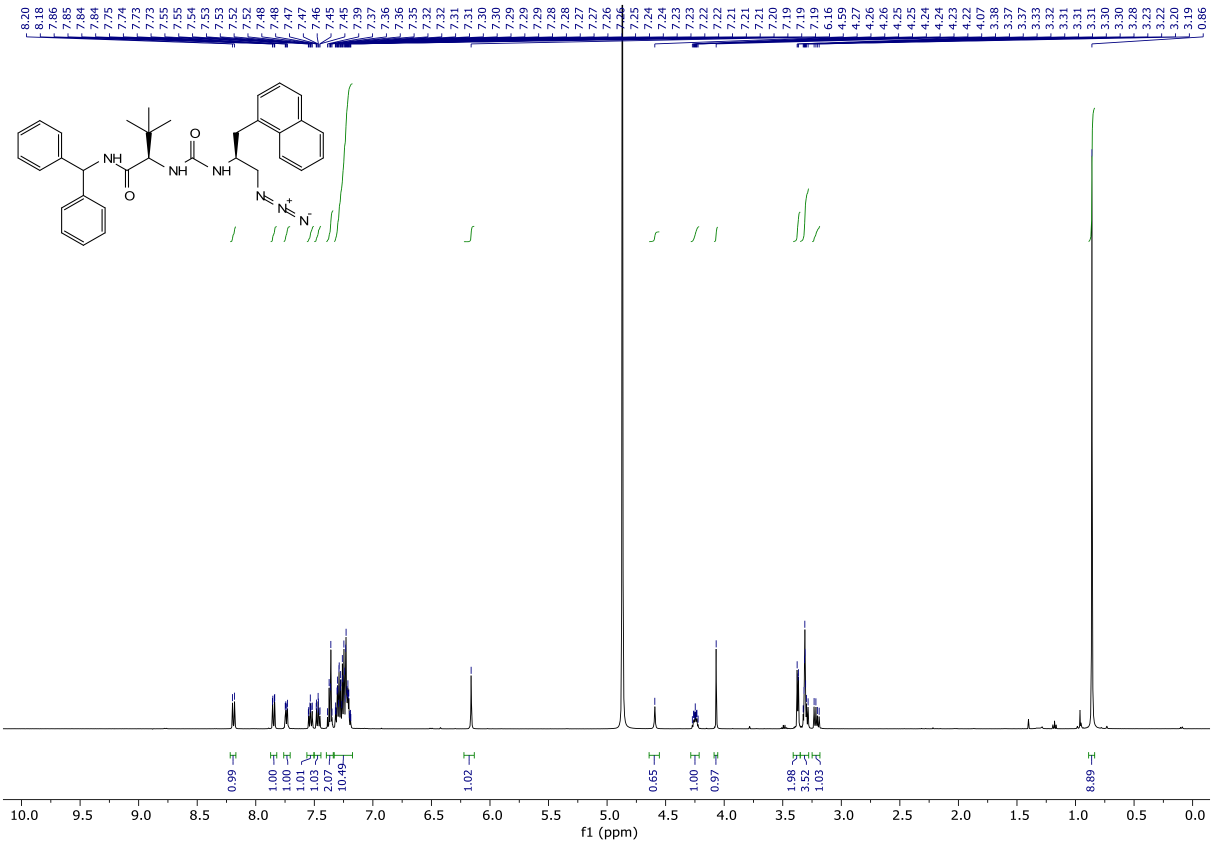

^{13}**C NMR** (126 MHz, Methanol-d_4) of **S22**

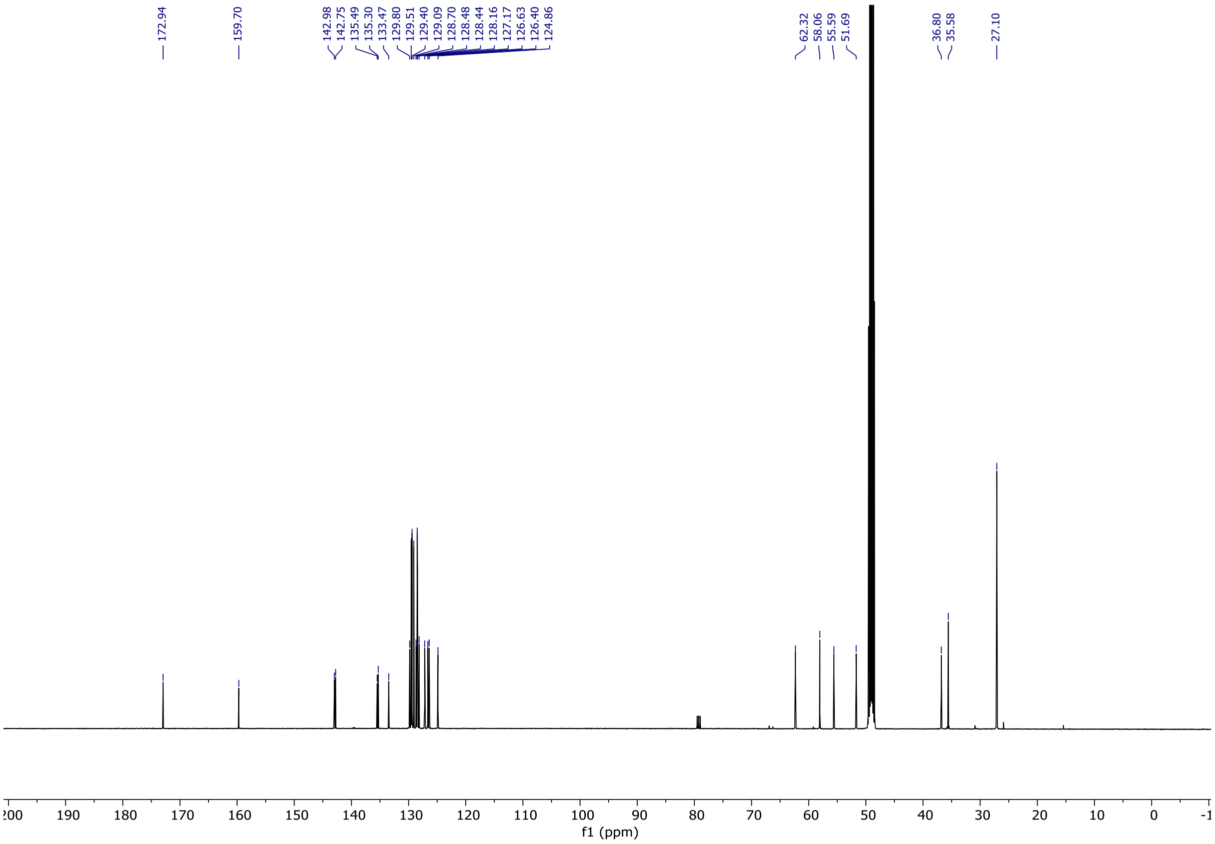

2.3 Copies of HPLC traces

(S)-4-((benzyloxy)methyl)-3-methylcyclohex-2-en-1-one (2a)

(Chiralpak AS-H, hexane/isopropanol = 70/30, 1 mL/min)

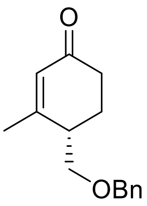

Racemic

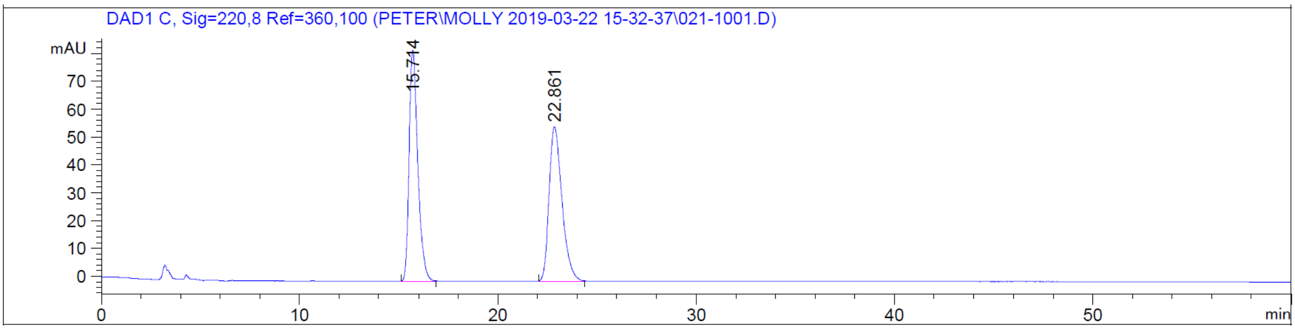

Signal 3: DAD1 C, Sig=220,8 Ref=360,100

Peak #	RetTime [min]	Type	Width [min]	Area [mAU*s]	Height [mAU]	Area %
1	15.714	BB	0.4627	2522.97363	82.99234	50.0491
2	22.861	BB	0.6948	2518.02441	55.55838	49.9509
Totals :				5040.99805	138.55072	

Enantiomerically enriched (99% ee)

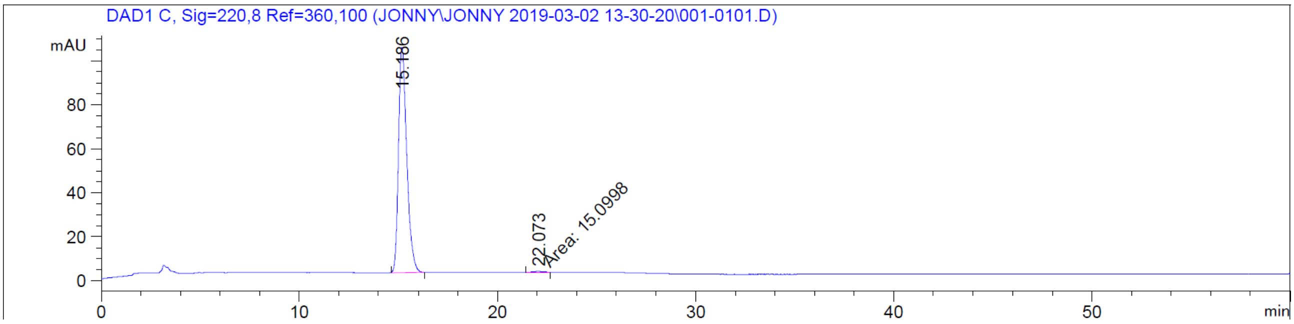

Signal 3: DAD1 C, Sig=220,8 Ref=360,100

Peak #	RetTime [min]	Type	Width [min]	Area [mAU*s]	Height [mAU]	Area %
1	15.186	BB	0.4445	2975.49756	102.54519	99.4951
2	22.073	MM	0.6248	15.09975	4.02775e-1	0.5049
Totals :				2990.59731	102.94796	

(S)-4-(methoxymethyl)-3-methylcyclohex-2-en-1-one (2b)

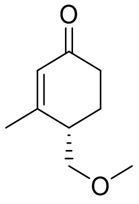

(Chiralpak AD-H, hexane/isopropanol = 95/5, 1 mL/min)

Racemic

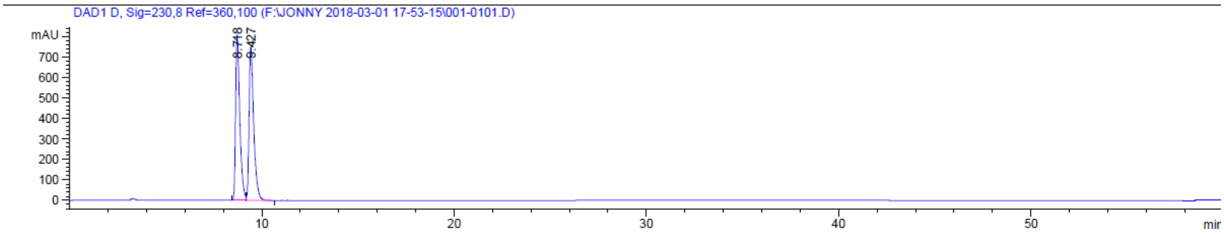

Signal 4: DAD1 D, Sig=230,8 Ref=360,100

Peak #	RetTime [min]	Type	Width [min]	Area [mAU*s]	Height [mAU]	Area %
1	8.718	BV	0.2308	1.23747e4	807.54541	49.6030
2	9.427	VB	0.2537	1.25727e4	749.58081	50.3970

Totals : 2.49474e4 1557.12622

Enantiomerically enriched (96% ee)

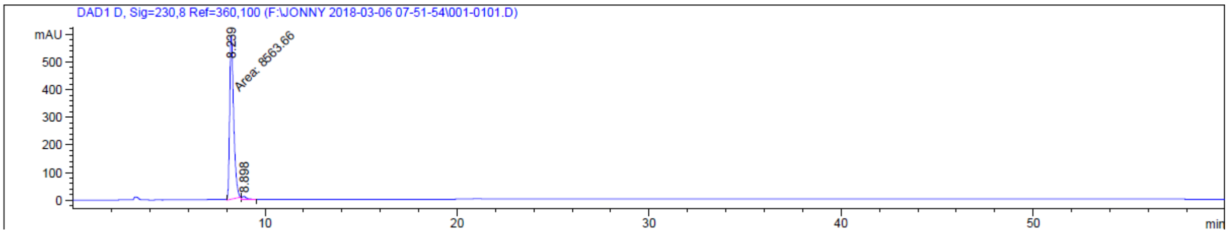

Signal 4: DAD1 D, Sig=230,8 Ref=360,100

Peak #	RetTime [min]	Type	Width [min]	Area [mAU*s]	Height [mAU]	Area %
1	8.239	MM	0.2402	8563.65918	594.28741	97.9566
2	8.898	VB	0.2418	178.63654	10.64399	2.0434

Totals : 8742.29572 604.93140

(S)-4-((allyloxy)methyl)-3-methylcyclohex-2-en-1-one (2c)

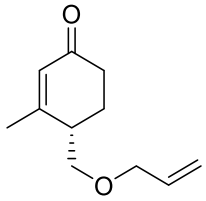

(Chiralpak AS-H, hexane/isopropanol = 70/30, 1 mL/min)

Racemic

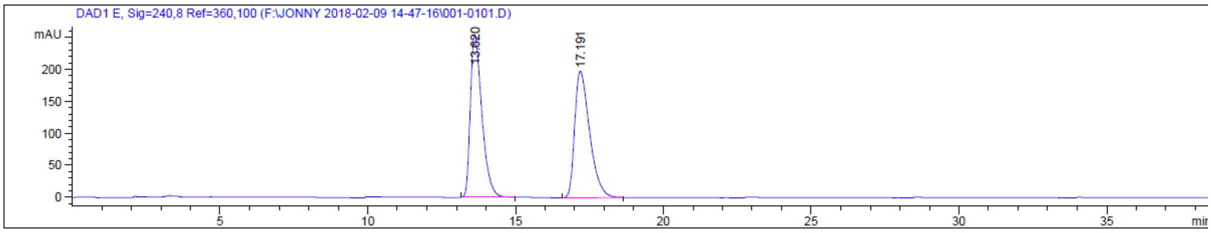

Signal 5: DAD1 E, Sig=240,8 Ref=360,100

Peak #	RetTime [min]	Type	Width [min]	Area [mAU*s]	Height [mAU]	Area %
1	13.620	BB	0.4165	6893.32178	254.11879	49.8071
2	17.191	BB	0.5419	6946.73047	197.72774	50.1929
Totals :				1.38401e4	451.84653	

Enanantiomerically enriched (98% ee)

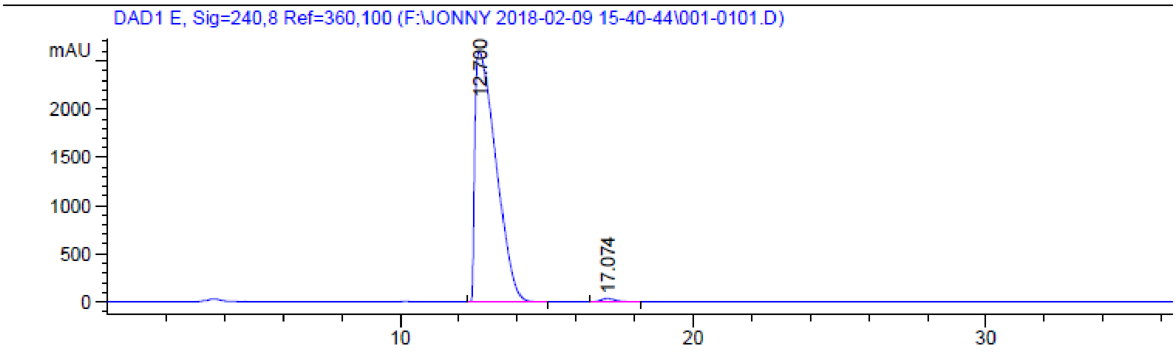

Signal 5: DAD1 E, Sig=240,8 Ref=360,100

Peak #	RetTime [min]	Type	Width [min]	Area [mAU*s]	Height [mAU]	Area %
1	12.700	BB	0.7935	1.30495e5	2600.03125	99.1232
2	17.074	BB	0.5053	1154.28345	34.94859	0.8768
Totals :				1.31649e5	2634.97984	

(S)-3-methyl-4-(((3-methylbut-2-en-1-yl)oxy)methyl)cyclohex-2-en-1-one (2d)

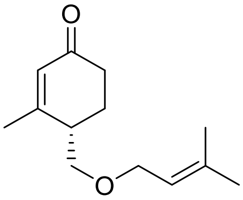

(Chiralpak AS-H, hexane/isopropanol = 95/5, 1 mL/min)

Racemic

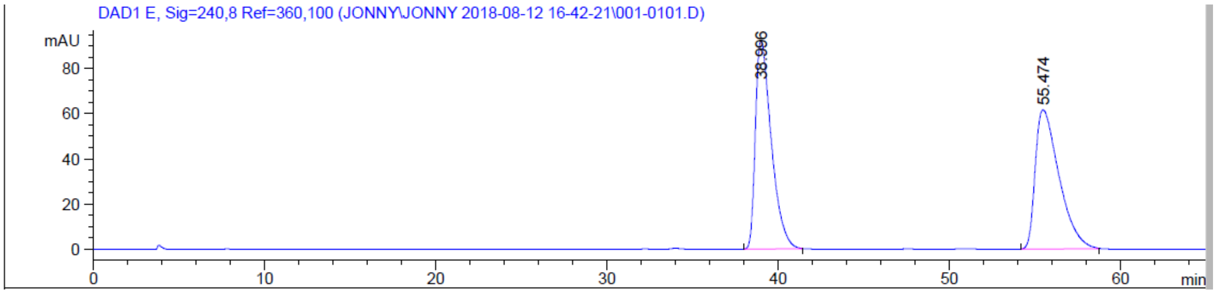

Signal 5: DAD1 E, Sig=240,8 Ref=360,100

Peak #	RetTime [min]	Type	Width [min]	Area [mAU*s]	Height [mAU]	Area %
1	38.996	BB	0.9832	5970.24365	92.23606	50.0104
2	55.474	BB	1.4405	5967.75879	61.69810	49.9896

Totals : 1.19380e4 153.93417

Enantiomerically enriched (99% ee)

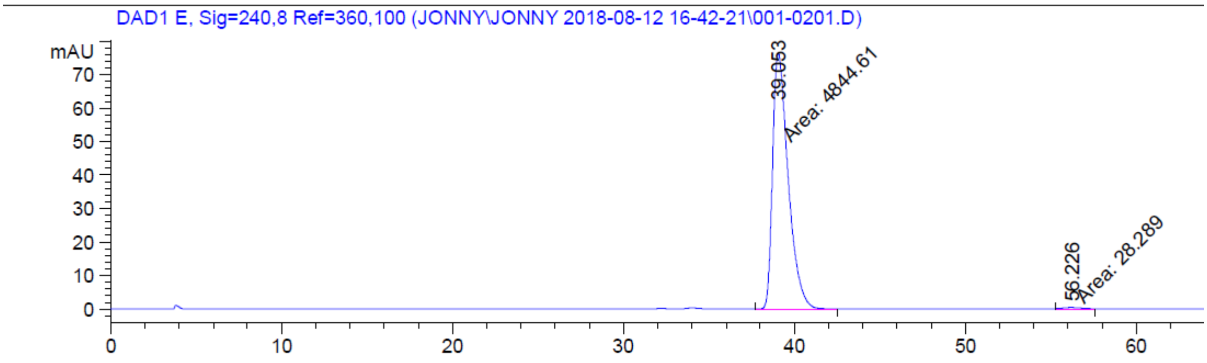

Signal 5: DAD1 E, Sig=240,8 Ref=360,100

Peak #	RetTime [min]	Type	Width [min]	Area [mAU*s]	Height [mAU]	Area %
1	39.053	MM	1.0545	4844.60693	76.56857	99.4195
2	56.226	MM	1.1533	28.28904	4.08822e-1	0.5805

Totals : 4872.89597 76.97739

(S)-3-methyl-4-((prop-2-yn-1-yloxy)methyl)cyclohex-2-en-1-one (2e)

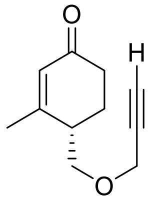

(Chiralpak AS-H, hexane/isopropanol = 70/30, 1 mL/min)

Racemic

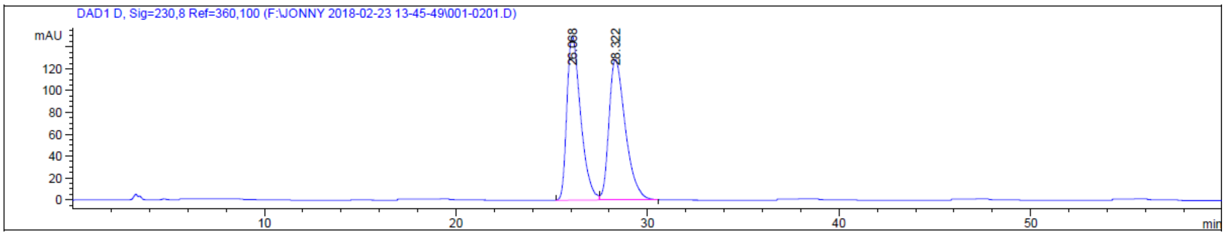

Signal 4: DAD1 D, Sig=230,8 Ref=360,100

Peak #	RetTime [min]	Type	Width [min]	Area [mAU*s]	Height [mAU]	Area %
1	26.068	BV	0.7612	7468.18262	150.44040	49.4210
2	28.322	VB	0.9115	7643.17139	128.58408	50.5790

Totals : 1.51114e4 279.02448

Enantiomerically enriched (99% ee)

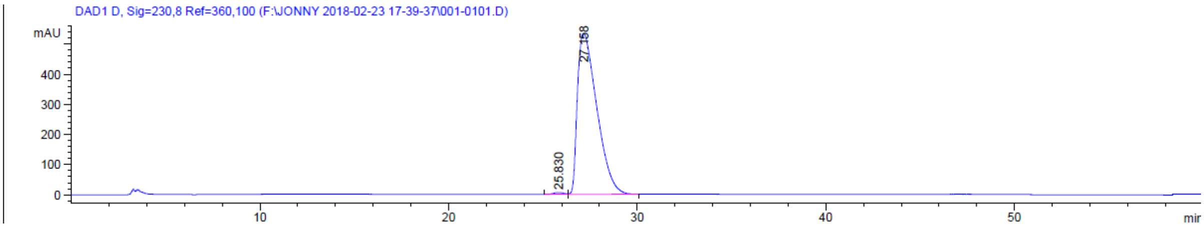

Signal 4: DAD1 D, Sig=230,8 Ref=360,100

Peak #	RetTime [min]	Type	Width [min]	Area [mAU*s]	Height [mAU]	Area %
1	25.830	BV	0.5954	259.36972	6.76373	0.6765
2	27.158	VB	1.1066	3.80824e4	533.64917	99.3235

Totals : 3.83418e4 540.41290

(S)-4-(((tert-butyldimethylsilyl)oxy)methyl)-3-methylcyclohex-2-en-1-one (2f)

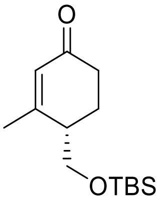

(Chiralpak AS-H, hexane/isopropanol = 90/10 to 70/30, 1 mL/min)

Gradient: t=0 min: 90/10 – t=25 min: 70/30 – t=55 min: 70/30 – t=55 min: 90/10 – t=60 min: 90/10

Racemic

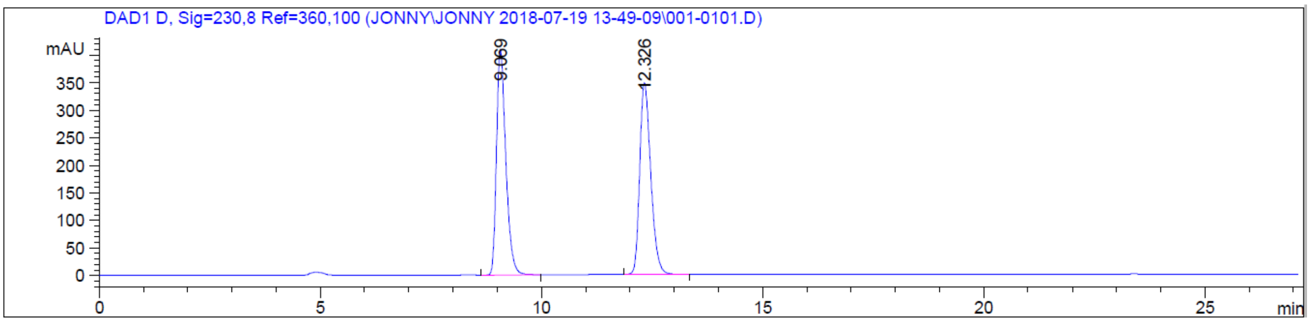

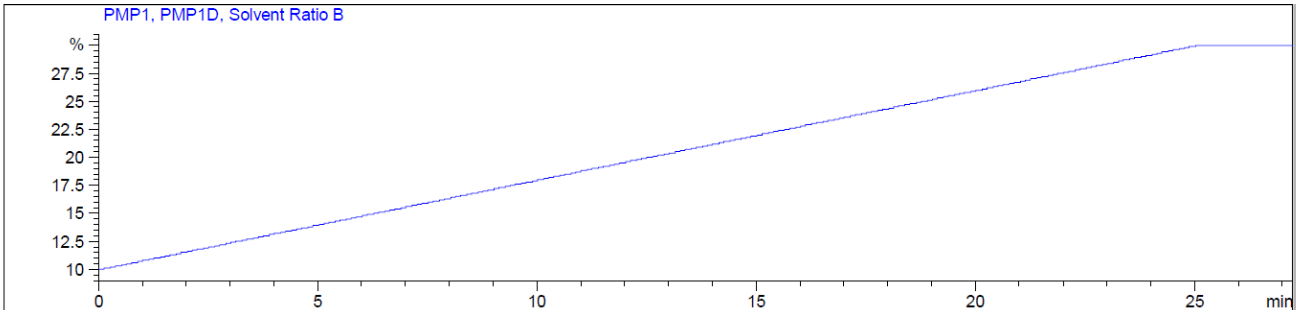

Signal 4: DAD1 D, Sig=230,8 Ref=360,100

Peak #	RetTime [min]	Type	Width [min]	Area [mAU*s]	Height [mAU]	Area %
1	9.069	VB	0.2223	5988.45996	410.18536	49.8863
2	12.326	BB	0.2658	6015.76416	347.71387	50.1137

Totals : 1.20042e4 757.89923

Enantiomerically enriched (99% ee)

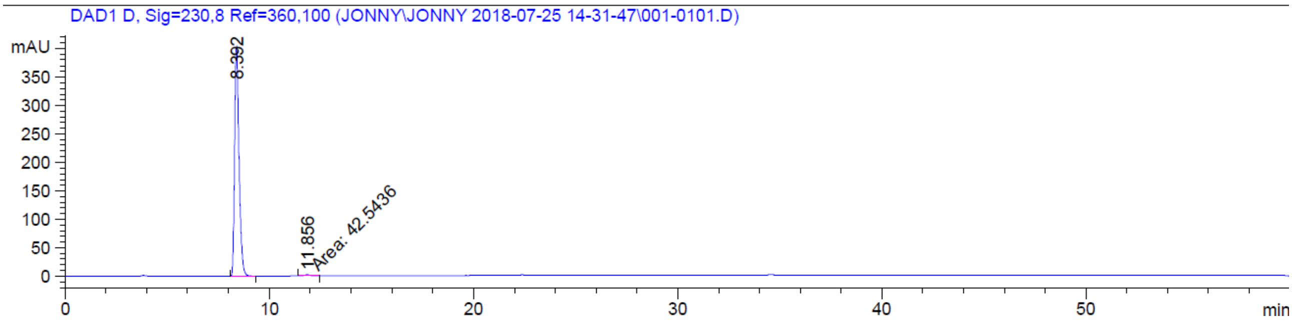

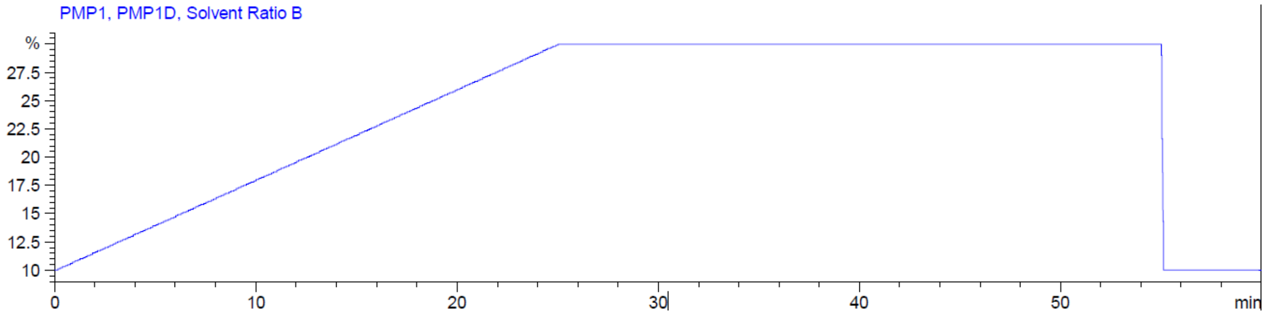

Signal 4: DAD1 D, Sig=230,8 Ref=360,100

Peak #	RetTime [min]	Type	Width [min]	Area [mAU*s]	Height [mAU]	Area %
1	8.392	BB	0.2309	6104.79980	402.59439	99.3079
2	11.856	MM	0.3494	42.54363	2.02953	0.6921

Totals : 6147.34343 404.62392

(S)-4-(hydroxymethyl)-3-methylcyclohex-2-en-1-one (2g)

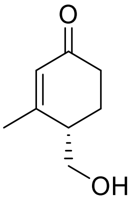

(Chiralpak AS-H, hexane/isopropanol = 70/30, 1 mL/min)

Racemic

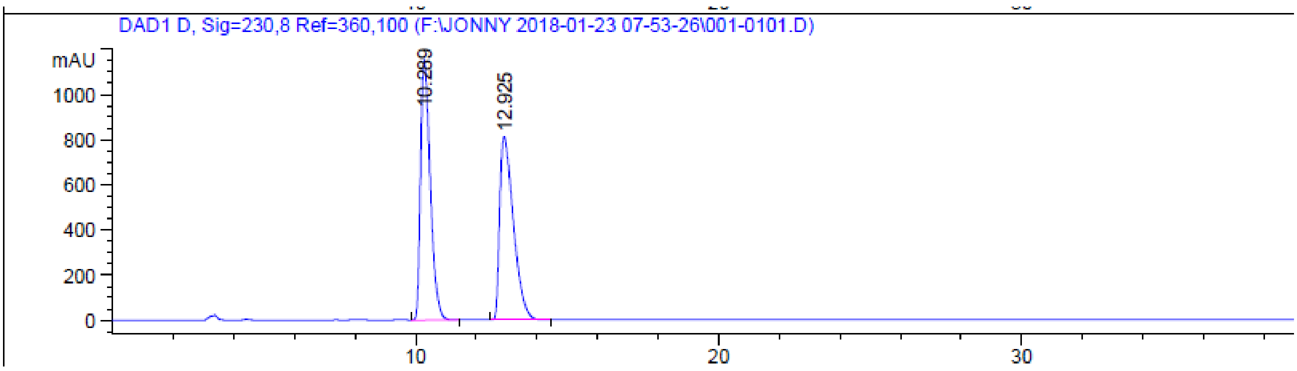

Signal 4: DAD1 D, Sig=230,8 Ref=360,100

Peak #	RetTime [min]	Type	Width [min]	Area [mAU*s]	Height [mAU]	Area %
1	10.289	BB	0.3400	2.53910e4	1148.01318	49.9178
2	12.925	BB	0.4861	2.54746e4	811.88940	50.0822
Totals :				5.08656e4	1959.90259	

Enantiomerically enriched (85% ee)

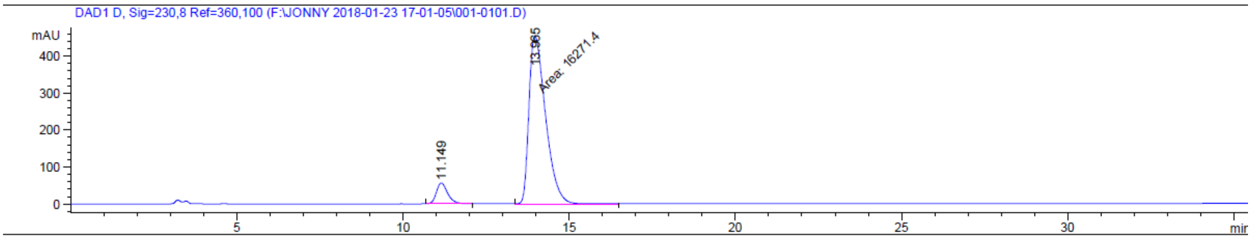

Signal 4: DAD1 D, Sig=230,8 Ref=360,100

Peak #	RetTime [min]	Type	Width [min]	Area [mAU*s]	Height [mAU]	Area %
1	11.149	BB	0.3575	1327.38489	56.61264	7.5425
2	13.965	MM	0.5930	1.62714e4	457.35071	92.4575
Totals :				1.75987e4	513.96334	

Enantiomerically enriched (99% ee)

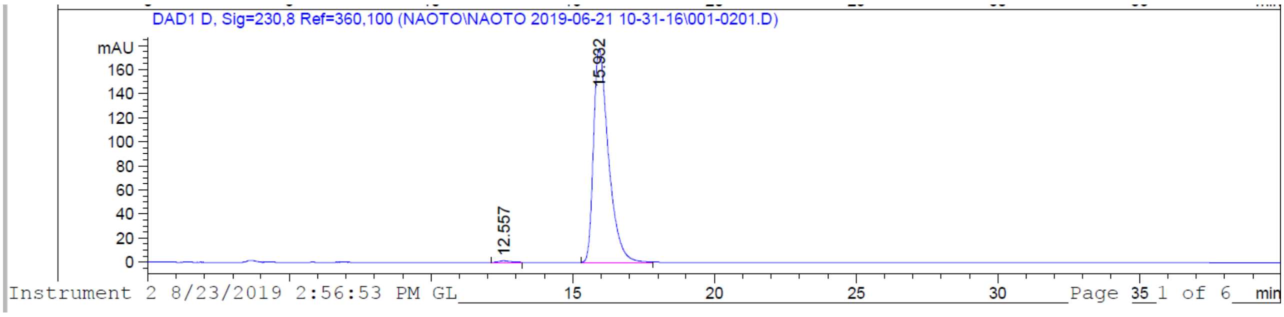

Instrument 2 8/23/2019 2:56:53 PM GL Page 35 1 of 6

Signal 4: DAD1 D, Sig=230,8 Ref=360,100

Peak #	RetTime [min]	Type	Width [min]	Area [mAU*s]	Height [mAU]	Area %
1	12.557	BB	0.3721	36.61283	1.39527	0.5592
2	15.932	BB	0.5495	6510.39063	177.69194	99.4408

Totals : 6547.00346 179.08721

(S)-4-((benzyloxy)methyl)cyclohex-2-en-1-one (2h)

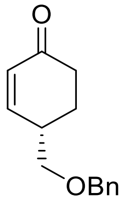

(Chiralpak AS-H, hexane/isopropanol = 70/30, 1 mL/min)

Racemic

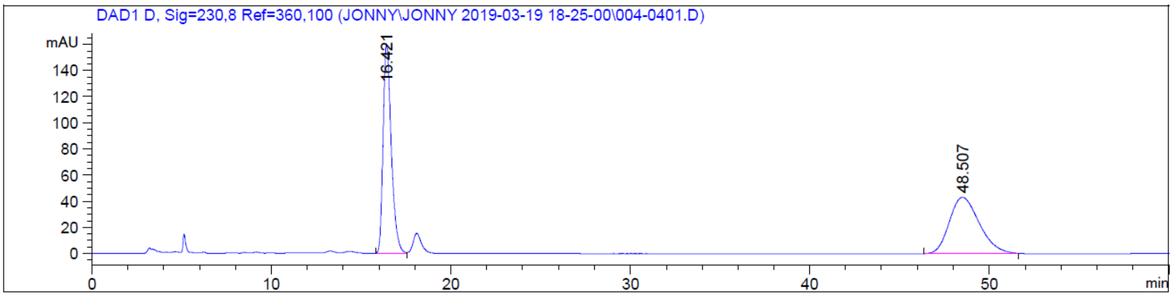

Signal 4: DAD1 D, Sig=230,8 Ref=360,100

Peak #	RetTime [min]	Type	Width [min]	Area [mAU*s]	Height [mAU]	Area %
1	16.421	BV	0.4814	5085.22900	160.60374	50.2528
2	48.507	BB	1.7827	5034.05957	43.22495	49.7472
Totals :				1.01193e4	203.82870	

Enantiomerically enriched (94% ee)

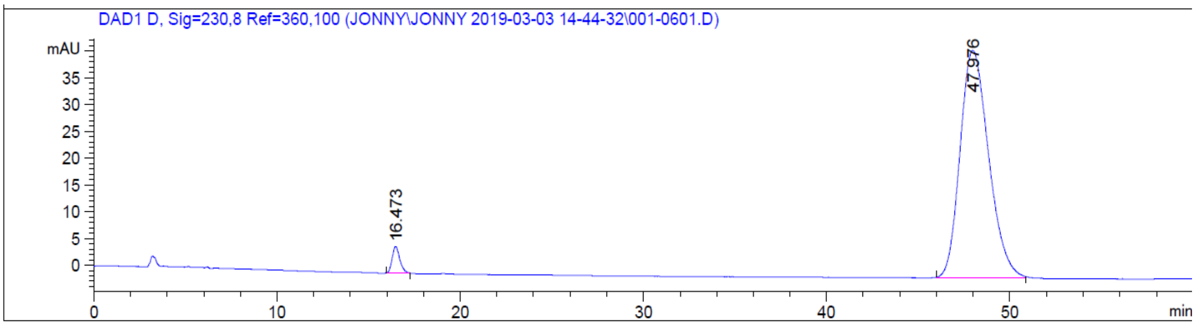

Signal 4: DAD1 D, Sig=230,8 Ref=360,100

Peak #	RetTime [min]	Type	Width [min]	Area [mAU*s]	Height [mAU]	Area %
1	16.473	BB	0.4442	147.03416	5.01325	3.0960
2	47.976	BB	1.6475	4602.13770	42.49578	96.9040
Totals :				4749.17186	47.50902	

tert-butyl (S)-((2-methyl-4-oxocyclohex-2-en-1-yl)methyl)(phenyl)carbamate (2i)

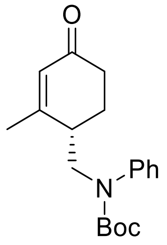

(Chiralpak AD-H, hexane/isopropanol = 90/10, 1 mL/min)

Racemic

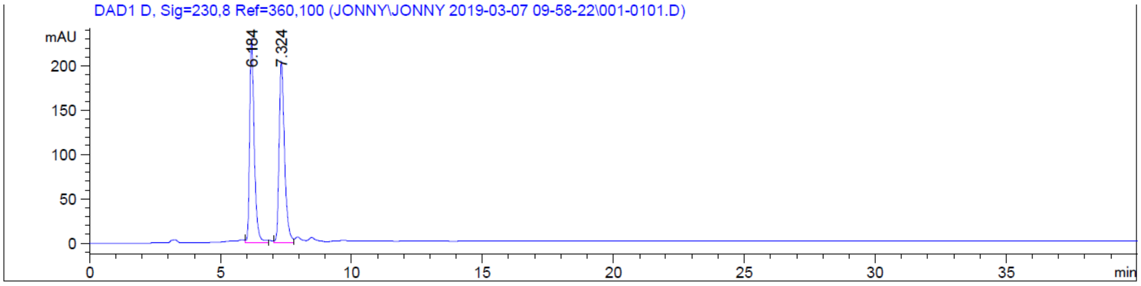

Signal 4: DAD1 D, Sig=230,8 Ref=360,100

Peak #	RetTime [min]	Type	Width [min]	Area [mAU*s]	Height [mAU]	Area %
1	6.184	VB	0.1862	2803.81885	229.98761	49.0076
2	7.324	BV	0.2189	2917.37793	203.97720	50.9924
Totals :				5721.19678	433.96481	

Enantiomerically enriched (98% ee)

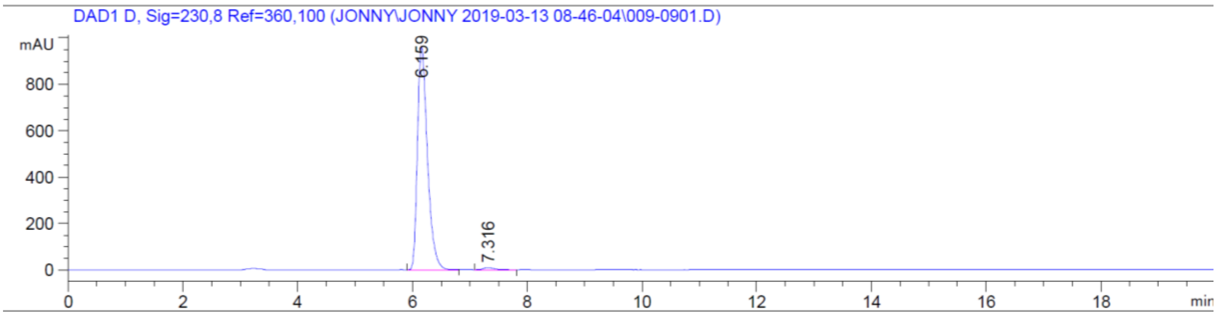

Signal 4: DAD1 D, Sig=230,8 Ref=360,100

Peak #	RetTime [min]	Type	Width [min]	Area [mAU*s]	Height [mAU]	Area %
1	6.159	VV	0.1855	1.16636e4	961.99121	98.9599
2	7.316	VB	0.2138	122.58715	8.72879	1.0401

Peak #	RetTime [min]	Type	Width [min]	Area [mAU*s]	Height [mAU]	Area %
Totals :				1.17862e4	970.72000	

tert-butyl (S)-((2-methyl-4-oxocyclohex-2-en-1-yl)methyl)(prop-2-yn-1-yl)carbamate (2j)

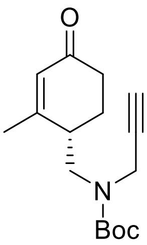

(Chiralpak AD, hexane/isopropanol = 90/10 to 70/30, 1 mL/min)

Gradient: t=0 min: 90/10 – t=25 min: 70/30 – t=55 min: 70/30 – t=55 min: 90/10 – t=60 min: 90/10

Racemic

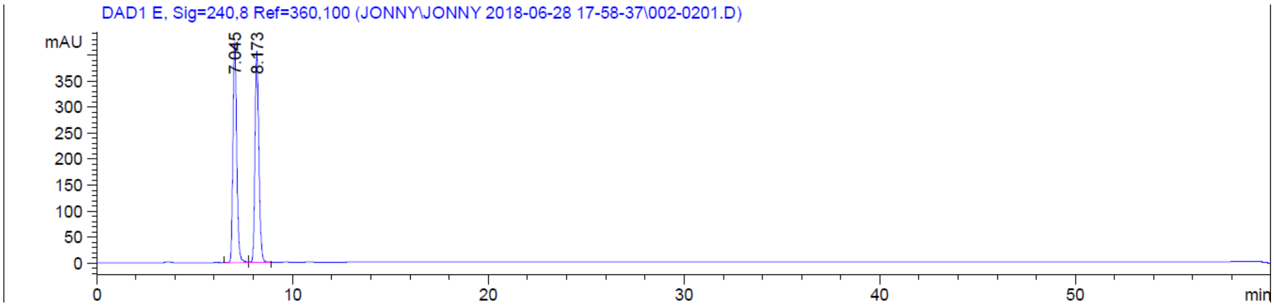

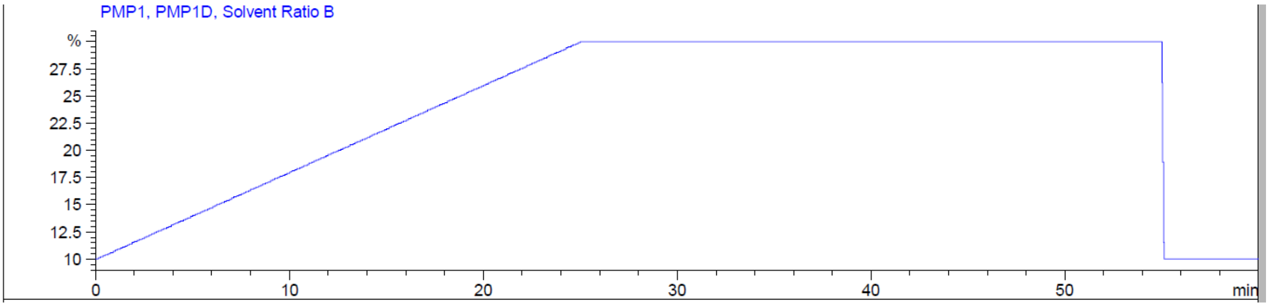

Signal 5: DAD1 E, Sig=240,8 Ref=360,100

Peak #	RetTime [min]	Type	Width [min]	Area [mAU*s]	Height [mAU]	Area %
1	7.045	BV	0.2080	5639.05811	421.73697	50.0425
2	8.173	VB	0.2141	5629.48730	405.18539	49.9575

Totals : 1.12685e4 826.92236

Enantiomerically enriched (96% ee)

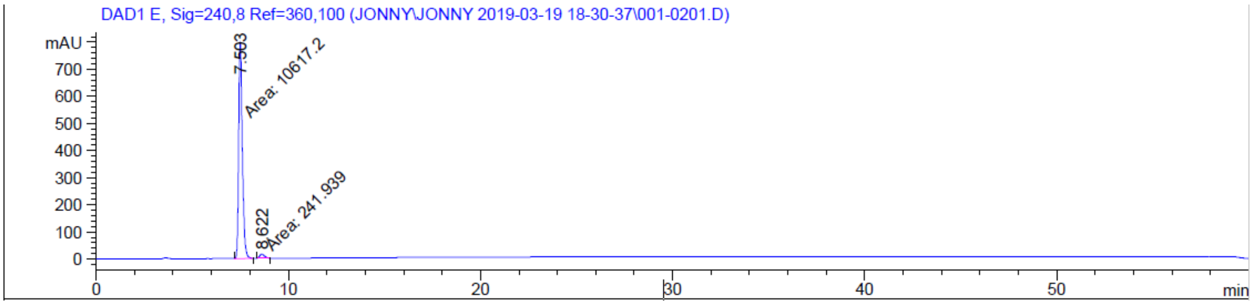

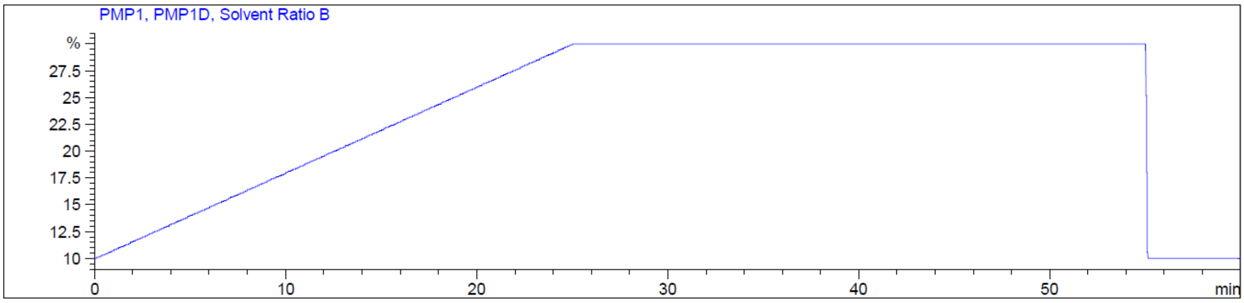

Signal 5: DAD1 E, Sig=240,8 Ref=360,100

Peak #	RetTime [min]	Type	Width [min]	Area [mAU*s]	Height [mAU]	Area %
1	7.503	MM	0.2225	1.06172e4	795.31525	97.7720
2	8.622	MM	0.2789	241.93925	14.45928	2.2280

Totals :	1.08591e4	809.77453

ert-butyl (S)-(1,3-dioxoisoindolin-2-yl)((2-methyl-4-oxocyclohex-2-en-1-yl)methyl)carbamate (2k)

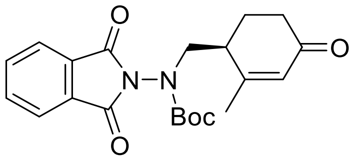

(Chiralpak AD, hexane/isopropanol = 90/10 to 70/30, 1 mL/min)

Gradient: t=0 min: 90/10 – t=25 min: 70/30 – t=55 min: 70/30 – t=55 min: 90/10 – t=60 min: 90/10

Racemic

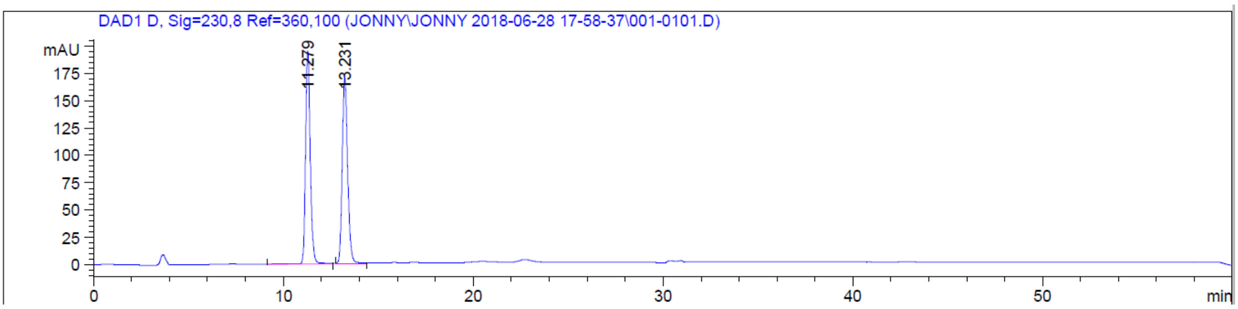

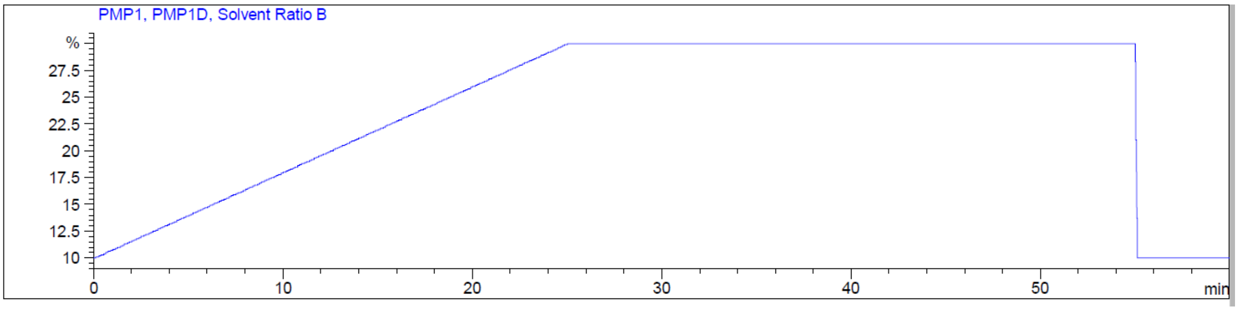

Signal 4: DAD1 D, Sig=230,8 Ref=360,100

Peak #	RetTime [min]	Type	Width [min]	Area [mAU*s]	Height [mAU]	Area %
1	11.279	VB	0.2675	3404.79419	195.19441	50.0962
2	13.231	BB	0.3073	3391.71851	171.05820	49.9038

Totals : 6796.51270 366.25261

Enantiomerically enriched (93% ee)

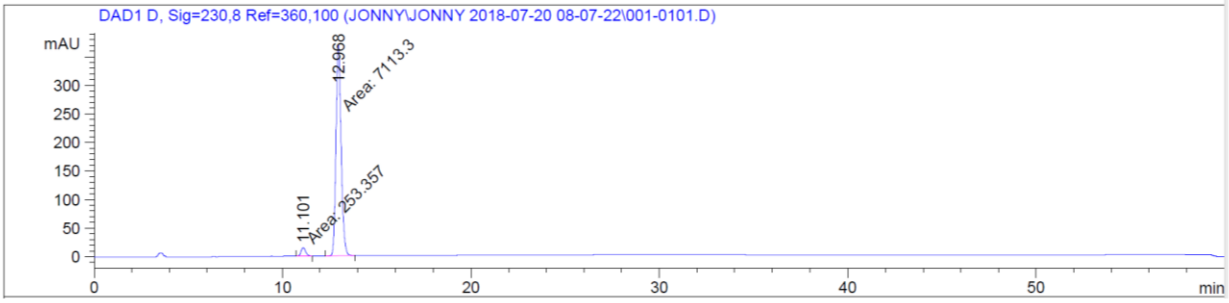

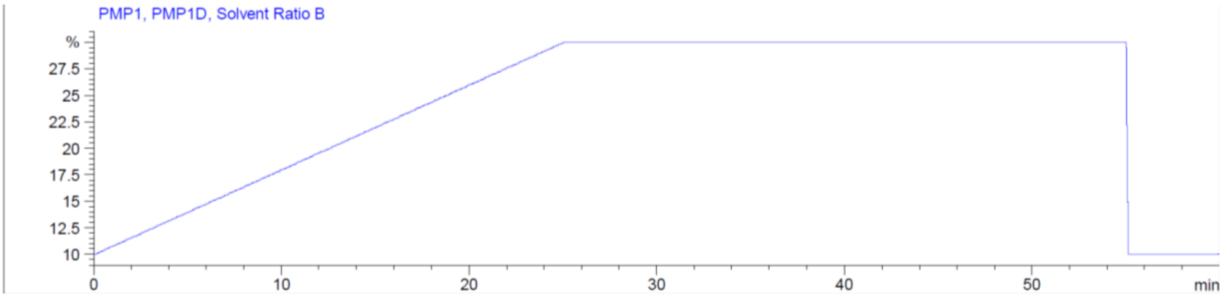

Signal 4: DAD1 D, Sig=230,8 Ref=360,100

Peak #	RetTime [min]	Type	Width [min]	Area [mAU*s]	Height [mAU]	Area %
1	11.101	MM	0.2793	253.35730	15.12096	3.4392
2	12.968	MM	0.3192	7113.30127	371.38730	96.5608

Totals : 7366.65857 386.50826

tert-butyl (S)-((tert-butyldimethylsilyl)oxy)((2-methyl-4-oxocyclohex-2-en-1-yl)methyl)carbamate (2l)

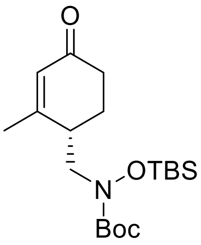

(Chiralpak AS-H, hexane/isopropanol = 90/10 to 70/30, 1 mL/min)

Gradient: t=0 min: 90/10 – t=25 min: 70/30 – t=55 min: 70/30 – t=55 min: 90/10 – t=60 min: 90/10

Racemic

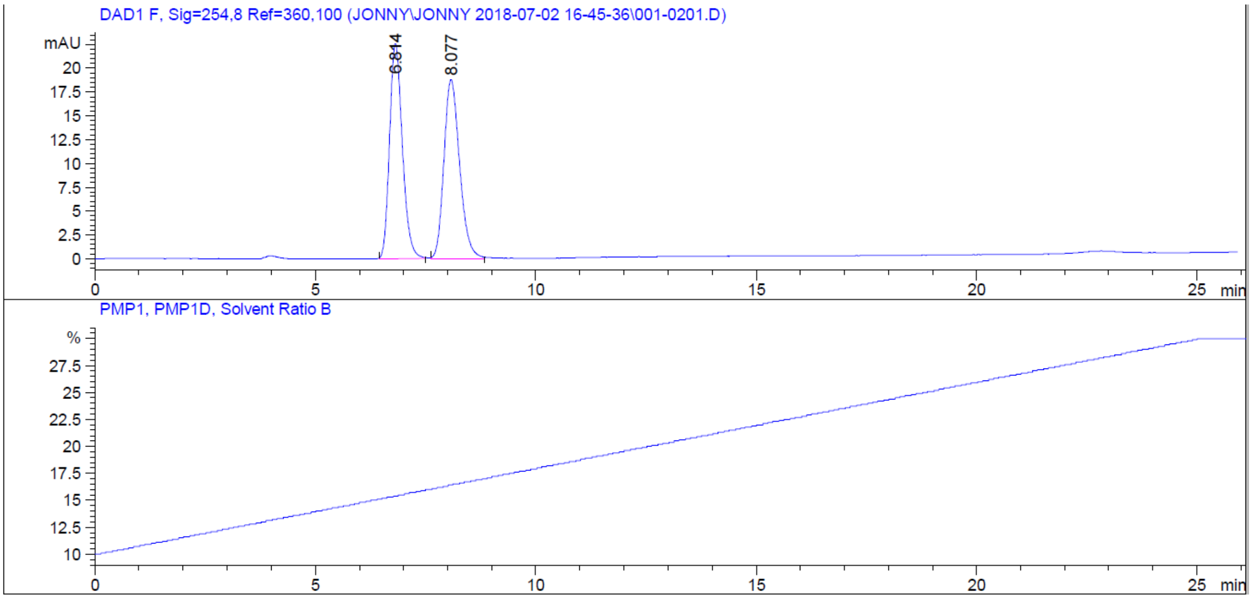

Signal 6: DAD1 F, Sig=254,8 Ref=360,100

Peak #	RetTime [min]	Type	Width [min]	Area [mAU*s]	Height [mAU]	Area %
1	6.814	BB	0.3117	456.91620	22.61480	49.6335
2	8.077	BB	0.3838	463.66428	18.79531	50.3665

Totals : 920.58047 41.41011

Enantiomerically enriched (94% ee)

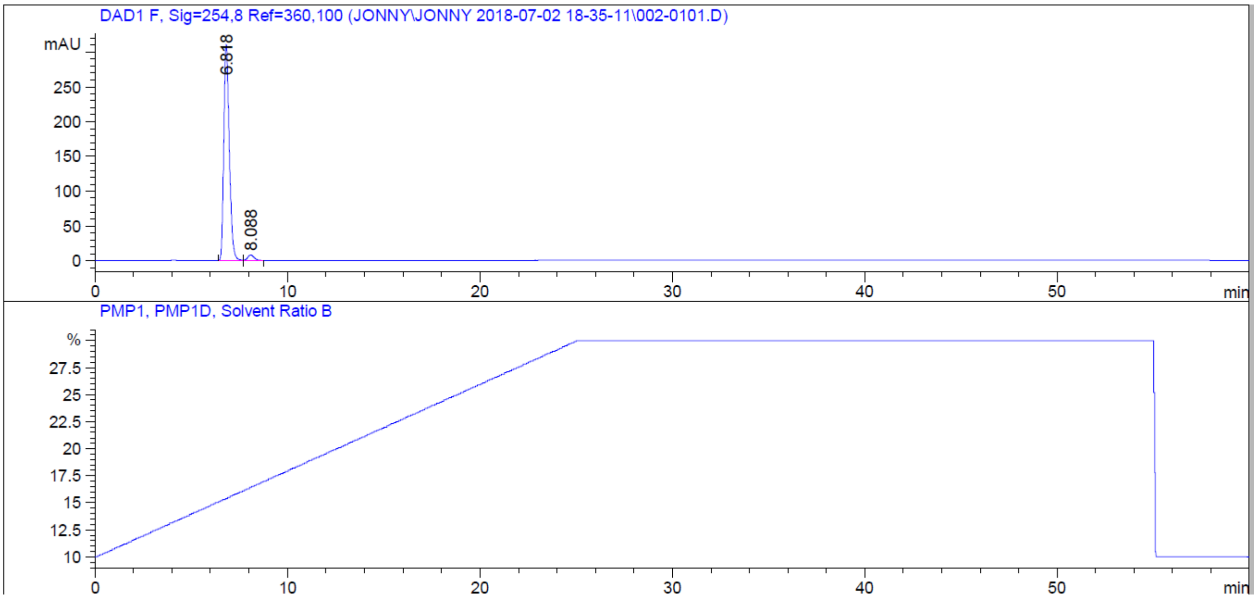

Signal 6: DAD1 F, Sig=254,8 Ref=360,100

Peak #	RetTime [min]	Type	Width [min]	Area [mAU*s]	Height [mAU]	Area %
1	6.818	BV	0.3087	6194.50977	310.60507	96.8416
2	8.088	VB	0.3786	202.02948	8.11148	3.1584

Totals : 6396.53925 318.71656

(S)-4-((1H-indol-1-yl)methyl)-3-methylcyclohex-2-en-1-one (2m)

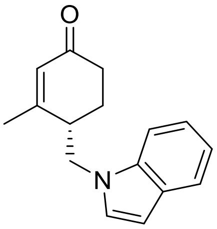

(Chiralpak AD-H, hexane/isopropanol = 95/5, 1 mL/min)

Racemic

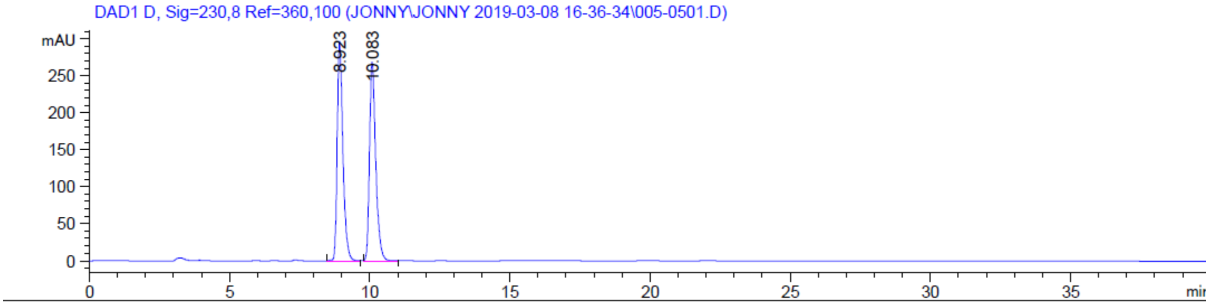

Signal 4: DAD1 D, Sig=230,8 Ref=360,100

Peak #	RetTime [min]	Type	Width [min]	Area [mAU*s]	Height [mAU]	Area %
1	8.923	BB	0.2122	4168.90039	296.24362	49.9874
2	10.083	BB	0.2353	4171.00049	268.33078	50.0126

Totals : 8339.90088 564.57440

Enantiomerically enriched (90% ee)

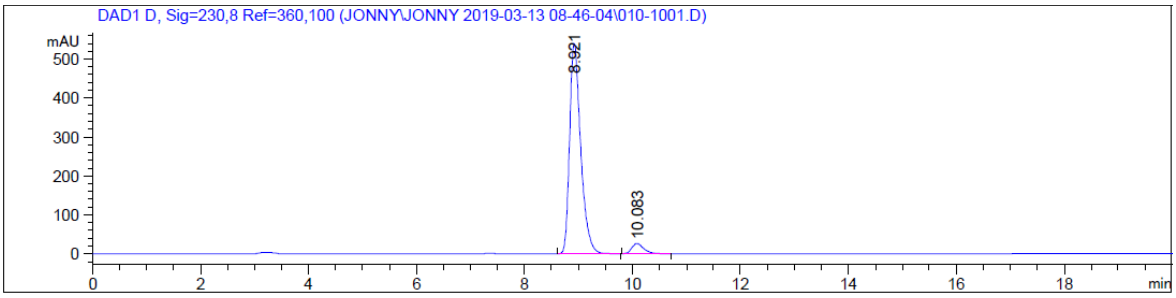

Signal 4: DAD1 D, Sig=230,8 Ref=360,100

Peak #	RetTime [min]	Type	Width [min]	Area [mAU*s]	Height [mAU]	Area %
1	8.921	BB	0.2164	7699.76367	539.79749	94.9232
2	10.083	BB	0.2347	411.81213	26.58802	5.0768

Peak #	RetTime [min]	Type	Width [min]	Area [mAU*s]	Height [mAU]	Area %
Totals :				8111.57581	566.38551	

tert-butyl (S)-furan-2-yl((2-methyl-4-oxocyclohex-2-en-1-yl)methyl)carbamate (2n)

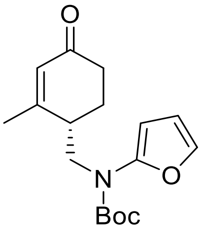

(Chiralpak AD-H, hexane/isopropanol = 90/10, 1 mL/min)

Racemic

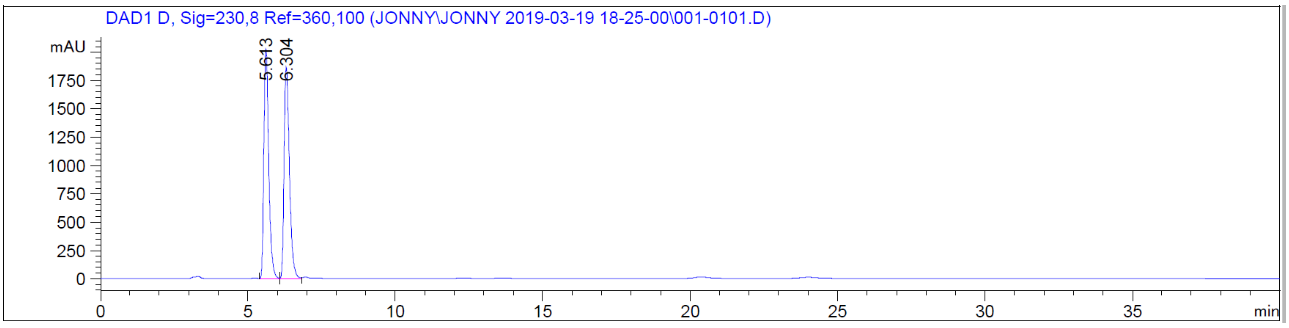

Signal 4: DAD1 D, Sig=230,8 Ref=360,100

Peak #	RetTime [min]	Type	Width [min]	Area [mAU*s]	Height [mAU]	Area %
1	5.613	VV	0.1779	2.32727e4	2027.19385	49.8410
2	6.304	VV	0.1946	2.34212e4	1863.62048	50.1590
Totals :				4.66940e4	3890.81433	

Enantiomerically enriched (99% ee)

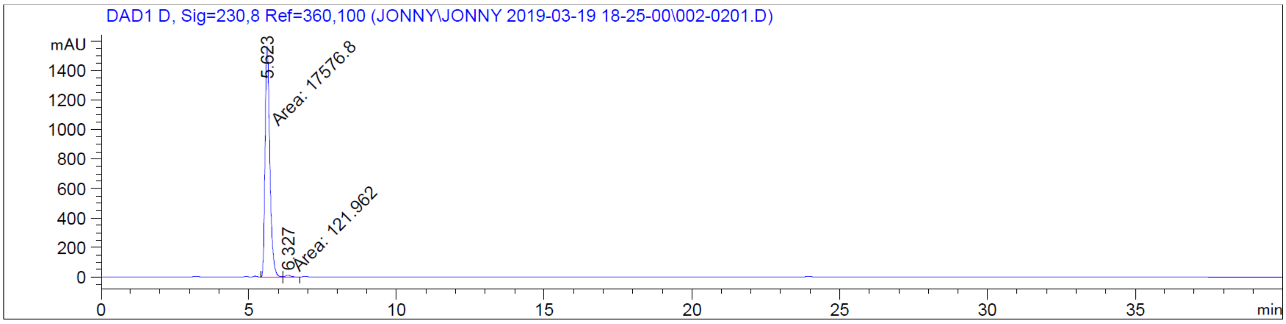

Signal 4: DAD1 D, Sig=230,8 Ref=360,100

Peak #	RetTime [min]	Type	Width [min]	Area [mAU*s]	Height [mAU]	Area %
1	5.623	MM	0.1875	1.75768e4	1562.52673	99.3109
2	6.327	MM	0.1912	121.96220	10.62852	0.6891
Totals :				1.76987e4	1573.15525	

benzyl (S)-furan-2-yl((2-methyl-4-oxocyclohex-2-en-1-yl)methyl)carbamate (2o)

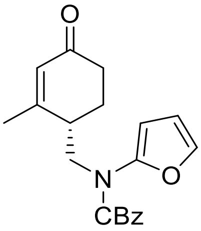

(Chiralpak IA, hexane/isopropanol = 90/10, 1 mL/min)

Racemic

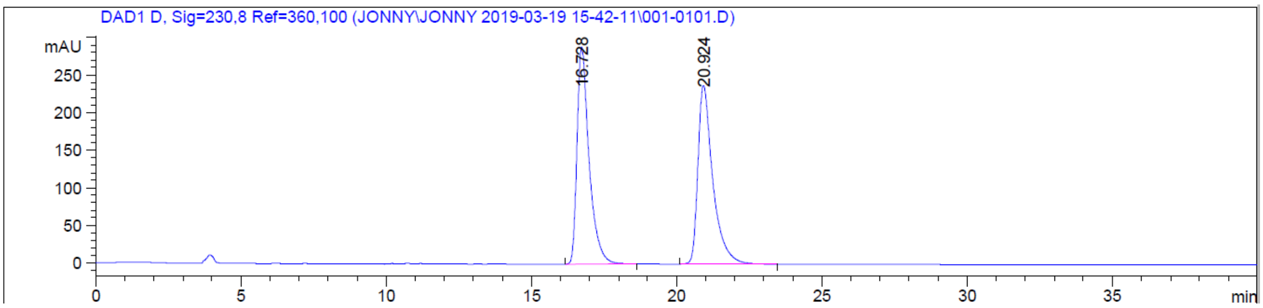

Signal 4: DAD1 D, Sig=230,8 Ref=360,100

Peak #	RetTime [min]	Type	Width [min]	Area [mAU*s]	Height [mAU]	Area %
1	16.728	BB	0.4284	8323.62695	288.69269	49.5246
2	20.924	BB	0.5284	8483.44531	237.71533	50.4754
Totals :				1.68071e4	526.40802	

Enantiomerically enriched (97% ee)

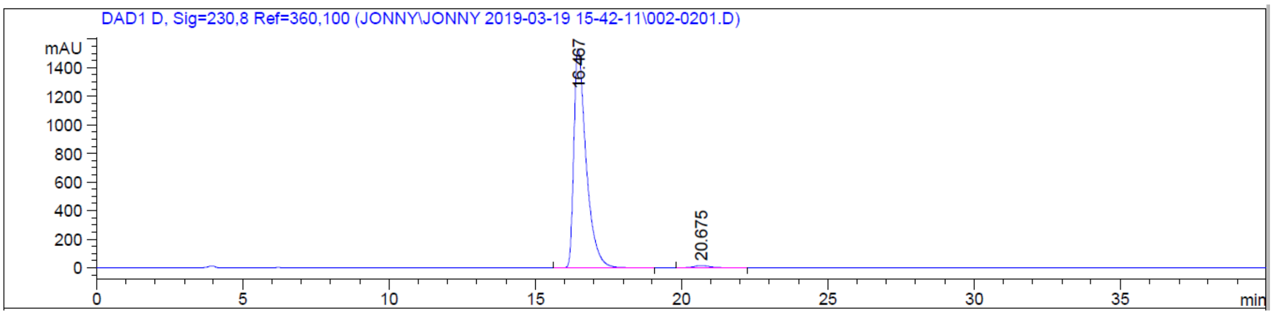

Signal 4: DAD1 D, Sig=230,8 Ref=360,100

Peak #	RetTime [min]	Type	Width [min]	Area [mAU*s]	Height [mAU]	Area %
1	16.467	BB	0.4368	4.47903e4	1533.11121	98.5676
2	20.675	BB	0.5839	650.91125	16.23034	1.4324
Totals :				4.54413e4	1549.34154	

(R)-5',6'-dihydro-[1,1':2',1''-terphenyl]-4'(1'H)-one (2p)

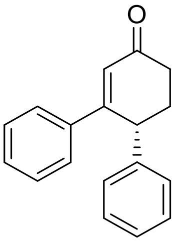

(Chiralpak OD-H, hexane/isopropanol = 90/10, 1 mL/min)

Racemic

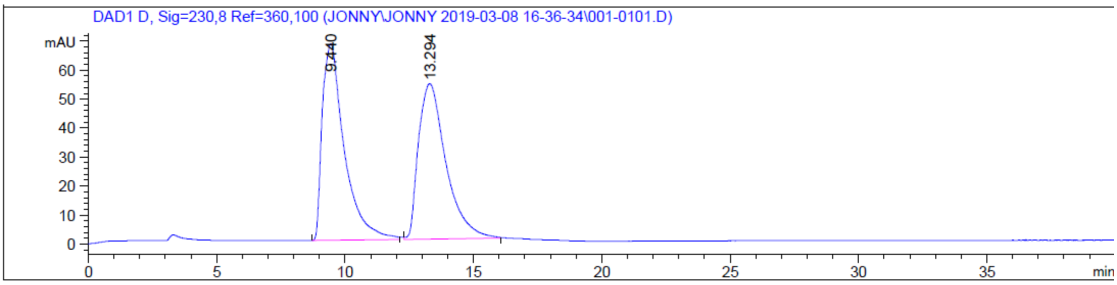

Signal 4: DAD1 D, Sig=230,8 Ref=360,100

Peak #	RetTime [min]	Type	Width [min]	Area [mAU*s]	Height [mAU]	Area %
1	9.440	BB	0.8942	4039.70679	67.69150	50.3764
2	13.294	BB	1.1619	3979.34692	53.48973	49.6236

Peak #	RetTime [min]	Type	Width [min]	Area [mAU*s]	Height [mAU]	Area %
Totals :				8019.05371	121.18122	

Enantiomerically enriched (94% ee)

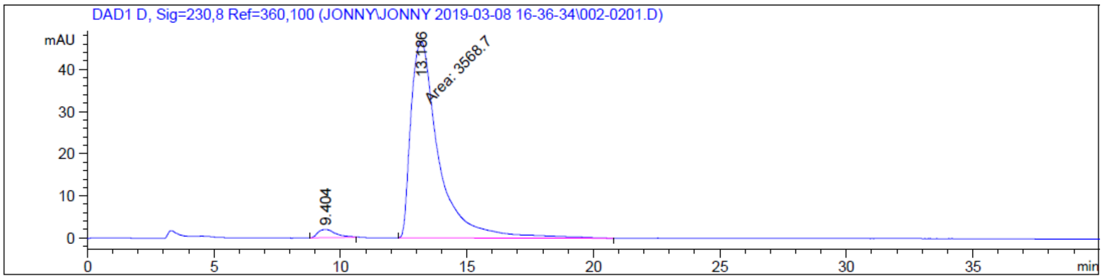

Signal 4: DAD1 D, Sig=230,8 Ref=360,100

Peak #	RetTime [min]	Type	Width [min]	Area [mAU*s]	Height [mAU]	Area %
1	9.404	BB	0.6764	101.29274	2.02225	2.7600
2	13.186	MM	1.2741	3568.69653	46.68128	97.2400
Totals :				3669.98927	48.70353	

(S)-3,4-diethylcyclohex-2-en-1-one (2q)

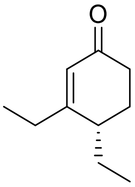

(Chiralpak AS-H, hexane/isopropanol = 70/30, 1 mL/min)

Racemic

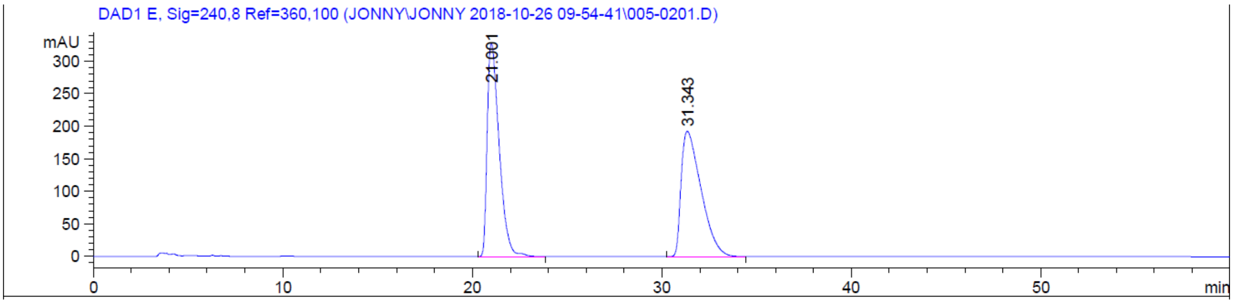

Signal 5: DAD1 E, Sig=240,8 Ref=360,100

Peak #	RetTime [min]	Type	Width [min]	Area [mAU*s]	Height [mAU]	Area %
1	21.001	BB	0.6745	1.43800e4	328.65839	50.5456
2	31.343	BB	1.1140	1.40695e4	193.11928	49.4544
Totals :				2.84495e4	521.77766	

Enantiomerically enriched (97% ee)

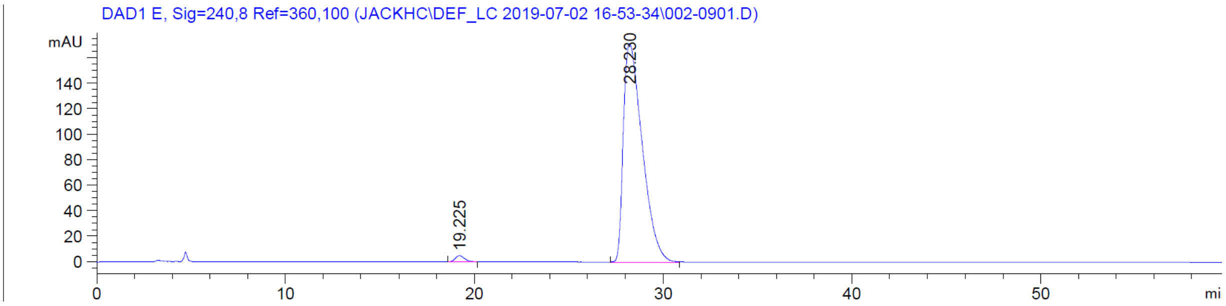

Signal 5: DAD1 E, Sig=240,8 Ref=360,100

Peak #	RetTime [min]	Type	Width [min]	Area [mAU*s]	Height [mAU]	Area %
1	19.225	BB	0.5213	166.74107	4.84712	1.3801
2	28.230	BB	1.0697	1.19150e4	171.67178	98.6199
Totals :				1.20817e4	176.51890	

(S)-3,4-dipropylcyclohex-2-en-1-one (2r)

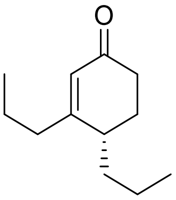

(Chiralpak AS-H, hexane/isopropanol = 90/10 to 70/30, 1 mL/min)

Gradient: t=0 min: 90/10 – t=25 min: 70/30 – t=55 min: 70/30 – t=55 min: 90/10 – t=60 min: 90/10

Racemic

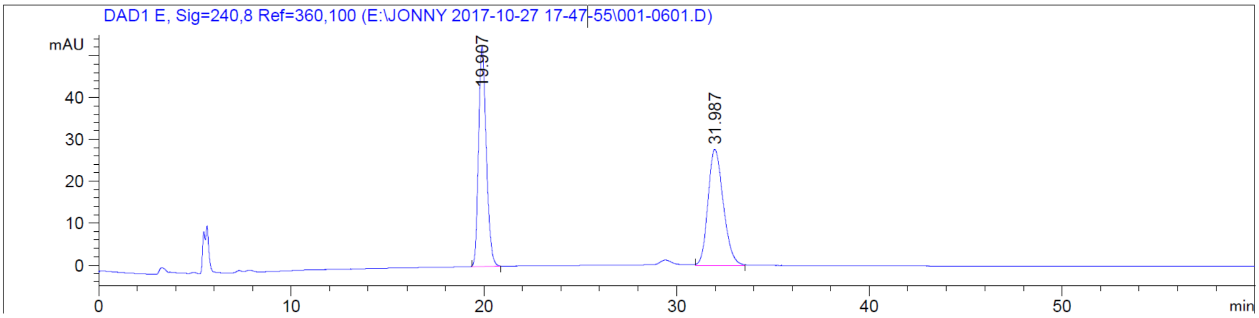

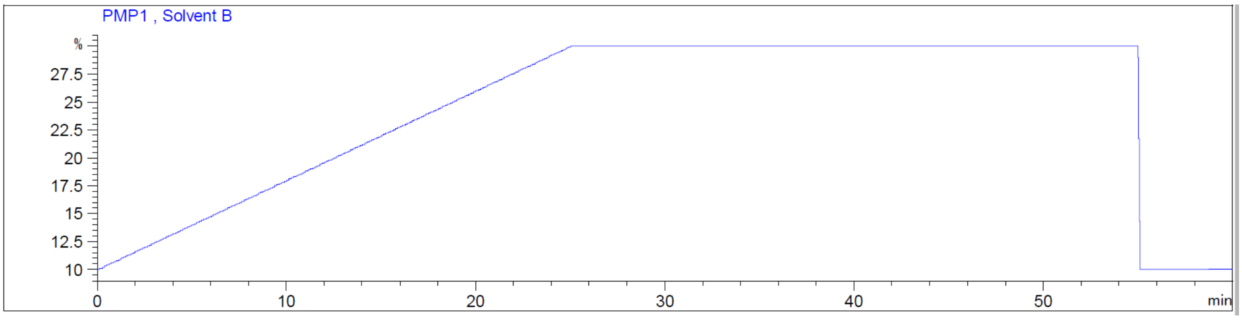

Signal 5: DAD1 E, Sig=240,8 Ref=360,100

Peak #	RetTime [min]	Type	Width [min]	Area [mAU*s]	Height [mAU]	Area %
1	19.907	BB	0.4295	1468.44519	52.62455	49.1561
2	31.987	BB	0.8480	1518.86768	27.68671	50.8439

Totals : 2987.31287 80.31126

Enantiomerically enriched (97% ee)

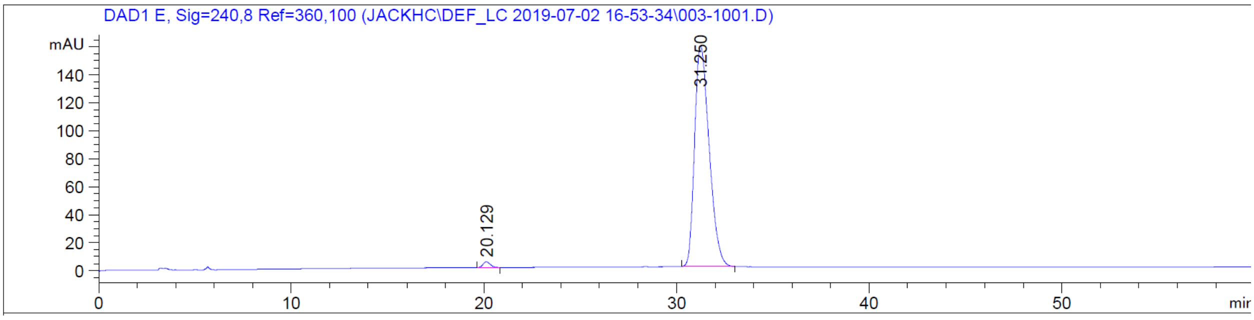

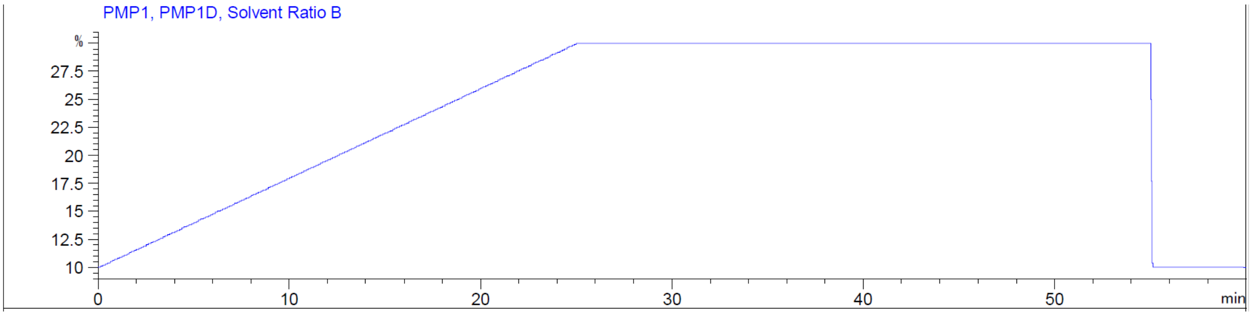

Signal 5: DAD1 E, Sig=240,8 Ref=360,100

Peak #	RetTime [min]	Type	Width [min]	Area [mAU*s]	Height [mAU]	Area %
1	20.129	BB	0.4088	109.45236	4.13475	1.2990
2	31.250	BB	0.8182	8316.56836	157.97891	98.7010

Totals : 8426.02072 162.11366

(S)-6-methyl-5,6-dihydro-[1,1'-biphenyl]-3(4H)-one (2s)

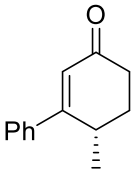

(Chiralpak OD-H, hexane/isopropanol = 85/15, 1 mL/min)

Racemic

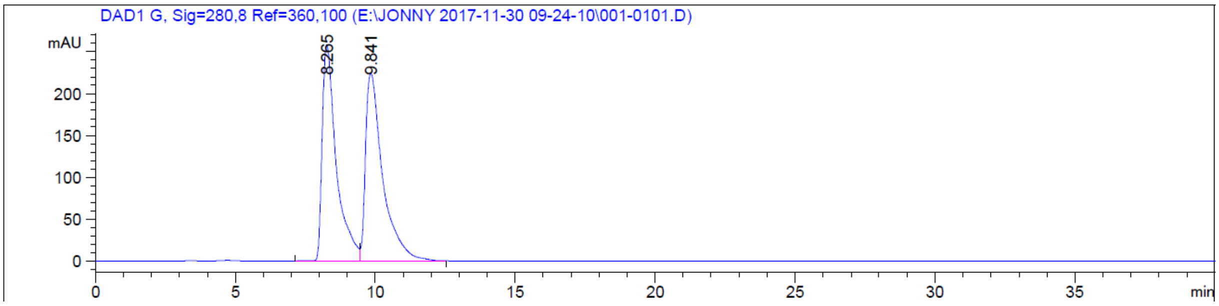

Signal 7: DAD1 G, Sig=280,8 Ref=360,100

Peak #	RetTime [min]	Type	Width [min]	Area [mAU*s]	Height [mAU]	Area %
1	8.265	BV	0.5267	9083.68652	258.06824	48.5998
2	9.841	VB	0.6366	9607.10645	223.89732	51.4002
Totals :				1.86908e4	481.96556	

Enantiomerically enriched (95% ee)

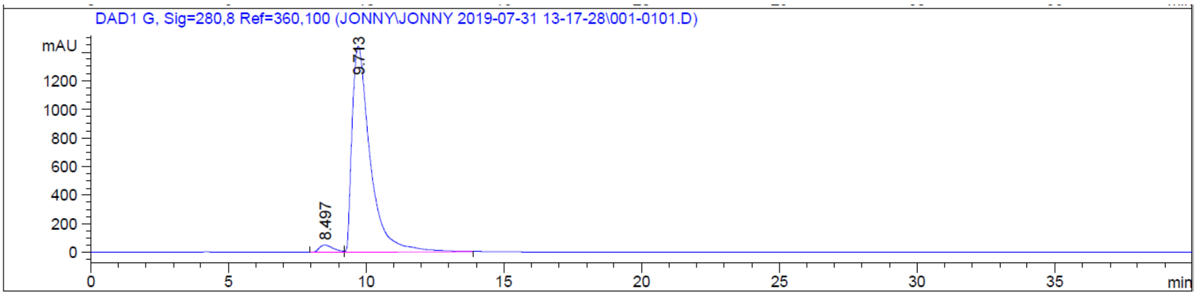

Signal 7: DAD1 G, Sig=280,8 Ref=360,100

Peak #	RetTime [min]	Type	Width [min]	Area [mAU*s]	Height [mAU]	Area %
1	8.497	BV	0.5495	1810.90759	51.09455	2.6618
2	9.713	VB	0.6968	6.62215e4	1439.25488	97.3382
Totals :				6.80324e4	1490.34943	

(S)-4'-methoxy-6-methyl-5,6-dihydro-[1,1'-biphenyl]-3(4H)-one (2t)

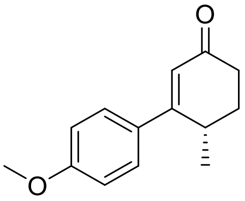

(Chiralpak AD-H, hexane/isopropanol = 90/10 to 70/30, 1 mL/min)

Gradient: t=0 min: 90/10 – t=25 min: 70/30 – t=55 min: 70/30 – t=55 min: 90/10 – t=60 min: 90/10

Racemic

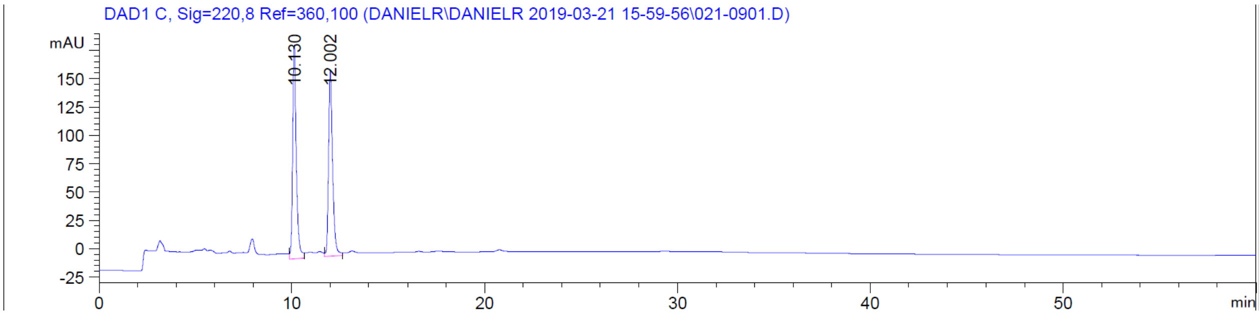

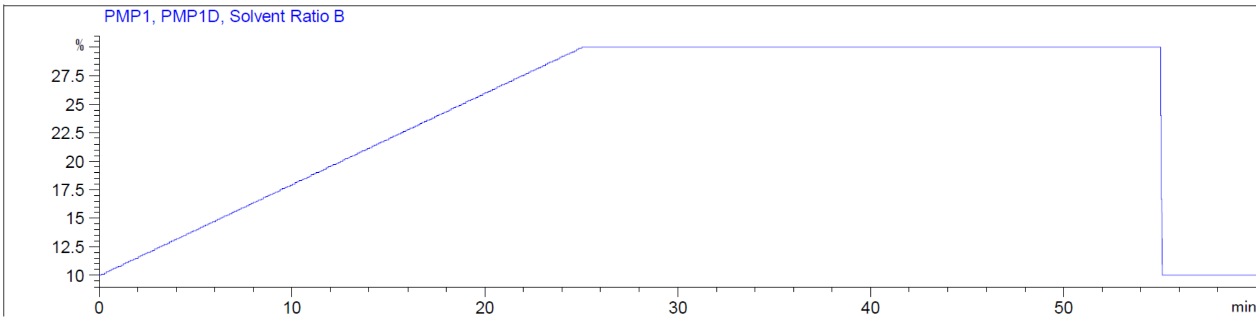

Signal 3: DAD1 C, Sig=220,8 Ref=360,100

Peak #	RetTime [min]	Type	Width [min]	Area [mAU*s]	Height [mAU]	Area %
1	10.130	BV	0.1989	2512.85278	189.26759	51.2748
2	12.002	VB	0.2208	2387.90283	163.14302	48.7252

Totals : 4900.75562 352.41061

Enantiomerically enriched (93% ee)

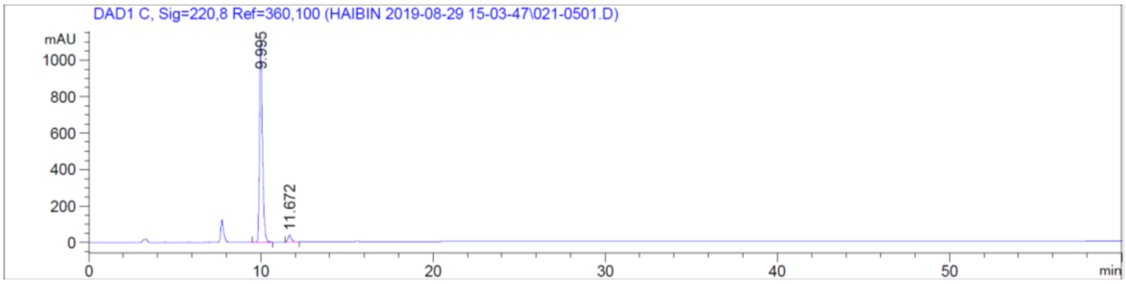

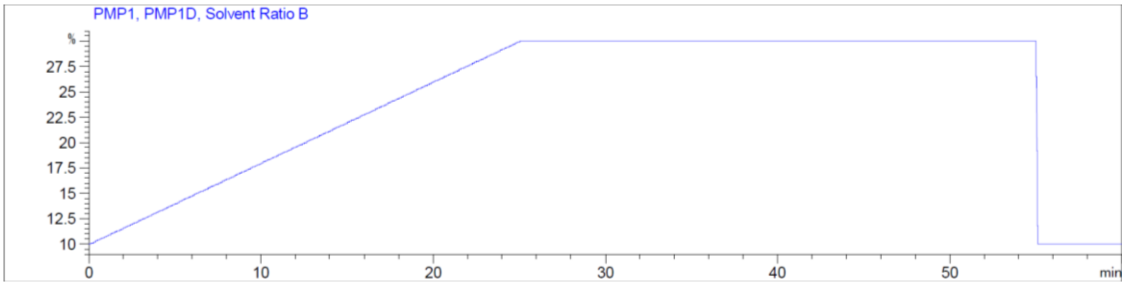

Signal 3: DAD1 C, Sig=220,8 Ref=360,100

Peak #	RetTime [min]	Type	Width [min]	Area [mAU*s]	Height [mAU]	Area %
1	9.995	BB	0.1808	1.31082e4	1101.80310	96.2998
2	11.672	BB	0.1954	503.66174	38.80825	3.7002

Totals : 1.36119e4 1140.61135

(S)-(2-methyl-4-oxocyclohex-2-en-1-yl)methyl 4-methylbenzenesulfonate (4a)

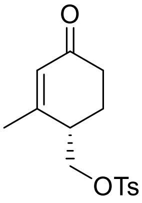

(Chiralpak AD-H, hexane/isopropanol = 90/10 to 70/30, 1 mL/min)

Gradient: t=0 min: 90/10 – t=25 min: 70/30 – t=55 min: 70/30 – t=55 min: 90/10 – t=60 min: 90/10

Racemic

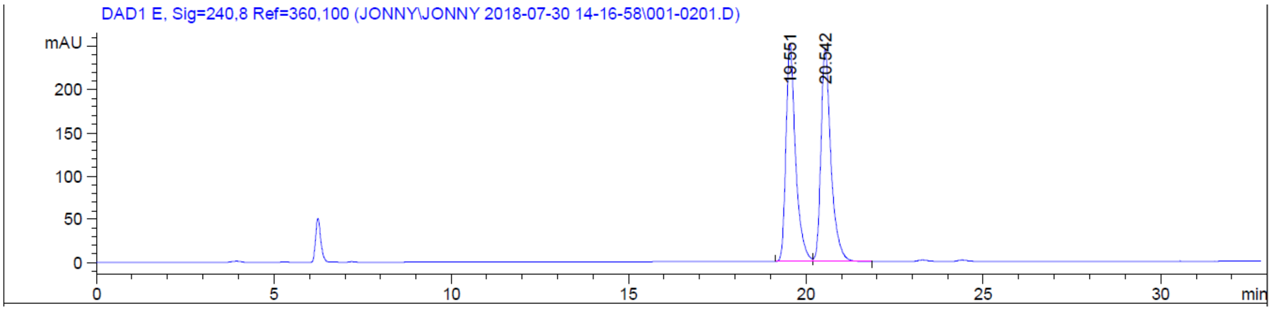

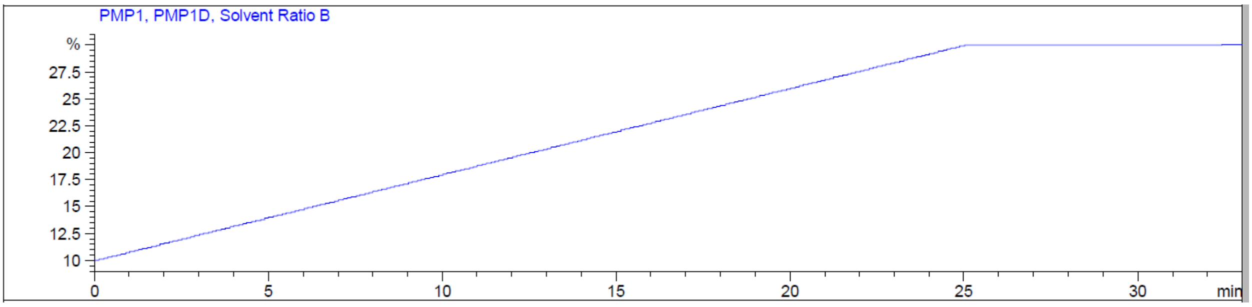

Signal 5: DAD1 E, Sig=240,8 Ref=360,100

Peak #	RetTime [min]	Type	Width [min]	Area [mAU*s]	Height [mAU]	Area %
1	19.551	BV	0.2834	4739.95898	252.01918	49.6264
2	20.542	VB	0.2961	4811.31787	246.10129	50.3736
Totals :				9551.27686	498.12047	

Enantiomerically enriched (99% ee)

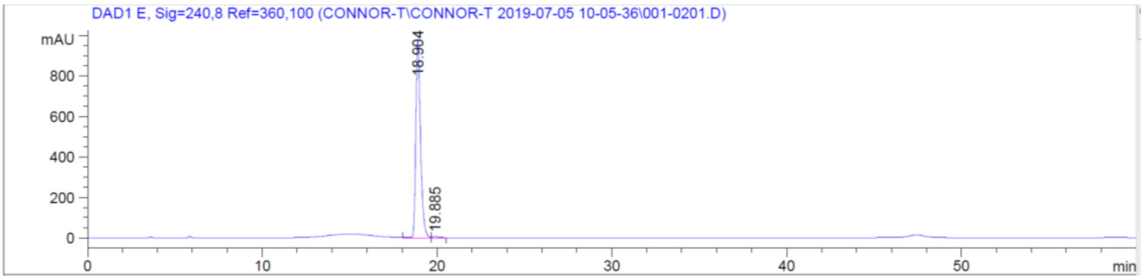

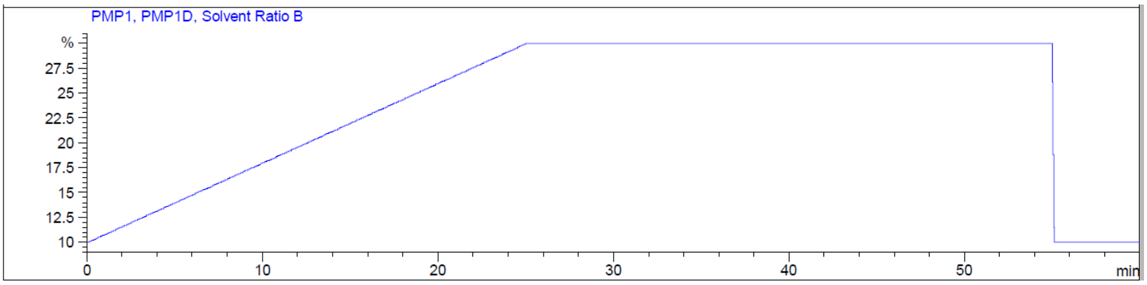

Signal 5: DAD1 E, Sig=240,8 Ref=360,100

Peak #	RetTime [min]	Type	Width [min]	Area [mAU*s]	Height [mAU]	Area %
1	18.904	VV	0.2808	1.81411e4	976.06671	99.2915
2	19.885	VB	0.2914	129.44725	6.52614	0.7085

Totals : 1.82705e4 982.59285

(S)-4-(azidomethyl)-3-methylcyclohex-2-en-1-one (4b)

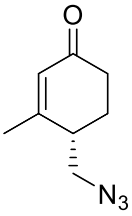

(Chiralpak AS-H, hexane/isopropanol = 70/30, 1 mL/min)

Racemic

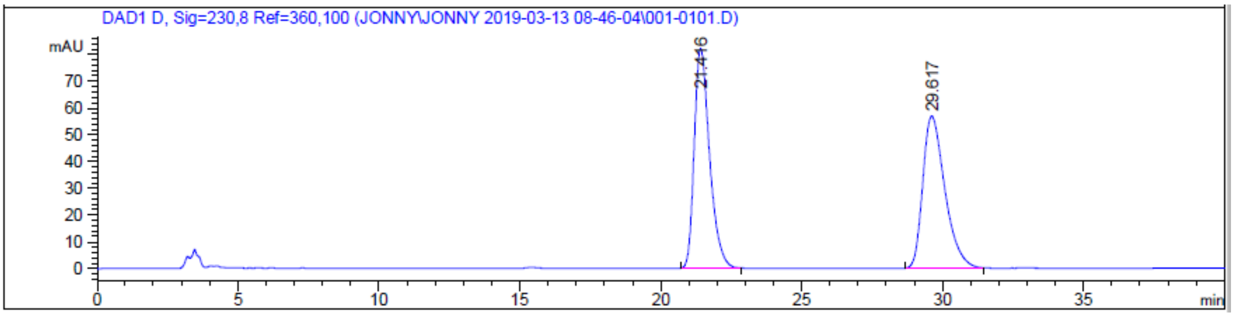

Signal 4: DAD1 D, Sig=230,8 Ref=360,100

Peak #	RetTime [min]	Type	Width [min]	Area [mAU*s]	Height [mAU]	Area %
1	21.416	BB	0.5771	3107.86377	82.20157	50.0235
2	29.617	BB	0.8324	3104.93774	56.92233	49.9765

Totals : 6212.80151 139.12389

Enantiomerically enriched (99% ee)

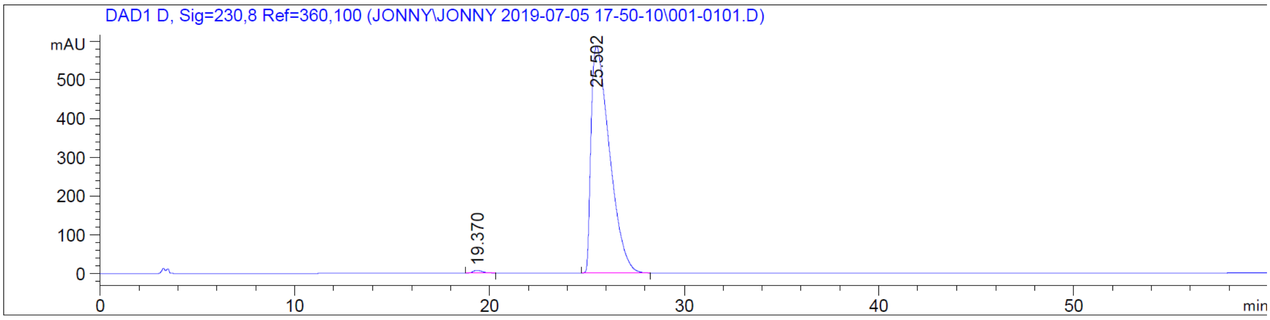

Signal 4: DAD1 D, Sig=230,8 Ref=360,100

Peak #	RetTime [min]	Type	Width [min]	Area [mAU*s]	Height [mAU]	Area %
1	19.370	BB	0.4887	231.30156	7.20183	0.5964
2	25.502	BB	1.0092	3.85505e4	586.23370	99.4036

Totals : 3.87818e4 593.43553

(S)-3-methyl-4-((phenylthio)methyl)cyclohex-2-en-1-one (4c)

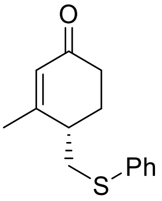

(Chiralpak AS-H, hexane/isopropanol = 70/30, 1 mL/min)

Racemic

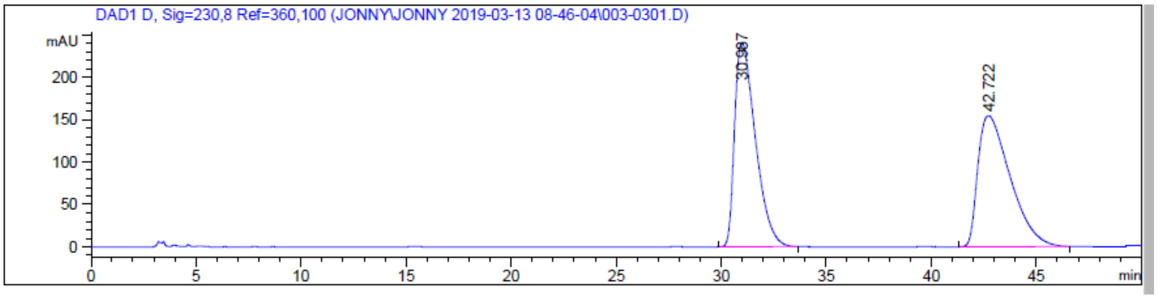

Signal 4: DAD1 D, Sig=230,8 Ref=360,100

Peak #	RetTime [min]	Type	Width [min]	Area [mAU*s]	Height [mAU]	Area %
1	30.987	BB	1.0654	1.65594e4	241.05490	50.0428
2	42.722	BB	1.6323	1.65310e4	154.74957	49.9572
Totals :				3.30904e4	395.80447	

Enantiomerically enriched (97% ee)

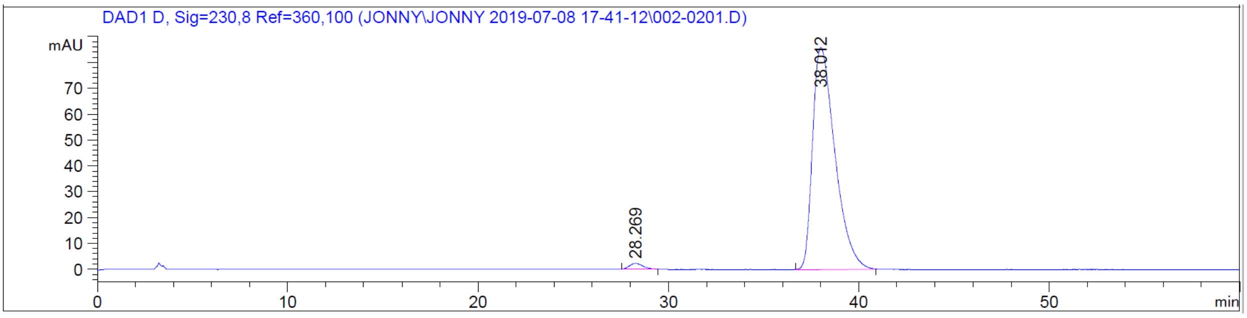

Signal 4: DAD1 D, Sig=230,8 Ref=360,100

Peak #	RetTime [min]	Type	Width [min]	Area [mAU*s]	Height [mAU]	Area %
1	28.269	BB	0.6852	114.13937	2.25260	1.6012
2	38.012	BB	1.2526	7014.10840	85.90630	98.3988
Totals :				7128.24776	88.15890	

(S)-S-methyl O-((2-methyl-4-oxocyclohex-2-en-1-yl)methyl) carbonodithioate (4d)

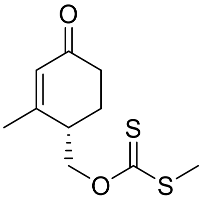

(Chiralpak AS-H, hexane/isopropanol = 70/30, 1 mL/min)

Racemic

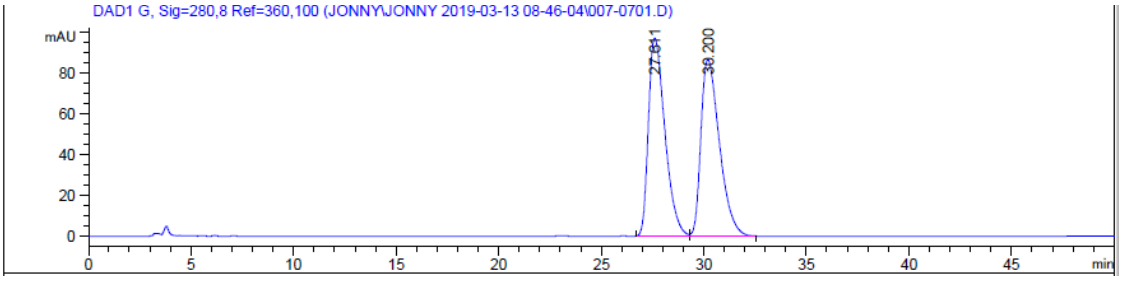

Signal 7: DAD1 G, Sig=280,8 Ref=360,100

Peak #	RetTime [min]	Type	Width [min]	Area [mAU*s]	Height [mAU]	Area %
1	27.611	BV	0.8598	5412.90186	96.88644	49.9306
2	30.200	VB	0.9676	5427.95654	86.56201	50.0694

Totals : 1.08409e4 183.44846

Enantiomerically enriched (99% ee)

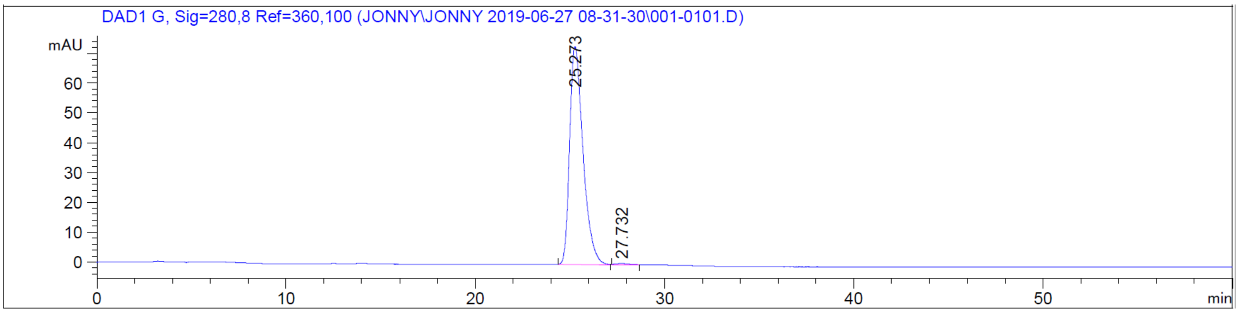

Signal 7: DAD1 G, Sig=280,8 Ref=360,100

Peak #	RetTime [min]	Type	Width [min]	Area [mAU*s]	Height [mAU]	Area %
1	25.273	BB	0.7540	3598.72388	73.15093	99.4047
2	27.732	BB	0.5973	21.55198	4.41689e-1	0.5953

Totals : 3620.27586 73.59262

(3aR,7aS)-3a-methyl-3-thioxohexahydroisobenzofuran-5(3H)-one (4e)

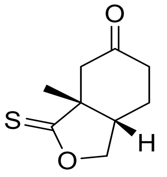

(Chiralpak AS-H, hexane/isopropanol = 70/30, 1 mL/min)

Racemic

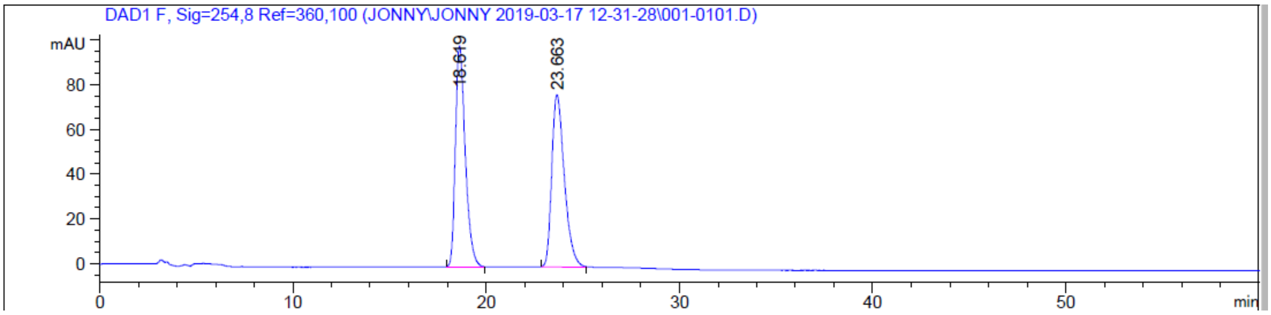

Signal 6: DAD1 F, Sig=254,8 Ref=360,100

Peak #	RetTime [min]	Type	Width [min]	Area [mAU*s]	Height [mAU]	Area %
1	18.619	BB	0.5409	3489.74048	98.61083	50.0184
2	23.663	BB	0.6929	3487.16846	76.91927	49.9816
Totals :				6976.90894	175.53011	

Enantiomerically enriched (97% ee)

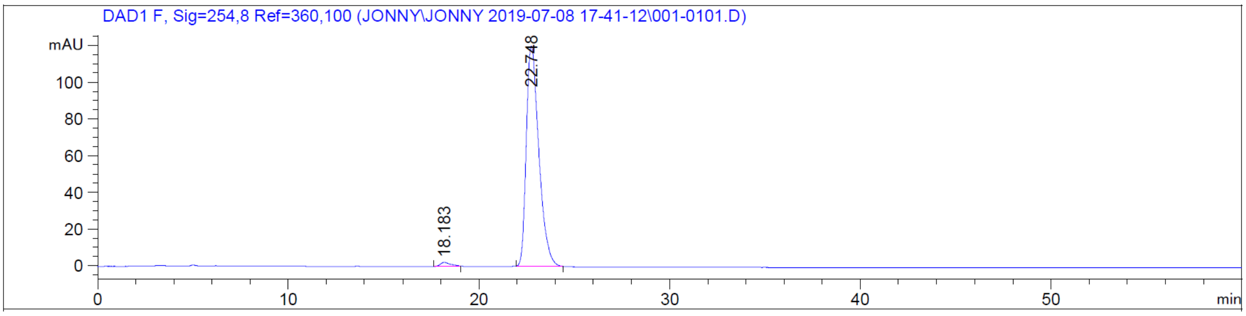

Signal 6: DAD1 F, Sig=254,8 Ref=360,100

Peak #	RetTime [min]	Type	Width [min]	Area [mAU*s]	Height [mAU]	Area %
1	18.183	BB	0.5695	86.40878	2.18456	1.5474
2	22.748	BB	0.6980	5497.60156	120.10945	98.4526
Totals :				5584.01035	122.29401	

tert-Butyl (*2aS,2a1S*)-6-hydroxy-2a1-methyl-5-oxo-2a,2a1,3,4,5,7-hexahydrobenzo[cd]indole-1(2H)-carboxylate (4f)

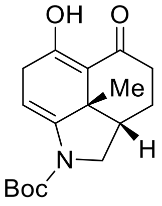

(Chiralpak AS, hexane/isopropanol = 99/1, 1 mL/min)

Racemic

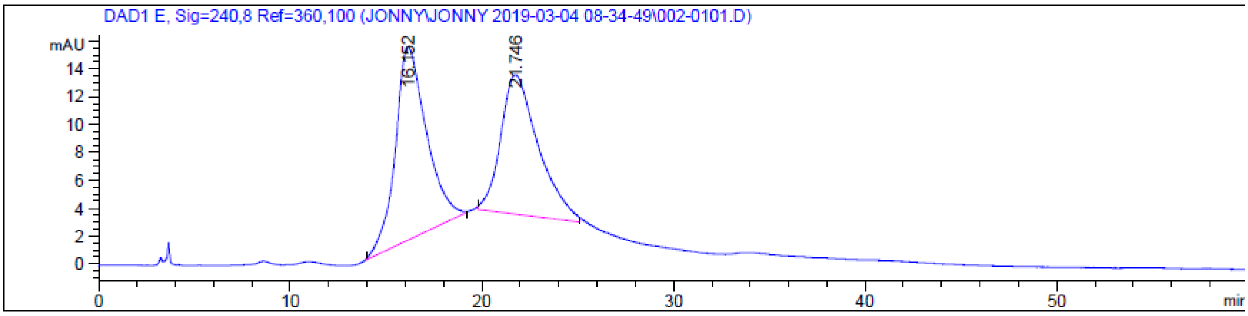

Signal 5: DAD1 E, Sig=240,8 Ref=360,100

Peak #	RetTime [min]	Type	Width [min]	Area [mAU*s]	Height [mAU]	Area %
1	16.152	BB	1.5692	1564.09851	13.89599	53.3592
2	21.746	BB	1.8339	1367.16504	10.00704	46.6408
Totals :				2931.26355	23.90304	

Enantiomerically enriched (98% ee)

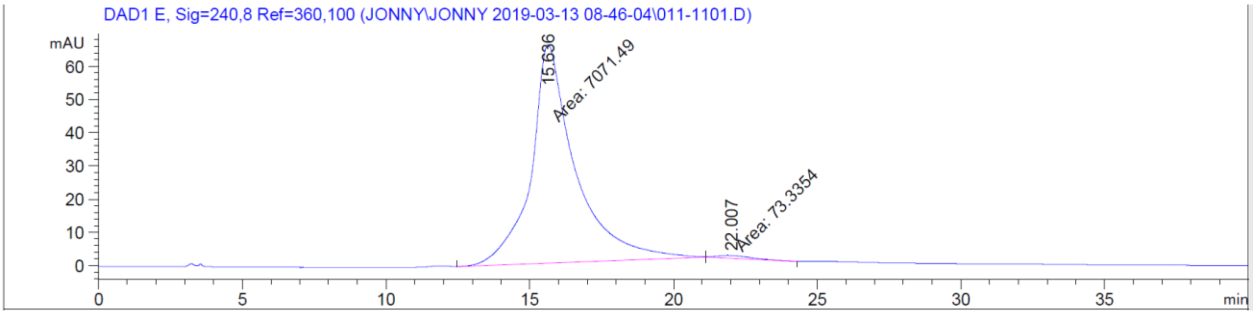

Signal 5: DAD1 E, Sig=240,8 Ref=360,100

Peak #	RetTime [min]	Type	Width [min]	Area [mAU*s]	Height [mAU]	Area %
1	15.636	MM	1.7908	7071.49365	65.81398	98.9736
2	22.007	MM	1.3783	73.33542	8.86800e-1	1.0264
Totals :				7144.82907	66.70078	

2.4 Computational details

Computational Methods:

Geometry optimizations were carried out with *Gaussian*[18] at the ONIOM(M06-2X/6-31G(d,p):UFF) level of theory and all stationary points were confirmed by vibrational analysis at the same level. M06-2X+D3/def2-TZVP single point energies[19] were then evaluated including an SMD description of diethyl ether.[20] Quasi-rigid-rotor harmonic oscillator Gibbs free energies were evaluated at 298K with *Goodvibes*[21a-b] using vibrational scaling factors: 0.98 for the high-level layer[22] and 0.87 for the low-level layer.[23] Molecular graphics were prepared using *Pymol*.[24]

Reaction mechanism:

Transition structures (TSs) were located for substrate **1a** undergoing successive **α**-deprotonation and ɣ-reprotonation by BIMP catalyst **3i**, resulting in the Gibbs energy profile shown in the figure below. The reprotonation TSs are higher in energy, making this the rate- and enantio-determining step.[25] An extensive search of different conformations was made for the ɣ-reprotonation TS structures (*vide infra*), but not for the **α**-deprotonation. It is possible that the deprotonation step could proceed through more stable TSs, but it will only reinforce the observation that the rate and selectivity of the reaction was determined during the ɣ-reprotonation. As it is discussed in the manuscript, this is consistent with the experimental observations with D-labelled substrates.

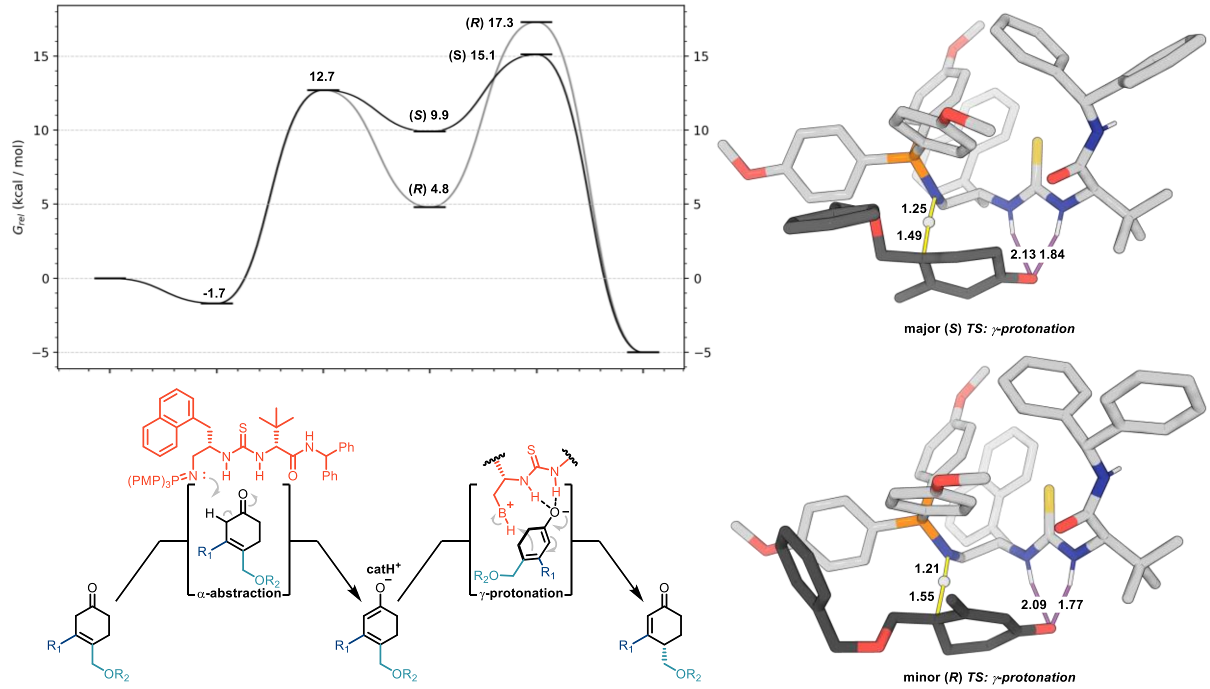

The most stable major and minor transition structures in the enantiodetermining step are shown (bond lengths in Å).

Gibbs energy profile for the reaction of **1a** catalysed by **3i**, showing the **α**-deprotonation and ɣ-reprotonation steps for (*R*) and (*S*) products.

Once that it was established that the ɣ-reprotonation determines the selectivity of the reaction, the contribution of each TSs to the product distribution was calculated by a Maxwell Boltzmann distribution using its calculated ΔΔG. These contributions were added for all TSs yielding (*R*) and (*S*) products, respectively, which allows comparison of the enantiomeric excesses determined experimentally and by the calculations. We found a good agreement in both cases: 98% for **1a** (exp: 99%) and 87% for **1h** (exp: 92%)

Conformational analysis of the ɣ-reprotonation step:

We performed a systematic conformational analysis of competing TSs, including varied substrate ring conformations and rotations about single bonds, different catalyst conformations and different interactions between the catalyst and the substrates:

- Conformation of the -OBn substituent of the substrate:

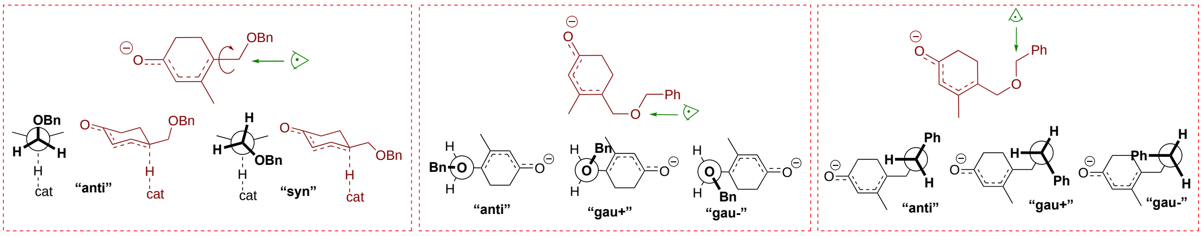

- "Chair" or "Boat" conformation of the substrate ring:

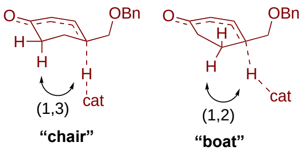

- "Activation mode" of the catalyst: the substrate is re-protonated from the R-N=P(PMP)$_3$ group of from the thiourea group:

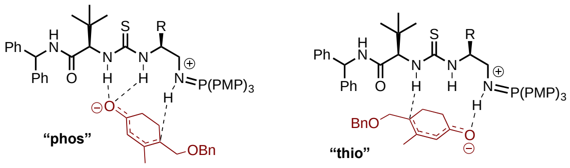

The protonation from the thiourea group was only considered for combination of catalyst **3i** and substrate **1a**. The energy penalty for TSs corresponding to the “thio” protonation is for all cases (for the different conformations of the substrate) larger than 14 kcal/mol. Therefore, the “thio” reprotonation mode was not studied for substrate **1h**.

- Conformations of the catalyst: Two possible conformations of the backbone of the catalyst were considered for the combination of catalyst **3i** and substrate **1a**:

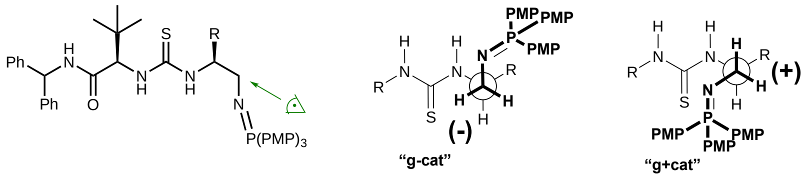

In all TSs (with different substrate conformations) the energy penalty of the conformation (-) is higher than 5 kcal/mol; therefore, the study was not repeated for substrate **1h**.

TSs corresponding to “thio” reprotonation mode and (-) catalyst conformation were not sought, since they correspond to the combination of two destabilizing factors.

-Conformations of the catalyst naphthyl ring: There are 6 possible conformations of the naphthyl group. To reduce the number of calculations, initially one of them (3a) was considered, and only in those cases in which the energy was less than 4 kcal/mol (with respect to the most stable TSs) the rest of the conformations were included in the search. In those cases in which the 6 conformations were considered, the energy spans less than 2.5 kcal/mol, indicating that the threshold used to filter out the necessity of including all conformations is appropriate.

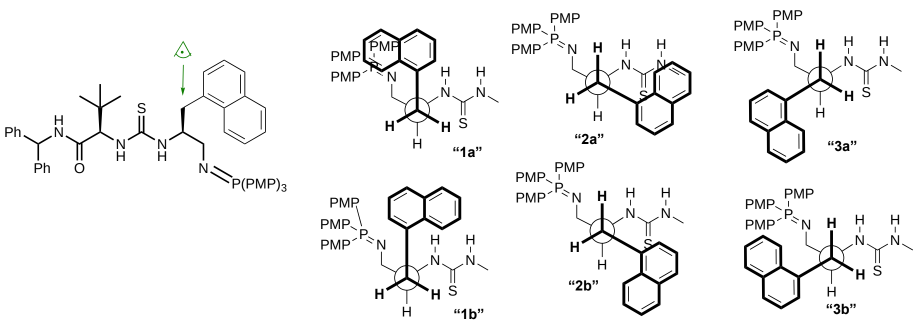

To differentiate this large number of TSs, in following sections (see supplementary computational file) they are named according to a scheme that includes information of the substrate, absolute configuration of the product and the conformation of substrate and catalyst. For example:

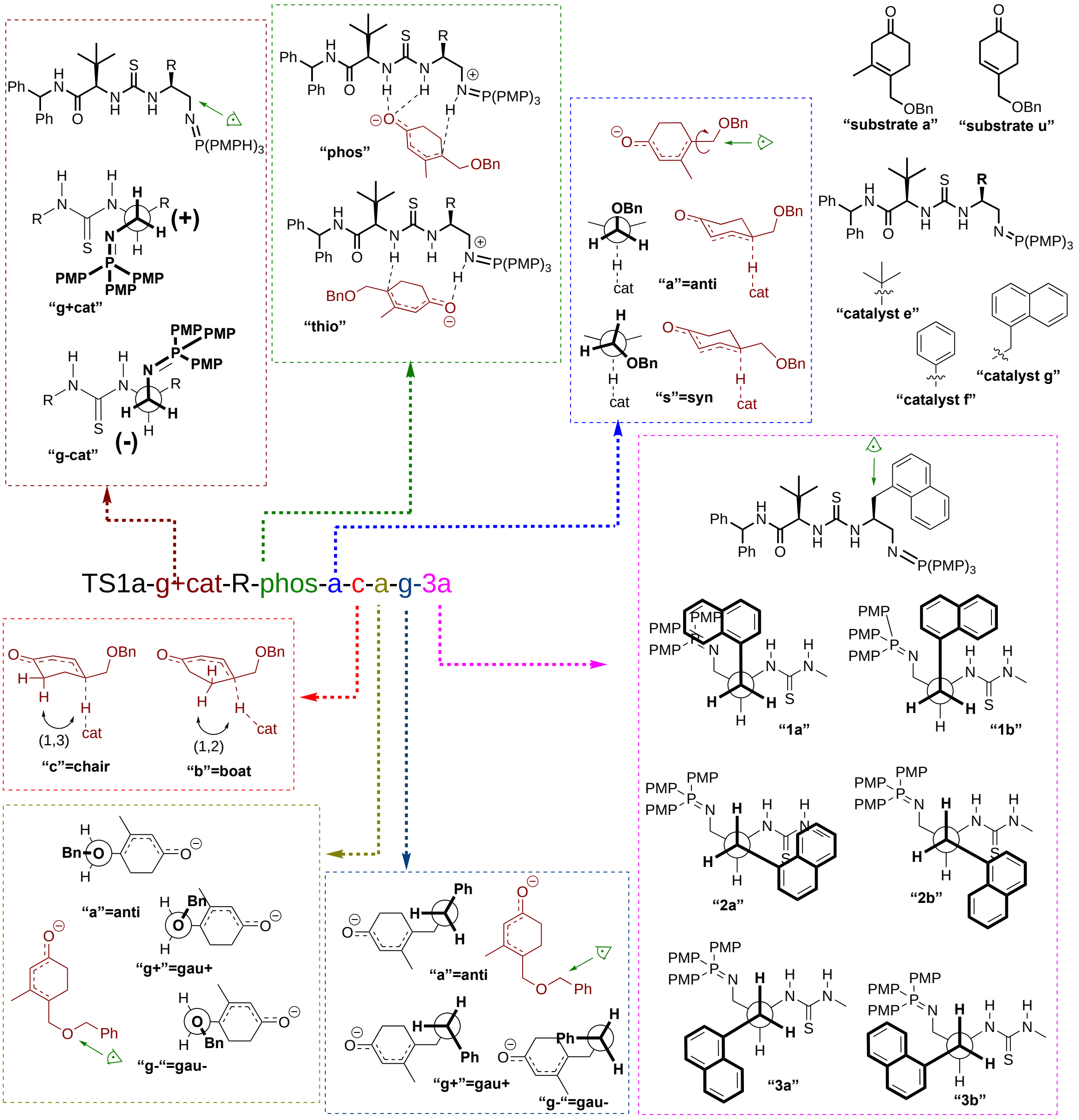

-Iminophosphorane pK_{BH+}:

Calculations of imnninophosphorane pK_{BH+} were carried out using a similar methodology described by Kaljurand and col.[26] for predicting the basicity of phosphazene bases (B3LYP/6-311+(d,p). In this case we employed SMD solvation model (solvent: acetonitrile) and made a RRHO treatment of the vibrational contributions to the free energy using Goodvibes defaults.[21a-b]

For the calculation of the ΔpK_{BH+} between methyl and PMP substituted BIMP, the Gibbs free energy difference of the following equilibrium was calculated:

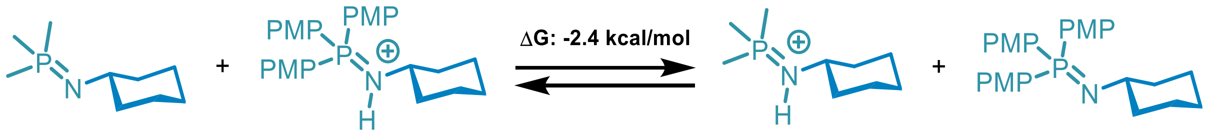

This difference corresponds at r.t. to a difference of 1.7 pK_a units. Based on previous experimental data this would suggest a theoretical iminophosphorane pK_{BH+} of approximately 26.7 with trimethylphosphine in acetonitrile.[27]

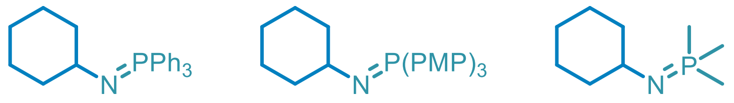

pK_{BH}+	22.7^{exp}	25.0^{exp}	26.7^{calc} (proposed pK_{BH}+)

Multivariate analysis: We performed a systematic conformational analysis of competing TSs, including varied substrate ring conformations and rotations about single bonds. In the preferred TSs, the thiourea binds the substrate oxygen while the phosphazene participates as proton acceptor and then donor. Alternative modes of N-H proton transfer from the catalyst to substrate from the (thio)urea were much higher in energy and are not expected to contribute to the observed reactivity.

After locating 112 structures within 10 kcal/mol of the most stable reprotonation TS, we reasoned that statistical modelling would enable us to identify geometric features that are correlated with conformational stability and hence enantioselectivity. H-bond distances, transferring N-H/C-H bond distances, cosine of dihedral angles, the dipole moment, distance between catalyst and substrate centers of mass (computed with xTB[28a-c]), and catalyst:substrate D3-dispersion energy (computed with pyDFTD3[29]) were compiled for each TS. Atom numbers below correspond to the numbering used for the dihedral angles. We extracted all heavy-atom dihedrals for the substrate, and all backbone dihedrals of the catalyst. We removed angle terms which were invariant across all of the conformers. The data is provided as a supporting CSV file.

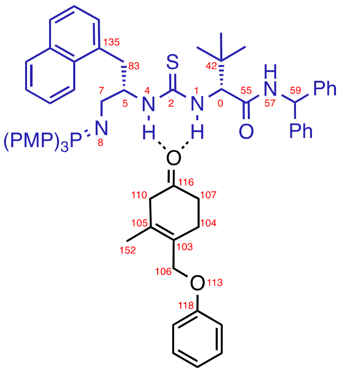

Firstly, we performed a decision tree analysis[30] (with JMP Pro 15.1.0) to understand qualitatively which geometric terms are dominant in determining conformational energies. This simple model shows that four of the dihedral angles in the substrate are influential, two ($f_{105\text{-}103\text{-}104\text{-}107}$, $f_{103\text{-}104\text{-}107\text{-}116}$) in the 6-membered ring, and two involving the orientation of the ether substituent ($f_{105\text{-}103\text{-}106\text{-}113}$, $f_{103\text{-}106\text{-}113\text{-}118}$).

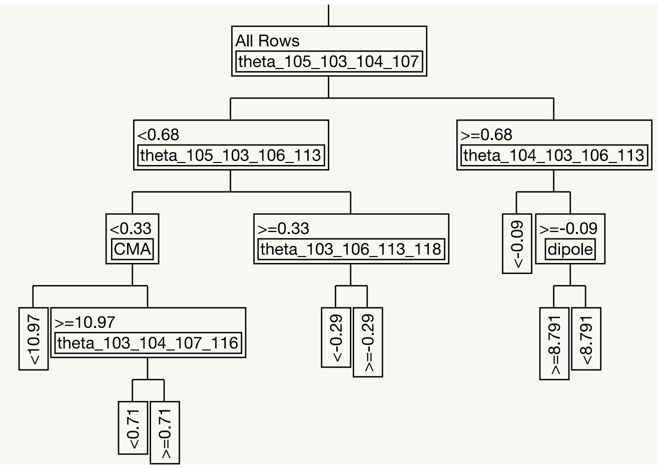

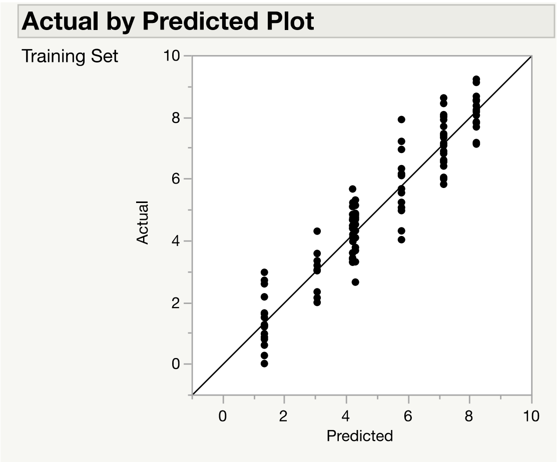


```
## theta_105_103_106_113   0.8629     0.1400   6.165 4.18e-08 ***
## theta_105_103_104_107   1.0431     0.1504   6.933 1.76e-09 ***
## theta_103_106_113_118   0.6647     0.1400   4.747 1.08e-05 ***
## theta_103_104_107_116   0.8976     0.1253   7.164 6.71e-10 ***
## CMA                    0.5812     0.1639   3.546 0.000708 ***
## theta_104_103_106_113   0.4181     0.1525   2.742 0.007772 **
## theta_2_4_5_7          -0.3399     0.1309  -2.596 0.011510 *
```

Crossvalidation

k-fold		SSE	RSquare
5	Folded	74.5446472	0.8815
	Overall	66.1540318	0.8948

Secondly, we performed a multivariate linear regression (MLR[31]) in *R* (version Version 1.2.5042) to generate a quantitative model to predict conformational energies. We used 70:30 train-test split of the data. Features were standardized and colinear features were removed prior to performing the regression. Parameters were retained which were statistically significant at the 0.1% level of confidence by ANOVA.[32]

Compared against DFT computed conformational energies, an MLR model with 7 terms resulted in an R^2 of 0.85 (compared against a training set of 78 conformers). Compared against the held-out test set of the remaining 34 conformers the Q^2 was 0.80. 5-fold cross-validation Q^2 was also 0.80. The code and output is given below.

We note that alternative models, such as a one-layer neural network give R^2 of 0.97 (70% train) and Q^2 of 0.93 (30% train) give exemplary quantitative predictive performance, but are harder to interpret.

R code:

```
require('dplyr')
require('cvq2')
require('e1071')
require('caret')
require('corrplot')
require('MASS')
require('tibble')
require('ggplot2')
require('cvq2')
```

Reading the data: the first column contains conformer names, the second the relative energies (kcal/mol). All other columns are geomrtric and electronic features/descriptors. These include dihedral angles, forming/breaking bond distances, hydrogen bond distances, dipole moment, catalyst:substrate D3-dispersion energy, catalyst:substrate center-of-mass distance.

```
data <- read.csv(paste(dataDirectory, 'conformers.csv', sep=""), header = TRUE)
confs <- data[2:ncol(data)]
```

Processing the data: we *standarize* ("scale") the features - each column is transformed into *z-scores*. We then find the correlation between each pair of features and remove colinear features. Where the magnitude of the Pearson correlation coefficient r is greater than 0.6 we only keep one of the features (of the two features, the one with the higher mean absolute correlation to all other variables is removed).

Plotting the correlations between all variables (including y-values). Having previously removed correlated features there are no intervariable correlations above 0.6. We also see that the y-value is not highly correlated against any singular feature and so a multivariate model is required.

```
# scale all columns except the first (the relative energies, i.e. the 'y-values')

confs[,-1] <- scale(confs[,-1])


## this computed the correlation between columns (apart from the y-values)

comat = cor(confs[,-1])

hc = findCorrelation(comat, cutoff=0.6)


## the features that are highly-correlated are now removed

hc = sort(hc) + 1

confs = confs[,-c(hc)]


# correlation plot using pearson algorithm
comat <- cor(confs)
comatr <- round(comat, 1)
sig <- cor.mtest(confs, conf.level = .95)
corrplot <- corrplot(comatr, p.mat = sig$p, sig.level = c(.001, .01, .05), pch.cex = .9,insig = "l
abel_sig", pch.col = "white",
type = "lower", tl.col = "black", tl.srt = 45, method="square", cl.align = "r", tl.cex = .5)
```

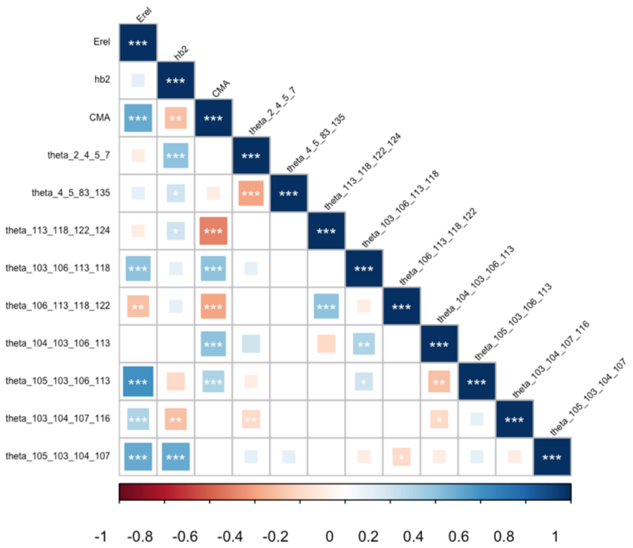

The data is randomly split into train (70%) and test (30%) sets. We will use the random seed 123 to make the partition reproducible. A multivariate linear regression is performed.

```
smp_size <- floor(0.70 * nrow(confs))
set.seed(123)
train_ind <- sample(seq_len(nrow(confs)), size = smp_size)
TRAIN <- confs[train_ind, ]
TEST <- confs[-train_ind, ]

MLR = lm(formula = Erel ~ ., data = TRAIN)
n <- nrow(na.omit(TRAIN))

Stepwise multivariate linear regression
null=lm(Erel~1, data=TRAIN)
full=lm(Erel~ . , data=TRAIN)
MLR =step(null, scope=list(lower=null, upper=full), direction="forward", trace = FALSE) #
TRUE to show all steps

train.predicted <- predict(MLR, TRAIN)

test.predicted <- predict(MLR, TEST)

summary(MLR)

## Call:
## lm(formula = Erel ~ theta_105_103_106_113 + theta_105_103_104_107 +
##     theta_103_106_113_118 + theta_103_104_107_116 + CMA + theta_104_103_106_113
+
##     theta_2_4_5_7, data = TRAIN)
##
## Residuals:
##      Min       1Q   Median       3Q      Max
## -2.31183 -0.63251  0.05817  0.56926  2.60499
##
## Coefficients:
##                       Estimate Std. Error t value Pr(>|t|)
## (Intercept)             5.0191     0.1152  43.584  < 2e-16 ***
## theta_105_103_106_113   0.8629     0.1400   6.165 4.18e-08 ***
## theta_105_103_104_107   1.0431     0.1504   6.933 1.76e-09 ***
## theta_103_106_113_118   0.6647     0.1400   4.747 1.08e-05 ***
## theta_103_104_107_116   0.8976     0.1253   7.164 6.71e-10 ***
## CMA                     0.5812     0.1639   3.546 0.000708 ***
## theta_104_103_106_113   0.4181     0.1525   2.742 0.007772 **
## theta_2_4_5_7          -0.3399     0.1309  -2.596 0.011510 *
## ---
## Signif. codes:  0 '***' 0.001 '**' 0.01 '*' 0.05 '.' 0.1 ' ' 1
##
## Residual standard error: 0.9951 on 69 degrees of freedom
## Multiple R-squared:  0.8526, Adjusted R-squared:  0.8355
```

```
## F-statistic:  49.9 on 8 and 69 DF,  p-value: < 2.2e-16
```

Cross Validation: Hold-out test-Set, leave-one-out, k-fold cross-validation

```
# Q2 (using test data)
MLR_fit = MLR[["call"]][["formula"]]

data_train <- TRAIN [ c(names(MLR$model)[ ])]
data_test <- TEST [ c(names(MLR$model)[ ])]

result_q2.ext<- q2(data_train, data_test)
result_q2.ext

## -- PREDICTION PERFORMANCE (model and prediction set available)
## #Elements Model Set:        78
## #Elements Prediction Set:   34
##
## mean (observed):    4.6766
## mean (predicted):   4.8543
## rmse (nu = 0):      0.9888
## q^2:                0.8011

# Leave One Out Cross Validation
subset_all <- confs[ c(names(MLR$model)[ ])]
result_all.Q2.loo<- looq2(subset_all, MLR_fit)
result_all.Q2.loo

## -- PREDICTION PERFORMANCE (cross validation)
## #Runs:              1
## #Groups:            112
## #Elements Training Set:  111
## #Elements Test Set:      1
##
## mean (observed):    4.9651
## mean (predicted):   4.9637
## rmse (nu = 1):      1.0302
## q^2:                0.8160

# K-fold Cross Validation (K=5)
K = 5 # EDIT to amount of K-fold

result_all.Q2.kfold <- cvq2( subset_all, MLR_fit, nFold = K)
result_all.Q2.kfold

## -- PREDICTION PERFORMANCE (cross validation)
## #Runs:              1
## #Groups:            5
## #Elements Training Set:  89 (+1)
```

```
## #Elements Test Set:      23 (-1)
##
## mean (observed):     4.9651
## mean (predicted):    4.9452
## rmse (nu = 1):       1.0863
## q^2:                 0.7969
```

Plot the model-predicted against actual values

```
#train data set
train.actual = TRAIN$Erel
train.frame = tibble(train.predicted, train.actual)
#train.frame

#test data set
test.actual = TEST$Erel
test.frame = tibble(test.predicted, test.actual)

# plotting function
ggplotRegression <- function (MLR) {

  q = ggplot(train.frame, aes( x = train.actual , y = train.predicted , color="Train" ) ) +
    geom_point() +
    xlab("DFT Relative Energy (kcal/mol)") +
    ylab("Predicted Relative Energy (kcal/mol)") +
    ggtitle( paste("Multivariate Regression Model: ", signif(nrow(TRAIN)/nrow(confs)*100, 2
), "% Train: ", signif(nrow(TEST)/nrow(confs)*100, 2), "% Test" ) ) +
    theme_classic() +
    theme(plot.title = element_text(hjust = 0.5)) +
    labs( subtitle =  MLR[["call"]][["formula"]])  +
    stat_smooth(method = "lm", col= "black") +
    labs(caption = paste("R2 = ",signif(summary(MLR)$r.squared, 2))  )
  q + geom_point(data=test.frame, aes(x=test.actual, y=test.predicted, color="Test"))
}

qq <- ggplotRegression(MLR); qq
```

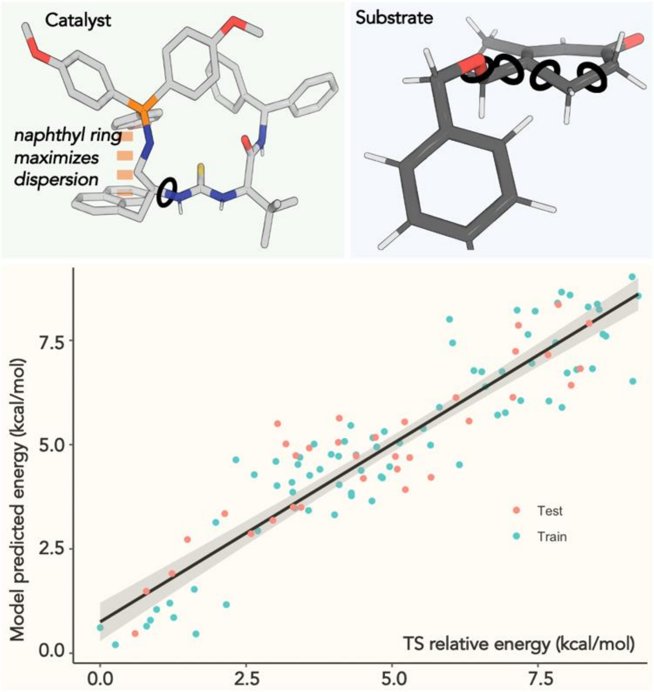

Interpretation: One dihedral angle in BIMP catalyst **3i** have a significant influence on TS conformational energies. An intramolecular dispersion interaction between the naphthyl ring and one of the P-aryl groups favors the conformation shown. Rotation of this dihedral angle (marked) on the LHS of the catalyst structure away from the conformation shown causes an increase in energy. These conformational features are conserved in the catalyst across the more stable (*R*) and (*S*) transition structures. In common with the decision tree analysis, the same dihedrals in the substrate are significant in their influence on the relative energy. The exocyclic dihedrals in the substrate are decisive in terms of enantioselectivity and differ between (*R*) and (*S*) structures.

3. References

(1) (a) Juliá-Hernández, F.; Arcas, A.; Vicente, J. Pd(II)-Catalyzed Deprotection of Acetals and Ketals Containing Acid Sensitive Functional Groups. *Tetrahedron Lett.* **2014**, *55*, 1141. (b) Garcia, A.; Hernandez, J. COMPLEJO ORGANOMETALICO DE PD (II) Y SU USO EN LA HIDROLISIS DE CETALES Y ACETALES **2012**, ES 2372457.

(2) Jones G. B.; Wright J. M.; Hynd G.; Wyatt J. K.; Warner P. M.; Huber R. S.; Li A.; Kilgore M. W.; Sticca R. P.; Pollenz R. S. Oxa-Enediynes: Probing the Electronic and Stereoelectronic Contributions to the Bergman Cycloaromatization. *J. Org. Chem.*, **2002**, 67, 5727.

(3) Ishikawa, H.; Bondzic, B. P.; Hayashi, Y. Synthesis of (-)-Oseltamivir by Using a Microreactor in the Curtius Rearrangement. *Eur. J. Org. Chem.* **2011**, *2011*, 6020.

(4) Iyer, A.; Jockusch, S.; Sivaguru, J. A Photo-Auxiliary Approach – Enabling Excited State Classical Phototransformations with Metal Free Visible Light Irradiation. *Chem. Commun.* **2017**, *53*, 1692.

(5) Murar, C. E.; Thuaud, F.; Bode, J. W. KAHA Ligations That Form Aspartyl Aldehyde Residues as Synthetic Handles for Protein Modification and Purification. *J. Am. Chem. Soc.* **2014**, *136*, 18140.

(6) Kuttner, J. R.; Warratz, S.; Hilt, G. Straightforward Synthesis of Nonconjugated Cyclohex-3-Enone and Conjugated 4-Methylenecyclohex-2-Enone Derivatives. *Synthesis (Stuttg).* **2012**, *44*, 1293.

(7) Zhang, H.; Li, C.; Xie, G.; Wang, B.; Zhang, Y.; Wang, J. Zn(II)- or Rh(I)-Catalyzed Rearrangement of Silylated [1,1′-Bi(Cyclopropan)]-2′-En-1-Ols. *J. Org. Chem.* **2014**, *79*, 6286.

(8) (a) Smith, A. B.; Nolen, E. G.; Shirai, R.; Blase, F. R.; Ohta, M.; Chida, N.; Hartz, R. A.; Fitch, D. M.; Clark, W. M.; Sprengeler, P. A. Tremorgenic Indole Alkaloids. 9. Asymmetric Construction of an Advanced F-G-H-Ring Lactone Precursor for the Synthesis of Penitrem D. *J. Org. Chem.* **1995**, *60*, 7837. (b) Barco, A.; Benetti, S.; Bianchi, A.; Casolari, A.; Pollini, G. P.; Romagnoli, R.; Spalluto, G.; Zanirato, V. Enantioselective Synthesis of the Hexahydronaphthalene Nucleus of (−)-Compactin from Ethyl (1R,2S)-2-Methyl-4-Oxocyclohexanecarboxylate and 2-(3-Nitropropyl)-1,3-Dioxolane as Four Carbon Bifunctional Annelating Agent. *Tetrahedron* **1994**, *50*, 11743.

(9) Yamaguchi, S.; Ura, M.; Izuta, S.; Okamoto, A. Chemically Activatable Alkyne Tag for Low PH-Enhanced Molecular Labeling on Living Cells. *Bioconjugate. Chem.* **2016**, *27*, 1976.

(10) (a) Stork, G.; Tang, P. C.; Casey, M.; Goodman, B.; Toyota, M. Regiospecific and Stereoselective Synthesis of (±)-Reserpine and (-)-Reserpine. *J. Am. Chem. Soc.* **2005**, *127*, 16255. (b) Kozmin, S. A.; Rawal, V. H. Chiral Amino Siloxy Dienes in the Diels-Alder Reaction: Applications to the Asymmetric Synthesis of 4-Substituted and 4,5-Disubstituted Cyclohexenones and the Total Synthesis of (-)-α-Elemene. *J. Am. Chem. Soc.* **1999**, *121*, 9562. (c) Jiang, G.-J.; Fu, X.-F.; Li, Q.; Yu, Z.-X. Rh(I)-Catalyzed [5 + 1] Cycloaddition of Vinylcyclopropanes and CO for the Synthesis of α,β- and β,γ-Cyclohexenones. *Org. Lett.* **2012**, *14*, 692.

(11) Buzas, A.; Gagosz, F. Gold(I) Catalyzed Isomerization of 5-En-2-Yn-1-Yl Acetates: An Efficient Access to Acetoxy Bicyclo[3.1.0]Hexenes and 2-Cycloalken-1-Ones. *J. Am. Chem. Soc.* **2006**, *128*, 12614.

(12) Li, Y.; Li, J. X.; Ouyang, X. H.; Wang, Q. A.; Li, J. H. Manganese-Catalyzed Intermolecular Oxidative Annulation of Alkynes with γ-Vinyl Aldehydes: An Entry to Bridged Carbocyclic Systems. *Org. Lett.* **2017**, *19*, 6172.

(13) Oriyama, T.; Kobayashi, Y.; Noda, K. Chemoselective and Practical Deprotection of Alkyl Trialkylsilyl Ethers in the Presence of Aryl Trialkylsilyl Ethers by a Catalytic Amount of Sc(OTf)3. *Synlett* **1998**, *10*, 1047.

(14) Iwasa, S.; Yamamoto, M.; Kohmoto, S.; Yamada, K. Synthesis of Thionolactones from Homoallylic Xanthates. *J. Chem. Soc. Perkin Trans. 1* **1991**, *0*, 1173.

(15) Chong, H.-S.; Song, H. A.; Dadwal, M.; Sun, X.; Sin, I.; Chen, Y. Efficient Synthesis of Functionalized Aziridinium Salts. *J. Org. Chem.* **2010**, *75*, 219.

(16) Farley, A. J. M.; Sandford, C.; Dixon, D. J. Bifunctional Iminophosphorane Catalyzed Enantioselective Sulfa-Michael Addition to Unactivated α-Substituted Acrylate Esters. *J. Am. Chem. Soc.* **2015**, *137*, 15992.

(17) Whalen, D. L.; Weimaster, J. F.; Ross, A. M.; Radhe, R. Mechanism of the Acid- and General-Base-Catalyzed Conjugation of 3-Cyclopentenone and 3-Cyclohexenone; Electrostatic Effects in the Conjugation of β,γ-Unsaturated Ketones. *J. Am. Chem. Soc.* **1976**, *98*, 7319.

(18) Frisch, M. J. *et al.*, Gaussian 16, Revision B.01, Gaussian, Inc., Wallingford CT, 2016

(19) Zhao, Y.; Truhlar, D. The M06 suite of density functionals for main group thermochemistry, thermochemical kinetics, noncovalent interactions, excited states, and transition elements: two new functionals and systematic testing of four M06-class functionals and 12 other functionals. *Theor. Chem. Acc.* **2008**, *120*, 215.

(20) Marenich, A.V.; Cramer, C. J.; Truhlar, D. G. Universal Solvation Model Based on Solute Electron Density and on a Continuum Model of the Solvent Defined by the Bulk Dielectric Constant and Atomic Surface Tensions. *J. Phys. Chem. B.* **2009**, *113*, 6378.

(21) (a) Grimme, S. Supramolecular Binding Thermodynamics by Dispersion-Corrected Density Functional Theory. *Chem. Eur. J.* **2012**, *18*, 9955; (b) I. Funes-Ardoiz, R. S. Paton, (**2016**) GoodVibes v3.0.1. http://doi.org/10.5281/zenodo.595246

(22) Kesharwani, M. K.; Brauer, B.; Martin, J. M. Frequency and Zero-Point Vibrational Energy Scale Factors for Double-Hybrid Density Functionals (and Other Selected Methods): Can Anharmonic Force Fields Be Avoided? *J. Phys. Chem. A.* **2015**, *119*, 1701.

(23) Simón, L.; Paton, R. S. The True Catalyst Revealed: The Intervention of Chiral Ca and Mg Phosphates in Brønsted Acid Promoted Asymmetric Mannich Reactions. *J. Am. Chem. Soc.* **2018**, *140*, 5412.

(24) The PyMOL Molecular Graphics System, Version 1.8 Schrödinger, LLC

(25) Peng, Q.; Duarte, F.; Paton, R. S. Computing Organic Stereoselectivity – from Concepts to Quantitative Calculations and Predictions. *Chem. Soc. Rev.* **2016**, *45*, 6093.

(26) Kaljurand, I.; Saame, J.; Rodima, T.; Koppel, I.; Koppel, I. A.; Kogel, J. F.; Sundermeyer, J.; Kohn, U.; Coles, M. P.; Leito, I. Experimental Basicities of Phosphazene, Guanidinophosphazene, and Proton Sponge Superbases in the Gas Phase and Solution. *J. Phys. Chem. A.* **2016**, *120*, 2591.

(27) Núñez, M. G.; Farley, A. J. M.; Dixon, D. J. Bifunctional Iminophosphorane Organocatalysts for Enantioselective Synthesis: Application to the Ketimine Nitro-Mannich Reaction. *J. Am. Chem. Soc.* **2013**, *135*, 16348.

(28) (a) Grimme, S.; Bannwarth, C.; Shushkov, P. A Robust and Accurate Tight-Binding Quantum Chemical Method for Structures, Vibrational Frequencies, and Noncovalent Interactions of Large Molecular Systems Parametrized for All spd-Block Elements (Z = 1–86) *J. Chem. Theory Comput.* **2017**, *13*, 1989; (b) Bannwarth, C.; Ehlert, S.; Grimme, S. GFN2-xTB—An Accurate and Broadly Parametrized Self-Consistent Tight-Binding Quantum Chemical Method with Multipole Electrostatics and Density-Dependent Dispersion Contributions. *J. Chem. Theory Comput.* **2019**, *15*, 1652; (c) Pracht, P.; Caldeweyher, E.; Ehlert, S.; Grimme, S.; *ChemRxiv* **2019**, DOI: 10.26434/chemrxiv.8326202.v1

(29) Paton Lab. (2019, August 11). pyDFTD3: version 1.0 (Version v1.0). Zenodo. http://doi.org/10.5281/zenodo.3365475

(30) Banerjee, S.; Sreenithya, A.; Sunoj, R. B. Machine learning for predicting product distributions in catalytic regioselective reactions. *Chem. Chem. Phys.* **2018**, *20*, 18311.

(31) Santiago, C. B.; Guo, J.-Y.; Sigman, M. S. Predictive and mechanistic multivariate linear regression models for reaction development *Chem. Sci.* **2018**, *9*, 2398.

(32) Ardkhean, R.; Mortimore, M.; Paton, R. S.; Fletcher. S. P. Copper Catalysed Asymmetric Conjugate addition to β-substituted cyclopentenone forming quaternary centres with the aid of Quantitative Structure-Selectivity relationship. *Chem. Sci.* **2018**, *9*, 2628.
